# Analysis of SINE Families B2, Dip, and Ves with Special Reference to Polyadenylation Signals and Transcription Terminators

**DOI:** 10.3390/ijms22189897

**Published:** 2021-09-13

**Authors:** Nikita S. Vassetzky, Olga R. Borodulina, Ilia G. Ustyantsev, Sergei A. Kosushkin, Dmitri A. Kramerov

**Affiliations:** Laboratory of Eukaryotic Genome Evolution, Engelhardt Institute of Molecular Biology, Russian Academy of Sciences, 119991 Moscow, Russia; nvas@eimb.ru (N.S.V.); olgabor13@gmail.com (O.R.B.); ustian@mail.ru (I.G.U.); toki@mail.ru (S.A.K.)

**Keywords:** SINE, retroposon, retrotransposon, RNA polymerase III, transcription terminator, polyadenylation

## Abstract

Short Interspersed Elements (SINEs) are eukaryotic non-autonomous retrotransposons transcribed by RNA polymerase III (pol III). The 3′-terminus of many mammalian SINEs has a polyadenylation signal (AATAAA), pol III transcription terminator, and A-rich tail. The RNAs of such SINEs can be polyadenylated, which is unique for pol III transcripts. Here, B2 (mice and related rodents), Dip (jerboas), and Ves (vespertilionid bats) SINE families were thoroughly studied. They were divided into subfamilies reliably distinguished by relatively long indels. The age of SINE subfamilies can be estimated, which allows us to reconstruct their evolution. The youngest and most active variants of SINE subfamilies were given special attention. The shortest pol III transcription terminators are TCTTT (B2), TATTT (Ves and Dip), and the rarer TTTT. The last nucleotide of the terminator is often not transcribed; accordingly, the truncated terminator of its descendant becomes nonfunctional. The incidence of complete transcription of the TCTTT terminator is twice higher compared to TTTT and thus functional terminators are more likely preserved in daughter SINE copies. Young copies have long poly(A) tails; however, they gradually shorten in host generations. Unexpectedly, the tail shortening below A_10_ increases the incidence of terminator elongation by Ts thus restoring its efficiency. This process can be critical for the maintenance of SINE activity in the genome.

## 1. Introduction

Short INterspersed Elements (SINEs) were discovered four decades ago. Initially, highly repetitive DNA regions dispersed in the human (Alu elements) and mouse (B1 and B2 elements) genomes were found [1,2]. After a while, the nucleotide sequences of these three SINEs were determined in a small number of their genomic copies [3,4,5,6]. As time went on, dozens of SINE families were described in the genomes of various organisms [7], and great progress has been made in understanding the origin, structure, transcription, and amplification of these mobile genetic elements (reviewed in [8,9,10]). Human and primate Alu [11,12] were studied more thoroughly; thus, analysis of other SINEs can shed light on both the general and specific properties of SINE families.

SINEs are nonautonomous as they encode no proteins and their spread in the genome (amplification) relies on the reverse transcriptase of Long Interspersed Elements (LINEs). SINEs also feature short length (usually 150–300 bp) and transcription by RNA polymerase III (pol III) as a result of their origin from small cellular RNAs synthesized by pol III. There are three types of such RNA ancestors, 7SL RNA, 5S rRNA, and various tRNA species. Oddly enough, the first pair of discovered SINEs, primate Alu and rodent B1, emerged from 7SL RNA after deletion of its central region, which gave rise to the common ancestor of all such SINEs in rodents, primates, and tree shrews [13,14,15,16]. Apart from that, 7SL-derived SINEs emerged independently only in hagfish [17], i.e., this group is quite small. The group of SINEs originating from 5S rRNA is larger and includes several SINE families from fish, lizards, rodents, bats, and butterflies [7,18,19,20,21]. However, the majority of SINE families emerged by the reverse transcription of tRNA molecules [22]. Apart from the tRNA-derived *head*, most such SINEs have the central part (*body*) and *tail* [9]. Most mammalian SINEs have an A-rich tail, just like the 3′-end of LINE1 (L1), and the L1 enzyme requires an A-rich RNA terminus to prime its reverse transcription [23]; thus, the amplification of such SINEs depend on L1 [24]. Although the genomic sequences upstream of SINE copies can modulate their transcription [25,26], the pol III promoters reside within SINEs [9,12]. In tRNA- and 7SL-derived SINEs, these promoters include two 11-bp boxes (A and B) in the head spaced by 30–40 bp [7,27]. SINE sequences can be transcribed by RNA polymerase II as a part of gene introns; however, only the pol III-generated SINE transcripts can be templates for LINE reverse transcriptase that generate new genomic copies of SINEs.

Mammalian genomes commonly harbor one to four SINE families amounting to hundreds of thousands of copies each [7,9]. Most mammalian SINE families can be found in taxa of different ranks such as families or orders [9,10]. By all appearances, a newly emerged SINE family is inherited by all descendent species. Genomic SINE copies of a species can substantially vary (up to 35% nucleotide substitutions or even more in old SINEs). Such substitutions arise as the SINE family ages in evolution. In addition, SINE families can be divided into subfamilies of more similar sequences, which reflect the pattern of SINE evolution and amplification. Similar to biological species, SINEs diverge and their variants have different amplification potentials. Successful SINE subfamilies can decrease or lose the capacity for retrotransposition over time and their copies demonstrate significant sequence differences from each other and the consensus sequence. Younger successful subfamilies can take over. The copies of recent subfamilies demonstrate low sequence divergence; thus, the mean similarity of SINE copies is indicative of their age. SINE subfamilies have been thoroughly studied for Alu in humans and certain primates [11]. Amplification waves were demonstrated for this SINE (the most significant occurred 50 million years ago) [28], and currently, new genomic copies are rare and correspond to a few subfamilies [11].

Analysis of SINEs of different mammalian groups allowed us to reveal a specific structure of the A-rich tail in a number of SINE families [29]. There was a stretch of adenosine residues at the very 3′-end of such SINE copies. This oligo(A)-tail was usually preceded by a TCTTT or TTTT motif, a potential terminator of pol III transcription. Upstream of this terminator, there was one or, more commonly, several AATAAA motifs. These motifs allowed us to assign such SINEs to the class designated as T**^+^**. It should be noted that the identification of T**^+^** SINEs is not trivial since certain (or many) SINE copies lack these motifs (particularly, the terminator) as a result of nucleotide substitutions and indels in SINE sequences. At the same time, other L1-mobilized mammalian SINE families have neither pol III terminators nor AATAAA motifs; these are considered as T**^–^** SINEs [29]. T**^+^** SINEs were found only in mammals, and all 12 such SINE families seem to have an independent origin. All T**^+^** SINEs are tRNA-derived, while T**^–^** SINEs originate from tRNA, 7SL RNA, or 5S rRNA.

We assumed and experimentally confirmed that RNA of T**^+^** SINEs transcribed by pol III can be polyadenylated [30]. This was demonstrated for eight SINE families; however, most experiments were conducted on mouse B2, jerboa Dip, and bat Ves [31]. Pol III transcription starts at the first SINE nucleotide and stops at the terminator, after which a poly(A) tail up to 250 nt is synthesized at the 3′-end. Such polyadenylation requires the AAUAAA sequence in the vicinity of the 3′-end (as mentioned above, this motif is typical of T**^+^** SINEs). Previously, it was generally accepted that AAUAAA-dependent polyadenylation is limited to pol II-transcribed RNA (such as mRNA). However, pol lll-generated transcripts of T**^+^** SINEs can also be polyadenylated despite the difference in the structure of RNA polymerases and the mechanisms of their termination and transcription. Further analysis demonstrated that the polyadenylation (AATAAA) and transcription terminator signals are necessary but not sufficient for polyadenylation of SINE transcripts. T**^+^** SINEs (B2, Dip, and Ves) proved to have two regions indispensable for transcript polyadenylation [31]. The motif β is immediately downstream of box B, while the motif τ precedes the region of AATAAA repeats. In Dip and Ves, the τ motif is a long polypyrimidine region (up to 50 nt); similar TC-rich regions are found in four other T**^+^** families (Can, C, Eri-1, and Tal). Detailed analysis of the τ region in B2 demonstrated the critical role of the TGTA signal in this 18-nt sequence [32]. This signal in B2 RNA induces its polyadenylation via the interaction with the CFIm complex [32]. This factor interacts with the UGUA sequence in the vicinity of the AAUAAA signal and meditates the polyadenylation of almost half of human mRNA [33]. The nucleotide sequences within the β regions involved in the polyadenylation of B2 and Ves RNA were also identified [32]; they have limited similarity: ACCACATgg in B2 and aGGGCATGT in Ves (uppercase letters indicate positions critical for polyadenylation, while conserved nucleotides are underlined). The CATG(G/T) motif was also found in this region in other T**^+^** SINEs [31]. Protein factors interacting with β signals in B2 and Ves transcripts have not been identified yet.

Polyadenylation of SINE transcripts significantly increases their lifespan in the cell [30,34], apparently, due to the binding of the poly(A)-tail by PABP (*poly*(A)-binding protein), which is known to protect mRNA from 3′-exonuclease degradation [35,36]. A long poly(A) segment in a SINE RNA is strictly required for retrotransposition by L1 reverse transcriptase [37]. Very long poly(A) stretches are found at the end of young copies of Alu, B1, and ID [38,39]; since they lack the pol III terminators, these stretches are transcribed along with the SINE, and transcription terminates downstream at random Ts [9,12]. Such poly(A) stretches prime Alu reverse transcription during retrotransposition [37,40]. This mechanism is likely employed by other mammalian T**^–^** SINEs.

A different model was proposed for T**^+^** SINEs [9,29,30]. We believe that the primary transcripts of such SINEs lack the poly(A), but non-template synthesis provides a long poly(A)-tail due to the transcription terminators and polyadenylation signals. Later, this tail primes reverse transcription of SINE RNA. This model has an implicit problem. RNA synthesis by pol III stops at the terminator sequence, commonly, four or more thymidines [41,42]. As a result, the number of 3′-terminal Us in the resulting transcript is less than the number of Ts in the terminator (e.g., [43,44,45]). This should apply to both genes and T**^+^** SINEs transcribed by pol III. Consequently, each retrotransposition cycle shortens and disrupts the SINE terminator after a few or single cycles. There should be a mechanism restoring the terminator length in such SINEs. Previously [30], we proposed the synthesis of several U residues at the 3′-end of the SINE primary transcript. Indeed, there are cellular poly(U) polymerases [46] that add one or several Us to the 3′-ends of certain small RNAs such as U6 or microRNAs, which is required for their maturation or triggering RNA degradation [47,48].

Here, we conducted an extensive bioinformatics study of three SINE families (B2, Dip, and Ves) that belong to the T**^+^** class. B2 is found in three related rodent families: Muridae (mice, rats, and gerbils), Cricetidae (hamsters, voles, lemmings, and New World rats and mice), and Spalacidae (blind mole-rats, bamboo rats, mole-rats, and zokors) [49], which amount to 25% of mammals by the number of species. Dip SINE is typical of jerboas and birch mice (Dipodidae) [49]. Ves was initially described in the Vespertilionidae family of microbats [50] and later found in Phyllostomidae, Mormoopidae, and Molossidae bats [51,52]; it is thought to be present in all Yangochiroptera bats (18% of mammalian species). The whole-genome analyses of these SINE families allowed us to divide them into a variety of diverse subfamilies of different ages. Special attention was paid to the variation of the sequences of β, τ, and AATAAA signals, as well as pol III terminators and poly(A)-tails. Amazingly, young SINE copies have short terminators, while a substantial proportion of older ones have long effective terminators. This finding clearly contradicts the above hypothesis of terminator restitution by adding U residues to the 3′-end of SINE primary transcripts. Apparently, the terminators can somehow elongate in genomic SINE copies with time. Certain problems of transcription termination and polyadenylation of SINEs revealed by bioinformatics analysis of SINEs were addressed by biochemical methods.

## 2. Results

### 2.1. Overview of B2 SINE Family

**Figure 1:**
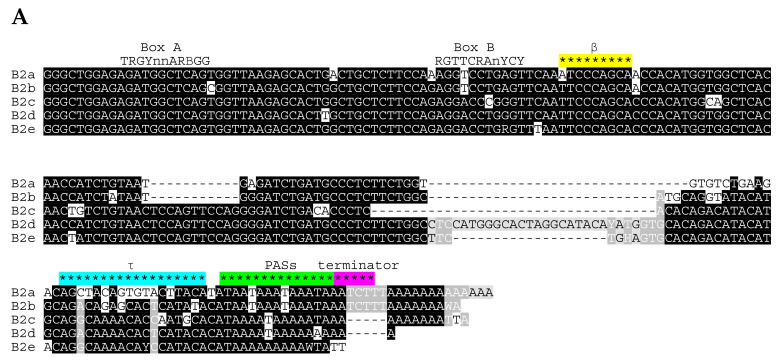

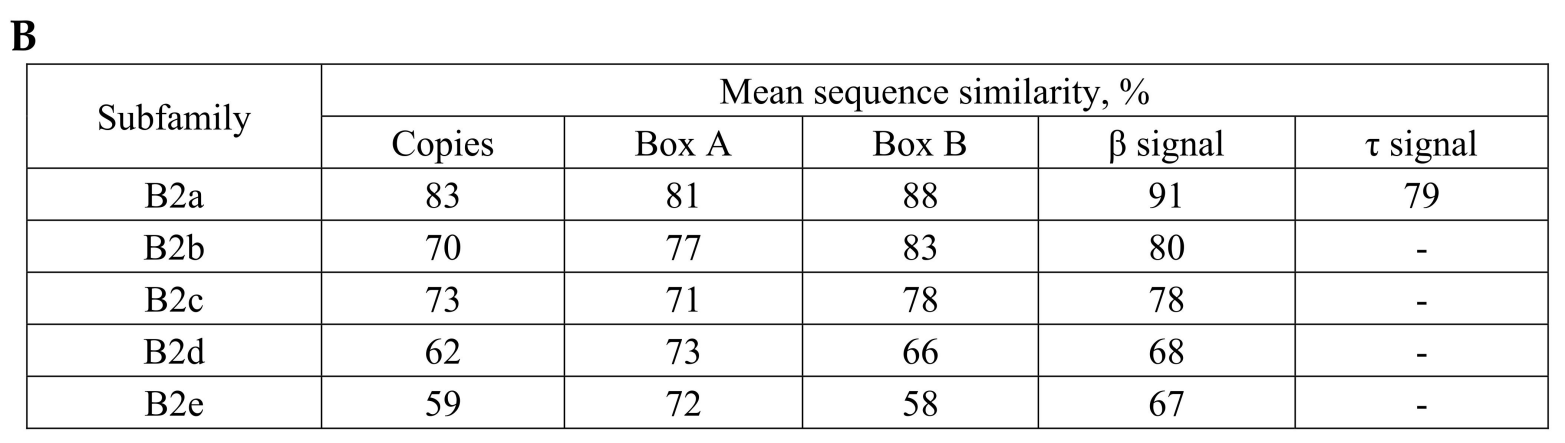
Subfamilies of SINE B2 in the mouse genome. (**A**) Consensus nucleotide sequences of five B2 subfamilies. Consensus box A and box B of pol III promoter are shown above the alignment. Positions of β and τ signals, PASs, and pol III transcription terminator are marked by asterisks. (**B**) Mean sequence similarity of B2 copies and their functional parts within subfamilies.

The study was initiated by an exhaustive computer search for B2 copies in the genome of *Mus musculus* (GRCm38/mm10). A total of 153,991 B2 sequences have been found. Surprisingly, many of them were in tandem B2-B2, B1-B2, and B2-B1 repeats (18,757, 6368, and 17,757 dimeric copies, respectively). These were excluded from further analysis since we do not know if they emerged by integration of one SINE into the tail of another one (head-to-tail) or are complex SINEs retrotransposed from dimeric templates similar to Alu. The remaining 92,352 full-length B2 copies were divided into five subfamilies (a, b, c, d, and e) distinguished by relatively long indels in addition to single nucleotide substitutions (Figure 1A). B2a and B2b share the diagnostic 11-bp deletion in the central part, while B2d and B2e have 28- and 9-bp insertions, respectively, in the 3′-part. The 27-bp region upstream of the B2a tail significantly differs from the corresponding regions in other B2 subfamilies.

The mean similarity of copies within subfamilies decreases in the following order: B2a, B2c, B2b, B2d, and B2e (Figure 1B). The high similarity of B2a copies (83%) and low similarity of B2d and B2e ones (62 and 59%, respectively) indicate that the B2a subfamily is relatively young. This is also indicated by the proportion of B2 copies with target site duplications (TSDs) from B2a to B2e (Table 1); conceivably, TSDs degrade and vanish with time. Being the youngest and most abundant (64% of all B2 copies), B2a corresponds to the classical mouse B2 [6]. Repbase Update (RU) [53] divides B2 into three subfamilies: mm1a and mm1t (distinguished by two nucleotide substitutions) and mm2 distinguished from them at 16 positions. The B2d subfamily corresponds to B3 in RU (suggesting it as a separate SINE family, which is misleading; actually, it was originally described as B2l, B2 long subfamily [54]).

The first 101 bp of the consensus sequences are very similar in the five B2 subfamilies. This particularly applies to A and B boxes of the pol III promoter as well as to the β signal. The latter differs in B2a and B2b by a single nucleotide from three other subfamilies (A and C, respectively). Experiments with single-nucleotide substitutions (but not 2–3-nt ones) within this region had no significant effect on the β signal efficiency in B2a [32]; accordingly, the corresponding sequence in B2c, B2d, and B2e should also be effective in polyadenylation of their transcripts. The τ signal of B2a is missing in other subfamilies (below, we test the capacity of the corresponding B2b region to induce the transcript polyadenylation). Commonly, the β and τ signals as well as boxes A and B are more conserved than the entire sequences of the subfamilies (Figure 1B and Figure S1), which is consistent with the functionality of these regions.

The polyadenylation signals (AATAAA) at the end of SINEs are typical for all subfamilies except B2e (Figure 1A). Most copies have two, three, or more such overlapping signals. In the consensus sequences of B2a and B2b, they are immediately followed by ТСТТТ, a potential terminator of pol III transcription (Figure 1A). No such terminator signal is found in the consensus sequences of B2c, B2d, and B2e, although some of their genomic copies can include this signal (the incidence of TCTTT in B2 subfamilies is indicated by the number of asterisks in Table 1). We believe that all these B2 subfamilies are T**^+^** SINEs; however, the terminator signals were lost with time in most copies of retrotranspositionally inactive B2c, B2d, and B2e.

A similar analysis of B2 was conducted for four more rodents with sequenced genomes: brown rat *Rattus norvegicus* (Muridae), Chinese hamster *Cricetulus griseus* (Cricetidae), deer mouse *Peromyscus maniculatus* (Cricetidae), and blind mole rat *Nannospalax galili* (Spalacidae) (Table 1). The rat genome harbors about 121,000 copies of monomeric B2, which can be divided into the same five subfamilies with a higher proportion of B2a compared to the mouse. The genomes of cricetids (*C.* *griseus* and *P.* *maniculatus*) lack B2a, i.e., this young and active B2 subfamily is limited to murids; B2e is also missing in cricetids. At the same time, the B2b subfamily is much more abundant in cricetids compared to murids. In *P. maniculatus* but not *C.* *griseus*, this active subfamily divides into three variants, B2b1, B2b2, and B2b3; their age and abundance decrease in the same order (Table 1). These variants are distinguished by deletions of different lengths in the region preceding the SINE tail (Figure S2). B2b and its variants have the TCTTT terminator signal. The B2c subfamily in cricetids can be divided into four variants (B2c1 to B2c4), although B2c2 is absent in *P. maniculatus* (Table 1). They are distinguished by deletions in the same region as in B2b (Figure S2). In *C. griseus*, B2c1 and B2c4, but not other B2c variants, often contain the TTTTT terminator. Similar to the above rodents, the genome of *N. galili* (spalacid) harbors B2d but has no other B2 subfamilies (Table 1). The *N. galili* B2d has two variants, major B2d1 and minor B2d2 (Figure S2). The B2d-specific 25-bp region (deleted in B2b) significantly varies between rodent families, which can indicate that it is under low selective pressure.

**Figure 2:**
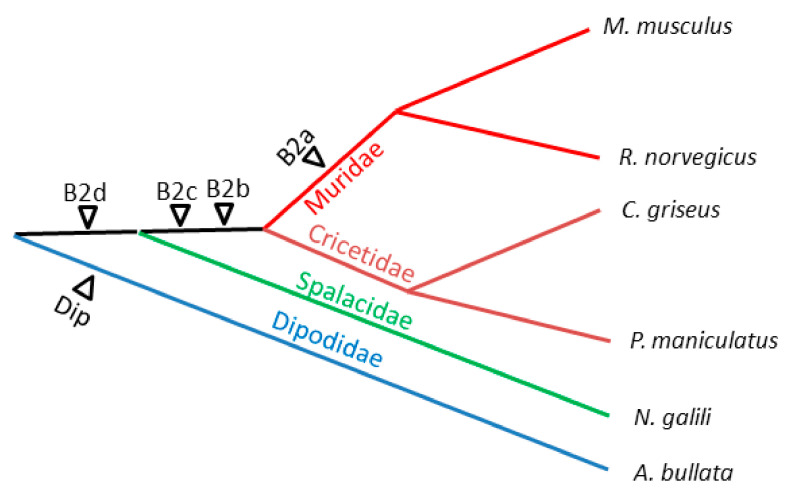
Origin of B2 subfamilies in evolution of Myomorpha rodents. The order of B2 subfamilies emergence is shown in accordance with their distribution among rodent families; the divergence of copies in the subfamilies (i.e., their age) was also taken into account. The position of B2e is not indicated on the tree since this minor murid subfamily could not be found in other rodents. Dip SINE is specific for Dipodidae, a basal family of Myomorpha.

The distribution of B2 subfamilies in rodents as well as the divergence of B2 copies within these subfamilies gives us an insight into the sequence of their emergence in rodent evolution. Apparently, B2d emerged first in the common ancestor of murids, cricetids, and spalacids (Figure 2). After the split of spalacids but before the split between murids and cricetids, B2d gave rise to B2c after the 35-bp deletion and to B2b after two (11- and 28-bp) deletions (Figure 1 and Figure 2). Finally, the substitution of the 27-bp sequence preceding the AATAAA region in B2b gave rise to the B2a subfamily (Figure 1 and Figure 2). Due to this substitution, B2a acquired the τ signal promoting the polyadenylation of its transcripts.

### 2.2. Analysis of Transcription Terminators and A-Tails in B2a Copies of Different Age

**Figure 3:**
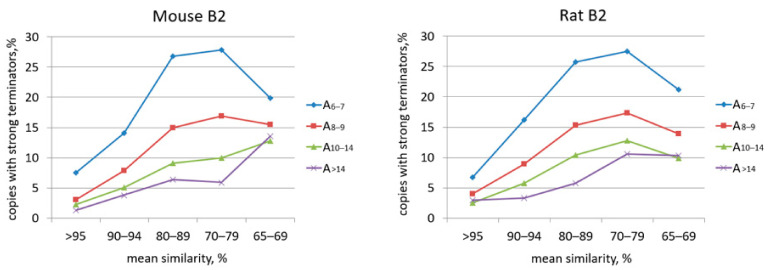
Dependence of the fraction of B2 copies with strong (efficient) terminators TCT_≥4_ and T_≥5_ on the mean similarity of these copies. Separate graphs for copies with different oligo(A)-tail lengths are shown.

Next, we focused on the analysis of potential pol III terminators, oligo(A) tails, and the age of B2 copies. Mouse and rat B2 genomic copies with putative terminators preceding poly(A) (76,598 and 100,208) were analyzed. B2 copies were divided into groups using three parameters: (i) mean sequence similarity, (ii) length of pure or almost pure poly(A) tail, and (iii) presence or absence of efficient TCT_>3_ terminators. Figure S3 indicates that younger copies (with a higher mean similarity) have longer A-tails than older B2s. The plots in Figure 3 illustrate as the proportion of B2 copies with efficient terminators changes with their age (mean similarity). Such plots were also generated for groups of B2 copies with A-tails of different lengths (Figure 3). Unexpectedly, the proportion of copies with efficient terminators increased as the mean similarity dropped to 70–79%. The incidence of such terminators also increased as the length of A-tails decreased (indicative of their aging) to peak in B2 copies with A_6–7_ tails and mean similarity of 70–79% (Figure 3). A similar pattern was observed for both mouse and rat B2s.

**Figure 4:**
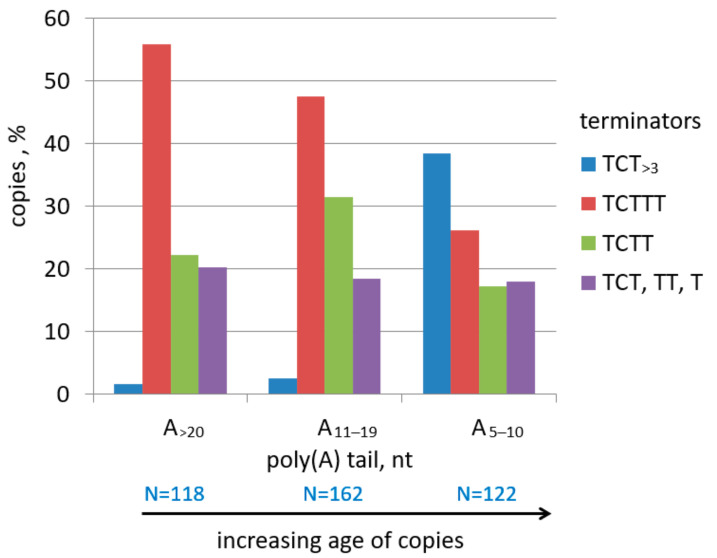
Distribution of different terminators among random mouse B2a copies with long (A_≥20_), medium (A_11–19_), and short (A_5–10_) oligo(A)-tails. The B2a sample has 97% copies with the C residue in their terminators. The numbers of B2a copies analyzed are shown as ‘N = number’.

To visualize the relationship between the lengths of the terminator and A-tail, we selected 402 random copies of mouse B2a, divided them into three groups based on the A-tail length (A_5–10_, A_11–19_, and A_≥20_), and evaluated the proportion of B2 copies with potential terminators of specific lengths (Figure 4). It turned out that 48–55% of copies with long tails (A_>10_) have moderately efficient terminator TCTTT and only 2–3% of copies have terminators with a longer T stretch. On the contrary, such efficient terminators become major (38%) in the group of B2 with A_5–10_ tails, while the proportion of copies with TCTTT decreases to 25%. These data indicate terminator elongation in a significant fraction of B2a copies with their A-tail shortening.

**Figure 5:**
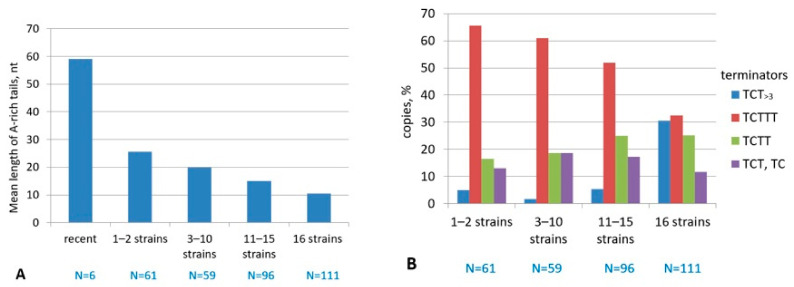
Analysis of tails of random B2a copies with different expansion in mouse strains. The age of B2a copies increases from left to right. The ‘recent’ B2 copies are the youngest as they have recently integrated into the genes and caused their mutations. (**A**) Mean length of A-rich tails in recent copies and B2a copies of four groups present in a different number of mouse strains. The numbers of B2a copies analyzed are shown as ‘N = number.’ (**B**) Distribution of different terminators among B2a copies from mice of four groups.

A different approach was applied to analyze the relationship between the terminator structure, A-rich tail length, and B2a age. In addition to the reference genome assembly (GRCm38/mm10, strain С57BL/6J), the genomes of 15 other mice are available [55]. These include three subspecies of *Mus musculus* (*M. m. musculus*, *M. m. domesticus,* and *M. m. castaneus*) as well as the closely related species *M. spretus*; other ones are classical laboratory mouse strains. A random sample of B2a copies from the С57BL/6J genome was divided into the following groups: (i) present in 1–2 strains, (ii) 3–10 strains, (iii) 11–15 strains, and (iv) all 16 strains (Tables S1–S4). We assumed that copies found in a greater number of strains correspond to older ones. In addition, six copies (Figure S4) induced mutations after recent B2 integrations into genes [56,57,58,59,60,61]; such copies are referred to as recent. Analysis of the A-tails (Figure 5A) and terminators (Figure 5B) was carried out in the age groups of B2. The mean A-tail length was 58 bp in the recent copies; it was significantly lower (25 bp) in the copies limited to 1–2 mouse strains and further decreased to 10 bp in the copies specific for 16 strains (Figure 5A). This indicates that A-tails shorten with the age of B2 copies and such shortening is particularly rapid in recently integrated copies.

Five of the recent B2 copies had the TCTTT terminator; and one, TCTTTT (Figure S4; these data were not included in the diagram in Figure 5B due to low sample numbers). The distribution of terminator patterns was similar in the three groups of relatively young B2 copies (1–2, 3–10, and 11–15 mouse lines): TCTTT was the most abundant (52–65%), longer terminators ТСТ_>3_ occurred in as few as 2–5% of copies, and the sequences in the rest of copies were incapable of transcription termination (TCTT, TCT, and TC) (Figure 5B). By contrast, the proportion of B2a copies with TCTTT decreased to 32% in the latter group (16 mouse lines including *M. spretus*) and that with efficient terminators TCT_>3_ increased to 30% (Figure 5B). The length of T stretch after TC in these terminators varied from 4 to 12 (6.1 on average); notice that 41% of efficient terminators in this sample had no С and were composed of T_4–10_ (T_5.4_ on average) (Table S4). The data obtained indicate an extension of the T stretch in the transcription terminators in many relatively old B2a copies (emerged before the split between *M. musculus* and *M. spretus*). Analysis of terminators in B2 copies in the same loci in different mouse lines demonstrates that they vary by the length of the T stretch extended by T residues and more frequently by A to T substitutions in the poly(A) tail (exemplified in Figure S5).

### 2.3. History of Retrotranspositional Activity of B2a Variants

**Figure 6:**
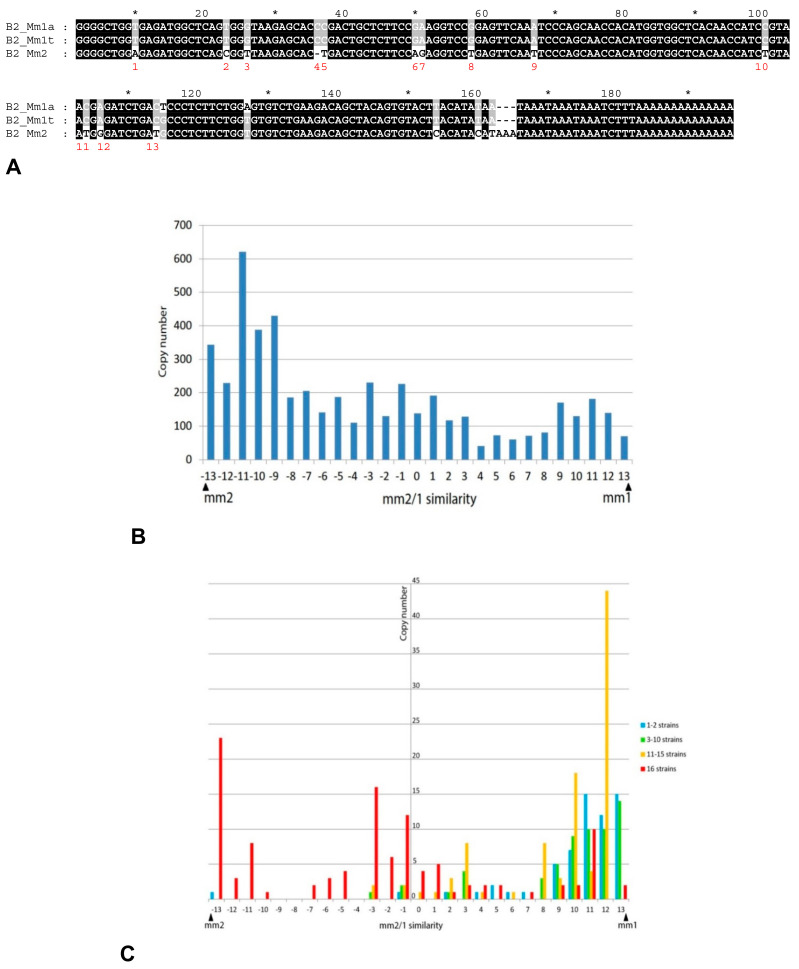
The analysis of mm1 and mm2 variants of the B2a subfamily. (**A**) Consensus nucleotide sequence of mm1a, mm1t, and mm2 variants. Thirteen diagnostic positions are numbered in red (below the alignment). (**B**) Distribution of 5000 B2a copies according to the degree of their similarity to mm1 and mm2 consensus sequences. (**C**) Distribution of B2a copies of four age groups according to the degree of their similarity to mm1 and mm2 consensus sequences. (For details, see text.).

As mentioned above, RU recognizes three B2 subfamilies: mm1a, mm1t (with only two different nucleotides), and mm2 (16 nucleotides different from mm1) (Figure 6A). All mouse B2 copies in the UCSC Genome Browser are annotated according to this classification. We tested the distribution of B2a copies among mm1 (the ‘a’ and ‘t’ variants were combined) and mm2. The diagnostic positions distinguishing mm1 and mm2 (13 positions were used and 3 ones in the 3′-part were excluded) were used to segregate 5000 random B2a copies. The results are presented in Figure S6, where the diagnostic nucleotides corresponding to mm1 and mm2 are marked in green and red, respectively. Only minor fractions of copies had the full sets of 13 characters (about 5 and 14.4% for mm1 and mm2, respectively). In the remaining copies, the diagnostic nucleotides were combined in different proportions. Figure 6B shows the distribution of the copies with different similarities to mm1 and mm2 throughout the analyzed set. The similarity was evaluated by 13 diagnostic positions and the identity to mm1 and mm2 was taken as +13 and −13, respectively. The diagram shows that copies with varying degrees of similarity to the mm1 or mm2 consensus are represented in approximately equal numbers, although mm2-like copies are slightly more abundant. These data suggest that the division of B2 into mm1 and mm2 subfamilies is not entirely irrelevant. Mm1 and mm2 represent the extreme cases in the variety of B2 sequences, the majority of which correspond to intermediate variants. The variation of diagnostic characters can be due to their independent emergence as well as to the conversion between copies (as observed for Alu copies of different families [62,63]).

Next, we tried to identify B2a variants that were most active in different periods of *M. musculus* evolution. The relative age of B2 copies was evaluated based on their presence in different mouse strains, since older copies should be present in more strains (see Figure 5A). Random copies present in (i) 1 or 2 strains, (ii) 3–10 strains, (iii) 11–15 strains, or (iv) 16 strains were selected. Figure 6C demonstrates that the copies present in all 16 lines (i.e., emerged before the divergence between *M. musculus* and *M. spretus*) have a substantial fraction of mm2-like copies but the greater part belongs to the mm1/mm2 hybrid.

The majority of younger B2 copies found in 1–2, 3–10, and 11–15 mouse strains are closer to mm1, yet there are many hybrid copies. These data suggest that mm2-like B2a copies actively amplified in the mouse genome, but later mm1-like B2a and mm1/mm2 hybrids gradually became much more active than mm2.

We tried to gain more information about very recently active B2 copies by searching sequences in the С57BL/6J mouse genome nearly identical to the above-mentioned six B2 copies that induced gene mutations (Figure S4). The data on such sequences presumably including the parental B2 copies that induced the mutations are given in Table S5. Close relatives of B2 copies that induced the mutations of the *Ndufs4*, *TNF*, and *Slc27a4* genes constitute tribes of 19, 14, and 26 members with an intra-tribe sequence similarity from 99.4 to 100%. Clearly, all these copies are young; their mean poly(A)-tail length is 21.5, 21.1, and 22.7 bp, respectively. The TCTTT terminator predominates in these copies, at 73.7% in the *Ndufs4* tribe and 100% in two other ones. It is hardly possible to identify the parental copies for these tribes; however, they clearly had the TCTTT terminator and a long A-tail. B2 tribes relevant to mutations in three other genes (*Ptpn6*, *Nrcam*, and *Comt1*) included only one or two members with 99.5–100% similarity, which narrows the range of parental copies that induced these gene mutations. The *Comt1* case is particularly interesting: candidate parental B2 includes two identical copies that emerged after an extended duplication in chromosome 5. They have a strong transcription terminator TCT_9_ and no A-tail (Table S5). This instance clearly confirms our concept (see Introduction) since the transcription termination at TCT_9_ followed by AAUAAA-dependent polyadenylation is the only reasonable scenario giving rise to the B2 descendant with an A_52_-tail.

### 2.4. Experimental Analysis of B2 Transcription Termination and Polyadenylation

**Figure 7:**
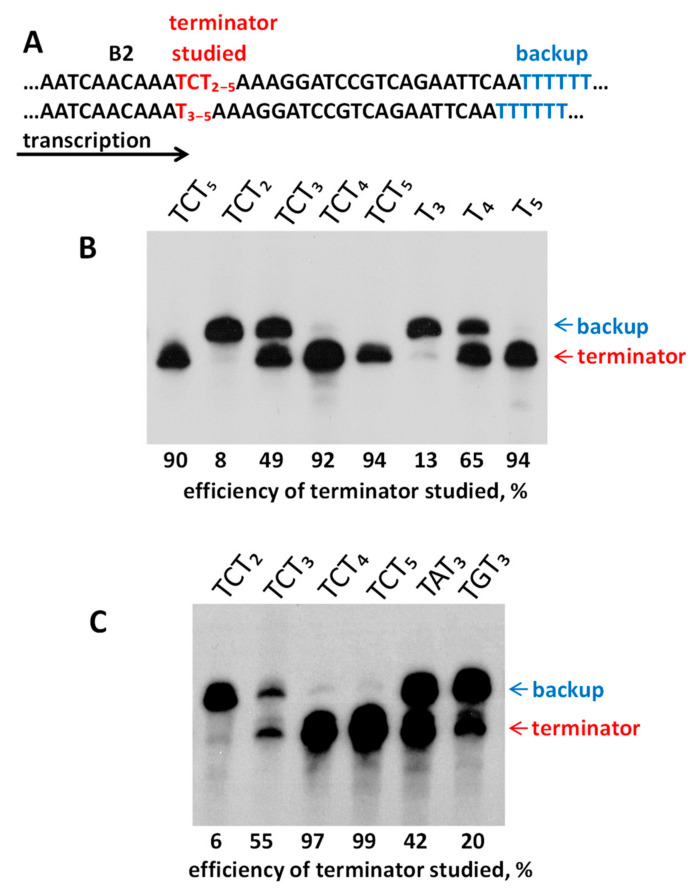
Analysis of transcription terminator efficiency in B2. (**A**) 3′-Terminal sequence of B2 in constructs used for cell transfections. Terminators studied are shown in red and backup terminators are shown in blue. (**B**) Northern blot analysis of RNA from cells transfected by B2 constructs with different terminator sequences. The terminator efficiencies (the ratio of the lower band signal to the total signal) are shown below the blot image. (**C**) A similar experiment including the TATTT and TGTTT terminators.

Orioli et al. (2011) carried out an extensive study of the efficiency of pol III terminators of human tRNA genes in a cell-free system [64]. Here, we tested the efficiency of B2 transcription terminators in human cells transfected with this SINE. The terminator efficiency depends on the flanking sequences [42]; hence, the same terminator can demonstrate different efficiency in a tRNA gene and B2 SINE (in the latter, the terminator is always flanked by several A residues). Accordingly, the capacity of different sequences within B2 had to be evaluated. We used a mouse B2a copy (clone Mm14 [6,30]) to generate the following constructs. (i) In both AATAAA signals, T was replaced with C to suppress polyadenylation in cells so that the transcripts are homogeneous in length. (ii) The original transcription terminator was replaced with T-elements of different lengths, T_3–5_ or TCT_2–5_. (iii) A T_7_ block was added 21 bp downstream of the T-element as a backup terminator (Figure 7A). These constructs were used to transfect HeLa cells and the cellular RNA was analyzed by Northern hybridization. The ratio between the signals corresponding to B2 transcripts terminated at the studied and backup terminators was quantified using a phosphorimager (Figure 7B). As expected, the transcription termination was minor at TCTT and TTT. Conversely, TCT_4_, TCT_5_, and T_5_ effectively terminated transcription. Finally, moderate transcription efficiency was observed for TCTTT (50%) and TTTT (65%). These data indicate that the proportion of terminated transcripts can be deduced from the length and structure of T stretches.

**Figure 8:**
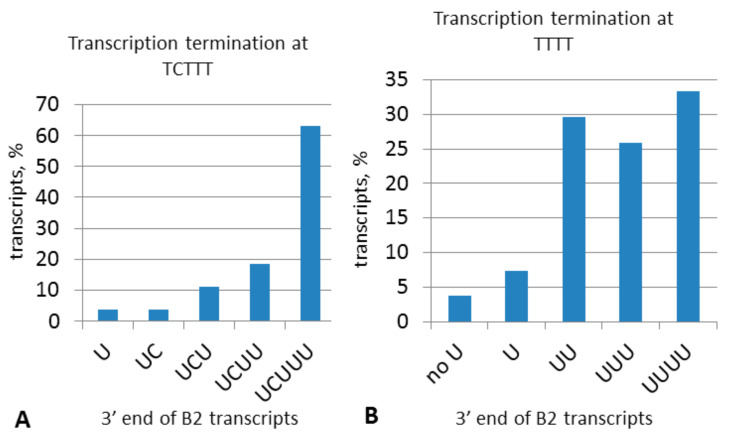
Analysis of 3’ ends of pol III transcripts of B2 terminated at TCTTT and TTTT. HeLa cells were transfected by B2-containing constructs, isolated RNAs were reverse transcribed, and resulting cDNAs were cloned and sequenced. Diagrams demonstrate the distribution of 3’ ends of RNA transcribed from B2 with the TCTTT (**A**) or TTTT (**B**) terminators. Twenty-seven clones were analyzed for each terminator.

Most young B2a copies have TCTTT as the terminator (Figure 4 and Figure 5B, Table S6), which makes it particularly interesting at which nucleotides and with which frequency transcription terminates at TCTTT. In the case of termination at the last nucleotide, the synthesized RNA ends in UCUUU and the same TCTTT terminator is restored after reverse transcription. We transfected HeLa cells with a B2 copy with the TCTTT terminator (similar to that presented in Figure 7 but with both AATAAA signals). CDNA was synthesized using an oligo(dT) primer. In this case, cDNA was synthesized from B2 RNA as well since the transcripts that terminated at TCTTT were polyadenylated in the cell. After PCR and cloning, B2-derived cDNAs were sequenced. The data obtained (Figure 8A) indicate that transcription terminates at the last nucleotide of TCTTT in the majority of cases (63%). Other transcripts terminated at any of the preceding nucleotides up to the first T in the terminator. The resulting distribution is similar to the length distribution of terminators in young B2a copies in the mouse genome (Figure 4 and Figure 5B) where TCTTT copies amount to 55–65%. Clearly, the frequency pattern of pol III termination at different TCTTT positions defines the 3′-end structure of the transcripts, which subsequently defines variations of terminators in the nascent B2 copies. It is critical that a B2 copy with TCTTT can with a high incidence give rise to a new copy with the same moderately efficient terminator.

A similar experiment was conducted using a B2 construct with TTTT instead of TCTTT. The sequences of the cloned cDNA indicate that transcription could terminate at any T of the terminator and even at the preceding nucleotide (Figure 8B), yet the majority of transcripts terminated at the second, third, or fourth T. Interestingly, 33% of transcripts terminated with four U, which is almost half as much compared to transcripts with the 3′-UCUUU in the above experiment. Thus, the TCTTT terminator has notably better chances to be conserved in descendant copies than TTTT.

**Figure 9:**
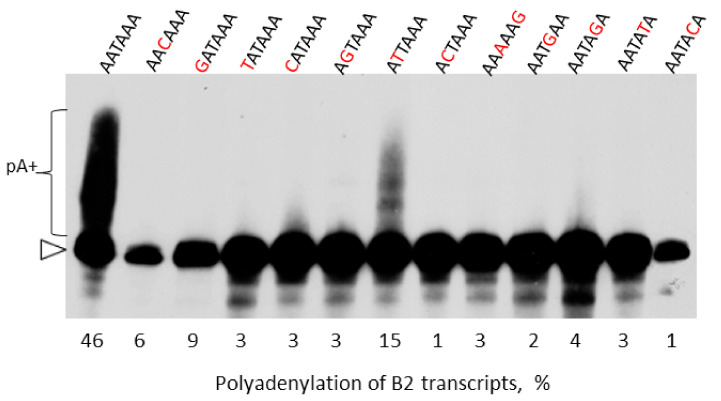
Polyadenylation capacity of canonical PAS (ААТААА) and its variations in B2. The tested hexanucleotide signals are PAS replacements in protein-coding genes. HeLa cells were transfected by B2 constructs with different signals, and isolated RNA was analyzed by Northern hybridization. The major band corresponds to the primary B2 transcript, whereas the smear marked as “pA+” represents polyadenylated B2 transcripts. Polyadenylation efficiency (percentage of pA+ transcripts of B2) is shown below the blot.

Sequence analysis of mRNA indicates that, in addition to the canonical polyadenylation signals AAUAAA and AUUAAA (58 and 15% mRNAs, respectively), other similar hexanucleotides can function as polyadenylation signals [65]. Ten such sequences (usually with a single-nucleotide substitution compared to AAUAAA) have been identified and their involvement in the formation of mRNA 3′-ends has been experimentally confirmed for some of them (see references in [65]). We assumed that such AAUAAA-related signals can also provide polyadenylation of transcripts of B2 and other T**^+^** SINEs. This can be significant for old SINE copies with altered AATAAA sequences. Thus, B2 modifications were constructed where the first AATAAA inactivated by the T to C substitution and the second one included the alterations tested (Figure 9). After the transfection of HeLa cells with these constructs, the isolated RNA was analyzed by Northern hybridization. Only transcripts with AATAAA and АТТААА signals could be relatively efficiently polyadenylated: 46 and 15%, respectively (Figure 9). Apparently, the scarcity of B2 with ATTAAA relative to AATAAA is due to the threefold efficiency difference of these polyadenylation signals.

**Figure 10:**
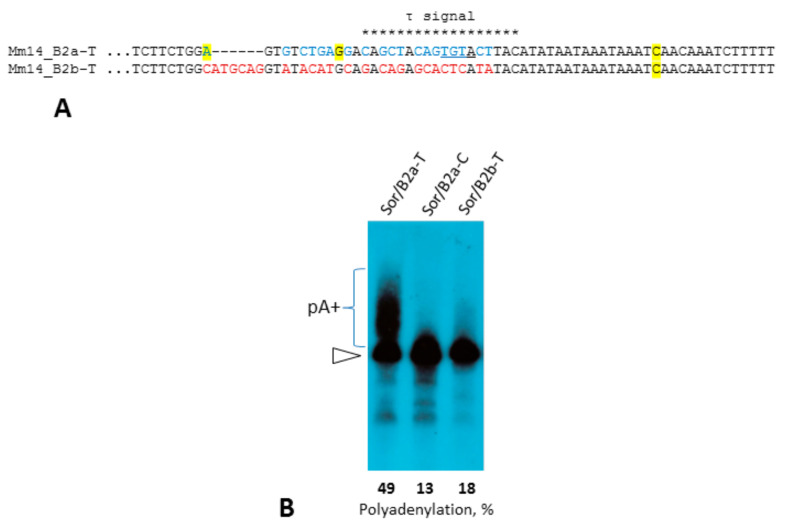
Ability of B2b region corresponding to τ signal in B2a to induce transcript polyadenylation. (**A**) 3′ Region of a B2a copy (Mm14 clone) used in the experiment. Nucleotides specific for this copy are marked in yellow. Nucleotides that convert this region to a B2b-specific sequence are given in red. (**B**) Northern blot hybridization of RNA from HeLa cells transfected by chimeric constructs made of shrew SINE Sor and the 3′ region of B2 (see text for details). PAS was inactivated by a T to C substitution in Sor/B2a-C.

The B2a sequence upstream of the AATAAA region differs from that in other B2 subfamilies (Figure 1). Previously, we demonstrated that this region contains the τ signal responsible for efficient polyadenylation of this SINE. B2b is clearly a T**^+^** SINE (Figure 1), which questions if it has a functional equivalent of the τ signal. Previously, we made a construction of Sor (a shrew T**^–^** SINE) with the 81-bp 3′-terminal sequence of B2a to study the role of this region [31,32]. This construct (Sor/B2a-T) was used to derive another one (Sor/B2b-T) with the corresponding region from B2b (Figure 10A). The transfection of Hela cells with these constructs and the subsequent Northern hybridization demonstrated that the B2b-specific sequence can hardly induce polyadenylation unlike the B2a region with the τ signal (Figure 10B). Thus, B2b and presumably more ancient subfamilies B2c, B2d, and B2e have no functional equivalent of the τ signal. Still, all these subfamilies have the conserved β signal, which likely allows their transcripts to be polyadenylated. The acquirement of the τ signal in B2a SINE that emerged in murids should enhance their capacity for polyadenylation and could alter its regulation, which favored the retrotranspositional success of B2a.

### 2.5. Dip SINE

**Figure 11:**
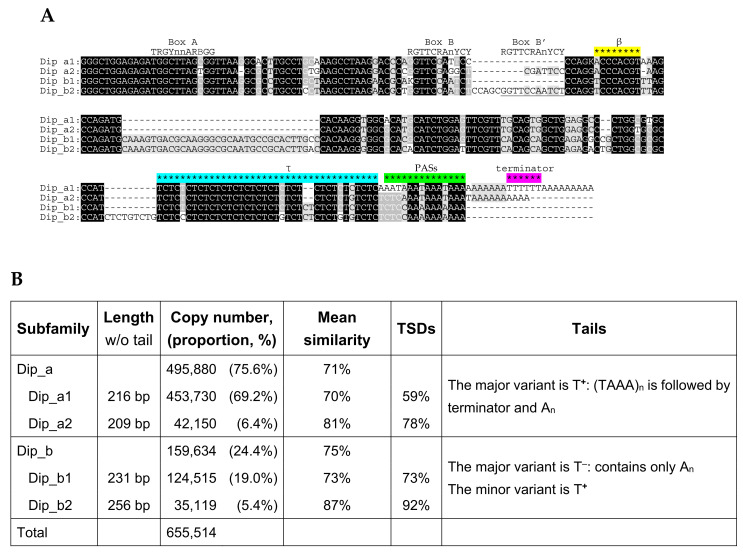
Subfamilies of Dip in the genome of jerboa *Allactaga bullata*. (**A**) Consensus sequences of two Dip subfamilies, each with two variants (a1, a2, b1, and b2). Consensus sequences of A and B boxes of pol III promoter are shown above the alignment. An additional B box in Dip_b2 is underlined and marked as box B′. Positions of β and τ signals, PASs, and pol III transcription terminator are marked by asterisks in different colors. (**B**) Features of Dip subfamilies and their variants. Mean sequence similarity of Dip variants and percentage of Dip copies with TSDs are specified.

The consensus sequence of Dip SINE established previously [49] from a small number of copies in the genomes of jerboas and birch mice was used to find its genomic copies in the genome of the Gobi jerboa *Allactaga bullata.* After establishing the consensus sequences of Dip variants, all of them were used for exhaustive search of Dip sequences. Their total number proved very high: 1,276,950, which is 8.3 times that of B2 in the mouse genome. Almost a half of these copies are within multimers composed of Dip alone (di- and trimers) or with B1; SINE units are tandemly arranged (head-to-tail) and the number of units can be as high as eight (Table S6). In further analysis of Dip, only monomeric structures (655,514 copies) were analyzed. Two subfamilies, Dip_a and Dip_b, were identified differing by a 34-bp insertion in the central part of Dip_b (Figure 11A). Both subfamilies can be further split into two. Dip_a2 has a 7-bp insertion including the box B of the pol III promoter relative to Dip_a1 (Figure 11A). This resulted in two overlapping B boxes **GGTTCGA*GGCT****CGATTCC* (shown in bold and italics, respectively). In the same region, Dip_b2 has a 16-bp duplication also doubling box B (Figure 11A). It is not improbable that two overlapping (Dip_a2) or nearby (Dip_b2) B boxes favor the transcriptional activity of these SINEs.

The number of Dip_a copies in the genome of *А. bullata* is three times that of Dip_b, and both variants ‘1′ significantly outnumber the variants ‘2′ (Figure 11B). According to the mean similarity and proportion of copies with TSDs, the age of Dip variants decreased in the order Dip_a1, Dip_b1, Dip_a2, and Dip_b2 (Figure 11B). Previous experiments with a Dip_a1 copy allowed us to localize the β signal of polyadenylation immediately downstream of box B, although its sequence was not studied in detail [31]. By analogy with the β signal of B2 (Figure 1), the C-rich sequence ACCCACGT spaced by 5 bp from box B can be proposed as the β signal in Dip (Figure 11A). This sequence is conserved except for the first nucleotide replaced with T in Dip_b1 and Dip_b2. As demonstrated previously, a long polypyrimidine motif preceding the A-tail serves as the τ signal in Dip [31]. This motif is found in all Dip variants (Figure 11A). These signal sequences as well as the polyadenylation and transcription termination signals are required for the polyadenylation of Dip RNA. However, PAS is missing in Dip_b consensus sequences and the terminator is found only in Dip_a1 (Figure 11A). Thus, only the latter Dip variant can be considered as a T**^+^** SINE according to their consensus sequences. At the same time, our data indicate numerous T**^+^** copies among other Dip variants. Individual copies of this SINE were further analyzed to evaluate the proportion between its T**^+^** and T**^–^** copies.

The potential Dip terminators often included TATTT or, less commonly, TCTTT and TGTTT. This is distinct from B2, where TCTTT was the major moderate terminator. Orioli et al. [64] reported that TATTT and TGTTT terminated tRNA transcription in vitro in 50 and 40% cases, respectively. We evaluated the efficiency of these terminators in B2 transfection constructs; TATTT and TGTTT terminated SINE transcription in 42 and 20% cases, respectively (Figure 7C). Accordingly, these sequences were considered terminators in the subsequent analysis.

Dip copies with terminators were identified in Dip_a and Dip_b sets (487,241 and 154,759 sequences, respectively) by the presence of the TNT_m_A_n_ motif, where *m* ≥ 1 and *n* ≥ 5 (Table S7). These copies were divided into three groups by the A-tail length: А_5–10_, А_11–20_, and A_>20_; the first group was 20 and 9 times more abundant than two other groups together for Dip_a and Dip_b, respectively. Copies with proper terminators were rare among Dips with А_11–20_ and A_>20_ tails and appeared below the threshold of 5%. At the same time, Dip copies with А_5–10_ tails and T_≥4_ or TVT_≥3_ terminators (V = C, A, or G) amounted to 45 and 23% in Dip_a and Dip_b subfamilies, respectively (Table S7 and Figure S7). Only rudimentary terminators (T_2–3_ or TVT_1–2_) could be found in the remaining copies. The data obtained indicate that not only Dip_a but also a substantial fraction of Dip_b copies can be considered as T**^+^** SINEs as well as that the incidence of proper terminators greatly increases as the A-tail shortens below ten nucleotides.

**Figure 12:**
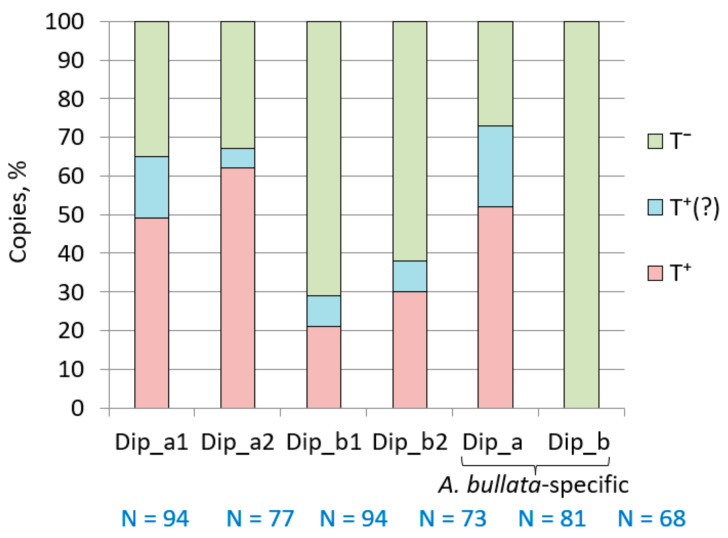
Percentage of Dip copies (variants a1, a2, b1, and b2) with T**^+^** and T**^−^** tails. Conventional T**^+^** SINEs (with very short terminator rudiments or A_<5_) are referred to as ‘T**^+^**(?).’ (See Figures S8–S11 for the samples of random Dip_a1, _a2, _b1, and _b2 copies). Two rightmost bars show the proportion of Dip_a and Dip_b copies present in the *A. bullata* genome but absent in the homologous *J. juculus* loci. (See Figures S12 and S13 for the samples of such Dip_a and Dip_b copies). The numbers of analyzed copies are shown below as ‘N = number’.

We also performed a manual analysis of relatively small samples of this SINE as an alternative approach. Random samples of all Dip subfamilies from *А. bullata* were aligned and the PASs, transcription terminators or their rudiments, and oligo(A)-tails were identified (Figures S8–S11). Dip copies were considered as T**^+^** SINEs if at least one PAS, proper or rudimentary terminator (T_≥2_ or TVT_≥2_), and A_>3_ tail were present. The copies with PAS and a more rudimentary terminator or oligo(A) tail were considered as conventional T**^+^** SINEs. Analysis of these sets demonstrated a significant portion of T**^+^** SINEs in all Dip variants: 48% in Dip_a1, 62% in Dip_a2, 21% in Dip_b1, and 30% in Dip_b2 (Figure 12). Conventional T**^+^** SINEs were minor in all cases, while T**^–^** copies were abundant and predominated in Dip_b1 (71%) and Dip_b2 (62%) (Figure 12). Thus, the analysis of individual copies demonstrated a substantial fraction of T**^+^** SINEs in the Dip_b subfamily, although no transcription terminator can be found in the consensus sequences (Figure 11A) due to the excess of T**^–^** copies. The Dip_a2 consensus sequence also lacks the terminator, although T**^+^**SINEs predominate in this variant (62%). This can be attributed to the high incidence of rudimentary terminators as well as size variation of the PAS region (the number of AATAAA units varied from 1 to 5; Figure S9), which complicates the alignment of the 3’-terminal sequences and terminator identification in the consensus sequence.

Since the genome of another jerboa *Jaculus jaculus* has been sequenced, we tried to identify relatively young Dip copies of *A. bullata*. *A. bullata* and *J. jaculus* belong to sister subfamilies Allactaginae and Dipodinae diverged ~20.5 million years ago (Mya), while the Dipodidae family emerged ~43 Mya [66,67]. Our rough estimate of *A. bullata* Dip SINEs that are missing in the homologous loci of *J. jaculus* amounts to 20,000 copies. Thus, as low as ~2% of all *A. bullata* copies appeared after the divergence of Allactaginae and Dipodinae, which indicates a decline in the activity of this SINE. Random samples of such *A. bullata* Dip_a and Dip_b sequences were analyzed (Figures S12 and S13). The distribution between T**^+^**, conventional T**^+^**, and T**^–^** Dip_a copies was similar to that in random Dip_a1 and Dip_a2 sequences (Figure 12). Unexpectedly, all sampled Dip_b copies of *A. bullata* missing in the corresponding *J. jaculus* loci proved to be T**^–^** copies (Figure 12). Thus, most Dip_a copies that emerged in *A. bullata* in the recent 20 My are T**^+^** SINEs, while all (or most) of such Dip_b copies are T**^–^** SINEs.

The number of Dip copies with long A-tails (A _≥ 20_) in *A. bullata*-specific copies was as low as 129, which amounts to ~0.65% of all *A. bullata*-specific copies (Figure S14). The majority of these copies with long A-tails (85%) belonged to the Dip_b subfamily and had 3′-terminal regions typical of T**^–^** copies. If long A-tails mark relatively recent SINE copies, these data indicate that Dip_b remained retrotranspositionally active over a longer period than Dip_a.

In order to find out if Dip is still active or its amplification declined, highly similar groups of Dip copies were extracted from the genome of *A. bullata,* and the divergence of their nucleotide sequences was evaluated. Thirty-three groups (tribes) were identified, each of which included Dip_a copies with highly similar (>95%) nucleotide sequences (excluding the TC-motif and A-tail since these simple sequences are prone to post-integration modifications). These young tribes were designated as Dip_a_Y1, Dip_a_Y2, etc. in descending order of copy number (the corresponding 33 consensus sequences are given in Figure S15). Only the Y1 and Y2 tribes are relatively large (1789 and 121 sequences) while the remaining tribes include 12 sequences on average. The similarity within Y1 is 98% suggesting that they emerged about 2.4 Mya (Figure S16); ~10% of Y1 copies emerged 0.7 Mya or later. The Y2 tribe is slightly older, the mean age of its copies is 3.6 Mya (Figure S16). Interestingly, Dip copies of Y1 and Y2 as well as three lesser tribes (Y3, Y7 и Y13) have the ACCTGCCTATTTCTGTA motif within the TC-region (Figure S15). As a matter of speculation, this motif can overcome the cellular mechanisms against retrotransposition. Thus, the Dip_a subfamily largely declined; however, minor copies were active relatively recently and still can remain active.

No tribes as abundant as Dip_a_Y1 were found among young Dip_b. The most abundant Dip_b tribes (Y1-Y4) included 108, 27, 15, and 11 copies; similarity analysis of Dip_b_Y1 copies indicates the maximum retrotranspositional activity 3.0 Mya (Figures S17 and S18). Thus, the Dip_b activity has also largely declined. Although the Dip_b subfamily (particularly, Dip_b2) is younger than Dip_a (Figure 10B), certain Dip_a copies (Y1 tribe) demonstrated the highest amplification activity. It is worth mentioning that the recently active tribes of both Dip_a and Dip_b are T**^+^** SINEs, while older Dip_b copies amplified in the *A. bullata* lineage soon after split from the *J. jaculus* lineage are T**^–^** SINEs (see below and Figure 12). Apparently, Dip had many amplification waves (fading waves in the recent 20 My) and successful Dip_a and Dip_b variants replaced each other.

**Figure 13:**
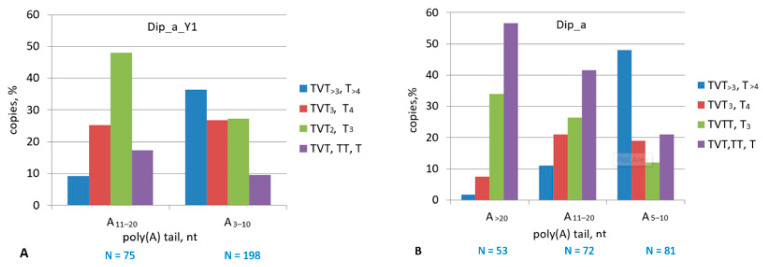
Distribution of different terminators among Dip_a copies with poly(A) tails of different lengths. (**A**) Dip_aY1 copies. (**B**) Random Dip_a copies. The numbers of analyzed copies are shown below as ‘N= number.’.

The analysis of A-rich tails and terminators among recent SINE copies is particularly interesting since their less-degraded sequences allow more adequate conclusions about the parental SINEs. Thus, the 3′-terminal sequences of 189 random Dip_a_Y1 copies were analyzed (Figure S19). The tails of 16 copies had neither terminators nor A-tail (ended with (AAAT)_≤13_) and were excluded from further analysis. The remaining copies were divided into two groups with A_3–10_ and A_≥11_ tails. Moderate pol III terminators (mainly TATTT) were equally presented (25%) in both Dip_a_Y1 groups, while the incidence of efficient terminators in the group with short oligo(A) tails was four times that in the group of long oligo(A) tails (Figure 13A). Thus, similar to SINE B2a, the terminators tend to elongate as the oligo(A) tails shorten. As a result of this process, 80% of all Dip_a_Y1 copies have terminators and thus are capable of retrotransposition.

Finally, a similar analysis was performed with all (rather than young) Dip_a copies, which largely include old copies integrated more than 20 Mya. Although Dip_a amplification declined long ago, *A. bullata* genome contains its copies with long A-tails (A_≥20_); such copies amounted to 0.6% of the subfamily (Table S8). One can propose that a minor fraction of Dip_a escaped radical reduction of poly(A) tails observed in aging SINEs. In rare cases, long poly(A) tails of SINEs can contribute to the functioning of nearby genes, which explains their conservation; other specific features of the loci (e.g., chromatin structure) can also prevent SINE poly(A) shortening. The transcription terminators or their rudiments were analyzed in Dip_a samples with A-tails of different lengths (Figures S20–S22). At most, 10% of copies with А_≥20_ tails have Т_≥4_ or TVТ_≥3_ terminators, the remaining copies contained their rudiments (Figure 13B). Terminators are found in 32 and 67% copies with A_11–20_ and A_5–10_ tails, respectively (Figure 13B), i.e., the terminators tend to elongate as the tails shorten. (This generally corroborates the above analysis of all genomic copies of Dip_a demonstrating the terminators only in the copies with A_5–10_ tails.) We believe that Dip_a with long (А_≥20_) tails are not young SINEs, which suggests that the recovery (elongation) of terminators is directly linked to the poly(A) tail shortening; and only indirectly, to their age. Stated differently, the copies without significant reduction of the poly(A) tail demonstrated no terminator elongation.

### 2.6. Ves SINE

**Figure 14:**
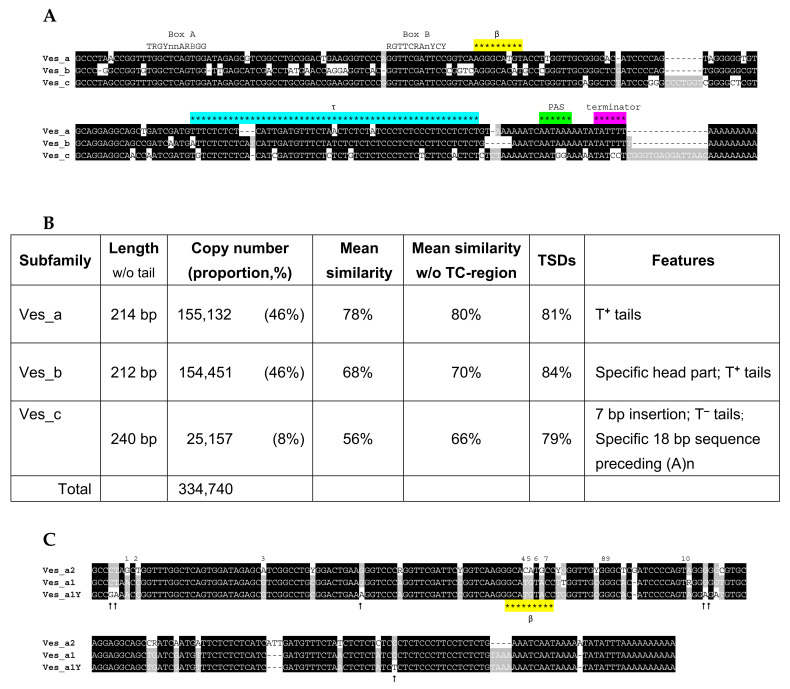
Subfamilies of Ves in genome of bat *Myotis lucifugus*. (**A**) Consensus sequences of three Ves subfamilies. Consensus sequences of A and B boxes of pol III promoter are shown above the alignment. Positions of β and τ signals, PASs, and pol III transcription terminator are marked by asterisks in different colors. (**B**) Features of Ves subfamilies. The mean sequence similarity of Ves subfamilies and the percentage of Ves copies with TSDs are specified. (**C**) Consensus sequences of two Ves_a variants (a1 and a2) as well as of young Ves_a1Y copies. Nucleotide positions that distinguish Ves_a1 and Ves_a2 are numbered. Nucleotide substitutions specific for Ves_a1Y are marked with arrows.

The consensus sequence of Ves SINE established previously [50] from a small number of copies in the genome of the water bat *Myotis daubentonii* was used to find its genomic copies in the genome of the little brown bat *Myotis lucifugus*, the first chiropteran with a sequenced genome. After finding consensus sequences of Ves variants, all of them were used for exhaustive search of Ves sequences. A total of 378,564 Ves copies were found in the *M. lucifugus* genome, 11.5% (43,824 copies) of which were parts of dimeric SINEs, which is much lower compared to multimeric B2 (40%) or Dip (49%). Analysis of all monomeric Ves copies allowed us to divide them into three subfamilies, Ves_a, Ves_b, and Ves_c (Figure 14A,B). Ves_c is the oldest and least abundant (8% copies) subfamily distinguished by the 7-bp insertion in the central part and the absence of the PAS and terminators in the 3′-terminal region. Most Ves_c copies have the sequence TATCCTCGGGTGAGGATT with the reverse complement motif (underlined) preceding the A-tail (Figure 14A and Figure S23). Two other subfamilies amount to 46% copies each; Ves_b is clearly older than Ves_a. The head sequence of Ves_b demonstrates notable differences from Ves_a and Ves_c, which are similar in this region. In contrast to Ves_c, the other two subfamilies have the PAS and terminator, i.e., they are T^+^ SINEs (Figure 14A, Figures S24 and S25). The 3′-regions of Ves_a and Ves_b are shorter and more conserved compared to those of B2 and Dip, in particular, due to the only AATAAA sequence, unlike repetitive TAAA in B2 and Dip. The majority of pol III transcription terminators in Ves_a and Ves_b are TAT_≥3_. By analogy with TCT_3_ in B2, the TAT_3_ should have better chances to persist in retrotransposition than T_4_.

The sequences of the terminators and their rudiments were also analyzed in the complete sets of Ves_a, Ves_b, and Ves_c copies from *M. lucifugus* (Table S9). They were divided into three groups by the A-tail length: А_5–10_, А_11–20_, and A_>20_; the first group was 16–20 times as large as the other two groups for Ves_a and Ves_c; and 60 times, for Ves_b. The proper terminators were rare among Ves copies with А_11–20_ and A_>20_ tails (below the 5% threshold). At the same time, T_≥4_ and TVT_≥3_ terminators (where V is A >> C/G) in SINE copies with А_5–10_ tails occurred in 48%, 36%, and 24% copies of Ves_a, Ves_b, and Ves_c, respectively. Other copies had rudimentary terminators T_2–3_ or TVT_1–2_. According to these data, the shortening of poly(A) tails was commonly accompanied by the terminator elongation (particularly for Ves_a).

Similar to Dip, all Ves copies have a long TC-motif promoting polyadenylation of Ves transcripts [31], i.e., functioning as the τ signal. The β signals proved to vary in Ves_a and Ves_b consensus sequences: AGGGCATGT and AGGGCACAT, respectively (Figure 14A). Since the β signal was experimentally studied only in a Ves_a copy [31,32], the capacity of the second sequence remained unclear. Thus, we tried a Ves_a copy without the β signal or with the TG to CA substitution in the β-signal. This substitution had little effect on the transcript polyadenylation efficiency (Figure S26); thus, this sequence in Ves_b can serve as the β signal.

Further analysis of Ves_a revealed two variants (a1 and a2) distinguished by 23 nucleotide positions in their consensus sequences (Figure 14C). The separation of Ves_a1 and Ves_a2 using these diagnostic characters is much better than that of the mm1 and mm2 variants of B2a (Figure S27). The number of Ves_a1 copies is 1.5 times that of Ves_a2. The a1 variant is younger than a2 as indicated by the higher main similarity, 88% и 81%, respectively. The β signal in Ves_a1 corresponds to the Ves_a sequence, while that in Ves_a2 corresponds to the β signal of Ves_b (AGGGCACAT).

We tried to identify relatively young Ves copies by finding the loci with Ves in *M. lucifugus* that are missing in *M. myotis* [68]. About 25,000 such copies have been identified. Among 172 random copies, 169 and 3 corresponded to Ves_a1 and Ves_a2, respectively (Figure S28). The *Myotis* genus split about 10 Mya [69]. Accordingly, almost exclusively Ves_a1 copies amplified in the *M. lucifugus* lineage in the recent 10 My. Apparently, Ves_b and Ves_c became inactive long before that.

Even younger Ves copies were identified in the *M. lucifugus* genome as clusters of highly similar sequences. In the case of Ves_a, 81 such tribes with at least 10 sequences (231 at most) have been found (with a total number of 2359). All tribes corresponded to Ves_a1 consistent with the above data on the small age and activity of this variant. The consensus sequences of 75 tribes are distinguished by 1 to 3 nucleotides, which allows us to assign them to the same supertribe (Figure S29). The consensus sequence of all tribes has six substitutions relative to the Ves_a1 that likely distinguish recently active copies, this variant is referred to as Ves_a1Y (Figure 14C). Sequence identity was evaluated for the four largest tribes: 18–48% copies were identical, and the identity distribution peaked at 98.7–99.3%, which indicates their active amplification from ~1 Mya to the present (Figure S30). In the case of Ves_b, only three small tribes have been found (38, 11, and 5 copies) with the mean similarity from 93 to 99% (Figure S31); this indicates the most recent retrotranspositional activity several Mya.

**Figure 15:**
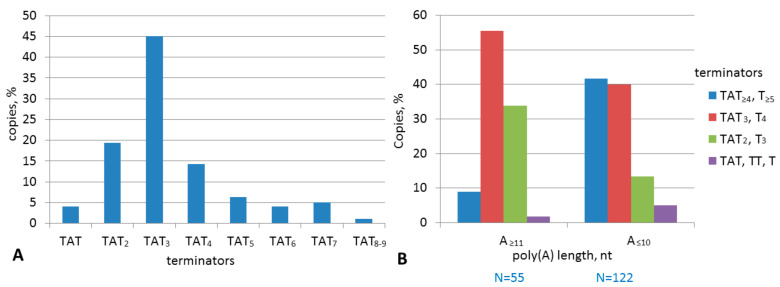
Analysis of pol III terminators in a young Ves tribe (Ves_a1Y_187). (**A**) Distribution of TAT_n_ terminators among copies of the tribe. (**B**) Distribution of terminators among Ves_a1Y_187 copies with long (A_≥11_) and short (A_≤10_) poly(A) tails. The numbers of analyzed copies are shown below as ‘N = number’.

Young tribes are of particular interest in terms of the analysis of the terminators and poly(A) tails since mutations only slightly changed their sequences. We analyzed the 3′-terminal sequences of a Ves_a1Y tribe (187 copies) (Figure S32). TATTT was the most common terminator (45%), longer efficient terminators TA(T)_4–9_ were found in 32% of copies, and rudimentary terminators were found in 23% of copies (Figure 15A). Further analysis of the terminators in the copies with different poly(A) tail lengths demonstrated that the incidence of efficient terminators is 4.7 higher in the copies with А_≤10_ compared to those with А_≥11_ (Figure 15B). Other Ves_a1Y tribes demonstrate very similar distribution patterns of the terminators and poly(A) tails. These data confirm our model of time changes in the 3′-terminal sequences of T**^+^** SINEs.

## 3. Discussion

Previously, we assigned B2, Dip, and Ves to T**^+^**SINEs considering their PAS (AATAAA) and pol III transcription terminators (T_>3_) in their 3′-regions [29]. This conclusion relied on the analysis of a very small number of copies. Here, we analyzed either all full-length monomeric copies of these SINEs or their relatively small samples. A detailed analysis of large data volumes allowed us to draw interesting conclusions concerning SINEs and their 3′-terminal regions, in particular. These three families were selected since the β and τ signals required for the polyadenylation of their RNA have been revealed previously in these SINEs [31,32].

SINEs were divided into subfamilies using indels at least several nucleotides long as diagnostic characters. In many cases, single-nucleotide substitutions are also used to distinguish SINE subfamilies; this can be fruitful (e.g., for Alu) but more often recombination of such single-nucleotide characters provide for unreliable conclusions. We illustrated this for the mm1 and mm2 sequences described as B2 subfamilies in (Figure 6 and Figure S6). Ichiyanagi et al. [70] have recently reported a large B2 cluster mm1o considered as an evolutionary intermediate between mm1 and mm2. Indeed, there is a trend of B2 evolution from mm2 to mm1o to mm1; however, most sequences in this continuous series cannot be assigned to the subfamilies due to irregular combinations of the diagnostic characters. Relatively long indels used in this work allow a more reliable but less detailed SINE division into subfamilies.

Apart from the analysis of B2, Dip, and Ves subfamilies, the relative age of these SINEs and different aspects of their sequences were studied with a focus on the structure of their 3′-terminal regions with PAS, pol III terminators, and oligo(A) tails.

### 3.1. B2 Family

According to the divergence of the copies and wide distribution among rodents (Muridae, Cricetidae, and Spalacidae), the most ancient B2 subfamilies are abundant B2d (B3 in RU) and small B2e. The B2b and B2c subfamilies are younger; they are found in murids and cricetids but not in spalacids. Finally, the B2a family is the youngest and emerged in murids; it amounts to two-thirds of all B2 in the rat and mouse genomes. The consensus sequences of all subfamilies except B2e have PASs, while B2a and B2b also have pol III terminators (Figure 1); thus, at least the latter two families are T**^+^** SINEs. B2c and B2d copies also include sequences with the TCTTT terminator, although they are minor (Table 1). Interestingly, the terminator can be found in most B2d copies of the mole rat (Spalacidae). We consider the B2c and B2d subfamilies as T**^+^** SINEs as well and attribute the low incidence of pol III terminators in their copies to the shortening of terminators in generations of gradually decaying B2 in mice and rats. All B2 subfamilies have the regions corresponding to the β signal identified previously in B2a, and single-nucleotide substitutions in the first position should not be critical [32]. The τ signal region has a different sequence in other B2 subfamilies. This potential signal was experimentally tested but failed to promote transcript polyadenylation (Figure 10). Apparently, the polyadenylation of B2b, B2c, and B2d transcripts relies on the β-signal and PAS alone.

We also experimentally tested other hexanucleotides than AAUAAA for the capacity to promote polyadenylation of B2 transcripts. Eleven sequences with a single-nucleotide substitution relative to AAUAAA are found in 40% of mRNAs and function as a PAS [65]; AUUAAA is the most common among them (15% mRNAs). Our data indicate that only AUUAAA out of these sequences can function as a PAS in pol III-generated B2 transcripts, yet, this signal is three times less efficient than AAUAAA. This explains the presence of the AATAAA signal in B2 and other T**^+^** SINEs and suggests that mutations in this signal prevent the polyadenylation and retrotransposition of such mutant copies. However, it should be noted that the presence of several AATAAA signals in B2 and most other T**^+^** SINEs likely makes their polyadenylation and retrotransposition tolerant to individual PAS mutations.

In the context of this study, the efficiency of different pol III terminators in B2 had to be evaluated. Experiments on cell transfection with B2 constructs demonstrated 50, 42, and 20% transcription termination for the ТCTTT, TATTT, and TGTTT signals, respectively. Overall, these data are consistent with the termination efficiencies for these sequences in a model tRNA gene, although tRNA transcription termination at TTTT was more efficient (~95%) [64]. The nucleotides adjacent to the pol III terminator are known to influence its function [42], this can explain the different efficiency of TTTT for B2 and the tRNA gene. In our experiments, an addition of at least one T to TCTTT or TTTT increased the termination efficiency to nearly 100%, while their shortening by a single T disabled these signals. Thus, TTTT and TVTTT (where V = C, A, or G) are the shortest terminators with moderate efficiency.

In order to understand how the TCTTT and TTTT terminators change in retrotransposition of B2 (or other T**^+^** SINEs), the frequency of termination at each nucleotide of these terminators was experimentally evaluated. As anticipated, many transcripts terminated at nucleotides preceding the last T, which means that the terminators shorten and become nonfunctional after reverse transcription. However, 63% of transcripts terminated at the last nucleotide of TCTTT; and 33%, at the last TTTT. This better persistence gives TCTTT an advantage over TTTT in B2 evolution.

We focused on the largest and youngest subfamily B2a. The age of B2a copies proved to be inversely related to the length of their poly(A) tails. The poly(A) tail length of B2 copies that recently integrated into genes and affected their function was 58 bp on average. Such long poly(A) tails were reported for recently integrated Alu that caused human hereditary disorders [11,12]. Apparently, long poly(A) tails in the recent Alu copies are inherited from very rare master copies [12,40]; i.e., their origin is distinct from that in B2. The length of the poly(A) tail in older B2 copies integrated into the genome of C57Bl mice or their common ancestor with NZO was 25 bp on average. The poly(A) tail was further shortened with the age of B2 copies that emerged before the divergence between *M. musculus* and *M. spretus*, its mean length was as low as 10 bp. However, older copies demonstrate A-tail reduction down to zero length. The shortening of poly(A) tails with the age of Alu copies was reported previously [38]; the underlying mechanism is not known but can be due to the DNA polymerase slippage on simple sequences.

Sequence analysis of B2a terminators demonstrated that the majority (55%) of young copies (with poly(A)_>20_) have moderately efficient terminators TCTTT or more rarely TTTT; rudimentary terminators (TCTT, TCT, and TC) were found in 40% of such copies. This distribution is consistent with our experimental data on transcription termination at different TCTTT nucleotides in B2. The data obtained indicate that the B2 copies with the TCTTT terminator have good chances to generate new copies with the same terminator. This is even more vivid for the recent B2 copies (Figure S4 and Table S5). Thus, B2 copies with the TCTTT terminator can be continuously reproduced. Our statistics of transcription termination at TTTT nucleotides suggests its lower reproducibility in the descendant copies, which favors the extinction of such T**^+^** SINEs.

These data have led us to an unexpected conclusion that the aging of B2 copies and shortening of their poly(A) tails significantly increases the proportion of copies with longer and more efficient pol III terminators. The tail shortening below A_~10_ is the critical point; the proportion of TCT_>3_ and T_>4_ terminators is 38% among B2 copies with A_5–10_ tails or as low as 2% in those with longer A-tails (Figure 4). The TCTTT rather than rudimentary terminators seems to elongate since the increased proportion of efficient terminators correlated with the decreased proportion of TCTTT. The mechanism of T amplification in pol III terminators is unclear; we can think of DNA replication slippage at TTT. Such T amplification is opposite to the shortening of the terminators in the course of pol III-dependent transcription and subsequent retrotransposition. We believe that this T amplification is critical for the proliferation advancement of B2 and other T**^+^** SINEs since it restores the efficiency of pol III terminators.

### 3.2. Dip Family

Dip SINE emerged and amplified in the ancestors of dipodids (jerboas, jumping mice, and birch mice) after their separation from muroid rodents [49] (Figure 2). Here we analyzed Dip copies from the sequenced genome of the Gobi jerboa *Allactaga bullata*. Their sequences were divided into two subfamilies, Dip_a and Dip_b, based on the 34-bp insertion in the latter. Each family can be further divided into two variants. Dip_b2 is distinguished from Dip_b1 by a 16-bp sequence that likely emerged by duplication, 11 bp of which is an extra B box of the pol III promoter. Apparently, Dip_a2 emerged from Dip_a1 by a similar duplication followed by a deletion of several nucleotides; as a result, Dip_a2 has two B boxes overlapping by four nucleotides. Similarly, an internal duplication in rodent B1 gave rise to another B box [14] that remains active in at least certain copies [71]. It is not improbable that an extra box B allows SINEs to bypass cellular mechanisms repressing their retrotransposition.

All four Dip variants have the putative β (AC-rich motif) as well as the τ (TC-rich motif) polyadenylation signals. According to the consensus sequences, both Dip_a variants have PASs, while the terminator is found only in Dip_a1; the consensus sequences of Dip_b1 and Dip_b2 lack both signals (Figure 10). At the same time, analysis of random individual Dip copies indicates that both T**^+^** and T**^–^** copies are present; however, T**^+^** predominates in Dip_a (~60%); and T**^–^**, in Dip_b (60–70%; Figure 11). Thus, in contrast to B2a, Dip_a and Dip_b represent mixed T**^+^**/ T**^–^** SINEs. On the one hand, the T**^+^** characters could be lost in old copies (98% Dips emerged 43 to 20 Mya); on the other hand, certain copies amplified as T**^–^** SINEs. Their 3′-terminal regions include relatively long poly(A) without PAS and terminators or their rudiments; such sequences are typical for Dip_b, particularly, those that emerged in the recent 20 My, i.e., after the divergence between *A. bullata* and *J. jaculus*. The amplification of such Dip copies likely relies on the mechanism proposed for the human Alu when only a small number of copies with very long poly(A) tails are retrotranspositionally competent.

The search for highly similar (young) groups of Dip copies has revealed just a few of them in *A. bullata*. Among 33 tribes of Dip_a, only two, Y1 and Y2, were relatively large and included 1789 and 121 copies with the mean age of 2.4 and 3.6 My, respectively. The largest Dip_b tribe included 108 copies with the mean age of 3.0 My. Amazingly, all found young copies of both Dip_a and Dip_b were T**^+^** SINEs. Apparently, T**^–^** Dip_b amplified in the period from ~20 to ~5 Mya but later T**^+^** Dip_b started to amplify. However, both Dip_a and Dip_b rarely amplify in the recent several My and Dip can be considered as almost extinct in *A. bullata*.

The analysis of 3′-terminal sequences in T**^+^** Dips demonstrated some differences from those in B2a. The number of AAAT repeats (and hence PASs) varied much more in Dip, from 1 to 12 (commonly 4–5). This can be partially attributed to the older age of Dip copies giving more opportunities for tandem amplification of AAAT. The majority of B2 terminators are TCТ_≥3_ and less commonly Т_≥4_; while the most common Dip terminators are TAТ_≥3_ and Т_≥4_, TСТ_≥3_ and TGТ_≥3_ occur much rarer. We believe that similar to the TCTTT terminator, TATTT has good chances to be preserved after retrotransposition, which explains its high incidence in the 3′-terminal sequences of T**^+^** Dips.

Despite the age of Dip_a copies, about 0.6% of them have long tails (A_>20_). Presumably, not all old Dip copies undergo shortening of their poly(A) tails. As low as 10% of Dip_a copies with long poly(A) tails have functional terminators (TVТ_≥3_ and Т_≥4_). As with B2a, the shortening of Dip_a A-tails is accompanied by the elongation of their rudimentary terminators, which restores their function. Thus, 67% of Dip_a copies with A_5–10_ tails have TVТ_≥3_ and Т_≥4_ terminators. Such terminator elongation can be considered as a significant factor restoring the capacity of Dip copies for retrotransposition.

### 3.3. Ves Family

Previously, Ray et al. [52] divided Ves copies in *M. lucifugus* into 31 subfamilies. In our opinion, the assignment of copies to such small subfamilies can be problematic due to the recombination of their diagnostic nucleotides; in addition, it substantially complicates the detailed analysis of their copies. Accordingly, we divided Ves SINE into three easily distinguished subfamilies (a, b, and c). Moreover, we performed a detailed analysis of the Ves structure, particularly, its 3′-terminal region.

The Ves_c subfamily is the oldest and had lost retrotranspositional activity long ago. Arguably, it gave rise to two other subfamilies, older Ves_b with specific head structure and younger Ves_a. Ves_c has no PAS and thus should be considered as a T**^–^** SINE. However, there is an 18-nt potential loop upstream of the oligo(A) tail. As a matter of speculation, Ves_c could amplify as a T**^+^** SINE if this loop can functionally substitute PAS.

The 3′-terminal structure of Ves_a and Ves_b clearly indicates that they are T**^+^** SINEs. In contrast to B2a and Dip, these regions in Ves_a and Ves_b are shorter and more conserved: they always have a single PAS. Unknown factors (e.g., specific Ves structure or properties of bat enzymes) prevent tandem amplification of PAS observed in B2, Dip (and other T^+^ SINEs, data not shown). In addition, TAT_≥3_ is the most common terminator in Ves unlike TCT_≥3_ in B2.

The Ves_a subfamily can be further divided into two variants (a1 and a2) distinguished by 23 nucleotide positions. The analysis of Ves copies that appeared in the *M. lucifugus* genome before its divergence from *M. myotis* (at least 10 Mya) demonstrated that most of them (98%) correspond to Ves_a1. Thus, the activity of Ves_a2 declined long ago. The search for highly similar groups of Ves_a copies has revealed young tribes that emerged less than 1 Mya; their consensus sequence (Ves_a1Y) featured six extra single-nucleotide substitutions relative to Ves_a1. In one of such large Ves tribes, the bulk of copies had the TAT_3_ terminator, and the incidence of strong terminators ТAТ_≥4_ increased 4.7-fold in the copies with the poly(A) tails shorter than 11 nt relative to those with longer tails. These data indicate that the TAT_3_ terminator can be well maintained after retrotransposition and that the terminators can elongate in Ves copies as they get older.

The long TC-motif functioning as a τ signal of polyadenylation is found in all three Ves subfamilies. According to the consensus sequences, the TC-motifs are very similar in Ves_a and Ves_b. Young Ves_a1 copies even from different tribes have highly similar TC-motifs; however, it can substantially vary in old copies due to nucleotide substitutions and indels. Analysis of the β-signal of polyadenylation has revealed the AGGGCATGT as such signal in Ves_a1. Our previous experiments using a Ves_a1 copy showed a significant contribution of this sequence to the efficiency of polyadenylation [31,32]. In this work, the analysis of other Ves sequences has revealed a TG to CA substitution in sequence in Ves_a2 and Ves_b. Although many dinucleotide substitutions substantially decreased the β signal efficiency [32], here we experimentally demonstrated that the TG to CA substitution has little effect on the efficiency of Ves transcript polyadenylation. Thus, currently retrotranspositionally incompetent Ves_a2 and Ves_b have a functional β-signal.

## 4. Materials and Methods

### 4.1. Bioinformatics Methods

The genomic data were downloaded from NCBI Genomes (https://www.ncbi.nlm.nih.gov/genome) (accessed on 1 May 2020) except rat blind mole-rat (Ensembl, https://www.ensembl.org) (accessed on 1 May 2020). The following assemblies were used: *Mus musculus*, GRCm38/mm10; *Rattus norvegicus*, Rnor_6.0; *Cricetulus griseus*, CriGri-PICR; *Nannospalax galili*, S.galili_v1.0; *Peromyscus maniculatus*, HU_Pman_2.1; *Jaculus jaculus*, JacJac1.0; *Allactaga bullata*, AllBul_v1_BIUU; *Myotis lucifugus*, Myoluc2.0.; and *Myotis myotis*, MyoMyo_v1_BIUU. The genomes from the Mouse Genomes Project (https://www.sanger.ac.uk/data/mouse-genomes-project/) (accessed on 1 May 2020) were compared using the UCSC Genome Browser (http://genome.ucsc.edu) (accessed on 1 May 2020).

Multiple sequence alignments were generated using *MAFFT* [72] and edited by *GeneDoc*. We used custom Perl scripts based on the Smith-Waterman algorithm to find genomic copies of SINEs with at least 65% identity and 90% length overlap with the query sequence. After all subfamilies were identified, the genome banks were successively depleted using their consensus sequences and all hits were combined for further analysis.

We considered only ample subfamilies (≥1% of the total number of full-length copies). Subfamilies were identified manually and/or by a domestic script *SubFam*, which generates sequential groups of sequences (typically 100) after a *MAFFT* alignment with reordering (‘reorder’ option). Large-scale separation of subfamilies was a complex task combining several approaches: fuzzy pattern match (*patmatch*) [73] using individually selected indel ± flank sequences; *meshclust* [74] with individually adjusted parameters (largely—id varying around 0.65), and *SubFam*; irrelevant regions (e.g., variable (CG)_n_) were excluded from the analysis. Tribes were identified by *meshclust* with a higher stringency (id 0.95 or 0.99); clusters with similar flanks were eliminated to get rid of truncated dimers.

The mean similarity was determined for 100 randomly selected sequences using the *alistat* program (Eddy S., Cambridge, 2005). The proportion of TSDs was determined by an original algorithm (TSDSearch described at https://sines.eimb.ru/) (accessed on 1 May 2020). The incidences of pol III polyadenylation/termination signals were calculated by analyzing outputs of *patmatch* within regions of interest, which were truncated by the consensus coordinates in numerous multiple alignments.

SINE insertion/deletion loci across genomes were identified by mapping ~200 bp flanking regions of each SINE-containing locus using BWA-MEM [75] The presence or absence of a SINE was inferred from the locus size and manually verified.

The age of young SINE copies was evaluated considering that ~0.8% nucleotides change in 1 My in rodent sequences under no selective pressure [76].

### 4.2. Experimental Methods

The B2-T, B2-C, Sor/B2a-T, Sor/B2a-C, and Ves-Δτ constructs were described previously [31]. Nucleotide substitutions in B2 PASs (AATAAA) were introduced by PCR using B2-T as a template. PCR was also used to generate substitutions in the terminator of B2-C. Amplified DNA fragments were cloned into pGEM-T (Promega, Madison, WI, USA). The τ region of Sor/B2a-T was converted into B2b-specific sequences using the Phusion Site-Directed Mutagenesis Kit (Thermo Fisher Scientific, Waltham, MA, USA). The same method was applied to introduce nucleotide substitutions into the β signal of Ves-Δτ. The plasmids designed for transfection were isolated by the NucleoBond PC 100 kit (Macherey-Nagel, Dylan, Germany).

HeLa cells (ATCC, CCL-2) were grown to an 80%-confluent monolayer in 60 mm Petri dishes using DMEM with 10% fetal bovine serum. Cells on one plate were transfected by 5 μg of plasmid DNA mixed with 10 μL of TurboFect reagent (Thermo Fisher Scientific) according to the manufacturer’s protocol. In this work, we used human cells (HeLa) for transfection because, unlike any mouse cells, they contain no B2 and thus no B2 transcripts. This allows simple monitoring of RNA transcribed from the B2 constructs introduced.

The cellular RNA was isolated 20 h after transfection using the guanidine-thiocyanate method [77]. RNA samples (10 μg) obtained after each transfection were separated by electrophoresis in 6% polyacrylamide gel with 7M urea. Blotting and Northern hybridization with B2-, Ves-, or Sor-specific probes labeled by α[^32^P]-dATP was carried out as described previously [31]. The hybridization signals were quantified by scanning the membranes in a Phosphorimager Cyclone (Packard, USA).

To study the 3ʹ ends of B2 transcripts (Figure 8), RNA from HeLa cells transfected by B2 constructs was used for the synthesis of cDNA, PCR amplification, cloning, and sequencing as described previously [30].

## 5. Conclusions

(i) In T**^+^** SINEs, the minimal terminators include TCT_3_, TAT_3_, and T_4_.
(ii) The TCT_3_ and apparently TAT_3_ remain intact twice as frequently as T_4_ at the 3′-ends of pol III-generated transcripts and hence in the daughter SINE copies.
(iii) The minimal and rudimentary terminators tend to elongate and become efficient as SINE copies get older and their poly(A) tails shorten.

**Figure 16:**
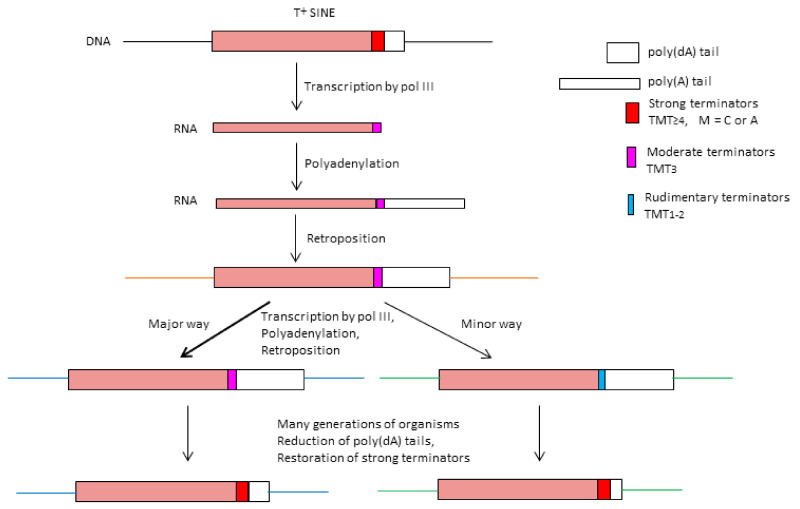
Schematic retrotransposition of T**^+^** SINEs. Following pol III transcription, SINE RNA can be polyadenylated. Terminators can shorten as a result of SINE transcription and retrotransposition. The long-term persistence of SINEs in genomes over many host generations can be due to the poly(A) tail reduction and subsequent terminator elongation (restoration). (See text for further explanation.).

These conclusions substantially extend our model of retrotransposition of T**^+^** SINEs (Figure 16). One or several retrotransposition cycles of a SINE with a strong terminator, TMT_>3_ (M is C or A), shorten it to a moderately efficient TMT_3_. Further retrotransposition events give rise to SINE copies with the same (more often) or shorter nonfunctional terminator (less often). All new SINE copies have long poly(A) tails after polyadenylation of pol III-synthesized transcripts. After many generations of hosts, the poly(A) tails shorten in most of these copies but the terminators or their rudiments elongate and become efficient in some of them. This model explains the maintenance of functional terminators in T^+^ SINEs. The main factors of their maintenance are (1) relatively high capacity of moderately efficient terminators TMT_3_ to persist after retrotransposition and (2) elongation of terminators accompanied by the shortening of poly(A) tails. Insufficient rate of terminator recovery can be one of the factors of SINE decline. On the other hand, an elongation of a rudimentary terminator in a particular retrotranspositionally incompetent copy can restore its activity and give rise to a new SINE tribe or even subfamily.

## Figures and Tables

**Table 1:** B2 subfamilies in rodent genomes.

			B2a	B2b				B2c					B2d			B2e
					B2b1	B2b2	B2b3		B2c1	B2c2	B2c3	B2c4		B2d1	B2d2	
***Mus musculus***	proportion		**63.9%**	**7.8%**				**5.4%**					**20.2%**			**1.6%**
	total number		**~59,013**	**~7203**				**~4987**					**~18,655**			**~1478**
	mean similarity		**83%**	**70%**				**73%**					**62%**			**59%**
	TSDs		**73%**	**64%**				**62%**					**54%**			**23%**
	TCT_3_		****	***				*					*			*
***Rattus norvegicus***	proportion		**71.0%**	**6.2%**				**5.5%**					**15.3%**			**2.0%**
	total number		**~85,872**	**~7499**				**~6652**					**~18,505**			**~2419**
	mean similarity		**81%**	**76%**				**66%**					**59%**			**62%**
	TSDs		**76.9%**	**64.7%**				**43.3%**					**44.6%**			**60.0%**
	TCT_3_		***	**												*
***Cricetulus griseus***	proportion			**46.3%**				**19.4%**	11.7%	3.3%	2.2%	2.2%	**34.3%**			
	total number			**~33,945**				**~14,223**	~8578	~2419	~1613	~1613	**~25,147**			
	mean similarity			**80%**					70%	76%	68%	77%	**63%**			
	TSDs			**76.9%**					67.7%	68.4%	54.5%	69.2%	**58.9%**			
	TCT_3_			***					*			*	*			
***Peromyscus maniculatus***	proportion			**39.5%**	24.7%	9.3%	5.5%	**20.4%**	15.8%		1.8%	2.8%	**40%**			
	total number			**~32,914**	~20,582	~7749	~4583	**~16,999**	~13,166		~1500	~2333	**~33,330**			
	mean similarity				79%	81%	92%		74%		80%	80%	**64%**			
	TSDs				68.8%	80.8%	97.0%		76.6%		81.8%	64.7%	**61.1%**			
	TCT_3_				**	***	****									
***Nannospalax galili***	proportion												**100%**	93.5%	6.5%	
	total number												**~245,653**	~229,686	~15,967	
	mean similarity												**73%**	75%	64%	
	TSDs												**58.5%**	59.0%	57.6%	
	TCT_3_													***		

The incidence of TCTTT in B2 subfamilies is indicated by the number of asterisks. TSDs, Target Site Duplications.

## Data Availability

Not applicable.

## Supplementary Materials

The following are available online at https://www.mdpi.com/article/10.3390/ijms22189897/s1.

## Abbreviations

SINE	Short Interspersed Element
LINE	Long Interspersed Element
PAS	Polyadenylation signal
My	Million years
Mya	Million years ago
RU	Repbase Update
