# Supplementary material for: Analysis of SINE Families B2, Dip, and Ves with Special Reference to Polyadenylation Signals and Transcription Terminators
Source: Int J Mol Sci. 2021 Sep 13;22(18):9897. doi: 10.3390/ijms22189897 (PMC8466645; doi:10.3390/ijms22189897)
Supplement: Supplementary file 1 [file ijms-22-09897-s001.zip › Supplementary Vassetzky/SINE B2. Figs S1-S6. Tables S1-S5/Fig S1.pdf]

β signal

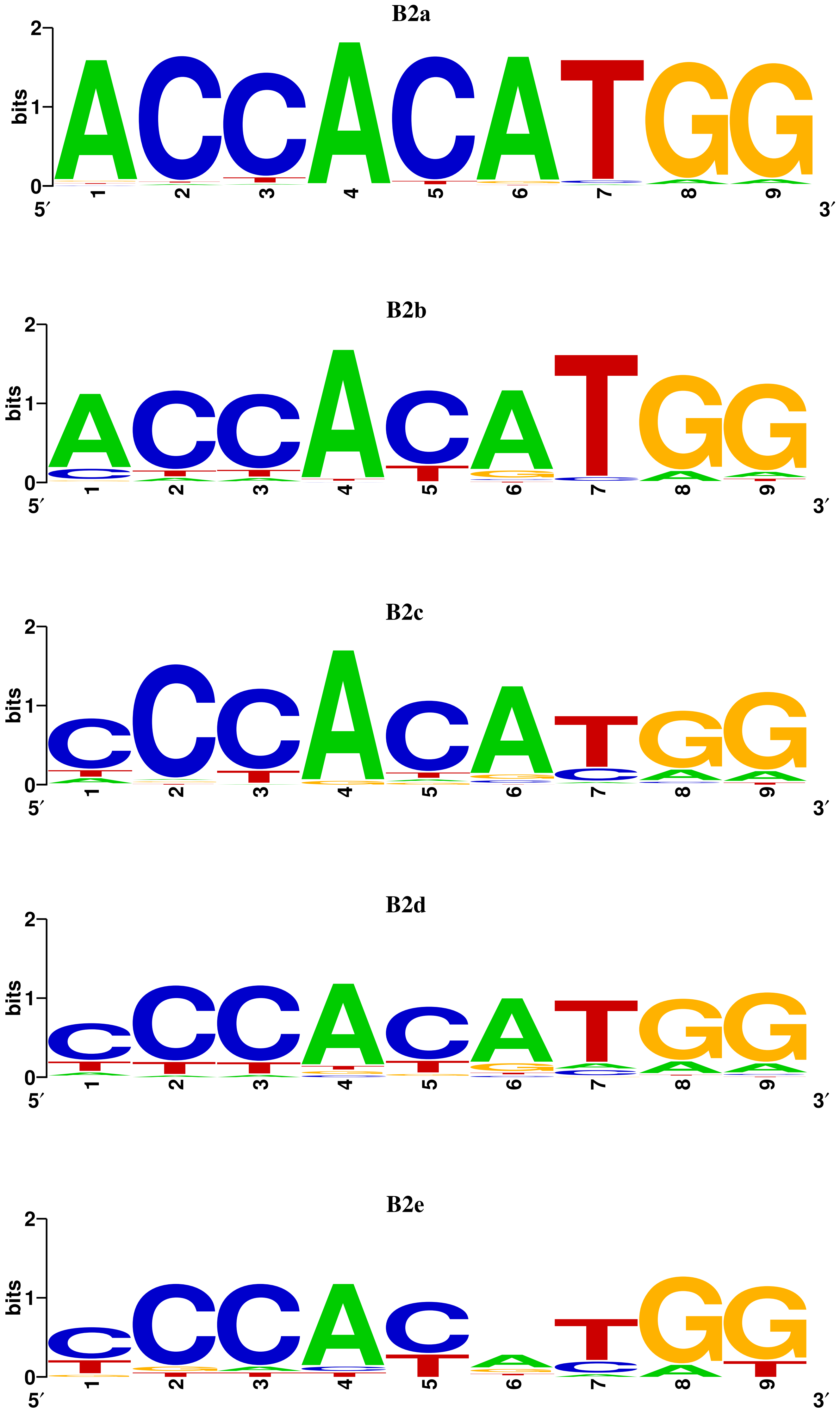

τ signal

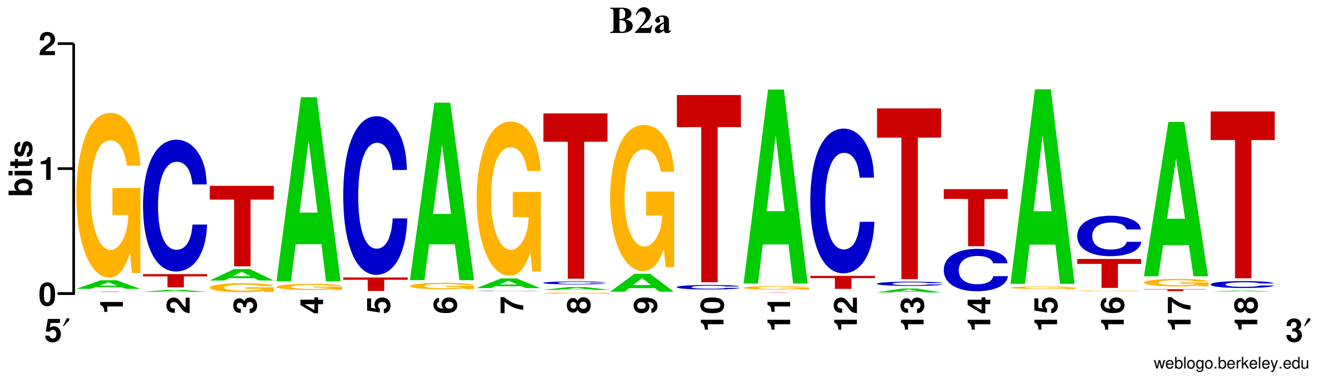

Figure S1. Conservation of β signal in B2 copies of five subfamilies and τ signal in B2a subfamily from the M. musculus genome. Sequence logos were generated by WebLogo 3.1.
