# Supplementary material for: Analysis of SINE Families B2, Dip, and Ves with Special Reference to Polyadenylation Signals and Transcription Terminators
Source: Int J Mol Sci. 2021 Sep 13;22(18):9897. doi: 10.3390/ijms22189897 (PMC8466645; doi:10.3390/ijms22189897)
Supplement: Supplementary file 1 [file ijms-22-09897-s001.zip › Supplementary Vassetzky/SINE B2. Figs S1-S6. Tables S1-S5/Fig S3.pdf]

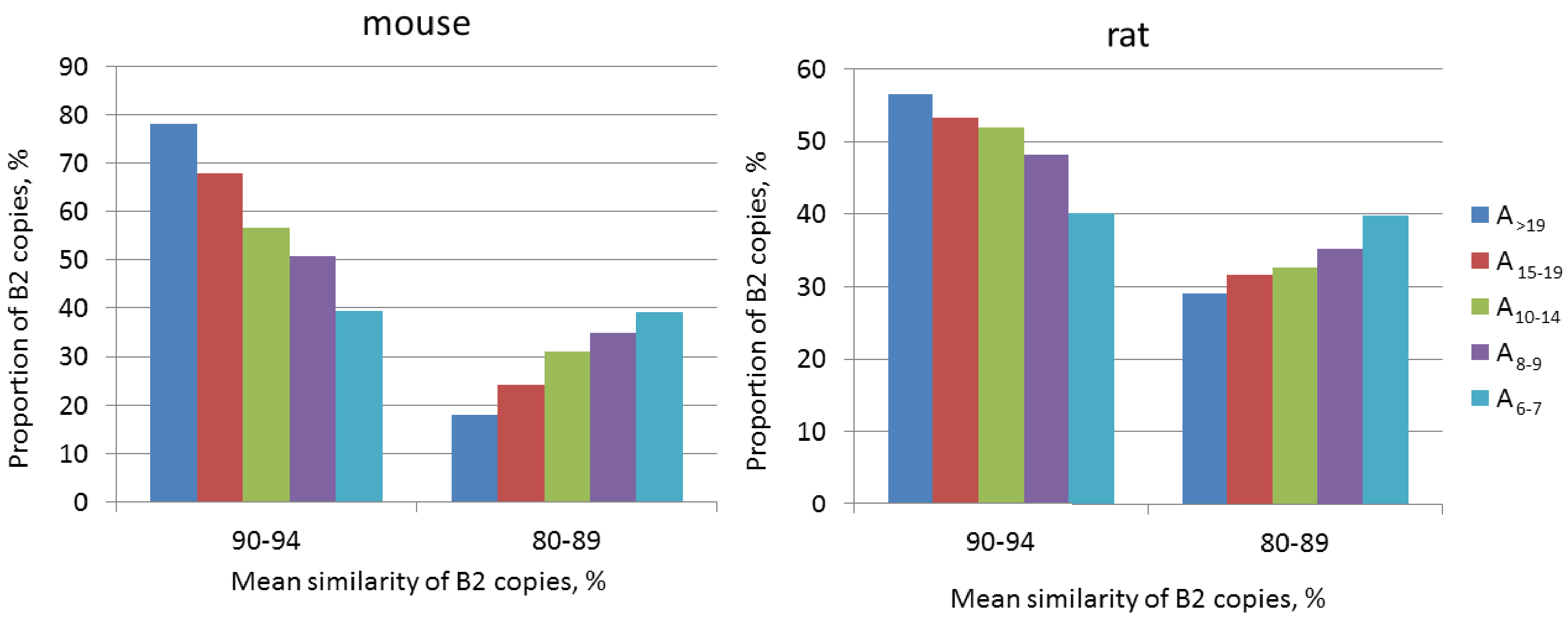

Figure S3. Inverse relationship between the length of oligo(A) tails and age of B2 copies in the mouse and rat genomes. The bars show the percentage of B2 copies with oligo(A) tails of different lengths for copies with the mean similarity of 90-94% and 80-90% (younger and older, respectively).
