# Supplementary material for: Analysis of SINE Families B2, Dip, and Ves with Special Reference to Polyadenylation Signals and Transcription Terminators
Source: Int J Mol Sci. 2021 Sep 13;22(18):9897. doi: 10.3390/ijms22189897 (PMC8466645; doi:10.3390/ijms22189897)
Supplement: Supplementary file 1 [file ijms-22-09897-s001.zip › Supplementary Vassetzky/SINE B2. Figs S1-S6. Tables S1-S5/Fig S4.pdf]

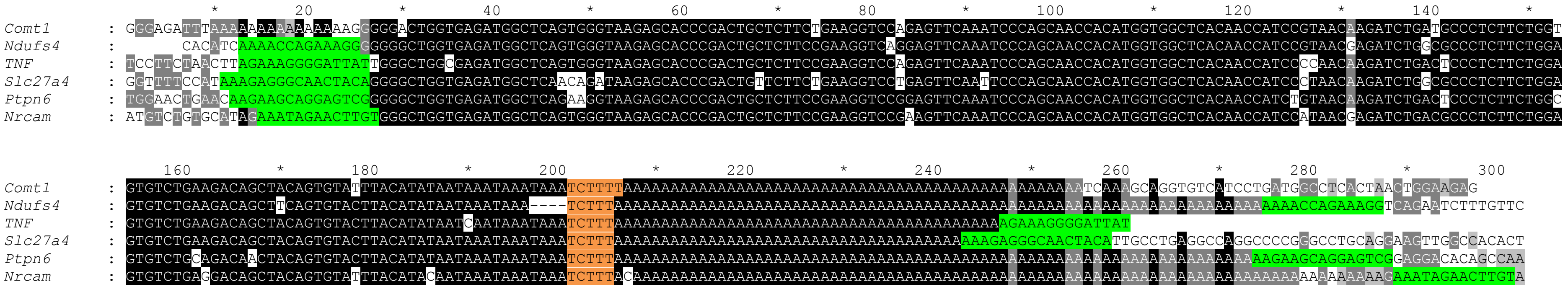

Figure S4. Recently integrated B2 copies that induced gene mutations. The names of B2 copies correspond to the gene names. Terminators and TSDs are highlighted in brown and green, respectively. References to the B2-induced mutations are given below.

Comt1:

Kember RL, Fernandes C, Tunbridge EM, Liu L, Payá-Cano JL, Parsons MJ, Schalkwyk LC (2010). A B2 SINE insertion in the Comt1 gene (Comt1(B2i)) results in an overexpressing, behavior modifying allele present in classical inbred mouse strains. Genes Brain Behav. 2010; 9(8): 925-32.

Ndufs4:

Leong DW, Komen JC, Hewitt CA, Arnaud E, McKenzie M, Phipson B, Bahlo M, Laskowski A, Kinkel SA, Davey GM, Heath WR, Voss AK, Zahedi RP, Pitt JJ, Chrast R, Sickmann A, Ryan MT, Smyth GK, Thorburn DR, Scott HS (2012). Proteomic and metabolomic analyses of mitochondrial complex I-deficient mouse model generated by spontaneous B2 short interspersed nuclear element (SINE) insertion into NADH dehydrogenase (ubiquinone) Fe-S protein 4 (Ndufs4) gene. J Biol Chem. 2012; 287(24): 20652-63.

TNF:

Lacey D, Hickey P, Arhatari BD, O'Reilly LA , Rohrbeck L , Kiriazis H, Du X, Bouillet P. (2015) Spontaneous retrotransposon insertion into TNF 3'UTR causes heart valve disease and chronic polyarthritis. Proc Natl Acad Sci USA, 112: 9698-703

Slc27a4:

Moulson CL, Martin DR, Lugus JJ, Schaffer JE, Lind AC, Miner JH (2003). Cloning of wrinkle free, a previously uncharacterized mouse mutation, reveals crucial roles for fatty acid transport protein 4 in skin and hair development. Proc Natl Acad Sci USA.;100: 5274-9

Ptpn6:

Nesterovitch AB, Szanto S, Gonda A, Bardos T, Kis-Toth K, Adarichev VA, Olasz K, Ghassemi-Najad S, Hoffman MD, Tharp MD, Mikecz K, Glant TT (2011). Spontaneous insertion of a B2 element in the ptpn6 gene drives a systemic autoinflammatory disease in mice resembling neutrophilic dermatosis in humans. Am J Pathol.; 178: 1701-14

Nrcam:

Morelli KH, Seburn KL, Schroeder DG, Spaulding EL, Dionne LA, Cox GA, Burgess RW. (2017) Severity of Demyelinating and Axonal Neuropathy Mouse Models Is Modified by Genes Affecting Structure and Function of Peripheral Nodes. Cell Rep.;18: 3178-3191.

The nucleotide sequence of B2 (Nrcam) was kindly provided by Drs. Robert Burgess and Gregory Cox.
