# Supplementary material for: Analysis of SINE Families B2, Dip, and Ves with Special Reference to Polyadenylation Signals and Transcription Terminators
Source: Int J Mol Sci. 2021 Sep 13;22(18):9897. doi: 10.3390/ijms22189897 (PMC8466645; doi:10.3390/ijms22189897)
Supplement: Supplementary file 1 [file ijms-22-09897-s001.zip › Supplementary Vassetzky/SINE B2. Figs S1-S6. Tables S1-S5/Fig S5.pdf]

chr4:108733470-108733658

C57BL_6NJ	ATCTTTTTTTTAAAAAAAG
NZO_HlLtJ	ATCTTTTTTAAAAAAAAAG
NOD_ShiLtJ	ATCTTTTTTAAAAAAAAAG
FVB_NJ	ATCTTTTTTAAAAAAAAAG
DBA_2J	ATCTTTTTTAAAAAAAAAG
CBA_J	ATCTTTTTTAAAAAAAAAG
C3H_HeJ	ATCTTTTTTAAAAAAAAAG
AKR_J	ATCTTTTTTAAAAAAAAAG
BALB_cJ	ATCTTTTTTAAAAAAAAAG
A_J	ATCTTTTTTAAAAAAAAAG
LP_J	ATCTTTTTTAAAAAAAAAG
129S1_SvImJ	ATCTTTTTTAAAAAAAAAG
WSB_EiJ	ATCTTTTTTAAAAAAAAAG
CAST_EiJ	ATCTT-AAAAAAAAAAAAG
PWK_PhJ	ATCTT-AAAAAAAAAAAAG
SPRET_EiJ	ATCTT-AAAAAAAAAAAAG

chr15:84109854-84110050

C57BL_6NJ	ATCTTTTTTTAAAAAAAAG
NZO_HlLtJ	ATCTTTTTTTAAAAAAAAG
NOD_ShiLtJ	ATCTTTTTTTAAAAAAAAG
FVB_NJ	ATCTTTTTTTAAAAAAAAG
DBA_2J	ATCTTTTTTTAAAAAAA-G
CBA_J	ATCTTTTTTTAAAAAAAAG
C3H_HeJ	ATCTTTTTTTAAAAAAAAG
AKR_J	ATCTTTTTTTAAAAAAAAG
BALB_cJ	ATCTTTTTTTAAAAAAAAG
A_J	ATCTTTTTTTAAAAAAAAG
LP_J	ATCTTTTTTTAAAAAAAAG
129S1_SvImJ	ATCTTTTTTTAAAAAAAAG
WSB_EiJ	ATCTTTTTTTAAAAAAA-G
CAST_EiJ	ATCTTTTTTTAAAAAAA-G
PWK_PhJ	ATCTTTTTTTAAAAAAA-G
SPRET_EiJ	ATCTTAAAAAAAAAAAAA

chr7:80888245-80888435

C57BL_6NJ	ATCTTTTTTAAAAAAT
NZO_HlLtJ	ATCTTTTTTAAAAAAT
NOD_ShiLtJ	ATCTTTTTTAAAAAAT
FVB_NJ	ATCTTTTTTAAAAAAT
DBA_2J	ATCTTTTTTAAAAAAT
CBA_J	ATCTTTTTTAAAAAAT
C3H_HeJ	ATCTTTTTTAAAAAAT
AKR_J	ATCTTTTTTAAAAAAT
BALB_cJ	ATCTTTTTTAAAAAAT
A_J	ATCTTTTTTAAAAAAT
LP_J	ATCTTTTTTAAAAAAT
129S1_SvImJ	ATCTTTTTTAAAAAAT
WSB_EiJ	ATCTTTTTTAAAAAAT
CAST_EiJ	ATCTTT-AAAAAAAAT
PWK_PhJ	ATCTTTTAAAAAAAAT
SPRET_EiJ	ATCTTTTTTAAAAAAT

chr16:17080102-17080296

C57BL_6NJ	ATATTTTTTTAAAAAAAAAG
NZO_HlLtJ	ATATTTTTTTAAAAAAAAAG
NOD_ShiLtJ	ATATTTTTTTAAAAAAAAAG
FVB_NJ	ATATTTTTTTAAAAAAAAAG
DBA_2J	ATATTTTTTTAAAAAAAAAG
CBA_J	ATATTTTTTTAAAAAAAAAG
C3H_HeJ	ATATTTTTTTAAAAAAAAAG
AKR_J	ATATTTTTTTAAAAAAAAAG
BALB_cJ	ATATTTTTTTAAAAAAAAAG
A_J	ATATTTTTTTAAAAAAAAAG
LP_J	ATATTTTTTTAAAAAAAAAG
129S1_SvImJ	ATATTTTTTTAAAAAAAAAG
WSB_EiJ	ATATTTTTTTAAAAAAAAAG
CAST_EiJ	ATATTTTTTTAAAAAAAA-G
PWK_PhJ	ATATTTAAAAAAAAAAAA-G
SPRET_EiJ	ATAAATCTTTAAAAAAAA-G

```
chr12:83518163-83518353

C57BL_6NJ    ATTATTTTAAAAAAG
NZO_HlLtJ    ATTATTTTAAAAAAG
NOD_ShiLtJ   ATTATTTTAAAAAAG
FVB_NJ       ATTATTTTAAAAAAG
DBA_2J       ATTATTTTAAAAAAG
CBA_J        ATTATTTTAAAAAAG
C3H_HeJ      ATTATTTTAAAAAAG
AKR_J        ATTATTTTAAAAAAG
BALB_cJ      ATTATTTTAAAAAAG
A_J          ATTATTTTAAAAAAG
LP_J         ATTATTTTAAAAAAG
129S1_SvImJ  ATTATTTTAAAAAAG
WSB_EiJ      ATTATTTTAAAAAAG
CAST_EiJ     ATTATTTTAAAAAAG
PWK_PhJ       ATATTTTTTAAAAAG
SPRET_EiJ     ATATTTTTTTAAAAG
```

```
chr15:5102826-5103034

C57BL_6NJ    ATCTTTAAAAAAAT
NZO_HlLtJ    ATCTTTAAAAAAAT
NOD_ShiLtJ   ATCTTTAAAAAAAT
FVB_NJ       ATCTTTAAAAAAAT
DBA_2J       ATCTTTAAAAAAAT
CBA_J        ATCTTTAAAAAAAT
C3H_HeJ      ATCTTTAAAAAAAT
AKR_J        ATCTTTAAAAAAAT
BALB_cJ      ATCTTTAAAAAAAT
A_J          ATCTTTAAAAAAAT
LP_J         ATCTTTAAAAAAAT
129S1_SvImJ  ATCTTTAAAAAAAT
WSB_EiJ      ATCTTTAAAAAAAT
CAST_EiJ     ATCTTTAAAAAAAT
PWK_PhJ      ATCTTTAAAAAAAT
SPRET_EiJ    ATCTTTTAAAAAAT
```

```
chr11:97116609-97116793

C57BL_6NJ    ATTAATTTTAAAAAAAG
NZO_HlLtJ    ATTAATTTTAAAAAAAG
NOD_ShiLtJ   ATTAATTTTAAAAAAAG
FVB_NJ       ATTAATTTTAAAAAAAG
DBA_2J       ATTAATTTTAAAAAAAG
CBA_J        ATTAATTTTAAAAAAAG
C3H_HeJ      ATTAATTTTAAAAAAAG
AKR_J        ATTAATTTTAAAAAAAG
BALB_cJ      ATTAATTTTAAAAAAAG
A_J          ATTAATTTTAAAAAAAG
LP_J         ATTAATTTTAAAAAAAG
129S1_SvImJ  ATTAATTTTAAAAAAAG
WSB_EiJ      ATTAATTTTAAAAAAAG
CAST_EiJ     ATTAATTTTAAAAAAAG
PWK_PhJ      ATTAATTTTAAAAAAAG
SPRET_EiJ    ATTAATTTTTTAAAAAG
```

```
chr1:59793467-59793649

C57BL_6NJ    ATCTTTAAAAAAAAAG
NZO_HlLtJ    ATCTTTAAAAAAAAAG
NOD_ShiLtJ   ATCTTAAAAAAAAAAG
FVB_NJ       ATCTTTAAAAAAAAAG
DBA_2J       ATCTTTAAAAAAAAAG
CBA_J        ATCTTTAAAAAAAAAG
C3H_HeJ      ATCTTTAAAAAAAAAG
AKR_J        ATCTTAAAAAAAAAAG
BALB_cJ      ATCTTAAAAAAAAAAG
A_J          ATCTTTAAAAAAAAAG
LP_J         ATCTTTAAAAAAAAAG
129S1_SvImJ  ATCTTTAAAAAAAAAG
WSB_EiJ      ATCTTAAAAAAAAAAG
CAST_EiJ     ATCTTTAAAAAAAAAG
PWK_PhJ      ATCTTTAAAAAAA--G
SPRET_EiJ    ATCTTTTTTAAAAA-G
```

```
chrX:102410389-102410581

C57BL_6NJ    ATCTTTTAAAAAAAAAAG
NZO_HlLtJ    ATCTTTTAAAAAAAAAAG
NOD_ShiLtJ   ATCTTTTAAAAAAAAAAG
FVB_NJ       ATCTTTTAAAAAAAAAAG
DBA_2J       ATCTTTTAAAAAAAAAAG
CBA_J        ATCTTTTAAAAAAAAAAG
C3H_HeJ      ATCTTTTAAAAAAAAAAG
AKR_J        ATCTTTTAAAAAAAAAAG
BALB_cJ      ATCTTTTAAAAAAAAAAG
A_J          ATCTTTTAAAAAAAAAAG
LP_J         ATCTTTTAAAAAAAAAAG
129S1_SvImJ  ATCTTTTAAAAAAAAAAG
WSB_EiJ      ATCTTTTAAAAAAAAAAG
CAST_EiJ     ATCTTT-AAAAAAAAAAG
PWK_PhJ      ATCTTT-AAAAAAAAAAG
SPRET_EiJ    ------------------
```

```
chr14:62928931-62929124

C57BL_6NJ    ATGTTTAAAAAAAAG
NZO_HlLtJ    ATGTTTAAAAAAAAG
NOD_ShiLtJ   ATGTTTAAAAAAAAG
FVB_NJ       ATGTTTAAAAAAAAG
DBA_2J       ATGTTTAAAAAAAAG
CBA_J        ATGTTTAAAAAAAAG
C3H_HeJ      ATGTTTAAAAAAAAG
AKR_J        ATGTTTAAAAAAAAG
BALB_cJ      ATGTTTAAAAAAAAG
A_J          ATGTTTAAAAAAAAG
LP_J         ATGTTTAAAAAAAAG
129S1_SvImJ  ATGTTTAAAAAAAAG
WSB_EiJ      ATGTTTAAAAAAAAG
CAST_EiJ     ATGTTTTAAAAAAAG
PWK_PhJ      ATTTTTAAAAAAAAG
SPRET_EiJ    ATGTTTTTAAAAAAG
```

```
chr14:52153728-52153928

C57BL_6NJ    CTCTTAAAAAAG
NZO_HlLtJ    ATCTTTAAAAAG
NOD_ShiLtJ   ATCTTTAAAAAG
FVB_NJ       ATCTTTAAAAAG
DBA_2J       ATCTTTAAAAAG
CBA_J        ATCTTTAAAAAG
C3H_HeJ      ATCTTTAAAAAG
AKR_J        ATCTTTAAAAAG
BALB_cJ      ATCTTTAAAAAG
A_J          ATCTTTAAAAAG
LP_J         ATCTTTAAAAAG
129S1_SvImJ  ATCTTTAAAAAG
WSB_EiJ      NNCTTTAAAAAG
CAST_EiJ     ATCTTAAAAAAG
PWK_PhJ      ATCTTTAAAAAG
SPRET_EiJ    ATCTTAAAAAAG
```

```
chr15:84109854-84110050

C57BL_6NJ    ATCTTTTTTTAAAAAAAAG
NZO_HlLtJ    ATCTTTTTTTAAAAAAAAG
NOD_ShiLtJ   ATCTTTTTTTAAAAAAAAG
FVB_NJ       ATCTTTTTTTAAAAAAAAG
DBA_2J       ATCTTTTTTTAAAAAAA-G
CBA_J        ATCTTTTTTTAAAAAAAAG
C3H_HeJ      ATCTTTTTTTAAAAAAAAG
AKR_J        ATCTTTTTTTAAAAAAAAG
BALB_cJ      ATCTTTTTTTAAAAAAAAG
A_J          ATCTTTTTTTAAAAAAAAG
LP_J         ATCTTTTTTTAAAAAAAAG
129S1_SvImJ  ATCTTTTTTTAAAAAAAAG
WSB_EiJ      ATCTTTTTTTAAAAAAA-G
CAST_EiJ     ATCTTTTTTTAAAAAAA-G
PWK_PhJ      ATCTTTTTTTAAAAAAA-G
SPRET_EiJ    ATCTTAAAAAAAAAAAAA-
```

chr2:9900243-9900434

C57BL_6NJ	ATCTTTAAAAAAAAAAAAG
NZO_HlLtJ	ATCTTTAAAAAAAAAAAAG
NOD_ShiLtJ	ATCTTTAAAAAAAAAAAAG
FVB_NJ	ATCTTAAAAAAAAAAAAAG
DBA_2J	ATCTTTAAAAAAAAAAAAG
CBA_J	ATCTTTAAAAAAAAAAAAG
C3H_HeJ	ATCTTTAAAAAAAAAAAAG
AKR_J	ATCTTTAAAAAAAAAAAAG
BALB_cJ	ATCTTTAAAAAAAAAAAAG
A_J	ATCTTTAAAAAAAAAAAAG
LP_J	ATCTTTAAAAAAAAAAAAG
129S1_SvImJ	ATCTTTAAAAAAAAAAAAG
WSB_EiJ	ATCTTAAAAAAAAAAAAAG
CAST_EiJ	ATCTTAAAAAAAAAA---G
PWK_PhJ	ATCTTAAAAAAAAAAGAAG
SPRET_EiJ	ATATTAAAAAAAAAA---G

chr1:12272434-12272614

C57BL_6NJ	ATCTTTTAAAAAAAT
NZO_HlLtJ	ATCTTTTAAAAAAAT
NOD_ShiLtJ	ATCTTTTAAAAAAAT
FVB_NJ	ATCTTTTAAAAAAAT
DBA_2J	ATCTTTTAAAAAAAT
CBA_J	ATCTTTTAAAAAAAT
C3H_HeJ	ATCTTTTAAAAAAAT
AKR_J	ATCTTTTAAAAAAAT
BALB_cJ	ATCTTTTAAAAAAAT
A_J	ATCTTTTAAAAAAAT
LP_J	ATCTTTTAAAAAAAT
129S1_SvImJ	ATCTTTTAAAAAAAT
WSB_EiJ	ATCTTTAAAAAAAAT
CAST_EiJ	---------------
PWK_PhJ	---------------
SPRET_EiJ	---------------

chr11:14117171-14117367

C57BL_6NJ	ATTTAAAAAAAAAG
NZO_HlLtJ	ATTTAAAAAAAAAG
NOD_ShiLtJ	ATTTAAAAAAAAAG
FVB_NJ	ATTTAAAAAAAAAG
DBA_2J	ATTTAAAAAAAAAG
CBA_J	ATTTAAAAAAAAAG
C3H_HeJ	ATTTAAAAAAAAAG
AKR_J	ATTTAAAAAAAAAG
BALB_cJ	ATTTAAAAAAAAAG
A_J	ATTTAAAAAAAAAG
LP_J	ATTTAAAAAAAAAG
129S1_SvImJ	ATTTAAAAAAAAAG
WSB_EiJ	ATTTAAAAAAAAAG
CAST_EiJ	ATTAAAAAAAA--G
PWK_PhJ	ATTTAAAAAAAAAG
SPRET_EiJ	ATTTTTAAAAAAAG

chr16:17919203-17919386

C57BL_6NJ	ATCTTTTTTAAAAAAAG
NZO_HlLtJ	ATCTTTTTTAAAAAAAG
NOD_ShiLtJ	ATCTTTTTTAAAAAAAG
FVB_NJ	ATCTTTTTTAAAAAAAG
DBA_2J	ATCTTTTTTAAAAAAAG
CBA_J	ATCTTTTTTAAAAAAAG
C3H_HeJ	ATCTTTTTTAAAAAAAG
AKR_J	ATCTTTTTTAAAAAAAG
BALB_cJ	ATCTTTTTTAAAAAAAG
A_J	ATCTTTTTTAAAAAAAG
LP_J	ATCTTTTTTAAAAAAAG
129S1_SvImJ	ATCTTTTTTAAAAAAAG
WSB_EiJ	ATCTTTTTTAAAAAAAG
CAST_EiJ	ATCTTT-AAAAAAAAAG
PWK_PhJ	ATCTTTTTTAAAAAAAG
SPRET_EiJ	-----------------

chr13:17991337-17991525

```
C57BL_6NJ    ATCTTTTTTTAAAGAAAAAAG
NZO_HlLtJ    ATCTTTTTTTAAAGAAAAAAG
NOD_ShiLtJ   ATCTTTTTTTAAAGAAAAAAG
FVB_NJ       ATCTTTT-AAAAAAAAAAAAG
DBA_2J       ATCTTTTTTTAAAGAAAAAAG
CBA_J        ATCTTTTTTTAAAGAAAAAAG
C3H_HeJ      ATCTTTTTTTAAAGAAAAAAG
AKR_J        ATCTTTTTTTAAAAAAAAAAG
BALB_cJ      ATCTTTTTTTAAAGAAAAAAG
A_J          ATCTTTTTTTAAAGAAAAAAG
LP_J         NTCTTTTTTTAAAGAAAAAAG
129S1_SvImJ  ATCTTTTTTTAAAGAAAAAAG
WSB_EiJ      ATCTTTTTTTAAAGAAAAAAG
CAST_EiJ     ATCTTTTTTTAAAAAAAAAAG
PWK_PhJ      ATCTTT---AAAAAAAAAAAG
SPRET_EiJ    ---------------------
```

Figure S5. Examples of polymorphic terminators in B2 copies. Original terminator nucleotides are in blue and additional T residues are in red. Positions of B2 copies in the mouse genome (strain C57BL/6B, assembly GRCm38/mm10) are shown above. SPRET_EiJ – *Mus spretus*. WSB_EiJ, CAST_EiJ, and PWK_PhJ are *Mus musculus* subspecies: *M. m. domesticus*, *M.m. castaneus*, *M. m. musculus*, respectively
