# Supplementary material for: Analysis of SINE Families B2, Dip, and Ves with Special Reference to Polyadenylation Signals and Transcription Terminators
Source: Int J Mol Sci. 2021 Sep 13;22(18):9897. doi: 10.3390/ijms22189897 (PMC8466645; doi:10.3390/ijms22189897)
Supplement: Supplementary file 1 [file ijms-22-09897-s001.zip › Supplementary Vassetzky/SINE B2. Figs S1-S6. Tables S1-S5/Table S1.pdf]

Table S1. Random B2a copies in genomes of 1 or 2 mouse strain(s)

#	Chromosome: position	Strains	Tail with a terminator	Terminator	A-rich tail length, nt
1	chr11:107581768-107581957	C57BL_6NJ NZO_HlLtJ	TCTAAAAAAAAAAAAAAAAAAAAAAAAAAAGAAA	TCT	30
2	chr17:24098897-24099087	C57BL6NJ	TCTTTAAAAAAAAAAAAAAAAAAAAAAAAAAAAAGAAAG	TCTTT	34
3	chr8:22496673-22496881	C57BL_6NJ NZO_HlLtJ	TCTTTAAAAAAAAACAAAAAAAAAAAAA	TCTTT	23
4	chr17:35851355-35851546	C57BL_6NJ	TCTTTAAAAAAAAAAAAAAAAAAAAAAAAA	TCTTT	25
5	chr4:19614863-19615050	C57BL_6NJ	TCTTTAAAAAAAAAAATTTAAAAAAAACAAAAAAAAAAAAAC AAAAAAAAAAAAACAAAAAAACAAAACAAAAAAACAAAAAAA CAAAAAAAAAAAAAAAAGAAA	TCTTT	95
6	chr14:47698157-47698350	C57BL_6NJ	TCTTTAAAAAAAAAAAAAAAAAAAAAAAAAAAAAAGAGAACA	TCTTT	37
7	chr3:88684620-88684810	C57BL_6NJ	TCTTTAAAAAAAAAAAAA	TCTTT	13
8	chr4:108108803-108108995	C57BL_6NJ	TCTTTAAAAAAAAAAAAAAAAAAAA	TCTTT	20
9	chr13:119787991-119788182	C57BL_6NJ	TCTTTAAAAAAAAAAAAAAAAAAGAA	TCTTT	21
10	chr16:30291696-30291892	C57BL_6NJ	TCTTAAAAAAAAATTGTCAAAAAAAAAAAAAAAAA	TCTT	30
11	chr19:45985835-45986024	C57BL_6NJ	TCTTTAAAAAAAAAAAA	TCTTT	12
12	chr2:119206500-119206696	C57BL_6NJ	TCTTAAAAAAAAAAAAATAAAATAAAATAAAATAAAATATA	TCTT	37
13	chr7:45359014-45359202	C57BL_6NJ	TCTTTAAAAAAAAAAAAAAAAAAAAAAAAAAAAAAAAAAAAAAA AAAAAAAAAAAAAAA	TCTTT	51
14	chr16:32207050-32207238	C57BL_6NJ	TCTTTAAAAAAAAAAAAAAAAAAAAAAAAAAAAAAAAAAAAA AAA	TCTTT	38
15	chr12:28830883-28831075	C57BL_6NJ	TTAAAAAAAAAAAAAAAAAAA	TT	19
16	chr9:71923639-71923826	C57BL_6NJ	TCTTTAAAAAAAAAAAAAAAA	TCTTT	16
17	chr17:29977257-29977448	C57BL_6NJ	TCTTTAAAAAAAAAAAAAAAAAAATCCAAAATTTAAAAAAA AAAAAAAAAAAAA	TCTTT	49
18	chr11:17975352-17975543	C57BL_6NJ	TCTTTAAAAAAAAAAAAAAAAAAAAAAAAAAAAAAAAAAAAAAA AAAAAAAAAAA	TCTTT	47
19	chr7:38135206-38135392	C57BL_6NJ NZO_HlLtJ	TCTTTAAAAAAAA	TCTTT	8
20	chr3:89124648-89124838	C57BL_6NJ	TCAAAAAAAAAAAAAAAAAAAGAAAAAAAAAAAAA	TC	33
21	chr4:122867123-122867318	C57BL_6NJ	TCTTTAAAAAAAAAAAAAAAAA	TCTTT	17
22	chr12:101992989-101993180	C57BL_6NJ	TCTTTAAAAAAAAAAAAAAAAAA	TCTTT	18
23	chr2:153596013-153596202	C57BL_6NJ NZO_HlLtJ	TCAAAAAAAAAAAAAAGAAAAAAAAA	TC	24
24	chr9:72779435-72779644	C57BL_6NJ	TCTTTAAAAAAAAAAAAAAAAAAAAAAAAAA	TCTTT	26
25	chr1:172270895-172271085	C57BL_6NJ	TCTTTTAAAAAAAAAAAAAAAA	TCTTTT	16
26	chr8:71673632-71673822	C57BL_6NJ	TCTTAAAAACAAAAAAAAAAAAAA	TCTT	20
27	chr15:77546233-77546424	C57BL_6NJ	TCTTTAAAAAAAAAAAAAATAAATCTTTAAAAAAAAAAAAAAAA AAAAAAAAAAAAA	TCTTT	32 (54)
28	chr11:31275722-31275914	C57BL_6NJ	TCTTTAAAAAAAAAAAAAAAAAAA	TCTTT	19
29	chr4:133627440-133627635	C57BL_6NJ NZO_HlLtJ	TTTAAAAAAAAAAAAAA	TTT	14
30	chr11:69724024-69724214	C57BL_6NJ	TCTTTAAAAAAAAAAAA	TCTTT	12
31	chr2:120329602-120329794	C57BL_6NJ	TCTTTAAAAAAAAAAAAGAA	TCTTT	15
32	chr17:55602395-	C57BL_6NJ	TCTTTAAAAAAAAAAAAAAAAAAAA	TCTTT	20

	55602587	NZO_HlLtJ			
33	chr7:29191804-29191996	C57BL_6NJ	TCTAAAAAAAAAGAAAAAAAAAAAA	TCT	23
34	chr4:108846560-108846747	C57BL_6NJ NZO_HlLtJ	TCTTTAAAAAAAAAAAAA	TCTTT	13
35	chr1:128365174-128365366	C57BL_6NJ NZO_HlLtJ	TCTTAAAAAAAAAAAAAAAAAAAAAAAA	TCTT	24
36	chr9:65978531-65978723	C57BL_6NJ	TCTTTAAAAAAAAAAAAAAAAAAAAAAAAAAA	TCTTT	27
37	chr4:124622165-124622353	C57BL_6NJ	TCTTTAAAAAAAAAAAAAAAAAA	TCTTT	18
38	chr8:70524099-70524287	C57BL_6NJ	TCTTAAAAAAAAAAAAAAAAA	TCTT	17
39	chr7:80163259-80163451	C57BL_6NJ	TCTTTAAAAAAAAAAAAAAAAAAAA	TCTTT	20
40	chr12:101888961-101889151	C57BL_6NJ	TCTTTAAAAAAAAAAAA	TCTTT	12
41	chr1:93266017-93266209	C57BL_6NJ NZO_HlLtJ	TCTTAAAAAAAAAAAAAAAAAAAAAAAAAAA	TCTT	27
42	chr8:72278028-72278220	C57BL_6NJ	TCTTTTAAAAAAATAAAAAAATAAAAAAATAAAAAAATAAAAATAAAAA CGAACGAACGAACGAACGAGAGAGAGAGAGAGAGAGAGAGAGAGAGA GAAAGAAAGAGA	TCTTTT	102
43	chr2:104608551-104608744	C57BL_6NJ NZO_HlLtJ	TCTTTATTAAAAAAAAAAATAAAATAAAA	TCTTT	25
44	chr1:88696968-88697155	C57BL_6NJ	TTTTAAAAAAAAAAAAAAAAAAAAAAA	TTTT	23
45	chr14:45823218-45823413	C57BL_6NJ	TCTTTAAAAAAAAATAAA	TCTTT	13
46	chr1:168396353-168396549	C57BL_6NJ	TCTTTAAAAAAAAAAAAAAAAAAAA	TCTTT	20
47	chr9:65978531-65978723	C57BL_6NJ	TCTTTAAAAAAAAAAAAAAAAAAAAAAAAAAA	TCTTT	27
48	chr1:101579171-101579363	C57BL_6NJ NZO_HlLtJ	TCTTAAAAAAAAAAAAAAA	TCTT	15
49	chr2:128759494-128759686	C57BL_6NJ	TCTTTAAAAAAAAAAAAAAAAAAAA	TCTTT	20
50	chr17:74345036-74345221	C57BL_6NJ	TTAAAAAAAAAAAA	TT	12
51	chr11:75691621-75691816	C57BL_6NJ	TCTTTAAAAAAAAAAAAAAAAAAAAAA	TCTTT	22
52	chr2:119620822-119621008	C57BL_6NJ	TCTTAAAAAAAAAAAAAAAAAAAAAAAA	TCTT	24
53	chr11:75691621-75691816	C57BL_6NJ	TCTTTAAAAAAAAAAAAAAAAAAAAAAAAAA	TCTTT	26
54	chr11:75691621-75691816	C57BL_6NJ	TCTTTAAAAAAAAAAAAAAAAAAAAAA	TCTTT	22
55	chr12:69302828-69303016	C57BL_6NJ	TCTTTAAAAAAAAAAAAAAAAAAA	TCTTT	19
56	chr14:81656468-81656817	C57BL_6NJ	TCTTTAAAAAAAAAAAAAAAAAAAAAAA	TCTTT	23
57	chr14:81656598-81656787	C57BL_6NJ	TCTTAAAAAAAAATTAAAAAAAAA	TCTT	20
58	chr8:60684661-60684852	C57BL mm10	TCTTTAAAAAAAAAAAAAAAAAAAAAAAAAAA	TCTTT	27
59	chr13:107965195-107965386	C57BL_6NJ	TCAAAAAAAAAAAAAAAAAAA	TC	19
60	chr5:146270094-146270286	C57BL_6NJ	TCTTTAAAAAAAAAAAAAAAAAAAA	TCTTT	20
61	chr19:24308736-24308924	C57BL_6NJ	TCTTAAAAAAAAAAAAAAAAAAAAA	TCTT	21
					Mean length= 25.6

Terminator statistics

Sequences	Number	Fraction
T>3	3	4.9%
TCTTT	40	65.6%
TCTT	10	16.4%
Others	8	13.1%
