# Supplementary material for: Analysis of SINE Families B2, Dip, and Ves with Special Reference to Polyadenylation Signals and Transcription Terminators
Source: Int J Mol Sci. 2021 Sep 13;22(18):9897. doi: 10.3390/ijms22189897 (PMC8466645; doi:10.3390/ijms22189897)
Supplement: Supplementary file 1 [file ijms-22-09897-s001.zip › Supplementary Vassetzky/SINE B2. Figs S1-S6. Tables S1-S5/Table S2.pdf]

Table S2. Random B2a copies in genomes of 3 to 10 mouse strains

#	Chromosome: position	Number of strains	Tail with a terminator	terminator	A-rich tail length, nt
1	chr18:5350773-5350961	5	TCTTAAAAAAAAAAAA	TCTT	12
2	chr18:76833638-76833828	7	TCTTTAAAAAAAAAAAAAAAAAAAAA	TCTTT	21
3	chr19:6290666-6290856	9	TCTTTAAAAAAAAAAAAAAAAAAAAAAA	TCTTT	23
4	chr19:24470737-24470926	4	TCTTTAAAAAAAAAAAA	TCTTT	12
5	chr14:11964242-11964434	5	TCTTTAAAAAAAAAAAAAAA	TCTTT	15
6	chr14:32331245-32331425	10	TAAAAAAAAAAAAAA	T	14
7	chr1:59784199-59784390	9	TCTTTAAAAAAAAAAAAA	TCTTT	13
8	chr5:62439904-62440095	7	TCTTTAAAAAAAAAAAAAAAAAAAAAAAAA	TCTTT	25
9	chr11:116169755-116169946	9	TCTTTAAAAAAAAAAAAAAAAAAAAAA	TCTTT	22
10	chr16:84882195-84882386	10	TCTAAAAAAAAAAAAAAAAAAAAAAAAAAAAAAAAAAAAA	TCT	37
11	chr6:5841131-5841321	9	TCTTTAAAACAAACAAACAAAAC	TCTTT	18
12	chr15:29283358-29283547	8	TCTTTAAAAAAAAAAAA	TCTTT	12
13	chr15:75850595-75850787	10	TCTTTAAAAAAAAAAAAAA	TCTTT	14
14	chr16:15583691-15583883	10	TCTTTAAAAAAAAAAAAAAAAAAAAAAAA	TCTTT	24
15	chr13:63501760-63502051	4	TCTTTAAAAAAAAAAAAA	TCTTT	13
16	chr19:22807962-22808148	9	TCTTTAATAAAAAAAAA	TCTTT	12
17	chr1:38072204-38072394	10	TCTTTAAAAAAAAAAAAAAAAAAAAAAAAAAAAAAAAAA	TCTTT	34
18	chr2:30389863-30390054	6	TCTTTAAAAAAAAAAAAAAAAAAAA	TCTTT	20
19	chr14:36064739-36064929	8	TCTTAAAAAAAAAAAAAAAAAAA	TCTT	19
20	chr4:147855313-147855504	7	TTAAAAAAAAAA	TT	10
21	chr1:191971975-191972166	10	TCTTTAAAAAAAAAA	TCTTT	10
22	chr6:72675395-72675585	10	TCTTTAAAAAAAAAAA	TCTTT	11
23	chr5:106796937-106797129	4	TCTTAAAAAAAAAAAAAAAAAAA	TCTT	19
24	chr11:103834303-103834494	4	TCTTTAAAAAAAAAAAAAAAAAAAAAAAAA	TCTTT	25
25	chr6:47587296-47587488	4	TCTTTAAAAAAAAAAAAAAAAAAAAAAAAAAAAAA	TCTTT	30
26	chr2:128296655-128296846	7	TCTTAAAAAAAAAAAAAAAAAA	TCTT	18
27	chr11:93691668-93691858	9	TTTTTTTAAAAAAAA	TTTTTTT	8
28	chr14:14214287-14214478	8	TCTTAAAAAAAAAAAAGAAAAGAAAAA	TCTT	23
29	chr5:106796837-106797159	4	TCTTAAAAAAAAAAAAAAAAAAA	TCTT	19
30	chr5:106796937-106797129	10	TTTAAAAAAAAAAAAAAAA	TTT	16
31	chr6:72531340-72531534	5	TCTAAAAAAAAAAAAAAAA	TCT	16
32	chr4:137398263-137398455	4	TCTTTAAAAAAAAAAAAAAAAA	TCTTT	17

33	chr6:48683691-48683881	4	TCTTTAAAAAAAAAAAA	TCTTT	12
34	chr2:71398736-71398924	8	TCTTTAAAAAAAAAA	TCTTT	10
35	chr4:129475034-129475226	10	TCTTTAAAAAAAAAAAAAAAAAAAGAAAGAAA	TCTTT	27
36	chr12:55074054-55074243	4	TCAAAAAAAAAAAAAAAAAAAAAAAAAAAA	TC	28
37	chr6:49065772-49065956	5	TCTTTAAAAAAAAAA	TCTTT	10
38	chr6:49065772-49065956	10	TCTTTAATAAAAAAAAAAAAAAAAA (?)	TCTTT	20
39	chr9:72292349-72292551	8	TCTTTAAAAAAAAAAAAAAAA	TCTTT	16
40	chr1:179682712-179682901	9	TCTAAAAAAAAAAAAAAAAAAAAAAAAAAAAAAAAA	TCT	33
41	chr4:99537248-99537439	7	TCTTTAAAAAAAAAAAAA	TCTTT	13
42	chr3:73398294-73398486	10	TCTTTAAAAAAAAAAAAAA	TCTTT	14
43	chr2:126998854-126999044	7	TCTAAAAAAAAAAAAAA	TCT	14
44	chr15:85054463-85054655	7	TCTTTAAAAAAAAAAAAAAAAAAAAAAAAAAAAA	TCTTT	29
45	chr12:110644921-110645112	5	TCTTTAAAAAAATAAAAATAAAAA	TCTTT	19
46	chr11:120153752-120153943	7	TCTATAAAAAAAAAAAAAAAAAAAAAAAAAAAAAAAAAAAAA	TCT	38
47	chr18:14676022-14676213	9	TCTTTAAAAAAAAAAAAAAATAAAAAA	TCTTT	22
48	chr10:80045312-80045503	10	TCTTTAAAAAAAAAAAAAAAAAAAAAAAAAAA	TCTTT	27
49	chr19:32738436-32738628	4	TCTTAAAAAAAAAAAAAAAAAAAAAA	TCTT	22
50	chr14:56134393-56134588	6	TCTTAAAAAAAAAAAAAAAAAAA	TCTT	19
51	chr10:127467358-127467550	4	TCTTTAAAAAAAAAAAAAAAAAAAAAAA	TCTTT	23
52	chr4:137760116-137760302	4	TCTTTAAAAAAAA	TCTTT	8
53	chr1:46785922-46786114	3	TCTTAAAAAAAAAAAAAAAAAAAAAAAAAAAAAAAAAAAAAAA	TCTT	39
54	chr2:153173361-153173550	10	TCTTAAAAAAAAAAAAAAAAAAAAAAAAAAAAAAAAAAAAAA	TCTT	38
55	chr1:166960683-166960876	10	TCTTTAAAAAAAAAAAAAA	TCTTT	14
56	chr1:166960553-166960906	10	TCTTTAAAAAAAAAAAAAA	TCTTT	14
57	chrX:74357261-74357579	9	TTAAAAAAAAAAAAAAAA	TT	16
58	chr2:153173361-153173550	10	TCTTAAAAAAAAAAAAAAAAAAAAAAAAAAAAAAAAAAAAAA	TCTT	38
59	chr7:99431627-99431856	9	TTTAAAAAAAAAAAAAAAAAAAAAAAAAA	TTT	26
					Mean length = 19.9

Terminator statistics

Sequences	Number	Fraction
T>3	1	1.7%
TCTTT	36	61.0%
TCTT	11	18.6%
Others	11	18.6%
