# Supplementary material for: Analysis of SINE Families B2, Dip, and Ves with Special Reference to Polyadenylation Signals and Transcription Terminators
Source: Int J Mol Sci. 2021 Sep 13;22(18):9897. doi: 10.3390/ijms22189897 (PMC8466645; doi:10.3390/ijms22189897)
Supplement: Supplementary file 1 [file ijms-22-09897-s001.zip › Supplementary Vassetzky/SINE B2. Figs S1-S6. Tables S1-S5/Table S3.pdf]

Table S3. Random B2a copies in genomes of 11 to 15 mouse strains

#	Chromosome: position	Number of strains	Tail with a terminator	Terminator	A-rich tail length, nt
1	chr11:51715360-51715550	13	TCTTTAAAAAAAAAA	TCTTT	10
2	chr13:43664040-43664235	12	TCTTTAAAAAAAAAAAAAAAAA	TCTTT	17
3	chr13:66942593-66942777	13	TTAAAAAA	TT	6
4	chr14:30578497-30578683	13	TTTAAAAAAAAA	TTT	9
5	chr14:30578497-30578683	13	TCTTAAAAA	TCTT	5
6	chr14:30578497-30578683	11	TCTTAAAAAAAAAAAAAAAAAAAA	TCTT	20
7	chr18:10297708-10297895	15	TCTTAAAAAAAAAAA	TCTT	11
8	chr19:47606649-47606832	13	TTAAAAAAAACAAAA	TT	13
9	chr14:24531504-24531696	13	TCTTAAAAAAAAAGAAAAAAAAAAGAAAAA	TCTT	26
10	chr14:27207291-27207481	13	TCTTTAAAAAAAAAAAAAAA	TCTTT	15
11	chr14:30062273-30062465	13	TCTTTAAAAAAAAAAAAAA	TCTTT	14
12	chr14:30578497-30578683	13	TTTAAAAAAAAACAAA	TTT	13
13	chr14:31490385-31490575	13	TCTTTAAAAAAAAAAAAAAAAAAA	TCTTT	19
14	chr14:35691462-35691647	11	TCTTAAAAAAAA	TCTT	8
15	chr2:18412592-18412770	13	TCTTTAA	TCTTT	2
16	chr2:29597710-29597901	13	TCTTTAAAAAAAAAA	TCTTT	10
17	chr3:64173540-64173724	15	TCTTTAAAAAAAAA	TCTTT	9
18	chr5:24397428-24397619	11	TCTTTAAAAAAAAAAAAAAA	TCTTT	15
19	chr9:22407820-22408014	11	TCTTAAAAAAAAAATTGAAAAAAAA	TCTT	21
20	chr9:32195046-32195237	13	TCTTAAAAAAAAAAAA	TCTT	12
21	chr9:44053720-44053926	15	TCTTAAAAAAAAAAAAAAA	TCTT	15
22	chrY:90759898-90760086	14	TCTTTAAAAAAAAAAA	TCTTT	11
23	chr10:4351213-4351404	12	TCTTTAAAAAAAAAAAAAAAAAA	TCTTT	18
24	chr10:95535916-95536111	15	TCTTTAAAAAAAAAAAAAA	TCTTT	14
25	chr11:23823909-23824100	12	TCTTTAAAAAAAAAAAAAAAAAAAAAAA	TCTTT	23
26	chr12:94096263-94096454	11	TCTTAAAAAAAAAAAAAAA	TCTT	15
27	chr13:18527215-18527405	12	TAAAAAAAAA	T	9
28	chr17:88152809-88152999	14	TCTTAAAAAAAAAAAAA	TCTT	13
29	chr11:107232676-107232866	15	TCTTTAAAACAAACAAACAAAACAAAACAAAA	TCTTT	27
30	chr3:88683096-88683288	15	TCTTTAAAAAAAAAAAA	TCTTT	12
31	chr6:91874847-91875036	13	TCTTTAAAAAAAAAAAA	TCTTT	12
32	chr13:23540051-23540241	15	TCTTTAAAAAAAAAAAAA	TCTTT	13

33	chr17:26675628-26675819	15	TCTTAAAAAAAAAA	TCTT	10
34	chr17:29113145-29113334	15	TCTTAAAAAAAA	TCTT	8
35	chr18:10574475-10574666	15	TCTTTAAAAAAAAAAAAA	TCTTT	13
36	chr9:119310815-119311007	14	TCTTTAAAAAAAAAAAAAA	TCTTT	14
37	chr12:13001564-13001755	13	TCTTTAAAAAAAAAAAAAA	TCTTT	14
38	chr14:31554676-31554883	15	TCTTTAATAAATAAATAAATAAATAAATAAAT	TCTTT	27
39	chr18:34355901-34356093	13	TCTTTAAAAAAAAAAAAAA	TCTTT	14
40	chr1:106181690-106181880	13	TCTTTTAAAAAAAAAAAA	TCTTTT	12
41	chr14:21643225-21643417	12	TCTTTAAAAAAAAAAAAAAAAAAAAAAAAAAAAAAA	TCTTT	31
42	chr6:51074276-51074464	13	TCTTTAAAAAAAAAAAAAA	TCTTT	14
43	chr11:97846758-97846939	14	TCTTTAAAAAAA	TCTTT	7
44	chr8:22609531-22609714	15	TCTTTAAAAAAAAA	TCTTT	9
45	chr8:114337906-114338100	13	TCTTAAAAAAAAAAAAAA	TCTT	14
46	chrX:140970475-140970666	13	TCTTTAAAAAAAAAAAAA	TCTTT	13
47	chr14:45544697-45544887	15	TCTTAAAAAAAAAAAAA	TCTT	13
48	chr15:77411890-77412081	13	TCTTTAAAAAAAAAAAAA	TCTTT	13
49	chr6:113350910-113351100	15	TCTTAAAAAAAAA	TCTT	9
50	chr11:107301265-107301438	12	TAAAAAAAAAAAAA	T	13
51	chr1:131913063-131913254	15	TCTTAAAAAAAAA	TCTT	9
52	chr3:83998650-83998840	13	TCTTAAAAAAAAAAAGAA	TCTT	14
53	chr6:117878239-117878427	12	TCTTTAAAAAAA	TCTTT	7
54	chrX:95069064-95069254	15	TCTTTAAAAAAAAAAAA	TCTTT	12
55	chrX:155253601-155253780	15	TCTTTAATTTATTTAAAAATAAATAAATAAATAAA	TCTTT	30
56	chr10:67162435-67162625	13	TCTTTAAAAAAAAAAAAA	TCTTT	13
57	chr11:106126950-106127141	13	TCTTTAAAAAAAAAAAGAAAGAAAAGAAAAGCTA TTAAAAAAAAAAAAAAAA	TCTTT	47
58	chr11:107289242-107289437	11	TCTTTAAAAAAAAAAAAAA	TCTTT	14
59	chr12:31096211-31096402	12	TCTTTAAAAAAAAAAAAAAAA	TCTTT	16
60	chr14:34331392-34331581	15	TCTAAAAAAATAAAAAAAATAAAAAAA	TCT	24
61	chr17:26109816-26110008	15	TCTTAAAAAAAAAAAAAAAAA	TCTT	17
62	chr3:20009951-20010141	13	TCTTTAAAAAAAAAAAAAAAAAAAAAAAAAAAAAA	TCTTT	30
63	chr4:36958065-36958254	15	TCTTTAAAAAAAGAAAAAGAAAT	TCTTT	18
64	chr6:49447538-49447725	15	TCTTTAAAAAAAAAAACAAAAAA	TCTTT	18
65	chr11:20091033-20091222	15	TAAAAATTAAAAAAAAAAAAAGAAAAAA	T	27
66	chr11:107330097-107330289	15	TTTAAAAAAAAAAGAAA	TTT	14
67	chr3:79625435-79625623	14	TCTTTAAAAAAAAAAAA	TCTTT	12
68	chr7:126743892-126744082	15	TCTTAAAAAAAAAAA	TCTT	11

69	chr8:85706724-85706912	15	TTTAAAAAAAAAA	TTT	10
70	chr15:77843869-77844059	13	TCTTTAAAAAAAAAAAAAAAAAA	TCTTT	18
71	chr18:46928983-46929173	12	TCTTTAAAAAAACAAAAAACAAAAAAAAA	TCTTT	24
72	chr11:107380577-107380769	13	TTAAAAAAAAA	TT	9
73	chr2:130916236-130916423	15	TCTTTAAAAAAAAAAA	TCTTT	11
74	chr2:127151968-127152159	11	TCTTAAAAAAAAAAAAA	TCTT	13
75	chr3:20523309-20523501	12	TCTTTAAAAAAAAAAAAGAAAA	TCTTT	16
76	chr3:51535076-51535265	13	TCTTTAAAAAAAAAAAA	TCTTT	12
77	chr4:104474978-104475165	15	TCTTTAAAAAAA	TCTTT	7
78	chr5:87028163-87028353	13	TCTTAAAAAAAAAAAAAAAA	TCTT	16
79	chr6:82636622-82636814	12	TCTTAAAAAAAAAAAAA	TCTT	13
80	chr9:48462214-48462400	14	TTTAAAAAAAAAATAA	TTT	12
81	chr1:191553289-191553474	12	TTTAAAAAAAAAAAAA	TTT	13
82	chr2:174321465-174321652	13	TCTTTAAAAAAAAAAAAAA	TCTTT	14
83	chr9:14399569-14399757	12	TCTTTTAAAAAAAAAAAGAAAAA	TCTTTT	16
84	chr1:12272434-12272614	13	TCTTTTAAAAAAA	TCTTTT	7
85	chr2:60163028-60163218	14	TCTTAAAAAAAAAAAAGAAA	TCTT	15
86	chrX:48507467-48507658 Mm1t	12	TCTTTAAAAAAAAAATAAAAAAAATAAAAA	TCTTT	23
87	chr1:132242147-132242338	15	TCTTTAAAAAAAA	TCTTT	8
88	chr16:17919203-17919386	15	TCTTTTTTAAAAAAA	TCTTTTTT	7
89	chr3:95795999-95796191	14	TTAAAAAAAAAAAATAAA	TT	15
90	chr13:18542264-18542450	12	TTAAAAAAAAAGAA	TT	12
91	chr13:17991337-17991525	15	TCTTTTTTTAAAGAAAAAA	TCTTTTTTT	10
92	chr1:136358246-136358434	15	TCTTTAAAAAAAAA	TCTTT	9
93	hr8:70620149-70620336	14	TCTTAAAAAAAAGA	TCTT	10
94	chr11:107306791-107306979	13	TTTAAAAAAAAAAAAAGAAA	TTT	16
95	chr4:152319403-152319590	15	TTAAAAAAAAAGAAA	TT	13
96	chr10:77005875-77006061	15	TCTTTAAAAAAAAAA	TCTTT	9
					Mean length = 15,0

Terminator statistics

Sequences	Number	Fraction
$TC(T)_{>3}$	5	5.2%
TCTTT	50	52.0%
TCTT	24	25.0%
Others	17	17.7%
