# Supplementary material for: Analysis of SINE Families B2, Dip, and Ves with Special Reference to Polyadenylation Signals and Transcription Terminators
Source: Int J Mol Sci. 2021 Sep 13;22(18):9897. doi: 10.3390/ijms22189897 (PMC8466645; doi:10.3390/ijms22189897)
Supplement: Supplementary file 1 [file ijms-22-09897-s001.zip › Supplementary Vassetzky/SINE B2. Figs S1-S6. Tables S1-S5/Table S4.pdf]

Table S4. Random B2a copies in genomes of 16 mouse strains

#	Chromosome: position	Number of mouse strains + rat	Tail with a terminator	Terminator	A-rich tail length, nt
1	chr11:11253175-11253361	16 + rat	TCTTTAAAAAAAAA	TCTTT	9
2	chr3:58979601-58979784	16	TCTTTTAAAAAATTATTTTTAAAAAAA	TCTTTT	6+7
3	chr11:87445563-87445758	16	TCTTAAAAAAAAAAAGAAA	TCTT	15
4	chr2:9900243-9900434	16	TCTTTAAAAAAAAAAAA	TCTTT	12
5	chr11:106473456-106473644	16	TTAAAAAAAAA	TT	9
6	chr4:40832833-40833016	16	TCTTAAAAAAAAAAAACCACAAAAAAACCCAAAAA	TCTT	29
7	chr16:17080102-17080296	16 + rat	TTTTTTTAAAAAAAA	TTTTTTT	8
8	chr12:51201319-51201506	16	TCTAAAAAAAAA	TCT	9
9	chr12:72597777-72597968	16 + rat	TCTTAAAAAAGAAAAAA	TCTT	13
10	chr1:72704299-72704490	16	TCTTAAAAAAAAAAAAAAAAAAAAAAAAAA	TCTT	26
11	chr7:80888245-80888435	16 + rat	TCTTTTTTAAAAAA	TCTTTTTT	6
12	chr7:44653577-44653763	16	TCTTTAAAAAAAATAAAAAAA	TCTTT	16
13	chr1:131919929-131920106	16	TCTTTTTTTTTTTTAA	TCTTTTTTTTTTTT	2
14	chr8:122558573-122558764	16	TCTTTAAGAAAGAAAGAAAGAGAGAGAGAAAGGAAGAAA	TCTTT	34
15	chr2:120987919-120988111	16	TCTTAAAAAAAAA	TCTT	9
16	chr13:63474573-63474762	16	TCTTTAAAAAAAAAAAA	TCTTT	12
17	chr19:29681055-29681242	16	TCTTAAAAAAAAAAAA	TCTT	12
18	chr12:58944138-58944323	16	TCTTTAAAAAAAA	TCTTT	8
19	chr7:12902328-12902516	16	TCTTAAAAAAAGAAAAAGAAA	TCTT	17
20	chr6:29626515-29626698	16	TCTTTAAAAAGAAAGAAAGAAAGAAAGAAAGAAA	TCTTT	29
21	chr10:88850836-88851025	16	TCTTTTAAAAAA	TCTTTT	6
22	chr2:84016247-84016434	16	TCTAAAAAAAAAAAAA	TCT	13
23	chr11:114799834-114800025	16	TCTTAAAAAAAAAAAAAAAAAA	TCTT	18
24	chr4:11565217-11565398	16	TCTTTAAAAAAAA	TCTTT	8
25	chr12:83518163-83518353	16	TTATTTTAAAAAA	TTTT	6
26	chr11:97116609-97116793	16	TTATTTTAAAAAAA	TTTT	7
27	chr3:16280238-16280430	16	TCTTTTTTTTAAAAAAAA	TCTTTTTTTT	8
28	chr9:74161854-74162039	16	TCTTTAAAAAAAAAAAAAGACA	TCTTT	16
29	chr4:132177838-132178025	16	TCTTTAATTTAAAAAAAAA	TCTTT	14 ?
30	chr9:108932108-108932298	16	TATTTTTAAATAAATAAA	TTTTT	11
31	chr19:45969930-45970128	16	TTTCTTTAAAAAAAAAAAGAAA	TCTTT	14
32	chr1:74318336-74318545	16	TCTTTTAAAAAATTTTAAAAAAAGAGAGAGAGAG	TCTTTT	28 ?

33	chrX:135033311-135033498	16	CCTTTTTAAAAAAAA	TTTTT	8
34	chr6:126808958-126809142	16	TCTAAAAAAAAAAA	TCT	11
35	chr1:139443079-139443263	16	TCTTAAA	TCTT	3
36	chr2:131598117-131598299	16	TCTTTAAAAAAAGAAAA	TCTTT	12
37	chr10:4342045-4342228	16	TCTTTAAAAAAAA	TCTTT	8
38	chr4:43997138-43997327	16	TCTTAAAAAAAAAAAAAA	TCTT	14
39	chr1:135353308-135353492	16 + rat	TCTTTAAAAAAAA	TCTTT	8
40	chr1:134740547-134740737	16	TCTTAACAAAAAATAATAA	TCTT	15
41	chr17:42841435-42841623	16	TTAAAAGAAAAAAA	TT	12
42	chr11:5162698-5162891	16	TCTTTTTAAAAAAA	TCTTTTT	7
43	chr2:152921603-152921793	16	TCTTTAAAAAAAAAAAAA	TCTTT	13
44	chr7:79331615-79331797	16	TTTTCTTTTAAAAAAA	TTTTCTTTT	7
45	chr15:5102826-5103034	16	TCTTTAAAAAAA	TCTTT	7
46	chr12:84078508-84078689	16	TCTTTAGAAAAAAAAAGAAAAAGAA	TCTTT	20
47	chr5:107389963-107390149	16	TCTTTAAAAAAAAA	TCTTT	9
48	chr8:111324306-111324495	16 + rat	TCTTTAAAAAAA	TCTTT	7
49	chr4:135995984-135996172	16	TCTTTAAAAAAAAAA	TCTTT	10
50	chr16:10855044-10855232	16	TCTTAAAAAAACAAAA	TCTT	12
51	chr1:59793467-59793649	16	TCTTTAAAAAAAAA	TCTTT	9
52	chr4:116930235-116930417	16	TCTTAAAAAAA	TCTT	7
53	chr11:51758764-51758951	16	TCTTAAAAAAAAAA	TCTT	10
54	chr1:55076708-55076885	16	TCTTAAAAAAAAAA	TCTT	10
55	chr7:46921268-46921458	16	TCTTTTTTAAAAAATAAAAA	TCTTTTTT	12
56	chr4:156211095-156211288	16	TCTTTAAAAAAAAAAAAGAAAGAAA	TCTTT	20
57	chr1:9914899-9915079	16	TTTAAAAAAA	TTT	7
58	chr10:58581064-58581253	16	TCTTAAAAAAAAAAAAAAA	TCTT	15
59	chrX:93479646-93479832	16	TCTTTTTTAAAAAAA	TCTTTTTT	7
60	chr2:71352944-71353129	16	TTTTAAAAA	TTTT	5
61	chrX:102410389-102410581	16	TCTTTTAAAAAAAAAA	TCTTTT	10
62	chr9:40814648-40814839	16	TCTTAAAAAAAAAAA	TCTT	11
63	chr1:86486479-86486671	16	TCTTCAAAAAAAAA	TCTT	9
64	chrX:101367251-101367434	16	TTTTAAAAAA	TTTT	6
65	chr1:63184386-63184576	16	TCTTAAAAAAAAAA	TCTT	10
66	chr1:44054311-44054497	16	TCTTTAAAAAAAA	TCTTT	8
67	chr5:121391506-121391695	16	TCTTCAAAACAAAAAAA	TCTT	13
68	chr2:71104394-71104585	16	TCTTTAAAAAAAA	TCTTT	8

69	chr16:96076681-96076873	16	TCTTTAAAAAAAAAAAAAAAA	TCTTT	16
70	chr4:108733470-108733658	16	TCTTTTTTTAAAAAAA	TCTTTTTTT	7
71	chr18:20859543-20859731	16	TCTTTTAAAAAAAAA	TCTTTT	9
72	chr9:53458176-53458365	16	TCTTTAAAAAAA	TCTTT	7
73	chr6:137038476-137038667	16	TCTTAAAAAAAAAAA	TCTT	11
74	chr11:14117171-14117367	16	TTTAAAAAAAAA	TTT	9
75	chr9:99231994-99232154	16	TCTTTAAAAAA	TCTTT	6
76	chrX:142384174-142384361	16	TCTTTAAAAAA	TCTTT	6
77	chr10:93952407-93952595	16	TTTTACAAAA	TTTT	6
78	chr17:28949098-28949288	16	TCTTCAAAAAAAGAAAAA	TCTT	14
79	chr7:141286621-141286808	16 + rat	TCTTAAAAAAAAAAA	TCTT	11
80	chr11:87963942-87964124	16	TCTTTTTTTTTTTTAAA	TCTTTTTTTTTTTT	3
81	chr11:97266785-97266969	16	TTAAAAAAATAAAAAAAAA	TT	16
82	chr10:67084395-67084581	16 + rat	TTAAAAAAAAAAA	TT	11
83	chr14:52153728-52153928	16 + rat	TCTTAAAAAA	TCTT	6
84	chr13:9021926-9022123	16	TTTTTTAAAAAGGAAAAAA	TTTTTT	13
85	chr5:99776422-99776617	16 + rat	TCTTTAAAAAAAAAAAAAA	TCTTT	14
86	chr19:28660709-28660898	16	TCTTTTTTAAAAA	TCTTTTTT	5
87	chr7:98445838-98446027	16	TTTAAAAAAGAAAAAGAAAAAA	TTT	19
88	chr5:115497460-115497654	16	CCTTTAAAA	TTT	4
89	chr14:55209292-55209483	16	TTTTTTTTTTAAAAAAA	TTTTTTTTTT	7
90	chr13:54476657-54476859	16	TCTTAAAAAAAAAAAAAA	TCTT	14
91	chr1:89983521-89983718	16	TTTTTTTAAAAAAA	TTTTTTT	6
92	chr11:52041315-52041499	16 + rat	TCTTTAAGAAAAAA	TCTTT	8
93	chr1:156060816-156061003	16	TCTTAAAAAAAAATAAA	TCTT	12
94	chr6:100414122-100414307	16	TTCTTTAAAAA	TCTTT	7
95	chr3:113768153-113768345	16	TTTAAAAAAAA	TTT	8
96	chr17:28794966-28795150	16 + rat	CCTTTTAAGAA	TTTT	5
97	chr17:29196508-29196704	16	TCTTTTTTTAAAAAAAA	TCTTTTTTT	8
98	chr14:62928931-62929124	16	TTTAAAAAAAA	TTT	8
99	chr4:124105585-124105773	16	TCTTTAAAAAA	TCTTT	6
100	chr12:86096247-86096466	16	TCTTAAAAAAAAAAAAAAAA	TCTT	16
101	chr10:27573065-27573242	16	TCTTTTTTGAAAAA	TCTTTTTT	6
102	chrX:162704808-162705004	16	TCTTTAAAAAAATAAAAA	TCTTT	12
103	chr6:95815808-95816005	16	TCTTTAAAAAAATAAAATAAAAA	TCTTT	18
104	chr2:25816010-25816199	16	TCTTTTTTAAAAAAA	TCTTTTTT	7

105	chr18:35304838-35305023	16 + rat	TCTTTTAAAAAAA	TCTTTT	7
106	chr15:99009278-99009464	16 + rat	TCTTACAAAAACAAAA	TCTT	12
107	chr10:84552809-84552993	16 + rat	TTTTTAAAAA	TTTTT	5
108	chr15:84109854-84110050	16	TCTTTTTTTAAAAAAAA	TCTTTTTTT	8
109	chr11:80082956-80083146	16	TCTTTAAGAAAAAAAAA	TCTTT	12
110	chr9:110059856-110060037	16	TCTTTAAAAA	TCTTT	5
111	chr9:75487303-75487490	16 + rat	TTTTTTAAAA	TTTTTT	4
					Mean length = 10.5

Terminator statistics

Sequences	Number	Fraction
TC(T)$_{>3}$	34	30.6%
TCTTT	36	32.4%
TCTT	28	25.2%
Others	13	11.7%
