# Supplementary material for: Analysis of SINE Families B2, Dip, and Ves with Special Reference to Polyadenylation Signals and Transcription Terminators
Source: Int J Mol Sci. 2021 Sep 13;22(18):9897. doi: 10.3390/ijms22189897 (PMC8466645; doi:10.3390/ijms22189897)
Supplement: Supplementary file 1 [file ijms-22-09897-s001.zip › Supplementary Vassetzky/SINE Dip. Figs S7-S22. Tables S6-S8/Fig S10.pdf]

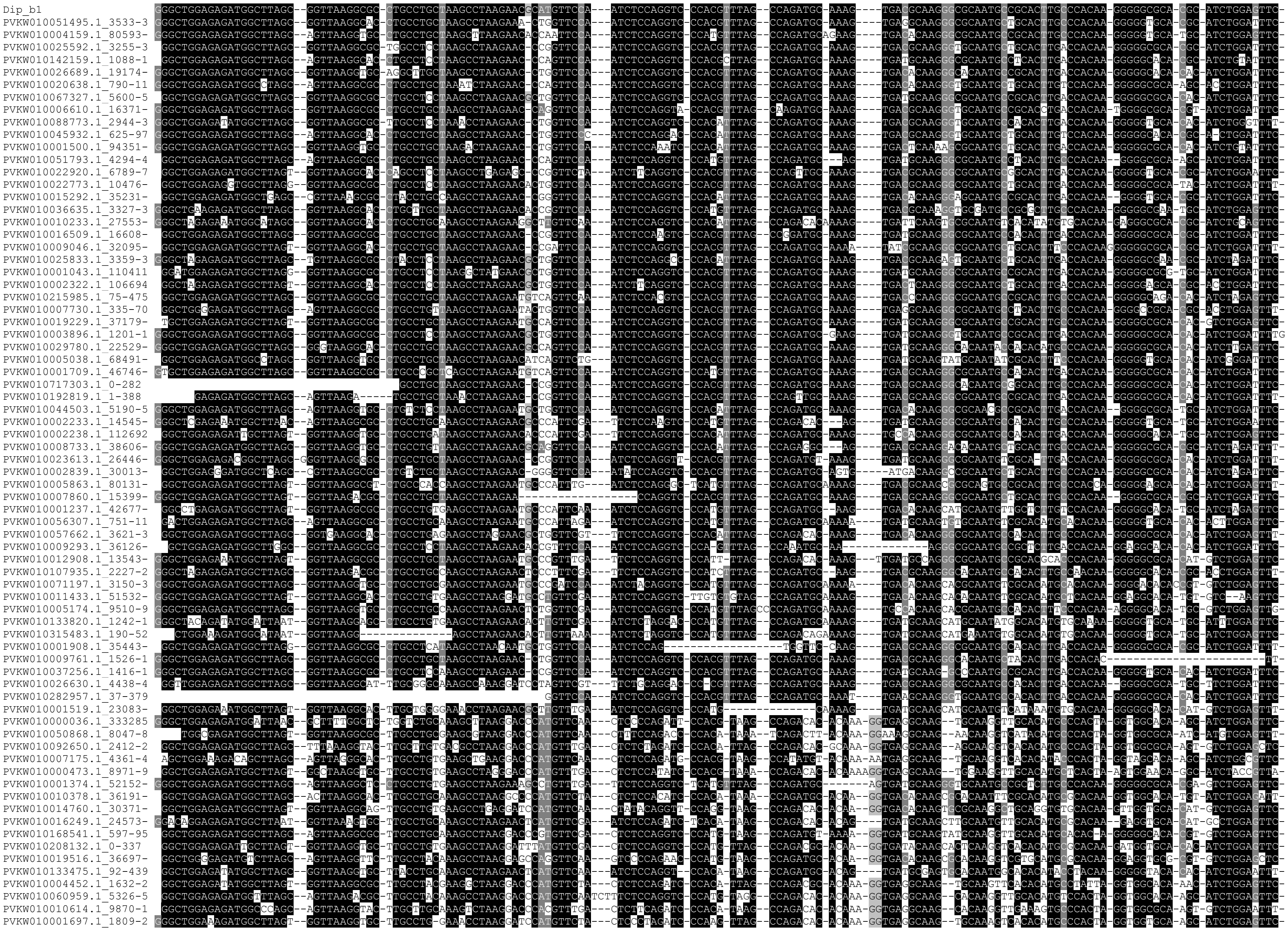
Dip_b1
PVKW010051495.1_3533-3
PVKW010004159.1_80593-
PVKW010025592.1_3255-3
PVKW010142159.1_1088-1
PVKW010026689.1_19174-
PVKW010020638.1_790-11
PVKW010067327.1_5600-5
PVKW010006610.1_16371-
PVKW010088773.1_2944-3
PVKW010045932.1_625-97
PVKW010001500.1_94351-
PVKW010051793.1_4294-4
PVKW010022920.1_6789-7
PVKW010022773.1_10476-
PVKW010015292.1_35231-
PVKW010036635.1_3327-3
PVKW010010233.1_27553-
PVKW010016509.1_16608-
PVKW010009046.1_32095-
PVKW010025833.1_3359-3
PVKW010001043.1_110411
PVKW010002322.1_106694
PVKW010215985.1_75-475
PVKW010007730.1_335-70
PVKW010019229.1_37179-
PVKW010003896.1_1201-1
PVKW010029780.1_22529-
PVKW010005038.1_68491-
PVKW010001709.1_46746-
PVKW010717303.1_0-282
PVKW010192819.1_1-388
PVKW010044503.1_5190-5
PVKW010002233.1_14545-
PVKW010002238.1_112692
PVKW010008733.1_38606-
PVKW010023613.1_26446-
PVKW010002839.1_30013-
PVKW010005863.1_80131-
PVKW010007860.1_15399-
PVKW010001237.1_42677-
PVKW010056307.1_751-11
PVKW010057662.1_3621-3
PVKW010009293.1_36126-
PVKW010012908.1_13543-
PVKW010107935.1_2227-2
PVKW010071197.1_3150-3
PVKW010011433.1_51532-
PVKW010005174.1_9510-9
PVKW010133820.1_1242-1
PVKW010315483.1_190-52
PVKW010001908.1_35443-
PVKW010009761.1_1526-1
PVKW010037256.1_1416-1
PVKW010026630.1_4438-4
PVKW010282957.1_37-379
PVKW010001519.1_23083-
PVKW010000036.1_333285
PVKW010050868.1_8047-8
PVKW010092650.1_2412-2
PVKW010007175.1_4361-4
PVKW010000473.1_8971-9
PVKW010001374.1_52152-
PVKW010010378.1_36191-
PVKW010014760.1_30371-
PVKW010016249.1_24573-
PVKW010168541.1_597-95
PVKW010208132.1_0-337
PVKW010019516.1_36697-
PVKW010133475.1_92-439
PVKW010004452.1_1632-2
PVKW010060959.1_5326-5
PVKW010010614.1_9870-1
PVKW010001697.1_1809-2

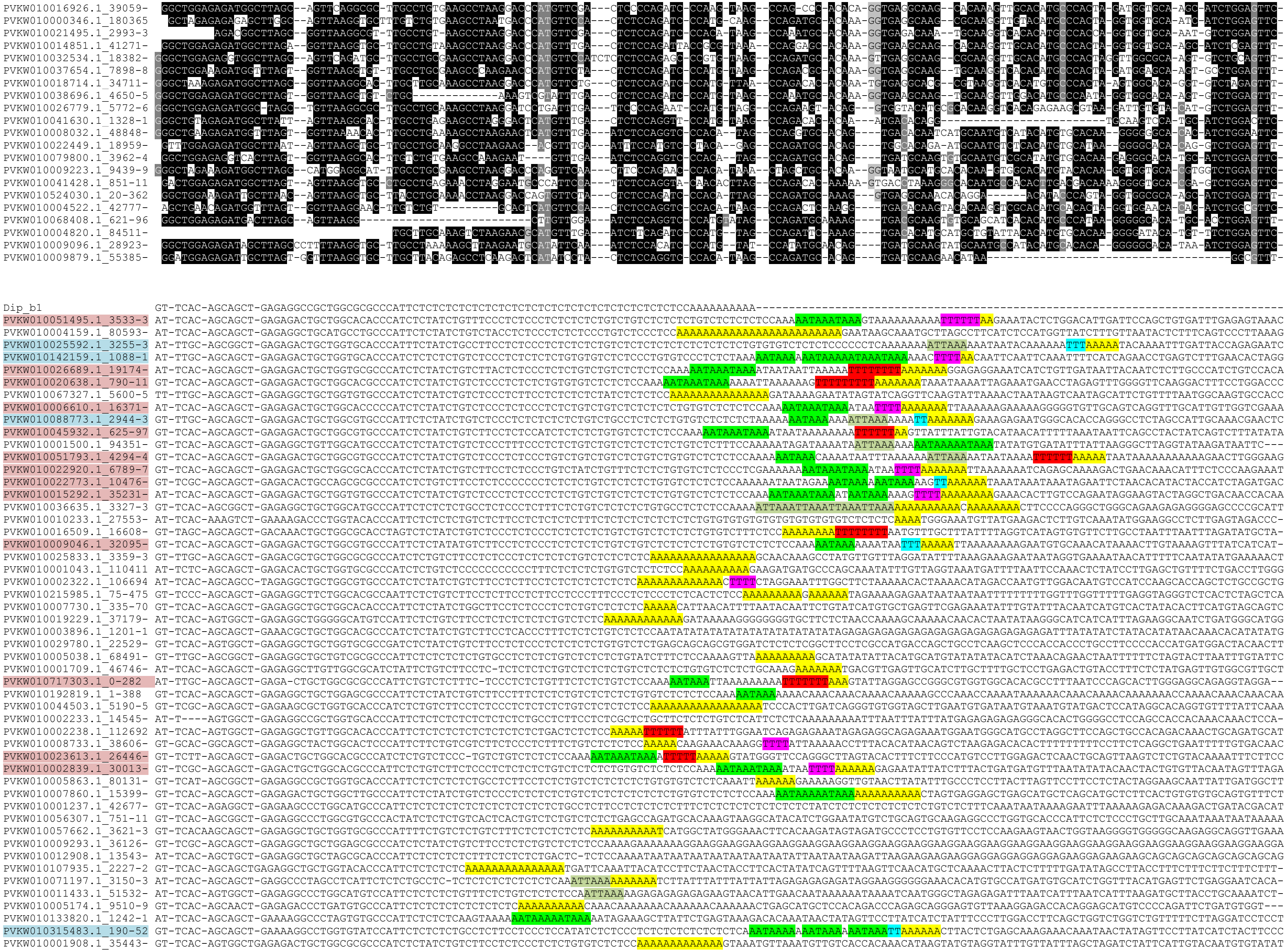

PVKW010016926.1_39059-
PVKW010000346.1_180365
PVKW010021495.1_2993-3
PVKW010014851.1_41271-
PVKW010032534.1_18382-
PVKW010037654.1_7898-8
PVKW010018714.1_34711-
PVKW010038696.1_4650-5
PVKW010026779.1_5772-6
PVKW010041630.1_1328-1
PVKW010008032.1_48848-
PVKW010022449.1_18959-
PVKW010079800.1_3962-4
PVKW010009223.1_9439-9
PVKW010041428.1_851-16
PVKW010524030.1_20-362
PVKW010004522.1_42777-
PVKW010068408.1_621-96
PVKW010004820.1_84511-
PVKW010009096.1_28923-
PVKW010009879.1_55385-
Dip_b1
PVKW010051495.1_3533-3
PVKW010004159.1_80593-
PVKW010025592.1_3255-3
PVKW010142159.1_1088-1
PVKW010026689.1_19174-
PVKW010020638.1_790-11
PVKW010067327.1_5600-5
PVKW010006610.1_16371-
PVKW010088773.1_2944-3
PVKW010045932.1_625-97
PVKW010001500.1_94351-
PVKW010051793.1_4294-4
PVKW010022920.1_6789-7
PVKW010022773.1_10476-
PVKW010015292.1_35231-
PVKW010036635.1_3327-3
PVKW010010233.1_27553-
PVKW010016509.1_16608-
PVKW010009046.1_32095-
PVKW010025833.1_3359-3
PVKW010001043.1_110411
PVKW010002322.1_106694
PVKW010215985.1_75-475
PVKW010007730.1_335-70
PVKW010019229.1_37179-
PVKW010003896.1_1201-1
PVKW010029780.1_22529-
PVKW010005038.1_68491-
PVKW010001709.1_46746-
PVKW010717303.1_0-282
PVKW010192819.1_1-388
PVKW010044503.1_5190-5
PVKW010002233.1_14545-
PVKW010002238.1_112692
PVKW010008733.1_38606-
PVKW010023613.1_26446-
PVKW010002839.1_30013-
PVKW010005863.1_80131-
PVKW010007860.1_15399-
PVKW010001237.1_42677-
PVKW010056307.1_751-11
PVKW010057662.1_3621-3
PVKW010009293.1_36126-
PVKW010012908.1_13543-
PVKW010107935.1_2227-2
PVKW010071197.1_3150-3
PVKW010011433.1_51532-
PVKW010005174.1_9510-9
PVKW010133820.1_1242-1
PVKW010315483.1_190-52
PVKW010001908.1_35443-

```
PVKW010009761.1_1526-1 GA-CCAC-AGCAGCT-GAGAGACTGCTGGCGCGCCCATCTCTATCTGTCTCCCTCTCCCTCTCTGTCTGTCTCTATCTGTGTTTCTCTCCAAAAATAAATAAGATAATTTAAAATTTTTCTAAGAGCAATTTACAGATTCAATGCAATCACCATTAAAATTTCAATGTCAT
PVKW010037256.1_1416-1 GT-TAGC-AGCAGCA-----CCCATCTCTATCTGTCTTCCTCTCCCTCTCTCCAAAAAAAAAAAAAAAAAAAAAAAGGAATGAATATATGCTTAAAGGAAAAAAAAAATAAAGCACATTTAGAAAACATTCCAACGTAACTACACAGTGTTCAAGTTTCATAAGTATGAGG
PVKW010026630.1_4438-4 AT-TCAC-AGCAGCT-GAGAGGCTGCTGGCGCGCCCATCTCTCTCTCTCTCTCTCTCTCTCTCTCTCTCTCTCTCTCTCTCTCTCTCTATATATATATATATATATATATATATATATATATTTTTTTTTTTTTTTTTTTTTTTTAAAAAGAACTTGTCAAGCCAGACCTAAGAG
PVKW010282957.1_37-379 GT-TCGC-AGCAGCT-GAGACACTGGTGGCGCGCCCTTCTCTGTCTTCCTCTCCCTCTCTCTCTCTCTCTCTGTGTCTCTCTCCAAAAAAAAATAATAATAAATAAAAAGTTTTAAAATTAGAACCTTTAATACATATAACCCAAGTCTATTATCACTTTAAAATGTTT
PVKW010001519.1_23083- GT-TCAC-AGGGTCT-GGAAGTCCCTGGTGTTCCCATTCTCTCTCTCTTTCTCTCTCTGTCTCTCCCTCTTCCTCCTTCTTTCTGACACTCTTTCAGAAATAAAATTTTAAAAGACCAAGGTCACTAGACCTTGTAGGCAAGCACCTAACCTCTGAGTCATCTCTCCAG-C
PVKW010000036.1_333285 AA-TTTC-AGTGGGT-GAGGCCCTGGTATGTCTATTCTCTCTCTCTCTGTCTGTCTTTGTCTCTAAAATAAAAAAAAATAAAAACTGGGCGTGGTGGCCCATGCCTTTAATCTTTGCATCTGGTTTTAAGCAAACCCTGGCTAAGGCTTTGCAAGTAAACAATTTTAAG
PVKW010050868.1_8047-8 GA-TTTC-AGTGGCT-GAGGCCCTGGCATACCAATTCTCTCTCTCTCTCTCTCTCCCTCTCTCTTTCTCTAAAATAAAAGTCTTGGCACTTGGGAGGCAGAGGTAGGAGGGTCACTGAGAGTTCAAGGCCACCCTGAGACTACATAGTGAACTATGTAGCTTGAGCTACAG
PVKW010092650.1_2412-2 AA-TTTC-AGTGGCT-GAGG-CCCTGGTGCACCAATTCTCTCTCTCTAAAAATAATAATAATAGCCGGGTGTGGTGGCTCACGCCTTTAATCCCAGCACTTGGGAGGCAGAGGTAGGAGGATCACCATAAGTTTGAGGCCACCCTGAGAATACAGAGTGAATTCCAGGTCA
PVKW010007175.1_4361-4 AA-TTGC-AGTGACT-GAGGCCCTGGTGCATCTAATCTCTTTCACTCTCTCTCTCTCTCTCTCTGTCTCATATAAAAAAAGCAAACATAGAGGCACAAAACAAAAATTTTCTTTTGAAATAATCCTAGTAAGTAGCAACAATTCGATAAATGCATGGGCTTATACTTGG
PVKW010000473.1_8971-9 GA-TTGC-AGTGGCT-GAGACCCTGGCACACCAATTCTCTCTCTCTCACTCTAAAAAAAAAAAAAAAAATCTGCAATATCATTAATTAAAATAACAAACAAAAATAATCTATATTCTTTATTTTCTTCTTTTAAAAATATTTTTATTTTATTTTTATTTATTTATTTTAGA
PVKW010001374.1_52152- GG-TCAC-AGCGGCT-GAGAAGCATGGTTCACCCATTCTTTCTCTCTCTCTCTCAAAAAAAAAAAAAAAAAAAAAAGGACAGTGGAGAGAAACTGAGGGAGACACCCAACATCGATATCTGGCCTCCTACAGACATGTATGCATGCATACCCAACCACGCACATGTATT
PVKW010010378.1_36191- AA-TTGC-ATTGGCTAGAAG-CCATGGTGCACCATCTCTCTCTCTCTCTCTCTCTCTCACACACACACACACACACACACACACACTTGCTCTTTCTCTTTCATAAAAAATACAAAAAAGGAGCCAAGTCATTATAACCTCTTTTACCTTTGAAACTTTTCCATCTA
PVKW010014760.1_30371- AA-TTAC-AATGTCTGGAGGCCCTGGCATGCCAATTCTCTCTCTCTCTCCCTCTCTCTCTTGCTCTCGCTGCCTCTCATAAAAAAAGGGGGGAGGCAGTCTGTTGGGCTTGCCTCAAAAACAGATTTAGTAGATTGATAGTTCTTCCTGATCCTGCAGGATTAAGCAAACA
PVKW010016249.1_24573- AT-TTGC-AGTGGCTGAAGGCCTTGACGTGCCCATTCTGTCTCTCTCAAACAAATTTTTTAAAAGTAACTTTTTAAAAAATGACTGTTTCATGGAAACAATGTAGAAGAGTGATAGAGGATACCCAATGTTCACCTCTGCCCTCTGAACACATGTGCATGGGGAAAGTGCT
PVKW010168541.1_597-95 AATTTAC-AATGGGTAGAGGCCCTGGCGCACCCATTCTCTCTCTCTCTCTCTCTCACTCTTGCATAAAAAAAAATCTATTCTTATCAACAAACTGTCTTCGGGAAAGAGGGCAGAGGTGGCTGGGACATACCTGCAGAGTTGGCAAAGTCAAGATGGTTTGCACTGATCAG
PVKW010208132.1_0-337  GA-CTAC-AATGACTGGAGG-CTCTGGTGTGCCAATTCTCTCTCTGTCTCTCACTCTCTCATAAAATAAAATTAAAAAAAAATTTTTAAATGAAGTTCTAATCTGTGCTATAACACAGATGAACCTTGGAGACATTATGCTAAGAAAAAAGCAAACTTTACCATTTCTT
PVKW010019516.1_36697- AA-TTGC-AGTGGCTGGAGG-CCCTGGCGTGCCAGTTCTCTCTTGCTCTCTCGCAAATAAATAAATAAATAAATAAATAAATAAATAAATATTTGTTTTTAATGGCAGAAGAGAAGGGAAGAAAGAAGACAGAACTCTGGGCAGGACAGAAGAGAAATCTCATTTCCAT
PVKW010133475.1_92-439 GT-TTGC-AGCAGCTGAAGG-CCCTGGCATGACCATTCTCTCTCTCTCTCAAAATAAATAAAATGAACACCTATATGCACAACACATGCAAAGATTTTAGATAATATCTAGATACAGGCATATGCATGTATGCACGCAGCATACACACACGTGTCTGTCCATTTATCTAAC
PVKW010004452.1_1632-2 AA-ATGC-AGTGGCT-GAGG-CTCTGGCGCACCAATTCTCTATCTCTCCTCTCTGTCTCTCTCACTAACTCCCTTTTTAAAACAAAAGCCAAGAACATAGGGCTGAGCTCCCCACTCAGGTCAAGATTACAGCCTAAGCCATGACTTCTGAGAACTTTCCCATCTCAAGG-
PVKW010060959.1_5326-5 AA-TTGC-AGTGGCT-GAGGTCCTGGTGCATCAATCCTCTGTCTGTCTGTCTCTCTTAGCTCTCTATGTCTTTTGCTTTCTCTAAAAAAAAAAAAGTTCTTAGGGATGTGCTAGAGAAATGGGTCAGCAATAAGTACTTCCTGCACAAGCACAAGGGCCTCAGGGGCCTGA
PVKW010010614.1_9870-1 GA-TTGT-AGTAGTT-GAGGCCCTGGCATGCCAATTTTCTCCCCCCCCCCTCTAAAATAAAAAAATAAAGAAAGAGTCTAATTCTTTTTGGTGAGCTTTATCTCCTCTAGAGAACATTCTATGGCACTTCATAAGATAGTGAAGAGGAAAATGGAGCTGTAGATTAGACC
PVKW010001697.1_1809-2 AA-TTGC-AGTGGCT-GAGG-CCCTGGCATGTCAATTCTCTCTCTCTCTGTCTCTCTCACTCTTTCTCTAAAAGTAGAAAACAATTTTTTCTTTACCTATGAGAGAGAGGCAGAGAGAGAGAGACAGAGAATAGGTGTGTCAGAGCCTCTTGCCACTGGAAATGAACTT
PVKW010016926.1_39059- AA-TTGC-AATGACC-GAGGCCCTGGCTTCTCTATCTCTTTCCCACCCCCTCTCTAAAATAAAAAATAAAAATAAAAATTAAGAAAAAAAGAACACCATGGCTGGCAGAAGCCCCCAGGCTCTGGTCTATGTGGAAGACATTTCCAAGTGACCAGATAGTAGGGCTTGGCC
PVKW010000346.1_180365 AA-TTGC-AGTGGCT-GAGACCCTAGTATGACAATTCTTTCTCCCCACATCCTCTCACTCGCTTGCTCTTGCTGGCTCTAAAAATAAATAAATACATAAATAAGTAACCATCAAATTTCCATTACCAATGTTGGAAGAAATACAGATTTTTAAAAAAATGTTTACCTAAT-
PVKW010021495.1_2993-3 AA-TTGC-AGTGGCT-GAGGCCCTGCTGTGCCAATTCTCTCTCTCCTCCTCCTCCCTCTCTTCTCCCCTCCCTTTCTTGTTGTCTCAAAAAAAAAAAAAATTTAAAAACCATTGCCCATTAACCTTGCAAAACCGACACTGTGTCAAGAGTAGAGTTATCAGTGGGTGTTT
PVKW010014851.1_41271- GA-TTTC-AGTGGAT-GAG-CCCTGGCACACCAATTCTCTCTCATGCTCTCTCTCTCTAAAATAAAATAAAAATCCTAAGCAGAGTCACACAGTAACAGAAATGCCAAGTCCATATCTTTCCTTTGAGATGCTCATGAATTAAAAACACACATTTCTCTATATTTAGAACA
PVKW010032534.1_18382- TA-TTGC-AGTGGCT-GAGA-CCCTGGCATGCCAA-TTCTCTCTCTCTCTCTCTCTTACACTCTCTCTAAAGCAAACAAACAAACAAAAAAAAAACCACCTCAAACAATAAAATAAAAATAACACAAAATTACACCAACTCTCAGAGCTTTCAAAGGACTCAGAGTTTTAG
PVKW010037654.1_7898-8 GA-TTTC-AGTAGCT-GGGACTGGCACACCAAATTCTCTCACTCTTGCTCTCTCTAAAATAAAATAAATAAAAAAAAGAAAAAAGATGTGGAGGAAAATATGGAGATAGACTGAAGTCTGATACTGAAGGTTGGGTTTCTATGACAGTACAGAATGCTCACTCATCTGCAG
PVKW010018714.1_34711- GA-TTTC-AGTGGCT-GAGAATTCTCTATTTCTCCTCTCTCTGTTCCTTTCTCTCTCTCTCTCTCACTCGCTCACTCTCTAAAAAATAAAAAAAAAATTAAAAGGCTACAATATAATGTTGAAGTGTTTTCATAAGAGTTAATTAAAATTTTACTGTAACTTGTTCTAA
PVKW010038696.1_4650-5 GA-GTGC-AGTGGCT-GAGACCCTGGTGTGCCAATTCTCTCTCTCTCTCTCTCTCTCTCTCTCTCTCTCTAAAAGAAGAAGGAAGGAAAGAAATGGTTTATGGAAGGAAATGGTTTATTTTTGGCTTACATTTGCAAGACGAAGTCTATCCTGGTGGGGAAAATATGGC
PVKW010026779.1_5772-6 GA-TTGC-AGTGGCTAGAAGCCCTGGCATGCCAATTCTCTCTCTCTCATAAATTAAATAAATAAATAAATAAATAAATAAATAAATAAATAAATAAAACATATATATTAAAATTGTCAATAAAAATTGAGAAAAATTATGAAAGAAAACTAATTTTTTTTAAAGTAA
PVKW010041630.1_1328-1 --------AGTAGCTAGAGG-CCCTGGCATGCCAA-TTCTCTTTCTCTTTCACTCTTGCATAAAAAAAGGCCAGTCTGTTGGGCTTGCCTCAAAAGTAAATTAATAAATGGGAGAAGGGAAAGTATTTCCTTTAAAAGCAGGAATGAGACAAGGTGTCAACTATTGGACGG
PVKW010008032.1_48848- AC-TCAC-AGCAGCC-CTGGAAACCCATTCTCTCTCTGTGTCTCCCTCTGTCTCTCCCTCTCTCTCTCAAAAATAAAAAAAAAATGAGACAAAGGAGAAAGATTATCGAAGAGAACAGTAAGTTAGTTCACCATTGTTTTTAGGATTTCTGGAGGGGAGGGTGGATTT
PVKW010022449.1_18959- GT-TCCC-AATGGCT-GAAAGGCCCTGCTGCACCCATTCTCTATCTCTC-TCTGCCTCTTTCTCTCTCTCTATCTCTCAAAAAATGAATAATAATAATGATAAAAGAAAACACAAAATAAAATTGCAGAGTAAAAATTTCTTCCAATTTCATTGTCACAATCAGGTATATT
PVKW010079800.1_3962-4 GT-GCAC-AGCATCT-GAAAGGCCTTGGCATACCCATTCTCTCTCTCTCTCCTTCTCAAAAGTAAAATAAAAAATTTTATTTCAAGAGTGAGAGAGATGACTCAGTGGTTAAAAATACTTACTGCATAAGCATGAGGGCCTGAGACTGCCAAGTTCAAATCCCCAGAAC
PVKW010009223.1_9439-9 AT-TTGC-AGTTGTT-AAGGCCTTGGTGTACCAA-TTTTCTCTCTCCTCTCTCTTGCTCTTTCTTTTATAAAAAAAAAAATGAGCAAGTGATTGAGTGTTGCCCATCTTAATTAAGGTTTGAGCATTCTTCAGTCAGATGATTAAGTAGCCAGGAAACACAGAGGCAAGGG
PVKW010041428.1_851-11 AT-TCAC-AATGGCT-GAGAATGACGTACCCATTCTCTATTTGTCTGTCTCTCTCTTTCTCTGTCTCTCTCAAAAAAAAATCGCAATGAGATAACACCTCATTCTACTTAGGTAGGCTATTGTTTAAAAAAACAATTAGCAATGCTGAAGAGGATGTACAGAAAAGAT---
PVKW010524030.1_20-362 A-------AGTGTCT-GAGGCTGCCCACAGCCAATTCTCTCCCTCTCTCTCTGTCTCTCACTCTCTAAAAAAAAAATTACTGTTTATGACATTGAATTTAAACACAGAAAAGTAATCTGCTGTAATAAACTGATAGCTTTGATACAGATATGATTGTATCATCCACTTCTA
PVKW010004522.1_42777- CA-GTAT-AGTAGCCACAGGCCCTGGTATGCCAATATTCTCATTCTTTCTCTTTATCACTCTGTCTTTCTCACTTTTGCTCCCACTCTCGCTCTCTCATATAAAATAAAGGCCAGTCTGTTGGGCTTGCTTCGAAAATAATTGTCTAAAATAGTTGTTGCTATTACGAGGC
PVKW010068408.1_621-96 GT-CCAC-AGTGGCT-GAAAGGCCCTGGCTCACCCATTCTCTGCCTCTTCCTCTCTCTGTCTCTCTCTCTCAAATTTAAAAAAAAAAAATTGCTGTTCTTATGTTGACCATCTCATGCTTTTCTGTGTTCCAGGCTGCCCTGCAACCCCTGTGCCACCACCAATGCCTGTG
PVKW010004820.1_84511- AT-TCAC-AGTGGCT-GAAAAGACCCTGGCATGCCCATTCTATCTCTCTCTCTCTCTTTCTCTTTCTGCTTCTTTCTGTCTCTCAAAAATAAAATAAAATATTAAGCATTCAGGATATGATGGAGTATGGAGTTGTGAAGAAGAAAGTAAGAGAACATCTGAGAAATGT
PVKW010009096.1_28923- TT-TCAC-AG-AGCTGAAAGGCCCCACTGTGTCCACTCTGTGTATGTGTGTGTGTGTGTGTGTGTGTTTGTCTGTCTGTCTCTCAAAAATAAATAAAGATCATAAATAATAATAATAGTAATAAACCTCTTGACCTTCTCCCTGCATTGCATTTAAAGCTACCCACTACTG
PVKW010009879.1_55385- GC-CTGA-GAAGCCT-AAGGACCCTGGTGCACCAATTCTTTCTCTCTATCTCTCTCTATCTAAAAAAATAAAATTTTTTTAAAAAGAAAAAAAGGAAAGAAATTCTGACATGTCGTACAGCACAGGTGAACCTTGGAGGACATCATGCTATGTGTTTACAGATGGTAAAC
```

	Copy number	Proportion
T^+ copies	20	21%
Conventional T^+ copies	7	8%
T^- copies	67	71%
Total	94	

Figure S10. Random Dip_b1 copies from the *A. bullata* genome. Nucleotide sequences are highlighted as follows: PASs, strong pol III terminators, medium terminators, rudimentary terminators, and oligo(A). Conventional T^+ copies do not contain either a terminator or an oligo(A).
