# Supplementary material for: Analysis of SINE Families B2, Dip, and Ves with Special Reference to Polyadenylation Signals and Transcription Terminators
Source: Int J Mol Sci. 2021 Sep 13;22(18):9897. doi: 10.3390/ijms22189897 (PMC8466645; doi:10.3390/ijms22189897)
Supplement: Supplementary file 1 [file ijms-22-09897-s001.zip › Supplementary Vassetzky/SINE Dip. Figs S7-S22. Tables S6-S8/Fig S11.pdf]

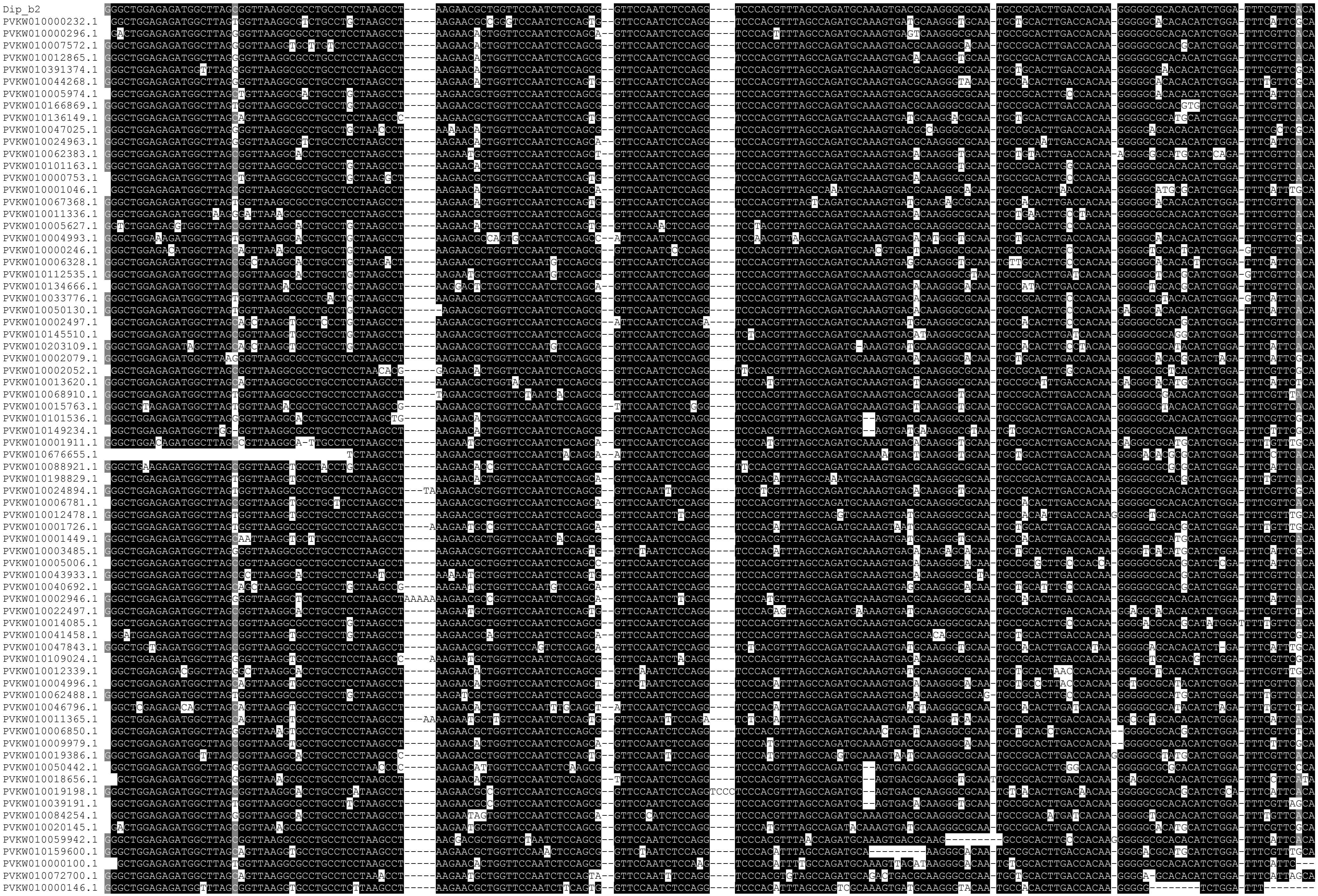
Dip_b2
PVKW010000232.1
PVKW010000296.1
PVKW010007572.1
PVKW010012865.1
PVKW010391374.1
PVKW010044268.1
PVKW010005974.1
PVKW010166869.1
PVKW010136149.1
PVKW010047025.1
PVKW010024963.1
PVKW010062383.1
PVKW010101163.1
PVKW010000753.1
PVKW010001046.1
PVKW010067368.1
PVKW010011336.1
PVKW010005627.1
PVKW010004993.1
PVKW010000246.1
PVKW010006328.1
PVKW010112535.1
PVKW010134666.1
PVKW010033776.1
PVKW010050130.1
PVKW010002497.1
PVKW010145510.1
PVKW010203109.1
PVKW010002079.1
PVKW010002052.1
PVKW010013620.1
PVKW010068910.1
PVKW010015763.1
PVKW010101536.1
PVKW010149234.1
PVKW010001911.1
PVKW010676655.1
PVKW010088921.1
PVKW010198829.1
PVKW010024894.1
PVKW010006781.1
PVKW010012478.1
PVKW010001726.1
PVKW010001449.1
PVKW010003485.1
PVKW010005006.1
PVKW010043933.1
PVKW010040692.1
PVKW010002946.1
PVKW010022497.1
PVKW010014085.1
PVKW010041458.1
PVKW010047843.1
PVKW010109024.1
PVKW010012339.1
PVKW010004996.1
PVKW010062488.1
PVKW010046796.1
PVKW010011365.1
PVKW010006850.1
PVKW010009979.1
PVKW010019386.1
PVKW010050442.1
PVKW010018656.1
PVKW010019198.1
PVKW010039191.1
PVKW010084254.1
PVKW010020145.1
PVKW010059942.1
PVKW010159600.1
PVKW010000100.1
PVKW010072700.1
PVKW010000146.1

```
                  GCAGCTGAGAGACTGCTGGCGC GCCCA TCTCTgTCTgtCTccCTCTCtCTCTCTCTCTcTCTCTCTCTcTctctctataaAaAAAaaaAaAaAAaaAAaaaaaaaaaaAaaaaaaaaaaaaaawaaaaagtaaaaaaa aattaaaaawraaaaaaattaat ga awt a ataaa   t  aa
Dip_b2            GCAGCTGAGAGACTGCTGGCGC-GCCCA-TCTCTGTCTGTCTCCCTCTCTCTCTCTCTCTCTCTCTCTCTGTCTCTCTCTCAAAAAAAAAA-----------------------------------------------------------------------------------------------
PVKW010000232.1   GCAGCTGAAAGGCTGCTGGGGC-ACCCA-TCTCTATCTGTCTTCCTCTCCCTCTCTCTGTCTCTCTCTCTGTGTCTCTCTCCAAAAAATAATAATAATAATAAAAATAAACACAAAAAACAGCCATGCTTGGTACACAATAGCAATTCAGGCATATTACCTTTGTGGGTAGGACTTAACCCTTTAA
PVKW010000296.1   GCAGCTGAGACACTGTTGGTGC-ACCCA-TTTCTGTCTTCCTCTCCCTCTCCCTCTCCCTCTCTCTCTCTCTCTCCCTCTCCCTGTGTGTCTCTCCCCCAAAAAATAATAAAAAAATAAGAGTACATTGTGAAAGCCTGTAGTAGATAAGAGTGAATGAAAAAGAGTCATGAGATGCAGTCAGAA
PVKW010007572.1   GCAGCTGAGACACTGCTGCCGC-GCCCA-TCTCTGTCTCCCTCTCCCTCTCCCTCTCTCTCTCTCTCTTTCTGTCTCTCTCTCTCTGTCTCTCCAAAAAATAATAAAAAGTAAAAAGTTTAAAAAAATAAAAAGAAAAGAAAACAACATGAGATGCTGCTCTTAGGTCTTGGCCAGAACTGTA
PVKW010012865.1   GCAGCTGAGACACTGCTGGTGC-GCCCA-TCTCTGTCTCCCCCCCCCCACCCCCGTCTCTCTGTCTCTCTCTCTCTCTATGTCTCTCTCCAAAAAAATAACAACAACAAAAAAAAAAACTGCATTATGTTGGGGAAACTTGTAAGAAAGTGGTAAAAGAAACAATGAGCTCCCCTCCCCCAATTTG
PVKW010391374.1   GCAGCTGAGACACTGCTAGCGC-GCCCA-TCTCTGTCTTCCTCTCCCCCTCTCTCTCTCTCTGTCTCTCTCTCTGTGTCTCTCTCCAAAAAAATAATAGAAAATAAAAAATAAAAAAGTTTAAAAAATAGGGCTGGAGAGATGGCTTAGCGGTTAAACGCTTGCCTATGGAGCCT
PVKW010044268.1   GCAGCTGAGACACTGCTGGCGC-GCCCA-TCTCTGTCTTCCCATTGCTCGCTCTCTCTCTCTCTCTGTCTCTGTCTCTCTCTGTGTGTGTGTCTCTCTCCAAAAAAAAAAAAAAGAGTTAAATTTCCCATATCTAAGAACATTGTGACTTTAAAGATAGCTCATGCTTTATTTATGCTAATTCT
PVKW010005974.1   GCAGCTGAGAGACTGCTGGCAC-GCCCA-TCTCTATCTGTCTCCCTCTCCCTCTATGTCTGTCTGTCTCTCTCTCTGTGTGTCTCTCCAAAAATAAATAATAAAAATTTTAAAAAAGAATCTCAGAACCTCCTCCTCTTTCTACCTCCCCTTTCCCCTTCCCAACCAGCCGTAATTGAGACCTCAA
PVKW010166869.1   GCAGCTGAGAGACTGCTGGCGC-ACCCA-TCTCTATCTGTCTTCCTCTCCCTCTCTCTCTGTCTCTCTCTCTGTCTCCCTCCAAAAAAAAATGATTTTGACAATGATCTTATGATTAGGATTATTATACATTGATTACTTTAGAATTAGGATCAGGGCTGGAGAGATGGCTTAGCGATTAAGGCTC
PVKW010136149.1   GCAGCTGAGACACTGCTGGCGC-ACCCA-TCTCTGTCTTCCTCTCTCTCTCTCTCTGTCTCTCTCTGTGTGTCTCTCTCCAAAAAAAAAAATAAAAAAAAAAAGAAAGGTAGTCCGAGACCACTGTGGTGGTGACCCCGGTGGGGGCAAGAGTTCCCGACAGGAGAGCGTGACTGGGAGGGCGTGC
PVKW010047025.1   GCAGCTGAGACACTGCTCTCGT-GCCCA-TCTCTGTCTTCCCATTACTCTCTCTCTCTCTCTGTCTCTCTTTCTGTCTCTCTCTCTCTGTCTCTCTCCAAGAAAATAATAAAAATAAAAAAGTTTAAAATGTCCAAGAAGCTCTGACAATTCCTGGCTCTGAAGGGAATGTATGGAATCTCTGGGC
PVKW010024963.1   GCAGCTGAGACACTGCTGGCGC-GCCCA-TCTCTGTCTTCCTCTCCCTCTCTGTCTCTCTCTGTGTCTCTCTCCAAAAAAAAATTTTTTTAATTTAAAAAATATATATATATGTATGTCACCAACACTCATGAGGCTAGAGCAAGAGGATTGAAAGTTCCAGGCCAGCCTGCACTACAGAGAGAA
PVKW010062383.1   GCAGCTGAGAGGCAGCTGGCGT-GCCCT-TCTCTATCTGTCTTCCTCTCCCTCTCTCTGTTCTCTGTTTCTCTTTGTGTATCTCTCCAAAAAAAAAAAAAAAAAAAAAAAAAAAAAAAAAAAAAAAA
PVKW010101163.1   GCAGCTGAGAGACTGCTGGCAC-GCCCA-TCTCTGTCTCCCTCTCCCTCTCTGTCTATCTCTCTCTGTGTCTCTCTCCAGAAATAAATAAAATAAAATAAAAATTTAAAGGAGATATGAGGAGAGGTTTGGAGGTGAAGCAGGGTCAGTCAGCATTGCTGCCTCAGAGCTCCTGGGCCTGTGAGGT
PVKW010000753.1   GCAGCTGAGAGACTGCTGGTGC-ACCCA-TCTCTATCCGTCTTCCTCTCCCTCCCTCTCTGTCTGTCTCTCTCTCTCTCTGTGTCTCTCTCCAAAAATAAATAATAAAAATAATTTAAAAAAATTTAAAGGTCTGGGTGACTAATTGTAGAAAAGGTTAGGCTAATGAAAATGGACTTCAGATGGA
PVKW010001046.1   GCAGCTGAGAGGCTGCTGGAGC-GCCTA-TCTCTGTCTGTCTTCCTCTCCCTCTCTCTGTCTCTCTCTGTGTCTCTCTCCAAAAAAAAAAAAAAAAAAAAAAGTATTTGAGGGGCTGGGGAGTTGGCTCACCATTTAAAGGTTTTATTTGCAAAGCCCATGGACCCAACATGGATCCAAGATAGA
PVKW010067368.1   GCAGCTGAGAGACTGCTGGCAC-GCCCA-TCTCTATCTGTCTCCCTCTCGCTCTCTGTCTCTCTCTGTGTTTCTCTCCAAAAATAAATAAAAATAATAAGAAAATTAAAAAAAAACTATGAAAGCAGATTAGGACATTATTAGAAATTTTTGACAGACTCAGTATTGAATACTTCTAAATCTATA
PVKW010011336.1   GCAGCTGAGAGACTGCTGGCGC-ACCCA-TCTCTGTCTTCCTCTCCCTCTCTCTCTCTTTGTGTCTCTCTCCAAAAATAAAAAAATAAATAAATAAAAAATAAAAAGAAAAAAATATACGTCTGGAGAGATGGTTTAGTGGTTAAGGCGCCTTCCTGACAAGCCTAAGAACACCAGTTCGATCT
PVKW010005627.1   GCAGCTGAGAGACTGCTGGCGC-GCCCA-TCTCTGTCTCCCTCTCCCTCTCTGTCTGTCTCTGTGTCTCTCTCCAAAAATAAATAAAAAATAATAAAAAATTTTTTAAAAAGTATGCATAGTCTTTAACAGTCCCAATACTGTTTAAAAGTCCAAAGTATCTTCTGACATGCAAGACAGTCTCTTT
PVKW010004993.1   GCAGCTGAGAGGCTGCTGGCAC-GCCCA-TCTCTATCTGTCTTCCTCTCCCTCTCTCTGTCTCTCTCTGTGTCTCTCTCCAAAATAAATAAATAAATAAATAAATAAATAAATAAATAAATAAATAAATAAATAAGAAAGAAAGAAAGAAAGAAAGAAAGAAAGAAAGAAAGAGAAAAATGTAAAAATTTATCT
PVKW010000246.1   GCAGCTGAGAGACTGCTGGCGC-GCCCA-TCTCTGTCTCCCTCTCCCTCTCTGTCTATCTCTCTCCGTGTCTCTCTCCAAAAATAAATAAAATAATAAAAAATTTTTAAAAAAGAATATACATAAATAAAATTAAGAAGGCTCTAATAACTCACAGGGATTATGGTAGAGGACATAAAAGGTAAGG
PVKW010006328.1   GCAGCTGAGAGACTGCTGGCAC-GCCCA-TCTCTGTCTCCCCCTCCCTCTCTGTCTATCCCTCTCTGTCTCTCTCTCCAAAAAAATAAAAAAATAATAAAAAAATTTTTTTGAAGAGTGGAGTTTCTAAGGGAAAAATGGGGGGAGGGAGTGGATTACCATGGACTATTGTCTATAATTATGGAAG
PVKW010112535.1   GCAGCTGAGAGACTGCTGGCAT-GCCTA-TCTCTGTCTCCCTCTCCCTCTCTGTCTGTCTGTCTCTCTCTGTGTCTCTCTCCAAAAATAAATAAAATAATTTAAAAAAAATTAAAAAAAAACACAAAACTGAGGCATTGTTATTTCTTTGTCTCCATGCAGATCCGTCCTCAGAAATGCCCAAG
PVKW010134666.1   GCAACTGAGAGGCTGCTAGCGC-ACCCA-TCTCTGTCTCCCTCTCCCTCTCTGTCTGTCTCTCTATGTCTCTCTCCAAAAATAAATAAAATAAATAAATAAAAATTTTTTTTAAAAGAATAATTTGAACAGCTACCTTGCCTACCACATGCTTCAGAGACTTGAGTAATCAAAATTTCCCCTCCA
PVKW010033776.1   GCAGCTGAGAGACTGCTGGTGC-ACCCA-TCTCTGTCTCCCTCTCCCTCTGTCTGTCTTTCTGTGTCTCTCTCCAAAAATAAATAAAAAAATTTTAAAAATGTTATGTTAACTATAAAGAGGTATCTCATCCCCTTTAATTTTATGGGCTGCCATCAGGGTGGCATGCTAAGTTTTTGGGTATGAT
PVKW010050130.1   GCAGCTGAGAGACTGCTGGCGC-GCCCA-TCTCTGTCTTCCTCTCCCTCTCTATCTCTCTCTGTGTCTCTCTCCAAAAAAAATAAATAAAATAAATTTTAAAAATTAAAAAAAAAAAAAGAACTGGGTAACACCAGAGGTTAATGGTAAGACCCTACTGCTAAAGGCACCATACACTGTCAGCAC
PVKW010002497.1   GCAGCTGAGAGACTGCTGGCGC-ACCCA-TCTCTGTCTTCCTCTCCCTCTATCTCTCTCTGTGTCTCTCTCCAAAAATAAAATAATTTTTAAAAACTTTAAAAAAATAAATTTAAAAATAGAAACATGCTTAACTTTTTGCATCTAGAATATTAGATGAAAGGAAGTTGTTATTAATATCTATT
PVKW010145510.1   GCAGCTGAGAGGCTGCTGGCGT-GCCCA-TCTCTGCCTTCCTCTCCCTCTCTATCTCTCTGTGTCTCTCTCCAAAAATAAAATACTTTAAAAAAATTTAAAAAAATAAAATAAAATAAAATAAAATACAGACAAACAATGGATAATATAATTCCAATATTTTATTATTTAGTAGTTTCTAAAAA
PVKW010203109.1   GCAGCTGAGAGACTGCTGGTGC-GCCCA-TCTTTGTCTCCCTCTCCCTCTCTGTCTATCTCTGTGTCTCTCTCTCCAAAATAAATAATAAAAATAATTTTAAAAATTTTTAAAAAGAAAGTGAATACAAGGTGGTACATCCAATTGAGGGGAAGAAAACTAAATCATGTGAGGAGATAACAGGGAT
PVKW010002079.1   ACAGCTGAGACACTGCTGGCGC-GCCCA-CTTCTGTCTTCCTCTCTCTCTCTGTGTCTCTCTCCAAAATAAATAATAAATAAATAAATAAATAAATAAATAAATAAATAAATAAATAAATAAATAAATTTTCTACAACAAATACATATTACAAACTAATGATAAGCATATAAAATAATAATCAGGTAAAAGG
PVKW010002052.1   GCAGCTGAGACACTGCTCTCGC-GCCCA-TCTCTGTCTTCCTCTCCCTCTCCCTCCCTCTCTCTCTCTCTCTCTTTCTCTCTCTCTCTCTCCAAAAAATAAGATAAAATAAAAAGTTTAAAAAAAAAAAAAAAAAAAAGGAAAAGACCAGGACGTGATGGAGTCTTAGCTGAATTCTACCA
PVKW010013620.1   GCAGCTGAGACACTGCT-GCGT-GCCCA-TCTCTGTCTTCCCCTCTCCCTCTCTCTCCCTCTCTCTCTTTGTGTCTCTCTCCAAAACTAACTAAATAAATAAAATAAAATAAAATAAAATAAAATAAAATAAAATAAAATAAAATAAAATAAATGCCACAAGTAGAATAAGTTTGTGTGTGAGAGT
PVKW010068910.1   GCAGCTGAGAGAATGCTGGCGC-GCCCA-TCTCGGTCTCCCTGTCCCTCTCTGTCTCTGTGTCTCTCTCCAAAATAAATAAAATAATTTTTAAAAAAATTAAGAAAAGAATGTAGAGCAAGAAACCCTCACTCAAATACACTGAACAGTCCAGAATGACCAGAAACTTATTTTCTGACCTTCCAT
PVKW010015763.1   GCAGTTGAGAGACGGCTGGCGC-GCCCA-TCTTTATCTCTCTCTCTCTATCTGTCTTCCTCTCTCTCTGTCTCTGTCTCGCTCCAAAAAATAAAAAATAAAATAAGAAAGAAATATCCTGAAGTACAACCCAGACTGAGGCCCTAATTATCACACATGAACACCAATAACCATACTAAGAAAGACC
PVKW010101536.1   GCAGCTGAGACAGTGCTGGTGC-GCACA-TCTCTGTCTTCCCCCCTCTCTCTCTCTGTCTCTCACTCTGTGTCTCTCTCCAAAAAATAATAAAAATAAAAAGTTTAAAAAAATGAAAAAGAGAAATGAATTGTGGCTATCTGTTCCCTAGTTATAACTTTATTTATTTCACAAAGTGTTA
PVKW010149234.1   GCCAGTGAGACACTGCTGGCAC-GCCCA-TCTCTGTCTTCCTCTCCCTCTCTCTCTCTCCCCCCCTCCCCCGTCTCTCTCCGTGTCTCTCTCCAAAAAATAATAATAAAAAATAAAAAGTTTAAAAAAAAAATAAGGTGGAAAGTGAGTGAGTGAAGAAGACACTTGATGTTGCCCTCTAGCCTCCAC
PVKW010001911.1   GCAGCTGAGAGGCTGCTGGCGC-GCCCT-TCCCTATCTGTCTTCCTCTCCCTTTCTCTCTGTCTCTCTCTGTGTCTCTCTCCAAATAAAAACAAAATGCTATAGAATATCAGATAAAAACTGGGCTGTGTTGTTGGAGCCCAAGTTGGGTGATATGCTTTCTTGCTTGGGCATTTCCATGTTATT
PVKW010676655.1   GCAGCTGAGATGCTGCTGGCAC-GCCCA-TCTCTATCTGTCTTCCTCTCCCTCTCTCTCTGTTTCTCTCTCTGTGTCTCTCTCCAAAAATAAAAAGCAAAAGCAGAGACTTATGTGTTGTTCCTTGGGAATTTTCCCCCTCCTTAAATCAGAGTAC------------------------------
PVKW010088921.1   GTGGCTGAGAGGCTGCTGGCGC-GCCCA-TCTCTATCTGTCTTCCTCTCCCTCTCTCTCTGTCTCTCTCAAAAAAAAAAAAAAAGGGCCTAAGACCCAAGTTCAATTCTCTAGGACCCATCCTATAAAGGCAGATGCAATCAAGTGCTGGGATTACAGGCCTGAGCCACCTCAATCTGCTTCAA
PVKW010198829.1   GCAGCTGAGAGGCTGCTGGCGC-GCCCA-TCTCTATCTGTCTTCCTCTCCCTCTCTCTCTGTCTCTCTCCAAAAGAAAAAAAATCAAAGCCAAGATCTTGTTGTTTAGTCCTTTTAATTCAGAGTATTTTTAAGAACTACCATGCACTTAGATTCCAGTTCAATGACTAGAACTGCCACAGCCCTA
PVKW010024894.1   GCAGCTGAGAGGCTGCTGGCAT-GCCCA-TCTCTATCTGTCTTCCTCTCCCTCTCTCTCTGTCTCTCTCCAAAAAAAAAAAAAAAATGGTAAAAGACACTTGCTTGCAAAGCCTGCTGGCCTGGGATCAATTCTCTAATACACACATAAAGCCAGATGCACAAAGTGGCACATGCATCGGGAATTT
PVKW010006781.1   GCAGCTGAGAGACTGCTGGCGC-GCCAA-TTCTCTGTCTCCCTCTCTGTCTCTCTCTCTCTCCAAAAATAAATTTAAAAAAAAAAAAAAAGAAGAAGAAATGTTAAATGTGGGGCTGGAGAGAGGGTCCAGCAATTAAGGGTCTTGTCTGAAAATACTAAGGACCTGGGTTTGATTCCTTAGT
PVKW010012478.1   GCAGCTGAGAGGCTGCTGGCAC-GCCCA-TCTCTGTCTTCCTCTCCCTCTCTCTCTCTGTCTCTCTCTGTGTCTCTCTCAAAAAAAAAAAAAAAAAAGAAGAAAATGTTCTTCTTCACATATTAAAGATAACCAAAGTTCAGTGATATTAAGTATTTTGTCCAAACTCTTGGGCTATGAAAATGAA
PVKW010001726.1   GCAGCTGAGAGGCTGCTGGTGG-GCCCA-TCTCTATCTGTCTTCCTCTCCTTCTCTCTCTGTGTCTCCAAAAAAAAAAAAAAAAAAGATAGGGTCTCTTTTAGCTCAGGTTGGCCTGGTGATGACCTTGAACTTCTAATCTTCCTACTACTATCACCTAGTTCTGTGGTTATGGGCATGAGTC
PVKW010001449.1   ACAGCTGAGAGGCTGCTAGTGC-GCCCA-TCTCTATCTGTCTTCCTCTCCCTCTCTTTCTGTTTCTCTCTCCAAAAAAAAAAAAAAATTATATGTGCGATTCTCATAGCAGCATTTGTACAAAAAACAAGCACAGACTCTGTAGTGCATGGACCTTAAAAATTCTAGGTGGGGGGGGGCTGGAGAG
PVKW010003485.1   GCAGCCAAGAGGCTGCTGGTGC-GCCCA-TCTCTATCTGTCTTCCTCTTCCCCTCTCTCTGTCTCTCTCTCAAAAAAAAAAAAAAAAAAAAAACTTAGTGGGTCTCCTGGTCAGTGATCCTGAAGAATTTCAAAATGTTAACAGCAGAGTATTAAACTATATGGTAAGTAGGAAGGAGTTTCTG
PVKW010005006.1   GCAGCTGAGAGAATGCTGGCGC-GCCCA-TCTCTATCTGTCTTCCTCTCCCTCTCTCTGCATCTCTCTCCAAAAAAAAAAAAAAAGTTCTTGCCCCTTCACATGCTAAATAGGTGATTTGCACGTGTTAGTTTTTTCCCCATTCATCAAAGATAATGTCACAGGAACCTGCCAGAGACTGTCATG
PVKW010043933.1   GCAGCTGAGAGGCTGCTGGCGT-GCCCA-TATCTATCTGTCTTCTTCTCCCTCTTTCTCTCTGTCTCTCTCCAAAAAAAAAAAAAAATACTTTCAAAACCTCACCAGACACCTGAGCTGGCTGTGCACACGTGTGCATTGTCTATTTAGAGATCAGACTAACCAACAGCAATGCACCATGAGGTGCTT
PVKW010040692.1   GCAGCTGAGAGACTGCTGCCGC-GCCCA-TTTTTGTCTCCCTCTCTATCTCTCTCTGTCTCTCTCCAAAATAAATAAAAATAATAATAATAAATTTATTTTTAAATTGCCTTAAGGGGCCTGGAGAGGTGGCTTAGCGGATAAGCCCTTGCCTGTGAAGCCTAAGGACCCTGGTTTGAGGCTT
PVKW010002946.1   GCAGCTGAGAGGCTGCTGGCAC-GCCCA-TCTATGTCTTCCTCTCTCTCTCTCTCTCTCTGTCTCTCTCTGTGTCTCTCTCCACAAAAAAAAAGTACAAAAGGGGGGATTTGTCCAGGCATCAGTATTGTTAGAGGTTCCTCTGGGGCTTGAAGTGCTGTCTTTCTTGCAGGCCATGGGTCTGAAA
PVKW010022497.1   GCAGCTGAGAGACTGCTGGCGC-GCCCA-TCTCTATCTGTCTTCCTCTCCTTCTCTCTCTGTCTCTCTCAGTTCCTCTCAAAAAAAATTGTGTAAACTTCCTTTGACTCAGGGAACATATGGTCAGTGCCTCCTTTAGTTAAGTTCAGAAGATTAAGTTTGATTATGAAAAAAACACTATGAACAT
PVKW010014085.1   GCAGCTGAGAGACTGCTGGCAT-GCCCA-TCTCTCTCTCCCTCTCCCTCTCTGTCTGCCTCTCTCTGTCTCTCTCCAAAAATAAATAAGTAAATAAAAAAGAATGAAAAAAATCACCTCTGGTGGCGAAAACTAGCCTCTTGTTAGAAGTAACCGTCAACTTTTCTGTCCATCCCAACCAGGCAGCAA
PVKW010041458.1   GCAACTGAGAAACTGCTGGCGC-GCCCA-TCTCTATCTGTCTTCCTCTTTCCCTCTCTCTGTCTCTCTCTCTCTCTCTCTCTGTCTCTCCAAAAATTAAAATATATATATATATAGACCAATTTTATCCAAGGCTTTCTAGGGACATATATCTGAAAATAAACTTTACCAGGAGATAATCACCGG
PVKW010047843.1   GCAGCTGAGAGACTGCTGGCGC-GCCCA-TCTCTATCTGTCTTCCTCTCCCTCTCTCTTTGTCTCTCTCTGTGTCTCTCTCCAAAAATAAATAAAAATAATTTTAAAAAATC-TCATTCTTGTTCTAGTGGCACCACTAACATGGAGGAGGATATAAATCTGAAATTTCCAATGGAAAACCTCTGG
PVKW010109024.1   GCAGCTGAGAGGCTGCTGGTGC-GCCCA-TCTCTATCTGTCTTCCTCTCCCTCTCTCTGTGTCTCTCTCTCTCTCTCAAAAAATGAATGAAAGGACCTTTTATTTTATTTTATTTTATTTATTTTTCAAGCAGAGGGAGATAGAGACAGGCAGAAAAAATGGGCATGCCAGAGCCTTTAGCTGTTGC
PVKW010012339.1   GCAGCTGAGAGACTGCTGGCGA-GCCCA-TCTCTACCTGTCTTCCTCTCCCTCTCTCTCTGTCTCTCTCTGTGTCTCTCTCCAAGTAAAAAAACAATAAGTTGTTTATTTATTTATTGCAAGGAGATAAAGAGAGAGGTAAAGAAAGAAAGAGACAATGGGCACACCAGGGCCTGTAACCTCTGCA
PVKW010004996.1   GCAGCTGAGAGGCTGCTGGCAT-GCCCA-TCTCTATCTGTCTTCCTCTCCCTATCTCTCTGTGTCTCTATCCAAAATAAAAAACATAAATATATTAAAAGAAGTTATTATTGAAAGATATGTATTTAAACAACCTGACATAAGGCTCAAACAAGTGCAGTAGTGACACACAGTCATGGTGGGCAAC
PVKW010062488.1   GCAACTCAGAGACTGCTGGTGC-ACCCA-TCTCTGTCTGTCTCTCTCTCTCTGTGTCTCTCTCCAAAATAAATAAAAATAATAAAAAATAAATAAAGTGAGCGAGTGAGTAAGAGAAGAGAGAGAGAGAAAGAGAGGCTCCACAAATCCCAAGAACACAAGATGGTCCCTTTTTATATGTTTTCAGG
PVKW010046796.1   GCAGCTGAGAGGCTGCTGGCAC-GCCCA-TCTCTATCTGTCTTCCTCTCTCTCTCTCTGTCTCTCTCCAAAAAACAAAAAACAAAAACAAAAACAAAAAAACAAAGGACTTGAAAAGCAACTTAGTATAGGGTCACAGATAAACAGCGTACTAAACTTTTTTTTTTGTTGTTTTTTCAAGGCTGACCTAG
PVKW010011365.1   GCAGCTGAGAGACTGCTGGCAT-GCCCA-TCTCTGTCTTCCTCTCCCTCTCTCTCTCTCTGTCTCTCTCCAAAAAAATAAAAAGAATGTACAAAGCAAGAAAGTTTGAACTTGCTGTATTCAACCCTTTTTGTACAAAAGCAACTCATTCAAAGGGTTGAACAAGCTTATTACACACAGTACAG
PVKW010006850.1   GCAGCTGAGACACTGCTGGTGC-GCTCA-TCTCTGTCTTCCTCTCCCTCTCCCTCTCTCTCTCTTTCTCTCTCTCTCTCTCTGTGTGTCTCTCTCCAAAAAAACAAGAAAAGAAAAAAAGAAAAAAGGGGGAAAAAGACACTTCTCTTGAGTCACTTCTGCAGTGAGTCACAGACGTATATATT
PVKW010009979.1   GCAGCTGAGAGGCTGCTGGCAT-GCTCA-TCTCTATCTGTCTTCCTCTCCCCCGCCCCCTCTCTCTCTGTCTCTCTCAAAAAAAACCATGTTCCCAGTTGAACATCCAATGGAAACAGCCACCTGGAATTTTTTCCACCACTTCTGTTTGTGCAGCATATTCTTGAATCCTACATGATAAAAAA
PVKW010019386.1   GCAGCTGAGAGACTGCTGGCGC-GCCAATTCTCTATCTGTCTTCCTCTCCCTCTCTCTCTGTCTCTCTCTGTGTCTTTCTCTAAAAAAAAAATATTAATAAATAAAATAAAATAAAGTAAAAAG-ACAAAGACTTCCTGGTAAGATGGTGGCATAGGAGCCATGCCAAACCAGCCTAGGGAGGGAT
PVKW010050442.1   GCAGCTGAGACACTGCTTGAGT-GCCCA-TCTCTGTCTTCCTCCCTCTCCCTCTCTCTCTCTCTCTCTCTCTGTGTCTCTCTCCAAAAAAATAAGATAAAATAAAAAGTTAAAAAAAAGTGAGAAGATGGAAAGGCAAAGCCAGTATCTTGTTGGGTGAAGTGTTTTGTCACTTGACACAGTAGTCCTT
PVKW010018656.1   GCAGCTGAGATACTGCTGACGC-GCCCA-TCTGTCTTCCTCTCCCTCTCTCTCTGTGTCTCTCTCCAAAAAAATATTAAAAAATAAAAAGTTAAAAAAAAATAGGGCTGGAGAGATGGCTGAGCAGATACGGTACAAGTCTGCAAAGCCTAAGAATACAAGTCTGATTCACCAGTACCCATGTA
PVKW010019198.1   GCAGCTGAGAGGCTGCTGGTGT-GCCCA-TCTCTATCTGTCTTCATCTCCCTCTCTCTCTGTCTCTCTCCAAAACAAAAAAAAGTTCAGGCATGAGTGTATCAAGGGTTAGAAAGCTTTTCATGTGCCCTTTCCAGTTGTTAAAACAAACACGTTCTCATTACTTATTCATGCCAAACATTTCTGT
PVKW010039191.1   GCAGCTGAGAGGCTGCTGGTGT-GCCCA-TCTCTATCTGTCTTCCTCTCCCTCTCTCTCTGTCTCCAAACAAAAAAAAACCACATTATTCAAGGCTGGGTACATGACTTGCCCGGCATATGTATGGTCCTGGATTTAATCCTCATCACCAAAAAGAAATAAAAATAAAAACTACTAGACAGGTAAA
PVKW010084254.1   GCAGTTGAGAGACTGCTGGCAC-GCCCA-TTC---TCTGTCTCCATCTCCTTCTCTCTCTACAAAAACATAAAATAAAATAAAATAAAATAAAATAAAATAAAATAAAATAAAATAAAATAAAATAAAAAATAAAATAAAAATGAAATAAAATAAAATAAATAAAAATCTAAAAACAAACAAACAAAAAAA
PVKW010020145.1   GCAGCTGAGAGACTGCTGGCGC-GCCCA-TCTCTGTCTTCCTCTCCACCCCCCCCAAAAAAGAAAAAAATACTATGGAATGCTAACAAATATTTACATGTCTGTTTGAGGATTACAGATAATTCTAGTATTTTGTGGATTTGTTTTGTTGTTGTTTTTTTTCAGCATGTACAGATTTTCTCTACTT
PVKW010059942.1   GCAGTTGAGAGACTGCTGGCAC-GCCCA-TCTCTGTCTCCCTCTCCCTCTCTGTCTGTCTCTCTCTGTGTCTCTCTCCAAAAATAAATAAAATAATAAAAAAAATTTTTAAAAAGTGAGAGTAGCATTATATATGGGTGTTAACATTAACAGAAGTGCTTACCAGGCAGTTTGGTGAGCAAAATAA
PVKW010159600.1   GCAGCTGGGAGGCTGCTGGCAT-GCCCA-TCTCTATCTGTCTTCCTCTCTCTCTCTCTCTCTCTCTCTCCAAAATAAATAAATAAATAAACAAATAAAAAATAAATAAATAAATAAAATCCCCACCAATAAACAAGTAAATGAGCATTTACTGTTGAAATAAGCATTTCAACAAAAGTACAAATGTCTAA
PVKW010000100.1   GCAGCTGAGGGACTGCTGGCGT-GCCCA-TCTCTGTCTTCCTCTCTCTCTCTCTCTCTCTCTCTCTCTCTCTCTCTGTCTCTCTCTGTCTCTCTCCAAAAATAAAAAATTAAAAATTAAAAAGTGACTTCCAAAAAATGCATTGTTCTCCATAAGTTTTGGTAATGATTATTATAATAAACAGACAA
PVKW010072700.1   GCAGCTAA--------------TGCCCA-ACTCTATCTGTCTTCCTCTCCCTCTCTCTCTGTCTCTTTCCTAAAAAAATTTTTTAATGCATAATGGTGCATGTTAACTAAAAATAAAAGAATAAAATAATGATAGTACATACTGACAACATGCTCAATACTGTGGACAGAAATTTTATGTTGCATT
PVKW010000146.1   -------------TGCTGGCAC-GCCCA-TCTCTATCTGTCTTCCTCTCCCTCTCTCTCTGTCTCTCTTTGTGTACTCTTCTCCCCCCAAAAATTAATAAGTGATATAGTTCTAATGAACAAGAACATAGAGTAAGCCATTCATTCATCCAAAAGGGAATAATGGGGGTATAGCCAGGAAAGATCA
```

	Copy number	Proportion
T^+ copies	22	30 %
Conventional T^+ copies	6	8%
T^- copies	4 5	62%
Total	73	

Figure S11. Random Dip_b2 copies from the *A. bullata* genome. Nucleotide sequences are highlighted as follows: PASs, strong pol III terminators, medium terminators, rudimentary terminators, and oligo(A). Conventional T^+ copies do not contain either a terminator or an oligo(A).
