# Supplementary material for: Analysis of SINE Families B2, Dip, and Ves with Special Reference to Polyadenylation Signals and Transcription Terminators
Source: Int J Mol Sci. 2021 Sep 13;22(18):9897. doi: 10.3390/ijms22189897 (PMC8466645; doi:10.3390/ijms22189897)
Supplement: Supplementary file 1 [file ijms-22-09897-s001.zip › Supplementary Vassetzky/SINE Dip. Figs S7-S22. Tables S6-S8/Fig S13.pdf]

```
                                     *        20         *        40         *        60         *        80         *       100         *       120         *
PVKW010006534.1 : AAATTTGATATGCATTTTAATATCTACCAAGGAGGCTGGAGAGATGGCTTAGC----GGTTAAGGCGCCTGCCTGCTAAGCCTAAGAAC-CAGGTTCCAATCT----------------CCAGGTCCCATGTTTAG-
PVKW010018780.1 : TATAAGAATGTTTAACAAAATTAAAGAGGATAGGGCTGGAGAGATGGCTTAGA----GGTTAAGGTGCCTGCCTGCTAAGCCTAAGAAC-CCGGTTCCAATCT----------------CCAGGTCCCACATTTAG-
PVKW010013669.1 : TGGCACAAATGTAAGTTAAAAGATGAGGAAGAGGGCTAGAGAGATGGCTTAGC----GGTTAAGGTGCCTGCCTGCTAAGCCTAAGAAC-CCGGCTCCAATCT----------------CCAGGTCCCATGTTTAG-
PVKW010000122.1 : GAAGATAACATTTGAATAAGAATCTGAATTCTGGGCTGGAGAGATGGCTTAGC----GATTAAGGCGCCTGCATCCTAAGCCTAAGAATGCTGGTTCCTATCT----------------CCAGGTCCCACGTTTAG-
PVKW010004445.1 : TGATTTTTATTCATATAAAAACGAAACATGTCGGGCTGGAGAGATGGCTTAGC----AGTTAAGGCGCCTGCCTGCAAAGCCTAAGAAC-CCAGTTCCAATCT----------------CCAGGTCCCACATTTAG-
PVKW010031174.1 : TTCAGAAGACTGAGTGTAAGAGTATTACAATTGGGCTGAAGAGATGGCTTAGC----CGTTAAGGTGCCTGCCTCCTAAGCCTAAGAACGCCAGTTCCAATTT----------------CCAGGTCCCACATTTAG-
PVKW010009668.1 : ATTTAAAAAAATTAATTTAAAAATAAAAATAAGGGCTGGAGAGATGGCTTAGC----GGTTAAGGCGCCTGCCTGCTAAGCCTAAGAAC-CCGGTTCCAATCT----------------CCAGGTCCCACATTTAG-
PVKW010006270.1 : GTCCTGAGTGATGAGGATGTAACAGCCAACCTGGGCTGGAGAGATGGCTTAGC----TGTTAAGGCGCCTGCTTCCTAAGACTAAGAACGCTGGTTCCAATCT----------------CCAGGTCCCACATTGAG-
PVKW010010393.1 : TTCCCTTTTATTTTTTTTAAAAAGCAGGGCTCGGACTGGAGAAATGGCTTAGC----GGTTAAGGCGCCTGCCTCCTAAGCCTAAGAACGCTGGTTCCAATCT----------------CCAGGTCCCATGTTTAG-
PVKW010031684.1 : GATGGCTGGGTGGTTAAAGATGCTGGTTAAAGAGGCTGGAGAGATGACTTAGC----GGTTAAGGCGCCTGCCTGCGAAGCCTAAGAACGCCCGTTTGATTCT----------------CCAGGTCCCACGTTTAG-
PVKW010056884.1 : TTAGCACATCAAATATCATTAAAAGCAATGTCAGGCTGGAGAG--GGCCTAGT----GGTTTAGGCGCCTGCCTGCCAAGCCTCAGAACGCCTATTCGATTCT----------------CCAGATCCCACATTTAG-
PVKW010032239.1 : TGGACTTCTAATACTCAAGAATAGAGGTATTTGGGCTGGAGAGCTGGCTTAGC----GGTTAAGGCGCCTGCCTCCTAAGCCTAAGAACTCCGGTTCCAATCT----------------TCAAGTCCCACG-TTAG-
PVKW010023471.1 : TTTAACCTTTAAAAAAGGTTCCTTTTTTTGGAGGGCTGGAGAGATGGCTTAGA----GGTTAAGGTGCCTGCCTCCTAAGCCTAAGAACAATGGTTCCAATCT----------------CCAGGTCCCACGTTTAG-
PVKW010017375.1 : CCCTTCCCAGTGTTCTTAAAAACCACAGGCCAGGGCTGGAGAGATGGCTTAGA----GGTTAAGGCACCTGCCTCCTAAGCCTAAGAAC-CCGGTTCCAATCT----------------CCAGGTCCCACGTTTAG-
PVKW010014973.1 : TAAAAAAATTCAACAGCAAAATGGGCCACTGAGGGCTGGAGAGATGGCTTAGG----AGTTATGGCGCCTGCCTCCTAAGCCTAAGAACACTGGTTCCAATCTCCAGCAGTTCCAATCTCCAGGTCCCACGTTTAG-
PVKW010012032.1 : CTGAACTAGAATTGATCAATAAAAACGTGCCGGGGCTGGAGAGATGGCTTAGC----AGTTAAGGCGCCTGCCTCCTAAGCCTAAGAACACTGGTTCCAATCTCCAGGGGTTCCAATCTCCAGGTCCCACGTTTAAA
PVKW010037207.1 : TGAAAGCCTTAAAAAAAAAAAAAAAAGACTGAGGGCTGGAGAGATTGCTTAGG----GGTTAAGGCACCTGCCTCCTAAGCCTAGGAACACAGGTTCTAATCTCCAGCGGTTCCAATCTCCAGGTCCCACGTTCAG-
PVKW010001988.1 : TTTCTGAGGCAACTATAAAAACATAATTATTTGGGCTGGAGAGATGGCTTAGC----GGTTAAGGCACCTGCCTCCTAAGCCTAAGAACTCTGGTTCCAATCTCCAGCAGTTCCAATCTCCAGGTCCCACGTTTAG-
PVKW010021498.1 : GTCAGTTCAAATCATTTTAAAACTTAAAAATTGGGCTGAAGAGATGGCTAGGC----GGTTAAGGCCCCTGCCTCCTAAGACTAAGAACGCTGGTTCCAATCTCCAGCCGTTCCAATCTCCAGGTCTCACGTTTAG-
PVKW010000820.1 : GCTACATGAAATCCTATCTAAAAAAAAAAGGTGAGCTGGAGAGATGGCTTAGC----GGTTAAGGCGCCTGCCTCCTAAGCCTAAGAACACTGGTTCCAATCTCCAGCGGTTCCAATCTCCAGGTCCCACGTTTAG-
PVKW010001600.1 : GCTTGCCTTTCCTTATGTGAAAGCCAATTGGAGGGCTGGAGAGATGGCTTAGC----GGTTAAGGCACCTTCCTCCTAAGCCTAAGAACACTGGTTCCAATCTCCAGCAGTTCCAATCTCCAGGTCCCACGTTTAG-
PVKW010002752.1 : GTTCGATTAACATTTA--AAGTTAGCATAAAAGGGCTGGAGAGATGGTTTAGA----GATTAAGGCGCCTGCCTCCTAAGCCTAAGAACACTGGTTCCAGTCTCCAGCAGTTCCCATCTCCAGGTCCCACTTTTAG-
PVKW010000576.1 : TGTGCACAAAAGTAATATAAAAAGACTATCTCGGCCTGGAGAGATGGCTTAGT----GGTTGAGGCACCTGCCTCCTAAGCCTAAGAACGCTGGTTTCAATTTCCAGCGATTCCAATCTCCAGGTCCCACGTTTAG-
PVKW010006672.1 : GCAAGAGCAAGTGTTGTAAAGAATGTTGGTTAGGGCTGGAGAGATGGCTTAGC----AGTTAAGGCGCCTGCCTCCTAAGCCTAAGAACGCTGGTTCCAATCTCCAGCCGTTCCAATCTCCAGGTCCCATGTTTAG-
PVKW010003591.1 : TATCAAAAAAAAAAAAAAAAAAAAAAAACATAGGGCTGGAGAGATGGCTTAGC----GGTTAAGGCACCTGCCTCCTAAGCCTAAGAACGCTGGTTCCAATCTCCAGCGGTTCCAATCTCCAGGTCCCACGTTTAG-
PVKW010025398.1 : AGAGAGAAATAAAAAACCATAAAAAGCTTCCAGGGCTGGAGAGCTGGCTTAGT----GGTTAAGGCGCCTGCCTCCTAAGCCTAAGAACGCTGGTTCCAATCTATAGCGGTTCCAATCTCCAGGTCCCACGTTTAG-
PVKW010004907.1 : GCAATTCCTTGCATTTTTAAAAGCTGCTTCTGGGGCTGGAGAGATGGCTTAGC----GGTTAAGGCTCCTGCCTCCTAAGCCTAAGAAAGCTGGTTCCACTCTCCAGCTGTTCCAATCTCCAGGTCCAATGTTTAG-
PVKW010035556.1 : GAGATAGCTTAGCAGTTAAAGATGCTTACTTAGGGCTGGAGAGATAGCTTAGC----GGTTAAGGTGCCTGCCTCCTAAGCCTAAGAACGCCGGTTCCAATCTCCAGCAGTTCCAATCTCCAGGTCCCACGTTTAG-
PVKW010000339.1 : TGAATGCATTTATTTATATAAGAATTTTTTGCGGGCTGGAGAGATGGCTTAGT----GTTTAATCCACCTGCCTCCTAAGCCTAAGAATGCTGGTTCCAATCTCCAGCGGTTCCAAACTCCAGGTCCCACGTTTAG-
PVKW010019089.1 : CTATCACTTGTAGCCTTAAAAGTCTTGACTAGGGGCTTGAGAGATGGCTTAGC----AGTTAAAACACCTGCTTCCTAAGTCTAAGAACACTGGTTCCAATCTCCAGCAGTTTCAATCTCCAGGTCCTACGTTTAG-
PVKW010000834.1 : GTCTGTGTTTTAATTTTAAAAAAGCATTTTGTGGGCTGGAGATATGGCTTAG-------------CACTTGCCTCCTAAGCCTAAGAACGCTGGTTCCAATCTCTAGCAGTTCTAATCTCCAGGTACCACATTTAG-
PVKW010039861.1 : TTGTTTGGCCAAACTTGTTAAAACTGTAATGTGGGCTGGAGAGATGGCTTAGC----GGTTAAAGCGCCTGCCTCCTAAGCCTAAGAACACTGGTTCCAATCTCCAGCTGTTCCAATCTCCAGGTCCCACGTTTAG-
PVKW010013087.1 : GTACTGAAAACCTAATCAAAAGCCTATGACATGGGCGGAGAGATGGCTTAGG----GGTTAAGGTGTCTGCCTCCTAAGCCTAAGAACACTGGTTCCAATCTCCAGTGGTTCCAATCTCCAGGTCCCACGTTTAG-
PVKW010036625.1 : CTGGGACAAAATACCTTAAAAAAAAAAAGTTAGGGCTGGAGAGATGGCTTAGC----GGTTAAGGCGCCTGCCTGCTAAGCCTAAGAATGCTGGTTCCAATCTCCAGCAGTTCCAATCTCCAGGTCCCACGTTTAG-
PVKW010050425.1 : GGTGGGACCATGAAGAAAAAAATACCCTTTTTGGGCTGGAGAGATGGCTTAGC----GGTTAAGGCACCTGCCTGCTAAGCCTAAGAACGCTGGTTCCAATCTCCAGCGGTTCCAATCTCCAGATCCCACATTTAG-
PVKW010009699.1 : TTCAGAGGGAAAGATAATTTAAGAAGGCTTGTAGGCTGGAGAGATGGGTTAGT----GGTTTAGGCACCTGCCTGCTAAGCCTAAAGAATGCAGTTCCAATCT----------------CCAGGTCCCACGTTTAG-
PVKW010011748.1 : CCTGCAGACTTGGAGTAGAAATGGGTTACACAGGGCTGGAGAGATGGTTTAGT----GGTTAAGGCACCTGCCTGCTAAGCCTAAGCACG-TCATTCCAATCT----------------CCAGGTCCCACATTTAG-
PVKW010072702.1 : TATGGATGAGTCATCTTCGAAAACACACAGTGGGGCTGGAGAGATAGCTTAGT----GGTTAAGGCGCCTGCCTACTAAGCCTAAAAACACAGGTTCCAATCT----------------CCAGGTCCCACGTTTCG-
PVKW010009701.1 : GATGAAGTCGTAGTGTTAAAAAGTGCAACCAAGGGCTGGAGAGATTACTTAGT----GGTTAAGACACCTGCCTACTAAGCTTAGGAACGCCTGTTCAATTCT----------------CCAGGTCCCACGTTTAG-
PVKW010010323.1 : GGCAATGAAAATTTAATCAAAACCCTGTGGCTGGGCTGGAGAGATGGCTTAGC----AGCCAAGGGACCTACCTGCGAAGCCTAAGAATGCCCGTTTGATTCT----------------CCAGGTCCCATGTTTAG-
PVKW010014843.1 : GAATTATGGTTCTTACATTTAAAAAAAAGTTGGAGCTAGAAAGATGGCTTAGC----GGTTAAGGCGCCTGCCTGCCAAGCCTAAGAACCCTGGTTCCAATCT----------------CCAGGTCCCACATTTAG-
PVKW010062733.1 : TACTTCAAACCATTACATAAAAGCTCTTCATTGGGCTGGAGAGATGGCTTAGC----AGTTAAGGCACCTATCTGCTAAGCCTAAGAACACCGGTTCCAATCT----------------TCAGGTCCCACATATAG-
PVKW010058492.1 : AAGCACACAGTATCTTTAAAAATGACAGAGGAGGGCTAGAGAGATGGTTTGCTTAGCAGTTAAGGCACCTGCCTGTGAAGCCTAAGAACAC-CCGTTGGATCT----------------CCAGGTCCCATGTTTAG-
PVKW010012334.1 : AGTCTTCTGAAATAAATTATTAAAAAAACGAAGGGCTGGAGAGATGGCTTAGT----GATTAAGGTGCCTGCCTGCTAAGCCTCAGAACTCCGGTTCCAATCT----------------CCAGGTCCCACGTTTAG-
PVKW010005809.1 : GAGTATTCAAAGTATCAAAAAGCAATAGATCAGGGCTGGAGAGATGGCTTAGC----TGTTAAGGAGCCTGCCTGCTAAGCCTAATAATGCTGGTTCCAATCT----------------CCAGGTCCCATGTTTAG-
PVKW010021368.1 : TAGGTGAAGCCTCCAACAAGAACTACTTAGAGGGGCTGGAGAGATGGCTTAGG----G-TTAAGGCGCCTGCCTCTTAAGCCAAAGAACGCTGGTTCCAATCT----------------CTAGGTCCCACGTTTAG-
PVKW010021276.1 : AGGCATGAGGTGGCTTTATAAAAAACACATGGGGGCTGGAGAGATGGCTTAGT----GGTTAAGGCGCCTGCCCACTAACCCTAAGCACGCCGGTTCCAATCT----------------CCAAGTCCCACGTTTAG-
PVKW010091622.1 : AGGACATTCTGACTTAAAAAAAATGCCTAGCGGGGCTGGAGAGTTGGCTTAGC----GGTTAAGGTGCCTGCCTGCCAATCCTAAGAACGCCGGTTCCATTCT----------------CCAGGTCTCACGTTTAG-
PVKW010002734.1 : CAGGAAAATCCACAAGTTAAAAAGCTAACATAGGGCTGGAGAGATGGCTTAGC----AGTTAAGGCGCCTGCCTGCAAAACCTAAGAAAGCCTGTTCGAATCT----------------CCAGGTCCCACATTTAG-
PVKW010007034.1 : ACAAAATCTTATCAATCAAAAAATTAGCAATGGGGCTGGAGAGAAGGCTTAGCAG--GGTTAAAGCGCCTACTTGCAAATCCTAAGAACGCCCGTTTGAATCT----------------CCAGGTCCCATGTTTAG-
PVKW010006777.1 : TGGGTTATATGTGATAAGAAAGCAGAAAGCAGGGGCTGGAGAGATGGCTTAGC----GGTTAAGGCGCCTGCCTGTGAAGCCTAAGAACGCCCATTCGAATCT----------------CCAGTTCCTACGTTTAG-
PVKW010000703.1 : TTTGTTGTTCTTCAATGAAAATCCCCAAAATGGGGCTGGAGAGATGGCTTAGC----GGTTAAGGTGCCTGCCTCCTAAGCCTAA---------------TCT----------------CCAGGTCCCACGTTTAG-
PVKW010019070.1 : TACCTAGTTTTTGGTTAAAAGTACACATGCTTGGGCTGGAGATATGGCTTAGT----GGTTAAAGTGCCTGACTGCTAAGCCTAAGAACAAGCATTCGATTCT----------------CCAGGTCCCAGGTTTAG-
PVKW010032685.1 : ATTTGAGAATTAGCATCAAAACTATGAATGGAGGGATGGAGAGATGGCTTAGT----GGTTAAGGCAACTGCCTGATAAGCCTAAGAACATCCATTCGATTCT----------------CCAGGTCCCACGTTTAG-
PVKW010012381.1 : CTTTCATGAAGCTGTTTTTCGGATTTTCATGTGGGCTGGAGAGATGGCTTAGG----GGTTAAGGCGCCTGCCTCCTAAGCCTAAGAACACCCGGTTGATTCC----------------CCAGGTCCCACGTTTAG-
```

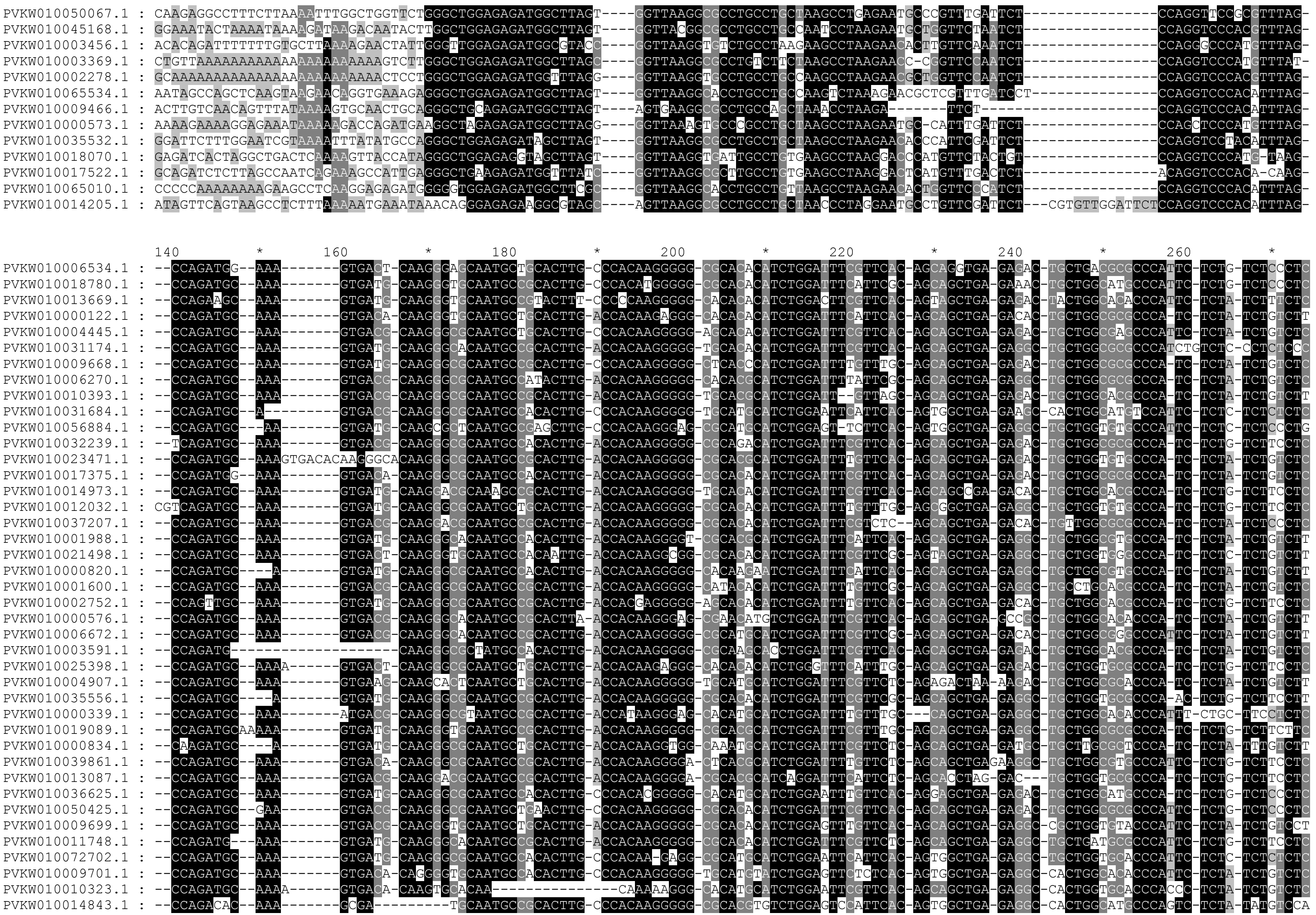

PVKW010050067.1
PVKW010045168.1
PVKW010003456.1
PVKW010003369.1
PVKW010002278.1
PVKW010065534.1
PVKW010009466.1
PVKW010000573.1
PVKW010035532.1
PVKW010018070.1
PVKW010017522.1
PVKW010065010.1
PVKW010014205.1
140 * 160 * 180 * 200 * 220 * 240 * 260 *
PVKW010006534.1
PVKW010018780.1
PVKW010013669.1
PVKW010000122.1
PVKW010004445.1
PVKW010031174.1
PVKW010009668.1
PVKW010006270.1
PVKW010010393.1
PVKW010031684.1
PVKW010056884.1
PVKW010032239.1
PVKW010023471.1
PVKW010017375.1
PVKW010014973.1
PVKW010012032.1
PVKW010037207.1
PVKW010001988.1
PVKW010021498.1
PVKW010000820.1
PVKW010001600.1
PVKW010002752.1
PVKW010000576.1
PVKW010006672.1
PVKW010003591.1
PVKW010025398.1
PVKW010004907.1
PVKW010035556.1
PVKW010000339.1
PVKW010019089.1
PVKW010000834.1
PVKW010039861.1
PVKW010013087.1
PVKW010036625.1
PVKW010050425.1
PVKW010009699.1
PVKW010011748.1
PVKW010072702.1
PVKW010009701.1
PVKW010010323.1
PVKW010014843.1

```
PVKW010062733.1 : --CCAGATGC--AGT---------GACG-CAAGGCCACAATGCTGCACTTG-CCCACAAGGGGG-CGCATACATCTGGAGTTCGTTCAC-AGCAGCTGA-GAGGC-TGCTGGCCTGCCCATTC-TCTA-TCTGTCTT
PVKW010058492.1 : --CCAGACAC--AAAAA-----GTAACG-CAAGCACGCAATGTCGCACATGAGCCACAAGGGGG-CGCACGCATCTGGAGTTTGTTCAC-GGCAGCTGA-GAGGC--CCTGGCATGCCCATTG-TCTC-TCTCTCTC
PVKW010012334.1 : --TCAGATGC--AAA-------GTGACG-CAAGGTGCAATGCTGCACTTG-CCCACAAGGGG--CACATGCATCTGGAGTTCGTTCAC-AGCAGCT----------------------------------CTCTCTC
PVKW010005809.1 : --CCAGAAGC--A---------GTGATG-CAAGGGCACAATGCCGCACTTG-ACCACAAGGGGG-CGCACACATCTGGAGTTGCTTCAC-AGCAGCTGA-GGCCCATTCTCTATCTGTTTCCC-TCTC-CCTCTCCC
PVKW010021368.1 : --CCAGATGC--AAA-------GG---------GGCACAATGCTGCACTTG-ACCACAAGGGGG-CTCACACATCTGGATTTTGTTCAC-AGCACCTGA-GAGGC-TGCTGGTGTGCCCA-TC-TCTG-TCTTCCTC
PVKW010021276.1 : --CCAGATGC--AAA-------GTGACA-AAAGCACGCAATGCTGCACGTG-ACCACAAGGGGG-CACATGCATCTGGATTTCATTCGC-TGCCGCTGA-GAGGC-TGCTGGCTCGCCCA-TC-TCTA-TCTGTCTT
PVKW010091622.1 : --CTAGACGC--A---------GTGTTG-CAAGGTGCAATGCCATGCTTA-CCCACAAGGGGAGCTCACAGGCCTGGAGTTCGTTTAC-AGCTGCTGA-GAGGC-TGCTGGCGCACCCATTC-TCTC-TCTCTCTT
PVKW010002734.1 : --CCAGATGT---AA-------GTGAGG-CAAGTGCGCAATGTTGCCTGTG-TGCACAAGGGGG-CGCATGTGTTTGGAGTTCATTTGC-AGCTGCTGA-GAAGC--CCTGGCATGCCCATTC-TCTC-TGCTTCTT
PVKW010007034.1 : --TCAGATGC--ACAA------GTGACC-CAAGTGTGCAATGCCACACATG-CGCATAAGAGGA-TGCACGCATCTGGAGTTCGTTCAC-AGCAACTGA-GAGGC--CCTGGTGCACCCATTC-TCTC-TCTCTACC
PVKW010006777.1 : --CCAGATGC--AAAA------GTGATG-CAAGTGCGCAATGTTGTACATG-TCCACAAGGGGG-AGCACACGTCAGCTGAGAGGTCCC--------------------GGTGCACTCATTGTCTGCCTCTTCCTC
PVKW010000703.1 : --CCAGATGC--ATA-------GTGACA-CAAGGACGCAATGCCGCACTTG-ACCACAAGGGGG-TGCACGCATATGGATTTTGTTAGT-AGTGGCTGA-GAGGC-TGCTGTAGCGCCCA-TC-TCTA-TCTGTCTT
PVKW010019070.1 : --CGAGATGC--A---------GTGACA-CAAGAGTGCAATGTCACACTTG-TCCACAAGGGGG-CGAACACACCTGGATTTCATTCAC-AGCAGTTGA-AAGGC-TGCTAGCGTGCTCATTC-TCTAATCTCTCTA
PVKW010032685.1 : --CCTGTCAC--ATA-------GTGACA-CAAGGGTTCAATGCTGCACTTT-CCCACAAGGGGG-CACACGTGTCTGGAATTCGTTCAT-AGCAGCTGA-GAGGC-TGCTGGCATGCCCATTC-TCTG-TCTCTCCC
PVKW010012381.1 : --CCAGACAC--AAA-------GTGATG-CAAGGTGCAGTGCTGCACTTG-CCCCCAAGGGGG-TGCACGTGTCTGGAGTTCATTCAC-TGCAGCTGA-GAGGC-TGCTGGTGTGCCCATTC-TCTA-TCTGTCTC
PVKW010050067.1 : --CCAGACGC--AAA-------GTGACG-CAAGGTGCAATGCCGCGCTTG-CCCACAAGGGGG-CTCATGCGTCTGGAGTTCGTTCAC-AGTGGCTGA-GAGGC-TGCTGGTGTGCCCATTC-TCTC-TCTCTCCA
PVKW010045168.1 : --CCAGACAC--AAAAT-----GTGACA-TAAGGTGCAAAGCTGCACTTG-CCCACAAGGGGG-CGTATGCATCTGAAATTCATTCAC-AGTGGCTGA-GAGGC-TGCTGGTGCACCCATTC-TCTC-TCTCTCTC
PVKW010003456.1 : --CCAGACGC--AAAA------GTGACG-CAAGTGCACAATGTCACACATG-CACATAAGGAGG-CGCACACATCTGGAGTTCATTCAC-AGAGCCTGA-AAAGG-CCCTGGCATGCCCATTC-TCTC-TCTGCCTC
PVKW010003369.1 : --CCAGATGC--AAA-------GTGACA-CAAGGGCACAATGCCGCACTTG-CCCACAAGGGGA-CGCACACATCTGGATTTCGTTCTC-AGCAGCTGA-GAGAC-TACTGGTGCGCCCATCTCTCTC-TGTCTCTC
PVKW010002278.1 : --CCAGACAT--AAAAA-----GTGACA-CAAGGTGCAATGCCATACTGG-CCCACAAGGGGC-CGCACGCGTCTGGAGTTTGTTCAC-AGATGCTGA-CACAC-CCATTCTCTCCCTC-TC-TCTC-TCTCTCTC
PVKW010065534.1 : --CCAGACGC--AA--------GTGACC-CAAGCACACAATGCTGCACTTG-CCCACAAGGGGA-CACACGCATCTGGAGTTTAGTCAC-AGCGGTTGA-AATGG-CCCTGGCGCACCCATTC-TCTC-TTTCTCTT
PVKW010009466.1 : --CCAGATGC--AAA-------GTGACG-CAAGGACACAATGCTGCACTTG-CCCACAAGGGGG-CGCACGCTTCTAGAGTT-TGTTCACAGAGTCTGA-GAGAC-CACGGGCACATCCATTC-TCTT-TCTCTCTC
PVKW010000573.1 : --CCAGACAC--AAA-------GTGATG-CAAGGGGCAATGCTGCTGTTG--CCCACAAGGGG-TGCACATATCTGGAGTTCATTCAC-GGCAGCTGA-GAGGC-CGCTAGAGTGCCAATTC-TCTA-TCTCTAAC
PVKW010035532.1 : --CCAGACGT--AAA-------GTGAAG-CAAGCACCCAATGCCACGTGCA-CGTGTCTGGAGT-TCGCTCGCAGTGGCTGAGAGGCCA-TTGGTGCAC-CCGTT-CTCTGTCTCTCTCTCCC-TCTC-TGTCTCTC
PVKW010018070.1 : --CCAGATGC--ACAAA-----GTGATG-CAAGCGTGCAAGGTCA-ACATG-CACACAAGGTGG-CACATGAGTCTGGAGTTGATTGC-AGTGACTGG-AGG---CCCTAACACACCAACTC-TCTC-CCTCTCTC
PVKW010017522.1 : --CCAGATGC--ATG-------GTTACA-CAAGCATGCAATGTTGCACATG-CACACAAGGTGG-CGCATGTGTCTGGAGTTTGATTTC-AGTGGTTGG-AGA---CCCTGGTACACCAACTC-TCTC-TCTCTCTC
PVKW010065010.1 : --CCAGATGC--AAA-------GTTATG-CAAGGGCGCAATGCTGCACTTA-ACCACAAGGGGG-TGCATGGGTCTGGAGTTTGATTCTCTATCTGTCTC-CCTCT-CTCTCTATCTCTCTG-TATC-TCTCTCCA
PVKW010014205.1 : --CCAGATGC--AAAAA-----CTGAGG-CAAGGGTGCAATGTCGCGCCTG-CCCACAAGTGGG-TGTACACGTCTGGAGTTCCTTTCC-AGCTGCTGA-GAGGC-TGGTGGTGTGCCCATTC-TCAA-AAAAAAAA
```

```
                    280         *        300         *        320         *        340         *        360         *        380         *        400         *
PVKW010006534.1 : TCCCTCTCTCTCTCTCTGTCTGTCTCTCTCTGTGTCTCTCTCCAAAAATAAATAAATAAATAAATAAATAAATAAATAAATAAATAAATAAAAACGTAAATATCTACCAAGGAATTTATCAGGAATATTGTCACATT
PVKW010018780.1 : TCCCTCTCTGTCTGTCTGTCTCTCTGTGTCTCTCTCCAAAAAAAAAAAAAAAAAAAAATTAAAGAGGATACAGATAAACTAAAATAATATAAAAAGATACAAATAAACACTGAGGAAATAAAGTCAGTATAGGATAA
PVKW010013669.1 : CCTCTCCCTCTCTGTCTGTCTGTCTCTCTCTGTGTCTCTCTCAAAAAAAAAAAAAAAAAAAAAAGATGAGGAAGAGAAAATAAGTCCAGAAATAAACCTTCTTATACATAATAAAGATATTTTCAACAAAGGTACTAG
PVKW010000122.1 : CCTCTCTTCCTCTCCCTCTCTCTTCTCTGTCTCTCTCAAAAAAAAAAAAAAAAAAAAAAAAAAGAATCTGAATTCTGTCACCAGACCAAGTCAACACATAGTATAAGTCATTAACTTCTGCAACAAATCACTAGGTC
PVKW010004445.1 : CCTCTCCCTCTCTCTCTGTCTCTCTCTGTGTCTCTCTCCAAAAAAAAAAAAAAAAAAAAAAAAAAAAAAAAAAAAAAAAAAGAAACATGTGGCTAGAAGCCAACTATTCAGTTCCTCCTCACCCTCTACACTCCATC
PVKW010031174.1 : TCTTTATGTTTCTCTGTGTCTCTCTCCAAAAAAAAAAAAAAAGAGTATTACAATAAAAACATTTATTTATTTATTATTTATTTATTTTGAGGTAGGGTCTCACTCTAGCCTAGGCTAACCTGGAATTCACTATGCA
PVKW010009668.1 : CCTCTCTCTGTCTCTCTCCAAAATAAATAAATAAATAATAAATAAAAATAAAAAAATAAAAAGCAGAAAAGTGTGAAAGTGACAGTTTTATATGACCTCACTGTGTGGTCACTGTCCATGCACTGTGTGCATTGTTT
PVKW010006270.1 : CCTCTGTCTCTCTCTGTCTCTCTCTGGCTCTCTAAAAAAAAAAAAAAAAAAAAAAAAAAACCAGCCAACCACTACCCAGTAGCAGACTGAGGCTGCATAATTACTGTGCCTGTACTTAGGACTAGCCTGAGAGAGT
PVKW010010393.1 : CCTCTCCTTCTCTCTGTCTCTCTCTGAAAAAAAAAAAAAAAAAAAAAAAAGCAGAGCTTCATTTTAGCCTAGGCTAATGCTGAACTCACCCTGTAGCCCAGGCTGGCCTCAAACTCACAATAATCCTCCTACCTTAGCT
PVKW010031684.1 : TCTCTCTGCCTCTTCCTCTCTCTCTCAAAAAAAAAAAAAAAAAAAAAAAAAAAAAAAAAAAAATGCTTGTTTGCCAAGCCTGCTGGCCCAGGTTCATTTCCCCAGTATGTACATAAAGCCATTTGTAGTGGAAAAAG
PVKW010056884.1 : CCTCTTCCTCTCTCTGTTTCTCTCTGTCTCTCTTTCTCTCAAAAAAAAAAAAAAAAAAAAAAGCAACGTCATCATTACTATGAGGCTTCTATCCGTGGTATACAGAAGCACTCTAAAATCTTCATTGGCAAGCCTCT
PVKW010032239.1 : TCCCTCTCCCTCTCCCTCTCTCTCTCTCTCTCTCTCTCTCTCTCCCAAAAAAAAAAAAAAAAAAAAGAATAGAGGTATTGGAGAGGCGTGTCTGCTTCTGTTTGGATACTCAACAAGCTCAGGAAGAAGAGAG
PVKW010023471.1 : CCTCTCCCTCTCTCTCTGTCTCTCGTTGTCTCTCTGTATGTCTCTCTCCAAAAATAAATAAAAATTAAAAAAATTAAAAATAAAAAAGGCTCCTTTTTGATAATCTGTTCTATAATGTGTTAACTGCGATGCAAAA
PVKW010017375.1 : CCTCTCCCTCTCTCTCTGTCTGTCTCTCTCTAAAAATAAAAAATAAAAAAAAAATCACAGACCAAAGCAGAACCTTATTTTTTCATCCCTCAAAAAAAAAAAAAAAAAAAAAAAAGATTTAATAAAGCATGCATCTGA
PVKW010014973.1 : TCCCTCTCCCTCTCCCTCTCTGTCTCTCTCTCTCTCCAAAAAAAAAAAAAAAAAAAAAATGGGGCACTGAACATTCTTTCCACCGCTAGCCTCAGTCAACACCTGGACCATGGTGTCAGAAGGCCACAGCCAAGGGA
PVKW010012032.1 : TCCCTCCCTCTCTCTCTCTGTCTTTCTCTGTGTCTCTCTCCAAAAAAAAAAAAAAAAAAAAAAAAAAGTGTTGGATTGCAACTAGGAAGCTTAGGTTGAGTTGCTGTTCTGAAAATGTTGCTGATCACCTACCTAAAA
PVKW010037207.1 : TCCCTTTCTCTCTCTCTCTATTTATCTCTGTCTCTCTCCAAAAAAAAAAAAAAAAAAAAAAAAAGACTGACTTTCCAGAAAGAATTTTGTCAGTTAATTGCCTTAAGACTTGAATTCTAAGTATTCTTTTAGTCTCC
PVKW010001988.1 : CCTCTCCCTCTCTCTTTGTCTTTCTCTGTGTCTCTCTCCAAAAAAAAAAAAAAAAAAAAAAAAAAAAAACAATGATTTGAAACTTTAAGATTATATTCTTAGGATATTAGAATTTTGTCAGTTATATAAGTCAGCTTG
PVKW010021498.1 : CCTCTCCCTCTCTCTCTGTCTCTCTCTGTGTCTCTCTCCAAAAAAAAAAAAAAAAAAAAAAAAAAAAAAAAAAAAAAAAAAAAAAAAAAACTTAATAATTACTATTAAAGAAACAGCACCAGCATATATGCATCATTAGT
PVKW010000820.1 : CCTCTCCCTCTTTCTCTGTCTCTCTCTGTGTCTCTATCCAAAAAAAAAAAAAAAAAAAAAAAGGTAAACCCAAAGTCATGATACAAATAGCATAAAAATTATTAGATGAGTACTTAAGACTTTAGCATGGAGAGCTTA
PVKW010001600.1 : CCTCTCCCTCTCTCTCTATCTCTCTCTGTGTCTCAAAAAAAAAAAAAAAAAAAAAAAACCTATTGGATATCACCTCCCTCCTATTTATATTGACATTTATGTTATTCCTCAAAAATGAATAAAAGAATGTACCTCAC
PVKW010002752.1 : TCTCTCTCTCTCTCTCTCTCTCTCTGTGTGTCTCTCTCCAAAAAAATAATAAAAAAGAAAAAGTTTAAAGTTAGCATAAAAGTAATGGGTTTCATTATATTTTCATACATATATGTCACTATACTTCGTTTCTCACATG
PVKW010000576.1 : CCTCTCCCACTCTCTCTGTCTCTCTCCAAAAAAAAAAAAAAAAAAAAAAGAGAGAGACTATGTATGTGTGTTTAATAGCACAAAAGAGGTCATTGAATTGTAGTACAAGAACTAAATTTTATGGGCCTCTAACAAGT
PVKW010006672.1 : CCTCTCTCTCCTCTCTCTCT---------GTTTCTCTCCAAAATAAATAAATAAATAAAAAGAATGTTGGTTAATAATGTGTTCTTGCATTTATAGCACCAGCCTGGGGAGAAGTGTCAGATGCTTCCTTCTTTCTT
PVKW010003591.1 : CCTCTCCCTCTCTGTCTCTCTCTCTGTCTCTCTCCAAAAATAAAATAAAATAAAATAATAAAATAAAATAAAATAAAAACCATATAGGACAGTAGGTTAGTGGAGTGGATGTCTCTTTTGGACTACTCTTTCATCTG
PVKW010025398.1 : TCCCTCTCTCTCAGTCTCTCTCTGTCTCTCTCCAAAAAAAAAAAAAAAAAAAAAAAAAAAAAGCTTCCAAGATATTAGTTATGGATTCAGAGCTGTTCTTGACCATCCCAGTTTCATGCAAAGAACCACAGAGCCTA
PVKW010004907.1 : CCTCTCCCTCTTTCTCTCTCTCTCTGTGTCTCTCTCCAAAAAAAAAAAAAAAAAAAAAAGCTGTTTCTCTCTCTCTTTTTTTTAATATATAATATGCTCTTGTTAGTGGGAATTGTAGGGGTAATTAGCTTGTC
```

```
PVKW010035556.1 : TCTCTCTGTCACTCTCTCTCAAAAAAAAAAAAAAAAAAAAAAAAAGATGCTTACTTATAAGCCTGCTGGCCAGAGTTCATTTCCATAGCATCCATATATATATATAACATGGAGCTACTGTTTGTAAGTACTACCCTCC
PVKW010000339.1 : CCCCTCTCTCTCTGTCTCTCTCTGTGTCTCTCTCCAAAAAAAAAAAAAAAAAAAAAAAAAAGAATTTTTGCATTTTTGCATGGCTGGAGAAAGAACTTAACAGTTAATGCTTTGCAGTAGCTGGAGACTCTGGAATA
PVKW010019089.1 : TCCCTCCCTCTGTCTCTCTCTCCAAAAAAAAAAAAAAAAAAAAAAAAGTCTTGACTAAGACAAATGGTGAAAACTTAAACCATCACAGTAAAAGCAAAGTTGACTGCATTTAGTCATTAAGAACCTAAACTGGGCAT
PVKW010000834.1 : CCTCTCCCTCTTTCTCTGTCTCTCTCTGTGTCTCTCTCCAAAAAAAAAAAAAAAAAAAAAGCATTTTGTATTATATAAAGTAACACTCTGTAAATAAGTATCATTAATGTAAAAGCAATATATATCCAAGTATATCT
PVKW010039861.1 : TCCCTCTCTTTCTCTGTGTCTCTCTCTCAAAAAAAAAAAAAAAAAAAAAAACTGTAATGCAAGAGCCAAATGCACTGTGATGTGTCACCAACGAATTAGCAAACATGACTTTTTCACCTGTGAGTTGATTAAAGAAA
PVKW010013087.1 : TTCCTCTCCC-CTTTCTCTCTGTCTCCCTCCAAAAAAAAAAAAAAAAAAAAAAAAAAAAAAAAAAAAAAAAAAGCCTCTGACAGGGGAGGTCATGAGCCTTAGGGGTATAAAGCCTGCTCTTGTCTGGCTAAATGTATA
PVKW010036625.1 : TCCCTCTCTGTCTGTCTCTCTCCAAAAACAAATAAAATAATAATAATAATAAAACAGTTTAGGGAAGCAAGGGTTTATTTCAGCTTACAGTTCCATCTTGCAGTCCATCATGGTGGGAAGGGCAGGGTGGCAGGAGC
PVKW010050425.1 : TCTCTCTGTCTGTCTCTCTCCAAAAAAAAAAAAATATATATATATATATATATATACCCTTTTCAATAGAGTCTGGAACAAGTCACTCCTCCTTATCCTTTCTCCCCTCTTTCCTCACACCTACCAAATAATACCA
PVKW010009699.1 : CCTCTCCTTCTCTCTCTCCAAAAAAAAAAAAAAAAAAAAAAAAAAAAAAAAAAAAAAAAAAAAAAAAAAGAAGGCTTGCATTTCACAAGAAGGGTATAGTGTATATTTCTATGTAAATGTAAATTTAGTCAATTGCTCT
PVKW010011748.1 : TCCCTCTCCCTCTCTCTCCAAAAAAAAAAAAAAAAAAAAAAAAAGGAAAAAGAAATGGGTTACACAGTATGTCCCCAAAGTTGTAGGACAGGCATGTCCTTTCTTTCCCGAAAGTGGAGTCTGTCCTAGGGAAACAG
PVKW010072702.1 : TCTCTGCCTCTCCCTCTCTCTGTGTCTCTCTCAAAAAAAAAAAAAAAAAAAAAAAACACCATGCTCATTCTTACATATCTGGGAATTGACCACAGTCAGTTGAATAGCAGCTGTTTGTTTCCAGTGGCTTATCTTCA
PVKW010009701.1 : TGTCTCTCCCCCAACCCCCGTCTTTCTCTCAAAAAAAAAAAAAAGTGCAACCAAATGGGACTTAATCCCAGCATTTGGGAGGCCAAGGTAGGAGGATCACCATGCAGCCATCCTGAGACTGCGTAATGAATTCCAGG
PVKW010010323.1 : TTCCTCTCTGTCTCAAAAAAAAAAAAAAAAAAACCTATAGCTTGGAAGGTCATAAGCCCTAAGGAGAACTACTACTGTTGTCTGGCTAAATAGATATATTATGCCCACAAAAATGCCCTCTAAGTGCCGGGTGTGGT
PVKW010014843.1 : TCTCTCTCTCTCTCTCAAATTAAAAAAAAAAGTTTACTAGGTCAAAAAGTGCTAGGAATACTTGAGTTTTTATTGCTTTTAAAATTTAGATGAGCCAGGTGTGGTGGCACATGCTTTTAATCCCAGCACTCAAGA
PVKW010062733.1 : CCTCTCCCTCTCTCTCTATTTCTCAAAAATAAAAAAAAAAAAAAAAAAAAAAAAGCTCTTCATTGCCAGGAGTGGTGGCACATGCCTATAATCCTAGCATACAGGAGGCTGAGGCAGGAAGATCATGAGTTCAAGGCC
PVKW010058492.1 : TCTCTCTCTCTCTGTCTCTCTTTCTCAAAAAAAAAAAATGACAGAGAAGCCGGGCATGGTGGCACACACCTTTAATCCCAGCACTCAGAAGGCAGAGGTAGGAGGGTCACTGTGAGTTCAAGGCCAGCCTGGGATTA
PVKW010012334.1 : TCTGTGTCTCTCAAAAAAAAAAAAAAAAAAAAAAAAAAAAAACCGAAAAGCCAGGTGTCATGGATGGTGGAACACTTTTAATCCCAGCACTCAGGAGGCAGAGGTAGGAAGATTGCCATGAGTTTGAGGCCAGCCTGA
PVKW010005809.1 : TCTCTCTCTGTCTCTCTCTGTGTCTCTCTCTCAAAAAAAAAAAAAAAAAAAAAAAGCAATGGATCAATAAAAATAAAGTTGCATGTGTGTAATTGGATGCTAGTGATTTTGAACAAAACAATTCAATGGAGTTATCT
PVKW010021368.1 : TCCCTCTCTCTCTGTGTGTCTCTCTCCACAAAAAAAAAAAAAAAAAAAAAAAAAGAACTACTTAGAGGAAATTCCATCCAAGAATTCTAAAACATGATTATGATAAATATAGCAAATTTAAAGAGAACATAAACAAA
PVKW010021276.1 : CCTCTCCCTCTCTCTGTGTCTCTCTGCAAAAAAAAAAAAAAAAAAAAAACATGGTCTTTGCTTTCAGCCTAAGAGTGACCAATCCTATGTGTCTACGTGTGACCTTTGGCGATTCCCTCAACGTCTCAGTCAAAGGG
PVKW010091622.1 : TCTCTCTCTGGCTCTCTCTCTCTCAAAAAAAAAAAAAAAAAAAAAAAAAAAAAAAAAAAAAAAAAAAAAAAAAAAAGCCTAGCCATGCCCATGGTGCAAGACATGTAGTCCCACTTGCAAGGGACTCTGAGCTTGAGG
PVKW010002734.1 : CCTCTCTCTTTCTCAAATTTAAAAAAAAAAAAAAAAAAAAAAAAAAAGCTAACATACAGGAATCAAGTGCCATTTGGAAAATAAAAACATCAAAGCAATCACCATTAAAACAGTATTATTAAAAGAACTAGAAGTTT
PVKW010007034.1 : TCTTCCTCTCTCTCTCAAATTAAAAAAAAAAAAAAAAAAAAAAAAAAAAAAAAAAAAAAAAAAAAAAAAAAAAAAAAAAAAAAAAAAAAAAAGCAAGTGGCGCACACCTTTAATCCCAG
PVKW010006777.1 : TATCTCTGTCTCTTTCTCTCTCAAATTAAAAGAAAGAAAGAAAGAAAGAAAGAAAGAAAGAAAGAAAGAAAGAAAAAGAAAGAAAGAAAGCAAACAAGCAAGAAAGCAAGCAAGCAGGCAAGCAGAAGGAGGAATTA
PVKW010000703.1 : CCTCTCCCTATCTCTCGGTCGCTCAAAAAAAAAAAAAAAAAAAAAAAAAAAAAAAAAAAAAAAAAAAAAAAAAAAAAAAAAAAAAAAAAAAAAAAAAAAAAAAAAAAGAAAAGAAAAGAAAAGAAAAAGA
PVKW010019070.1 : TCTCTCTCTCTGATTCTCTTTCTCAAAAAAAAAAAAAAAAAAAAAAAAGTACACATGCTTGTTTGCATCTGGTGCCAAGTCAGGCAGAGTCCTGTTGTGGCTTCTATGTCAAGAAGCTTGTTCTCCTGGAAGGACAA
PVKW010032685.1 : TCTCTCTCTGACTCTTTCTCAAAAAAAAAAAAAAAAAAAAAAAACTATGAATGGAGCCCTGGCAATATCTAAACCCAAGCCAGGATCTTAAGACTTGTTTTATCTTTCATCATACAGATAAGGAAGCCCAGAAGAGA
PVKW010012381.1 : TCTCAAAAAAAAAAAAAAAAAAAAAAAAGGATTTTCATGCACCCTTTGGTGAAGTTACTTATAATTATGTATAAATTCACACAATCAAATTGAATAACAAGGTAGGTTGGATATGTCATAATTTCTGATATGCAAGA
PVKW010050067.1 : AAAAAAAAAAAAAAAAAAAAATCGGCTGCTCCTGAGGACAAAACTCAGAGTGTGGTTTGGATTGTAAACACATTTTGGGATCAAAGACTGATAAGGAAGATACTTGAGTGATTTGGTTCTAGGCCTCAGAGGTGATG
PVKW010045168.1 : CAAAAAAAAAAAAAAAAAAAAAGATTAGACAATACAAAATAGACTATGAATCACCTGGAGAAAATATCTGAATCAAACTATAGCATTGAAAAAATAATGTAAAACATTAAAAAATATATAAAATGTGATCTTGTCAA
PVKW010003456.1 : TTCCCCCCTCTCTCTCTCTCTGTCTCTCTCTCTCTCAAAAAAAAAAAAAAAAAAAAAAAGAACTATTGTTGTGCCTTTCACTTTGTACATGAAGGAACTGTATTTTGGAATAAGTGACTTACTTCAGTGTCCATAGC
PVKW010003369.1 : TCCAAAAATAAATAATTTAAAAAAATTTTTAAAAAGTCTTATGTGAGGAAGCAGCACAGCATGTGCCCCAATCACAATCCATCAGCCATGGTTGCATAAATGGAGAGCCTGGGAGCAAAGGGTCATGAGCTTTCTCC
PVKW010002278.1 : TTTCTCTCTCTCTCTCTCTGCCTGTCTCCCTCAAAAAAGAAAAAAAAAAAAACTCCAACATTATTCCTTAGCACAAAGCACGTTATGCTACCTCCAAACTCAGACCTACTATGGCCTCCAGTGGAAGCATGGAGGACA
PVKW010065534.1 : TCTCAAAAAAAAAAAAAAAAAAAAAAAAAGGGCACCTGATGTTTCTCTCCCATAATTGAGCACACCTCCCTGCAGGTGAGCACACAGGCACAAGAGCACATACATGTTGCATACATAACACACACACATATGATATG
PVKW010009466.1 : TCTCTGACTCGCTCAAAAAAAAAAAAAAAAAAAAAAACTGCAACTGCAGAGTGTATTATCTGACCACAATGTAATTATGCTTTGCTGATAAGCATTAGAAAACTCTTAAATATTTTGTCTTTTATCTGTAAAATTTA
PVKW010000573.1 : TCTCTCAAAAAAAAAAAAAAAAAAAAAAAAAAAAAAAAAAAGGAAGACTAGATGAGCATGAGCATTTTCCTTTCTGTTACTTGGCCCATGACATGAACTGCTCTCCCTGCTTTGGATAACTATATTCTTTGAAAATA
PVKW010035532.1 : TCCAAAAAAAAAAAAAAAAAAAAATTATATACCAGCAAGAGGGCCTGGGAAATTAGTGGCATGTGTCTTCAATCACTCTGATGTCTGATCGGAGAGTCTACGAATTCACAGCTGCTGCTTTGAAACCGAAATCCCCA
PVKW010018070.1 : CTCTCTCACTCATAAAGGTGTGTGCCACCACACTCAGCAAAAAAAAAAAAAAAAAAAAAAAAATTAGCATAATGCTGAGAAGAAATAACAGTGGAGTGTTCAGCACTGAGACGTCTCTATCACACCTTCCAAGACTCA
PVKW010017522.1 : TGTCTCTCACACATAAAAGAAAGAAAGAGAAAGAAAGAAAGAAAGAAAGAAAGAAAGAAAGAAAGAAAGAAAGGAAGGAAGGAAGGAAGGAAGGAAGCCACTGTTTACCCACCAAGGCTGTGTTTCACC
PVKW010065010.1 : AAAAAAAAAAAAAAAAAAAAAAAAAAGGAGAGATGAAGCTGAGGCTACCTTCAGGGAGGTGAACCCTCACCTTGTCCTTCCTGCCCTAGGTACTCAGCCCAGGCCCAAGCCCTGGACCACAAATGCACTTGCTGTAA
PVKW010014205.1 : AAAAAAAAAAAAAGAAATAAACAAACAATTTTGTCCACAATGATTGTGTGCTTGTGATCCCTAATGGCAGTCTGTTACCCATTCTGAGATGGGCCGTGGTAAAAAAGGAAAAAAGAGAGAGAATTTGTCTTAAATGT
```

Figure S13. Random Dip_b copies present in the *A. bullata* genome, but absent in homologous loci of the *J. juculus* genome. All 68 copies have T^{-} tails. The nucleotide sequences of PASs and oligo(A)s are highlighted in green and yellow, respectively.
