# Supplementary material for: Analysis of SINE Families B2, Dip, and Ves with Special Reference to Polyadenylation Signals and Transcription Terminators
Source: Int J Mol Sci. 2021 Sep 13;22(18):9897. doi: 10.3390/ijms22189897 (PMC8466645; doi:10.3390/ijms22189897)
Supplement: Supplementary file 1 [file ijms-22-09897-s001.zip › Supplementary Vassetzky/SINE Dip. Figs S7-S22. Tables S6-S8/Fig S14.pdf]

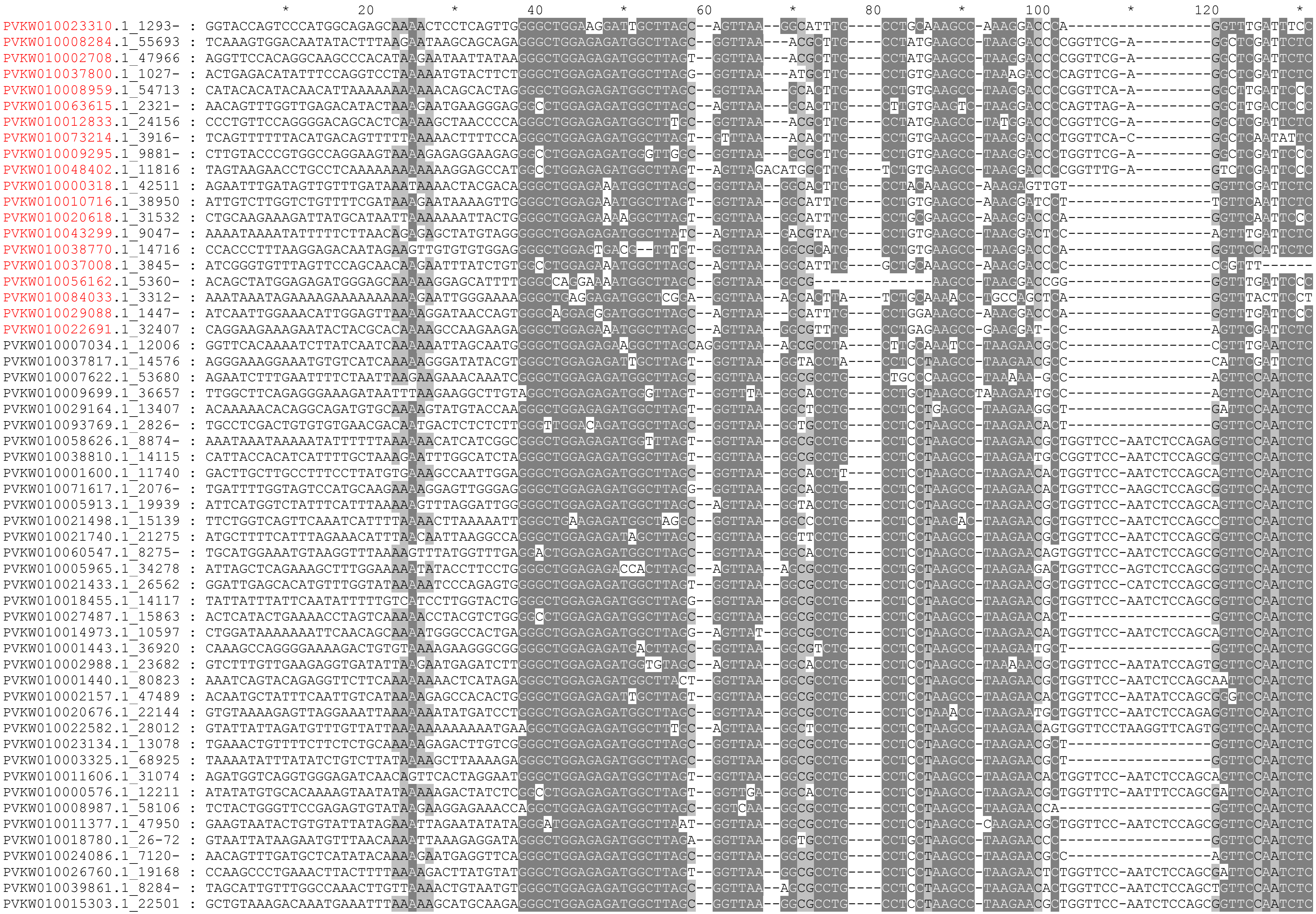

* 20 * 40 * 60 * 80 * 100 * 120 *
PVKW010023310.1_1293- : GGTACCAGTCCCATGGCAGAGCAAAACTCCTCAGTTGGGGCTGGAAGGATTGCTTAGC--AGTTAA--GGCATTTG----CCTGCAAAGCC-AAAGGACCCA-----------------GGTTTGATTTCC
PVKW010008284.1_55693 : TCAAAGTGGACAATATACTTTAAGAATAAGCAGCAGAGGGCTGGAGAGATGGCTTAGC--GGTTAA---ACGCTTG----CCTATGAAGCC-TAAGGACCCCGGTTCG-A---------GGCTCGATTCTC
PVKW010002708.1_47966 : AGGTTCCACAGGCAAGCCCACATAAGAATAATTATAAGGGCTGGAGAGATGGCTTAGT--GGTTAA---ACGCTTG----CCTATGAAGCC-TAAGGACCCCGGTTCG-A---------GGCTCGATTCTC
PVKW010037800.1_1027- : ACTGAGACATATTTCCAGGTCCTAAAAATGTACTTCTGGGCTGGAGAGATGGCTTAGG--GGTTAA---ATGCTTG----CCTGTGAAGCC-TAAAGACCCCAGTTCG-A---------GGCTCGATTCTC
PVKW010008959.1_54713 : CATACACATACAACATTAAAAAAAAAAACAGCACTAGGGGCTGGAGAGATGGCTTAGC--GGTTAA---GCACTTG----CCTGTGAAGCC-TAAGGACCCCGGTTCA-A---------GGCTTGATTCCC
PVKW010063615.1_2321- : AACAGTTTGGTTGAGACATACTAAAGAATGAAGGGAGGGCCTGGAGAGATGGCTTAGC--AGTTAA---GCACTTG----CTTGTGAAGTC-TAAGGACCCCAGTTAG-A---------GGCTTGACTCCC
PVKW010012833.1_24156 : CCCTGTTCCAGGGGACAGCACTCAAAAAGCTAACCCCAGGGCTGGAGAGATGGCTTTGC--GGTTAA---ACGCTTG----CCTATGAAGCC-TATGGACCCCGGTTCG-A---------GGCTCGATTCTC
PVKW010073214.1_3916- : TCAGTTTTTTACATGACAGTTTTTAAAAACTTTTCCAGGGCTGGAGAGATGGCTTAGT--GTTTAA---ACACTTG----CCTGTGAAGCC-TAAGGACCCTGGTTCA-C---------GGCTCAATATTC
PVKW010009295.1_9881- : CTTGTACCCGTGGCCAGGAAGTAAAAGAGAGGAAGAGGGCCTGGAGAGATGGGTTGGC--GGTTAA---GCGCTTG----CCTGTGAAGCC-TAAGGACCCTGGTTCG-A---------GGCTCGATTCCC
PVKW010048402.1_11816 : TAGTAAGAACCTGCCTCAAAAAAAAAAAAGGAGCCATGGCCTGGAGAGATGGCTTAGT--AGTTAGACATGGCTTG----TCTGTGAAGCC-TAAGGACCCCGGTTTG-A---------GTCTCGATTCCC
PVKW010000318.1_42511 : AGAATTTGATAGTTGTTTGATAAATAAAACTACGACAGGGCTGGAGAAATGGCTTAGC--GGTTAA--GGCACTTG----CCTACAAAGCC-AAAGAGTTGT-----------------GGTTCGATTCTC
PVKW010010716.1_38950 : ATTGTCTTGGTCTGTTTTCGATAAAGAATAAAAGTTGGGGCTGGAGAAATGGCTTAGT--GGTTAA--GGCATTTG----CCTGTGAAGCC-AAAGGATCCT-----------------TGTTCAATTCTC
PVKW010020618.1_31532 : CTGCAAGAAAGATTATGCATAATTAAAAAAATTACTGGGGCTGGAGAAAAGGCTTAGT--GGTTAA--GGCATTTG----CCTGCGAAGCC-AAAGGACCCA-----------------GGTTCAATTCCC
PVKW010043299.1_9047- : AAAATAAAATATTTTTCTTAACAGAGAGCTATGTAGGGGGCTGGAGAGATGGCTTATC--AGTTAA--GACGTATG----CCTGTGAAGCC-TAAGGACTCC-----------------AGTTTGATTCTC
PVKW010038770.1_14716 : CCACCCCTTTAAGGAGACAATAGAAGTTGTGTGTGGAGGGGCTGGAGTGACG--TTTGT--GGTTAA--GGCGCATG----CCTGTGAAGCC-TAAGGACCCA-----------------GGTTCCATTCTC
PVKW010037008.1_3845- : ATCGGGTGTTTAGTTCCAGCAACAAGAATTTATCTGTGGCCTGGAGAAATGGCTTAGC--AGTTAA--GGCATTTG----GCTGCAAAGCC-AAAGGACCCC-----------------CGGTTT------
PVKW010056162.1_5360- : ACAGCTATGGAGAGATGGAGCAAAAAAGGAGCATTTTGGGCCAGGAAAATGGCTTAGC--GGTTAA--GGCG--------------AAGCC-TAAGGACCGG-----------------GGTTTGATTCCC
PVKW010084033.1_3312- : AAATAAATAGAAAAGAAAAAAAAAAGAATTGGGAAAAGGGCTGAGGAGATGGCTCGGA--GGTTAA--AGCACTTA----TCTGCAAAACC-TGCCAGCTCA-----------------GGTTTACTTCCT
PVKW010029088.1_1447- : ATCAATTGGAAACATTGGAGTTAAAAGGATAACCAGTGGGCAGGAGGGATGGCTTAGC--AGTTAA---GCATTTG----CCTGGAAAGCC-AAAGGACCCA-----------------GGTTTGATTCCC
PVKW010022691.1_32407 : CAGGAAGAAAGAATACTACGCACAAAAAGCCAAGAAGAGGGCTGGAGAAATGGCTTAGC--AGTTAA--GGCGTTTG----CCTGAGAAGCC-GAAGGAT-CC-----------------AGTTCGATTCTC
PVKW010007034.1_12006 : GGTTCACAAAATCTTATCAATCAAAAAATTAGCAATGGGGCTGGAGAGAAGGCTTAGCAGGGTTAA--AGCGCCTA----CTTGCAAATCC-TAAGAACGCC-----------------CGTTTGAATCTC
PVKW010037817.1_14576 : AGGGAAAGGAAATGTGTCATCAAAAAGGGATATACGTGGGCTGGAGAGATTGCTTAGT--GGTTAA--GGTACCTA----CCTCCTAAGCC-TAAGAACGCC-----------------CATTCGATTCTC
PVKW010007622.1_53680 : AGAATCTTTGAATTTTCTAATTAAGAAGAAACAAATCGGGCTGGAGAGATGGCTTAGC--GGTTAA--GGCGCCTG----CTGCCCAAGCC-TAAAAA-GCC-----------------AGTTCCAATCTC
PVKW010009699.1_36657 : TTGGCTTCAGAGGGAAAGATAATTTAAGAAGGCTTGTAGGCTGGAGAGATGGGTTAGT--GGTTTA--GGCACCTG----CCTGTTAAGCCTAAGAATGCC-----------------AGTTCCAATCTC
PVKW010029164.1_13407 : ACAAAAACACAGGCAGATGTGCAAAAAGTATGTACCAAGGGCTGGAGAGATGGCTTAGT--GGTTAA--GGCTCCTG----CCTCCTGAGCC-TAAGAAGGCT-----------------GATTCCAATCTC
PVKW010093769.1_2826- : TGCCTCGACTGTGTGTGAACGACAATGACTCTCTCTTGGGTTGGACAGATGGCTTAGC--GGTTAA--GGTGCCTG----CCTCCTAAGCC-TAAGAACACT-----------------GGTTCCAATCTC
PVKW010058626.1_8874- : AAATAAATAAAAATATTTTTTAAAAAACATCATCGGCGGGCTGGAGAGATGGTTTAGT--GGTTAA--GGCGCCTG----CCTCCTAAGCC-TAAGAACGCTGGTTCC-AATCTCCAGAGGTTCCAATCTC
PVKW010038810.1_14115 : CATTACCACATCATTTTGCTAAAGAATTTGGCATCTAGGGCTGGAGAGATGGCTTAGT--GGTTAA--GGCGCCTG----CCTCCTAAGCC-TAAGAATGCCGGTTCC-AATCTCCAGCGGTTCCAATCTC
PVKW010001600.1_11740 : GACTTGCTTGCCTTTCCTTATGTGAAAGCCAATTGGAGGGCTGGAGAGATGGCTTAGC--GGTTAA--GGCACCTT----CCTCCTAAGCC-TAAGAACACTGGTTCC-AATCTCCAGCAGTTCCAATCTC
PVKW010071617.1_2076- : TGATTTTGGTAGTCCATGCAAGAAAAGGAGTTGGGAGGGGCTGGAGAGATGGCTTAGG--GGTTAA--GGCACCTG----CCTCCTAAGCC-TAAGAACACTGGTTCC-AAGCTCCAGCGGTTCCAATCTC
PVKW010005913.1_19939 : ATTCATGGTCTATTTCATTTAAAAAGTTTAGGATTGGGGGCTGGAGAGATGGCTTAGC--AGTTAA--GGTACCTG----CCTCCTAAGCC-TAAGAACGCTGGTTCC-AATCTCCAGCAGTTCCAATCTC
PVKW010021498.1_15139 : TTCTGGTCAGTTCAAATCATTTTAAAACTTAAAAATTGGGCTGAAGAGATGGCTAGGC--GGTTAA--GGCCCCTG----CCTCCTAAGAC-TAAGAACGCTGGTTCC-AATCTCCAGCCGTTCCAATCTC
PVKW010021740.1_21275 : ATGCTTTTCATTTAGAAACATTTAACAATTAAGGCCAGGGCTGGAGAGATAGCTTAGC--GGTTAA--GGTTCCTG----CCTCCTAAGCC-TAAGAACGCTGGTTCC-AATCTCCAGCGGTTCCAATCTC
PVKW010060547.1_8275- : TGCATGGAAATGTAAGGTTTAAAAGTTTATGGTTGAGGACTGGAGAGATGGCTTAGC--GGTTAA--GGCACCTG----CCTCCTAAGCC-TAAGAACAGTGGTTCC-AATCTCCAGCGGTTCCAATCTC
PVKW010005965.1_34278 : ATTAGCTCAGAAAGCTTTGGAAAAATATACCTTCCTGGGGCTGGAGAGACCACTTAGC--AGTTAA--AGCGCCTG----CCTGCTAAGCC-TAAGAAGACTGGTTCC-AGTCTCCAGCGGTTCCAATCTC
PVKW010021433.1_26562 : GGATTGAGCACATGTTTGGTATAAAAATCCCAGAGTGGGGCTGGAGAGATGGCTTAGT--GGTTAA--GGCGCCTG----CCTCCTAAGCC-TAAGAACGCTGGTTCC-CATCTCCAGCGGTTCCAATCTC
PVKW010018455.1_14117 : TATTATTTATTCAATATTTTTGTCATCCTTGGTACTGGGGCTGGAGAGATGGCTTAGG--GGTTAA--GGCGCCTG----CCTCCTAAGCC-TAAGAACGCTGGTTCC-AATCTCCAGCGGTTCCAATCTC
PVKW010027487.1_15863 : ACTCATACTGAAAACCTAGTCAAAAAACCTACGTCTGGGGCCTGGAGAGATGGCTTAGC--GGTTAA--GGCGCCTG----CCTCCTAAGCC-TAAGAACACT-----------------GGTTCCAATCTC
PVKW010014973.1_10597 : CTGGATAAAAAAATTCAACAGCAAAAATGGGCCACTGAGGGCTGGAGAGATGGCTTAGG--AGTTAT--GGCGCCTG----CCTCCTAAGCC-TAAGAACACTGGTTCC-AATCTCCAGCAGTTCCAATCTC
PVKW010001443.1_36920 : CAAAGCCAGGGGAAAAGACTGTGTAAAAGAAGGGCGGGGGCTGGAGAGATGACTTAGC--GGTTAA--GGCGTCTG----CCTCCTAAGCC-TAAGAATGCT-----------------GGTTCCAATCTC
PVKW010002988.1_23682 : GTCTTTGTTGAAGAGGTGATATTAAGAATGAGATCTTGGGCTGGAGAGATGGCTTGTAGC--AGTTAA--GGCACCTG----CCTCCTAAGCC-TAAAAACGCTGGTTCC-AATATCCAGTGGTTCCAATCTC
PVKW010001440.1_80823 : AAATCAGTACAGAGGTTCTTCAAAAAAAAACTCATAGAGGGCTGGAGAGATGGCTTAGCT--GGTTAA--GGCGCCTG----CCTCCTAAGCC-TAAGAACGCTGGTTCC-AATCTCCAGCAATTCCAATCTC
PVKW010002157.1_47489 : ACAATGCTATTTCAATTGTCATAAAAAGAGCCACACTGGGGCTGGAGAGATTGCTTAGT--GGTTAA--GGCGCCTG----CCTCCTAAGCC-TAAGAACACTGGTTCC-AATATCCAGCGGGTCCAATCTC
PVKW010020676.1_22144 : GTGTAAAAGAGTTAGGAAATTAAAAAAATATGATCCTGGGCTGGAGAGATGGCTTAGC--GGTTAA--GGCGCCTG----CCTCCTAAACC-TAAGAATGCTGGTTCC-AATCTCCAGAGGTTCCAATCTC
PVKW010022582.1_28012 : GTATTATTAGATGTTTGTTATTAAAAAAAAAAAATGAAGGCTGGAGAGATGGCTTTGC--AGTTAA--GGCTCCTG----CCTCCTAAGCC-TAAGAACAGTGGTTCCTAAGGTTCAGTGGTTCCAATCTC
PVKW010023134.1_13078 : TGAAACTGTTTTCTTCTCTGCAAAAAGAGACTTGTCGGGGCTGGAGAGATGGCTTAGC--GGTTAA--GGCGCCTG----CCTCCTAAGCC-TAAGAACGCT-----------------GGTTCCAATCTC
PVKW010003325.1_68925 : TAAAATATTTATATCTGTCTTATAAAAGCTTAAAAGAGGGCTGGAGAGATGGCTTAGC--GGTTAA--GGCGCCTG----CCTCCTAAGCC-TAAGAACGCT-----------------GGTTCCAATCTC
PVKW010011606.1_31074 : AGATGGTCAGGTGGGAGATCAACAGTTCACTAGGAATGGGCTGGAGAGATGGCTTAGT--GGTTAA--GGCGCCTG----CCTCCTAAGCC-TAAGAACACTGGTTCC-AATCTCCAGCAGTTCCAATCTC
PVKW010000576.1_12211 : ATATATGTGCACAAAAGTAATATAAAAAGACTATCTCGGCCTGGAGAGATGGCTTAGT--GGTTGA--GGCACCTG----CCTCCTAAGCC-TAAGAACGCTGGTTTC-AATTTCCAGCGATTCCAATCTC
PVKW010008987.1_58106 : TCTACTGGGTTCCGAGAGTGTATAAGAAGGAGAAACCAGGCTGGAGAGATGGCTTAGC--GGTCAA--GGCGCCTG----CCTCCTAAGCC-TAAGAACCA-----------------GGTTCCAATCTC
PVKW010011377.1_47950 : GAAGTAATACTGTGTATTATAGAAAATTAGAATATATAGGGATGGAGAGATGGCTTAAT--GGTTAA--GGCGCCTG----CCTCCTAAGCC-CAAGAACGCTGGTTCC-AATCTCCAGCGGTTCCAATCTC
PVKW010018780.1_26-72 : GTAATTATAAGAATGTTTAACAAAATTAAAGAGGATAGGGCTGGAGAGATGGCTTAGA--GGTTAA--GGTGCCTG----CCTGCTAAGCC-TAAGAACCC------------------GGTTCCAATCTC
PVKW010024086.1_7120- : AACAGTTTGATGCTCATATACAAAAGAATGAGGTTCAGGGCTGGAGAGATGGCTTAGC--GGTTAA--GGCGCCTG----CCTCCTAAGCC-TAAGAACGCC-----------------AGTTCCAATCTC
PVKW010026760.1_19168 : CCAAGCCCTGAAACTTACTTTTAAAAGACTTATGTATGGGCTGGAGAGATGGCTTAGT--GGTTAA--GGCGCCTG----CCTCCTAAGCC-TAAGAACTCTGGTTCC-AATCTCCAGCGATTCCAATCTC
PVKW010039861.1_8284- : TAGCATTGTTTGGCCAAACTTGTTAAAACTGTAATGTGGGCTGGAGAGATGGCTTAGC--GGTTAA--AGCGCCTG----CCTCCTAAGCC-TAAGAACACTGGTTCC-AATCTCCAGCTGTTCCAATCTC
PVKW010015303.1_22501 : GCTGTAAAGACAAATGAAATTTAAAAAGCATGCAAGAGGGCTGGAGAGATGGCTTAGC--GGTTAA--GGCGCCTG----CCTCCTAAGCC-TAAGAACGCTGGTTCC-AATCTCCAGCGGTTCCAATCTC

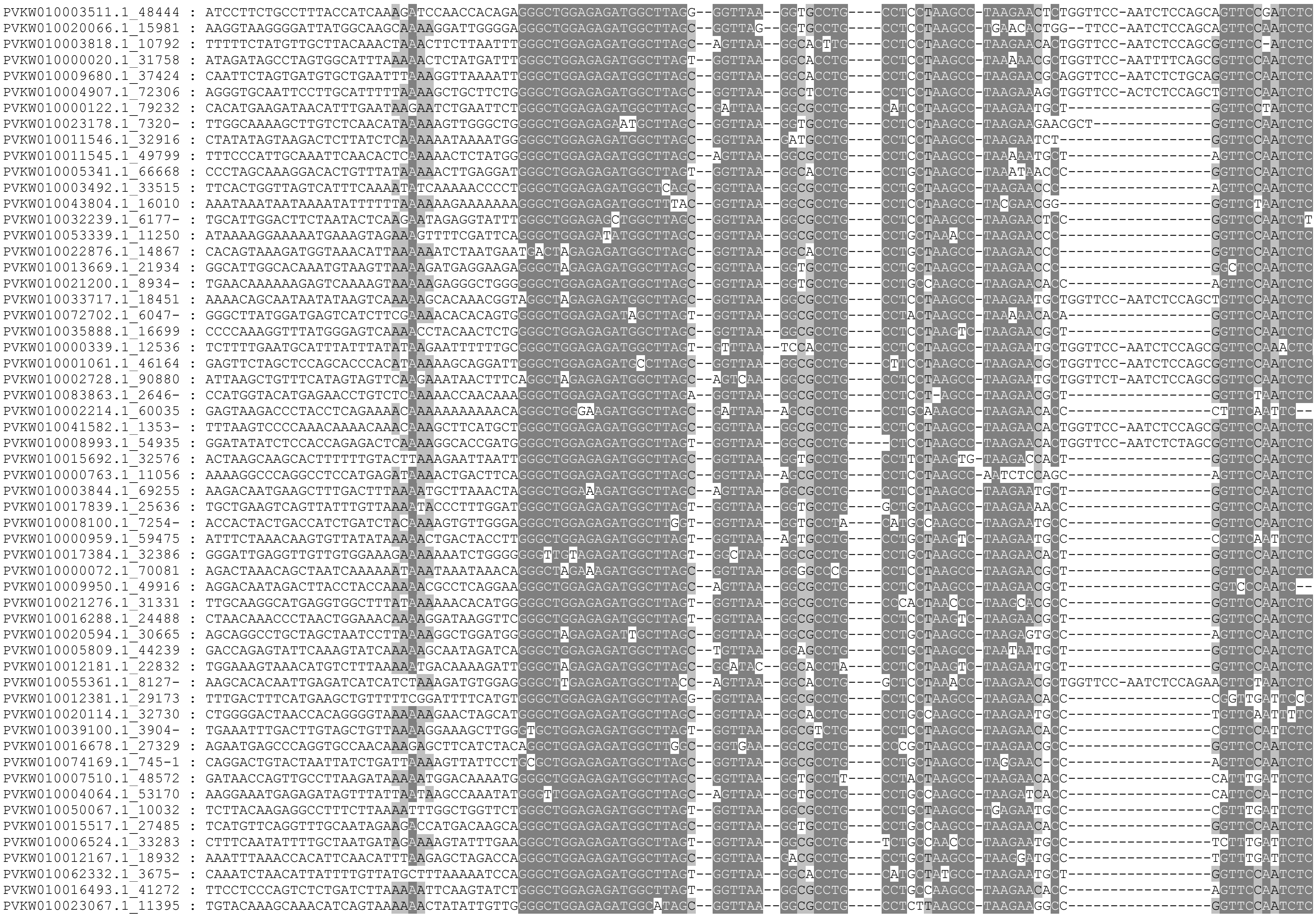
PVKW010003511.1_48444 : ATCCTTCTGCCTTTACCATCAAAGATCCAACCACAGAGGGCTGGAGAGATGGCTTAGG--GGTTAA--GGTGCCTG----CCTCCTAAGCC-TAAGAACTCTGGTTCC-AATCTCCAGCAGTTCCGATCTC
PVKW010020066.1_15981 : AAGGTAAGGGGATTATGGCAAGCAAAAGGATTGGGGAGGGCTGGAGAGATGGCTTAGC--GGTTAG--GGTGCCTG----CCTCCTAAGCC-TGAACACTGG--TTCC-AATCTCCAGCAGTTCCAATCTC
PVKW010003818.1_10792 : TTTTTCTATGTTGCTTACAAACTAAACTTCTTAATTTGGGCTGGAGAGATGGCTTAGC--AGTTAA--GGCACTTG----CCTCCTAAGCC-TAAGAACACTGGTTCC-AATCTCCAGCGGTTCC-ATCTC
PVKW010000020.1_31758 : ATAGATAGCCTAGTGGCATTTAAAAACTCTATGATTTGGGCTGGAGAGATGGCTTAGT--GGTTAA--GGCACCTG----CCTCCTAAGCC-TAAAAACGCTGGTTCC-AATTTTCAGCGGTTCCAATCTC
PVKW010009680.1_37424 : CAATTCTAGTGATGTGCTGAATTTAAAGGTTAAAATTGGGCTGGAGAGATGGCTTAGC--GGTTAA--GGCACCTG----CCTCCTAAGCC-TAAGAACGCAGGTTCC-AATCTCTGCAGGTTCCAATCTC
PVKW010004907.1_72306 : AGGGTGCAATTCCTTGCATTTTTAAAAGCTGCTTCTGGGCTGGAGAGATGGCTTAGC--GGTTAA--GGCTCCTG----CCTCCTAAGCC-TAAGAAAGCTGGTTCC-ACTCTCCAGCTGTTCCAATCTC
PVKW010000122.1_79232 : CACATGAAGATAACATTTGAATAAGAATCTGAATTCTGGGCTGGAGAGATGGCTTAGC--GATTAA--GGCGCCTG----CATCCTAAGCC-TAAGAATGCT-----------------GGTTCCAATCTC
PVKW010023178.1_7320- : TTGGCAAAAGCTTGTCTCAACATAAAAAGTTGGGCTGGGGCTGGAGAGAATGCTTAGC--GGTTAA--GGTGCCTG----CCTCCTAAGCC-TAAGAAGAACGCT--------------GGTTCCTATCTC
PVKW010011546.1_32916 : CTATATAGTAAGACTCTTATCTCAAAAAATAAAATGGGGGCTGGAGAGATGGCTTAGC--GGTTAA--GATGCCTG----CCTCCTAAGCC-TAAGAATCT-----------------GGTTCCAATCTC
PVKW010011545.1_49799 : TTTCCCATTGCAAATTCAACACTCAAAAACTCTATGGGGGCTGGAGAGATGGCTTAGC--AGTTAA--GGCGCCTG----CCTCCTAAGCC-TAAAAATGCT-----------------AGTTCCAATCTC
PVKW010005341.1_66668 : CCCTAGCAAAGGACACTGTTTATAAAAACTTGAGGATGGGCTGGAGAGATGGCTTAGT--GGTTAA--GGCACCTG----CCTGCTAAGCC-TAAATAACCC-----------------GGTTCCAATCTC
PVKW010003492.1_33515 : TTCACTGGTTAGTCATTTCAAAATATCAAAAACCCCTGGGCTGGAGAGATGGCTCAGC--GGTTAA--GGCGCCTG----CCTGCTAAGCC-TAAGAACCC------------------AGTTCCAATCTC
PVKW010043804.1_16010 : AAATAAATAATAAAATATTTTTTAAAAAAGAAAAAAAGGGCTGGAGAGATGGCTTTAC--GGTTAA--GGCGCCTG----CCTCCTAAGCC-TACGAACGG------------------GGTTCTAATCTC
PVKW010032239.1_6177- : TGCATTGGACTTCTAATACTCAAGAATAGAGGTATTTGGGCTGGAGAGCTGGCTTAGC--GGTTAA--GGCGCCTG----CCTCCTAAGCC-TAAGAACTCC-----------------GGTTCCAATCTT
PVKW010053339.1_11250 : ATAAAAGGAAAAATGAAAGTAGAAAGTTTTCGATTCAGGGCTGGAGATATGGCTTAGC--GGTTAA--GGCGCCTG----CCTGCTAAACC-TAAGAACCC------------------GGTTCCAATCTC
PVKW010022876.1_14867 : CACAGTAAAGATGGTAAACATTAAAAAATCTAATGAATGACTAGAGAGATGGCTTAGC--GGTTAA--GGCACCTG----CCTGCTAAGCC-TAAGAACCC------------------GGTTCCAATCTC
PVKW010013669.1_21934 : GGCATTGGCACAAATGTAAGTTAAAAGATGAGGAAGAGGGCTAGAGAGATGGCTTAGC--GGTTAA--GGTGCCTG----CCTGCTAAGCC-TAAGAACCC------------------GGCTCCAATCTC
PVKW010021200.1_8934- : TGAACAAAAAAGAGTCAAAAGTAAAAAGAGGGCTGGGGGCTGGAGAGATGGCTTAGC--GGTTAA--GGTGCCTG----CCTGCCAAGCC-TAAGAACACC-----------------AGTTCCAATCTC
PVKW010033717.1_18451 : AAAACAGCAATAATATAAGTCAAAAAGCACAAACGGTAGGCTAGAGAGATGGCTTAGC--GGTTAA--GGCGCCTG----CCTCCTAAGCC-TAAGAATGCTGGTTCC-AATCTCCAGCTGTTCCAATCTC
PVKW010072702.1_6047- : GGGCTTATGGATGAGTCATCTTCGAAAACACACAGTGGGGCTGGAGAGATAGCTTAGT--GGTTAA--GGCGCCTG----CCTACTAAGCC-TAAAAACACA-----------------GGTTCCAATCTC
PVKW010035888.1_16699 : CCCCAAAGGTTTATGGGAGTCAAAACCTACAACTCTGGGGCTGGAGAGATGGCTTAGC--GGTTAA--GGCGCCTG----CCTCCTAAGTC-TAAGAACGCT-----------------GGTTCCAATCTC
PVKW010000339.1_12536 : TCTTTTGAATGCATTTATTTATATAAGAATTTTTTGCGGGCTGGAGAGATGGCTTAGT--GTTTAA--TCCACCTG----CCTCCTAAGCC-TAAGAATGCTGGTTCC-AATCTCCAGCGGTTCCAAACTC
PVKW010001061.1_46164 : GAGTTCTAGCTCCAGCACCCACATAAAAAGCAGGATTGGGCTGGAGAGATGCCTTAGC--GGTTAA--GGCGCCTG----CTTCCTAAGCC-TAAGAACGCTGGTTCC-AATCTCCAGCGGTTCCAATCTC
PVKW010002728.1_90880 : ATTAAGCTGTTTCATAGTAGTTCAAGAAATAACTTTCAGGCTAGAGAGATGGCTTAGC--AGTCAA--GGCGCCTG----CCTCCTAAGCC-TAAGAATGCTGGTTCT-AATCTCCAGCGGTTCCAATCTC
PVKW010083863.1_2646- : CCATGGTACATGAGAACCTGTCTCAAAAACCAACAAAGGGCTGGAGAGATGGCTTAGA--GGTTAA--GGCGCCTG----CCTCCT-AGCC-TAAGAACGCT-----------------GGTTCTAATCTC
PVKW010002214.1_60035 : GAGTAAGACCCTACCTCAGAAAACAAAAAAAAAAAACAGGGCTGGGAAGATGGCTTAGC--GATTAA--AGCGCCTG----CCTGCAAAGCC-TAAGAACACC-----------------CTTTCAATTC--
PVKW010041582.1_1353- : TTTAAGTCCCCAAACAAAACAAACAAAGCTTCATGCTGGGCTGGAGAGATGGCTTAGC--GGTTAA--GGCGCCTG----CCTCCTAAGCC-TAAGAACACTGGTTCC-AATCTCCAGCGGTTCCAATCTC
PVKW010008993.1_54935 : GGATATATCTCCACCAGAGACTCAAAAGGCACCGATGGGGCTGGAGAGATGGCTTAGT--GGTTAA--GGCGCCTG-----CTCCTAAGCC-TAAGAACACTGGTTCC-AATCTCTAGCGGTTCCAATCTC
PVKW010015692.1_32576 : ACTAAGCAAGCACTTTTTTGTACTTAAAGAATTAATTGGGCTGGAGAGATGGCTTAGC--GGTTAA--GGTGCCTG----CCTTCTAAGTG-TAAGACCACT-----------------GGTTCCAATCTC
PVKW010000763.1_11056 : AAAAGGCCCAGGCCTCCATGAGATAAAACTGACTTCAGGGCTGGAGAGATGGCTTAGC--GGTTAA--AGCGCCTG----CCTCCTAAGCC-AATCTCCAGC-----------------AGTTCCAATCTC
PVKW010003844.1_69255 : AAGACAATGAAGCTTTGACTTTAAAATGCTTAAACTAGGGCTGGAAAGATGGCTTAGC--AGTTAA--GGCGCCTG----CCTCCTAAGCC-TAAGAATGCT-----------------GGTTCCAATCTC
PVKW010017839.1_25636 : TGCTGAAGTCAGTTATTTGTTAAAATACCCTTTGGATGGGCTGGAGAGATGGCTTAGT--GGTTAA--GGTGCCTG----GCTGCTAAGCC-TAAGAAAACC-----------------GGTTCCAATCTC
PVKW010008100.1_7254- : ACCACTACTGACCATCTGATCTACAAAAGTGTTGGGAGGGCTGGAGAGATGGCTTGGT--GGTTAA--GGTGCCTA----CATGCCAAGCC-TAAGAATGCC-----------------GGTTCCAATCTC
PVKW010000959.1_59475 : ATTTCTAAACAAGTGTTATATAAAAACTGACTACCTTGGGCTGGAGAGATGGCTTAGT--GGTTAA--AGTGCCTG----CCTGCTAAGTC-TAAGAATGCC-----------------CGTTCAATTCTC
PVKW010017384.1_32386 : GGGATTGAGGTTGTTGTGGAAAGAAAAAAATCTGGGGGGTTGTAGAGATGGCTTAGT--GGCTAA--GGCGCCTG----CCTGCTAAGCC-TAAGAACACT-----------------GGTTCCAATCTC
PVKW010000072.1_70081 : AGACTAAACAGCTAATCAAAAAATAAATAAATAAACAGGGCTAGAAAGATGGCTTAGC--GGTTAA--GGGGCCCG----CCTCCTAAGCC-TAAGAACGCT-----------------GGTTCCAATCTC
PVKW010009950.1_49916 : AGGACAATAGACTTACCTACCAAAAACGCCTCAGGAAGGGCTGGAGAGATGGCTTAGC--AGTTAA--GGCGCCTG----CCTCCTAAGCC-TAAGAACGCT-----------------GGTCCCAATC--
PVKW010021276.1_31331 : TTGCAAGGCATGAGGTGGCTTTATAAAAAACACATGGGGGCTGGAGAGATGGCTTAGT--GGTTAA--GGCGCCTG----CCCACTAACCC-TAAGCACGCC-----------------GGTTCCAATCTC
PVKW010016288.1_24488 : CTAACAAACCCTAACTGGAAACAAAAGGATAAGGTTCGGGCTGGAGAGATGGCTTAGT--GGTTAA--GGCGCCTG----CCTCCTAAGTC-TAAGAACGCT-----------------GGTTCCAATCTC
PVKW010020594.1_30665 : AGCAGGCCTGCTAGCTAATCCTTAAAAGGCTGGATGGGGGCTAGAGAGATTGCTTAGC--GGTTAA--GGCGCCTG----CCTGCTAAGCC-TAAGAGTGCC-----------------AGTTCCAATCTC
PVKW010005809.1_44239 : GACCAGAGTATTCAAAGTATCAAAAAGCAATAGATCAGGGCTGGAGAGATGGCTTAGC--TGTTAA--GGAGCCTG----CCTGCTAAGCC-TAATAATGCT-----------------GGTTCCAATCTC
PVKW010012181.1_22832 : TGGAAAGTAAACATGTCTTTAAAAATGACAAAAGATTGGGCTAGAGAGATGGCTTAGC--GGATAC--GGCACCTA----CCTCCTAAGTC-TAAGAATGCT-----------------GGTTCCAATCTC
PVKW010055361.1_8127- : AAGCACACAATTGAGATCATCATCTAAAGATGTGGAGGGGCTTGAGAGATGGCTTACC--AGTTAA--GGCACCTG----GCTCCTAAACC-TAAGAACGCTGGTTCC-AATCTCCAGAAGTTCTAATCTC
PVKW010012381.1_29173 : TTTGACTTTCATGAAGCTGTTTTTCGGATTTTCATGTGGGCTGGAGAGATGGCTTAGG--GGTTAA--GGCGCCTG----CCTCCTAAGCC-TAAGAACACC-----------------CGGTTGATTCCC
PVKW010020114.1_32730 : CTGGGGACTAACCACAGGGGTAAAAAAGAACTAGCATGGGCTGGAGAGATGGCTTAGC--GGTTAA--GGCACCTG----CCTGCCAAGCC-TAAGAATGCC-----------------TGTTCAATTTTC
PVKW010039100.1_3904- : TGAAATTTGACTTGTAGCTGTTAAAAGGAAAGCTTGGGTGCTGGAGAGATGGCTTAGT--GGTTAA--GGCGTCTG----CCTCCTAAGCC-TAAGAACACC-----------------CGTTCCATTCTC
PVKW010016678.1_27329 : AGAATGAGCCCAGGTGCCAACAAAGAGCTTCATCTACAGGCTGGAGAGATGGCTTGGC--GGTGAA--GGCGCCTG----CCCGCTAAGCC-TAAGAACGCC-----------------GGTTCCAATCTC
PVKW010074169.1_745-1 : CAGGACTGTACTAATTATCTGATTAAAAGTTATTCCTGCGCTGGAGAGATGGCTTAGC--GGTTAA--GGCGCCTG----CCTGCTAAGCC-TAGGAAC-CC-----------------AGTTCCAATCTC
PVKW010007510.1_48572 : GATAACCAGTTGCCTTAAGATAAAAATGGACAAAATGGGGCTGGAGAGATGGCTTAGC--GGTTAA--GGTGCCTT----CCTACTAAGCC-TAAGAACACC-----------------CATTTGATTCTC
PVKW010004064.1_53170 : AAGGAAATGAGAGATAGTTTATTAATAAGCCAAATATGGGTTGGAGAGATGGCTTAGC--AGTTAA--GGTGCCTG----CCTGCCAAGCC-TAAGATCACC-----------------CATTCCA-TCTC
PVKW010050067.1_10032 : TCTTACAAGAGGCCTTTCTTAAAAATTTGGCTGGTTCTGGGCTGGAGAGATGGCTTAGT--GGTTAA--GGCGCCTG----CCTGCTAAGCC-TGAGAATGCC-----------------CGTTTGATTCTC
PVKW010015517.1_27485 : TCATGTTCAGGTTTGCAATAGAAGACCATGACAAGCAGGGCTGGAGAGATGGCTTAGC--GGTTAA--GGTGCCTG----CCTGCCAAGCC-TAAGAACACC-----------------GGTTCCAATCTC
PVKW010006524.1_33283 : CTTTCAATATTTTGCTAATGATAGAAAAGTATTTGAAGGGCTGGAGAGATGGCTTAGT--GGTTAA--GGCGCCTG----TCTGCCAACCC-TAAGAATGCC-----------------TCTTTGATTCTC
PVKW010012167.1_18932 : AAATTTAAACCACATTCAACATTTAAGAGCTAGACCAGGGCTGGAGAGATGGCTTAGC--GGTTAA--GACGCCTG----CCTGCTAAGCC-TAAGGATGCC-----------------TGTTTGATTCTC
PVKW010062332.1_3675- : CAAATCTAACATTATTTTGTTATGCTTTAAAAATCCAGGGCTGGAGAGATGGCTTAGT--GGTTAA--GGCACCTG----CATGCTATGCC-TAAGAATGCT-----------------GGTTCCAATCTC
PVKW010016493.1_41272 : TTCCTCCCAGTCTCTGATCTTAAAAATTCAAGTATCTGGGCTGGAGAGATGGCTTAGT--GGTTAA--GGCGCCTG----CCTGCCAAGCC-TAAGAACACC-----------------AGTTCCAATCTC
PVKW010023067.1_11395 : TGTACAAAGCAAACATCAGTAAAAAACTATATTGTTGGGGCTGGAGAGATGGCATAGC--GGTTAA--GGCGCCTG----CCTCTTAAGCC-TAAGAAGGCC-----------------GGTTCCAATCTC

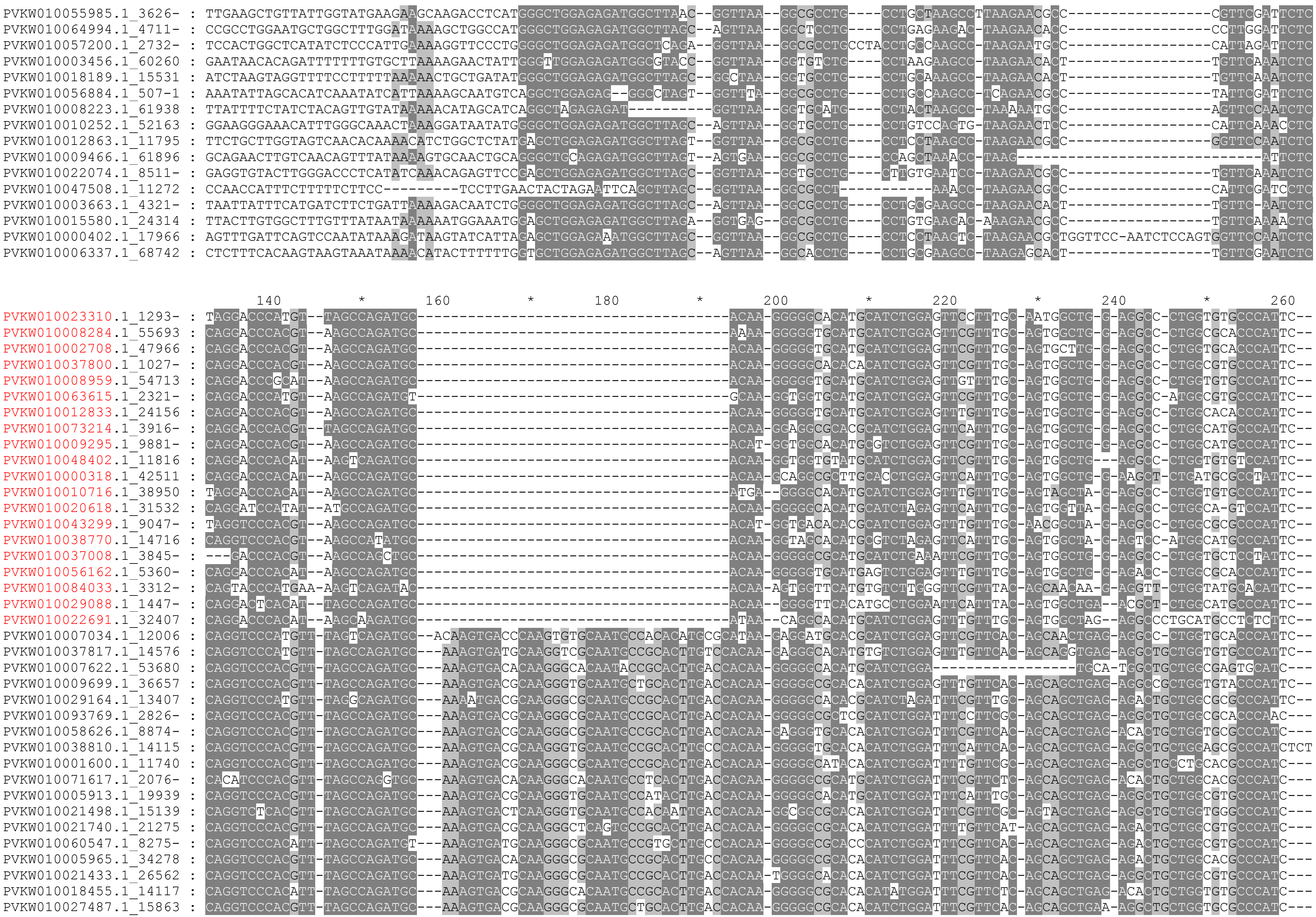
PVKW010055985.1_3626-
PVKW010064994.1_4711-
PVKW010057200.1_2732-
PVKW010003456.1_60260
PVKW010018189.1_15531
PVKW010056884.1_507-1
PVKW010008223.1_61938
PVKW010010252.1_52163
PVKW010012863.1_11795
PVKW010009466.1_61896
PVKW010022074.1_8511-
PVKW010047508.1_11272
PVKW010003663.1_4321-
PVKW010015580.1_24314
PVKW010000402.1_17966
PVKW010006337.1_68742
140
160
180
200
220
240
260
PVKW010023310.1_1293-
PVKW010008284.1_55693
PVKW010002708.1_47966
PVKW010037800.1_1027-
PVKW010008959.1_54713
PVKW010063615.1_2321-
PVKW010012833.1_24156
PVKW010073214.1_3916-
PVKW010009295.1_9881-
PVKW010048402.1_11816
PVKW010000318.1_42511
PVKW010010716.1_38950
PVKW010020618.1_31532
PVKW010043299.1_9047-
PVKW010038770.1_14716
PVKW010037008.1_3845-
PVKW010056162.1_5360-
PVKW010084033.1_3312-
PVKW010029088.1_1447-
PVKW010022691.1_32407
PVKW010007034.1_12006
PVKW010037817.1_14576
PVKW010007622.1_53680
PVKW010009699.1_36657
PVKW010029164.1_13407
PVKW010093769.1_2826-
PVKW010058626.1_8874-
PVKW010038810.1_14115
PVKW010001600.1_11740
PVKW010071617.1_2076-
PVKW010005913.1_19939
PVKW010021498.1_15139
PVKW010021740.1_21275
PVKW010060547.1_8275-
PVKW010005965.1_34278
PVKW010021433.1_26562
PVKW010018455.1_14117
PVKW010027487.1_15863

```
PVKW010014973.1_10597 : CAGGTCCCACGTT-TAGCCAGATGC---AAAGTGATGCAAGGACGCAAAGCCGCACTTGACCACAA-GGGGGTGCACACATCTGGATTTCGTTCAC-AGCAGCCGAG-ACACTGCTGGCACGCCCATC---
PVKW010001443.1_36920 : CAGGTCCCACGTT-TAGCCAGATGC---AAAGTGATGCAAGGGCACAATGCCGCACTTGACCACAA-GGGGGCGCACACATCTGGATTTCGTTCAC-AGCAGCTGAG-AGGCTGCTGGCGCACCCATC---
PVKW010002988.1_23682 : CAGGTCCCATATT-TATCCAGATGC---AAAGTGACCTAAGGGTGCAATGCCGCACTTGACCACAA-GGCAGCACACATCTGGATTTTGTTCTC-AGAAGCTGAG-AGGCTGCTGGCACGCCCATC---
PVKW010001440.1_80823 : CAGGTCCCACGTT-TAGCCAGATGC---AAAGTGATGCAAGGGCGCAATGCCGCACTTGACCACAA-GGGGGCGCACGCATCTGGATTTTGTTCAT-AGTAGCTGAG-AGGCTGCTGGTACGCCCATC---
PVKW010002157.1_47489 : CAGGTCCCACATT-TAGCCAGATGC---AAAGTGACGCAAGGGCGCAATGCCGCACTTGACCACAA-GGGGGCGCATGCATCTGGATTTTGTTCGC-AGCAGCTGAG-AGACTGCTGGTGCACCCATC---
PVKW010020676.1_22144 : CAGGTCCCACCGT-TAGCCAGATGC---AAAGTGATGCAAGGGCGCAATGCCACACTTGACCAGAA-GGGGGCGCACGCATCTGGATTTCATTCGC-AGCAGCTGAG-ACGCTGCTGGCAATCCCATC---
PVKW010022582.1_28012 : CAGGTCCCACGTT-TAGCCAGATGC---AAAGTGACGCAAGGGCGCAATGCCGCACTTGACCACAA-GGGGGCGCACGCATCTGGATTTCGTTCAC-AGCAGCTGAG-AGGCTGCTGGCGCGCCCATC---
PVKW010023134.1_13078 : CAGGTCCCACGTT-TAGCCAGATGC---AAAGTGACGCAAGGGTGCAATGCCTCATTTGACCACAA-GGGGGCGTACACATTTGGATTTCATTCGC-AGCAGCTGAG-AGGCTGCTGGCGCGCC-ATC---
PVKW010003325.1_68925 : CAGGTCCCTCGTT-TAGCCAGATGC-----AGTGACGCAAGGGCGTAATGCCGCACTTGACCACAA-GGGGGAGCACACATCTGGATTTCGTTCGT-AGCAGCTGAG-AGGCTGCTGGCACACCCATC---
PVKW010011606.1_31074 : CAGGTCCCACGTT-TAGCCAGATGC---AAAGTGATGCAAGGGCGCAATGCCGCACTTGACCACAA-GGGGGCACACACATCTGGATTTCATTCTC-AGCAGCTGAG-AGGCTGCTGGCGTGCCCATC---
PVKW010000576.1_12211 : CAGGTCCCACGTT-TAGCCAGATGC---AAAGTGACGCAAGGGCACAATGCCGCACTTAACCACAA-GGGAGCGAACATGTCTGGATTTCGTTCAC-AGCAGCTGAG-CCGCTGCTGGCACACCCATC---
PVKW010008987.1_58106 : CAGGTCCCACGTT-TAGCCAGATGC---AAAGTGACGCAAGGGTGCAATGCCGCACTTGCCCACAA-GGGGGCGCACACATCTGGATTTCGTTCAC-AGCAGCTGAG-AGACTGCTGGTGCGCCCATC---
PVKW010011377.1_47950 : CAGGTCCCACGTT-TAGCCAGATGC-----ACTGATGCAAGGGTGCAATGCCGCACTTGACCACAA-GGGGGCACACACATCTGGATTTCATTCAC-AGCAGCTGAG-AGGCTGCTGGCGTGCCCATC---
PVKW010018780.1_26-72 : CAGGTCCCACATT-TAGCCAGATGC---AAAGTGATGCAAGGGTGCAATGCCGCACTTGCCCACAT-GGGGGCGCACACATCTGGATTTCATTCGC-AGCAGCTGAG-AAACTGCTGGCATGCCCATTC--
PVKW010024086.1_7120- : CAGGTCCCACGGT-TAGCCAGATGC---AAAGTGACGCAAGGGCGCAATGCCGCACTTGACCACAA-GGGGGCGCACGCATCTGGATTTCGTTCAC-AGCAGCTGAG-AGGCTGCTGGCGTGCCCATC---
PVKW010026760.1_19168 : CAGGTCCCACGTT-TAGCCAGATGC---AAAGTGATGCAAGGGCACAATGCTGCACTTGACCACAA-GGTGGCGCATGCATCTAGATTTCATTCAC-AGCAGCTGAG-ACGCTGCTGGTGCACCCATC---
PVKW010039861.1_8284- : CAGGTCCCACGTT-TAGCCAGATGC---AAAGTGACACAAGGGCGCAATGCCGCACTTGACCACAA-GGGGACTCACGCATCTGGATTTTGTTCTC-AGCAGCTGAGAAGGCTGCTGGCGTGCCCATTC--
PVKW010015303.1_22501 : CAGGTTGAACGTT-TAGCCAGATGC---AAAGTGACGCAAGGGTGCAATGCTGCACTTGACCACAA-GGGGGCACACACATCTGGATTTCGTTCGC-AGCAGCTGAG-AGGCTGCTGGTGCGCCCATC---
PVKW010003511.1_48444 : CAGGTCCCACGTT-TAGCCAGATGC---AAAGTGACACAAGGGCACAATGCCGCACTTGACCACAA-GGGGGTGCATGCATCTGGATTTCGTTCTC-AGCAGCCAAG-AGACTGCTGGCGTGCCCATTC--
PVKW010020066.1_15981 : CAGGTCCCCCGTT-TAGCCAGATGC---AAAGTGATGCAAGGACGCAATGCTGCACTTGACCACAA-GGGGGCGCACACATCTGGATTTCGTTCGT-AGCAGCTAAG-AGGCTGCTGGCGTGCCCATC---
PVKW010003818.1_10792 : CAGGTCCCACGTT-TAGCCAGATGC---AAAGTGACGCAAGGGCACAATGCTGCACTTGACCACAA-GGGGGCGCACGCATCTAGATTACGTTCGC-AGCAGCTGAG-GGGCTGCTGGTGCCCCCATC---
PVKW010000020.1_31758 : CAGGTCCCACGTT-TAGCCAGATGC---AAAGTGATGCAAGGACACAATGCCACACTTGACCACAA-GGGGGCGCATGCATCTGGATTTCATTCAC-AGCAGCTGAG-AGGCTGCTGGTGCACCCATC---
PVKW010009680.1_37424 : CAGGTCCCACATT-TAGCCAGATGC---AAAGTGACGCAAGGGTGCAATGCCACACTTGACCACAA--GGGACGCATGCATCTGGATTTCATTCGT-AGCAGCTGAG-AGGCTGCTGGTGCGCCCATC---
PVKW010004907.1_72306 : CAGGTCCAATGTT-TAGCCAGATGC---AAAGTGAAGCAAGCACTCAATGCTGCACTTGACCACAA-GGGGGTGCATGCATCTGGATTTCGTTCTC-AGAGACTAAA-AGACTGCTGGCGCACCCATC---
PVKW010000122.1_79232 : CAGGTCCCACGTT-TAGCCAGATGC---AAAGTGACACAAGGGTGCAATGCTGCACTTGACCACAA-GAGGGCACACACATCTGGATTTCATTCAC-AGCAGCTGAG-ACACTGCTGGCGCGCCCATC---
PVKW010023178.1_7320- : CAGGTCCCACATT-TAGCCAGCTGC---AAAGTGATGCAAGGGCGCAATGCTGCACTTGACTACAA-GGGGGAGCATGCATCTAGATTTCGTTCAC-AGCAGCTGAG-AGGCTGCTGGC---CCCATC---
PVKW010011546.1_32916 : CAGGTCCCACGTT-TAGCCAAATGC---AAAGTGAAGCAAGGGCACAATGCCGCACTTGACCACAA-GGGGGCGCACACATCTGGATTTCGTTCAC-AGCAGCTGAG-AGACTGCTGGCGTGCTCATC---
PVKW010011545.1_49799 : CAGGTCCCACTTT-TAGCCAGATGC---AAAGTGACGCAAGGGCACAATGCCATACTTGACCACAA-GGGGGTGCACGCATCTGGATTTCGTTTGC-AGCAGCTGAG-CAACTGCTGGAATGCCCATC---
PVKW010005341.1_66668 : CAGGTCCCACGTT-TAGCCAGATGC---AAAGTGACACAAGGGCGCAATGCTGCACTTGACCACAA-GGGGGTGCACACATCTGGATTTCATTCAC-AGCAGCTGAG-AGACTGCTGGTGCGCCCATT---
PVKW010003492.1_33515 : CAGGTCCCACGTT-TAGCCAGATGC---AAAGGGACTCAAGGGCGCAATGCCACACTTGCCCACAA-GGGGGCGCATGCATCTGGATTTCATTCAC-AGCAGCTAAG-AGACTGTTGGCGCGCCCATTC--
PVKW010043804.1_16010 : CAGGTCCCACGTT-TAGCTAGATGC---AAAGTGACGCAAGGGCACAATGCCACACTTGCCCACAA-GGGGTTGCACACATCTGGATTTCGTTCAC-AGCAGCTGAG-AGACTGCTGGCACGCCCATTC--
PVKW010032239.1_6177- : CAAGTCCCACGT--TAGTCAGATGC---AAAGTGACGCAAGGGCGCAATGCCACACTTGACCACAA-GGGGGCGCAGACATCTGGATTTCGTTCAC-AGCAGCTGAG-AGACTGCTGGCGCGCCCATC---
PVKW010053339.1_11250 : CAGGTCCCACGTT-TAGCCAGATGC---AAAGTGACGCAAGGGCGCAATGCCGCACTTGCCCACAA-GGGGGTGCACACATCTGTATTTCGTTCAC-AGCAGCTGAG-AGACTGCTGGCGCGCCCATC---
PVKW010022876.1_14867 : CAGGTCCCACGTT-TAGCCAGATGC---AAAGTGACACAAGGGCGCAATGCCGCACTTGCCCACA--TGGGGCGCACGCATCTGGATTTTGTTCAC-AGCAGCTGAG-AGACTGCTGGTGCACCCATTC--
PVKW010013669.1_21934 : CAGGTCCCATGTT-TAGCCAGAAGC---AAAGTGATGCAAGGGTGCAATGCCGTACTTTCCCCCAA-GGGGGCACACACATCTGGACTTCGTTCAC-AGTAGCTGAG-AGACTACTGGCACACCCATTC--
PVKW010021200.1_8934- : CAGGTCCCACGTT-TAGCCAGACGC---AAAGTGACGCAAGGGCGCAATGCTGCACTTGGCCACAA-GGGGGCGCACGCATCTGGATTTCGTTCAC-AGCAGCTGAG-ACACTGCTGGTGTGCCCATTC--
PVKW010033717.1_18451 : CAGGTCCCATGTT-TAGCCAGATGC---AAAGTGACGCAAGGGCACAATGCCGCACTTGCCCACAA-GGGGGCGCACGCATCTGGATTTCGTTCAC-AGCAG-TGAG-AGGCTGCCCATCTCTATCTG---
PVKW010072702.1_6047- : CAGGTCCCACGTT-TCGCCAGATGC---AAAGTGATGCAAGGGCGCAATGCCACACTTGCCCACAA--GAGGCGCATGCATCTGGAATTCATTCAC-AGTGGCTGAG-AGGCTGCTGGTGCACCCATTC--
PVKW010035888.1_16699 : CAGGTCCCACGTT-TAGCCAGATGC---AAAGTGACGCAAGGGTGCAATGCTGCACTTGATCACAA-GGGGGTGCACACATCTGGATTTTGTTCGC-AGCAGCTGAG-ACGCTGCTGGAGCGCCCATC---
PVKW010000339.1_12536 : CAGGTCCCACGTT-TAGCCAGATGC---AAAATGACGCAAGGGCGTAATGCCGCACTTGACCATAA-GGGAGCACATGCATCTGGATTTTGTTTGC---CAGCTGAG-AGGCTGCTGGCACACCCATT---
PVKW010001061.1_46164 : CAGGTCCCACATT-TAGCCAGATGC---AAAGTGACGCAAGGGTGCAATGCCACACTTGACCACAT-GGGGACACACGCATCTGGATTTCATTCGC-AGCA---GAG-AGGCTGCTGGAGCACCCATTC--
PVKW010002728.1_90880 : CAGGTCCCACGTT-TAGCCAGATGC-----AGTGACACAAGGGTGCAATGCCGCACTTGACCACAA-GGGGGCGCATGCATCTGGATTTCGTTCGC-AGCAGCTAAG-AGGCTGCTGGTGTGCCCATC---
PVKW010083863.1_2646- : CAGGTCCCACGTT-TAGCCAGATGC-----AGTGACGCAAGGGCGCAATGTCGCACTTCACCACAA-GGGGGCGCACGCATCTGGATTTCGTTCCT-AGCAGCTGAG-AGACTGCTGGCCCGCCCATC---
PVKW010002214.1_60035 : ----TCCCACGTT-TAGCCAGAAACA-AAAAGTGATGCAAAGGTGCAATGCCACACTTGCCCATAA-GGGGATGCATGCATCTGGAGTTCGTTCAC-AGTGGCTGAG-AGGCCGCTGGCACGCCCATTC--
PVKW010041582.1_1353- : CAGGTCCCACGTT-TAGCCAGATGC---AAAGTGACACAAGGGTGCAATGCCGCACTTGACCACAA-GGGGGGGCAAGCATCTGGATTTCGTTCAC-AGCAGCTGAG-AGGCTGCTGGTGCACCCATC---
PVKW010008993.1_54935 : CAGGTCCCACATT-TAGCCAGATGC-----AGTGACACAAGGGCGCAATGCCACACTTGACCACAA-GGGGGCGCACGCATCTGGATTTCGTTCGC-AGCATCTGAG-AGACTGGTGGTGTGCCCATC---
PVKW010015692.1_32576 : CAGGTCCCACGTT-TAGCCAGATGC-----AGTGATGCAAGGGCGCAATGCCGCACTTGACCACAA-GGGGGCAAACATATCTGGATTTGGTTCTC-AGCAGCTGAG-AGGCTGCTGGCGCGCCCATC---
PVKW010000763.1_11056 : CAGGTCCCACGTT-TAGCCAGATGC---AAAG--------GGGTGCAATGCCGGACTTGACCACAA-GGGGGCGCACGCATCTGGATTTCGTTCAC-AGCAGCTGAG-AGGCTGCTGGCGTGCCCATC---
PVKW010003844.1_69255 : CAGGTCCCATGTT-TAGCCAGATGC-----AGTGACACAAGGGTGCAATGCCGCACTTAACCACAA-GGGGGCACACGCATCTGGGTTTCATTCGC-AGCAGCTGAG-AGGCCGCTGGCGCACCCATC---
PVKW010017839.1_25636 : CAGGTCCCACGTT-TAGCCAGATGC---AAAGTGACGCAAGGGCACAATGCTGCACTTGCCCACAA--GGGGCGCATGTGTCTGGAGTTCTTTCGC-AGCATCTGAG-AGGCTGCTGGTGCACCCATTT--
PVKW010008100.1_7254- : CAGGTCCCACGTT-TAGCCAGACGCA-AAAAGTGACACAAGGGTGCAATGCTGCACTTGCCCACAA-GAGGGCGCATGCGTCCGGAGTTCTTTCAC-AGCGGCTGAA-AGGCTGCTGACATGCCCATTC--
PVKW010000959.1_59475 : CATGTTCCACATT-TAGCCAGACAA-----AGTGACACAAGGGCACAATGCCACACTTGCCCACAA-GGGGGTGAATGCATCTGGAGTTCATTCAC-AGCAGCTGAG-AGGCTGTTGGCACACCTATTC--
PVKW010017384.1_32386 : TAGGTCCCATGTT-TAGCCAGATGC-----AGTGACACAAGGGCGCAATGCTGCACTTAACCACAA-GGGGGTGAATGCATCTGGAGTTCGTTCAC-AGCAGCTG------------GCACACCCATTC--
PVKW010000072.1_70081 : CAGGTCCCACATT-TAGCCAGATGC-----AGTGACGCATAGGCGCAATGCTGCACTTGACCACAA-GGGGGTGAACACATCTGGATTTCATTCGC-AGCAGCTGAG-AGGCTG-----GCACCCATC---
PVKW010009950.1_49916 : ----TCCCACATT-TAGCCAGATGC---AAAGTGACGCAAGGGTGCAATGCTGCACTTGACCACAA-GGGGGCGCACACATCTGGATTTCATTCGC-AGCAGCTGAG-AGGCTGCTGGCACGTCCATC---
PVKW010021276.1_31331 : CAAGTCCCACGTT-TAGCCAGATGC---AAAGTGACAAAAGCACGCAATGCTGCACGTGACCACAA-GGGGGCACATGCATCTGGATTTCATTCGC-TGCCGCTGAG-AGGCTGCTGGCTCGCCCATC---
PVKW010016288.1_24488 : CAGAACCCACATT-TAGCCAGATGC---AAAGTGACACAAGGGCGCAATGTCGCACTTGACCACAA-GGGGGCGCACACATGTGGATTTCGTTCAC-AGCAGCTGAG---ACTGCTGGCGCGCCCA-----
```

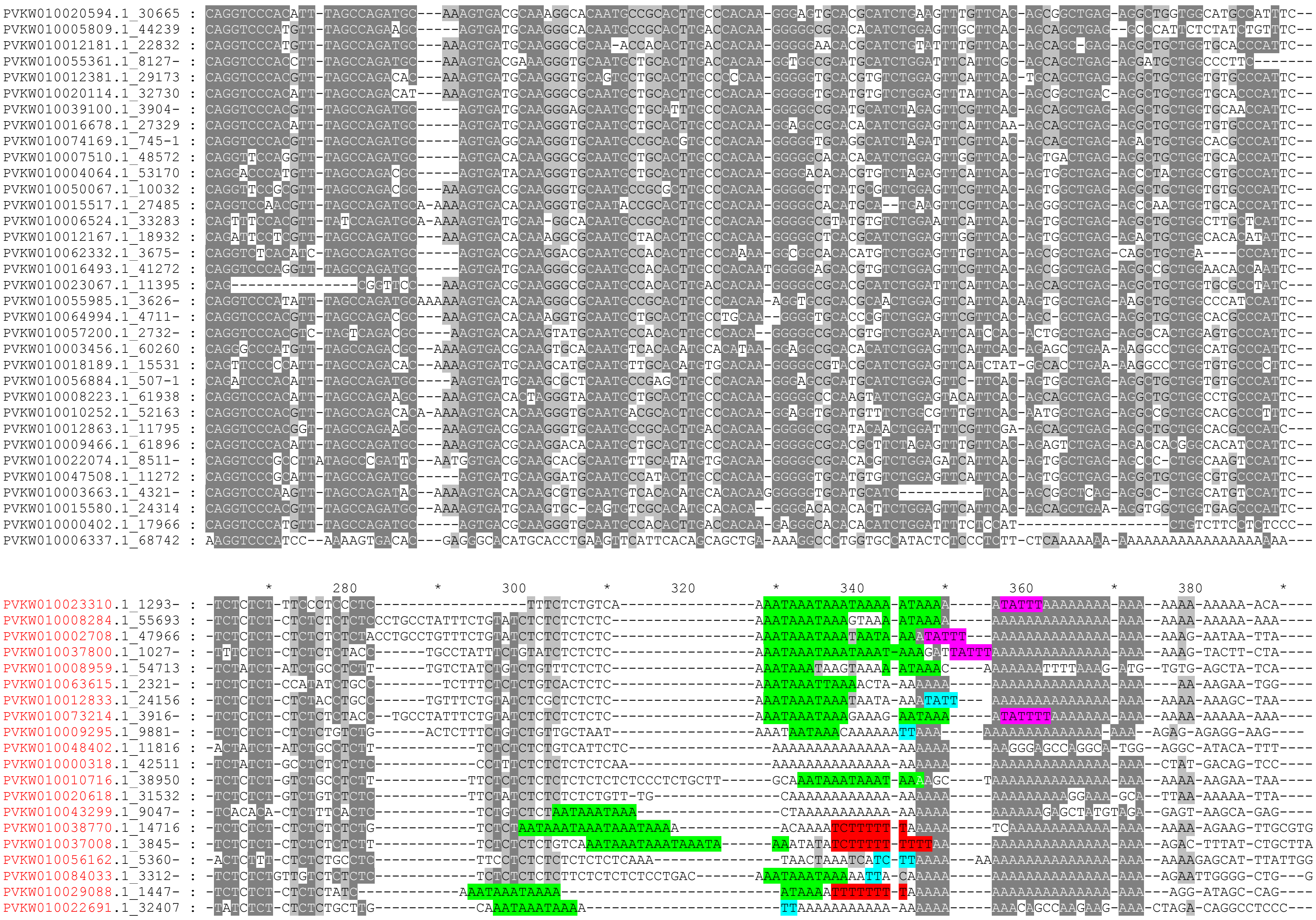

PVKW010020594.1_30665 : CAGGTCCCACATT-TAGCCAGATGC---AAAGTGACGCAAAGGCACAATGCCGCACTTGCCCACAA-GGGAGTGCACGCATCTGAAGTTTGTTCAC-AGCGGCTGAG-AGGCTGGTGGCATGCCATTTC--
PVKW010005809.1_44239 : CAGGTCCCATGTT-TAGCCAGAAGC-----AGTGATGCAAGGGCACAATGCCGCACTTGACCACAA-GGGGGCGCACACATCTGGAGTTGCTTCAC-AGCAGCTGAG--GCCCATTCTCTATCTGTTTC--
PVKW010012181.1_22832 : CAGGTCCCATGTT-TAGCCAGATGC---AAAGTGATGCAAGGGCGCAA-ACCACACTTGACCACAA-GGGGGAACACGCATCTGTATTTTGTTCAC-AGCAGC-GAG-AGGCTGCTGGTGCACCCATTC--
PVKW010055361.1_8127- : CAGGTCCCACCTT-TAGCCAGATGC---AAAGTGACGAAAGGGTGCAATGCTGCACTTGACCACAA-GGTGGCGCATGCATCTGGATTTCATTCGC-AGCAGCTGAG-AGGATGCTGGCCCTTC-------
PVKW010012381.1_29173 : CAGGTCCCACGTT-TAGCCAGACAC---AAAGTGATGCAAGGGTGCAGTGCTGCACTTGCCCCCAA-GGGGGTGCACGTGTCTGGAGTTCATTCAC-TGCAGCTGAG-AGGCTGCTGGTGTGCCCATTC--
PVKW010020114.1_32730 : CAGGTCCCACATT-TAGCCAGACAT---AAAGTGATGCAAGGGCGCAATGCTGCACTTGCCCACAA-GGGGGTGCATGTGTCTGGAGTTTATTCAC-AGCGGCTGAC-AGGCTGCTGGTGCACCCATTC--
PVKW010039100.1_3904- : CAGGTCCCACGTT-TAGCCAGATGC-----AGTGATGCAAGGGAGCAATGCTGCATTTGCCCACAA-GGGGGCGCACATGCATCTAGAGTTCGTTCAC-AGCAGCTGAG-AGGCTGCTGGTGCAACCATTC--
PVKW010016678.1_27329 : CAGGTCCCACATT-TAGCCAGATGC-----AGTGATGCAAGGGTGCAATGCTGCACTTGCCCACAA-GGAGGCGCACACATCTGGAGTTCATTCAA-AGCAGCTGAG-AGGCTGCTGGTGTGCCCATTC--
PVKW010074169.1_745-1 : CAGGTCCCACGTT-TAGCCAGATGC-----AGTGAGGCAAGGGTGCAATGCCGCACGTGCCCACAA-GGGGGTGCAGGCATCTAGATTTCGTTCAC-AGCAGCTGAG-AGACTGCTGGCACGCCCATTC--
PVKW010007510.1_48572 : CAGGTTCCAGGTT-TAGCCAGATGC-----AGTGACACAAGGGCGCAATGCTGCACTTGCCCACAA-GGGGGCACACACATCTGGAGTTGGTTCAC-AGTGACTGAG-AGGCTGCTGGTGCACCCATTC--
PVKW010004064.1_53170 : CAGGACCCATGTT-TAGCCAGACGC-----AGTGATACAAGGGTGCAATGCTGCACTTGCCCACAA-GGGGACACACGTGTCTAGAGTTCATTCAC-AGTGGCTGAG-AGCCTACTGGCGTGCCCATTC--
PVKW010050067.1_10032 : CAGGTTCCGCGTT-TAGCCAGACGC---AAAGTGACGCAAGGGTGCAATGCCGCGCTTGCCCACAA-GGGGGCTCATGCGTCTGGAGTTCGTTCAC-AGTGGCTGAG-AGGCTGCTGGTGTGCCCATTC--
PVKW010015517.1_27485 : CAGGTCCAACGTT-TAGCCAGATGCA-AAAAGTGACACAAGGGTGCAATACCGCACTTGCCCACAA-GGGGGCACATGCA--TGAAGTTCGTTCAC-AGGGGCTGAG-AGCCAACTGGTGCACCCATTC--
PVKW010006524.1_33283 : CAGTTTCCACGTT-TATCCAGATGCA-AAAAGTGATGCAA-GGCACAATGCCGCACTTGCCCACAA-GGGGGCGTATGTGTCTGGAATTCATTCAC-AGTGGCTGAG-AGGCTGCTGGCTTGCTCATTC--
PVKW010012167.1_18932 : CAGATTCCTCGTT-TAGCCAGATGC---AAAGTGACACAAAGGCGCAATGCTACACTTGCCCACAA-GGGGGCTCACGCATCTGGAGTTGGTTCAC-AGTGGCTGAG-AGACTGCTGGCACACATATTC--
PVKW010062332.1_3675- : CAGGTCTCACATC-TAGCCAGATGC-----AGTGACGCAAGGACGCAATGCCACACTTGCCCAAAA-GGCGGCACACATGTCTGGAGTTTGTTCAC-AGCGGCTGAG-CAGCTGCTGA----CCCATTC--
PVKW010016493.1_41272 : CAGGTCCCAGGTT-TAGCCAGATGC-----AGTGATGCAAGGGCGCAATGCCACACTTGCCCACAATGGGGGAGCACGTGTCTGGAGTTCGTTCAC-AGCGGCTGAG-AGGCCGCTGGAACACCAATTC--
PVKW010023067.1_11395 : CAG---------------CGGTTCC---AAAGTGACGCAAGGGCGCAATGCCACACTTGACCACAA-GGGGGCGCACGCATCTGGATTTCATTCAC-AGCAGCTGAG-AGGCTGCTGGTGCGCCTATC---
PVKW010055985.1_3626- : CAGGTCCCATATT-TAGCCAGATGCAAAAAGTGACACAAGGGCGCAATGCCGCACTTGCCCACAA-AGGTGCGCACGCAACTGGAGTTCATTCACAAGTGGCTGAG-AAGCTGCTGGCCCATCCATTC--
PVKW010064994.1_4711- : CAGGTCCCACGTT-TAGCCAGACGC---AAAGTGACACAAAGGTGCAATGCTGCACTTGCCTGCAA--GGGGTGCACCCGTCTGGAGTTCGTTCAC-AGC-GCTGAG-AGGCTGCTGGCACGCCCATTC--
PVKW010057200.1_2732- : CAGGTCCCACGTC-TAGTCAGACGC----AGTGACACAAGTATGCAATGCCACACTTGCCCACA--GGGGGCGCACGTGTCTGGAATTCATCCAC-ACTGGCTGAG-AGGCCACTGGAGTGCCCATTC--
PVKW010003456.1_60260 : CAGGGCCCATGTT-TAGCCAGACGC--AAAAGTGACGCAAGTGCACAATGTCACACATGCACATAA-GGAGGCGCACACATCTGGAGTTCATTCAC-AGAGCCTGAA-AAGGCCCTGGCATGCCCATTC--
PVKW010018189.1_15531 : CAGTTCCCCCATT-TAGCCAGACAC--AAAAGTGATGCAAGCATGCAATGTTGCACATGTGCACAA-GGGGGCGTACGCATCTGGAGTTCATCTAT-GGCACCTGAA-AAGGCCCTGGTGTGCCCCTTC--
PVKW010056884.1_507-1 : CAGATCCCACATT-TAGCCAGATGC----AGTGATGCAAGCGCTCAATGCCGAGCTTGCCCACAA-GGGAGCGCATGCATCTGGAGTTC-TTCAC-AGTGGCTGAG-AGGCTGCTGGTGTGCCCATTC--
PVKW010008223.1_61938 : CAGGTCCCACATT-TAGCCAGAAGC---AAAGTGACACTAGGGTACAATGCTGCACTTGCCCACAA-GGGGGCGCCCAAGTATCTGGAGTACATTCAC-AGTGGCTGAG-AGGCTGCTGGCCTGCCCATTC--
PVKW010010252.1_52163 : CAGGTCCCACGTT-TAGCCAGACACA-AAAAGTGACACAAGGGTGCAATACGCACTTGCCCACAA-GGAGGTGCATGTTTCTGGCGTTTGTTCAC-AATGGCTGAG-AGGCCGCTGGCACGCCCTTTC--
PVKW010012863.1_11795 : CAGGTCCCACGGT-TAGCCAGAAGC---AAAGTGACGCAAGGGTGCAATGCCGCACTTGACCACAA-GGGGGCGCATACAACTGGATTTCGTTCGA-AGCAGCTGAG-AGGCTGCTGGCACGCCCATC---
PVKW010009466.1_61896 : CAGGTCCCACATT-TAGCCAGATGC---AAAGTGACGCAAGGACACAATGCTGCACTTGCCCACAA-GGGGGCGCACGCTTCTAGAGTTTGTTCAC-AGAGTCTGAG-AGACCACGGCACATCCATTC--
PVKW010022074.1_8511- : CAGGTCCCGCCTTATAGCCCGATTC--AATGGTGACGCAAGCACGCAATGTTGCATATGTGCACAA-GGGGGCGCACACGTCTGGAGATCATTCAC-AGTGGCTGAG-AGCCC-CTGGCAAGTCCATTC--
PVKW010047508.1_11272 : CAGGTCCCGCATT-TAGCCAGATGC-----AGTGATGCAAGGATGCAATGCCATACTTGCCCACAA-GGGGGTGCATGTGTCTGGAGTTCATTCAC-AGTGGCTGAG-AGGCTGCTGGCGTGCCCATTC--
PVKW010003663.1_4321- : CAGGTCCCAAGTT-TAGCCAGATAC--AAAAGTGACACAAGCGTGCAATGTCACACATGCACACAAGGGGGGTGCATGCATC----------TCAC-AGCGGCTCAG-AGGCC-CTGGCATGTCCATTC--
PVKW010015580.1_24314 : CAGGTCCCACGTT-TAGCCAGATGC--AAAAGTGATGCAAGTGC-CAGTGTCGCACATGCACACA--GGGGACACACACTTCTGGAGTTCATTCAC-AGCAGCTGAA-AGGTGGCTGGTGAGCCCATTC--
PVKW010000402.1_17966 : CAGGTCCCATGTT-TAGCCAGATGC-----AGTGACGCAAGGGTGCAATGCCACACTTGACCACAA-GAGGGCACACACATCTGGATTTTCTCCAT-----------------CTGTCTTCCTCTCCC--
PVKW010006337.1_68742 : AAGGTCCCATCC--AAAAGTGACAC---GAGGGCACATGCACCTGAAGTTCATTCACAGCAGCTGA-AAAGGCCCTGGTGCCATACTCTCCCTCTT-CTCAAAAAA-AAAAAAAAAAAAAAAAAAAA---
* 280 * 300 * 320 * 340 * 360 * 380 *
PVKW010023310.1_1293- : -TCTCTCT-TTCCCTCCCTC-----------------TTTCTCTGTCA---------------AAATAAATAAATAAAA-ATAAAA-----ATATTTAAAAAAAA-AAA--AAAA-AAAAA-ACA----
PVKW010008284.1_55693 : -TCTCTCT-CTCTCTCTCTCCCTGCCTATTTCTGTATCTCTCTCTCTC----------------AAATAAATAAAGTAAA-ATAAAA-----AAAAAAAAAAAAAA-AAA--AAAA-AAAAA-AAA----
PVKW010002708.1_47966 : -TCTCTCT-CTCTCTCTCTACCTGCCTGTTTCTGTATCTCTCTCTCTC----------------AAATAAATAAATAATA-AAATATTT---AAAAAAAAAAAAAA-AAA--AAAG-AATAA-TTA----
PVKW010037800.1_1027- : -TTTCTCT-CTCTCTCTACC------TGCCTATTTCTGTATCTCTCTC----------------AAATAAATAAATAAAT-AAAGATTATTTAAAAAAAAAAAAAA-AAA--AAAG-TACTT-CTA----
PVKW010008959.1_54713 : -TCTATCT-ATCTGCCTCTT------TGTCTATCTGTCTGTTTCTCTC----------------AAATAAATAAGTAAAA-ATAAAC----AAAAAAATTTTAAAG-ATG--TGTG-AGCTA-TCA----
PVKW010063615.1_2321- : -TCTCTCT-CCATATCTGCC--------TCTTTCTCTCTGTCACTCTC----------------AAATAAATTAAAACTA-AAAAAA-----AAAAAAAAAAAAAA-AAA----AA-AAGAA-TGG----
PVKW010012833.1_24156 : -TCTCTCT-CTCTACCTGCC------TGTTTCTGTATCTCGCTCTCTC----------------AAATAAATAAATAATA-AAATATT--- AAAAAAAAAAAAAA-AAA--AAAA-AAAGC-TAA----
PVKW010073214.1_3916- : -TCTCTCT-CTCTCTCTACC--TGCCTATTTCTGTATCTCTCTCTCTC----------------AAATAAATAAAGAAAG-AATAAA-----ATATTTTAAAAAAA-AAA--AAAA-AAAAA-AAA----
PVKW010009295.1_9881- : -TCTCTCT-CTCTCTGTCTG------ACTCTTTCTGTCTGTTGCTAAT-----------------AAATAATAAACAAAAAATTAAA-----AAAAAAAAAAAAAA-AAA-AGAG-AGAGG-AAG----
PVKW010048402.1_11816 : -ACTATCT-ATCTGCCTCTT------------TCTCTCTCTGTCATTCTC------------------AAAAAAAAAAAAAA-AAAAAA-----AAGGGAGCCAGGCA-TGG--AGGC-ATACA-TTT----
PVKW010000318.1_42511 : -TCTATCT-GCCTCTCTCTC------------CCTTTCTCTCTCTCTCAA------------------AAAAAAAAAAAAAA-AAAAAA-----AAAAAAAAAAAAAA-AAA--CTAT-GACAG-TCC----
PVKW010010716.1_38950 : -TCTCTCT-GTCTGCCTCTT-----------TTCTCTCTCTCTCTCTCTCCCTCTGCTT------GCAAATAAATAAAT-AAAAGC----TAAAAAAAAAAAAAA-AAA--AAAA-AAGAA-TTA----
PVKW010020618.1_31532 : -TCTCTCT-GTCTGTCTCTC-----------TTCTATCTCTCTCTCTGTT-TG---------------CAAAAAAAAAAAAA-AAAAAA-----AAAAAAAAAGGAAA-GCA--TTAA-AAAAA-TTA----
PVKW010043299.1_9047- : -TCACACA-CTCTTTCACTC------------TCTGTCTCTAATAAATAAAA---------------------CTAAAAAAAAAAAA-AAAAAA-----AAAAAAGAGCTATGTAGA--GAGT-AAGCA-GAG----
PVKW010038770.1_14716 : -TCTCTCT-CTCTCTCTCTG------------TCTCTAATAAATAAATAAATAAAA------------ACAAAATCTTTTT-TAAAAA-----TCAAAAAAAAAAAA-AAA--AAAA-AGAAG-TTGCGTG
PVKW010037008.1_3845- : -TCTCTCT-CTCTCTCTCTT------------TCTCTCTCTGTCAAATAAATAAATAAATA------AAATATATCTTTTT-TTTTAA-----AAAAAAAAAAAAAA-AAA--AGAC-TTTAT-CTGCTTA
PVKW010056162.1_5360- : -ACTCTTT-CTCTCTGCCTC------------TTCCTCTCTCTCTCTCTCAAA---------------TAACTAAATCATC-TTAAAA---AAAAAAAAAAAAAAAA-AAA--AAAAGAGCAT-TTATTGG
PVKW010084033.1_3312- : -TCTCTCTGTTGTCTCTCTC------------TCTCTCTCTCTTCTCTCTCTCCTGAC-------AAATAAATAAAAATTA-CAAAAA-----AAAAAAAAAAAAAA-AAA--AGAATTGGGG-CTG---G
PVKW010029088.1_1447- : -TCTCTCT-CTCTCTATC-------------AAATAAATAAAA-------------------------ATAAAATTTTTTT-TAAAAA-----AAAAAAAAAAAAAA-AAA---AGG-ATAGC-CAG----
PVKW010022691.1_32407 : -TATCTCT-CTCTCTGCTTG------------CAAATAAATAAAA-----------------------TTAAAAAAAAAAA-AAAAAA-----AAACAGCCAAGAAG-AAA-CTAGA-CAGGCCTCCC---

```
PVKW010007034.1_12006 : -TCTCTCT-CTACCTCTTCC------------TCTCTCTCTC---------------------------AAATTAAAAAAAA-AAAAAA-----AAAAAAAAAAAAAA-AAA---AAA-AAAAA-AAA----
PVKW010037817.1_14576 : -TCTGTCT-CTCTGTCTCTC------------TCTCTTTCTC---------------------------------ACTCTCT-CCAAAA-----AAAAAAAAAAAAAA-AAA---AAA-AAAAA-AAA----
PVKW010007622.1_53680 : -TCTATCT-CTCTTCCTCTC------------CCTCTCTCTGTCTCTC-------------------TCTGTGTCTCTCT-CCAAAA-----AAAAAAAAAAAAAA-AAA---AGA-AAGAA-AGA----
PVKW010009699.1_36657 : -TCTATCT-GTCCTCCTCTC------------CTTCTC-----------------------------------------TCTCT-CCAAAA-----AAAAAAAAAAAAAA-AAA---AAA-AAAAA-AAA----
PVKW010029164.1_13407 : -TCTATCT-GTCTTCCTCTC------------CCTCTCTC---------------------------TCTGTGTCTCTCT-CCAAAA-----AAAAAAAAAAAAAA-AAG----CA-TGTGC-CAA----
PVKW010093769.1_2826- : -TCTGTCT-TCCTCTCCCTC------------TCTCTCTCTCTCTC---------------------TCTGTCTCTCTCT-CCAAAA-----AAAAAAAAAAAAAA-AAA----AG-ACTCT-CTC----
PVKW010058626.1_8874- : -TCTGTCT-TCCTCTCCCTG------------TCTCTCTGTCTGCCTCTC-----------------TCTGTGTCTCTCT-CTAAAA-----AAAAAAAAAAAAAA-AAT----CA-TCGGC-CAG----
PVKW010038810.1_14115 : ATCTGTCT-GTCTTCCTCTC------------CCTTTCTCTCTGTCTCTC-----------------TCTGTGTCGCTCT-CCAAAA-----AAAAAAAAAAAAAA-AAA----AA-AATTG--GC----
PVKW010001600.1_11740 : -TCTATCT-GTCTCCCTCTC------------CCTCTCTCTCTATCTCTC-----------------TCTGTGTCT-----CAAAAA-----AAAAAAAAAAAAAA-AAA----AA-CCTAT-TGG----
PVKW010071617.1_2076- : -TCTGTCT-TCCTTTCCCTC------------TCTCTCTCTCTGTCTCTC-----------------TCTGTGTCTCTCT-CCAAAA-----AAAAAAAAAAAAAA-AAA----AA-GGAAA-GAA----
PVKW010005913.1_19939 : -TCTATCT-GTCTTCCTCTC------------CATCTCTCTCTGTCGC-------------------TCTCTGTCTCTCT-CAAAAA-----AAAAAAAAAAAAAA-AAA----AA-AAGTT-TAG----
PVKW010021498.1_15139 : -TCTCTCT-GTCTTCCTCTC------------CCTCTCTCTCTGTCTCTC-----------------TCTGTGTCTCTCT-CCAAAA-----AAAAAAAAAAAAAA-AAA----AA-AAAAA-AAA----
PVKW010021740.1_21275 : -TCTATCT-GTCTTCCTCTC------------CCTTTCGGTCTCTC---------------------TCTGTGTCTCTCT-CCAAAA-----AAAAAAAAAAAAAA-AAT----TA-AGGCC-AGT----
PVKW010060547.1_8275- : -TCTATCT-GTCTCCCTCTC------------TCTCTCTCTCTCTCTCTCTCTCTCTCTCT------TTCTGTGTCTCTCT-CCAAAA-----AAAAAAAAAAAAAA-AAG----TT-TATGG-TTT----
PVKW010005965.1_34278 : -TCTATCT-GTCTTCTTCTC------------CCTCTCTCTCTCTGCCTGTTTCAC-----------TCTGTGTCTCTCT-CCAAAA-----AAAAAAAAAAAAAA-AAA----GA-AAAAA-GAA----
PVKW010021433.1_26562 : -TCTATCT-GTCTTCCTCTC------------CCTCTCTCTC-------------------------TCTCTGTCTCTCT-CCAAAA-----AAAAAAAAAAAAAA-AAA----AA-CTCCC-AGA----
PVKW010018455.1_14117 : -TCTGTCT-TCCTCTCCCTC------------TCCCTCTCTCTCTCTCTCTTTCTCTGTGT------CTCTCTGTCTCTCT-CAAAAA-----AAAAAAAAAAAAAA-AAA----AA-ACTTG-GTA----
PVKW010027487.1_15863 : -TCTATCT-GTCTTCCTCCCC---------CTTCTCTCTCTCTGTCTCTA-----------------TCTGTGTCTCTCT-CCAAAA-----AAAAAAAAAAAAAA-AAA----AA-AAAAC-CCT----
PVKW010014973.1_10597 : -TCTGTCT-TCCTCTCCCTC------------TCCCTCTCCCTCTCTG-------------------TCTCTCTCTCTCT-CCAAAA-----AAAAAAAAAAAAAA-AAA----AT-GGGGC-ACT----
PVKW010001443.1_36920 : -TCTATCT-GTCTTCCTTTC------------CCTCTCTCCC-----------------------------TGTCTCTCT-CCAAAA-----AAAAAAAAAAAAAA-AAA---AGA-AGGGC-GGG----
PVKW010002988.1_23682 : -TCTATCT-GTCTTCCTCTC------------CCTCTCTCTCTCTGTTTC-----------------TCTCTATCTCTCT-CCAAAA-----AAAAAAAAAAAAAA-AAA---AAA-AAAAA-AAA----
PVKW010001440.1_80823 : -TCTATCT-GTCTTCCTCTC------------CCTCTCTCTC---------------------------TCTGTCGCTCT-CCAAAA-----AAAAAAAAAAAAAA-AAA----AA-AAAAA-AAA----
PVKW010002157.1_47489 : -TCTATCT-GTCTTCTATCTG-----------TCTTCCTCTCCCTCTC-------------------TCTCTGTCTCACT-CCAAAA-----AAAAAAAAAAAAAA-AAG---AGT-GAGTA-CCA----
PVKW010020676.1_22144 : -TCTGTCT-GTCTTCCTCTC------------CCTTTCTCTCTG-----------------------TCTCTCTCTCTCT-CCAAAA-----AAAAAAAAAAAAAA-AAA----AA-AAAAA-AAA----
PVKW010022582.1_28012 : -TCTGTCT-TCCTCTCCCTC------------TCTCTCTGTCTTTCTCTCTC---------------TCTCTGTATCTCT-CAAAAA-----AAAAAAAAAAAAAA-AAC-----G-ATAGC-AGA----
PVKW010023134.1_13078 : -TCTGTCT-TCCTCTCCCTC------------TCTCTCTGTCTCTCTCTGTG---------------TGTCTCTCTCTCT-CTCAAA-----AAAAAAAAAAAAAA-AAA---AAA-AAAAA-AAA----
PVKW010003325.1_68925 : -TCTATCT-GTCTTCCTCTC------------CCTCTCTCTCTGTCTCTC-----------------TCTGTGTCTCTCT-CCAAAA-----AAAAAAAAAAAAAA-AAA----AA-AAAGC-TTA----
PVKW010011606.1_31074 : -TCTGTCT-TCCTCTTTCTC------------TCTCTC---------------------------------TCTCTCTCT-CCAAAA-----AAAAAAAAAAAAAA-AAG----AG-TTCAC-TAG----
PVKW010000576.1_12211 : -TCTATCT-GTCTTCCTCTC------------CCACTCTCTC-----------------------------TGTCTCTCT-CCAAAA-----AAAAAAAAAAAAAA-AAA---AGA-GAGAG-ACT----
PVKW010008987.1_58106 : -TCTATCT-ATTTCCCTCTC------------CCTCTCTCTC---------------------------TGTGTCTCTCT-CTAAAA-----ATGAAAAAAAAAAA-AAA----AA-AAAAA-AGG----
PVKW010011377.1_47950 : -TCTATCT-GTTTTCCTCTC------------CCTCTCTTTC---------------------------TCTGTCTCTCT-CCAAAA-----AAAAAAAAAAAAAA-AAA----GA-AAGAA-AGA----
PVKW010018780.1_26-72 : -TCTGTCT-CCCTCTCCCTC------------TCTGTCTGTCTCTC---------------------TCTGTGTCTCTCT-CCAAAA-----AAAAAAAAAAAAAA-AAA----TT-AAAGA-GGA----
PVKW010024086.1_7120- : -TCTGTCT-CCCTCTCCATC------------TCTCTC-----------------------------------------TCTCT-CTAAAA-----AAAAAAAAAAAAAA-AAA---AGA-ATAAG-GTT----
PVKW010026760.1_19168 : -TCTGTCT-TCCTCTCCCTC------------TCTCTC---------------------------------TGTCTCTCT-CCAAAA-----AAAAAAAAAAAAAA-AAA----AA-GACTT-ATG----
PVKW010039861.1_8284- : -TCTGTCT-TCCTCTCCCTC------------TCTTTCTCTG-----------------------------TGTCTCTCT-CTCAAA-----AAAAAAAAAAAAAA-AAA---AAA-CTGTA-ATG----
PVKW010015303.1_22501 : -TC--TCT-GTCTTCCCCTC------------CCTCTC---------------------------------TGTCTCTCT-CCAAAA-----AAAAAAAAAAAAAA-AAA---AAA-AAAAC-ATG----
PVKW010003511.1_48444 : -TCTATCT-GTCTTCCTCTC------------CCTCTCTCTC-----------------------------TGTCTCTTT-CAAAAA-----AAAAAAAAAAAAAA-AGA----TC-CAACC-ACA----
PVKW010020066.1_15981 : -TCTGCCT-TCCTCTCCCTC------------TCTCTCTCTG-----------------------------TGTCTCTCT-CCCAAA-----AAAAAAAAAAAAAA-AAA---GGG-ATTGG-GGG----
PVKW010003818.1_10792 : -TCTATTT-GTCTTCCTCTC------------CCTCTCTCTC-----------------------------------TGTCT-CAAAAA-----AAAAAAAAAAAAAA-AAC---TAA-ACTTC-TTA----
PVKW010000020.1_31758 : -TCTGTCT-GTCTTCCTCTC------------TCTCTC---------------------------------------TCT-CCAAAA-----AAAAAAAAAAAAAA-AAC---TCT-ATGAT-TTA----
PVKW010009680.1_37424 : -TCTATCT-GGCTTCCTCTC------------CCCCTGTCTC---------------------------TGTGTCTCTCT-CCAAAA-----AAAAAAAAAAAAAA-AAA---AAA-AAGCA-CTT----
PVKW010004907.1_72306 : -TCTATCT-GTCTTCCTCTC------------CCTCTTTCTCTCTCTC-------------------TCTGTGTCTCTCT-CCAAAA-----AAAAAAAAAAAAAA-AAA---AAG-CTGTT-TCT----
PVKW010000122.1_79232 : -TCTATCT-GTCTTCCTCTC------------TTCTCTCTCCCTCTCTCT-----------------TCTCTGTCTCTCT-CAAAAA-----AAAAAAAAAAAAAA-AAA---AAA-AGAAT-CTG----
PVKW010023178.1_7320- : -TCTGTCT-TCCTCTCCCTC------------TCTCTCTCTC-----------------------------TGTCTCTCT-CCAAAA-----AAAAAAAAAAAAAA-AAA---AAA-AAAAA-AGG----
PVKW010011546.1_32916 : -TCTATCT-GTCTCCCTTTC------------CCTCTCTGTC-----------------------------TGTCTCTCT-CCCAAA-----AAAAAAAAAAAAAA-AAA---AAA-GGCAT-GTG----
PVKW010011545.1_49799 : -TCTATCT-GTCTTCCTCTC------------CCTCTCTCTCTGTCTC-------------------TCTCTGTCTCTCT-CCAAAA-----AAAAAAAAAAAAAA-AAC---ACC-TATGC-AAC----
PVKW010005341.1_66668 : ---CATCT-GTCTCCCTCTC------------CCTCTCTCTC-------------------------TGTTTGTCTCTCT-CCAAAA-----AAAAAAAAAAAAAA-AAA---CAA-AAAAA-AAC----
PVKW010003492.1_33515 : -TCTATCT-GTCTCCCTCTC------------CTTCTCTCTCTG-----------------------TCTGTGTCTCTCT-CCAAAA-----AAAAAAAAAAAAAA-AAA---AAT-TATCA-CCC----
PVKW010043804.1_16010 : -TCTATCT-GTCTCCTTCTC------------CCTCTCTGTCCTC----------------------TCTATGTCTCTCT-CCAAAA-----ATAAATAGGTTTTA-AAA---AAA-TCAAA-AAA----
PVKW010032239.1_6177- : -TCTGTCT-TCCTCTCCCTC------------TCCCTCTCCCTCTCTCTCTCTCTCTCTC--------TCTCTCTCTCTCT-CCAAAA-----AAAAAAAAAAAAAA-AAA---GAA-TAGAG-GTA----
PVKW010053339.1_11250 : -TCTATCT-GTCTTCCTCTC------------CCTCTCTGTCTGTCTCTGTCTG-------------TCTCTGTGTATCT-CTCAAA-----AAAAAAAAAAAAAA-AAA---GTT-CTCGA-TTC----
PVKW010022876.1_14867 : -TCTATCT-GTCTCCCTCTC------------CCTCTCTCTC---------------------------TGTGTCTCTCT-CCAAAA-----AAAAAAAAAAAAAA-AAA---AAA-AAAAT-CTA----
PVKW010013669.1_21934 : -TCTATCT-TTCTCCCTCTC------------CCTCTCTGTCTGTCTGTCTCTC-------------TCTGTGTCTCTCT-CAAAAA-----AAAAAAAAAAAAAA-AAG---ATG-AGGAA-GAG----
PVKW010021200.1_8934- : -TCTATCT-GTCTTCTCTC-------------TC-------------------------------------TGACTCTCT-CCAAAA-----AAAAAAAAAAAAAA-AAA---AAG-AGGCT-GGA----
PVKW010033717.1_18451 : -TCTTCCT-CTCCCTCTCTC------------TCTGTCTGTC-------------------------TCTGTGTCTCTCT-CCCAAA-----AAAAAAAAAAAAAA-AAA---AAA-GGCAC-AAA----
PVKW010072702.1_6047- : -TCTCTCT-CTCTCTCTCTG------------CCTCTCCCTCTC-----------------------TCTGTGTCTCTCT-CAAAAA-----AAAAAAAAAAAAAA-AAA---AAC-ACCAT-GCT----
PVKW010035888.1_16699 : -TCTATCT-CTATCT-TCTC------------CCTCTTTC-------------------------------TGTCTCTCT-CCAAAA-----AAAAAAAAAAAAAA-AAA---AAA-CCTAC-AAC----
```

```
PVKW010000339.1_12536 : -TCTGCTTCCTCTCCCCCTC------------TCTCTCTGTCTCTC----------------------TCTGTGTCTCTCT-CCAAAA-----AAAAAAAAAAAAAA-AAA---AAA-AAGAA-TTT----
PVKW010001061.1_46164 : -TCTATCT-GTCTTCCTCTC------------CCTCTCTGTCTT------------------------TCTCTCTGTGTCT-CTAAAA-----AAAAAAAAAAAAAA-AAA----AA-AACAC-GTT----
PVKW010002728.1_90880 : -TCTATCT-GTCTTCCTCTC------------CCTC----------------------------------------TGTCTCTCT-CCAAAA-----AAAAAAAAAAAAAA-AAG---AAA-TAACT-TTC----
PVKW010083863.1_2646- : -TCTATCT-GTCTCCCTCTC------------CCTCTCTCTTTCTCTCTGTCTGTCTGTCTGTCTCTATCTGTGTCTCTCT-CCAAAA-----AAAAAAAAAAAAAA-AAA---AAA-AAAGC-ATT----
PVKW010002214.1_60035 : -TCTCTCT-CTCTCTCAAAA------------AAAAAAAAAAAAA-----------------------AAAAAAAACAAAG-CTGGGC-----ATGGTGACACATGC-TCT--TAAT-CCCAG-CAC----
PVKW010041582.1_1353- : -TCTGTCT-TCCTCTCTCTC----------------------------------------------------------TCT-TCAAAA-----AAAAAAAAAAAAAA-AAA---AAA-GACAT-GTG----
PVKW010008993.1_54935 : -TCTATCT-GTCTTCCTCTC----------------------------------------------------TGTCTCTCT-CCAAAA-----AAAAAAAAAAAAAA-AAA---AAA-GGCAC-CAA----
PVKW010015692.1_32576 : -TCTATCT-GTCTTCCTCTC------------CCTGTTTCTCTCTCTGTG------------------TCTCTGTCTCTCT-GCAAAA-----AAAAAAAAAAAAAA-AAA---AAG-AATTA-ATT----
PVKW010000763.1_11056 : -ACTATCT-GTCTTCCTGTC------------CCTCTCTCTG--------------------------TCTCTCTCTCTCT-CAAAAA-----AAAAAAAAAAAAAA-AAA---AAA-ACACC-TGA----
PVKW010003844.1_69255 : -TCTATCT-TCCTCTCTGTC------------TCTGTCTCTGTCTCTCTCTCTCTCTCTC--------TCTCTCTGTCTCT-CTCAAA-----AAAAAAAAAAAAAA-AAA---AAC-TTAAA-TTA----
PVKW010017839.1_25636 : -TCTATCT-GTTTTCCTCTC------------CCTCTCTCTCTGTCTCTC------------------TCTCTGTGTCTCT-CTGCAA-----AAAAAAAAAAAAAA-AAA---ATA-CCCTT-TGG----
PVKW010008100.1_7254- : -TCTCTCT-CTCTCTCTCTG------------TCTCTCTCTCTC------------------------TGTTTCTCTTTCT-CTCTCA-----AAAAAAAAAAAAAA-AAA---AAA-AACGT-GTT----
PVKW010000959.1_59475 : -TCTATCT-GTCTCTGACTC------------TCTCTCTCTCTCTCTCTTTCTCTC------------TCTCTGACTCTCT-CTCAAA-----AAAAAAAAAAAAAA-AAA---AAC-CTCTC-TTG----
PVKW010017384.1_32386 : -TCTATCT-TCCTCTCCCTC------------TCTCTCTCTC--------------------------TCAGTGTCTCTCT-CCAAAA-----AAAAAAAAAAAAAA-AAA---AGA-TCTGG-GGA----
PVKW010000072.1_70081 : -TCTATCT-GTCTTCCTCTC------------CCTCTCTATCTCTC----------------------TCTGTGTCTCTCT-CCAAAA-----AAAAAAAAAAAAAA-AAA---AAA-AACAA-GTT----
PVKW010009950.1_49916 : -TCTGTCT-TCCTCTCCCTC------------TCTCTC------------------------------------TGTCTCTCT-CAAAAA-----AAAAAAAAAAAAAA-AAA---AAA-CCCTC-AGT----
PVKW010021276.1_31331 : -TCTATCT-GTCTTCCTCTC------------CCTCTCTCTG------------------------------TGTCTCTCT-GCAAAA-----AAAAAAAAAAAAAA-AAA---ACA-TGGTC-TTT----
PVKW010016288.1_24488 : -TCTCTCT-GTCTCCCTCTC------------CCTCTC------------------------------------TGTTTCTCT-CAAAAA-----AAAAAAAAAAAAAA-AAA---GAC-AAGGT-TCA----
PVKW010020594.1_30665 : -TCTGTCT-GTCTTCCTCTC------------CCTCTCTGCC--------------------------------TCTCTCT-CCCAAA-----AAAAAAAAAAAAAA-AAA---GGC-TGGAT-GGT----
PVKW010005809.1_44239 : -CCTCTCC-CTCTCCCTCTC------------TCTCTGTCTCTC------------------------TCTGTGTCTCTCT-CTCAAA-----AAAAAAAAAAAAAA-AAA---AAA-GCAAT-GGA----
PVKW010012181.1_22832 : -TCTCTCT-GTCTCCCTCTC------------TCTCTGTCTC--------------------------CCTCTGTCTCTCT-CCAAAA-----AAAAAAAAAAAAAA-AAA---AGA-GAAAA-GAT----
PVKW010055361.1_8127- : -TCTGTCT-TCCTCTCCCTC------------TCTCTCTG----------------------------------TGTCTCTCT-CAAAAA-----AAAAAAAAAAAAAA-AAA---GAC-ATTCT--TA----
PVKW010012381.1_29173 : -TCTATCT-GTCTCTCTCAA------------AAAAAAAAAAAAAA----------------------AAAAAAAAGGATT-TTCATG-----CACCCTTTGGTGAA-GTT---ACT-TATAA-TTA----
PVKW010020114.1_32730 : -TCTGTCT--TATCTGTCTC------------TCTCTC--------------------------------------TCTCTCT-CAAAAA-----AAAAAAAAAAAAAA-AAA---AAA-AGAAC-TAG----
PVKW010039100.1_3904- : -TCTCTCT-TTCTCTCTCTC------------TCTCTCAAAAA-------------------------AAAAAAAAAAAAA-AAAAAA-----AAAAAGCTTGAGAA-AGA---CAG-CACAA-TTC----
PVKW010016678.1_27329 : -TCTATCT-GTCTCCCCCCC-------------CTCTCTCAA--------------------------AAAAAAAAAAAAA-AAAAAA-----AAGAGCTTCATCCA-GTC---ATC-CAGGC-TGG----
PVKW010074169.1_745-1 : -TCTGTCT-TCCTCTCCCTC------------TCTGTGTGTCTC------------------------TCTCTGTCTCTCT-TCAAAA-----AAAAAAAAAAAAAA-AAA---ACA-AAGTT-ATT----
PVKW010007510.1_48572 : -TCTATCT-GTGTGTGTCTC------------TCTCTCTCGCAA------------------------AAAAAAAAAAAAA-AAAAAA-----AAAGGACAAAATGT-TTA---ACA-AAACA-GCC----
PVKW010004064.1_53170 : -TCTCTCT-CTCTCTCTCTC------------TCTCTCTCTC----------------------------CAAAAAAAAAA-AAAAAA-----AAAAAAGAAGCCAA-ATA---TGT-TGTGT-TTG----
PVKW010050067.1_10032 : -TCTCTCT-CTCCAAAAAAA------------AAAAAAAAAAA-------------------------AAAATCGGCTGCT-CCTGAG-----GACAAAACTCAGAG-TGT---GGT-TTGGA-TTG----
PVKW010015517.1_27485 : -TCTCTCT-CTCTAGCTCTC------------TGTG--------------------------------------TGTTTCTCT-CAAAAA-----AAAAAAAAAAAAAA-AAA---GAC-CATGA-CAA----
PVKW010006524.1_33283 : -TCTCTCT-CTCTCCTCAAA------------AAAAAAAAAAA-------------------------AAAAAAAAAAAAG-GAAAGA-----AAAGGAAAAGAAAA-GTA---CTT-GAAAG-TTA----
PVKW010012167.1_18932 : -TCTCTGA-CTATCTCCAAA------------AAAAAAAAAAA-------------------------AAAAAAAAAAGAG-CTAGAC-----CAAGTAGAAAAAGG-AAC---TTA-TCTGT-ATG----
PVKW010062332.1_3675- : -TCTGTCT-CCCTCTCCAAA------------AAAAAAAAAAAAAA----------------------AAAAGGGAGAGAG-AGAAGA-----AGAAGAAAGAAAAA-ATC--CAGT-GACAT-TTA----
PVKW010016493.1_41272 : -TCTCTCT-GTCTCTCAAAA------------AAAAAAAAAA---------------------------------AAAAAAAA-AAAAAG-----GATCTGGGCTGGAG-AGC---TGG-AGCGA-TGG----
PVKW010023067.1_11395 : -TCTATCT-GTCTTCCTCTC------------CCTCTTTCACTGTCTTTC------------------TCTCTGTCTCTCT-CCAAAA-----AAAAACAAACAAAC-CAA---AAA-AAAAA-AAA----
PVKW010055985.1_3626- : -TCTCTCT-CTCTCTGCCTC------------TTCCTCTCTCTG------------------------TGTCTCTGTTTCT-CTCTGT-----ATCTGTCTCTCAAA-AAA---AAA-AAAAA-AAA----
PVKW010064994.1_4711- : -ACTGTCT-GTCTCTCTCTC------------TCTCTCTCTCTCTCTG--------------------TGTGTCACTCTCT-CTCAAA-----AAAAAAAAAAAAAA-AAA---AAA-GTTGG-CCA----
PVKW010057200.1_2732- : -TCTCTCT-CTGCCTCTTCC------------TCTCTCTCTCTCTGTT--------------------TCTCCCTGTCTCT-CTAAAA-----AAAAAAAAAAAAAA-AAA---AAA-GGTTC-CCT----
PVKW010003456.1_60260 : -TCTCTCT-GCCTCTTCCCC------------CCTCTCTCTCTCTCTG--------------------TCTCTCTCTCTCT-CAAAAA-----AAAAAAAAAAAAAA-AAA---AGA-ACTAT-TGT----
PVKW010018189.1_15531 : -TCTCTCT-GTCTCTTCATC------------TCTTGCTCTCTC------------------------TGTCTGTCTCTCT-CTTTCA-----AAAAAAAAAAAAAA-AAA---AAC-CCTGC-TGA----
PVKW010056884.1_507-1 : -TCTCTCT-CCCTGCCTCTT------------CCTCTCTCTGTTTCTC--------------------TCTGTCTCTCTTT-CTCTCA-----AAAAAAAAAAAAAA-AAA---AAA-AGCAA--CG----
PVKW010008223.1_61938 : -TCTGTCT-CCCTTTCTATC------------TCTCTCTG----------------------------------TGTCTCTCT-CCAAAA-----AAAAAAAAAAAAAA-AAA---AAG-CATCC-TCA----
PVKW010010252.1_52163 : -TCTCTGT-CTCTCAAAAAA------------AAAAAAAAAA-----------------------------AAAAAAAAGA-TAATAT-----GGTAAAAAGAAAAT-ATG---GTC-TTTTA-GGC----
PVKW010012863.1_11795 : -TCCATCC-GTCTCTCTCCA------------CAAAAAAAAAAAA-----------------------AAAAAAAATCTGG-CTCTAG-----TGTCAAGTATGAAC-TTA---ACT-TCCTC-TCC----
PVKW010009466.1_61896 : -TCTTTCT-CTCTCTCTCTG------------ACTCGCTCAA--------------------------AAAAAAAAAAAAA-AAAAAA-----AACTGCAACTGCAG-AGT---GTA-TTATC-TGA----
PVKW010022074.1_8511- : -TATCTTT-GTCTCGGTCTC------------TCTCTCAATC----------------------------TGTGTGTCTCT-CAGAAA-----AAAAAAAAAAAAAA-AAA---AAA-AAAAA-CAA----
PVKW010047508.1_11272 : -TCTATCT-GTCTCTCTCTC------------TGATTCTCAAAA------------------------AAAAAAAAAAAAA-AAAAAA-----AACAAGAATTCAGC-TCTTCTCCC-AACTC-TTC----
PVKW010003663.1_4321- : -TCTGCAT-CTTCCTCTATG------------TCTCTCTCTC--------------------------TCAAAAAAAAAAA-AAAAAA-----AAAAGACAATCTGG-TCA---AAT-CTAGA-ATC----
PVKW010015580.1_24314 : -TGTCTCT-CTCTCTCTCTC------------AAAAAAAAAAAAA-----------------------AAAAAAAAAAAAC-CTGAGA-----GTCCACCAATAAAC-AAA---AGA-GCCGT-GTT----
PVKW010000402.1_17966 : -TCTCTCT-CTGTCTCTCTC------------CAAAAAAAAAA-------------------------AAAAAAAAAAAAG-ATACGT-----ATCATTAACTAAAA-GTA---CCA-TCAGG-ACC----
PVKW010006337.1_68742 : -ACTTTTA-AAAAAATATTT------------GCTTTAGAGC--------------------------TAGAGAGATGGCT-TAGCAG-----TTAAGGCACTTTAC-TTG---AAG-CCTAA-GGA----


                              400         *        420         *        440         *        460
```

```
PVKW010023310.1_1293- : CAAGCTG--CTCAGTTCAT-GAGAGCCAGGAAGCAGAGAGAACACC-TTCTCACCCAGGCTTTCTCCTTT-TCC-
PVKW010008284.1_55693 : AAAAGAA--TAAGCAGCAG-AGGTGCCGGACTATTTTTGTGGCCAT-GGTCTTTACTTTTGGTGTACTTT-GCC-
PVKW010002708.1_47966 : TAATTTG--AAAAAAACAT-AAAATCAGGAAGGAGGCAAAAGGGAA-CCAAGAGTCGGATTTTCACAAAA-GAC-
PVKW010037800.1_1027- : AAATATA--TATGTTATGG-CTGGCCGAACACTCATCCCCATTCTA-GGTAGAAACAAACAACACAACAC-CCA-
PVKW010008959.1_54713 : CATCTTA--AAAAAAAAAA-AAAAAAAAAAAAACCAGCACTGGAAC-ATATGAGCCATGACTGTTTCTGT-GGG-
PVKW010063615.1_2321- : CAGGGTC--TCAGGCATAC-AACACAAAGAGATCAAAGGCACTTGT-AAGAGGTGCCAGCATTGATGACC-CCC-
PVKW010012833.1_24156 : CCCCAGT--CTGAGCATGC-AGGACAACACTCCATGTGCTACCCCC-AGAACACACATGCACCTCAGAGC-AGC-
PVKW010073214.1_3916- : ACCTTTT--CCAGCTCTGC-CAGAACTTCCTTTCCTTGGTGTAATGTATCCCAAGCTTGGAATGGCACAC-CAT-
PVKW010009295.1_9881- : AGACCAG--GATGTATTTC-AGGGGCACACCTTC---AATGGCCGA-AAGCTCTCCCTTGAGGACTCACC-CCC-
PVKW010048402.1_11816 : TTAATCT--AGGAGGTGAA-GGTAGGAAAATTTCTGTCAGGCCATC-CTGGGACTACAGAGTGAGTTCCA-GGT-
PVKW010000318.1_42511 : TGCTATT--AACAGGCAGT-GGGACTTTTCAGTTTGGCGTACTGAA-ATAATACATTGGTAGAGTTTCTG-AGT-
PVKW010010716.1_38950 : AAGTTTA--TTTTGTTCAC-AGTTCTGGAGGCTGGGAAGTCCCTAG-GGCCTGACGCTAGCATCTGCCGA-CCT-
PVKW010020618.1_31532 : CTGAACT--CCCAATAAAA-AAAAATCCTAACAA-GTTTTCTTTTT-TCTGGTAGGTAAGTTATTTTTAA-AGC-
PVKW010043299.1_9047- : AGACAAG--GCACAGAAAT-CAGAGCCTTTTCTGGGTTATTCTTTTTCTAAGTATCTCACATTTTCCAGC-CTC-
PVKW010038770.1_14716 : TGGGGTC--CCGGCCATGTGTAAACCACTAACTAGGTGGCCCTGGA-GAAGTCACTCTTGAGTCTCCCTT-TCT-
PVKW010037008.1_3845- : CAAGTCC--TCTCCCAAAC-CATGCTCTTTAGCCCACCACCATCTGCCTATCTGTTCCCATTCTTTTCCTTGCC-
PVKW010056162.1_5360- : AAACTTG--TCTAGCTAGC-CCAGCAAATGGAGGGGTAAACAAGAG-ACCATGCCTCAAGAAACAACGTGAACC-
PVKW010084033.1_3312- : AGAGAGG--GCTCAGAAGT-TAAGGCACTTGCCTAAATACCCAAGT-TTGCCAGACATGGTGGCGCACAC-CTT-
PVKW010029088.1_1447- : TATTTTG--GTTCCTGCTT-CCAAGTCAGAAGACCACAGGAGATGATGGGGCACTTGCTAACTTCTAGCC-TCTG
PVKW010022691.1_32407 : GGAGTTT--TCTCATTTAA-TCTATTAGCATTAG---TGCACACCCTTTTTGTACAACTGTAGTTCTACA-CAG-
PVKW010007034.1_12006 : AAAAAAA--AAAAAAAAAA-AAAAAAAAAAAAAA----AAAAAAAA-AAAAAAAAGCAAGTGGC--GCAC-ACC-
PVKW010037817.1_14576 : AAAAAAA--AAAAAAAAAA-AAAAAAAAAAAAAA----AAAAAAAA-AAAAAAAAGGATATACCAGCTCT-CCA-
PVKW010007622.1_53680 : AAAAAA---GAAAAAATGT-AATGTCAATAGAGA----GTTGTAAC-ATATTATTGGTGTTCTTTTCTTC-CAA-
PVKW010009699.1_36657 : AAAAAAA--AAAAAAAAAG-AAGGCTTGCATTTC----ACAAGAAG-GGTATAGTGTATATTTCTATGTA-AAT-
PVKW010029164.1_13407 : GTGTGGT--GGCTCACATC-TGTCATCCCAACCC----TCAGGAG--GCTGAGGTAGGAGGATC--ACAA-GTT-
PVKW010093769.1_2826- : ATACAAC--CCTTGAGGGGGTGAACTGAGTGAAT----CCCATAACGCATTAGCATATAATACGTAATCA-AAA-
PVKW010058626.1_8874- : GTGTGGT--GGTGTAGGCC-TGTAACCCCAGCAC----TAAGGAG--GCTGAGGCAGGAGGATC--ACAA-GTT-
PVKW010038810.1_14115 : ATCTAGC---TTCTGTACA-CTAGTTGTGTCTAG----TGTAACA--AAAACCATATTCAATG---TTTG-TTT-
PVKW010001600.1_11740 : ATATCAC-----CTCCCTC-CTATTTATATTGAC----ATTTATG--TTATTCCTCAAAAATGA--ATAA-AAG-
PVKW010071617.1_2076- : GAGAAGA--GAAGAGAAAA-GGAGTTGGAAAGTC----TTCATAA--ACTATTAAAGCTTGTTT--GCCC-ATT-
PVKW010005913.1_19939 : GATTGGC--ATGCCAGTCT-GAAGATTATATAAA----TCCTTTC--TTATAATTGGTCAAAAG--ACTA-CAA-
PVKW010021498.1_15139 : AAAAAAA--AAAAAAAAAA-CTTAATAATTACTA----TTAAAGA--AACAGCACCAGCATATA--TGCA-TCA-
PVKW010021740.1_21275 : GTTCCAC--ATACTTGGGT-AAGAATAACTGGAC----ACAGTTATGATTTGGGCAGTTGCTCCTGGCCT-CGC-
PVKW010060547.1_8275- : GAGTCAG--TAACTTAATG-ATATCCAAAATAAG----CGTGACG--TTTTTAGTT---------------TTG-
PVKW010005965.1_34278 : AAATAAA--AAGAAAATAT-ATACCTGCCTAAGA----ATCAAGG--GTGAAAATAAAATC-----TGGA-ATC-
PVKW010021433.1_26562 : GTGTAAA--GAATAGACAGGAGAAAGGAATCCAC----TCCCCCC--TCATTCTTGTTTCTGTT--TCTT-GTT-
PVKW010018455.1_14117 : CTGTCCT--GCTCTTAAAT-TTTAACCATTTTCA-----TATGTG--TGAATATGCTCCACTC---AAAA-TGA-
PVKW010027487.1_15863 : ATGTCTG--GGAAGGTCAT-AGGCTCTAAGAAGG-----AATCTA--CTACTGCTGTTTGGCT---AAAT-AGA-
PVKW010014973.1_10597 : GAACATT--CTTTCCACCG-CTAGCCTCAGTCAA----CACCTGG--ACCATGGTGTCAGAAG---GCCA-CAG-
PVKW010001443.1_36920 : GAAGGGT--CAATCAAAAT-TCAAAATATTCTGA----ATAAACA--TATGAAAACCACTTTTT--TGAA-TAA-
PVKW010002988.1_23682 : AAAAAAAAAAAGAATGAGA-TCTCAATACTGTGA----TGGTGGT--TTGGTAACAGTGACAT---AAAA-GTT-
PVKW010001440.1_80823 : AAAAAGC--TAATAGAACT-GGCACATGATTCAG----CTATATC--ACTTTTGGGTACATACCTAAAGG-AAT-
PVKW010002157.1_47489 : GATCAGC--CTGGGCTAGA-GTGAGACCCTGAAA----ACAAAAT--AAACAAATAGTCAAAC---AAAA-TAA-
PVKW010020676.1_22144 : AAAAAAA--AAAGCATGGT-CCTGAATATTTTGT----GCATGGT--TTAATCCTTAGGGCCTAGCTTAG-TAG-
PVKW010022582.1_28012 : AAACATA--GAGAAAAAAA-TTAACTGCCTGTAA----TTCCACC--ACTTAGACATAACTACT--GACA-ACA-
PVKW010023134.1_13078 : AAAAAGA--CTTGGCAAGC-CTGGTTCACTGAAG----TTAGGAA--AAGAAAGCAGTCTTGAG--ACAG-GAA-
PVKW010003325.1_68925 : AAAGAAA--TAGCCCAAAT-ATTAATAGTGGTTA----AGAGATG--GAAATATAACTTTTTA---TTTC-CTA-
PVKW010011606.1_31074 : GAAATAC--AATCAGACTG-CAGTATGTTCATGT----ATTAAAC--TTCTCAATAAAAATGTT--AAAA-ATG-
PVKW010000576.1_12211 : ATGTATG--TGTGTTTAAT--AGCACAAAAGAGG----TCATTGA--ATTGTAGTACAAGAAC---TAAA-TTT-
PVKW010008987.1_58106 : AGAAACC--TAGCTGAGCA-TTCAGCCTTCATTG----CTCTCCA--TTTCCTTCATGCTGATGTAATTA-TGT-
PVKW010011377.1_47950 : AAGAAAT--TAGAATATAT-AGAAGCAAAAAAAG----GCAAAGGT-TTTTTTATGCCAGCATT--TAGT-AAC-
PVKW010018780.1_26-72 : TACAGAT--AAACTAAAAT-AATATAAAAAGAAT----ACAAATAA-ACACTGAGGGAAATAAAGTCAGT-ATA-
PVKW010024086.1_7120- : CAATTCC--CAAGCCACTC-ACATAAGCTGGATGCTCTTGCAACAGCAAGAGCTCACAGTGACACACATG-CAT-
PVKW010026760.1_19168 : TATAATT--TCAGTTATAT-TCTCAAAATTGAGA----AAGCAC---TGAAAAATTGTTATTCC--AAGG-CAA-
PVKW010039861.1_8284- : CAAGAGC--CAAATGCACT-GTGATGTGTCACCA----ACGAATTA-GCAAACATGACTTTTTC--ACCT-GTG-
PVKW010015303.1_22501 : CAAGAAA--ATAGATGAAC-TTGGAAGGTTATAC----TAAGTGAA-GTAACCCAGGCCATATG--TAAG-TAC-
PVKW010003511.1_48444 : AATCCAC--CTGTCCAAAC-ACTCTAGACTTTGT----TTTAAGT--CTTTGGATAAAGAGACTC-CAGT-GAA-
```

```
PVKW010020066.1_15981 : AAAGACA--CAAAGCTAGG-TCTGTATTCAAGTA----AACACAGA-GCTGACTTGGAACTAG---AAAT-TCG-
PVKW010003818.1_10792 : ATTCAAT--CCAAGTACAC-TTTTAGAATGATTT----TAAGGTTG-AAAAGTCACAACAAATG--TGAG-TTA-
PVKW010000020.1_31758 : ACTTTCA--CTGCCAATAA-GAACTTGAATTTAA----AGAAACTC-AAAGCTTTGAGTATCTTT-ATTG-TTT-
PVKW010009680.1_37424 : AAAATAA--TGATAGGGTG-CTTGTAAATTGAAT----TTTGTTT--TTGAGGCAGGAAAAACA--TGAA-TTT-
PVKW010004907.1_72306 : CTCTCTC--TCTTTTTTTT-AATATATAATATGC----TCTTGTTA-GTGGGAATTGTAGGGGT--AATT-AGC-
PVKW010000122.1_79232 : AATTCTG--TCACCAGACC-AAGTCAACACATAG----TATAAGTC-ATTAACTTCTGCAACA---AATC-ACT-
PVKW010023178.1_7320- : TTGGGCT--GGAGATATGT-CTCGGTGGTTAAAG----GGACTTGC-TTGAAAAGCTTGACAACCTGAAT-TTG-
PVKW010011546.1_32916 : TCATCAT--GCCTGATTAA-AAAAAAAAAATGGG----CTGGAGA--GATGGTTTTGCTATTAAGGCATT-TGC-
PVKW010011545.1_49799 : ACAGAAG--GCTGAAATAT-GTGGATGGCATGAG----TTCAAGG--CCAGTCAAGTCTACATCATGAGT-TCA-
PVKW010005341.1_66668 : TTGAGGA--GGAGCCTTAT-GAATCTTACAAATT----TCAGGAGC-TTACTTTGTGAGTTTTGCTTACT-TCC-
PVKW010003492.1_33515 : AGGTAAA--TGTTGTAAGG-ATTTATCTAGATTA----TTATACAC-AAGATAGTGCATTTCTATTGCTG-TAT-
PVKW010043804.1_16010 : AAAAAAA--AAAAAAAGGA-AAAAAGAGAAGGAA----GGGAGGA--AAGAAAGGAAAGAAG------------
PVKW010032239.1_6177- : TTGGAGA---GGCGTGTCT-GCTTCTGTTTGGAT----ACTCAAC--AAGCTCAGGAAGAAGA---GAGT-ACT-
PVKW010053339.1_11250 : AAACCAC--CAGCTGAAAA-TTGTGCTCTCTTGA----TAATTAGT-ATCAGCATTGCCAACTGCCACTT-GCA-
PVKW010022876.1_14867 : ATGAAAA--CAAATACTGC-CAGGGTTGTGGAGA----AATAGGAA-CTCTCATTCACACTGCTGGTGGG-AAT-
PVKW010013669.1_21934 : AAAATAA--GTCCAGAAAT-AAACCTTCTTATAC----ATAATAAA-GATATTTTCAACAAA----GGTA-CTA-
PVKW010021200.1_8934- : GGGCTGG--GGATGTAGCT-CAGTGGTAGTGTAC----TTGCCTAACATGTATAAGGCTTTGGGTTAATC-CCC-
PVKW010033717.1_18451 : AGGTCTC--CAGGAAACTT-TATCTGAAACTCCT----GGAAGGCT-AGAATTAAGTATGAATAGGGTAG-AAC-
PVKW010072702.1_6047- : CATTCTT--ACATATCTGG-GAATTGACCACAGT----CAGTTGA--ATAGCAGCTGTTTGTTTCCAGTG-GCT-
PVKW010035888.1_16699 : TCTGGCC--AACCACTTGG-GTCAAGGGTCTTCC----AGTCCTAT-TTCTCAACCTTGTTCCAAGGTGA-GTG-
PVKW010000339.1_12536 : TTGCATT--TTTGCATGGC-TGGAGAAAGAACTT----AACAGTTA-ATGCTTTGCAGTAGCTG--GAGA-CTC-
PVKW010001061.1_46164 : TGGCCAA--GTAAGCCTGT-AACCCCAGTCCTGT----GGGAGGC--AGACAGGAGAATCAATGGGACTC-AGT-
PVKW010002728.1_90880 : AGGCCAG--GCATGGTGTC-ATACACATTTAATC----CAAGCACT-CGTGAGACTGAGGTAGGAGGACT-GCA-
PVKW010083863.1_2646- : TCTGAGC--TGGGTATGGT-GGCGCATGCCTTTA----ATCCCAG--CACTCAGGAGGCAGAAGTAGGAG-GAT-
PVKW010002214.1_60035 : TCGGAAG--GACAAGGAAG-GAGGGTTGCTGTGA----GTTTGAGG-GTAGCCTGAGATTACATAGTGAA-TTC-
PVKW010041582.1_1353- : CCACCAT--GCCTGGCTAA-AAAAAAAAAAGCTT----CACGCTGTGGGGAACAGAAACAGTCACCTGAGGGTC-
PVKW010008993.1_54935 : TAAACCA--ACATACTTGG-GAGAGAGTACTACC----TTTTTGACTGAGTGAACAAATGAGTACACTTT-GTT-
PVKW010015692.1_32576 : ATCTCAC--ATAATGTAGG-AGTTTTAGATAAAT----AATAGGGT-ATAGATGCCAGTAACTC--CAAG-GTG-
PVKW010000763.1_11056 : CTTCATA--TTTTTCAAAC-AACAAGAGCTGGAG----ACAGAGTT-CAGTGGTAGAGTGTTCACTTGCCTACA-
PVKW010003844.1_69255 : CCAATCA--TTTCAGACAG-TTTCAGACCTGAAA----AGCCCCAG-AATCTCTCTCTTGATG---CATC-ATT-
PVKW010017839.1_25636 : AGAAGTA--AAAGCGTAAT-TGATTCAGGAAAAG----AATGAGAG-CTCTAAGGTTATTTGGATAAAAT-CTC-
PVKW010008100.1_7254- : GGGATAG--GCAAAAGGAC-CATGGCAAACTTTT----GAAAAGAA-GGATTCCAAGATGGGAA--AGGC-CTC-
PVKW010000959.1_59475 : GCATAAT--GGCAATGGGG-AACTGTACAAAACT----GTGTCAGC-TCCAGCTAAGCACATAT--CAAT-CCT-
PVKW010017384.1_32386 : GGGAGCA--GGGTGCATAG-AAATAAGCTAATAT----AAGGAAAAAGCAAGAAAATGCCACTGCCTGCC-AAC-
PVKW010000072.1_70081 : ACAAAAT--CCAAATGTCT-AATCCTAACAAGAT----TTATATAG-TTAAGACAAGATATTGTCAGGCA-ACA-
PVKW010009950.1_49916 : GTTAGGG--GAAAACCTCC-AAGACTTAGATGAT-AATTAGAAGGTTCAATTGCCTCATGTGTACTATAA-TTT-
PVKW010021276.1_31331 : GCTTTCA--GCCTAAGAGT-GACCAATCCTATGT----GTCTACGT-GTGACCTTTGGCGATTCCCTCAA-CGT-
PVKW010016288.1_24488 : ATGGCCC--TAGCACCTGA-CCCAGGTATCCTTG----AGGCAAGACGCATTTGGCAATAGCTGCCTGCT-TTT-
PVKW010020594.1_30665 : TCAGAGA--TGACAAAACC-TCATGCTTCTTGCT----TCTGAGGCCACAGTCAGCTCTTGGCCAAATCT-GGA-
PVKW010005809.1_44239 : TCAATAA--AAATAAAGTT-GCATGTGTGTAATT----GGATGCTA-GTGATTTTGAACAAAACAATTCA-ATG-
PVKW010012181.1_22832 : TAATGAA--AGCATGATAA-GTGTGGAGGCCTTG----AAACTTAA-TTTCCATACCATGATTTTAATAC-TTC-
PVKW010055361.1_8127- : TTTCTGC--TTAAACAAAT-AGTGGCACAAAGTA----GACAACAC-TAGCTGTCAAGAAAAGAGGGGGA-AAG-
PVKW010012381.1_29173 : TGTATAA--ATTCACACAA-TCAAATTGAATAAC----AAGGTAGGTTGGATATGTCATAATTTCTGATA-TGC-
PVKW010020114.1_32730 : TGTGTAT--TGAGCACTTA-GTGTGTCCTGGGCC----CTTCATGT-GGCATTTCATCCAACTCTAAGAT-GAG-
PVKW010039100.1_3904- : CATAGGA--AACCTTAGTC-AGCCAATGGAGCTT----TCAGAGAGGAACTAGAATGAGACAGACCCAGC-ATT-
PVKW010016678.1_27329 : GGAAAAC-AGTCACATCAC-GTCACTGTTGGACT----GGAGAAGG-CCACCAATGACCCAAAGAGGCCA-CCG-
PVKW010074169.1_745-1 : CCCACAT--GATTATCCAA-CCTGCTGGTTCTCC----ACCCTGGC-TGCCCATTAGGACCACGTGCATG-GCT-
PVKW010007510.1_48572 : TGTCCTT--TTCAAGGGAA-GATTATAAATAATC----TTTTTTTA-TTTTTTTATTTTTATTTTTTATA-GTT-
PVKW010004064.1_53170 : TGGCCTA--GGGACATGAT-TTAGTTACCCCAAG----TACCATGT-TCCAATGTGTCAAAAGCTTGGTA-AGG-
PVKW010050067.1_10032 : TAAACAC--ATTTTGGGAT-CAAAGACTGATAAG----GAAGATAC-TTGAGTGATTTGGTT----CTAG-GCC-
PVKW010015517.1_27485 : GCAGTTT--TCTAGATTTT-TAAGACTTTATTTT--AATGAGAGAGGGGAAAAAAGGGAGAGGAAAACAT-ATG-
PVKW010006524.1_33283 : TTGGCTT--GGTCAGAGCC-ACTGGTTAAAGGAG----TTGTAAAC-TTATCCAGGACTTTTACTGGTGA-GGC-
PVKW010012167.1_18932 : GAGCTAC--CCAGATACAC-TGTCACTGTGGCAC----TGTGGCACTGTGGCTGCTGTTGTCTTCCAGAT-TGA-
PVKW010062332.1_3675- : CAACAGT--GGAACTGTAA-GTTCAGGTTAGGCT----TTAGAATC-TAATTCATTGTTTAAAATAAAAA-ACAG
PVKW010016493.1_41272 : CTTAACA--CTTAAGTGCA-CTTCCTATTCAAAT----GTGAAGGT-TTGAGTGAGGCAGAAACCGCAGA-GTT-
PVKW010023067.1_11395 : AAAAAAA--AAAAAAAAAA-AAAAAAAAAAAAAA----ACCAATAC-TGTTGCCCTGCTGCCAAACCAGA-AAT-
PVKW010055985.1_3626- : AAAAAAA--AAGAAGAAGA-AGAAGAAGAAGAAA----GACTCCAG-AGGACACATCAAGAGAGTGAATA-TGC-
```

```
PVKW010064994.1_4711- : GGTGCAG--TGGGAATTGA-AAATTGTACCTGGA----GTGGGAAA-GGAGCGGAACTTGGAGAGGGTTC-CAT-
PVKW010057200.1_2732- : GAATGAG--GGAAACCTGA-ACTTGTAGAGGGGT----CCTGCAGT-ACCACCAAGAGTAAAACCTTGGG-CAT-
PVKW010003456.1_60260 : TGTGCCT--TTCACTTTGT-ACATGAAGGAACTG----TATTTTG--GAATAAGTGACTTACTTCAGTGT-CCA-
PVKW010018189.1_15531 : TATTACC--CCATAAGGTG-CTCCATATTTTGTC----CTAAATGC-CTCACTACTTAGCAGACAGTAAG-AAA-
PVKW010056884.1_507-1 : TCATCAT--TACTATGAGG-CTTCTATCCGTGGT----ATACAGAA-GCACTCTAAAATCTTCATTGGCA-AGC-
PVKW010008223.1_61938 : AAACACCTGTTTTGATGTT-GTTTGAGTTTTTTTATAGTTTCTAT-TTCCTTATAACAGTCTTTCAGTT-TCA-
PVKW010010252.1_52163 : AAAGCCC--TCATGATTTT-CAAGATTCCCAGTG----TAATTAGT-GCTTGGAAAATGTCAGAGGTAAC-AAT-
PVKW010012863.1_11795 : AGCTATG--TCTCAGGCAG-CTTGGGACTCCTCT----GAGGAAAT-ATGGGTGAAACTCAAAGG-AACC-AAG-
PVKW010009466.1_61896 : CCACAAT--GTAATTATGC-TTTGCTGATAAGCA----TTAGAAAA-CTCTTAAATATTTTGTCTTTTAT-CTG-
PVKW010022074.1_8511- : GGGTTCC--TCCCCAGTCC-TGATAGAGTGGCCC----TTTGCCCT-ATGGAAATAGTCCATGGCATTTG-GTT-
PVKW010047508.1_11272 : CAGATAG--GATACCAACT-TTCATTTGGCAGAA----ACAAAACC-CATACAAATCATTGGCCAAGGGA-GCA-
PVKW010003663.1_4321- : TCAAAGT--ATCTTGAATT-CACACCCAGCTCCT----TCATAAAA-GCAGGTAATTGATTTATGATTCT-CAC-
PVKW010015580.1_24314 : GCTGGGG--AAGGGTCTGT-GCCCATAAAAGAGA----ATGCCCAC-CAGTAATGAAGTGGCTTCTTACC-AAC-
PVKW010000402.1_17966 : AATGCAT--GACTTTATGA-AGAAAAGGGATTTG----TTTGGTTC-ATGCTTAAATTAAAGAAGTAGAT-GTC-
PVKW010006337.1_68742 : CCCATGT--TCAACTCTCT-AGGTCTCATGTAAG----CCAGACAC-ACAAAGTGACACAAGCATGCAAG-GTT-
```

Figure S14. *A. bullata*-specific Dip copies with long ($A_{>20}$) tails. Names of Dip_a copies are shown in red; all other sequences belong to subfamily Dip_b. Nucleotide sequences are highlighted as follows: PASs, strong pol III terminators, medium terminators, and rudimentary terminators.
