# Supplementary material for: Analysis of SINE Families B2, Dip, and Ves with Special Reference to Polyadenylation Signals and Transcription Terminators
Source: Int J Mol Sci. 2021 Sep 13;22(18):9897. doi: 10.3390/ijms22189897 (PMC8466645; doi:10.3390/ijms22189897)
Supplement: Supplementary file 1 [file ijms-22-09897-s001.zip › Supplementary Vassetzky/SINE Dip. Figs S7-S22. Tables S6-S8/Fig S20.pdf]

```
DIP        : CTCTCTCTCTC--------------AAATAAATAAATAAAAAA---------------------------------------------------------------------------------------- : 208
PVKW010131 : TCCTCTCTC----------------AAATAAATATGTAAAAAAAAAAAAAAAAAAAAAA------------------------------------------------------------------------ : 225
PVKW010029 : CCCTCTCTCTG------TCGCTAATAAATAAAATCTTAAAAAAAAAAAAAAAAAAAAAAAAGGCTGGGCATGGTGACGCTCACCTT--------------------------------------------- : 260
PVKW010005 : CTCTCTG----------AAATAAATAAATGAAATGAAATGTAAAAAAAAAAAAAAAAAAAAAAAGAACTCATTTGGAAAACTTTTTGGAA----------------------------------------- : 262
PVKW010011 : CTCTCTCTCTC------TCTCTCTGAAATAAATATTAAAAAAAAAAAAAAAAAAAAAAAACAGCAGGTGTATGCTCTACAATTTCA--------------------------------------------  : 256
PVKW010022 : CTCTC----------------AAATAAATAAAACTTAAAAAAAAAAAAAAAAAAAAAACTTTTTAAAACAAAGATCTCAGCACTTGGCAGGCAGAGG---------------------------------- : 257
PVKW010034 : CTCTCTGTCTC------TAATAAATAAATAAAAATAAAATCTTTAAAAAAAAAAAAAAAAAAAAAA----------------------------------------------------------------- : 240
PVKW010007 : CTCTCTCTCTC------AAATAAATAAATAAATATTAAAAAAAAAAAAAAAAAAAAAAAAAAACCTCTGCACGTGAGTCT--------------------------------------------------- : 261
PVKW010021 : -----------------ACATAAATAAATAAAAATAAAATAAAATAAAAAAAAAAAAAAAAAAAAAAAAAAAAAAAAAAAAAAAAAAAAAAAAAAAAAAAAAAAAAAAAAAAAAAAAAAA------------------ : 257
PVKW010016 : -------------------------AAATAAAAAAAAAAAAAAAAAAAAAAAAAAAAAAAAAAAAAAAAAAAAAAAAAAAAAAAAAAAAAAACGCTACATAAATAACGTGTGAT------------------ : 263
PVKW010072 : -------------------AAATAAAAATAAAATCTTAAAAAAAAAAAAAAAAAAAAAAAAAAACCTTTCCTCA--------------------------------------------------------- : 228
PVKW010029 : ---TCTCT-------AATAAATAGATGAATAAAAAATATTAAAAAAAAAAAAAAAAAAAAAAAAAAAGCTTTTAGGCCAGGGAGAGAGACTGAATG----------------------------------- : 263
PVKW010003 : -------------------------AAATTAAAAAAAAAAAAAAAAAAAAAAAAAAAAAAAACCTTACTGTATGTGACTGAGTGTGGAATCTTGACAAGCATGGTATCTA--------------------- : 262
PVKW010052 : ---------------------AAATAAACAAAATTTTCCAAAAAAAAAAAAAAAAAAAAAAACACATAGCATGGTATCTAGGTGTGTAAAGACCGAGTTCAAATCATGACTCT------------------ : 260
PVKW010503 : ------------------------AGATAAAGATTTAAAAAAAAAAAAAAAAAAAAA-------------------------------------------------------------------------- : 205
PVKW010044 : TCTCTGCTTGA--------------AAATAAACCAATAAAAAAAGTAAAAAAAAAAAAAAAAAAAAAAAAAGAACCTAACCAGACAGGAAC---------------------------------------- : 261
PVKW010069 : CTC----------------------AAATTAAAAAAAAAAAAAAAAAAAAAAAAAAAAAAAAA-------------------------------------------------------------------- : 221
PVKW010063 : TGCTTGC------------------AAATAAATAAAAAAAAAATAAAAAAAAAAAAAAAAAAAAACTACCTTGAGATTCCATCTCACTCCTGTCAGATT-------------------------------- : 260
PVKW010011 : --------------------------AATAAAATAAAAATATTAAAAAAAAAAAAAAAAAAAAAAAAAAAAAAAAAAAAAAAAAAAAAAAAAAAGAAAGGAGCTGGGCATA-------------------- : 259
PVKW010057 : TTCTGTCTCTC--------------AAATAAGTAAAAAAAAAAAAAAAAAAAAAAAAAAAAAAAAAAAAGGCCAGGCATGGTGGCATACCC---------------------------------------- : 257
PVKW010014 : TT--------------------AGTAAATAAATTAATTTAAAAAAAAAAAAAAAAAAAAAACAGCCCTTCTGAGCCAGGCATGGTGGCACATGCCTTTAAT------------------------------ : 261
PVKW010021 : TTCTCTCA------AATAAATAAATAAAAATAAAACTACCAAAAAAAAAAAAAAAAAAAAAAAAAAAAAAAAAAAAAAAAAAAA----------------------------------------------- : 259
PVKW010000 : CCCTCTCTGTCTTTAATAAGTAAACAAATAAATAAACATGAAATCTTAAAAAAAAAAAAAAAAAAAAAGAATCTGTTTAAG-------------------------------------------------- : 258
PVKW010040 : CTCTCTCTCTC----------AAATTAATAAATAAATAAAAATAAAATATTAAAAAAAAAAAAAAAAAAAAAAAGAATATATAAAGA-------------------------------------------- : 266
PVKW010219 : TTCTCTCTCTC----------AAATAAATAAATAAATAAAATATCAAAAAAAAAAAAAAAAAAAAAAAAAAAAAAAAAAAAAAAAA-------------------------------------------- : 269
PVKW010304 : CTCTCTCTCTC--------TCAAATTAATAAAAATAAAATTTAAAAAAAAAAAAAAAAAAAAAAAAAAA-------------------------------------------------------------- : 254
PVKW010062 : CTGTCTGTCACTC----TAATAAATAAATAAATAAGAAATTTAAAAAAAAAAAAAAAAAAAAAAGGGCTCTGGGGAA------------------------------------------------------ : 268
PVKW010003 : CTCTCTCTCTC----------AAAAAAAAAAAAAAAAAAAAAAAAAAAAAAAAAAAAAGAAATGC------------------------------------------------------------------ : 269
PVKW010034 : CT----TTCTC----------TAATAAATAAATAAAATCTTAAAAAAAAAAAAAAAAAAAAAAAAACTATGGCTGGGGAGGCCATAAGACCTAGCCAGG-------------------------------- : 267
PVKW010991 : CTGTCGCTCTC----------AAACAAACAAACAAAAAAAAAAAAAAAAAAAAAA---------------------------------------------------------------------------- : 222
PVKW010041 : CTGTTGCTCTC----------AAACAAATAAATAAAAATTAAAAAAAAAAAAAAAAAAAAAAAAAGAATGTAAGAGCCAAAGGAAGGGTAGGACT------------------------------------ : 267
PVKW010002 : CTGTTGCTCCC----------AAATAAATAAATAAACCAAAAAAAAAAAAAAAAAAAAAAAAGAAGAAGAAATACAAATGGCCAAT--------------------------------------------- : 269
PVKW010380 : ---------------------GTGCAAATAAATACATTTAACAAAAAAAAAAAAAAAAAAAAA-------------------------------------------------------------------- : 217
PVKW010064 : ------------------AATAAATAAATAAACAAAATCTAAAAAAAAAAAAAAAAAAAAAAAGGGCCAGGCATGGTGGCTCATGACTTCAATCCCAGCACTGGGGAGG---------------------- : 269
PVKW010043 : -------TCTC--------------AAACAAATAATTAAAAAAAAAAAAAAAAAAAAAAGAGTTCAAGGGCAACCTGGCCAAGAGCTGTCTCAAAACAAAAACCAAA------------------------ : 267
PVKW010036 : TTCCCTCTCTC------AAATAAATACATCAATCTTAAAAAAAAAAAAAAAAAAAAAAAAAAAAAGAGCCAGGTGTGGTGGTGCAT--------------------------------------------- : 263
PVKW010102 : CTCTCTCTCTC------AAATAAATAAGTTAAATATATTAAAAAAAAAAAAAAAAAAAAAAACCTTGGGATAAACCTAACAGACACTGA------------------------------------------ : 258
PVKW010009 : ---------------------AAATAAATAAAAAGATTAAAAAAAAAAAAAAAAAAAAAAAGCTCTTGCTTCTTCTTACATTGAGTTTCCTGTGTGTTACCATTCTATGGCAGA-A--------------- : 261
PVKW010061 : CTCTTTCTCTC--------------AAATAAAATAAGTTAAAAAAAAAAAAAAAAAAAAAAGCTTCTGCATGGCCAAAGCAACAATCATGAGAGTGAAGAGACACTC------------------------ : 262
PVKW010543 : ---------------------AAATAAGTTAACATGTTAAAAAAAAAAAAAAAAAAAAAGAGGGCTGGAGAGATTGGCTTAGCTTAGCAGTTAAGGCATTTGCCTACAAA--------------------- : 260
PVKW010024 : ----CTCCCTC----------AAATAAATAAAAGTTAAAAAAAAAAAAAAAAAAAAAGAGGGCTGGAGAGATGAGGGCTGGAGAGATGGCTTAGTGGT--------------------------------- : 260
PVKW010038 : ----------------CTAACAAATAAATAAAATAGAATATTTAAAAAAAAAAAAAAAAAAAAAGGAGAGAGGGGAGCTGGAGAGATGGCTTAGCAGTTAA------------------------------ : 255
PVKW010031 : ---------------------AAATAAATAAATATTTAAAAAAAAAAAAAAAAAAAAAAGGAGGTAGAGGTGAGCACAGAGCAAGAAGTTTTTTTTCTTTTTTGG-------------------------- : 258
PVKW010000 : ---------------------AAATAAATAATTTTTTTTTCTAAAAAAAAAAAAAAAAAAAAAAGAAAGAAAGAAAGTCACAATCAGACAGTGGAAGAACACATAGGAAGCTG------------------ : 262
PVKW010011 : ---------------------AAATAAATAAATAAATCCAAAAAAAAAAAAAAAAAAAAAGGAAAATTTGACTATTTCAATTAAACATGCAATGGACATCATTTTCTTA---------------------- : 261
PVKW010093 : ------------------AACAAATAAATATAAAATATCAAAAAAAAAAAAAAAAAAAAAAAAAAAAAAAAAAAAAAAAAAAAAAAAAAAAAAAGCAGATGGTTAAG------------------------ : 257
PVKW010020 : ---------------------AATAAATAGAATATTAAAAAAAAAAAAAAAAAAAAAAAAAAAAAAAAAAAAAAAAAAAAAAAAAAAAAAAAAAAAAAAAAAAAAAAAAAAAAAAAAA-------------------- : 264
PVKW010006 : CTTTCTCTCTC----------AAATAAATAAAATTTAAAAAAAAAAAAAAAAAAAAAAAACAAG------------------------------------------------------------------- : 272
PVKW010118 : -------------------------AAATAAATTAAAAAAAAAAAAAAAAAAAAACACCCTGAGACTACATAGTGAATTCCAGGTCAGCCCAGGTCAGCCAGGGCTAGAGCGAAACCCTAC---------- : 258
PVKW010159 : ----------------------AATAAATAAAATTAAAAAAAAAAAAAAAAAAAAAAAGAATTCTTAGATAAATGGGTTCCTGGGGCCTCACTGGTGGAAGCAGGTTGCAGGTGTGCCTGT---------- : 252
PVKW010074 : CTCTGTTGTTC-------------TCAATAAAATTAAAAAAAAAAAAAAAAAAAAACGGGCTGGAGAGATGGCTTAGCAATTAAGCATTTGCCT------------------------------------- : 263
PVKW010055 : ---------------------AAATAAATTAAATTACCTAAAAAAAAAAAAAAAAAAAAAGGTTCAGCACTCAGGAGGCAGAGCTGTAGGAGGATCACTGTGAGTTC------------------------ : 261
PVKW010009 : -----------------------ATAAATACCAAAAAAAAAAAAAAAAAAAAAGGAAAGGAAAGGAAAAGAATAGGGGGAAGGGCAAGGAAATAAAAATGCTCTGTTG----------------------- : 263
PVKW010011 : ----------------------AATACATAAAATTAAAAAAAAAAAAAAAAAAAAAAGGTAAGGCAGTGGCGCTATGAGATAACTCAGTTTGTAAAGTGCCTGATTTGTA--------------------- : 266
```

Terminator statistics:

	Number of Copies	Percentage
T>4	1	1.8%
T4, TVTTT	4	7.5%
TTT, TVTT	18	34.0%
TT, T	30	56.6%
Total	53	

Figure S20. Tail sequences of Dip_a copies with poly(A)$_{>20}$. PAS, AATAAA; terminators: TTTT, TVTTT, and T>4 ; rudimentary terminators: TTT, TVTT, and TT.
