# Supplementary material for: Analysis of SINE Families B2, Dip, and Ves with Special Reference to Polyadenylation Signals and Transcription Terminators
Source: Int J Mol Sci. 2021 Sep 13;22(18):9897. doi: 10.3390/ijms22189897 (PMC8466645; doi:10.3390/ijms22189897)
Supplement: Supplementary file 1 [file ijms-22-09897-s001.zip › Supplementary Vassetzky/SINE Dip. Figs S7-S22. Tables S6-S8/Fig S21.pdf]

```
DIP         : TCT----CTC-TCTCTCTC-------------------AAATAAATAAATAAAAAA--------------------------------------------------------------------------- : 208
PVKW010055 : CCT----CTT-TCTCTGTC-------------------AAATAAATTAATAAAAAATAAAAAAAAAATTAAAAAAAAAAAGAATCTGAGCTTTGGGCAGAGAGATAGC----------------------- : 262
PVKW010008 : TCT----CTC-TCTCTCTC-------------------AAATAAATAAGTAAAATAAAATAAAATAAAATAAAGAAAATCCAACTTTTTAAAAAAAAAAAGAAA--------------------------- : 258
PVKW010041 : TCT----CTC-CCTTTCTC-------------------AAATAAATAATTTAAAAAAAAAAAAAAAAAAGG------------------------------------------------------------ : 225
PVKW010209 : CCT----CTT-TCTCTATC-------------------AAATAAATAAATAAAAATAAAAATAATTTAAAAAAAAAAAACAAAAACAAAACAACAACAAAAAA---------------------------- : 262
PVKW010139 : TCT----ATC-ACTCTCTC-------------------AAATAAATAAATAAATAAGTAAATTTAAAAAAAAAAAAGATGCCCCCTTCCTAATTCCTG--------------------------------- : 263
PVKW010003 : TTT----CTC-TCTCTCTC-------------------CAATAAATAAAGAAAAATTAAAAAAAAAAACCCCAAAAAACAAAAAACAACAACAAAAAACCACATGTGCCTATTG----------------- : 263
PVKW010069 : -------CTC-TCTGTCTC-------------------TAATAAATAAAAAAATAAATAAAATCTTTAAAAAAAAAAAAAGCCGGGCGTGGAGGCGCACGCCTTGAATCTCAGCACTCGGGA---------- : 263
PVKW010259 : CCT----CTC-TCTGTCTC-------------------AAATAAATAAATAAATAAATAAATCTTAAAAAAAAAAAAAGAGTG------------------------------------------------- : 224
PVKW010046 : TCT----CTC-TCTCTCTC-------------------AAATAAATAAATAAAAATAAAATCTTAAAAAAAAAAAAAAAACAACGCCGGGCGTGGTGGTGCACACCTTTAAT-------------------- : 263
PVKW010028 : -------CTC-TTTGTCTC-------------------AAATAAATAAAAATAAAATCTTTAAAAAAAAAAAGAATGCTTACATTTAATTTCTGACCCTGTAATGAGCTATGGTTCTG-------------- : 263
PVKW010016 : TCT----TTC-CTTCTACC-------------------AAATAAATAAATAAAAATTTAAAAAAAAAAAGTGAAAGGAAGAGGAAATGTGCAATAAA----------------------------------- : 264
PVKW010042 : CCTCTGCCTC-ATTCTCTC-------------------AAATAAATAAATAAAAATATTTTAAAAAAAAAAAAAAAAGGAGAAGGTGAGGTGA--------------------------------------- : 262
PVKW010024 : CCT----CTT-TCTCTGTC-------------------AAATAAATAAATAAATAAATAAATATATGAGCCACCACGCCTGGTTTCAAAAAAAAAAATGTTAAAAG-------------------------- : 262
PVKW010042 : CCT----CTT-TCTGTCTC-------------------TAATAAATAAATAAATAAATAAAAATAAAATCCTTTAAAAAAAAAAAAGGAATGGTTTTCCTAAGGAA-------------------------- : 262
PVKW010044 : TCT--TTCTC-TCTCCCTC-------------------AAATAAATAAATAAAACATTAAAAAAAAAAAGAGGATCCAGGCTTGGCAGCACACAG------------------------------------- : 260
PVKW010001 : CCT----CTT-CTTCTCTC-------------------AAATGAATAAAAACTAAATGTTTAAAAAAAAAAAACAGAAAAAAGTTATTTGTATTATTTGTATTTTGTTTT---------------------- : 262
PVKW010013 : -------TTC-CCTCTCTC-------------------AAATAAATCAATAAAAATATTTAAAAAAAAAAAAAAAAAGGTTCTTATTTGCATAGCCTGATGGTCTGGGTTCAATTCTCCAG----------- : 262
PVKW010007 : -------CCC-ACTCTCTC-------------------AAATATATGAATTAAAAAAAAAAAAAAAAAAC-------------------------------------------------------------- : 211
PVKW010004 : -----------TCTCTGTC-------------------AAATAAATAAATAAATAAATAAATAAATAAATAAATTTTTTTTAAAAAAAAAAATAGCTTCTCACCCATAATATAAATGT-------------- : 263
PVKW010220 : TCT----TTC-TCTCTCTC-------------------AAATAAATAAAAATAAAATATTTTTGGTTTAATTTAAAAAAAAAAAAAAC-------------------------------------------- : 237
PVKW010028 : TCT----CTC-CCTCTCTC-------------------AAATAAATAAAAACAAAACTTTTTTTTTTAATTAAAAAAAAAAAAAGCCCAAGTCTATCACTTAGCATGCTAGTATGATTAG------------ : 261
PVKW010133 : CCT----CTT-TCTCTGTC-------------------AAATAAATGAAAAAAAATTAAAAAAAAAAAAACAGGCTTTTGACAAAGTTCAACATCCCTATGAAAAAAA------------------------ : 260
PVKW010074 : -------CTC-TCTCTCTC-------------------AAATAAGTAAAAAAAAAAATAAAAAAGAAAATACCAAATACTACAACAAAATGATAAAAATTAATATCCATCTT-------------------- : 258
PVKW010026 : TCT----TTC-CCTCTCTC-------------------AAATAAGTAAAATATATTTAAAAAAAAAAAAACAGTGAACAAAGAGAACAGGGCACTG------------------------------------ : 264
PVKW010030 : TCT----CTG-ACTCTCTC-------------------AAATAAATCAAACTTTAAAAAAAAAAAAAAGCTGGGATGGAGTGATGGCTTAGCAGT------------------------------------- : 261
PVKW010028 : TCT--CCCTCTCTGCTTGC-------------------AAATAAATAAATAATCTTTAAAAAAAAAAAAAAAAAGAAATGCAGACAAGAGTACAGGTGAAAG------------------------------ : 262
PVKW010194 : -------TGC-GCTCTCTC-------------------AAATAAATAAATTAAAAAAAAAAAAGCATATGGAGGAGTAGAACCCCCAGGGTCAAGTGCTCCATCTAGTCCCC-------------------- : 259
PVKW010029 : --------------------------------------AAATAAATAAATAAAAATAACATCTTTAAAAAAAAAAAAGAGCCGGGTGTGGTGGCGCACACTTTTAATCCCAGCACTCGGGA----------- : 263
PVKW010050 : -------TCC-TCTCTCTC-------------------AAATAAATAAACTATTAAAAAAAAAAAAGATATCAAGGGCTGGAGAGATGGCTTAGCAATTAAGGTGCTTGCCTGCAAAG-------------- : 263
PVKW010043 : ---------------TCTC-------------------AAATAAATTTTAAGTTAAAAAAAAAAAGATAGAGCTAGGTCAGGTGTGGTAGCACTAGGGAGTCAGAGGTAGGAAGATCACTGTGA-------- : 262
PVKW010018 : -------CCC-TTTCTGTC-------------------AAATAAATTAAAAAAAAAAAAATTAGGGTTAGAGAGATGGCTTAGTGGTTAAAGCACTTGCCTGCAAAGTCAAAG------------------- : 267
PVKW010009 : -------CTC-TCTGTCTC-------------------AAATAAAAATAAAATCTTAAAAAAAAAAAAAAAGCCAGGCTTGATGGTACATAAGCCTGAGATCTCAGCACT---------------------- : 265
PVKW010013 : TGG----CTG-TCACTCTC-------------------AAATAATAAATAAACAAACAAATTTTTTTTTTAAATATTAAAAAAAAAAAAAAAACAACA---------------------------------- : 269
PVKW010136 : TCT----CTG-TTGCTCTC-------------------AAATAAATAAATAAAAAATCAGCAAAAAAATATATTTAAAAAAAAAAAAA-------------------------------------------- : 269
PVKW010005 : TGT----ATC-TCTCTCTC-------------------AAATAAATAAATAAAAAATAAAATATTTTAAAAAAAAAAAAGTTCCATATGCATGTGCCACCATGTAC-------------------------- : 269
PVKW010591 : -------CTC-TTTCTCTC-------------------AAATAAATAATAAATAAAAATTAAAATATATTAAAAAAAAAAAGA---------------------C-------------------------- : 232
PVKW010050 : TCT----CTG-TCACTCTC-------------------AAATAAATAAATAAATAATATACAAAAAAAAAAAAAAAAGAAAGAAAAAAACAGACTG--TACTAGGTACC---------------------- : 268
PVKW010040 : TGT----CTG-TCGCTCTC-------------------AAATAAATAAATAAAAAATAAACAAAAAAATTAAAAAAAAAAAAAACAGAATTAAGGCCAGGTGTGGTGG------------------------ : 267
PVKW010060 : TGT----CTG-TTGCTCTC-------------------AAATAAATAAATAAAAATGAACAAAAATTAAAAAAAAAAAAAGGTAGTCGTCACTGGGGCATGTGTGTGCATCC-------------------- : 272
PVKW010027 : TGT----CTG-TCTCTCTT-------------------AAATAAATAAATAAAAGTAAACAAAAATTAAAAAAAAAACACCAAGATATAGTGGCTGGAGAGAT---------------------------- : 268
PVKW010008 : -------CTT-TCTTTCTCTACT---------------AAGTGAATAAATAAAAATAAAAACTTTAAAAAAAAAAAAGTGTGTGTCCCGCCGGGCGTGGTAGCGCA-------------------------- : 267
PVKW010027 : TCT----CTC-TCTCTCTC-------------------AAATAAATAAAATAAAATAAAAAGTTAAAAAAAAAAAGAATGTGGGGTTGACGCCGCGCATGGTGGCGCAAG---------------------- : 269
PVKW010000 : TCT----CTG-TTGCTCTC-------------------AAATAAATAAAAAAAAAAATTTAAGTAGATGGTGATTTTTAAGTCTCCTAATTCTAAATAACTGATGTGTTTG--------------------- : 278
PVKW010010 : TGT----TTG-TCACTCTC-------------------AAATAAACAAATTAAAAGAAACAAAAAAATTAAAAAAAAAAAGAATGGCATCTTTCTTGTTGGTCATTATAGCTCGG----------------- : 251
PVKW010011 : CGT----CTG-TTGCACTC-------------------AAATAAATAAATAATTAAAAAAAAAAAAAAAAACTATTTGAGGGTTAGAGGAATGGCTTAGCAGTTAAGGCACTTG------------------ : 268
PVKW010010 : --------------------------------------AAATAAATAAAAATTAAACAGCAAACTTAAAAAAAAAAAAAAGATGGGTATGGCCATGTAGTTTGGAGCTGCAAGGGGCAAAGACTTG------ : 258
PVKW010009 : --------------------------------------AAAAAATAAAACGTAAAAATAAAACTTAAAAAAAAAAAAAAAAAAGAACAGGAAGGACTGACCAACATACAGAAAAGTAAAGCCCTCAGT---- : 263
PVKW010035 : --------------------------------------AAATAAATAAATTTAAAAAAAAAAAAAAAAAAGCTGGGCATGGTGGCGCACGCCTTTAATCCCAGCACTAGGTAGGAGGATTGCTGT------- : 259
PVKW010002 : --------------------------------------AAATAAATACAATTAAAAAAAAAAAAAAAAAACAAGAACACCAAGGACTCATCCTTCTCCTTCATTATGAAGCCAGACTCAGGCATTTTG---- : 259
PVKW010108 : TAT----CTT-TCTCTCTC-------------------AAATAAATAAACAAAATATTAAAAAAAAAAAAAAAAGCTGAGGAAGCTGGGCGTGGTAGCACACAC---------------------------- : 265
PVKW010011 : -------CTC-TCGCTCTC-------------------AAATAAATAAATAAATAAAATATATTTAAAAAAAAAAAAAAACTCTTGTCAGCTGGTTACCAGCCACCCTTCCAATCTGC-------------- : 254
PVKW010000 : TCT----TTC-TCTCTCTC-------------------AAATAAATAAATAAATAATTAAAAAAAAAAAAAAGAATTTGAATTCTGATCCCATCCTCAGAAAACTTGCA---------------------- : 262
PVKW010001 : CCC----CTC-TCTGTCTC-------------------TAATAAATAAATAAATAAAAATAAAATCTTTTTTTTTTTTAAAAAAAAAAAAGAGTGTCCTAAAAAT--------------------------- : 258
PVKW010007 : CCC----CTC-TCTGTCTC-------------------CAATAAATAAACAAATAAAAATAAAATCTTTAAAAAAAAAAAGTGGGCCAAGAGTCTGGTG--------------------------------- : 257
PVKW010057 : --------------------------------------AAATATATATATATATATCTTTGAAAAAAAAAAAAGGGTAACTGGGATTACACCAGGTCAGTACCCACCAAAAAAGCCTGGTATGTAGGA---- : 263
```

```
PVKW010021 : -----------TATCTTTC-------------------AAATAACAATAAAAAAGTTAACAAGATTATTTAAAAAAAAAAAGAACAACAACAAAAAGGATTGGGTGAGGAGATGGC---------------- : 261
PVKW010007 : -------CTT-CCACTTTC-------------------AAGTAAATAAATAATTAAAAAAAAAAAAAAAAAGAGGGCTGGAGAGATGGCTTAATGGTTAAGGCACCCATCTGCAA-A--------------- : 258
PVKW010052 : -----------TGAGTCTC-------------------TAATAAATAAATAAAAATTAAAAAAAAAAAAAGAAAGTTGTGGAATCTTTAGGAGGTGGAACCTTGCTGGAGGAAGTTA--------------- : 256
PVKW010078 : CCCA-CATCT-CTCCTTGC-------------------AAATATATATATATATATATAAAATTAAAAAAAAAAAACAAATAATAGTTCATAT-------------------------------- : 263
PVKW010073 : --------------------------------------AAATAAATAATCAAAAAAAAAAAATTTTTTTTTTTTTTGGTTTTCCAAGGTAGGGTCTCACTCTAGCTCAGACTGACCTGGAATTCACTATGTAA : 262
PVKW010369 : -----------CAGCTTGC-------------------AAATAAATAAAAATATTAAAAAAAAAAA------------------------------------------------------------------ : 204
PVKW010025 : -----------CTGCTTGC-------------------AAATAAAAATTTTAAATTAAAAAAAAAAATACATAGCCGGGCGTGGTGGCACACACCTTTAATCCCAGCACT---------------------- : 258
PVKW010084 : --------TT-CTGCTTGC-------------------AAATGAATAATCAATAAAAACATTAAAAAAAAAAAAAAACCAGCCAGGTGTGGTGGTGCATGCCTTTAATCCCAGAGGTAGGAGGA-------- : 253
PVKW010006 : ---------------TTGC-------------------AAATAAATAAATTAAAAAAAAAAAAGAGCCAGGTGTGGTGCCTTTAATCCCAGCACTCAGGAGGCAGAGGTAGGAGGA-T--TGCTGTGAGTTC : 256
PVKW010008 : --------------------------------------AAGTAAATTAAAAAAAAAAAAAAAAAAAACCCACAAAGCATTTGACAATAATTGCTTTAATTCTCCGTATCTTCCCTCAAAGAGTTAAAA---- : 259
PVKW010001 : TTT----CTC-TCTTTCTC-------------------AAATAAATTAAATTTATTAAAAAAAAAAAAAAAGAATCTTGGGCTGGAGAGGTGGCTAAGCAATTA----------------------------- : 265
PVKW010008 : --------------------------------------AAATAAATAAAAATTTTTATTAAAAAAAAAAAAAGAAATGTGAATTAGTGGCTGGGGAGATGATTCAGTGGTTAAAGGGGCTTGCTC------ : 254
PVKW010067 : --------------------------------------AATTAAGTAAAATTAAAATAATGGTTAAAAAAAAAAAAAAAAGCAGCGCTAGGTGGCACACACCTTTAATCCCAGCCCTTGGAAGGCAGAGGTA : 251
PVKW010109 : TGC----CTC-TTTCTCTC-------------------AAATAAATAAATAAACAAACATTAATTTAAAAAAAAAAAAAAAAAGGAATGCTGTCTTTGAGACACGAAGTGGCTGT----------------- : 257
PVKW010113 : GCT----CTC-TCTCTCTCGCTCTCCCCCCTCCCCCGCAAATAAATAATTAAAAAAAAAAAAAAGTC------------------------------------------------------------------ : 266
PVKW010037 : -------------------------------------AAAATAAATAAATATTAAAAAAAAAAAGGTTCAAACATATGAGCAAACATAAGCGGGCAGTGCCCCCATGCCATTCCCATG--------------- : 248
```

Terminator statistics:

	Number of Copies	Percentage
T>4	8	11%
T4, TVTTT	15	21%
TTT, TVT	19	26.4%
TT, T	30	41.6 %
Total	72	

Figure S21. Tail sequences of random Dip_a copies with poly(A)$_{11-20}$. PAS, AATAAA; terminators: TTTT, TVTTT, and T>4; rudimentary terminators: TTT, TVTT, and TT.
