# Supplementary material for: Analysis of SINE Families B2, Dip, and Ves with Special Reference to Polyadenylation Signals and Transcription Terminators
Source: Int J Mol Sci. 2021 Sep 13;22(18):9897. doi: 10.3390/ijms22189897 (PMC8466645; doi:10.3390/ijms22189897)
Supplement: Supplementary file 1 [file ijms-22-09897-s001.zip › Supplementary Vassetzky/SINE Dip. Figs S7-S22. Tables S6-S8/Fig S8.pdf]

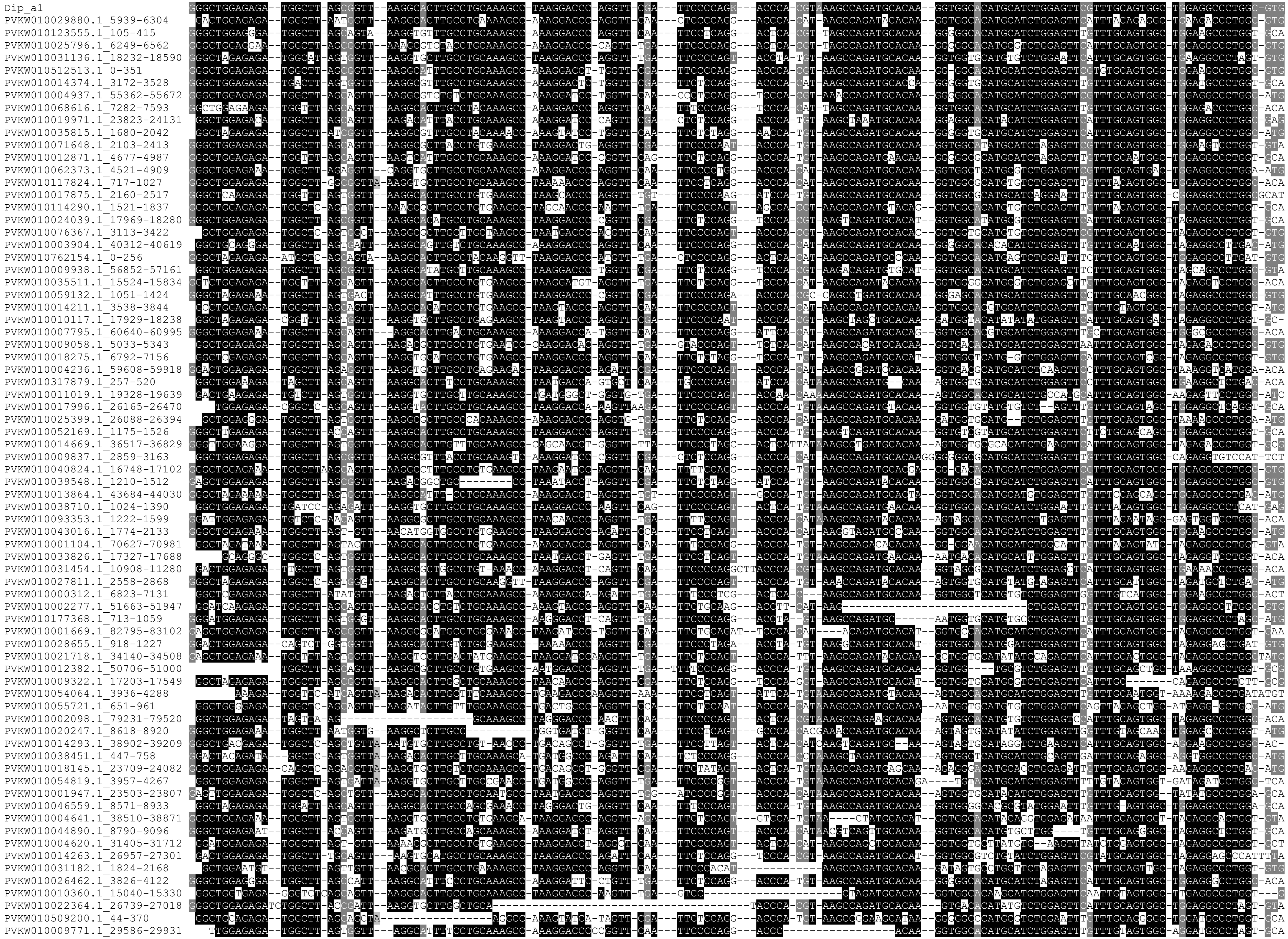
Dip_a1
PVKW010029880.1_5939-6304
PVKW010123555.1_105-415
PVKW010025796.1_6249-6562
PVKW010031136.1_18232-18590
PVKW010512513.1_0-351
PVKW010014374.1_3172-3528
PVKW010004937.1_55362-55672
PVKW010068616.1_7282-7593
PVKW010019971.1_23823-24131
PVKW010035815.1_1680-2042
PVKW010071648.1_2103-2413
PVKW010012871.1_4677-4987
PVKW010062373.1_4521-4909
PVKW010117824.1_717-1027
PVKW010017875.1_2160-2517
PVKW010114290.1_1521-1837
PVKW010024039.1_17969-18280
PVKW010076367.1_3113-3422
PVKW010003904.1_40312-40619
PVKW010762154.1_0-256
PVKW010009938.1_56852-57161
PVKW010035511.1_15524-15834
PVKW010059132.1_1051-1424
PVKW010014211.1_3538-3844
PVKW010010117.1_17929-18238
PVKW010007795.1_60640-60995
PVKW010009058.1_5033-5343
PVKW010018275.1_6792-7156
PVKW010004236.1_59608-59918
PVKW010317879.1_257-520
PVKW010011019.1_19328-19639
PVKW010017996.1_26165-26470
PVKW010025399.1_26088-26394
PVKW010052169.1_1175-1526
PVKW010014669.1_36517-36829
PVKW010009837.1_2859-3163
PVKW010040824.1_16748-17102
PVKW010039548.1_1210-1512
PVKW010013864.1_43684-44030
PVKW010038710.1_1024-1390
PVKW010093353.1_1222-1599
PVKW010043016.1_1774-2133
PVKW010001104.1_70627-70981
PVKW010033826.1_17327-17688
PVKW010031454.1_10908-11280
PVKW010027811.1_2558-2868
PVKW010000312.1_6823-7131
PVKW010002277.1_51663-51947
PVKW010177368.1_713-1059
PVKW010001669.1_82795-83102
PVKW010028655.1_918-1227
PVKW010021718.1_34140-34508
PVKW010012382.1_50706-51000
PVKW010009322.1_17203-17549
PVKW010054064.1_3936-4288
PVKW010055721.1_651-961
PVKW010002098.1_79231-79520
PVKW010020247.1_8618-8920
PVKW010014293.1_38902-39209
PVKW010038451.1_447-758
PVKW010018145.1_23709-24082
PVKW010054819.1_3957-4267
PVKW010001947.1_23503-23807
PVKW010046559.1_8571-8933
PVKW010004641.1_38510-38871
PVKW010044890.1_8790-9096
PVKW010004620.1_31405-31712
PVKW010014263.1_26957-27301
PVKW010031182.1_1824-2168
PVKW010026462.1_3826-4122
PVKW010010360.1_15040-15330
PVKW010022364.1_26739-27018
PVKW010509200.1_44-370
PVKW010009771.1_29586-29931

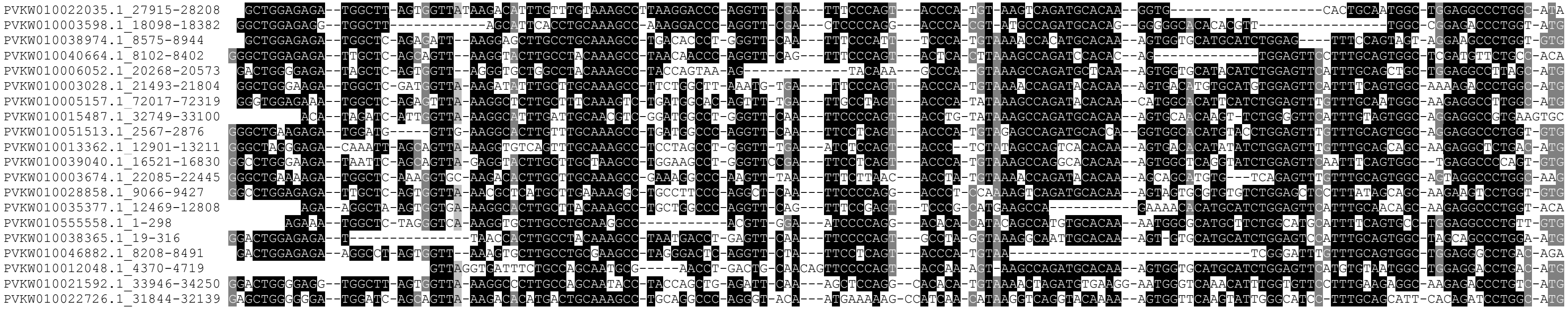

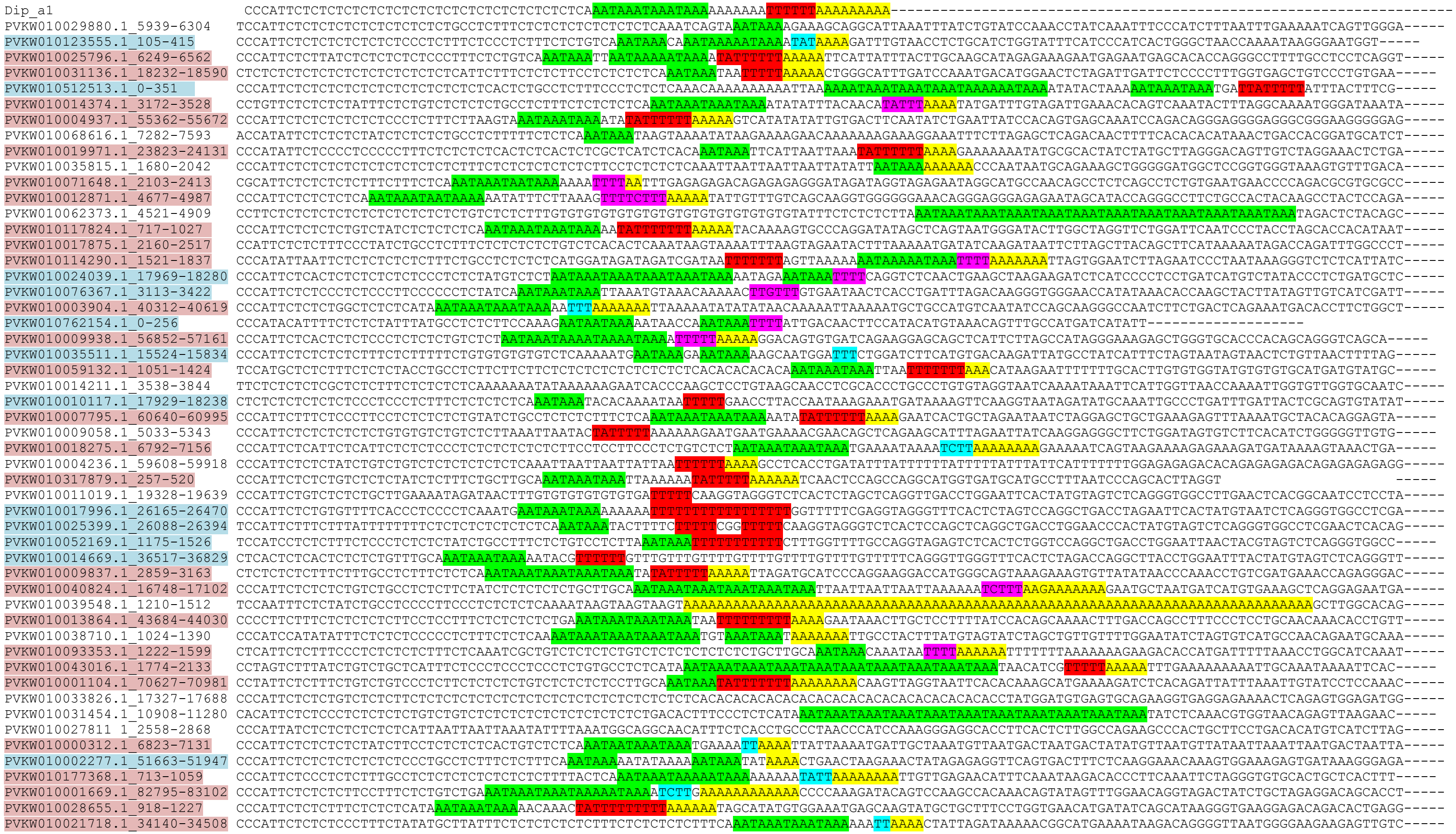

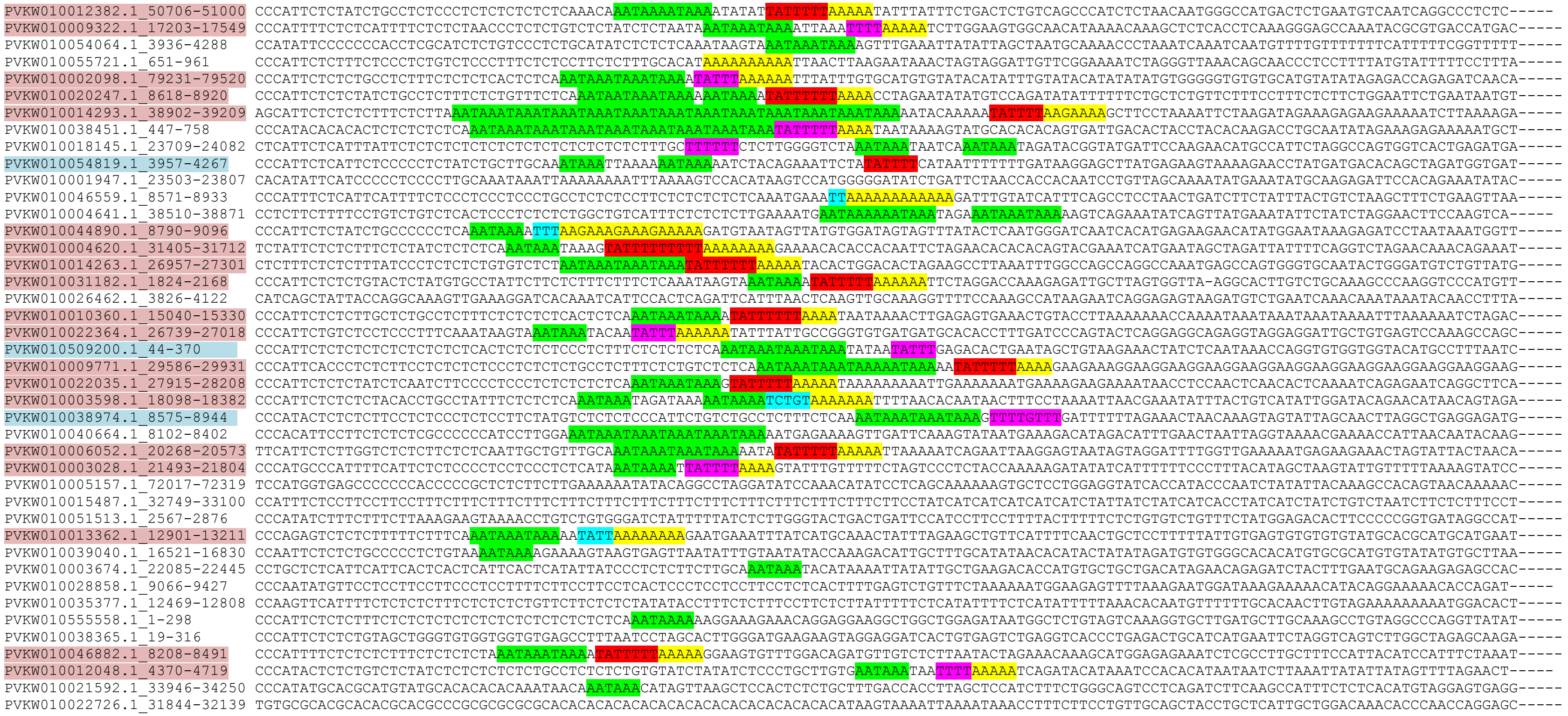

	Copy number	Proportion
T^+ copies	46	49%
Conventional T^+ copies	15	16%
T^- copies	33	35%
Total	94	

Figure S8. Random Dip_a1 copies from the *A. bullata* genome. Nucleotide sequences are highlighted as follows: PASs, strong pol III terminators, medium terminators, rudimentary terminators, and oligo(A). Conventional T^+ copies do not contain either a terminator or an oligo(A).
