# Supplementary material for: Analysis of SINE Families B2, Dip, and Ves with Special Reference to Polyadenylation Signals and Transcription Terminators
Source: Int J Mol Sci. 2021 Sep 13;22(18):9897. doi: 10.3390/ijms22189897 (PMC8466645; doi:10.3390/ijms22189897)
Supplement: Supplementary file 1 [file ijms-22-09897-s001.zip › Supplementary Vassetzky/SINE Dip. Figs S7-S22. Tables S6-S8/Fig S9.pdf]

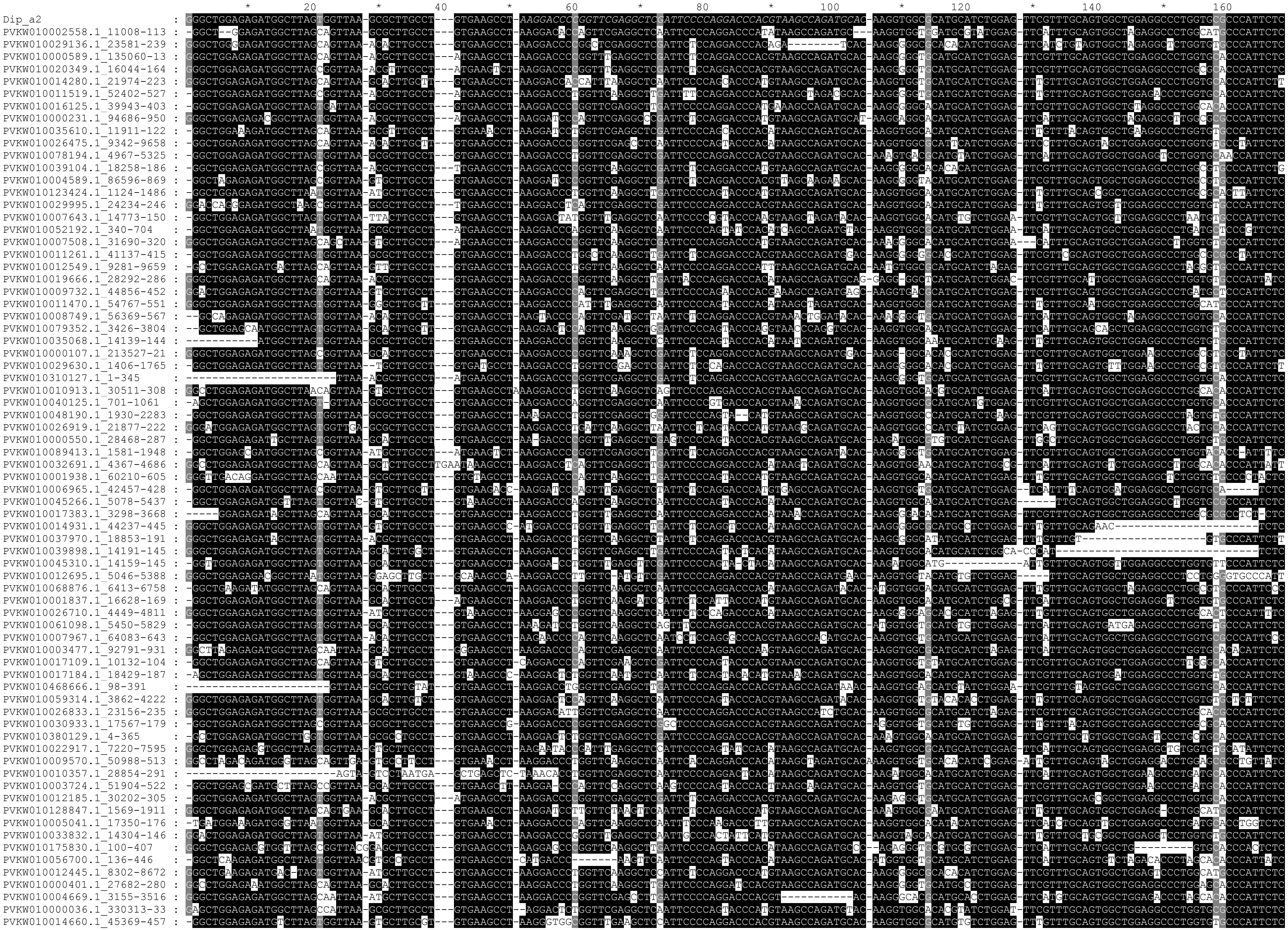

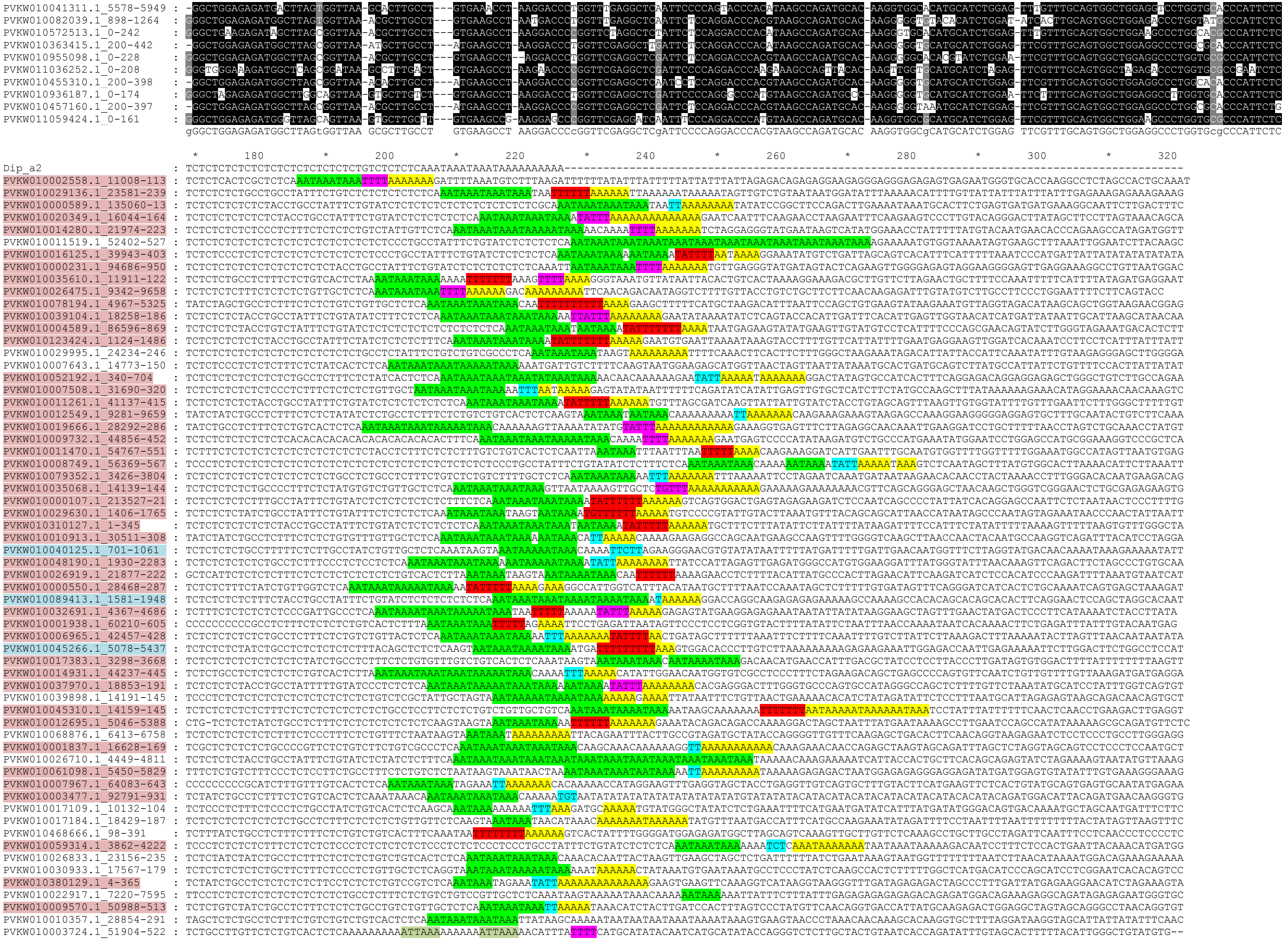

PVKW010041311.1_5578-5949
PVKW010082039.1_898-1264
PVKW010572513.1_0-242
PVKW010363415.1_200-442
PVKW010955098.1_0-228
PVKW011036252.1_0-208
PVKW010455310.1_200-398
PVKW010936187.1_0-174
PVKW010457160.1_200-397
PVKW011059424.1_0-161
180
200
220
240
260
280
300
320
Dip_a2

```
PVKW010012185.1_30202-305 : TCTCTCTCTCTCTCTACCTGCCTATTTCTGTATCTCTCTCTCTCAAATAAATAAATAAAATAAAACATAAAGGAGAGACTAATGGAGAGAGGGAGGGGATACAATGGAGATTGGAGTTCTAAAGGGGAATGGGGGAGGGGAGGGAAATATC
PVKW010128847.1_1569-1911 : TCTGTTTCTTTCCCTCTCTGTCTGTTGCTCTCAAATAAATAAATAAATAACATAAACAACAACCAAAAACAGCTATTCAAATTGAAAGAAATCAGGACAACTTTGGACACAGCAAAACAAGTGAGGATTGACAGACACAGAGTGGAAGGTTGA
PVKW010005041.1_17350-176 : TCTCTCTGCCTCTTTCTCTCTCTTTCTGTCTTGCTAATAAATAAAATAAAAATAAAACAAAAAACAATTAAGAATCTAGAGGATTCCATCAGAGTTGGCCTTGTTTGGTTCAGCTGACCAACATATATTTGTAGGATTCTATGACTTACTCTG
PVKW010033832.1_14304-146 : TTTCTCCCCCCCCCCCACCCTCTGTCTATTACTCTCAAATAAATAAAAATAAAAAATGTATATAAAGCATACGACATAGTCAGTGGTTACTAAATGTTCATTTCCTACTCATAGACTCTCTTACTTCTTCTTGCCTGAGAAAGGGTGTTCTA
PVKW010175830.1_100-407   : TCTCTCTTCCCCTCTCTGTCTTTAATAAATAAATAATAGATAAAAAATAAAATCAGAAAGGCTTTTGCCAAAGCCCTTGGTTTCCCCTGAGAAACAGATAGTAAGACCCTATTGCTGAAGACATCTCTTGCCTGAGAGGCAAGGTCACTGAGA
PVKW010056700.1_136-446   : TCTCTCTCTCTAATAAATAAGTAAAATAGAGACTTATTGAGAGGGGGAAGAAATATGATGGGAGGTGGATTTGTGAGGGGGAAAGTGGGCTTGGCGAGGGAATTATCATGGTTTATTGTCTATAATTATGGAAGCTATCAATAAAAATTTCTT
PVKW010012445.1_8302-8672 : TCTCTCTCTCTCTCTCTCTCTTTCTCCCTAATTATTCAGTTTCTCATGTGAAACAAGTCAAACAAAGGAACAAAATCTCTTTCTGGCTGAAATTGAACATGATTTGGGCATACTCTACTCACATAGTCATGTGGGCAAGGACATGAACAAACTC
PVKW010000401.1_27682-280 : TCTTTCTCTGCCTCTTTCTCTCTGTGTGTGTCTGTCACTTTCAAATAAATAAACAAATAAATAAATAAAATAAATAAATAAATAAATAAAAAAGAAAAAGAAAAAAAAAAGAATGAAAACTCC-TCGGTGGGTGTCAGAGGAGTAGAGGGTCCT
PVKW010004669.1_3155-3516 : TCTCTCTCTCTCTCTCCCTGCCTATTTCTGTATCTCTCTCTCTCTCTTTCAAATAAATAAATAAAAATATTAAAAGAAGTAGGGTCCAGGAAAGAGCGAGGGGTTTGGTGAGTGTGAGGACACTCCCTCCCTCCCTCCCTCAGTGACTATTAT
PVKW010000036.1_330313-33 : TCTCTCTCTGTCTCTGCCTCTTTCTCTGTCTGTCTGTCACTCTTAAATCAATAAACCAAAATAAACAATTTTTCTAAAAAAAGAATAATAATCATATCTTCACTCCATTTTCTTCTCTCCCTTGCCAAGGCAACTCTAAAGATGGGTCACTGG
PVKW010014660.1_45369-457 : TCTCTCTCCCTATCTCTCTACCTCTTTCTCTCTCTGTCACTCTCAAATAAATAAATAAATATAAATAAACAACAACAACCCCCCCAAAGTACTTTTAAGTAATTCAAAAAGCTACATTTCCTGCACATTATTTTAATGGTAAATATAGGACAA
PVKW010041311.1_5578-5949 : TCTCTCTCCCTCTCTCTCTCTCCATATATATATATATATATATATATATATATATATATATATATATACACACATACATACATACATACATACATACATACCTCTTTCTCTCTCTGTCATTCTCAAATAAATAAATTAAAAT
PVKW010082039.1_898-1264  : TCTCTCTAACTGCCTATTTCTGTATCTCTCTCTCTCAAATAAACAAATAAATAAATAAATAAAATATTAAAAAAAGAATAGAGACTATATAAAGACATTTCACACAAACAAAATTAGGAAAAGGTTTTTCTTCCAAGATAATGTAGTAGATAG
PVKW010572513.1_0-242     : TCTCTCTCTACCTGCCTATTTCTGTATCTCTCTCTCAAATAAATAAATAAAAATAAATCTTAAAAAATAAAATAAAATT-------------------------------------------------------------------------
PVKW010363415.1_200-442   : TCCCTCTCTCTCTCTCTCTCTCTCCCCCTGCCTATTTCTGTATCTCTCTCTCAAATAAATAAATAAATAATAAAATATTT------------------------------------------------------------------------
                            TCTCTCTCTCTCTCTCTCTcTCTcTcTCTcTcTCTctctctaaaaaAaaaaaaaAAaaAAaaaaaaaaaaaaaaaaaaaaaaaaaaa
```

	Copy number	Proportion
T^+ copies	48	62%
Conventional T^+ copies	4	5%
T^- copies	25	33%
Total	77	

Figure S9. Random Dip_a2 copies from the *A. bullata* genome. Nucleotide sequences are highlighted as follows: PASs, strong pol III terminators, medium terminators, rudimentary terminators, and oligo(A). Conventional T^+ copies do not contain either a terminator or an oligo(A).
