# Supplementary material for: Analysis of SINE Families B2, Dip, and Ves with Special Reference to Polyadenylation Signals and Transcription Terminators
Source: Int J Mol Sci. 2021 Sep 13;22(18):9897. doi: 10.3390/ijms22189897 (PMC8466645; doi:10.3390/ijms22189897)
Supplement: Supplementary file 1 [file ijms-22-09897-s001.zip › Supplementary Vassetzky/SINE Dip. Figs S7-S22. Tables S6-S8/Table S6.pdf]

Table S6
Multimeric SINEs containing Dip in *Allactaga bullata*

Structure	Number of units	Multimer length, bp	Copy number	Number of Dip sequences
B1-Dip-Dip-B1-Dip-B1-Dip-B1	8	1,498	5,060	20,240
B1-DIP-B1-DIP-B1-B1	6	1,071	13,996	27,992
B1-B1-Dip-B1-B1-Dip	6	1,081	7,846	15,692
Dip-Dip-Dip-B1-Dip	5	1,017	7,943	31,772
B1-Dip-B1	3	550	45,533	45,533
Dip-Dip-B1	3	602	36,668	73,336
Dip-Dip-Dip	3	649	19,797	59,391
B1-Dip-Dip	3	589	19,195	38,390
B1-B1-Dip	3	589	19,824	19,824
Dip-B1	2	381	91,922	91,922
Dip-Dip	2	427	63,606	127,212
B1-Dip	2	374	70,133	70,133
B1-B1	2	321	59,134	-
B4 (ID-B1)	2	289	64,798	-
monomeric				
B1	1	153	470,441	
Dip	1			621,437
ID	1		112,631	
