# Supplementary material for: Analysis of SINE Families B2, Dip, and Ves with Special Reference to Polyadenylation Signals and Transcription Terminators
Source: Int J Mol Sci. 2021 Sep 13;22(18):9897. doi: 10.3390/ijms22189897 (PMC8466645; doi:10.3390/ijms22189897)
Supplement: Supplementary file 1 [file ijms-22-09897-s001.zip › Supplementary Vassetzky/SINE Dip. Figs S7-S22. Tables S6-S8/Table S7.pdf]

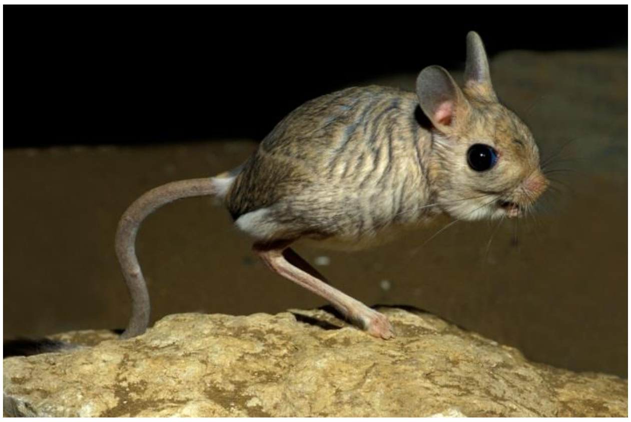

Gobi jerboa *Allactaga bullata*

Table S7

Incidence of TNT_mA_n patterns (≥5% & >1 sequence) **in**
Dip_a (487,241 sequences) and **Dip_b** (154,759 sequences) **banks**

	Dip_a			Dip_b		
	$A_{5\text{-}10}$	$A_{11\text{-}20}$	$A_{>20}$	$A_{5\text{-}10}$	$A_{11\text{-}20}$	$A_{>20}$
**T_2**	T_2 100% 65480	T_2 100% 11,438	T_2 100% 621	T_2 100% 25,261	T_2 100% 6,261	T_2 100% 467
**TNT**	T_3 59.0% 31,615<br>TAT 25.8% 13,857<br>TCT 8.1% 4, 364<br>TGT 7.1% 3,781	TCT 39.5% 289<br>TGT 31.8% 233<br>TAT 28.7% 210	TCT 52.3% 34<br>TGT 36.9% 24<br>TAT 10.8% 7	T_3 60.2% 13,698<br>TCT 23.4% 5,324<br>TAT 12.5% 2,842<br>TGT 4.0% 901	TCT 81.3% 610<br>TAT 9.6% 72<br>TGT 9.1% 68	TCT 87.3% 103<br>TGT 11.0% 13<br>TAT 1.7% 2
**TNT_2**	T_4 41.9% 17,435<br>TAT_2 39.2% 16,317<br>TCT_2 13.0% 5,418<br>TGT_2 5.8% 2,407	n/a	n/a	T_4 71.2% 6,201<br>TAT_2 16.8% 1,462<br>TCT_2 8.4% 730<br>TGT_2 3.6% 316	n/a	n/a
**TNT_3**	TAT_3 44.6% 15,646<br>T_5 30.9% 10,829<br>TCT_3 17.9% 6,275<br>TGT_3 6.6% 2,296	n/a	n/a	T_5 68.1% 3,408<br>TAT_3 17.7% 886<br>TCT_3 8.6% 430<br>TGT_3 5.6% 282	n/a	n/a
**$TNT_{\geq 4}$**	TAT_4 20.4% 13,113<br>TAT_5 18.6% 11,935<br>TAT_6 15.4% 9,930<br>T_6 8.8% 5,687<br>TAT_6 6.9% 4,411<br>T_7 5.5% 3,540<br>Others 24.4% 16,205	n/a	n/a	T_6 27.7% 1,633<br>T_7 16.8% 988<br>T_8 10.4% 616<br>TAT_4 10.2% 600<br>TAT_5 7.0% 414<br>Others 27.9% 1653	n/a	n/a

Terminator statistics:

Terminator	**Dip_a $A_{5\text{-}10}$** copy number	
TVT, TT	87,482	33.5%
TVTT, TTT	55,757	21.4%
TVTTT, TTTT	41,652	16%
$TVT_{\geq 4}$, $T_{\geq 5}$,	75,650	29%
Total:	260,541	

Terminator	**Dip_b $A_{5\text{-}10}$** copy number	
TVT, TT	34,328	52%
TVTT, TTT	16,206	24.6%
TVTTT, TTTT	7,799	11.8%
$TVT_{\geq 4}$, $T_{\geq 5}$	7,557	11.4%
Total:	65,890	

(V = A, C or G)
