# Supplementary material for: Analysis of SINE Families B2, Dip, and Ves with Special Reference to Polyadenylation Signals and Transcription Terminators
Source: Int J Mol Sci. 2021 Sep 13;22(18):9897. doi: 10.3390/ijms22189897 (PMC8466645; doi:10.3390/ijms22189897)
Supplement: Supplementary file 1 [file ijms-22-09897-s001.zip › Supplementary Vassetzky/SINE Dip. Figs S7-S22. Tables S6-S8/Table S8.pdf]

Table S8
Dip poly(A) in *Allactaga bullata*

	Dip (all)		DipA		DipB	
**A_{6-7}**	64.0%	228,490	72.8%	139,903	53.7%	88,587
**A_{8-9}**	17.9%	63,839	15.9%	30,581	20.2%	33,258
**A_{10-14}**	12.0%	42,825	8.7%	16,744	15.8%	26,081
**A_{15-19}**	4.1%	14,733	2.0%	3,811	6.6%	10,922
**A_{20-29}**	1.8%	6,415	0.5%	958	3.3%	5,457
**$A_{\geq 30}$**	0.2%	855	0.1%	119	0.5%	736
**total**		357,157		192,116		165,041

Copy numbers and percentage are shown.
