# Supplementary material for: Analysis of SINE Families B2, Dip, and Ves with Special Reference to Polyadenylation Signals and Transcription Terminators
Source: Int J Mol Sci. 2021 Sep 13;22(18):9897. doi: 10.3390/ijms22189897 (PMC8466645; doi:10.3390/ijms22189897)
Supplement: Supplementary file 1 [file ijms-22-09897-s001.zip › Supplementary Vassetzky/SINE Ves. Figs S23-S32. Table S9/Fig S23.pdf]

```
                         *        20         *        40         *        60         *        80         *       100         *       120         *       140         *       160         *       180         *       200         *
Ves_c      : ------------------------------GCCCTAG---CCGGTTTG--GCTCAG-TGGATAGAGCATCGGC----CTGCGGACC-----GAAGGGTCCCGGGTTCGATT-CCGGTC-AAGGG---CACGTACCTG--GGTTGCAGGCT-----------CTATCCCG----GGCCCTGGTC---------GGGGC---TCGTGCAGGAGG
NW00587106 : TGTGTACCTTAGTTTAAAATACAAGATTTAGCCCTAG---CTGGTTTG--ACTCAG-TCGATAGAGCATCGGC----CTGCAGACC-----AAAGAGTCCCGGGTTTGATT-CGGGTC-AGGGA---CACATACCTT--GGTTGTAGGCT-----CCTCC-CTGGCCCG----GGCCCTGGTC---------AGGGC---TTATGCAGGAGG
NW00587126 : TGTGCAGAGAAAATAAAAATGATCATGACAGCCTTAG---CCTGTTTT--GCTCAG-TGGATAGAGTGTTGGC----CTGCAGACT-----GAAGGGTTCCAGGTTTGATT-CCAGTC-AAGGG---CACGTACCTC--GGTTGCAGGCT-----------CAATCCCT----GGCCCTGGTC---------AGGCT---GCATGCAGGAGG
NW00587120 : CTAGTAAACAGCTTTAAAAAAGAAACTCCTGACCTAG---CCGGTTAG--GCTCAG-TGGATAGAGTGCCGGC----CTGTGGACC-----AAAGAGTCCCAGGTTTGATT-TCGGTC-AAGGG---TATGTACCTT--GGATGCAGGCT-----CTTCC-CCAGCCTG----GGCCCTGGTC---------AGGGC---TCATGCAGGAGT
NW00587423 : TTTCACCTAAAAATGTAAATGTCATCTCAAGCTCTGA---CCGGCTTG--GCTCAG-TGGATAGAGCGTTGGC----CTGCAGACC-----GAAGGGTCCCGGGTTTGATT-CAGGTC-AAGGG---CACGTACCTT--GGTTGCAGGCT-----CCTCC-CCGGCCCG----GGCCCTGATC---------AGGGC---TTGTGCAGGAGG
NW00587106 : GAATGAGCAGAAGTAAAAATTGACATCACAGGCCTAA---CCAGTTTG--GCTTAG-TAGGTAGAGCATCGGA----CTGTGGATC-----AAAGGGTCCTGGGTTTGATT-CCAGTC-AAGGG---CATGTACCTT--GGTTTCAGGCT-----CCTCT-CCAGCTGG----GGCCCTGGTC---------GAGGC---AACTGCAGGAGG
NW00587239 : TGCAAATAAGTTTAAAAATAACAATAAAGAGCCCTAG---TTGGTTTA--GCTCAG-TGGATAGAGCATCAGC----CTGCAGACC-----GAAGAGTCCCTGGTTTGATT-CTGGTC-AAGGG---CACATACCTT--GGTTGCAGGCT-----CCTCC-CCAGCCCC----GGACCTAGTC---------AGGGT---GCATGCAGGAGG
NW00587106 : ACAATAAAAAATCTAAAGATGAAGAAACCAGCCCTAG---TTACTTTG--GCTCAG-TGGATAGAGCATCGGC----CCATGGACC-----AAAGAATCCCAGGTTTGATT-CTGGTC-AAGGG---CACATACCT---AGTTGCAGGAT-----TCTCC-CTGGCCCA----GGCCCTGGTT---------GAAGC---TCATGCAGGAAG
NW00587535 : TGCATATTGCATGTTAAGAATTTACATCTTGCCCTAG---CCAG-TTG--GCTCAG-AGGATAGAGCATTGGC----CTGCGTACT-----GAAGGGTCCTGGGTTCGATT-CTGGTC-AAGGG---TACATACCTT--GGTTGCAGGCT-----CTTCC-CTGGTCTG----GGCCCTGGTC---------GGGGC---TCATGCAGGAAG
NW00587105 : TAAAAATAAGCCACTTTAAAGGAAATTCCTGCCCTAG---CCAGTTTG--GCTCAG-TAGATAGAGTGTCGAC----CTGCGGACC-----GAAGGGTCCTGGGTTCAATT-CAGGTC-AAGGG---CACATACCTC--GGTTGCAGGCT-----CCTCC-CCAGCCCA----GCCCCTGGTC---------AGGGC---TCCTGCAGAAGG
NW00587117 : GAGGCACAGAGCAATAAAAGTGAATAATGCACCCTAG---CCAGTTTG--GCTCAT-TGGATAGAGCATCGGC----CTGAGGACC-----AAAGGGTCCCAGGTTCGATT-CTGGTC-AAGGG---CACGTACCTT--GG-TGCAGGCT-----CTTCC-CCGGCCTG----GGCCCTGTTT---------GGGGC---GTGTGCAGGAGG
NW00587110 : AGAATTTGGCATCTAAATAGAGATGTGTGTGCCCTAG---CCGGTTTG--GCTCAG-CGAATAGAGCATTGGC----CTGCAGACC------GAGGGTCCCGGGTTCGATT-TCAGTT-AAGGG---CATGTACCTT--GGTTGCAGGCT-----CCTC--TTGGTCTG----GGCCCTGGTT---------GGGTC---TCGTGTAGGAGG
NW00587123 : TTTATTACTCAGAACTGTTTAAATATATCACCTGAGT---CCAGTTTG--GCACAG-TGAATAGAGTGTCGAC----TTGTGGACC-----AAAGGGTCCCGGGTTTGATT-CTGGTC-AAGGG---TACATACCTT--GGTTGCAGGCT-----CTT---CTGGGCCG----GACCGTGGTC---------AGGGC---ATGTGCAGGAGG
NW00587105 : TGCTGGAGGGGCTAAGAAATGTCATATCCTGCCCTAG---CCAGTTTG--GCTCAA-TGGATAGAGCCTCGGC----CTGCTCACC-----ACAGGGT-CCAGGTTTGATT-CTGATC-AAGGG---CATGTACCTA--GGTTGCAGGCT-----CCTCC-CTGGCCGG----GGCCCAGGTC---------AGGGC---TCATGCAGGAGG
NW00587140 : TGCTGGAGGGGCTAAGAAATATCATATCCTGCCCTAG---CCGGTTTG--GCTCAG-TGGATAATGCCTCGGC----CTGCCTACC-----ACAGGGT-CTGGGTTCAATT-CTGACC-AAGGG---CATGTACCTA--GGTTGCAGGCT-----CTTCC-CTGGCTGG----GGCCCAGATC---------AGCGC---TCATGCAGGAGG
NW00587106 : TGCTGGAGGGGCTAAGAAATGTCATATCCTGCCCTCG---CCGGTTTG--GCTCAG-TGGATAGAGCCTCAGC----CTGTGAACC-----GCAGGGT-CCAGGTTCGATT-CTGATC-AAGGG---CACATACCTA--GGTTGCAGGCT-----CCTCC-CTGGCCGG----GGCCTAGGTC---------AGGGC---TCATGCAGGAGG
NW00587106 : GTGAAGTGGTTGGTCAAGAATGGAGCCACAGCCCTGG---CCAGTTTG--GCTCAG-TGCATAGAGCATCGGT----CTGTGGACC-----AAAGGGTCCCAGTTTCAATT-CTGGTC-AAGGG---CACGTACCTC--AGTTGCAGGCT-----CCTTC-CCGACCCA----GACCCTGGTC---------AGGGC---TCATGCAGGAGG
NW00587572 : AAGAAAAAATAAAGTTAAAAATGAAAGACAGCCCCGG---CCAGTGTG--GCTCAG-TGGATAGAGCGTCGGC----CTGTGGACC-----AAAGGGTCCCGGGTTCGAAT-CTGGTC-AAGGG---CACGGACCTC--GGTTGCAGGCT-----CCTCC-CCGGCCCG----GGCCCTGGTC---------GGGGC---GTGTGCAGAAGG
NW00587263 : AATTCATCTTTAAGTAAAAAAACAAGACAGGCCCTGG---CCGGTGTG--GCTCAG-TGGATAGAGCGCCGGC----CTGCGGACC-----AAAGGGTCCCGGGTTCGACT-CCGGTC-AAGGG---CACGTGCTTT--GGTTGCAGGCT-----CCTCC-CTGGCCCT----CGCCCTCATC---------AGGGC---GCGTGGAGGAGG
NW00587112 : AAAAACTGTTAAAAAAAAATAGTTCTAAGAGCCCTAG---CTGGTTTG--GCTGAG-TGGATAGAAAGTCAGC----CTGCAGATT-----GAAGGGTCCTGGGTTCGATT-CCAGTC-AAGGG---CACATATCTT--GGTGGCAGGCT-----CCTCC-CCAGCCTG----GGCCCTGGCC---------AGGGT---GCATGCAGGAGG
NW00587113 : GAAAGTCCAGCGAATTAAAAAATGTATTTGGCCCTGG---CCAGTTTG--GCTCAT-TGGATAGAGCGTCGGC----CTGCGGACC-----CAAGGGTCCCGGGTTCGATT-CCGGTC-AAGGG---CACGTACCTC--GGTTGCAGGCT-----CCTCC-CCGGTCCG----GGGCCTGGCC---------AAGGC---TCTTGCAGGAGG
NW00587132 : CAGGGATCTCATGTTAAAAGTGAGGGTCACACCCTAG---CAGGCTTG--GCTCAG-TGGATAGAGCGTCGGC----CGGCAGACC-----CAAGTGTCCCAGGTTTGATT-CCGGTC-AAGGG---CACATACCCG--GGTGGCAGGCT-----CCTTC-CCGGCCCT----GGCTCTGGTC---------AGGGC---GTGTGCAGGAAG
NW00587123 : ATAATATGAGTTTTAAGATATAACAATCCAACCCTAG---CCAGTTTG--GCTCAG-TAGATAGAGTGTCAGC----CTGTGGACC-----GAAGGGTCCTAGGTTCAATT-CTGGTC-AAGGG---CATGTGTCTT--GTTTGTGGGCT-----CTTCC-CTGGCCCG----GGCCCTCATT---------GGGAC---ACTTGCAGGAGG
NW00587121 : CCCCAACTGCTGGAACTGAAAGGTTATTTGGCCCTAG---CCAGTTTG--GCTCAG-TGGATAGAGCGTCAGC----CTGCAGACC-----AAAGGGTCCCGGGTTCGATT-CCTGTC-AAGGG---CACATACCTC--AGTTGCAGGCT-----CCTCC-CTGGCCCG----GGCCTTGGCC---------CAGGC---TCATGCAGGAGG
NW00587222 : CAGAGACCCCATATCAAAAGTTTTTCTATAGCCCTGG---CTAGTGTG--GATCAG-TGGATAGAGCCTCAGC----CTGTGGATT-----GAAAGGTCCTGGGTTCGATT-TCCATC-AAGAC---CATGTGCCTT--GGTTGCAGGCT-----TCTCC-CCATCCTG----GGCCCTGGTC---------AGGGC---ATGTGCAGGAAG
NW00587114 : TATACTTAAAACTTACTTTAAGAAAAAATCCTGCTAG---ACTGTTTG--GCTCAG-TGAACAAAGCGTCAAC----CTTGGGACC-----AAAGGGTCCTGGGTTCGATT-CTGGTC-AAGGG---CACGTACCTT--GGTTGCAGGCT-----CCTCC-CTGGGCCG----GGCCCTGGTC---------AGGGC---ATGTGCAGGAGG
NW00587115 : TCAGCAGAATTAAAAGTCTCTTATTTTTTAGCTCTAG---CCTATTTG--GCTTAG-TGGATAGAGCATCGGC----CCATGGATT-----GAATGGTCCCAGGTTTGATT-CCGGTC-AAGGG---CACATACCTA--GGTTGCAGGCT-----TGATC-CCT---------GACCCTAGTC---------TGCGT---GGGTGCAGGAGG
NW00587477 : ATGTCATAACATCAAGAATAAACCAATTTCACCCTAG---CCAGTTTG--GCTCAG-TGGATAAAGTGT-GGC----CTGTGAACC-----AAAGGGTCCCAGGTTTGATT-CCAGTC-AAGGG---CACATACCTT--GGTTGCAGGCT-----CCTCT-CTGGCCCT----GGCCCTGGTT---------GGGGC---TCATGCAGGAGG
NW00587107 : GAAAGCAGTAGACATGAAGAACCAATATCAGCCCTTG---TCATTTTG--GCTCAG-TGGATAGAACATTGGC----CTATGGACA-----GAAGGGTCCCGGGTTTGATT-CCGGTC-AAGGG---CACGTACGTT--GGTTGCAGGTT-----CCTCCACTGGCCCG----GGTCCTGGTC---------AAGGC---CCGTGCAGGAGG
NW00587112 : GTAGGCAGACTTTTAAAATGGTCTAAATGAGCCCTCG---CTAGTTTG--GCTCAG-TGAATAGAGGGTCAGT----CTGCAGACT-----GAAAGGTTCTGGGTTCAATT-CTGGTT-AAGGG---CATGTACCTC--GGTTGCAGGCT-----CCTCC-CTGGCCCA----GGCCCTAGTC---------AGGGC---GTGTACAGGAGG
NW00587521 : AAACTCAGCAATCGAGATGAATAATATGTTGCCTTAG---CCAGTTTG--GCTCAG-TGGATAGAG-GTCACC----CTGCAGACC-----GAAGGCTTCCGGGTTCCATT-CTAGTC-AAGGG---CACATACCTC--AGTTGCAGGCT-----CCTCC-CTGGCCCGCCCAGGCCATGGTC---------AGGGC---GAGTGCAGGAGG
NW00587105 : AATCCGTTTGAAATCAAGGATGATGCCCTAGCCCTGG---CCGGTGTG--GCTCAG-TGGATAGAGCATCGGA----CTGCAGACC----AAAAGGGTCTCGGTTTCGATT-CTGGTC-AAGGC---CATGTGCCTC--GGTGGCAGGCT-----CTTCC-CCGGCCTG----GGCCCTGGTC---------AAGAT---GCGTGCAGGAGG
NW00587130 : TCTAAGTATCTATATAAATCAACAAGTCCAGCCCAAG---TCGGTTTG--GCTCAG-TGGATAGAGCGACGGC----CTGCGGACC-----AAAGGGTTCCCGGCTCGATT-CCCATC-AAGGG---CATGTACCTC--GGTTGCAGGCT-----CCTCC-CGGGTCAG----GGCCCTGGTC---------CAGGC---TCGTGCAGGAGG
NW00587112 : AACCCTGCCACTGGGCAGAGGAGGGGTCCAGCCCTGG---CCAGTGTG--GCTCAG-TGGATAGAGCATTGGC----CTGCGGACC-----GAAGGGTCCCCGGTTCGATT-CCGGTC-AAGGG---CACGTACCTC--AGTTGCAGGCT-----CCTCC-CTGGCCCA----GACCCTGGTT---------GGGGG---GTG-GCAGGAGG
NW00587122 : GGATGTTACATGCATAATGAGTATGTGACAGCCCTAG---CCCGTTTG--ACTCAG-TGGATAGAGCATTAGC----CTGCGGACT-----GAAGGGTCCAGGGTTCGATT-CTGGTC-AAGGG---CACATACCTT--GGTTGCAGGCT-----CCTCC-CTGGCCCA----GGCCCTGGTC---------GGGGC---GCATGTAGAAGG
NW00587174 : GGCTGAAAGCCCCAATGAAATAGTAAATTAGCCCTGG---CCGCTGTG--GCTCAG--AGATGGAGCATCGGC----CTGCGGACT-----GAAGGGTCCCAGGTTCGATT-CCGGTC-AAGGG---CATGTACCTG--GGTTGCAGGCT-----CCTCC-CCAACCTG----AGGCCTGGTC---------AGGGC---TTGTGCAGGAGG
NW00587129 : TTATTCATAACTGTAAAGAAAATCTCCCCAGCCCTAC---CCAGTTTG--GCTCAG-TGCATAGAGCATTGGC----CTGTGGACT-----GAAGGGTCCCGGGTTTGATT-CCAGTC-AAGGG---CACGTACCTC--GGCTGTAGGTT-----CCTCC-CTGGCTCG----GCCCCTGGTC---------AGGGC---TTGTGCAGAAGT
NW00587110 : TTTACCAGTTTATTTAAGAATATTATAAAGGCCCTAG---CTGGTTTG--GCTCAG-TGGATAGAGCGTTGAC----CTTGGGACC-----AAAGGGTCCTGGGTTCAATT-CTGGTC-AAGGG---CATGTACCTT--GGTTGCAGGCT-----CCTCC-CCAGCCCA---GGCCCCTAGTC---------GGGGC---TTGTGCAGGAGG
NW00587118 : GAGTGCAAAGAATAAGAGGAAAAGTGTGTCGCACTGG---CTGGTTTG--GTTCAG-TGGATAGAGCATCGGG----CTGTGAACC-----AAAGGGTCCCAGGTTCAATT-CTGGTC-AAGGG---CAAGTACCTT--GGTTGCAGGCT-----CCTTG-CAGGTTCG----GGCCCTGGTC---------AGGGC---TCCTGCAGGAGG
NW00587104 : GTATAAATTAATAATAAAAACTATGTGTTAGCCCTAA---CCAGTTTT--GTTCAG-TGGATAGAGCATTGGC----CTGTGGGCC-----AAAGGGTCTGGGGTTTGATT-CCAGTC-AAGGG---CACATACCTT--GGTTGCAGACT-----CTTCC-CTGGCCCA----GGCCCTGGTTGGGGG---GAGGGG---TGGTGCAGGAGG
NW00587106 : CTAAAATGTTCTCTAATAGAATAATTACCAGCCCTGG---TCGGTTTG--GCTCAG-TGCATAGAGCATTGGC----CTGTGGACT-----GAAGGGTCCCAGGTTTGATT-CCAGTC-AAGGG---CATGTACCTT--GGTTGCAAGCT-----CTATC-CT---------GGCCCTGGTC---------TGGGC---CCCTGAGGAGG
NW00587105 : CTAAGCATTCTTTAAATATTATGCATACAAGCCCTAG---CCGGTTTG--ACTCAG-TGGATAGAGCGTCAAC----CTGC-GACC-----AAAGGGTCCCAGGTTTGATT-CTGATC-AAGGG---CACGTACCTC--AGTTGCAGCCT-----CCTCC-CCGGCCTG----GACCCTGGTC---------GGGGC---TCATGCAGGAGG
NW00587110 : TTGTTGATAAGTGTTTAAAAAGAGTTTGGAGCCCTAG---CCAGTTTG--GCTCAA-TGGATAGAGCGTCAGC----CATTGGACT-----GAAGGGTCCCAGGTTCGATT-CTGGTC-AAGGG---CACGTACCTA--GGTTGCAGGTT-----CGATC-CCT---------GGCCTT-GTG---------GGGGC---ATGTTTGGAGG
NW00587104 : GAAAACAAATAACCATTAGAAAGTGGCAAAACCCTAG---CCAGTTTG--GCTCAG-TGGATAGAGTGTAGGC----CTGCAGGCT-----GAAGGGCCCCGGGTTCGATT-CTGGTC-AAGGG---CACATACCTA--GGTTGCAGGCT-----TGTTC-----------------CCTGGTC---------AGAAC---ATGTGTGGAGG
NW00587133 : GGGATAGCTTTGGAATATTAAAAAGTTGCAGAGTAGC---CTAGTTTG--GCTCAG-TGGATAGAGCATCAGC----CCTCGGACT-----CAAGGGTCCCAGGTTCGATT-CCAGTC-AAGGG---CACATACCTT---GTTACAGGCT-----CGATC-CCT---------GGCCCTGGTC---------GGGGC---GTGT---GGAGG
NW00587143 : GCCCTTATTTAAATTAAAAGGGAAGCTCAAGCCCTAG---CTGGTTTG--ACTCAG-TGGTTAGAGCATTGGC----ATGTGGGCT-----GAAGGGTCTCAGATTTTATT-CCAGTC-AAGGG---CATGTACCTC--GGTTGCAGGCT-----CACTC-CTC---------GGCCCATGTT---------GGGGT---GCGTGTGGAGG
NW00587303 : AAGGAAGCTTCAGGGAAAACGGAAAGTGTGGCTCTAG---CCGGTGTG--GCTCAG-TGGATAGAGCAGCAGC----CTTCGGACT-----GAAGGGTCCCAGGTTTCATT-CCAGTC-ATGGG---CACGTACCTT--GGTTGCAAGCT-----CAGTC-CCT---------GGCCCTGGTT---------GGGGC---ATGTGC-GGAGG
NW00587105 : TTTTTATTTTTTAAAAAAATCTTTGCTTCTGCCCTAG---CTGGCTTG--GCTCAG-TGGTTGGGCTCCAGC----CCTTGGACT-----GAGGGGTCCCAGATTCGATT-CCGGTC-AAAGA---CATGTACCTC--AGTTGCAGGCT-----CGATC-TCT---------GGCCCCAGTC---------AGGGT---ACCTGTGAGAGG
NW00587107 : AGTAAATTATTTTTAAAAAATCACATTCTAGCACCAG---CCAGTTTT--TCTCAG-TGGTTAGAGTGCCGGC----CCTCAAACC-----GAAGGGTCTCAGATTCAATT-CTGGTC-AAGGG---CACTTACCTC--AGTTGCAGGCT-----TGATC-CCT---------CGCCCTGGTC---------GGGGC---ACGTGTGGAAG
NW00587104 : AGTAGGTCCTTTAAAAGATTGGAGATTCCAGCCTTAG---CCAGTTTG--GTTCAG-TGGATAGAGCATTGAC----CTGTGGATT------GAGGCTGCCAGGTTCAATT-CTGGTC-AAGGG---CATGTACTTC--GGGTGCAGGCT-----CAGTC-CCT---------GAGCCTGGTG---------ATGCC---TCGTGTGGAGG
NW00587153 : ACAAAAGTGGAGGTAAAAATTACAGGCTATGCCCTAG---CCTGTTTG--GCTCAG-TGGTTGGAGCATCAGC----CTGCAGACT-----GAAGGGTCTTGGGTTCGATT-CTGGTC-AAGGG---CATGTACCTG--AGTTGCAGGCT-----CGAAT-CCC---------GGTCCCTACA---------GGGCA---CTGTGTGGAGG
NW00587120 : AGTTTCCTTGGCTATGAAACGGGGGTAAGAGCCCTCG---CCGGTTTG--GCTCAG-TGAACAGAGCATCAGC----CCTCGGACT---------------------GATT-CCGGTC-AAGGG---CACGTACCTT--GGTTACAGGCT-----TGATC-CCT---------GGCCCTGGTC---------AGGGG---GTGTGTGGAGG
NW00587109 : ACACACTCATGCATCCTATTTACTCTTTGGGCCCTAG---CTGATTTG--GTTCAA-TGGATAGAGTGTCTGC----CTACAGACT-----GAAGGGTCCTGGGTTTGATT-ATGGTC-AAAGG---CACATACCTT--GGTTGCAGGCC-----AGATC-TCT---------GGCCCTGGTT---------GGGGT---ACAAGCAGGAGG
NW00587119 : TTGCCATATTGCCTTAGAAAAAGAAAAGCAGCCCTAG---CCAGTCTG--GCTTAG-TGGATAGAGTATCGGC----CCGTAGAGT-----GAAGGGTCTCAGGTTCGATT-GGGGCC-AAGGG---CATGTACCTC--GGTTGCAGGTT-----CGATC-CCC---------GGCCCTCCTT---------GGGCT---GCATGGGGAGG
NW00587137 : AGCCAAGTCCTCCCATCTAAAGTGATGTCAGCCCTAG---CCAGTTTG--GCTCAA-TGGATAGAGCGTTGGC----CTGCAGACT-----GAAGGG--------TGGATT-CTGGTC-AAGGG---CACGTACCTT--AGTCGTAGGCT-----TAATC-CCC---------AGCCTCGGTT-----------GGC---CTGTGGGGAGG
NW00587134 : CATTCATCAGTTTTAAAAATATAAGATCATGCACTGG---CCTGTTTG--GCTCAG-TGGATAGAGCATCAGC----CCATGGACT-----GAAAGGTTCTAGGTTCAATT-CCAGTT-AAGAG---CACGTACCTT--GGTTGCAGGCT-----TGATC-TCT---------GGTCCCGGTT----------GGGC---GCCTATGGAGG
NW00587139 : AATGGACATGTGACTAAAATACTGGTTCCCACCCTAG---CTGGTTTG--GCTCAG-TGGATAGAGCATCGGC----CTGCAAACT-----GAAGGGTCTTGGTCCCATT-CTGGTC-AAGGG---CACAGACCTC--AGTT-TAGGCT-----TGATC-CCT---------GGCCTTGGTC---------AGAGC---GAGTGCAGGAGG
NW00587106 : TGTATTTTATTTTTAAGAAATTATTTTTCTGCCCTGG---CCAGTTTG--GCTCAG---------------GC----TTGCAGACT-----GAAGAGTCCCGAGTTCCATT-CTGGTC-AAGGG---CACGTACCTA--GGTTGCAGGTT-----TGATT-TCT---------GGCCCTGGTT---------GGGTT---ACGTGCAGGAGA
NW00587107 : GTGACTGAGTGGAAGCAGGAAGTGATTACTGCCCTCG---CTGGTTTG--GCTCAG-TGGCTACAGCA------------CAGACT-----GAAGGGTCCCGGGTTCGATT-CCCATC-AAGGG---AAAGTACCTT--GGTTGTAGGCT-----GGGCC-CCT---------GGCCCTGGTC---------CAGGC---GCGTGCGGAGG
NW00587106 : CGGCCCGCGGGCCGTAGTTTGCCCACGGCTAGTCTAG---CCAGTTTG--GCTCAG-TGGATAAAGCGTC------CCCTGCTACT-----AAAGAGTCCCGGGTTCGATT-GCAGTC-AAGGG---CACGTTC-----TGTTGCAAGCT-----------TCC---------AGCCTTGGTC---------GGAGC-----ATGTGGAGG
NW00587140 : CTATACAGCTATAAAAAAGAAGGATCTCTTGCCCTAG---CTGGTTTG--ACTCAG-TGGATAGAGCATCAGA----CTTCAGATT-----GAA-AGTCTTGGGTTCAATT-GGGGTC-AAGGG---CACGTACCTC--AGTAGCAGGCT-----------CCC---------GGCCCTGGTC---------AGGG----GAATGTGGAGG
NW00587186 : ATTAGGTTAAAGTGTTAAAATAAAAAAACATCCCTAG---TTGGTTTG--GCTCAG-TGGATAGAGCTTTGGC----CTGCGGACC-----AAAGGGTCCTGGG-TCAATT-CACATC-AAGGG---CACGTGCCTT--GGTTACAGACT-----CCTCC-CAG---------GCACTGGTCT---------GGAGT---GCCTACAGGAGG
NW00587126 : GAATTTAGTAAATCAAGAAAGCATGTTCTTGCCCTAA---CCGGCTTG--GCTCAG-TGGATAGAGCATCAGC----CGGAGGACT-----GAAAGGTCCCGGGTTCAATTTCCATTA-AAGGG---CACATACCTC--GATTGCAGGCT-----CCATC-CCT---------GGCCCTGGTT----------GGGC---CCATGCAGGAGG
NW00587104 : CTCAATGTTAGAAAAAAATATCTTATTCTAGCCCTTG---CCAGTTTG--GCTCAG-TCAATAGCGTGTCAGC----CTGTGGACC-----CAAGGGTC-CGGGTTTGATT-CTGATC-AAGGG---CACCTACCTT--GGTTGTAAGCA-----GCTCC-CCAGCCC-----AGGCCTGGTT---------GGGGC---TAGTGCAGAAGG
NW00587114 : TGAGCAGGGAGGTAAGAAATGCCCCCATCTGCTCTGG---CCAGTTTG--GCTCCG-TGGATAGAGCGTCAGC----CTGCAGACC-----GAAGGGTCCCAGGTTCGATT-CCGGTC-AAGGG---CAGGTACCTC--TGTTGCAGGCT-----CCTCC-CCAGCCTG---GGGCCCTGGTC---------AGAGT---GTGTGCAGGCGG
NW00587116 : GGAAGAGAGAGATAGAAACATCACTGATGAGCCCTAC---CTGATTTG--GTTCAG-TGGATAGTGCCTTGGC----CTGTGGACC-----AAAGGGTCCCAGGTTCAATT-CCAGTC-AAAGG---CA----------AGTTGCAAGCT-----CCTCC-CCTGCCTG----GGCCCTGATT---------GGAGG---ATGTGTAGGAGG
NW00587105 : TATATTTTAGTAAAAAAATTTGGGTCCAGCACCCTAG---CTGGTTTG--GCTCAG-TGGATAGAGCATTGGC----CCATGGACT-----GAGGGGCCCTGGGTTCAACT-CTGATC-AAGGG---CATGCACTTC--GGTTGCAGGCT-----TATCC-CTAG-----------CCCAGTT---------GGGGT---ATGTGTGGAGG
NW00587107 : AAAGACAAGATATATAAAAATGGGGGTGAAACCCTGG---CCAGTGTG--GCTAAG-GGATTAGAGTGTTGGC----CTGTACATC-----GAAGGGTCTTGGGTTCGATT-ACTGTC-AAGGG---CACATATCTG--GGTTACAGGTT-----CGATC-CCA---------AGCCCCAATC---------AGGGA---ACGTACGGAGG
NW00587117 : AATTAATGAGACATTAAAACATCAGTGGTTGCCTTGT---CCAGTT----GCTCAG-TGGTTAGAGCATTGGC----CAGCAGACC-----AAAGGGTTGTGGGTTCCATTCCCAGTC-AAGGA---CATGTACCTT--GGTTGCAGGCT-----CAATC-CCT---------GGCTCTGGTC---------AGGAT---GCTAATGGAGG
NW00587108 : TCAATAAATGGATGATAAAGATGTTATATAGCCCTGG---CCTATGTG--GATCAG-TGGTTAGAGCATAGGC----CCACCCACC--ACCAAAAGGTGGAGGGTTCTATTCCTGATC-AAGGG---CATGTGCCTG--GACTGCAGGTT-----CAATC-CCC---------AACCCCAATC---------AGGGT---GCATGTGGAGG
NW00587120 : GTCCAGAAGGCCAATTTAAAGACGTCTAGAGCCCTGG---CCAGCGTT--GCTCAG-TGGTTAGGGCATCGGC----CCACAGACC----CAAACAGTTGTGGGTCCTATTCCCAGTC-AAGGA---CACATACCTG--GATTGCAGGTT-----TGGTC-CCT---------GGTCCTGGTC---------GGGAC---ATGTGGGGAGG
NW00587122 : CCAATCATAACAAATTAAATTAGCCATCAAGCTCTGG---CCAGTGT---TCTCAG-TGGTTAGAGCATCGGC----CTGCAGACC-----GAAAGGTCTCAAGTTCAATTCCCAGTC-AAGGG---CACATACCTC--AGTTGTGGGCT-----GGATC-CCC---------AGAACCAATC---------AGGGT---GTGTGGGGAGG
NW00587109 : TCTGCCATTATTTTTCAAGAGTGCCATGCCATTCTTG---GCTGTGTT--TCTCAG-TGTTTAGAGTGTCGAC----CTGTGCACG-----AAAGGGTGGAGGGTTTGATT-CCAGTC-AAGGG---CATGTACCTG--GGTTTCAGGTT-----TGATC-TCC---------AGTCCCAGTT---------GGAGT---GCGTGTGCAAGG
NW00587259 : CACAACAGGGATTTAAAACAAAACAAAACAGTCCTGG---CCGGTGT---GCTCAG-TGGTTAGAGTGCCGGC----CTTTGCACC-----AAAGGGTTGCAGGTTCGATTCCTAGTG-AAGGG---CATGTACCTG--GGTTACAGGTT------GATC-CCT---------GGCCCCAGAT---------CAGGG---CAGTGTGGAGG
```

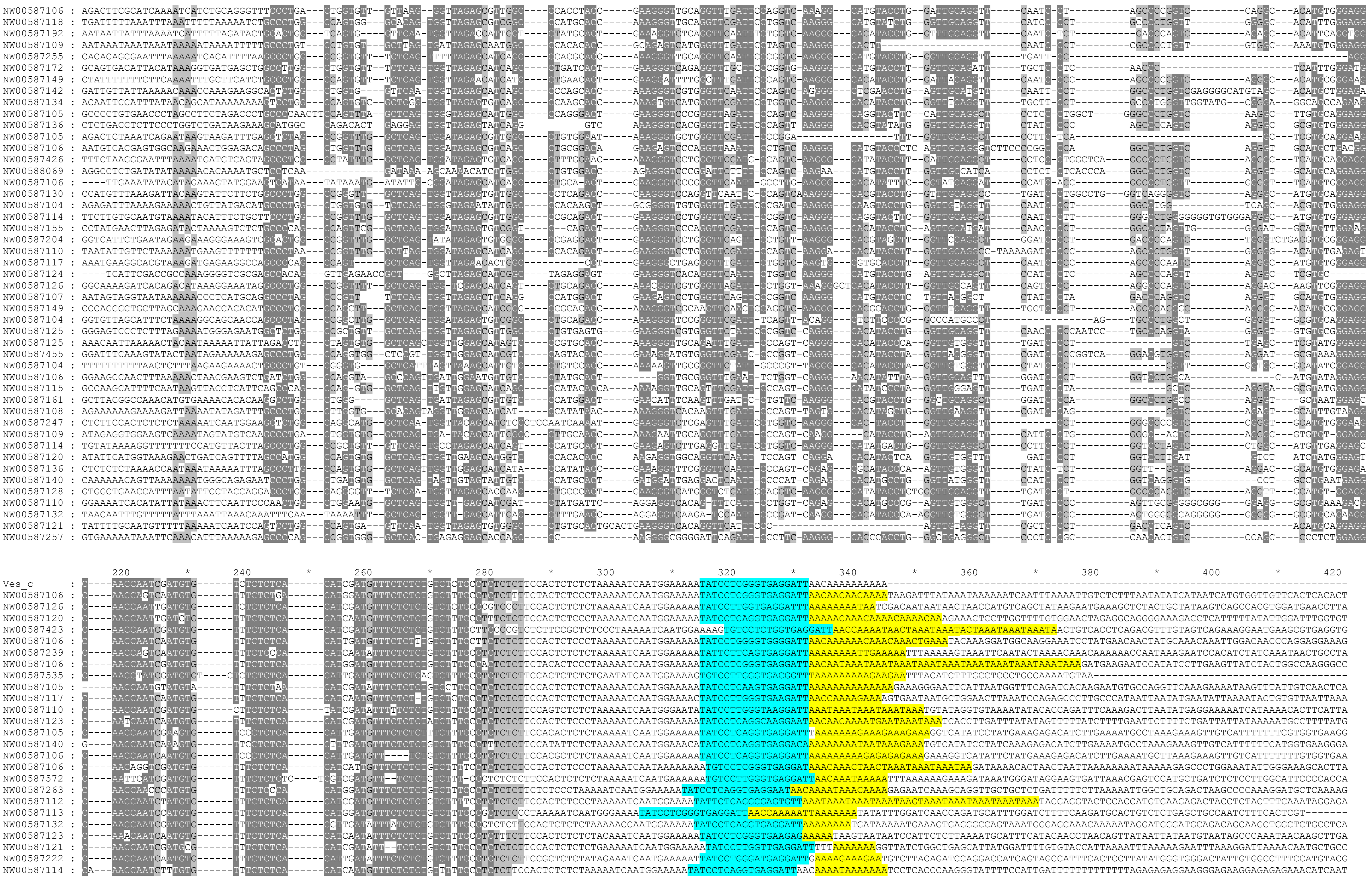

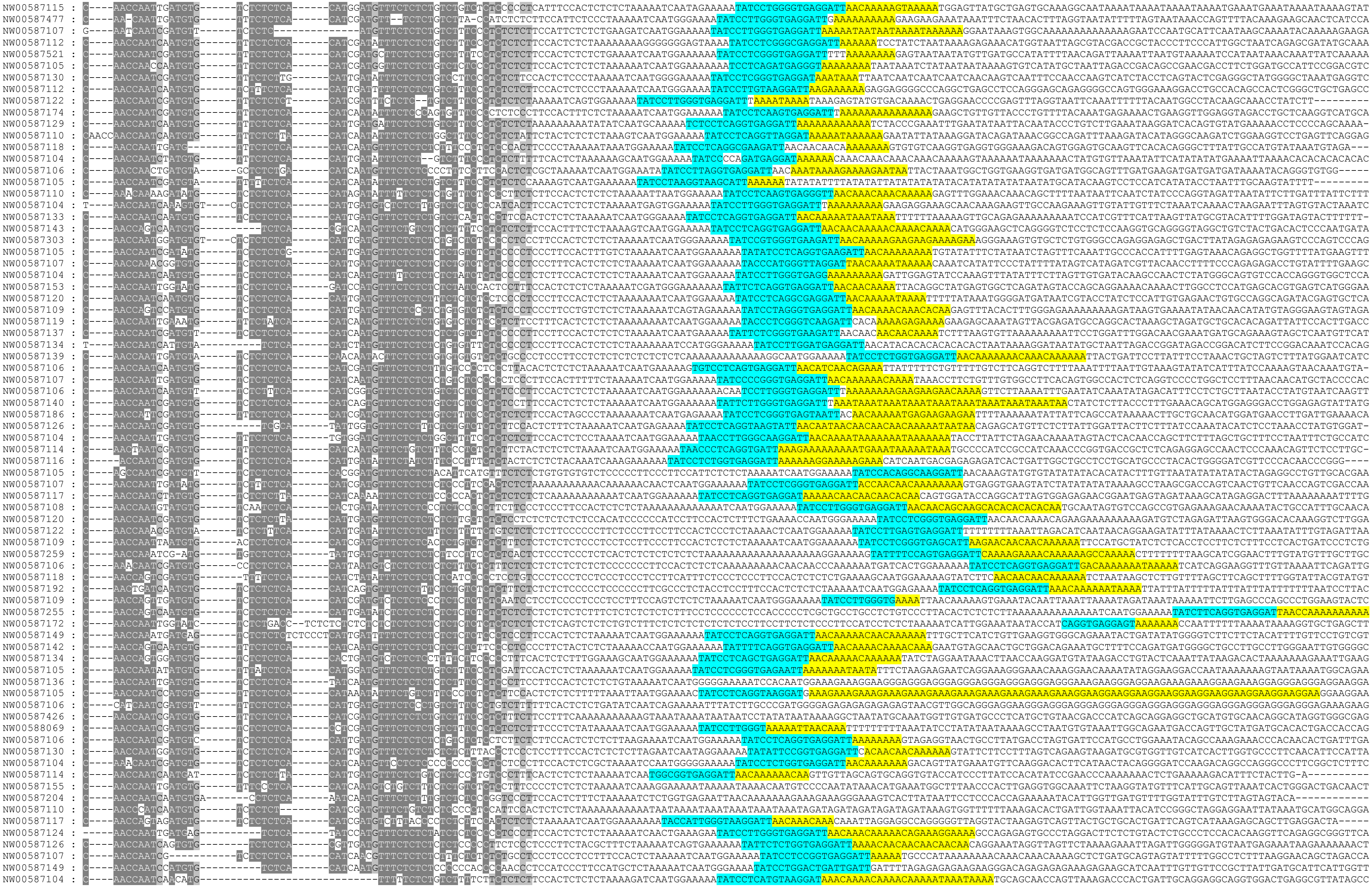
NW00587115
NW00587477
NW00587107
NW00587112
NW00587521
NW00587105
NW00587130
NW00587112
NW00587122
NW00587174
NW00587129
NW00587110
NW00587118
NW00587104
NW00587106
NW00587105
NW00587110
NW00587104
NW00587133
NW00587143
NW00587303
NW00587105
NW00587107
NW00587104
NW00587153
NW00587120
NW00587109
NW00587119
NW00587137
NW00587134
NW00587139
NW00587106
NW00587107
NW00587106
NW00587140
NW00587186
NW00587126
NW00587104
NW00587114
NW00587116
NW00587105
NW00587107
NW00587117
NW00587108
NW00587120
NW00587122
NW00587109
NW00587259
NW00587106
NW00587118
NW00587192
NW00587109
NW00587255
NW00587172
NW00587149
NW00587142
NW00587134
NW00587105
NW00587136
NW00587105
NW00587106
NW00587426
NW00588069
NW00587106
NW00587130
NW00587104
NW00587114
NW00587155
NW00587204
NW00587110
NW00587117
NW00587124
NW00587126
NW00587107
NW00587149
NW00587104

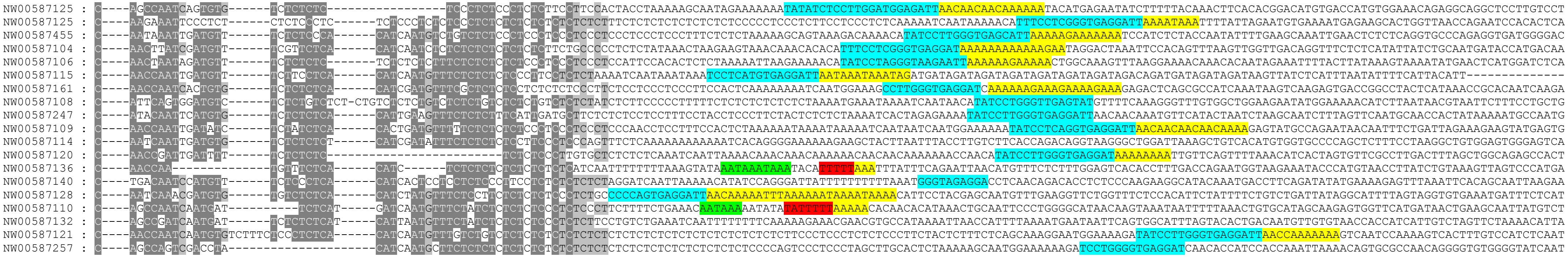

Figure S23. Random Ves_c copies from the *M. lucifugus* genome. Nucleotide sequences are highlighted as follows: specific 18-bp motif preceding an A-rich tail. There are PASs (green) and terminators (red) in two copies.
