# Supplementary material for: Analysis of SINE Families B2, Dip, and Ves with Special Reference to Polyadenylation Signals and Transcription Terminators
Source: Int J Mol Sci. 2021 Sep 13;22(18):9897. doi: 10.3390/ijms22189897 (PMC8466645; doi:10.3390/ijms22189897)
Supplement: Supplementary file 1 [file ijms-22-09897-s001.zip › Supplementary Vassetzky/SINE Ves. Figs S23-S32. Table S9/Fig S24.pdf]

```
                                   *        20          *        40          *        60          *        80          *       100     β signal    120
Ves_a            : ---------------------------GCCCTAACCGGTTTGGCTCAGTGGAT--AGAGCGTCGGCCTGCGGACTGAAGGGTCCCAGGTTCGA-TTCCGGTC-AAGGGCATGTA-CCTTGGTT
NW005871638.1_2  : AAATTGTGGGTTGAGAAAGTCACTCTGGCCCTAACCGGTTTGGCTCAGTGGAT--AGAGCGTCGGCCTGCGGACTGAAGGGTCCCGGGTTCGA-TTCCGGTC-AGGGGCATGTG-CCTTGGTT
NW005871332.1_6  : GGTGCAATGATCAAAACCAATACTATTGCCCTAACCGGTTTGGCTCAGTGGAT--AGAGCATCAGCCTGCGGACTCAAGGGTCCCAGGTTCGA-TTCCGGTC-AAGGGCATGTA-CCTTGGTT
NW005871213.1_2  : TCCATAACCAAAAAAAAAAAAAAATCAGCCAAGACCGGTTTGGCTCAGTGGAT--AGAGCGTCGGCCTGCGGACTGAAGGGTCCCAGGTTCGA-TTCCGGTC-AAGGGCATGTA-CCTTGGTT
NW005871780.1_1  : CAATAAGTATTACAAAGAGGACAATGGGCCCTAACCGGTTTGGCTCAGTGGAT--AGAGCATCAGCCTGCGGACTGAAGGGTCCCAGGTTTGA-TTCCGGTC-AAGGGCATGTA-CTTTGGTT
NW005871324.1_2  : TTTTTTTTTGATAAAGAGTTCAAGTGAGCCCTAACTGGTTTGGCTCAGTGGAT--AGAGCGTCGGCCTGCGGACTCAAGGGTCCCAGGTTCAA-TTCCGGTC-AAGGGCATGTA-CCTTGGTT
NW005871062.1_1  : CGAAAATGTAATTAAATGTGAGATGAGGCCCTAACCGGTTTGGCTCAGTGGAT--AGAGCATCGGCCTGCGGACTGAAGGGTCCCAGGTTCGA-TTCCAGTC-AAGGGCATGTA-CCTTGGTT
NW005871108.1_5  : AAAGCTAATGTTTAAAATAGATGTCTTGCCCTAACCGGTTTGGCTCAGTGGAT--AGAGCGTCGGCCTTCGGACTCAAGGGTCCCAGGTTCGA-TTCCAGTC-AAGGGCATGTA-CCTTGGTT
NW005871153.1_9  : AGGCTCACTTTAAAATTGCATCATACTGCCCTAACCGGTTTGGCTCAGTGGAT--AGAGCGTCGGCCTGCGGACTGAAGGGTCCCAGGTTCGA-TTCTGGTC-AAGGGCATGTA-CCTTGGTT
NW005871076.1_7  : AAGTAATCACTAAAAAATGAGACTTTAGCCAAAACCGGTTTGGCTCAGTGGAT--AGAGCGTTGGCCTGCGGACTGAAAGGTCCCGGGTTTGA-TTCCGGTC-AAGGGCATGTA-CCTGGGTT
NW005871066.1_7  : GAGTCCTCGAGTTAAGATGTGAGCATGGGCCAAACCGGTTTGGCTCAGTGGAT--GGAGCGTCGGCCTGCGGACTGAAGGGTCCCAGGTTCGA-TTCTGATC-AAGGGCATGTA-CCTTGGTT
NW005871054.1_8  : TAAAACAGTCATATAAAATATCAATCAGCCCTAACCGGTTTGGCTCAGTGGAT--AGAGCATCAGCCTGCGGACTCAAGGGTCCCAGGTTCGA-TTCCGGTC-AAGGGCATGTA-CCTTGGTT
NW005871136.1_4  : AGTCTTGTACAAAAAGAATCCTAAACAGCTGAAACCGGTTTGGCTCAGTGGAT--AGAGCGTCGGCCTGCGGACTGAGAGGTCCCAGGTTCGA-TTCCGGTC-AGGGGCATGTA-CCTGGGTT
NW005872463.1_7  : AAAAAAAAAAAAAAAAAAAATTAGAAGGCCGGAACCGGTTTGGCTCAGTGGAT--AGAGCGCCGGCCTGCGGACTGAAGGGTCCCAGGTTCGA-TTCCGGTC-GAGGGCATGTG-CCTGGGTT
NW005871333.1_4  : ATTATTTGTATATATTTTAAAATTTATGCCAAAACCGGTTTGGCTCAGTGGAT--AGAGCGTCGGCCTGCGGACTCAAGGGTCCCAGGTTCGA-TTCCGGTC-AAGGGCATGTA-CCTGGGTT
NW005871106.1_4  : TTACTGACTTATCAAAAAAATCTTCCCGCCGAGGCCGGTTTGGCTCAGTGGAT--AGAGCGTCGGCCTGCGGACTGAAGGGTCCCAGGTTTGA-TTCCGGTC-AAGGGCATGTA-CCTTGGTT
NW005871059.1_1  : AAAGATTAAAACAAAAAGAGACTTCATGCCAGCACCGGTTTGGCTCAGTGGAT--AGAGCGTCAGCCTGCGGACTGAAGGGTCCCGGGTTCGA-TTCCGGTC-AAGGGCATGTA-CCTTGGTT
NW005871121.1_4  : GAGTAGAGGGATAGGAAAACCTTTGACGCCGAAACCGGTTTGGCTCAGTGGAT--AGAGCATCGGCCTGCGGACTGAAAGGTCCCAGGTTCGA-TTCCGGTC-AAGGGCATGTA-CCTGGGTT
NW005871624.1_7  : GGCAGAGTGACCAAGAGGGTACACCTTGCCGAAACCGGTTTGGCTCAGTGGAT--AGAGCGTCGGCCTGCGGACTGAGGGGTCCCGGGTTCGA-GTCCGGTC-AAGGGCATGTA-CCTTGGTT
NW005871207.1_1  : CTATTTATTCTCTTAAGAAAACTTGCAGCCGAAACCGGTTTGACTCAGTGGAT--AGAGCGTTGGCCTGTGGACTGAAAGGTCCCAGGTTCGA-TTTCGGTC-AAGGGCATGTA-CCTGGGTT
NW005871210.1_1  : CAAATAGCGATATTAAAATATTTCTTTGCCGAAACTGGTTTGGCTCAGTGGAT--AGAGCGTCGGCCTGCGGACTGAAGGGTCCCGGGTTCGA-TTCTGGTC-AAGGGCATGTG-CCTGGGTT
NW005871354.1_8  : TCTCAATCTAAAATGAAGTAATAATGTGCCGAAACCGGTTTGGCTCAACTGAT--AGAGCGTCGGCCTGCGGACTGAAGGGTCCCAGGTTCGA-TTCCGGTC-AAGGGCATGTA-CCTGGGTT
NW005871413.1_9  : TCTAGAGGAAATAAAAGTAAGTCCTTCGCAGAAACCGGTTTGGCTCAGTGGAT--AGAGCGTCGGCCTGCGGACTGGAGGGTCCCAGGTTTGA-TTCCGGTT-AAGGGCATGTA-CCTGGGTT
NW005872128.1_3  : TGACTGAAATTAAAAATAATATACAGAGCCAAAACCGGTTTGGCTCAGTGGAT--GGAGCGTCGGCCTGCAGACTGAAAGGTCCCGGGTTTGA-TTCCGGTC-GGGGGCATGTG-CCTGGGTT
NW005871081.1_6  : GATTACATATTTTAAAATAATTTAGGTGCCGAAACCGGTTTGGCTCAGTGGAT--AGAGCGTCGTCCTGCGGGCTGAAGGGTCCCAGGTTGGA-TTCCAGTC-AAGGGCATGTG-CCTGGGTT
NW005871180.1_4  : TTGATATTATTTAAAACAAAAAAAGGGGCCCTAACTGGTTTGGCTCAGTGGAT--AGAGCGTCAGCCTGCAGACTCAAGGGTCCCAGGTTTGA-TTCCGGCC-AAGGGCATGTA-CCTTGGTT
NW005872499.1_5  : TTGATTCATGTCTTAAAATCCTAGAGAGCCCTAACCAGTTTGGCTCAGTGGAT--AGAGCATCAGCCTGCGGACTGAGGGGTCCCAGGTTCGA-TTCTGGTC-AAGGGCATGTA-CCTTGGTT
NW005871056.1_9  : CAGCATGACGAATAAAAACCCACAGTGACCAAAACCGGTTTGGCTCAGTGGAT--AGAGCGTTGGCCTGCGGACTGAAAGGTCCCAGGTTCGA-TTCCGGTT-AAGGGCATGTA-CCTTGGTT
NW005871108.1_3  : AATCTTTTGTTTAAAAAATGCTATACAGCCCTGACTGGTTTGGCTCAGTGGAT--GGAGCGTCGGCCTGCGGACTCAAGGGTCCTGGGTTCGG-TTCCGGTC-GGGGGCATGTG-CCTTGGTT
NW005871462.1_6  : ATATATCTGAAAATAAAATTCAATGATACCCTGACCAGTTTGGCTCAGTGAAT--AGAGCGTCAGCCTGCGGACTGAGGGGTCCCAGGTTCGA-TGCCGATC-AAGGGCATGTA-CCTTGGTT
NW005871275.1_1  : TATCCCAAAATAAATATAATTGATGGAGCCCTGACCGGTTTGGCTCAGTGGAT--AGAGCGTCGGCTTGCGGACTCAAGGGTCCCAGGTTCGA-TTCCGGTC-AAGGGCATGTA-CCTTAGTT
NW005871068.1_9  : CACGCGTGTCATAGGTTCGCCATCACTGCCCTAACCGGTTTGGCTCAGTGGAT--AGAGCGTTGGCCTGCAGGCTGAAAGGTCCCAGGTTCGA-TTCTGGTC-AAGGGCATGTA-CCTTAGTT
NW005871068.1_7  : TCCAACTTACTAATAAAAATGTATCAAGCCCTGGCTGGTTTGGCTCAGTGGAT--AGAGCATTGGCCTGCGGACTCAAGGGTCCCAGGTTCAA-TTCCGGTC-AAGGACAGGTA-CCTTGGTT
NW005871137.1_2  : ATGTCTTTTCTCAAGATTGGAAGACATGCCGTAACCGGTTTGACTCAGTGGAT--AGAGCGTAGGCCTGTGCACTCAAAGGTCCCAGGTTCGA-TTCCGGTC-AGGGGCATGTA-CCTTGGTT
NW005871641.1_3  : AAATGTATTTTAATAAACACTGCTTTTGCCGAAACCGGTTTGGCTCAGTGGAT--AGAGCGTCGGCCTGCGGACTGAGGGGTCCCAGGTTCGA-TTCCGGTC-AAGGGCATGTA-CCTGGGTT
NW005871055.1_1  : ATTCACTTGGCATTAAGAATAAACATGGCCCTAACCAGTTTGGCTCAGTGGAT--AGAGCGTCAGCCTGCGGACTAAAAGGTCTCAGGCTTGA-TTCTGGTC-AGGGGCATGTA-CCTTGGTT
NW005871095.1_5  : CCAAAAAGGATATCAAGAACTAGACCAGCCCTGACCAGTTTGGCTCAGTGGAT--AGAGTGTCGGCCTGCAGACTCAAGGGTCCCGGGTTCGA-TTCCAGTC-AAGGGCATGTG-CCTTGGTT
NW005871236.1_1  : ATGAGAGCCACTTAAAAATCCTATCTAGCCCTAACCGGTTTGGCTCAGTGGAT--AGAGTGTCGGCCTGCGGACTGAAGGGTCCTAGGTTCGA-TTCCAGTC-AAGGGCATGTA-CCTTGGTT
NW005871062.1_5  : CTACTATAATCAAAACAGCATGGTATTGCCCTGACCGGTTTGGCTCAGTGGAT--AGAGCGTCGGCCTGCAGACTCAAGGGTCCTGGGTTCGA-TTCCAGTC-AATGGCATGTA-CCTTGGTT
NW005871049.1_6  : GTTTTTATAGATTAAATTTAAAAATGAGCCAAGACCGGTTTGGCTCGGTGGAT--AGAGCGTCGGCCTGCGGACTGAAGGGTCCCGGGTTCGA-TTCCCGTC-AAGGGCATGTA-CCTTGGTT
NW005871051.1_3  : AGATGAAGGGCAAGAGGGAAATGAAGAGCCCTGACTGGTTTAGCTCGGTGGAT--AGAGCGTCAGCCTGTGCACTGAAGGGTCCCAGGTTCGA-TTCCAGTC-AAGGGCATGTA-CCTTGGTT
NW005871065.1_1  : AGTATTCGGTAAAGGTTAGTGGTTCTAGCCCTAACCAGTTTGGCTCAGTGGAT--AGAGCGTCAGCCTGCGGACTCAAGGGTCCCAGGTTCGA-TTCCGATC-AAGGGCATGTA-CCTTGGTT
NW005871058.1_9  : GATGAAGAGATTAAGAAGTACAGATTAGCCCTAACCGGTTTGGCTCGGTGGAT--ACAGCATTGGCCTGTGGACTGAAGGGTCCCAGGTTCGG-TTCCCATC-AAGGGCATGTA-CCTTGATT
NW005871064.1_1  : AGCACTATAAATAAGAATTGCTGAGAGGCCCTGACTGATTTGGCTTAGTGGAT--AGAGCATCGGCCTGCAGACTGAAGGGTCCCAGGTTCGA-TTCCAGTC-AAAGGCATGTA-CCATGGTT
NW005871050.1_2  : ATAAATGCATCATTTAATCAGAAAAGTGCTGAAACCGGTTTGGCTCAGTGGAT--AGAGCGTCGGCCTGCGGACTGAAGGGTCCCAGGTTCGA-TTCCAGTC-AAGGGCATGTA-CCTTGGTT
NW005871077.1_8  : CACCATGGGATCAAGAAGACCAATTTAGCCCTGACTGGTTTGGCTCAGTGGAT--AGAGCGTCGGCCTGCAGACTCAAGGGTCCCAGGTTCGA-TTCCAGTC-AAGGGCATGTA-CCTTGGTT
NW005871117.1_2  : CTGCACTTAATTATAAAACCCAGCTTTAGCCCTAACTGGTTGGCTCAGTGGAT--AGAGTGTCGGCCTGCGGACTGAAGGGTCCCAGGTTCAA-TTCCAGTC-AAGGGCATGTA-CCTTGGTT
NW005871344.1_1  : GTAAAGGAATCAAGAAGCCTTAGTTTAGCCCTAACCGGTTTGGCTCAGTGGAT--AGAGCGTCAGCCTGCAGACTGAAAGGTCCCAGGTTCGA-TTAAGGTC-AAGGGCATGTA-CCTTGGTT
NW005871473.1_6  : GTAATAAAAATACATATAAAAAAATAGCCCTAGCTGGTTTGGCTCAGTGGAT--GGAGCGTCAGCCTTCGGACTGAAGGGTCCTGGGTTCAA-TTCCAGTC-AAGGGCATGTA-CCTTGGTT
NW005871982.1_2  : ATAAAAGATTTTTTAAAAGTCCCTGAAGCCCTGGCTGGTTTGGCTCGGTGGAT--AGAGTGTCGGCCTGTGGACTCAAGGGTCCCAGGTTCGA-TTCCAGTC-AAGGGTATGTA-CCTTGGTT
NW005875779.1_4  : CAGGAGGCAGTTAAAAAGGCAATTCTGGCCCTGACCGGTTTGGCTCAGTGGAT--AGAGCGTCGGCCTGTGGACTGGGGGGTCCCGGGTTCGA-TTCCGGTC-AAGGGCATGTA-CCTTGGTT
NW005871833.1_1  : CACATCTTGCTAATGAAAAGAAAATAGGCCCTGACCGGTTTGGCTCAGTGGAT--AGAGCGTTGGCCTGTGGACTGAAGGGTCCCAGGTT-GA-TTTCGGTC-AAGGGCATGTA-CCTTGGTT
NW005871115.1_1  : CAAGATAGTATAAAAAACAATGTTTCTGCCCTAATTGGTTTGGCTCAGTGGAT--AGAGCGTCGGCCTGCGGACTCA--------AGGTTCAA-TTCCGGTC-AAGGGCATGTA-CCTTGGTT
NW005871152.1_1  : TCTTTGCTCATTAAGATGTTTATTCCAGCTGTAACCGGTTTGGCTCAGTGGAT--AGAGCGTTGGCCTGCGGACTGAAGGGTCCCAGGTTCCA-TTCCGGTC-AAGGGCATGGA-CCTTGGCT
NW005871048.1_5  : GCAACTTACATTTAAATATTTTATTCATCCCTGACCGATTTGGCTCAGTGGAT--AGAGCATCGGCCTGCAGACTGAAGGGTCCCAGGTTTGA-TTCTGGTC-AAGGGCATGTA-CCTTGTTT
```

```
NW005871311.1_1 : GAATTTGTAGTTTCAAAAATGAAATGCGCCCTGACCGGTTTGGCTCAGTGGAT--AGAGCGTCGGCCTGCAGACTCAAGGGTCCCAGGTTCGA-TTCCGGTC-AAGGGCATGTG-CCTTGGCT
NW005871051.1_2 : AGTGGTATTACACATGTCAGAAGGATAGCCCTAACTGGTTTGGCTCAGCAGAT--AGAGCTTCGGACTGAGGACTGAAGGGTCCCAGGTTTGA-TTCTGGTC-AAGGGCATGTA-CCTTGGTT
NW005871155.1_2 : TTGTGACCCAACCATAAAATTATTTTTGCCCTGGCCGGTTTGGCTCAGTAGAT--GGAGTGTCGGCCTGTGGACTGAAAGGTCCCAGGTTTGA-TTCAGGCC-AAGGGCACGTA-CCTTGGTT
NW005872153.1_3 : TTTTTTGAGATTTAAAAAGGTCAAATACACTGAACCGGTTTGGCTCAGTGGAT--AGAGCGTCTGCCTGCGGACTCAAGGGTCCCGGGTTCGA-TTCCGGTC-AAGGGCATGTA-CCCTGGTT
NW005871148.1_4 : TATGATTCTGCCTTAAAAAGGAATGAAGCCAAAACCGGTTTGGCTCAGTGGAT--AGAGCGTCAGCTTACGGACTGAAAGGTCCCA-GTTCGA-TTCCGGTC-AAGGGCATGTA-CCTGGGTT
NW005871082.1_2 : ATTGACTCCATAAAAGTACCCATTGGCGCCCTGACTGGTTTGGCTCAGTGGATA-AGATCATCGGCCTGTGGACTGAAAGGTCCCAGGTTTAT-TTCTGGTC-AAGGGCATGTA-CCTTGGTT
NW005871337.1_1 : ATCAAGCCATTTAAAAAGACATGGATAGCTCTAACCGGTGTGGCTCAGTGGAT--AGAGCATCAGCCTGTGGACTGGGGGGTCCCAGGTTTGA-TTCTGGTC-AAGGGCATGTA-CCTTGGTT
NW005871065.1_1 : AAAAAGAAGCATTAAAATAGGACGTCTTGCCGAACCGGTTTGGCTCAGTGGAT--GGAGCGTTGGCCTGTGGACTG-AGGGTCCAGGGTTCGA-TTCCGGTC-AGGGGCATGTG-CCTTGGTT
NW005871165.1_3 : TATAAGAACTAATCAAGAGCTGAGCCAGGCCGAACCGGTTTGGCTCAGTGGAT--AGAGCGTCGGCCTGAGGACTGAAGGGTCCCAGG------TTCCGGTC-AAGGGCATGTA-CCTGGGTT
NW005871073.1_3 : ATTGCATTCAGTAAAAAGCAGAAGCATGCCCTAGCTGGTTTGGCTCAGTGGAT--AGAGCGTTGTCCTGCAGACTGAAGGGTCCCAAGTTCAA-TTCTAGTC-AAGGACACATG-CTCGGGTT
NW005871563.1_1 : CAACATTAGTGTCTGGATTAAAACGTGTCCCTAGCCGGTTTGGCTTAGTGGAC--AGAGCGTTGGTCAGCAGACTGAAGGGTCCCGGGTTCAA-TTCTGGTC-AAGGGCACATG-CCCAGGTT
NW005871431.1_9 : TTTTCATGATACAAAGATGCCGCTGGAGCCCTGGCAGGTTTGGCTCAGTGGAT--AGTGCATCGACCTGTGGACTGAGGGGTCCCAGGTTCGA-TTCCGGTC-AAGGGTATGTA-CCTTGGCT
NW005871069.1_9 : ATAGGCCTACCTCAAAAAACCAGAAAAGCCCTGATCGGTTTGGCTCATTGGAT--AGAGCGTCGGCTTGGGGACTGAAGGTTCCCCGGTTCGA-TTC--GTC-AAGGTCATGGA-CCTTGGTT
NW005871112.1_3 : ATTTCTCTTGTCTAAATATGCTGAGTAGCTCTAACCGGTTTGGCTCAGTGGAT--AGAGCATCGGCCTGCGGACTGAAGGATCCCGGGTTCGA-TCCTGGTC-AGGGGCATGTA-CCTTGGTT
NW005871096.1_5 : AAATTTCCAACAAAATGTGTATGTATAGCCCTGACTGGTTT-GCTCAATGGAT--AGAGCATCGGCCTG-GGACTAAAGGGTCCCGGGTTCGA-TTCTAGTC-AAGGGCATGTA-CCTTGGTT
NW005871270.1_3 : AGGTGGGAATAAAAAAGGAAGGAATGGGCCCTGACTGGTTTGGCCGAGTGGAT--AGAGCGTCGGCCTGCAGACTCAAGGGTCCCAAGTTCGA-TTCTGGTC-AAGGGCATGTA-CCTTGGTT
NW005871767.1_1 : AATGCTTATAAAATCAACTGGTATATAGCCCTAACCGGTTTGGCTCAGTGGAT--AGAGCATCGGCCTGCGGACTCAAGGGTCCCAGGTTTGA-TTCTGGTC-AAGGGCATATA-CCTTGGTT
NW005871176.1_3 : GTAAATACGTTTAAATACCGTAAGGCAGCCAAAACCGGTTTGGCTCAGTGGCT--AGAGCGTCGGCCTGCGGACTGAAAGGTCCCAGGTTCGA-TTCCGGTC-AAGGGCATGTA-CCTGGGTT
NW005871060.1_1 : CAGTGTTATGTTTAAAAAGTACTACCAACCCTAACCAGTTTGGCTCAGTGGAT--AGAGCATTGGCCTGTGGACTGAAGGGTCCCGGATTCAA-TTCTGGTC-AAGGGTATGTG-CCTTGGAT
NW005871091.1_4 : CTGACTTTAAAATAGGGAGGTTAGCCCCAGCTGGCTGGTTTAGCTCAGTGGAT--AGAGCATCAGCCTGTGGACCGAAGGGGACCTGCTTCGA-TTCTGGTC-AAGGGCACATG-CCCAGGTT
NW005871534.1_3 : ACCTTTTATTTTAAACCTTAAGGTCTAGCCCTGGCTGGTTTGACTCAGTGGAT--AGAGCATTGGCCTGTGGG-AAAAGGGTCCCAGGTTCAA-TTCCAGTC-AAGGGCACCTG-CCTGGGTT
NW005871346.1_5 : CTTTTCAACATTCAAGATACAAATCTTGCCCTAGCTGGTTTGGTTCTGTGGAT--ACAGCAGTGGCCTGTGAACTGAAAGGTCCCTGGTTCGG-TTCTGGTC-AAGGGCACATG-CCCAGGAT
NW005871075.1_2 : CATTTGTTCATTCAAGAATATTTCTTGGCCCTAGTGGGTTTGGCTCAGTGGAC--AGAGCCTTGGCCTGTGGACTGAAGGGTCCCGGGTTAGA-TTCCGGTC-AAGGGCACATG-TCCAGGTT
NW005871048.1_1 : TCAGGATAGATTAAAATTCTACCTTTTGTCCTAGTTGGTTTGGCTCAGTGGAT--AGAGTGTCGGCCTGTGGACTGAAGGGTCCCAGGTTCGA-TTCCGGTC-AAGGGTACATG-CCCGGGTT
NW005871088.1_8 : CCTTTACCTGAAAAAAAAAATTTTCCAGCCCTAGGTGGTTTGGCTCTGTGGAT--AGAGCATTGACCTGTGGACTGAAGAGTTCCCAGTTTGA-TTCCAGCC-AAGGGCACATG-CCCGGGTT
NW005871126.1_9 : TATTCACATGAAAAGTAAGATTAGACAGCCCTAGATGGTTTGGCTCGGTGGAT--AGAGCATTGGCCTGTGAATGGAAGGGCCCCGGGTTCAA-TTCTGGTC-AAGGGCACATG-CCCGGGTT
NW005871419.1_8 : CAATGCCAGAATAAAATAAGCAAATAGAGCCCTAGCTGGTTTGGCTCAGTGGAT--AGAGCGTCAACCTACGGACTGAAGGGTCCTGGGTTCGA-TTCCAGTC-AAGGGCACATG-GTTGGGTT
NW005871067.1_7 : CATTTTTCTTTATTTGATAAACATGTTGCCCTAGCTGATTTGGCTCAGTGGGT--AGGGCATCAGCCTGTGGACTGAAGGGTCCTGGGTTTGA-TTCTGGTC-AAGGGCACATG-CCCAGGTT
NW005871349.1_1 : AATTATTCTTATTAAAATGTGTAATTTGTCCTAGCCGGTTTGGCTCAGTGGAT--AGAGCGTCGGCCTGAGGACTGACAGGTCCCGGGTTTGA-ATCTGGTC-AAGGGCACATG-CCCAGGTT
NW005871109.1_5 : TGGCTCACGAGCAGCAGTTTGCCGACCACTGCAGCTGGTTTGGCTCAGTAGAT--AGAGCGTTGGCCTGTGGACTGAAGGGTCCCCAGTTCGA-TTCTGGTC-AAGGGCACATG-CCTGGGTT
NW005874004.1_1 : TAATTATACTTAAAAAACAAGTGACAGTCCCTGGCTGGTTAGACTCAGTGGGT--AATGCATCGGCCTGGGGACTGAGGGGTCCCGGGTTTTA-TTCCGGTT-AAGGATAGAGG-CCCAGGTT
NW005871206.1_6 : GAATAATGTTTTAAAATGCGTAACAGGGCCCTAGCCAGCTTGGCTCAGTGGAT--AAAGCGTCAGCCTGCGGACTGAAGGGTCCCAGGTTCAA-TTCTGGCC-AAGGGCACATG-CCTGGGTT
NW005871123.1_4 : ATGAAGTCTTTAAGAACAGGATTGCTTGCTCTAGTTGGTTTGGCTCAGTGGCT--AGAGCTTCAGCCTGAGGACTGAAGGATCCTAGGTTCAA-TTCTGGTC-AAGGACACTTG-CCTGGGTT
NW005871055.1_6 : ACAGTGATTTTTAAACACAGTCTGGTAGCCCTAGCTGCTTTGGCTCAGTAGAT--AGAGCATCAGCCTGAAGACTGAAGTTTCCCAGGTTCAA-TTCCAGCC-AAGGGCACATG-CCAGGGTT
NW005871068.1_9 : GGAACAGGTAATAAAGAATACACTAGGGCCCTAGCTGGTTTGGCTCAGTGGAT--TGAGCATTGGCCTGCAGACTGAAGGGTCCCAGGTTCGA-TTCCCGTT-CAGGGCATGTA-CCTGGGTT
NW005871516.1_4 : TGGTGTCCTTATAAAAGGGGACATTTGGCCGTAGCTGATTTGGCTCAATGGAT--AGAGCATCGGCCTGCGGACTGAAGGGTCCCGGGTTCGA-TTCCAGTC-AAGGGCACGTG-CCCAGGTT
NW005871085.1_3 : TAGGTGCAGATAAAAAATAGCAGCTTTAGCCCTAGCTGGTTTGGCTCAGCGGAT--AGAGCATCAGCCTGCGGACTGAAGGGTCCCAGGTTTGA-TAACGGTC-AAGGGCACATG-CCTGGGTT
NW005871063.1_5 : TGAGTAGATTAAAAAACAGTGGTACATGCCCTGACTGGTTTTGCCCAATGGAT--AGAGCGTCAGCCTGCGGACTGAAGGGTTCTGGGTTCCA-TTCCGGTA-AAGGGCACATG-CCCAGGTT
NW005871093.1_5 : ATGAGCAAAATAAAAAGTAGCAGGTAAGCCCTAGCCGGTTTGGCTTAGTGGAT--AGAGCCTTGACCTTCAGACTGAAAGGTCCCGGGTTCAA-TTCTGGTC-AAGGGCACATG-CCCGGGTT
NW005881170.1_2 : GCTAAGGGATTAAGCAAACATTAATTAGCCCTAGCTGGCTTGGTTTGGTGGAT--AAAGCGTCGGCCTGCGGACTGAAGAGTCCTGGGTTTGG-TTCCAGTC-AAAGGCACATG-CCCAGGTT
NW005871097.1_6 : TTTACAAGAAAAAAAAAAGTTGCTCAGGCCCTAGCTGGTTTGGCTCAGTGGAC--AGAGCATCGGCCTGTGGACTGAAAGGTGCTGGGTTCAA-TTCCAGTC-AAGGGCACATG-CCCGGGTT
NW005871464.1_5 : CTGGACAAGTGGTTGTAGCTTTGCAGAAGTGAAAATCCAATGGCTCAGTGGAT--AGAGCATCAGCCTGTGAACTGAAAGGTCCCTGGTTCGA-TTC-GGTC-AAGGGCACATG-CCTGGGTT
NW005871049.1_2 : ATAAATTTAATTTTAAAAAATGGTAAAGCTCTCGCTGGTTTGGCTCAGTGGGT--AGAGCACT-GCCTGCAGACTGAAGGGTCCTGGGTTTGA-CTCTGGTC-AAAGGCACATG-CCTGAGTT
NW005871111.1_3 : GAAACTGAGAATCAAAACTGATAAATAGCCCTAGCTGGTGTGGCTCAGTGGAT--AGAGCGTTGGCCTTCGGACTGAAGGTTCCCAGGTTCGA-TTCCGGTC-AAGGGCATATG-CCTGGGTT
NW005871143.1_1 : CAAATACAGTGTTATTTAAAAAGTACCGCCCTGGCAGGAATGGGTCAGTGGGT--AGAGCATCGGCCTGCAGACTGAAGAGTCCTGAGTTTGA-TTCCGGCC-AAGGGCACATG-CCTGGGTT
NW005872372.1_5 : ACCTAAGTTAAACAACATTTACGATGCGCCCTCGCTGGTTCAGCTCAGTGGGT--AGAGCGTCGGCCTGTGGACTGAAGGGTCCTAGGTTCGA-TTCCAGTC-AAGGACACAAGCCCCCGGTT
NW005871909.1_5 : TGCAGAATTCACATTAAACAGCATCATGCCCTAGCCGGTTTGGCTCAGTGGAT--AGAGCGTCAGCCTGC-GACTGAAAGGTCCCAGGTTCTT-TTCTGGTC-AAGGGCACATG-CCCAGGTT
NW005875787.1_1 : GGCAACGATTTAAAAATAAAAACACTGGCCCCGGCTGGTTTGGCTGTGTGGAT--AGAGTGTCAGCCTGCGGACAGAAGGGTCCCGGATTTGA-TTCTGGTC-AAGGGCACATA-CCTGGGTT
NW005872538.1_1 : TTTTTTTAATAAAAACAAGGCTACATCGCCCTGGCCGGTTTGGCTCAGTGGAT--AGAGCATCAGCCTGTGGACCGAAGGGCCCCAGGTTCGA-TTCTGGTC-AAGGGCACATG-CCTGGATT
NW005876019.1_8 : ATGTTATTGAAGAAGACACAAAAAAAGGCCCTAGCCAATTTGGCTCAGTGG-T--AGAGCATTGGACTGCAGATGGAAGGGGCCTGAGTCCAA-TTCTGGTC-AAGGGCACATG-CCTGGGTT
NW005871054.1_8 : CTTTTTGCATTAAAAAATATGGCATTAGCCCTAGCTGGTTTGGCTCAGTGGAGA-AGAGCGTTGGCCTGCGGACTGAAAGGTCCTGGGTTCGA-TTCTGGTC-AAGGGCACATA--CCGGGTT
NW005871074.1_1 : ATCTGTATGGATAAGAATTGAGATAGTGCCCTAGCTGGTTTGGCTCAGTGGAT--AAAGCATCAGCCTGAGGACTGAAGGGTCCTGGGTTCGA-TTCTGGTC-AAGGGCACA-----------
NW005871068.1_3 : CTAGTGAAATAAGATATTGTAGGTCCAGCCCTAGCTGGTTTGGCTCAGTGGAT--AGAGCATTAGCCTATGGACCGAAGGGTCCCA-------------GTC-AAGGGTACCTG-CCTGGGTT
NW005871151.1_6 : ACTGTACCCTTAAATGTGGTTAAGGTAGCCCTAACTGGTTTGGCTCAGTGGAT--AGAATGTCGGTCTGCGGACTGAAAGGTCCCAAGTTCGA-TTCCGGCC-AAGGAAACATG-CCTGGGTT
NW005872725.1_2 : AGAGTGTGCCTTCAAGAGACTTTATCTGCCCTAGCTGGCTTGGCTCAGTGGAT--AGAGCATCAGCCTGCGGACTCAAGGGTCCCAGGTTTGA-TTCTAGCCTAAGGGCACATG-CCTGGGTT
NW005871311.1_1 : TACAAAGGAATATCAAGAATATCTTCAGCTCTAGCTGGTTTGCCTCAGTGGATAGAGAGCATCAGCCTGCAGACTGAAGGGTCCTGGGTTTGA-TTTCAGTC-AAGGGCACATG-CCCAGATT
NW005871048.1_6 : AGTTATAATGTAGAAATGTCTCCCAGTGCCCTAGCTGGTTTGGCTCACTGGAT--AGAGCGTCGGCCTG-----------------GGTTCGA-TTCCGGTC-AAGGGCATGTA-CCTTGGTT
```

```
NW005871163.1_1 : TTTCTGTTTAAGAAAACTATGATTCAGGCCCGAAGTGTTGTGGCTCAGTGGTT---AAGCACAGACCCACGAACTAAGAGTTCACCAGTTCGA-TTTCCAGT-CAGAGTACATG-CCAGGGTT
NW005871179.1_3 : --GCTATAAATGGCTACCACTGCTCTGTCCTTTGGTTAAAAACCTCCAAGAAT--AGAGCATTGGCCTATGGACTGAAGGCCCTGGGTTTGA-TTCCAGTC-ACAAG--------------
NW005871172.1_1 : TCTGCACAGCAAAAGAAACCATCAACAGCCCTGGCTGGTTTTGCTTGGTGGTT--AGAGCATTGGCCCTCGGACCATAGGATCTGGGTTCGAT-TTCCGGTC--------------CTGGGTT
NW005871095.1_2 : TCCTCTTTTAAAAATATTTTTATTTTTGCCCTGGCTGGTGTTGCTCAGTAGTT--AGAG--CTAGCCCGTACAGGGAAGGGTCATGGGTTCAATTCCTGGTA-AAGGACACGTG-CCTGACTT
NW005871196.1_3 : CAAAGTACTGATTTAAAAAGGCAACCAGCACTGGCCAGTGTAGCTCAGTAGTC--AGAATGTTAGCCCAC--ACCAAAGGGTTGAGGGTTTGATTCCAGGTC-AAGGGCACTTA-GCTGGGGT
NW005871133.1_1 : GGGCCTCTAGTTTTAAAATAATTCCAAGCTCTGGTCAGTGTGGCTAAGTGGTT--AAAGCATCAGCCCACGCACTGAAGGGTCTCCAGTTTGATTCCTGGTC-AAGGGCATGTA-CCTGGGTT

                          *       140         *       160         *       180         *       200         *       220         *       240
Ves_a             : GC-GGGCAC-AT--CCCCAGT---------AGGGGGTGTGCAGGAGGCAGCTGATC-GATG---TTTCTCTCTCATTG-------ATGTTTCTAACTCTCTATC--CCTCTCCCTTCCTCTCT
NW005871638.1_2 : GC-GGGCAC-AT--CCCCAGT---------GGGGGGTGTGCAGGAGGCAGCTGATC-GATG---TTTCTCTCTCATTG-------ATGTTTCTAACTCTCTATC--CCTCTCCCTTCCTCTCT
NW005871332.1_6 : GC-GGGCAC-AT--CCCCAGT---------AGGGGGTGTGCAGGAGGCAGCTGATC-GATG---TTTCTCTCTCATCG-------ATGTTTCTAACTCTCTATC--CCTCTCCCTTCCTCTCT
NW005871213.1_2 : GC-GGGCAC-AA--CCCCAGT---------GGGAGGTGTGCAGGAGGCAGCTGGTC-GATG---TTTCTCTCTCATCG-------ATGTTTCTGACTCTCTATC--TCTCTCCCTTCCTCTCT
NW005871780.1_1 : GC-GGGCAC-AT--CCCCAGT---------AAGGAGTGTGCAGGAGGCAGCTGATT-GATG---TTTCTCTCTCATTG-------ATGTTTCTAACTCTCTATC--CCTCTCCCTTCCTCTCT
NW005871324.1_2 : GT-GGGCAC-AT--CCCCAGT---------AGGGAGTGTGCAGGAGGCAGCTGATC-GATG---TTTCTCTCTCATCG-------ATGTTTCTAACTCTCTATC--CCTCTCCCTTCCTCTCT
NW005871062.1_1 : GC-GGGCAC-AT--CCCCAGT---------GGGGGGTGTGCAGGAGGCAGCTGATC-GATG---TTTCTCTCTCATCG-------ATGTTTCTCACTCTCTATC--CCTCTCCCTTCCTCTCT
NW005871108.1_5 : GC-GGGCAC-AT--CCCCAGT---------GGGGAGTGTGCAGGAGGCAGCTGATC-GGTG---TTTCTCTCTCATTG-------ATGTTTCTAACTCTCTATC--CCTCTCCCTTCCACTCT
NW005871153.1_9 : AC-GGGCAC-AT--CCCCAGG---------AGGGGGTGTGCAGGAGGCAGCTGATT-GATG---TTTCTCTCTCATTG-------ATGTTTCAAACTCTCTATC--CCTCTCCCTTCCTCTCT
NW005871076.1_7 : GC-GGGCAC-AT--CCCCAGT---------AGAAGATGTGCAGGAGGCAGCTGATC-GATG---TTTCTCTCTCATCG-------ATGTTTCTAACTCTCTATC--TCTCTCCCTTCCTCTCT
NW005871066.1_7 : GC-GGGCAC-AC--CCCCACT---------GGGGGGCGTGCAGGAGGCAGCTGATC-GATG---TTTCTCTCTCATCG-------ATGTTTCTAACTCTCTATC--CCTCTCCCTTCTTCTCT
NW005871054.1_8 : GC-GGGCAC-AT--CCCCAGT---------AGGGAGTGTGCAGGAGGCAGCTGATA-GATG---TTTCTCTCTCATCG-------ATGTTTCAAACTCTCTATC--CCTCTCCCTTCCTCTCT
NW005871136.1_4 : GC-GGGCAC-AT--CCCCAGT---------AGGAGGTGTGCAGGAGGCAGCTAATC-GATG---TTTCTCTCTCATTG-------ATGTTTCTAGCTCTCTATC--TCTCTCCCTTCCTCTCT
NW005872463.1_7 : GC-AGGCAC-AT--CCCCAGT---------AGGAGATGTGCAGGAGGCAGCTGATC-GATG---TCTCTCTCTCATCG-------ATGTTTCTAACTCTCTATC--TCTCTCCCTTCCTCTCT
NW005871333.1_4 : GC-GGGCAC-AT--CCCCAGT---------GGGGGATGTGCAGGAGGCGGCTGATC-GATG---TTTCTCTCTCATTG-------ATGTTTCTAACTTTCTATC--ACTCTCCCTTCCTCTCT
NW005871106.1_4 : GC-GGGCAC-AT--CCCCAGT---------GGGGGATGTGCAGGAGGCAGCTGATC-AATG---TTTCTCTCTCATCG-------ATGTTTCTAACTCTCTATC--TCTCTCCCTTCCTCTCT
NW005871059.1_1 : GC-GGGCAC-AT--CCCCTGT---------GGGACATGTGCAGGAGGCAGCTGATC-AGTG---TTTCTCTCTCATCG-------ATGTTTCTAACTCTCTATC--CCTCTCCCTTCCTCTCT
NW005871121.1_4 : GC-GGTCAC-AT--CCCCAGT---------AGGAGATGTGCGGGAGGCAGCTGATC-GATG---TTTCTCTCTCATCG-------ATGTTTCTAGCTCTCTATC--TCTCTCCCTTCCTCTCT
NW005871624.1_7 : GC-GGGCGC-AT--CCCCAGT---------GGGGGATGTGCGGGAGGCAGCTGATC-GATG---TTTCTCTCTCATCA-------ATGTTTCTGACTCTCTGTC--TCTCTCCCTTCCTCTCT
NW005871207.1_1 : GC-AGGCAC-AT--CCCCAGT---------AGGAGAAGTGCAGGAGGCAGCTGGTC-GATG---TTTCTCTCTCATCG-------ATGTTTCTGACTCTCTATC--TCTCTCCCTTCCTCTCT
NW005871210.1_1 : GC-GGGCAC-AT--CCCCAGT---------GGGGGATGTGCAGGAGGCAGCTGATC-GATG---TTTCTCTCTCATCA-------ATGTTTCTAACTCTCTATC--TCTCTCCCTTCCTCTCT
NW005871354.1_8 : GC-GGGCAC-AT--CCCCAGT---------AGGAGATGTGCAGGAGGCAGCTGATC-GATG---TTTCTCTCCCATCA-------ATGTTTCTAACTCTCTATC--TCTCTACCTTCCTCTCT
NW005871413.1_9 : GT-GGGCAC-AT--CCCCAGT---------GGGAGATGTGTAGGAGGCAGCTGATC-GATG---TTTCTCTCTCATCG-------ATGTTTCTTACTCTCTGTC--TCTCTCCCTTCCTCTCT
NW005872128.1_3 : GC-GGGCAC-AT--CCCCAGT---------GGGAGATGTGCAGGAGGCAGCTGGTC-GATG---TTTCTCTCTCATCG-------ATGTTTCTAACTCTCTATC--TCTCTCCCTTCCTCTCT
NW005871081.1_6 : GC-GGGCAC-AT--CCCCAGT---------AGGAGATGTGTAGGAGGCAGCTGATC-GATG---TTTCTCTCTCATCG-------ATGTTTCTAACTCTCTATC--TCTCTCACTTCCTCTCT
NW005871180.1_4 : GC-GGGCAC-AT--ACCCAGT---------AGGGAGTGTACAGGAGGCAGCTGATT-GATG---TTTCTCTCTCATCG-------ATGTTTCTAACTCTCTATC--CCTCTCCCTTCCTCTCT
NW005872499.1_5 : GT-GGGCAC-AT--CCCCAGT---------AGGAGGTGTGCAGGAGGCAGCTGATC-GATG---TTTCCCTGTCATCG-------ATGTTTCTAACTCTCTATC--CCTCTCCCTCCCTCTCT
NW005871056.1_9 : TC-GGGCAC-AT--CCCCAGT---------GGGGAGTGTGCAGGAGGCAGCTGATC-GATG---TTTTTCTCTCATCG-------ATGTTTCTAACTCTCTATC--CCTCTCCCTTCCTCTCT
NW005871108.1_3 : GC-GGGCAC-AT--CCCCAGT---------GGGGAGTGTGCAGGAGGCAGCTGATC-GATG---TTTCTCTCTCATCG-------ATGTTTCTGACTCTCTATC--CCTCTTCCTTCCTCTCT
NW005871462.1_6 : GC-GGGCAC-AT--CCTCAGT---------AGGGGGTGTGCAGGAGGCAGCTGATC-AATG---TTTCTCTCTCATCC-------ATGTTTCTAACTCTCTATC--CCTCTCCCTTCCTCTCT
NW005871275.1_1 : GC-GGGCAC-AT--CCCCAGT---------AGGGAGTGTGCAGGAGGCAGCTGATC-AATG---TTTCTCACTCATCG-------ATGTTTCTGACTCTCTATC--CCTCTCCCTTCCTCTCT
NW005871068.1_9 : GC-AGGCAC-AT--CCCCAGT---------AGGAGGTGTGCAGGAGGCAGCGGATC-GATG---TTTCTCTCTCATCA-------ATGTTTCTAACTCTCTATC--CCTCTCCCTTCCCCTCT
NW005871068.1_7 : GC-GGGCAC-AT--CCCCAGT---------AGGGGGTGTGCAGGAGGTGGCTGATC-GATG---TTTCTCTCTCATCA-------ATGTTTCTAACTCTCTATC--TCTCTCCCTTCTTCTCT
NW005871137.1_2 : GC-GGGCAC-AT--CCCCAGT---------GGGAGGTGTGCAGGAGGCAGCTGATC-GATG---TTTCTCTCTCATCA-------ATGTTTCTAACTCTCTATC--TCTCTCCCTTCCTCTCT
NW005871641.1_3 : GT-GGGCAC-AT--CCCCAGT---------GGGAGATGTGCAGGAGGCAGCTGATC-GATG---TTTCTCTCTCATCG-------ATGTTTCTAACTCTCTATC--TCTCTCCCTTCCTCTCT
NW005871055.1_1 : GC-GGGCAC-AT--CCCCAGT---------AGGGGGTGTGCAGGAGGCAGCTGATC-GATG---CTTCTCTCTCATCG-------ATGTTTCTAACTCTCTATC--CCTCTCCCTTCCTCTCT
NW005871095.1_5 : GC-GGGCAC-AT--CCCCAGT---------GGGGGGTGTGCAGGAGGCAGCTGATC-GATG---TTTCTCTCTCATCG-------ATGTTTCTAGCTCTCTGTC--CCTCTCCCTTCCTCTCT
NW005871236.1_1 : GC-GGGTAC-AT--CCCCAGT---------AGGGGGTGTGCAGGAGGCAGCTGATT-GATG---TTTCTCTCTCATCG-------ATGTTTCTAACTCTCTATC--CCTCTCCCTTCCTCTCT
NW005871062.1_5 : GC-GGGCAC-AT--CCCCAGT---------GGGGAGTGTGCAGGAGGCAGCTGATC-AATG---TTTCTCTCTCATCG-------ATGTTTCTAACTTTCTATC--CCTCTCCCTTCCTCTCT
NW005871049.1_6 : GC-AGGCAC-AT--CCCCAGT---------AGGAGGTGTGCAGGAGGCAGCTGGTC-GATG---CTTCTCTCTCATCA-------ATGTTTCTAGCTCTCTATC--CCTCCCCCTTCCTCTCG
NW005871051.1_3 : GC-AGGCAC-AT--CCCCAGT---------AGGGGGTGTGCAGGAGGCAGCTGATC-AATG---TTTCTCTCTCACTG-------ATGTTTCTAACTCTCTATC--CCTCTCCCTTCCTCTCT
NW005871065.1_1 : GC-AGGCAC-AT--CCCCAGT---------AGGAGGTGTGCAGGAGGCAGCTGATC-GATG---TTTCTCTCTCATCG-------ATGTTTCTAACTTTCTATC--CCTCTCCCTTCCTCTCT
NW005871058.1_9 : GT-GGCCAC-AT--CCCGAGT---------AGGAGGTGTGCAGGAGGCAGCTGATT-GATG---TTTCTCTCCCATCA-------ATGTTTCTAACTCTCTATA--CCTCTCCCTTCCTCTCT
NW005871064.1_1 : GT-GGGCAC-AT--CCCCAGT---------AGGGGGTATGCAGGAGGCAGCTAATT-GATG---TTTCTCTCTCATCA-------ATATTTCTAACTCTCTATC--CCTCTCCCTTCCTCTCT
NW005871050.1_2 : GC-GGGCAC-AT--CCCTAGT---------AGGAGATGTGCAGGAGGCAGCTGATC-GATG---TTTCTCTCTCATCA-------ATGTTTCTAA----CTATC--TCTCTCCCTTCCTCTCT
NW005871077.1_8 : GC-GGGCAC-AT--CCCCAGT---------AGGAGGTGTGCAGGAGGCAGCTGATT-GATG---TTTCTCTCTCATCG-------ATGTTTCTAA----CTATC--TCTCTCCCTTCCTCTCT
NW005871117.1_2 : GT-GGGCAC-AT--CCCCAGT---------GGGGGATGTGCAGGAGGCGGCTGATC-GATG---TTTCTCTCTCATCG-------ATGTTTCTAATTCTCTATC--CCTCTCCCTTCCTCTCT
NW005871344.1_1 : GT-GGGCAC-AT--CCCCAGT---------AGGGGGTGTACAGGAGGCAGCTGATC-GATG---TGTCTCTCTCATCG-------ATGTTTCTAACTCTCTATC--CCTCTCCCTTCCTCTCT
NW005871473.1_6 : GT-GGGCAC-AT--TCCCAGT---------AGGGGGTGTGCAGAAGACAGCTGATT-GATG---TTTCTCTCTCATCA-------GTGTTTCTAACTCTCTATC--CACCTCCCTTCCTCTCT
```

```
NW005871982.1_2 : GT-GGGCAC-AT--CCCCAGT---------AGGGAGCCTGCAGGAGGCAGCTGATC-AATG---TTTCTCTCTCATCA-------ATGTTTCTGACTTTCTATC--CCTCTCCTTTCCTCTCT
NW005875779.1_4 : GC-GGGCAC-AT--CCCCAGA---------GGGAGATGTGCAGGAGGCAGCTGGTC-GATG---TTTCTCTCTCATCG-------ATGTTTCTGGCTCTCCATC--CCTCTCTCTTCCTCTCT
NW005871833.1_1 : GT-GGGCAC-AT--CCCCAGT---------AGGGGGCGTGAAGGAGGCAGCTGATC-GATG---TTTCTCTCTCGTTG-------ATGTTTCTAACTCTCTACC--CCTCTCCCTTCTTCTCT
NW005871115.1_1 : GC-GGGCAC-AT--CCCCAGT---------TGGGGGTGTGCAGGAGGCAGCTGATC-AATG---TTTCTCTCTCATCG-------ATGTTTCTAACTCTCTATC--CCTCTCCCTTCCTCTCT
NW005871152.1_1 : GC-GGGCAC-AT--CCCCAGT---------CGGAGGTGTGCAGGAGGCAGCTGATC-TATG---TTTCTCGCTCATCG-------ATGTTTCTAACTCTCTATC--CCTCTCCCTTCCTCTCT
NW005871048.1_5 : GC-AGGCAC-AT--CCCCAGT---------GAGGGGTGTGCAGGAGGCAGCTGATC-AATG---CTTCTCTCTCATTG-------ATATTTCTAACTCTCTATC--CCTCTCCCTTCCTCTCT
NW005871311.1_1 : GC-GGGCAC-AT--CCCCAGT---------GGGGGGCGTGCAGGAGGCAGCTGATTGGATG---TTTCTCGCTCATCG-------ATGTTTCTAACTCTCTATC--CCTCTCCCTTCCTCTCT
NW005871051.1_2 : GC-AGGCAC-AT--CCCCAGT---------AGGGAGTGTGCAGGAGGCAGCTGATC-GATA---TTTCTCTCTCATTG-------ATGTTTCTAACTCTCTAT---CCTCTCCCTTCCTCTCT
NW005871155.1_2 : GC-GGGCAC-AT--CCCCAGT---------GGGAGGTGTGCAGGAGGCAGCTGATC-AATG---TTTCTCTCTCATCG-------ATGTTTCTAATTCTCTATC--CCTCTCCCTTCCTCTCT
NW005872153.1_3 : GC-GGGCAC-AT--CCCCAGT---------GGGGGGCGTGCAGGAGGCAGCTGATC-GA-------TCTCTCTCATCA-------ATGTTTCTAACTCTCTATC--CCTCTCCCTTCTTCTCT
NW005871148.1_4 : GC-AGGCACAAT--CCCCAGT---------AGGAGATGTGCAGGAGGCAGCTGATT-GATG---TTTCTCTCTCATCA-------ATGTTTCTAACTCTCTATC--CCTCTCCCTTCCTCTCT
NW005871082.1_2 : GT-GGGCAC-AT--CCCCAGT---------AGGAGGTGTGTAGGAGGCAGCTGATC-GATG---TTTCTCTCTCATTG-------ATGTTTCTAACTCTCTATC--CCTCTACCTTCCTCTCT
NW005871337.1_1 : GT-GGGCAC-ATTCCCCCAGT---------AAGGGGTGTGCAGGAGGCAGCTGATC-GATG---TTTCTCTCTCATTG-------ATGTTTCTA--CCTCTATC--CCTCTCCCTTCCTCTCT
NW005871065.1_1 : GC-GGGCAC-AT--CCCCAGT---------GGGGGCCGTGCAGGAGGCAGCTGATC-GA-------TCTCACTCATCG-------ATGTTTCTAGCTCTCTATC--CCTCTCCCTTCTTCTCT
NW005871165.1_3 : GC-GGGCAC-AT--CCCCACT---------GGGGGGCGTGCAGGAGGCAGTTGATC-GA-------TCTCTCTCATCG-------ATGTTTCTAACTCTCTATC--CCTCTCCCTTCTTCTCT
NW005871073.1_3 : GA-GGACTC------------------------GGACCATGCAGGAGACAGCCAATC-AGTG---AGTCTCTCTTATTAT----TGATGTTTCTATCTGTCTCTC--CCTCTTCCTTCCTCTCT
NW005871563.1_1 : GC-GGGCTCTAT--CCCCAGT---------AGGGGTCATGCAGGAGGCAGCCAATC-AATG---ATTCTC-ATCAC-------TGATGTTTCCATCTCTCTCTC--CCACTACCTTCCTCTCT
NW005871431.1_9 : GC-GGTCAC-AT--CCCCAGC---------TGGGGATGTGCAGGAGGCGGCTGATC-AATG---TTTCTCTCTCATCA-------ATGATTCTAACTCTCTATC--CCTTTCCCTTCCTCTCT
NW005871069.1_9 : GC-AGGCAC-AT--CCCCAGT---------AGGGGGTAGGCAGGAGGCAGCTAATT-GATG---TTTCTCTCTCATCA-------ATGTTTCTGACTCTCTATT--CCTCTCCCTTCCTCTCT
NW005871112.1_3 : GT-GGGCAC-AT--CCCC------------------------GGAGGCAGCTGATC-GATG---TTTCTCTCTCATCG-------ATGTTTCTGACTGTCTATC--CCTCTCCCTTCCTCTCT
NW005871096.1_5 : GC-GGGCAC-AT--CCCCAGT---------AGGGAGTGTGCAGGAGGGAGCTGATC-AATG---TTTCTCTCTCATCG-------ATGTTTCTAACTCTCTATC--CCTCACCCTTCTTCTCT
NW005871270.1_3 : GT-GGGCAC-AT--ACCCAGT---------GGGGAGTGTGCAGGAGGCAGCTGGTC----------------------------GATGTTTCTGGCTCTCTATC--CCTCT-CCTTCCTCTCT
NW005871767.1_1 : GT-GGGCAC-AT--CCCCAGT---------GGGAAGTGTGCAGGAGGCAGCTGATC-A---------------------------ATGTTTCTAACTCTCTATC--CCTCTCCCTTCTTCTCT
NW005871176.1_3 : GC-GGGCAC-AT--CCCCAGT---------GGGAGGTGTGCAGGAGGCAGCTGATC-GATG---------------------------TTTCTAA----CTATC--TCTCTCCCTTCCTCTCT
NW005871060.1_1 : GC-GGGCTCGGT--CCCCAGT---------GGGGGGTGTGCAGGAGGCAGCTGATT-GATG---TTTTTCTCTCATCA-------ATGTTTCTAA----CTATC--CCTCTCCCTTCCTCTCT
NW005871091.1_4 : GC-GGGCTCGAT--CCCCAGT---------AGGAGGTGTGCA-AAGGCAGCCAACC-AATG---ATTC----TCATCAT----TGATGTTTCTACCTCTCTCTC--CCTCTCCCTTCCTCTCT
NW005871534.1_3 : GT-GGGCTCCAT--CCCCAGT---------GGGGGCCGTGCAGGAGGCAGATGATC-AATG---ATTC----TCATCAT----TGATGTTTTTATCTCTCTGTC--TCTCTCCCTTCCTCTCT
NW005871346.1_5 : GT-GGGCTCAGT--CCCCAGT---------GGGGGGTGTGCAGGAGGCAGCTGATA-AATG---ATTCTC--TCATTGT----TGATGTTTCTGTG----------TCTCTCCCTTCCTCTCT
NW005871075.1_2 : GC-GGGCTCGAT--TCCAAGT---------AGGGGGCATGCAGGAGGCAGCCAATC-AATG---ATTCTCTCTTATCAT----TGATGTTTCTATCTCTCTCTC--CCTCTCCCTTCCTCTCT
NW005871048.1_1 : CC-AGGCTCGAT--CTCTAGT---------AGGAGGTGTGCAGGAGGCAGCCACTC-AATG---ATTCTCTCTCATCAC----TGATGTTTCTATCTCTTTCTC--CCTCTCCCTTCCTCTCT
NW005871088.1_8 : TC-AGGCTCAAT--CCCCTGT---------AGGGAGCATGCAGGAGGCAGCCAATC-AATG---ATTCTCTCTCATCAT----TGATGTTTCTATCTCTCTCTC--CCTTTCCCTTCCTCTCT
NW005871126.1_9 : GC-AGGTTCGAT--CCCTAGT---------AGGGAGTATGTAGGAGGCAGCCAATC-AATG---ATTC--TCTCATCAT----TGATGTTTCTCTCTCTCTCTC--CCTCTCCCTTCCTCTCT
NW005871419.1_8 : GC-AGGCTCAAT--CCCCAGT---------AGTGAGCATGCAGGAGGTAGCCAATT-ACTG---ATTCTTTCTCAACAT----TGATGTTTCTATCTCTATTTC--CCTTTCCTTTCCTCTCT
NW005871067.1_7 : GT-GGGCTCGAT--CCCCAGT--------GGGGGGGCTTGCAGAAGGCAGCTGATC-AATG---ATTCTTTCTCATCAT----TAATGTTTCTA--TCTCTCTC--ACTCTCCCTTCCTCTCT
NW005871349.1_1 : TC-AGGCTTGAT--CACCAGT---------AGGGGGCATGCGGGAGGCAGCCAATC-AGTG---ATTCTCTC-CATCAT----TGATGTTTCTATCTCTCTCTC--CCTCTCCCTTCCTCTCT
NW005871109.1_5 : TC-GGGCTAGAT--CCCCAGT---------AGGGGGTGTGCAGGAGGCAGCTGATC-AATG---ATTTTCTCTCATCAT----TGATGTTTCTATCTCTATCTC--CCTGTCCCTTCCTCTCG
NW005874004.1_1 : GC-AGGTTCAAT--CCCCAGT---------GGAGGGTATGCAGGAGGCAGCCGATC-AATG---ATTCTCTCTCATCAT----TGATGTTTCTATCCCTCTCTCAACCTATCCCTTCCTCTCT
NW005871206.1_6 : GC-GGGCTCGAT--CCCCAGT---------AAGGGGCGTGCTGGAGGCAGCCGATC-AATG---ATTCTCTCTCATCAT----TGATGTTTCTATCTCTC------CTTCTCCCTTCCTCTAT
NW005871123.1_4 : GT-GGGCTTGAT--TCCCAGC---------AGGGCACATGCAGGAGGCAGCTGATC-AGTG---ATTCTCTCTCATCAT----TGATGATTC----TCTCTCTC--CTTCTCCCTTCCTCTCT
NW005871055.1_6 : GT--GGCTCGAT--CCCCAGT---------AGAGGGCGTGCAGGAGGCAGCCAATC-AATG---ATTCTCTCTTATCAT----TGATGTTTCTATCTCTCTCTC--TTTGTCCCTTCCTCTCT
NW005871068.1_9 : GT-GGTCCTGAT--CCCCAGT---------AGGGGGCGTGCAGGTTGCAGCCAATC-AATGAATATTCTTTCTCATCAT----TGATATTTCTATCTCTCTCTC--CCTCTCTCTTCCTGTCT
NW005871516.1_4 : GC-AGGCTCCAT--TCTCAGTA--------GGGGGGGCATGCAGGAGGCAACCGATC-CATG---ATTCTCTCTCATTAT----TGATGTTTCTATCTC--------TCTCTCCCTTCTTCTCT
NW005871085.1_3 : GT-GGGCTTGGT--CCCCACT---------AGGGGGCGTGCAGGAGGCCGCCAATA-AATG---ATTCTCTCTCATCAT----TAATGTTTCTATCTCTC----------------CCTCTCT
NW005871063.1_5 : GTGGGGGCTTGT--TTCCAGT---------GGGGGACATGCAGGAGGCAGCCAATC-AATG---ATTCTCTCTCATCAT----TGATGTTTCTATCTCTC------CCTCTCCCTTCTGCTCT
NW005871093.1_5 : GT-----------------------------GGGGGCATGCAGGAGGCAGCCAATC-AATG---ATTCTCTCTCATCAT----TGATGTTTCTATCTCTC------CCTCTCCCTTCCTCTTT
NW005881170.1_2 : GC-GGACTCCAT--CCCCCGT---------AGGGGGCGTGCCGGAGGCAGCCAATC-CATG---ATTTTCTCTCATCAT----GGATGTTTCTA--TCTCTCTC--CCTCCCCCTTCCTCTCT
NW005871097.1_6 : GC-GGGCTCGAT--CCCCAGTG----GGGGGGCGGGTGTGCAGGAGGCAGCTGATC-AATG---ATTCTCTCTCATCAG----TGATGTTTC----TCTCTCTC--CCTCTCCATGCCTCTCT
NW005871464.1_5 : AT-GGGCTTGAT--CCCCAGT---------GGGGGGCTTGCAAGAGGCAGCCAATC-AATG--- TTCTCTCTCTTCTT----TGATGTTTCTATCTCTCTCTC--CCTCTTCCTTCCTCTCT
NW005871049.1_2 : GT-GGGCTCAAT--CCCCAAT---------AGGGGGCATGCAGGAGGCAGTCAATC-AATG---ATTCTCTCTGATCAT-----TGTGTTTCTATCTCTATCTC--CCTTTTCTTTCCTCTCT
NW005871111.1_3 : GC-AGGCTCGAT--CCCCAGT---------AGGGGGCATGCAGGAGGCAGCCGATC-AATC---AT-----------------TGATGTTTCTCTCTCTCTCTCCCCCTCTCCCTTCCTCTCT
NW005871143.1_1 : GC-GGGCTCGAT--CCCTAGT---------GGGGGCCGTGCAGGAGGCAGCTGATC-AATG------------------------ATGTTTCTA--TCTCTCTC--CCTCACCCTTCCTCTCT
NW005872372.1_5 : GC-TGGCTCCAT--CCCCAGT---------GGGGGGCGTGTAGGTGGCAGTGGATC-AATG---ATTCTCTCTTATCAT----TGATGTTTCTATCTTTCTCTC--CCTCTCCCTTCCTCTCT
NW005871909.1_5 : GT-GGGCTCGAT--CCCCAGT---------GGGGGCTGTGCAGGAGACAGCCGATC--ATG---ATTTTCTCTCATCAT----TGATGTTTCTATCTC--------TTTCTCCCTTTCTCTCT
NW005875787.1_1 : GT-GGGCTCAAT--CCCCAGT---------ATGGGGCATGCAGGAGGCAGCTGATC-GATG---ATTCTC--TCATCAT----TGATGTTTCTATCTCTCTCTC--CCATTCCCTTCTATATA
NW005872538.1_1 : GC-GGGCTCGGT--CC------------------AACGTGCAGGAGGCAGCCGATC-AATG---ATTCCTTCTCATCAT----GGATGTTTCTA----------TCTCTCTCTTCCTCTCT
NW005876019.1_8 : GC-GGGCTTGAT--CCCCAGT---------AGGGGGAGTGCAAGAGGCAGCCAATT-GATG---ATTCTCTCTCATCATTCATTGATGTTTCTA--TGTCTCTC--CCTCTCCCTTCCTCTCT
NW005871054.1_8 : GT-GGGCTTGAT--CCCCAAT---------AGGGGGTGTTCAGGAGGCACCCGATC-AATG---ATTTTCTCTCATCAT----TGATGTTTCTCTCTT--------CCTCTCCCTTCCTCTCT
```

```
NW005871074.1_1 : ------------------CCAGT----------AGGGCGTGTGCAGGAGGCAGCCGATC-AATG---ATTCTCTCTCATCAT----TGATGTTTCTATCTC-CTCCC--CCTCTCCCTTCCTCTCT
NW005871068.1_3 : TT-GGGCTAGAT--CCCCAGT----------GGGGGGTGTGCAGGAGGCAGCCGATC-AATG---TTGCTCTCTCATCAT----CGATATTTCTGTCTCTCTCTC--CCTCTCCCTTCCTCTCT
NW005871151.1_6 : TT-GGGCTTCAT--CCCCACT----------GTGGGGT-----------CAGCCGATC--AGG---ATTTTCTCTCATCAT----TGATGTTTCTATCTCTCTCT---CCTCTCCCTTCCTCTCT
NW005872725.1_2 : GT--GGCTTGAT--CCCCAGTGGGGGGGGGGGGGGCAGGCAGGAGGCAGCCAATC-AATG---ATTCTCTCTCATCAT----TGATGTTTCTATC-----------TCTCCCTCTTCCTCTCT
NW005871311.1_1 : GC--GACTCAAT--TTCCAGT----------AGGGGGTGTACAGAAGGTAGCCTATC-AATG---ATTC-CTCTCATCAC----TGATGTTTCTATCTCTCTTTC--CCTCTTTCTTCCTCTCT
NW005871048.1_6 : GC-AGGCAC-AT--CCCCAGT----------AGTGGGTGTGTAGGAAGCAGCTGATC-AATG---CTTCTCTCTCATCG--------ATGTTTCTAACTCTCTATC--CCTCTCCCTTCCTCGCT
NW005871163.1_1 : GC-GGGCTCGAT--CCCCAGT----------AGGGGGTGTGCAGGAGGCAGCTAATC-GATGATGTTTCTCTCTCATCA--------ATGTTTCTATCTTTCTATC--CCTCTCTCTTCCTCTCT
NW005871179.1_3 : ----GGCTCAAT--CCCCATT----------AGGGGGTGTGCTGGAGGCAGCAAATC-AATG---ATTCTCTCTCATCA-------------TTTCTCTCTCTC--TCTCCCTCTTCCTCTCT
NW005871172.1_1 : GC-AGACCTGAT---CCCAGC--CTGGGTCAAGGCATATGCAGGAGGCAACCGATC-GATG-----TGTCTCACATCG--------ATGTCTCTCTCTCTCTC--TCTCTCCCCCTCTCTTT
NW005871095.1_2 : GC-AGTTTCCAT--CCCCAGC--CTCCACTAGGGTGAGTGTGGCAGGCAACCAATT-GATG----TTCTCTCACATTAT--------TGTTTCTCTCTCTCTC--TCTCTCTCTCTCTCTCT
NW005871196.1_3 : GC-AGGTTCAAT--CCCCAGA--TTTGGTTGGGGCATGTATGGAGGCAACCAATC-GATG---TGTCTCTCTCA-CAT----CAATATTTCTCTCTCTCTCTC--TCTCTCTCTCTCTCTCT
NW005871133.1_1 : GC-AG----------------------CAGAGGAAGAGTGTGGAGGCAACCAATC-TATG---TGTCTCTCTCA-CAT----TGATGTTTCTCTCTCTCTCTC--TCTCTTTCTCTCTATCT

                     *       260         *       280         *       300         *       320         *       340         *       360
Ves_a           : GT--AAAAAATCAATAAAAATATATTTTAAAAAAAAAA----------------------------------------------------------------------------------------
NW005871638.1_2 : GT-AAAAAAATCAATAAAA-TATATTTAAAAAAAAAAAAAAAAAAGAGAAAGTCACTCTGATCCATGTGAAAAATCAGAGTCTTGTACCTCTCCCAGGGAGAGGTCTGGAGAGAGAGCAAGAA
NW005871332.1_6 : GT--AAAAAATCAATAAAA-TATATTTTTAAAAAAACAAACAATACTATTAGCTACACGACGGACTTTAAGTCATGCTCACCGTGTCTCCACCAGCATCTCTCTTCTGCCCTAAGACACAATC
NW005871213.1_2 : GT--AAAAAATCAATAAAAATATATTTTTTAAAAAAATCAACAATATAATTTCTAATAGTGAAAAGTGCAGTGATAGGGCTAAAATCAAAGCCATGGATAACACCAAATGCTGATGAGGAAGT
NW005871780.1_1 : GT--AAAAAATCAATAAAA-TATATATTTTTTAAAAACAAAAACAAAAAAACAACAAAGAGGACAATGGGCCCAGCTGGCGAGGGTGAAGAAGAGGACCTTCACCAAGGAGCTATTATGTGAT
NW005871324.1_2 : GT--AAAAAATCAATAAAA-TATATTAAAAAAAAAAGAGTTCAAGTGTGACAATCAGTTCTAGCACAAAACCAGATGGCTTTTCCTCCATTCGGTTACGTATAATTCAACAAATGTAGGTGTA
NW005871062.1_1 : GT-AAAAAAATCAATAAAA-TATATTTTTTTTAAAAAATGTGAGATGAGGAGTTTATCTTGGGTCATCTTGGCAAGCCCAAGGTATTCACTGGGGTCCTGCTAAGTCAGAGAGGAGGCAGGAG
NW005871108.1_5 : GT--AAAAAATCAATAAAA-TATATATTTAAAATAAAATAAAATAAAATAGATGTCTTATTGTACTTTATCTCTTTATAGATCAAGTGTTGATAGAAGGCAACCAGTGCATTGCTTTCATGGT
NW005871153.1_9 : AT--AAAAAACCAATAAAA-TATATTTAAAAATAAAATAAAATTGCATCATAATTGCTTATTTACTATTTATTTTACTTATTTAGTGTAACACTTTAAAAAATAACATTTGCTTCTGACTTCT
NW005871076.1_7 : GT--AAAAAATCAATAAAA-TATATTTAAAAAAAAAAAAATAAAAATGAGACTTTACTTACTTATTTATTTATTTATTTATTTTCATTTACAGTTTACTTTCAGTATTATTTTGTATTGGTTTC
NW005871066.1_7 : GT--AAAAAATCAATAAAA-TATATTTTAAAAAAAAAGATGTGAGCATGGGATCTCCAAGGGCTCCAAGAGAATGGAGAAAAATCACTCACTTTGTTGTTGTTCTCACACCTCAACTCCATGG
NW005871054.1_8 : GT--AAAAAATCAATAAAA-TACTTAAAAAAATAAATAAATAAAATAAAATATCAATCAATTCACAGAATTTTCACCCAGCTCACCTCCATACTTATACCAACACATCAGCATGCATTCAGTA
NW005871136.1_4 : GT--AAAAAATCAATAAAA-TATATTTAAAAAAAAAAGAATCCTAAACAAAAATCCAGGGAAAGGAGAAATTATCCTAGTTGAAAATATGGAAATACATGAAGAGTACTAGAAAGGGTAGTGG
NW005872463.1_7 : GT--AAAAAATCAATAAAA-TATATTTTTTAAAAAAAATAACTATGGATACCAATGTGCTGGTACATGTGCCGTAACTTTGGATACTACAGAAGAAAACACAGGAAAAAAATTCATATACAAG
NW005871333.1_4 : GT--AAAAAATCAATAAAA-TATATTTTTAAAAAAATTTTATAAAATAAATATTATAAAAGCATATAATTTTAAAACATAAAAATATGAATGGGGAAATTTTTATCAACCTCAGGGGAGAGGC
NW005871106.1_4 : GT--AAAAAATCAATAAAA-TATATTAAAAAAAAATCTTCCCATTTTATCATATCTTATCATTGTTTATGATGTCTTTTTTTAAATTCCCAAATTCAAATTTTGGGTTTATAGTAAAATGTAT
NW005871059.1_1 : GT--AAAAAATCAATAAAA-TATATTTAAAAAAAAAAACAACAAAAAAACAAAAAGAGACTTCAGAGGAAGTAGATGCTTGTGGGAATTCATTTGTAGAAAACGATGCTAGAGTAACACTCCA
NW005871121.1_4 : GT--AAAAAATCATTAAAA-TATATTTAAAAAAAAAAAGAAAACCTTTGAGTTTTAGGTAAAGATTAAGAGGGCATAGTTTTAAAGCACTCATTTAAAATTAGGGATAAGCAAATTCACGCTT
NW005871624.1_7 : GT--AAAAAATCAATAAAA-TATATTTAAAAAAAAAAAAAAAAAAGAGGGTACACCTGGAGTGACGCAGAACTCCTGTTACATAGTTGGGCTCATCTCTTCACCTCTTCATACTTCAGGTTCC
NW005871207.1_1 : GT--AAAAAATCAATAAAA-TATATTTAAAAAAAAAAAGAAACTTGCAGATGCATACAAACATAATCTGTCTTTCAGCTAATACCTGCATTGCTTGATGCATCAAGTTATGGCTGGACTATA
NW005871210.1_1 : GT--AAAAAATCAATAAAA-TATATTAAAAAAATATTTCTTCTAATTAATTTCCTTTCAATGTGCACAAATTCGTGCACCAGGCCACTAGTACTGTAATGACAATGTATGGTGTCACATGGGT
NW005871354.1_8 : GT--AAAAAATCAATAAAA-AATATATTAAAAAATAAATAAAATGAAGTAATAATGTGAGGTTTAAGTGGCTCTAATTGCTTTTTCAACTACTATTTTTACTTTCTTCGTGATTACCTTGAGA
NW005871413.1_9 : GT--AAAGAATCAATAAAA-TATATTTTAAAAAAAAAGTAAGTCCTTCATTTTCCAAAGCAAACCCAAACTAACAAGAGGGACATTTCCAGATGTGAAGTGATGGTCTTTGTACAACTTTCAT
NW005872128.1_3 : GT--AAAAAATCAATAAAA-TATATTAAAAAAAATTATATACAGAGATCCAACAGCAGACTAGAGGATCCCAAGAATCAAGTCAAAGATTTGAAACACGAAGAAGCAAAAAACATCCATCTGG
NW005871081.1_6 : GT--AAAAAATCAATAAAA-TATATTAAAAAATAATAATTTAGGTAACATTACTGACTGTATGAAAATGGTATTTGTTGTTTTTACTATTTTATGTCCCCCACATTAAAAGGCAGATTTAAGG
NW005871180.1_4 : TT--AAAAAATCAATAAAA-TATATTTTAAAAAGGGGGGGAGGGTAGTGGAGTAATCTCCTGTAAGTAAACTACATCAAGCCTTAATTACTTTAAATTTTTTCACTTTAAAATTTTTTCGCTT
NW005872499.1_5 : GT--AAAAAATCAATAAAA-TGCATTAAAAAAAATTCTAGAGTGTAGGTTAACTATTTTGTCCCTGGAAAACAGCCTTTTCCTAATTCCTTCTTGATAAAATATATAACTATTCTTTTATTT
NW005871056.1_9 : GT-AAAAAAATCAATAAAA-TATATTAAAAAAAAAAAACCCACAGTGGATGCCTTTTCAATTCTCTGAAAAGGGAGCTCGCACTTCCTGTTCCTGTGTTGAAATGTTGCCTTTTTCTTACAAT
NW005871108.1_3 : GT--GGAAAATCAATAAAA-TATATTTTAAAAAATGCTATACAAATGGAATCATACAGTATGTAACCTTTCGGGATTGACTTTTTGTGTTGTTGTTAATCCTCACCTGAGGATATTTTTCCCC
NW005871462.1_6 : GT--AAAAAATCAATAAAA-TATTTTTTAAAAATTCAATGATAAAATTTAAAAGTGCTATATTGCATATTTGAAAGTTACTGAGAGAATAGATCTTAAAAGTTCTCATGACAAAGAAATTTTA
NW005871275.1_1 : GT--AAAAAATCAATAAAG-TATATAACAAAATATAATTGATGGATTATTTGCAGGTTTGGATGTAACCTTCATTAGCATAAGAGTGACTTAGAGTTGACTGAAGATTTATTTTTAGTTTAGA
NW005871068.1_9 : GT--AAAAAATCAATAGAA-TATATTTTTTAAAAAATGTGTCCTTAAAATTGTGCATCTTCTTCATTGTTAATTATACAAAAACAATAATATGTTTATTGTCTATAACAATATTGACTTTTAC
NW005871068.1_7 : GT--AAAAAATAAATAAAA-TATATTTTAAAAATGTATCAAACATGAAAAAAGAAATGCAAACAGAAACAAAACTAATCTAAATGGGTATACTTATTCATGGAGAATCCATTAGCATTATTGA
NW005871137.1_2 : GT-AAAAAAATCAATAAAA-AATATATTAAAAAAAAAAAAGATTGGAAGACATGCTTTTATTCTGATCCATGAATATTTATCATCATACTGTTTTGGGAACCCCATAAGAATTTTTTCTTCTAA
NW005871641.1_3 : GT----AAAATCAATAAAA-TATATTAAAAAAAAATAAACACTACTTTTATAATTATTCAAAGTGTGTGTATACATTTTTGGGGGATACACTATATATGTCTCATTGTACCACAGTTTAACG
NW005871055.1_1 : GT-AAAAAAATCAATAAAA-TATATATATTTTAAAAAGAATAAACATGAAATTTAAATGTAGAAGCCTGAGTAGCTCATTTTGTCCAAAAGATTTACAAAGCAACTTTGGCAGCCTTTAATGT
NW005871095.1_5 : GTAAAAAAAATCAATGAAA-TATATTTAAAAAAAAAAAAGAACTAGCCCATGAATTTAACTTTGGAAGCACTGGAGAGTGTTTGCATTCAGAAGCCTCTTCTGCTACATGCATTAAAATGAAC
NW005871236.1_1 : GTAAAAAAAATTAATAAAA-TATATTTTAACAATCCTATCTAATAAAGAGGGAATATGCTAATTGACCCTCACATTGTTGCAACGATGGCAGCACCCACAGCCAATAAGGATGGAATATGCTA
NW005871062.1_5 : GT-AAAAAAATTAATAAAA-TATATTATAAAAAACAACAGCATGGTATTGTCAGATAAACAATACACAGATCATTGGAACAGAATAGATAGCCCAGCTATAAAACCACATGTATATGGATAGA
NW005871049.1_6 : GT-AAAAAAATCAATAAAAATATATTTTAAAAAAAAATTTTAAAATGAGAGACATTTGTCCTGCTGATAACAAATAGATAAAGCTGTGGTGGTAATGCACAGGATAGATCATCTGAGCAGAAT
NW005871051.1_3 : GT--AAAAAATAAGTAAAATAAATTTTTAAAAAAGAGGGAAATGAAGAGTGTAGTAATGAAGCCATGATGTAAGTCCATTGACTGGCAGGACAGTCTGTGAGGTACACATAATTATTCCTT
NW005871065.1_1 : GTAAAAAAAATCAATAAAA-TATATTTTTTTAAAAAAGGTTAGTGGTTCTAGGCCATAGCTGAGCGAAAGACTGACCTCATTCTTAGACTGTTTTGTTTTGAGTTGTCCTAGGTCAGTGCTTC
```

```
NW005871058.1_9 : GT--AAAAAATCAATAAAA-TATATTTTTTTAAAAAAAGAAGTGCAGATTGGTTGTTATAGAATAGTCATGGGGTGTAAAGTACACCATTGGGAATATAGTCAATAATCTTTCTATATAATAA
NW005871064.1_1 : GT--AAAAAATCAATAAAA-CATATTTTTTTAAAAAAAGAATTGCTGGGAGGATTCAAGTGAAACAATGCTGTGTCTGTGCCCCAGGTACCTGAAGTGCCCAGTGTTGCATAGAGCAAACCCT
NW005871050.1_2 : GT--AAAAAATCAATAAAC-TATATTTAAAAAAAGAGAAAAGTATGGCCACTCTTTTTTCAACCAAATAGATTGTACATGTCTTACTGATTCAGGAATTCACATTCTCTACAATGCAGGTGAG
NW005871077.1_8 : GT--AAGAAATCAATAAAA-TATATTTTTTAAAAAAGAAGAGCAATTTAGCCAATTGTGGCATCAAAGGTTGTGAATATTCTGTTTTCAAAAGAATATACTGTTTTCATCAGTATTCACACTA
NW005871117.1_2 : GT--AAAAAATCAATAAAA-AATATATTTTTTTAAAACAAACAAAAAAAAACAGCTCTAACCTACTGGTGCTGAGATCGCTGATCATGGATTTTTCAGGGTTCAGTAACTTGGAACTACTCATA
NW005871344.1_1 : GT---AAAAATCAATAAAA-TATTTTTTTTAAAAAAAGAAGCCTTGGTTTGGGAGCAACACAAACATTTTCTACCTCGTAAGCAATGACCAAGTTCTTGTCTAGGCTAAAAATGCAAAAATTG
NW005871473.1_6 : GT--GAAAAATCGATAAAA-TATATTTTAAAAATAAAATAAAATAAAATAAAATAAAATAAAGGGCTAGTATATTTTACATGGTTGGGGACAGGGTTTTCATGGGCACCGCTAGAATGGAGAC
NW005871982.1_2 : GT--AAAAATTCAATAAAA-TATATTATTAAAAAAAAGTCCCTGAAGTTACTGAGCTTTCATTCTGGTGTGGGAGACAGATAATAAAACAAGTACAAAGGTATTTTCAAACAGCAGAGTACAA
NW005875779.1_4 : GC--GAAAAATCAATAAAA-TATTTTTTTAAAGGCGATTCTGCCGCAGAATTTTAGTGATCAGTTTATGGCTGGACGTGTCTGTTCTGAACAATGATAACATTGGGCTGACATTCATAATGTG
NW005871833.1_1 : GT--AAAAAATCAATAAAA-TATATTTTTAAAAATAAAAGAAAATAGAATTCTACCGAGTCTCCTGTCTACCTACTCCAGGACTTCTGTGAGGATCAAATGGGATGAGGCACAGGAAAATGCT
NW005871115.1_1 : GT--AAAAAATCAATAAAA-TATATTTAAAAACAAAACAAAAAAAAACATTGTTTCTATTCCAGGGTCCCTTCATTTGAATGGATGGATAAAAGCTTCTAGTCATTTGAATTTAGTTTTGATA
NW005871152.1_1 : GT--ACAAAATCAATAAAA-TATTTTTTTTAAAAAAAGATGTTTATTCCAGACCCTAGAATCAGCCTTACCCTTTATTTCACGCGGTAATCAACATGAGGCATCCCCTTTGGAGAAGACGTGG
NW005871048.1_5 : AT-AAAAAAATCAATAAAA-AATATATTAAAACATAAAAATATTAAAATAAATATTTTATTCATTTTGAGTTTATTCTTGTATATGGTATGTTTGTTGTCTAGTTTTATTTTTTGCATGCACC
NW005871311.1_1 : GT--AAAAAATCAATAAAA-TATATTAAAAAAAATGAAATCCGTGTTGGAGGGAGCCTGTCGACCTGGGATAAATTTAGTTTCCGTACAAGTGGCCCCAGCTAGGTGAGGAGACCACCGCAGT
NW005871051.1_2 : GT--AAAAAATCAATAAAA-TATATGTTTTAAAAAAAAGTCAGAAGGATTGACATTTTTAAATTGCTATGTTTTCCTAATTGGAGGTACCTTCTACATTTCCATTTATTTATATCTTGTTTAA
NW005871155.1_2 : GT--AAAACTTCAATAAAA-TACATTAAAAAATTATTTTCGTTGCTACTTCATAACTGTAATGTTGCTACTGTTATGAATCATAATGTAAATATCTGATATGCAGGATGGTTTTAGGCGACCC
NW005872153.1_3 : GT--AAAAAATCAATAAAA-TATATTTTTTTAAAAAATAAGATAAAAATAAAAAGGTCAAATACTGCCTGACCCAGTCTTCAATTGGTGCCTCCCCTGTTCTTCCGCTGCTGCAGGCTCCTCT
NW005871148.1_4 : GT---AAAAATCAATAAAA-TATATTTAAAAAAAAAAAAAGGAATGAAATTCTGATAGATTTCAACATGGATCCACCTTGAAAATGCTAGGCTAAGTGAACTAAGGCAGACACAAAGGAACAA
NW005871082.1_2 : GT--AAAAAATCAATAAAA-TATATTCTTAAATGAAGTAGCCATTGGCATGAGTCTACAGGAGCTGAGCAGGACTGCTGAGGGGAGGACATCATGGACATCACTCCGGCATAGAGTCGCCAGG
NW005871337.1_1 : GT--AAAAAATCAATAAAA-TATATTAATTTTTTTTTAAAAAAGACATGGATAACTTCATAGCCCAATAGTTAATTACTAAGGAAATTGAATAATTAAATATATCCCTACAAATAAAATTCCA
NW005871065.1_1 : GT--AGAAAATCAATAAAA-TATATTAAAAAAATAAAAAATAAAATAGGACGTCTACAATATAAAACCCATGCTTATTTGCATGAAAACATCACCCACAAAGCTAAATAAGCCTTAAAAAGTT
NW005871165.1_3 : GT--AAAAAATCAATAAAA-TATATTTAAAAAAAAAAAGCTGAGCCAGGTCAGGATGCAGTCCTAAGAATTGGGCAGCAATAGATTATTCGTTGTTGTGGCATAACCATGGTTGTGAGAATTT
NW005871073.1_3 : GA------AATCAATAAAGATTTTTTAATATATTTTATTGATTTTTTACAGAGAGGAAGGGAGAGGGATAGAGCATTAGAAACATCGATGAGAGAGAAACATCGATCAGCTGCCTCCTGCACA
NW005871563.1_1 : GA------AATCAATAAAA-TATATTGATTTTTTACAGAGAGGGAGAGGGATAGATAGTTAGAAACATCGATGAGAAACATCAATCAGCTGCCTCCTGCACACTCCCTACTGGGGATGTGCCC
NW005871431.1_9 : GT--GAAAAATCAATAATA-TATATATATATATATTTAAAATATACTTTATTGATTTTTTACAGAGAGGAAGGGAGAGAGATAGAGAGTTAGAAATATCGATGAGAGAGAAACATCGATTAGC
NW005871069.1_9 : GT-AAAAAAATCAATAAAA-TATATTTCTTTAAAAAAAACAGAAAAAAAATGGTAATAAATTATCTAACCCTGCAACTTAAAGAATTAGAAAGAGAGCAACAAGAAAAGCCCACAGTAAGCAGA
NW005871112.1_3 : GT--AAAAAATCAATAAAG-TATATTTAAATGAATAAATAAATAAGCTGACTAGTGTCTACAGGAACCCCCATCCCCCCCCCCACGCTTCCCAATTTGATTAACATTGGGGATAGTGGACTAT
NW005871096.1_5 : GTAAAAAAAAATCAATAAAATATATTTTTAAAAAAGAAAGACTCATCTCAGTGTTAATTACAATAGCAAAACTTAATCAATAATGGGGGCATGTATGCATACAATGCAATTTTACAGTGCAAC
NW005871270.1_3 : GT--AAAAAATCAATAAAA-TATATATTTAAAAAAAAAGGAATGGGTTTCTGGGAAGAAGGTGGAAAACCAAGATGAGTTTGGTCTTTGGATGTGTTGAGTTTCAGTGTACTGTGGGACATCT
NW005871767.1_1 : GT-AAAAAAATCAATGAAA-CACATTTTTTAAAAAAATCAACTGGTATAAATACAGATGAAACAGGACAATAAGAGAGAACCTGGCTATGCTGATCAACTAAATGCTATCTCCGTGTCATTCC
NW005871176.1_3 : GT--AAAAAATCAATAAAA-TATAAAATAAATAAATAAATAAATACCGTAAGGCAATGACATCCCATTGGAAATACTATTAGTTCTCCCATCAAATTAGACTGAAATTAAAACCAGTTCAACA
NW005871060.1_1 : GT---AAAAATCAATAAAA-TATATATATATTTTTTAAAAGTACCACCATAGATATTTTATTAATGCATATTGAGTTCCTGAAACTCTTGGAAAGCTTCAGAATTGTTAGGGAAGTTACCAGA
NW005871091.1_4 : GA------AATCAATAAAAATATATTTTAAAAAATACGGAGGTTATCCTGGATAAGTGAGGTGGGCCTAGTGTAATTGTTATGCCCAGATTTCAGGATCCCCAAGAAACCACACCAGGGAGCC
NW005871534.1_3 : GA------AATCAATAAAA-TATATTTTTTTAAAAAGAAAGAGTATCTTTAAAAAAAGAAAAAAAAGAAAGAAAACTGTAGAAAGTTAGTAACTGCCACAAGCAAATGCCAGGCGGTGGCCAG
NW005871346.1_5 : GA------AATCAATAAAA-AACATTAAAAAAAAGATACAAATCTTTAAAAATTGTTAAATAAATATGGGGTGGGGGTGTAAAGAGAGAGGCAAATTTTTTACTCTATTGACTTCCATGTCAA
NW005871075.1_2 : GA------AATCAATAAAAATATATTTTTTTAAAAAGAATATTTCTTGAATGTATTGAATGCTCCCAAATGTTTTTAACACTAGAACAGATAAATAGCTTCTCTATATTTATGGCAGAATATT
NW005871048.1_1 : GA------AATCAATAAAAGAATTTTTTCTAAAATTCTACCTTTTCATGCTGGTGTTGGTTACCATAGTTGGGATTTTAAAATATTTTCAGTGTGAAGAAAGACAAGTCCACATGTTCCTTG
NW005871088.1_8 : GA------AATCAATAAAAAAAAATTAAAAAATGTTCATTCCTTCACTATCAGTCCGTGTGAGTCCTTTGTTCTGAGGTGGGTCTCTTGTAGACAGAAAATATATGGGTCATGTTTTCTTATC
NW005871126.1_9 : GA------AATCAATAAAAATATATTTAAAAAATAAGATTAGACAGAAACAACCAAATATCTGGAATCACAAATCTTATAATTATTGTTGAACTCAAATTTACAGTTGATATAGGTCCCTTCT
NW005871419.1_8 : GA------AATCAATAAAAATATATAATTTAAAAATAAATAAGCAAATAGAAGTTCAACACCACTCATTATGTGGATCTACAGTGAACAGTATCTATATGGTCAGAGTAACATAAAAACTTTA
NW005871067.1_7 : GA------AATCAATAAAAATATATTTTTAAAAAAGAGATATGTTTTCTTTGTAATCTAATGGAAGTATGTATGCTGTGTCCGTGTGTGTGTGTGTGTGTGTGTGTGTGAAAGCAGGTCAC
NW005871349.1_1 : GA------AATCAATAAAAAAAATATTTAAAAAATAAAAATGTGTAACTTGACTATAATTTGGCCATATAATTCCAGGGTATAAGAAATAGAGAGGATAGAGAATTAATTTAAAGGATACCAC
NW005871109.1_5 : AA------AATCAGTAAAAATATATTTTAAAAAATAAATAAATAAAAGAATATCTGAATACTGGTGGATTATGACCAAATCACTAAGCTAGTAAAGAGAGGAGGAAGGATAGATTTGGAGCAA
NW005874004.1_1 : GA------AATCAATAAAAATATATTTAAAAAAATAACAATTGACAGAAAGATTTCCAAATTCTTCTCCAGTGAGATTATCTTTTTAAAGTCAATGGCCACATCCATTTCAATTTTTGTTTTC
NW005871206.1_6 : GA------AATCAATAAAAAAAATATTAAAAAAATAAAAATAAATAAAATGCGTAACAGGAAGAATAGAAATTATATTGAAATATATAGTTCTAGCATCTGACCCCTGAGATTTTCAAGGAGT
NW005871123.1_4 : GA------AATCAATAAAAATATATTTAAAAATAATAAAAGAATAAGATTGCTTTGCTATCAAAGCCAAGTAAGGTGTGTCATTTGTTGTCCCTGATGAAGTCTGAGCAGCTCAGTTGCTAAG
NW005871055.1_6 : GA------AATCAATAAAAATATATATTTTTAAACACAGTCTGTTAGAAATGTTCATTCCCCTAATTGAGGCTCATTCTTGATCTCTACTGGCTGAGCTTTACTGTCAGTGCCCTAGATTTTA
NW005871068.1_9 : GA------AATCAATAATAAAGAAAAGAAGAGTACGCAAGTCAGTTGTAGTACATAAACACTTCAGCACCACGTAGTTTATTAAGTTCTCATTTTTGTTTGGGGTGCACTTAATCACTGTTTA
NW005871516.1_4 : GA------AATCAATAAAAATATATTTAAAAAATAAAAATAAAAAGGGGGGACATGTGGGCACAGGCGTGCCCAGGGGGAAGACCATGTGAAGAGGCAGCATCCACAAGCCCAGCCGAGAGGC
NW005871085.1_3 : GA------AATTAATTATATATATATTAAAGCTATAATTGAAAATATCAGCTTTAGTACTGGTGAATCTGGATTGAAAATTCTGCTGAAATATGTAGCCACAATCCTTCCTGAAACTTAC
NW005871063.1_5 : GA------TATCAATAAAGATATGTTTAAAACAAAACAAAACAAAACAAAACAAAAAACCAGTGTTACACAATGGAATACTATGCTGCTATAAAAAAGGAGCTCTTACCATTCACAACAGCAT
NW005871093.1_5 : GA------AATCAGTAAAAAAAAATATTTTTAAAAAGGCAGCTAGTAAGACTGGAAAGGTCTGGGCCTTGAGGGGCAGCCAGCTCAATCCAACAAGCAGAGATGAACCTCCACTGGTTATTAC
NW005881170.1_2 : GA------AATCAATTTAAAAAAACAACAACAAGCATTAATCAAGCTATTATACAAATAAGTACTGAAACTGTGGTAATCATTATCAAAGAGAGCAATGGATTTACAGGAGCATAAACAGAGA
NW005871097.1_6 : GA------AATTAATAAAAATACATATTTTTTAAAACTTGCTCAGGGACTCCTTGGCCTCCAATAGAAGAGGAATGGTAATATAAAATATATCCATGTGATGAAACAAAACACATCTCAAACT
NW005871464.1_5 : GA------AATCAATAAAAATATATGTATTAAAAAAGAATAGTCTGAGGTGGTTGCTTGCTGCTGTTGCCATGTCATGCTTTGTAAGAGAAAGTGAAAAGACTGCTATGGAAGGCTGGGTTAG
NW005871049.1_2 : GA------AGTCAATATAAAAAAATATATTTAAAAAAGTTAAATACATAGTGAGAAGCAAAGGGGAAAAGAAGGCATAGGTTAATATATTTGAGATAATGTGCAAACAGAGTGAAAGACCACA
NW005871111.1_3 : GA------AATCAATAAAAAATATATTTTAAAAAAACAAACCTGATGAATAGCTTGCCTGGGATGACACAGCTAATGGATTGACACAGCTGAGATTAGCACCTGATTCCAAAGTCCATGCTCT
```

```
NW005871143.1_1 : GA------AATCAATAAAAAGATTAAAAAAATAAAAGTACTTATCAAAAATGTGTGGACAGTACAAGCATGGACTAAGATGCTCGAGAACGGAAACCAACACCAGTTCTCTCACTGACCACAC
NW005872372.1_5 : GA------AATCAGTAAAGACATATGTTCACCAGCCACATTTCAAGTCCTTAATGCCCATGACGACTCGTGTCTACTGTGGGTCGATCGCATTGTGTTCGCCAAAAGACTATGTTACTATCCA
NW005871909.1_5 : GA------AATCAATAAAAATATGAAACAAATAAAAAAATTTCATAACTTTTACAATATCCTGTTATACATAATTATTAATAATGAACTACAATGTTCTAATGACTGATTACTATAATCGTGT
NW005875787.1_1 : TA------TATATATATATATATATATAAAATAAATAAAAACACCATGTCCCAGGCAACCATTTGAAAATAAAAACACAATAGTAATATCTCTTGCAGATAATATTCATTCAATTCAGTAAAA
NW005872538.1_1 : GA------AATCAATAAAGAATATATATTTGGAGAAAAAAAAACAATGCTACATCATTGATTGTCATCTGGTAGTAACATAAGGGTAATGAGAGAAACATGAATAAATGTTTAAGAACTACAC
NW005876019.1_8 : GA------AATCAATAAAAATATGTTTAAAAAATACGAATAAAGACACAAGAATGGAAAGGTATACTCTGCTCACTAATTGGAAAAATTAATATTGTTATGCCCTACCACAGGCTCAAAAGGG
NW005871054.1_8 : CT-CTAAAACTCAATAAAAAATATTTTTAAAATATGGCATTAAAATATTTTTCTTGATTGCTGAATTTTTTCGTGTCTTCTTAAATTTTGTACTGGAGGTGAGTACCTGAACCACATCCTTG
NW005871074.1_1 : G-------AATCAATGAAAATATATTTTAAAAAAAGAATTGAGATAGTAAATGCATAATGCCATCTTTAAAAATTTGTCCTTTGATTTCACATCCACTTTCAGAACTGCCTTCGGACTTGGCT
NW005871068.1_3 : GA-----AAATGAATAAAAGTATATTAAAATATATTGTAGGTCCAATGACCAATAACCTAGATTATTTTTGCAAAGAAATTAGTAGATATAAGAACATTATATCATTACAGGATAAAGTGGAG
NW005871151.1_6 : GA------AATCAATAAAAATATATTAAAAAATAAAAACAAATAAATGAATAAGTACAGTCACATGGGGTGTCAGGCCTGCAATATATAAATTTGGGGGGACAGGTCAGTCCATAACACCAGG
NW005872725.1_2 : GA------AATCAATAAAGAAGTGTATTTTTTAAAAAGGAGACTTTTATCAAAGCTTTTCTTCTCAGTACCCTTTACATTTAGTGTCCACAGATAACAAAGATGTGCACAAAAGCGCTATAGA
NW005871311.1_1 : GA----------AATAAAAATATTTTTTTAAAAAGAATGTCTTCAGCTTTTGACCTTTTATTTTCCAATATCAGATTATTTACTCTGCTCTGATATCCCCAGGAAGCTTTAAAGAAAATGCAG
NW005871048.1_6 : GT-AAAAAAATCAATCATAGGGAATTGTGTGTTACAATAACATTAAAAATGTATACCAGTGATATATATTTTGGAAAATACTGAAAAGTATGCAGAAGAAAATAAAAATAGCTGTTAATATTA
NW005871163.1_1 : CT---AAATATCAATAAAAACATATTTTTAAAAAAACCCCAAACACTATGATTCAGTAATTCCACTCTGAAAACCTGTCCACACCAAAACTTGTACATGAATGTTCACAGCAGCATTATTTAT
NW005871179.1_3 : GA------AATCAACACAAATATATTTAAAAACAAAACAAAACTCCAAGGAAAACACCTCAGTTTTCACTCAGCAAAAACTGGGTTCTCTTGAGTCACATGGTACCTTGTGTCAGGCAGTGAG
NW005871172.1_1 : CT--CTCTCTCTCCCCCTTTTCCCTCCCTTATACTCTCTCTAAAAATCAATGGAAAAAATATCCTCAGATGAGAATTAACCAAAAAAAAAAAAAGAAAAGAAAAAAAAGAAATCATTAACAAAAT
NW005871095.1_2 : CT--CTCTCTCTCCCTCTCTCCCTCTCTCTCTCTTTCCCCCTCTCAACTCTTTCTTAAACTCAATGGAAAAATATATCCTCAGGTGAGGATTTACAAATAAATAAAGCATTGTTTTCTGCA
NW005871196.1_3 : CT--CTCTCTCTCTCTCCCCCTCCCCATTTCCTCCCCTCCACTCTCTCTAAAAATCTATGGAAATTAACCAAAAAAAAGGGAAACAAAATATTATATACAGCAAAACTATCTTTCAAAAATTAA
NW005871133.1_1 : CT--TCCACCCCTCACCCCCCTCCTACATCACCCTTCCACTCTCTCTGAAAAGCAATGGAAAAAATATCCTCGGGTGAAGATTAACTATATAAATATCTCCAAAATCCCTTCAAATACCTTTG
```

Figure S24. Random Ves_a copies from the *M. lucifugus* genome. Nucleotide sequences are highlighted as follows: PAS, strong pol III terminators, medium terminators, rudimentary terminators, and oligo(A). Four lower copies have T¯ tails; their specific motif preceding an A-rich tail is marked in grey. The β signal sequences are shown in yellow (AGGGCATGT) or in red (AGGGCACAT) characters for Ves_a1 and Ves_a2, respectively. Other Ves_a2-specific downstream nucleotides are also shown in red characters.
