# Supplementary material for: Analysis of SINE Families B2, Dip, and Ves with Special Reference to Polyadenylation Signals and Transcription Terminators
Source: Int J Mol Sci. 2021 Sep 13;22(18):9897. doi: 10.3390/ijms22189897 (PMC8466645; doi:10.3390/ijms22189897)
Supplement: Supplementary file 1 [file ijms-22-09897-s001.zip › Supplementary Vassetzky/SINE Ves. Figs S23-S32. Table S9/Fig S25.pdf]

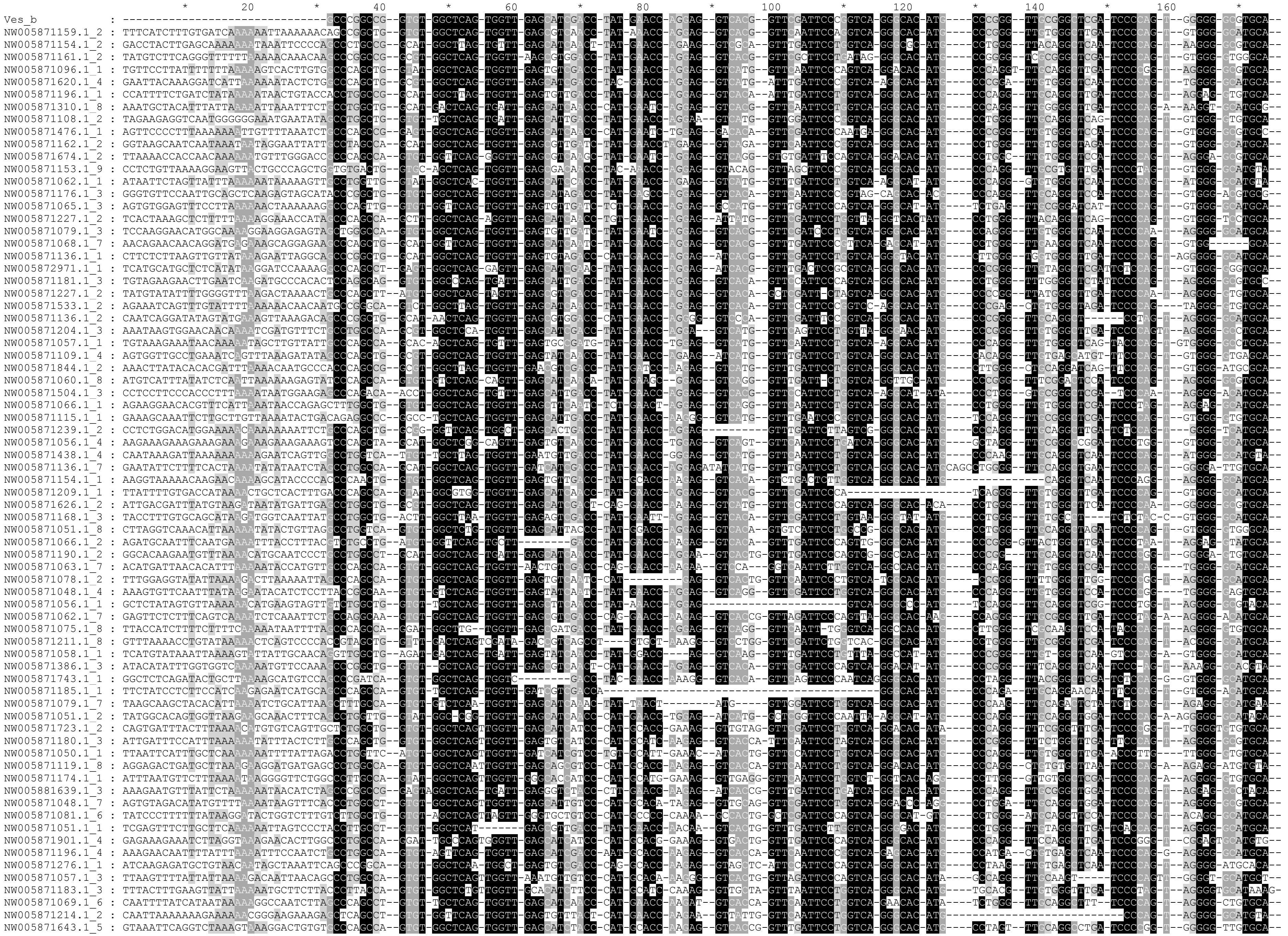
* 20 * 40 * 60 * 80 * 100 * 120 * 140 * 160 *
Ves_b
NW005871159.1_2
NW005871154.1_2
NW005871161.1_2
NW005871096.1_1
NW005871620.1_4
NW005871196.1_1
NW005871310.1_8
NW005871108.1_2
NW005871476.1_1
NW005871162.1_2
NW005871674.1_2
NW005871153.1_9
NW005871062.1_1
NW005871176.1_3
NW005871065.1_3
NW005871227.1_2
NW005871079.1_3
NW005871068.1_7
NW005871136.1_1
NW005872971.1_1
NW005871181.1_3
NW005871227.1_2
NW005871533.1_2
NW005871136.1_2
NW005871204.1_3
NW005871057.1_1
NW005871109.1_4
NW005871844.1_2
NW005871060.1_8
NW005871504.1_3
NW005871066.1_1
NW005871115.1_1
NW005871239.1_2
NW005871056.1_4
NW005871438.1_4
NW005871136.1_7
NW005871154.1_1
NW005871209.1_1
NW005871626.1_2
NW005871168.1_3
NW005871051.1_8
NW005871066.1_2
NW005871190.1_2
NW005871063.1_7
NW005871078.1_2
NW005871048.1_4
NW005871056.1_1
NW005871062.1_7
NW005871075.1_8
NW005871211.1_8
NW005871058.1_1
NW005871386.1_3
NW005871743.1_1
NW005871185.1_1
NW005871079.1_7
NW005871051.1_2
NW005871723.1_2
NW005871180.1_3
NW005871050.1_1
NW005871119.1_8
NW005871174.1_1
NW005881639.1_3
NW005871048.1_7
NW005871081.1_6
NW005871051.1_1
NW005871901.1_4
NW005871196.1_4
NW005871276.1_1
NW005871057.1_3
NW005871183.1_3
NW005871069.1_6
NW005871214.1_2
NW005871643.1_5

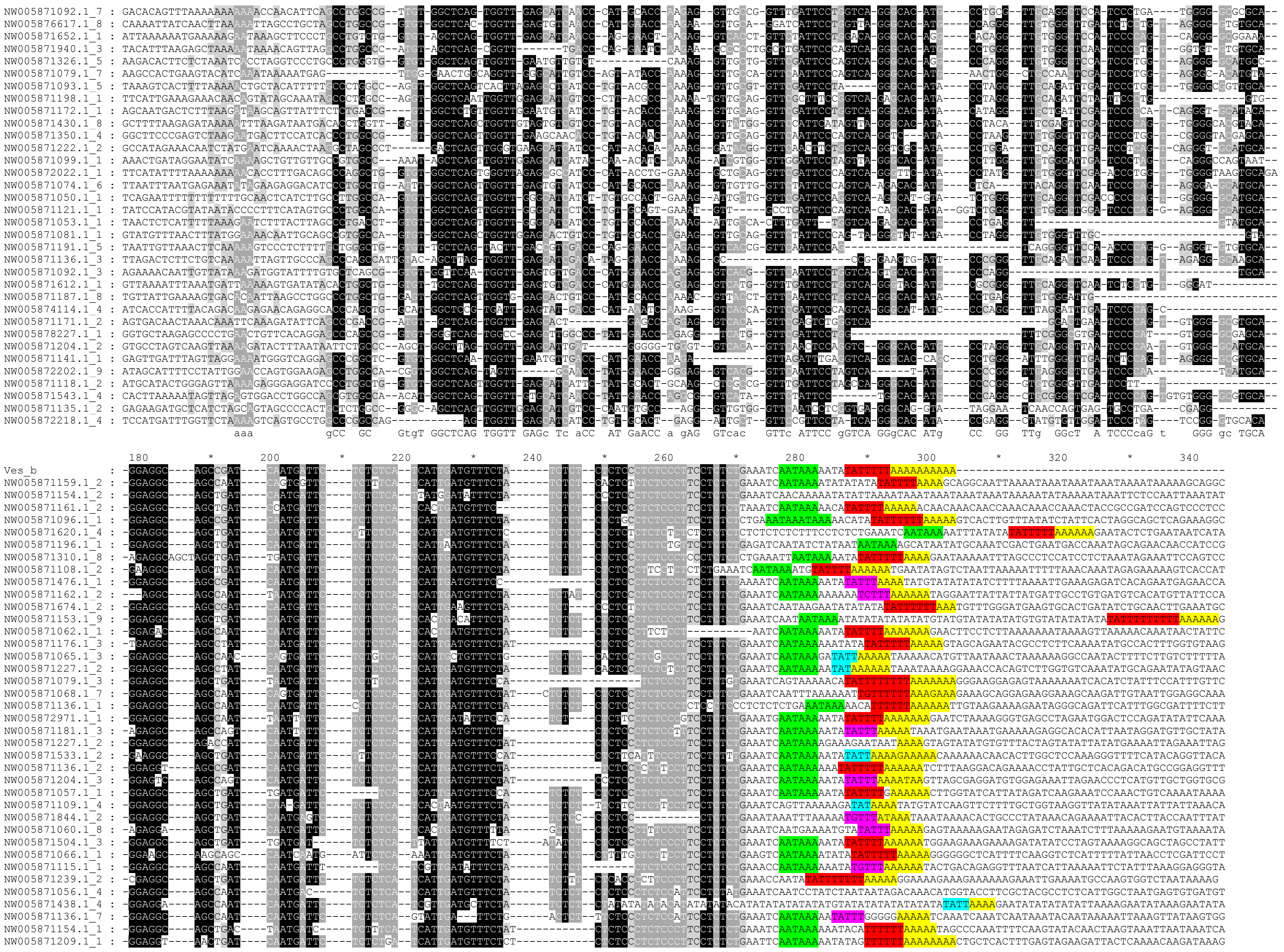

NW005871092.1_7
NW005876617.1_8
NW005871652.1_1
NW005871940.1_3
NW005871326.1_5
NW005871079.1_7
NW005871093.1_5
NW005871198.1_1
NW005871172.1_1
NW005871430.1_8
NW005871350.1_4
NW005871222.1_2
NW005871099.1_1
NW005872022.1_1
NW005871074.1_6
NW005871050.1_1
NW005871121.1_1
NW005871053.1_1
NW005871081.1_1
NW005871191.1_5
NW005871136.1_3
NW005871092.1_3
NW005871612.1_1
NW005871187.1_8
NW005874114.1_4
NW005871171.1_2
NW005878227.1_1
NW005871204.1_2
NW005871141.1_1
NW005872202.1_9
NW005871118.1_2
NW005871543.1_4
NW005871135.1_2
NW005872218.1_4
180 * 200 * 220 * 240 * 260 * 280 * 300 * 320 * 340
Ves_b
NW005871159.1_2
NW005871154.1_2
NW005871161.1_2
NW005871096.1_1
NW005871620.1_4
NW005871196.1_1
NW005871310.1_8
NW005871108.1_2
NW005871476.1_1
NW005871162.1_2
NW005871674.1_2
NW005871153.1_9
NW005871062.1_1
NW005871176.1_3
NW005871065.1_3
NW005871227.1_2
NW005871079.1_3
NW005871068.1_7
NW005871136.1_1
NW005872971.1_1
NW005871181.1_3
NW005871227.1_2
NW005871533.1_2
NW005871136.1_2
NW005871204.1_3
NW005871057.1_1
NW005871109.1_4
NW005871844.1_2
NW005871060.1_8
NW005871504.1_3
NW005871066.1_1
NW005871115.1_1
NW005871239.1_2
NW005871056.1_4
NW005871438.1_4
NW005871136.1_7
NW005871154.1_1
NW005871209.1_1

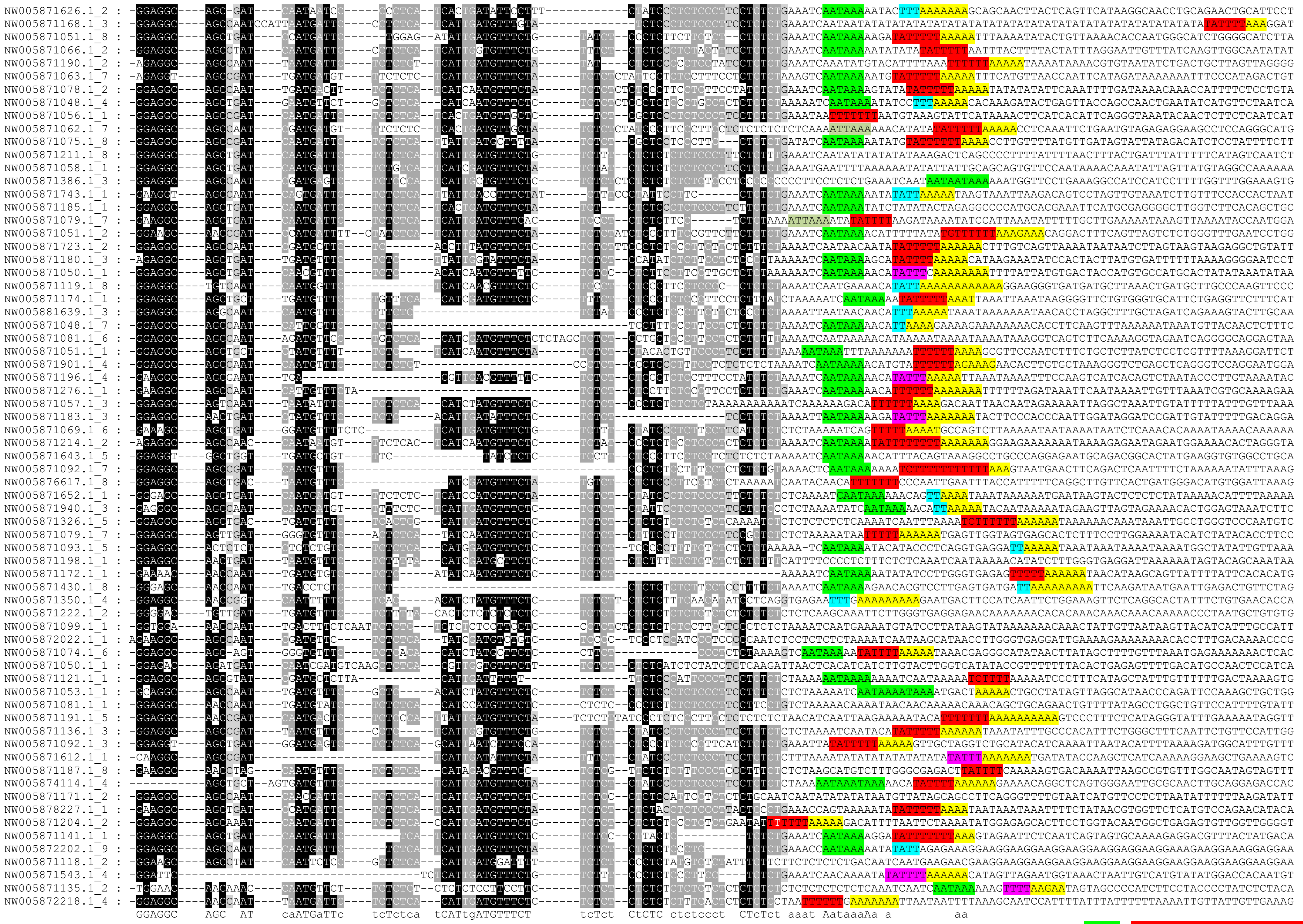

Figure S25. Random Ves_b copies from the *M. lucifugus* genome. Nucleotide sequences are highlighted as follows: PAS, strong pol III terminators, medium terminators, rudimentary terminators, and oligo(A).
