# Supplementary material for: Analysis of SINE Families B2, Dip, and Ves with Special Reference to Polyadenylation Signals and Transcription Terminators
Source: Int J Mol Sci. 2021 Sep 13;22(18):9897. doi: 10.3390/ijms22189897 (PMC8466645; doi:10.3390/ijms22189897)
Supplement: Supplementary file 1 [file ijms-22-09897-s001.zip › Supplementary Vassetzky/SINE Ves. Figs S23-S32. Table S9/Fig S26.pdf]

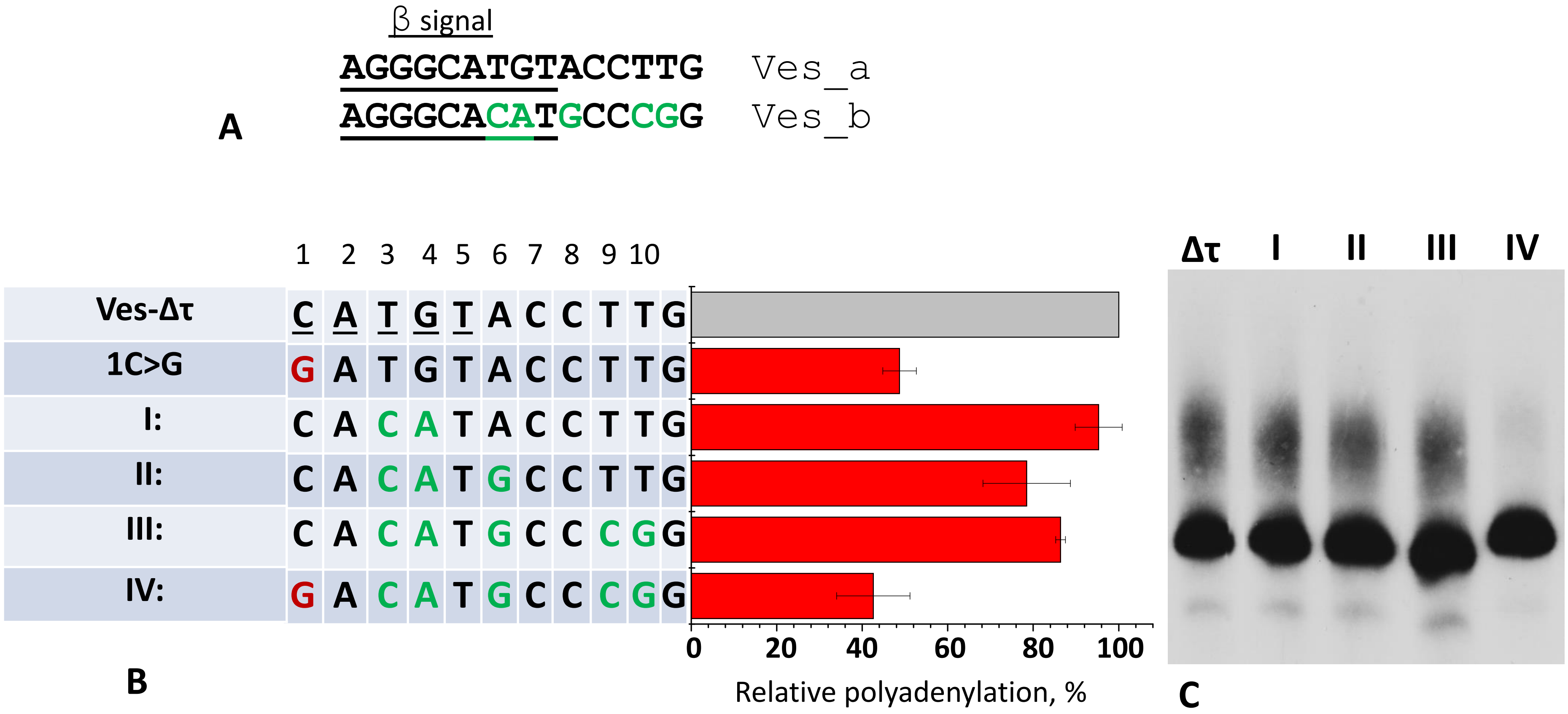

Figure S26. Testing the ability of the second β signal variant (AGGGCACAT) characteristic of the Ves_b, to activate polyadenylation of Ves transcripts. (**A**) Nucleotide sequences of two variants of the β-signal (underlined) with the downstream region. The Ves_b nucleotides distinct from those in Ves_a are shown in green. (**B**) Substitutions converting the β signal of Ves_a into of Ves_b one are highlighted in green. (**C**) Northern hybridization of RNA isolated from HeLa cells transfected by Ves-containing constructs. The band and smear correspond to the primary and polyadenylated transcripts of Ves, respectively. The diagram demonstrates the relative polyadenylation of Ves transcripts for the five constructs. The polyadenylation level of Ves_a transcripts with the first β signal variant (construct Ves-Δτ) was taken as 100%.
