# Supplementary material for: Analysis of SINE Families B2, Dip, and Ves with Special Reference to Polyadenylation Signals and Transcription Terminators
Source: Int J Mol Sci. 2021 Sep 13;22(18):9897. doi: 10.3390/ijms22189897 (PMC8466645; doi:10.3390/ijms22189897)
Supplement: Supplementary file 1 [file ijms-22-09897-s001.zip › Supplementary Vassetzky/SINE Ves. Figs S23-S32. Table S9/Fig S27B.pdf]

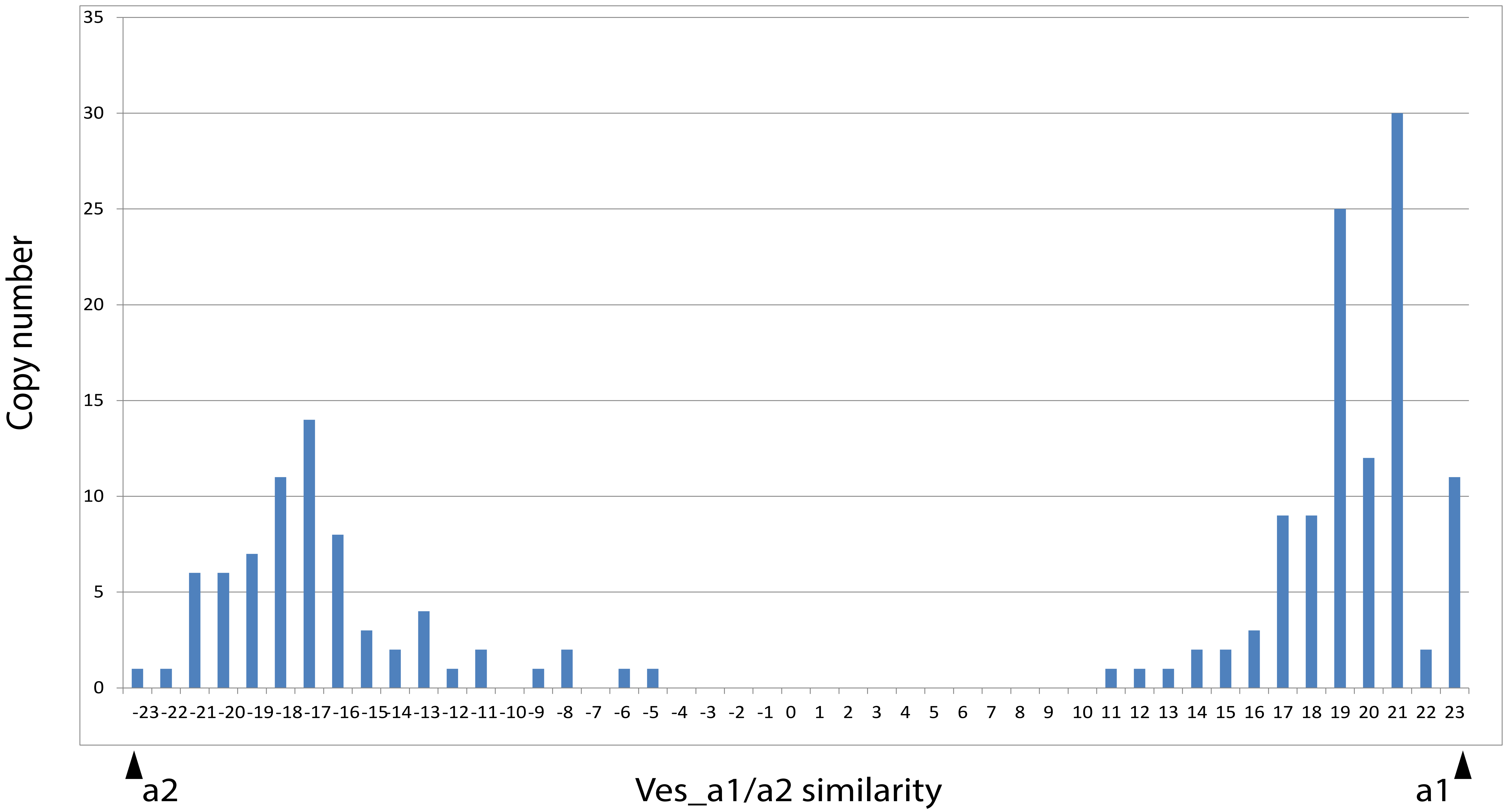

Figure S27B. Distribution of random Ves_a copies according to the degree of their similarity to a1 and a2 consensus sequences.
