# Supplementary material for: Analysis of SINE Families B2, Dip, and Ves with Special Reference to Polyadenylation Signals and Transcription Terminators
Source: Int J Mol Sci. 2021 Sep 13;22(18):9897. doi: 10.3390/ijms22189897 (PMC8466645; doi:10.3390/ijms22189897)
Supplement: Supplementary file 1 [file ijms-22-09897-s001.zip › Supplementary Vassetzky/SINE Ves. Figs S23-S32. Table S9/Fig S28.pdf]

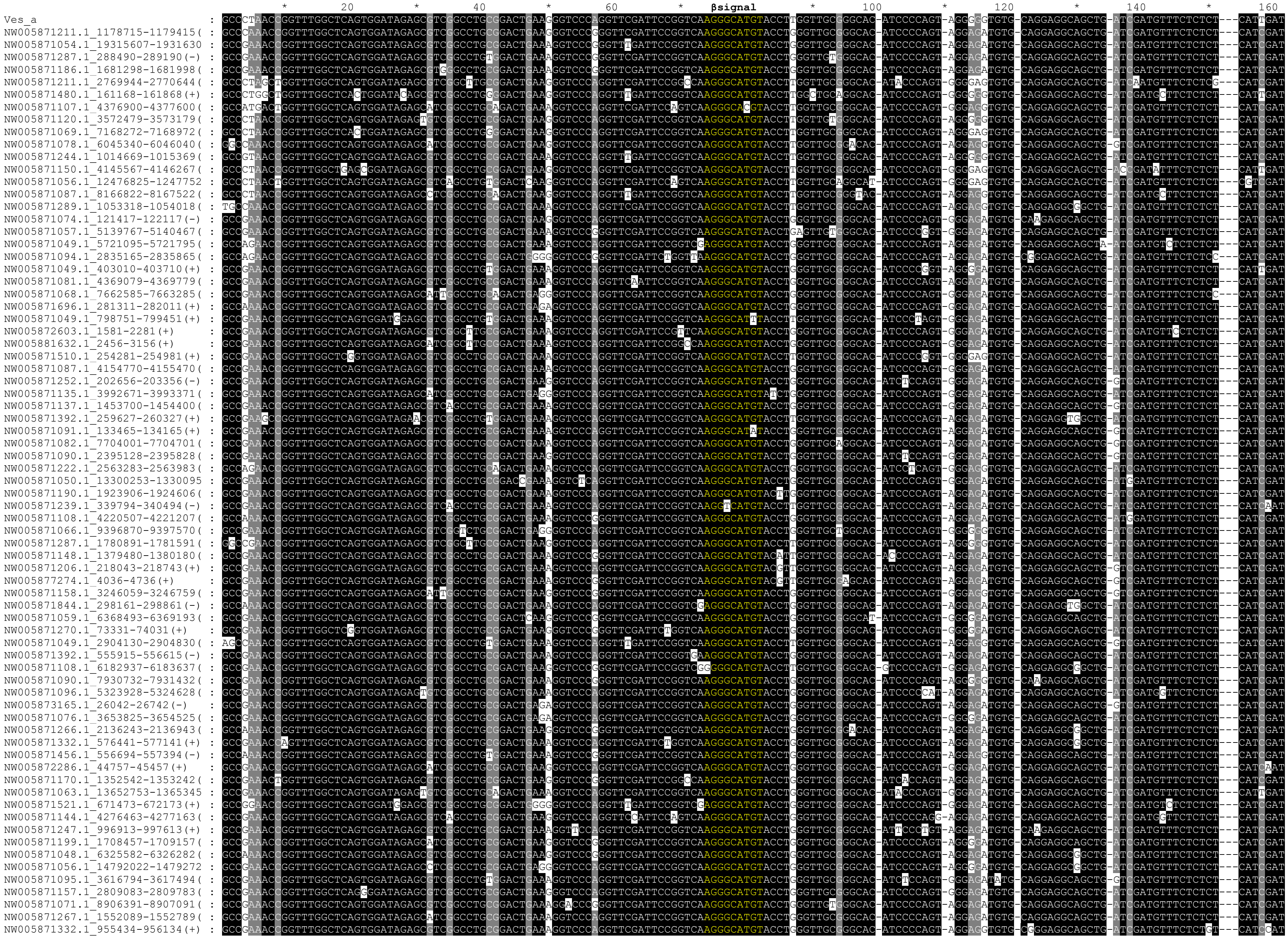
20
40
60
βsignal
100
120
140
160

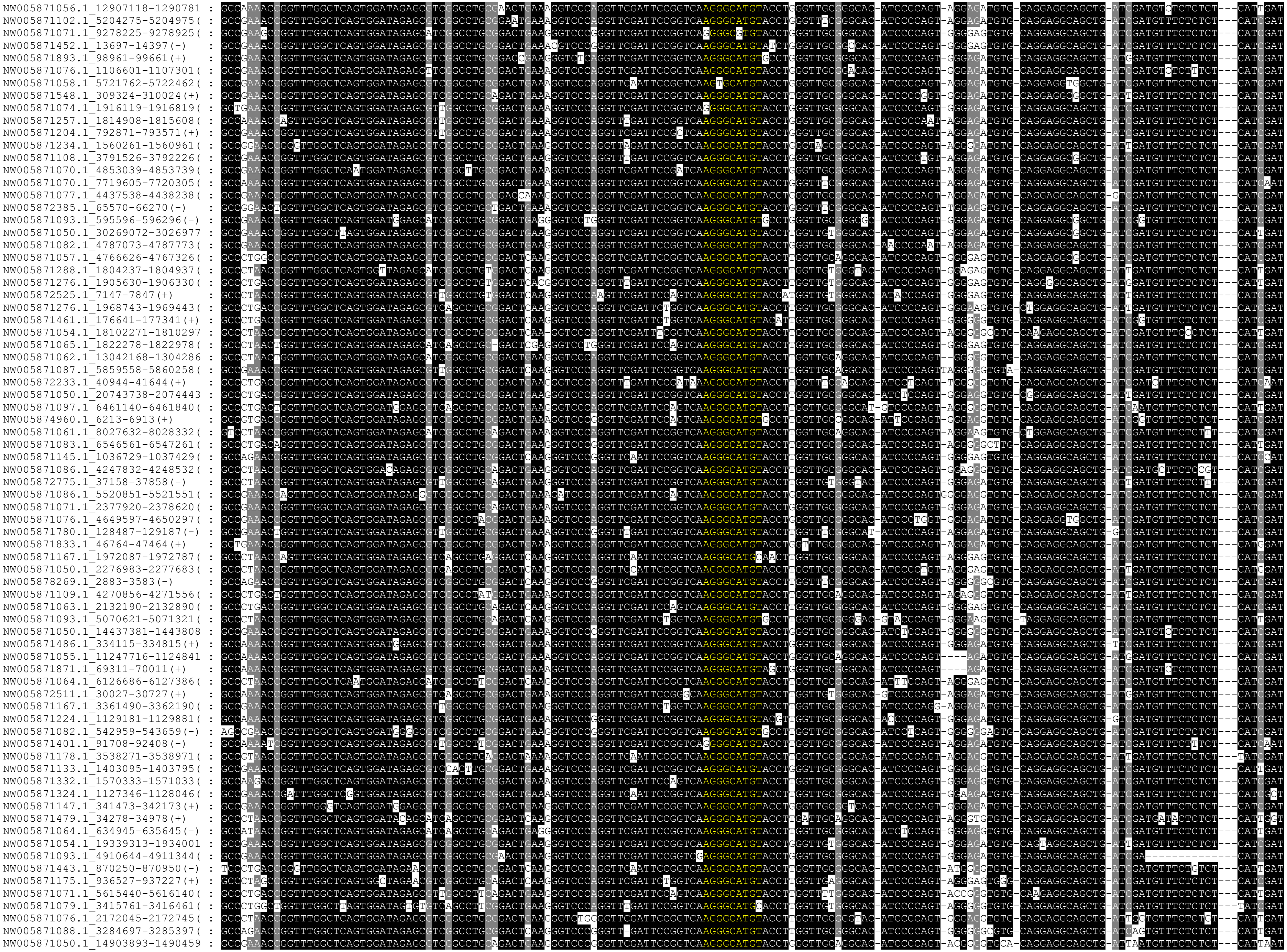
NW005871056.1_12907118-1290781 :
NW005871102.1_5204275-5204975( :
NW005871071.1_9278225-9278925( :
NW005871452.1_13697-14397(-) :
NW005871893.1_98961-99661(+) :
NW005871076.1_1106601-1107301( :
NW005871058.1_5721762-5722462( :
NW005871548.1_309324-310024(+) :
NW005871074.1_1916119-1916819( :
NW005871257.1_1814908-1815608( :
NW005871204.1_792871-793571(+) :
NW005871234.1_1560261-1560961( :
NW005871108.1_3791526-3792226( :
NW005871070.1_4853039-4853739( :
NW005871070.1_7719605-7720305( :
NW005871077.1_4437538-4438238( :
NW005872385.1_65570-66270(-) :
NW005871093.1_595596-596296(-) :
NW005871050.1_30269072-3026977 :
NW005871082.1_4787073-4787773( :
NW005871057.1_4766626-4767326( :
NW005871288.1_1804237-1804937( :
NW005871276.1_1905630-1906330( :
NW005872525.1_7147-7847(+) :
NW005871276.1_1968743-1969443( :
NW005871461.1_176641-177341(+) :
NW005871054.1_18102271-1810297 :
NW005871065.1_1822278-1822978( :
NW005871062.1_13042168-1304286 :
NW005871087.1_5859558-5860258( :
NW005872233.1_40944-41644(+) :
NW005871050.1_20743738-2074443 :
NW005871097.1_6461140-6461840( :
NW005874960.1_6213-6913(+) :
NW005871061.1_8027632-8028332( :
NW005871083.1_6546561-6547261( :
NW005871145.1_1036729-1037429( :
NW005871086.1_4247832-4248532( :
NW005872775.1_37158-37858(-) :
NW005871086.1_5520851-5521551( :
NW005871071.1_2377920-2378620( :
NW005871076.1_4649597-4650297( :
NW005871780.1_128487-129187(-) :
NW005871833.1_46764-47464(+) :
NW005871167.1_1972087-1972787( :
NW005871050.1_2276983-2277683( :
NW005878269.1_2883-3583(-) :
NW005871109.1_4270856-4271556( :
NW005871063.1_2132190-2132890( :
NW005871093.1_5070621-5071321( :
NW005871050.1_14437381-1443808 :
NW005871486.1_334115-334815(+) :
NW005871055.1_11247716-1124841 :
NW005871871.1_69311-70011(+) :
NW005871064.1_6126686-6127386( :
NW005872511.1_30027-30727(+) :
NW005871167.1_3361490-3362190( :
NW005871224.1_1129181-1129881( :
NW005871082.1_542959-543659(-) :
NW005871401.1_91708-92408(-) :
NW005871178.1_3538271-3538971( :
NW005871133.1_1403095-1403795( :
NW005871332.1_1570333-1571033( :
NW005871324.1_1127346-1128046( :
NW005871147.1_341473-342173(+) :
NW005871479.1_34278-34978(+) :
NW005871064.1_634945-635645(-) :
NW005871054.1_19339313-1934001 :
NW005871093.1_4910644-4911344( :
NW005871443.1_870250-870950(-) :
NW005871175.1_936527-937227(+) :
NW005871071.1_5615440-5616140( :
NW005871079.1_3415761-3416461( :
NW005871076.1_2172045-2172745( :
NW005871088.1_3284697-3285397( :
NW005871050.1_14903893-1490459 :

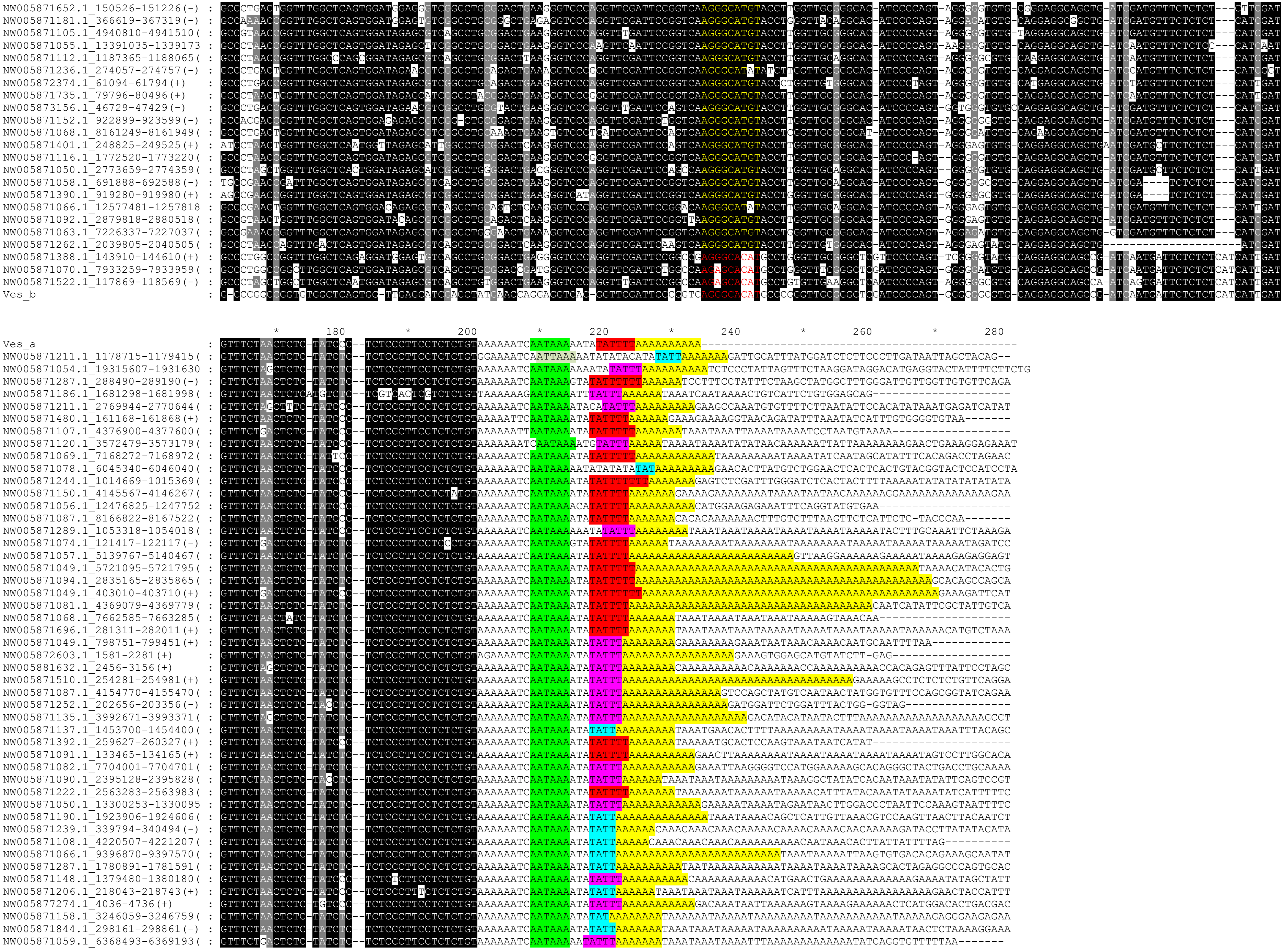
NW005871652.1_150526-151226(-)
NW005871181.1_366619-367319(-)
NW005871105.1_4940810-4941510(
NW005871055.1_13391035-1339173
NW005871112.1_1187365-1188065(
NW005871236.1_274057-274757(-)
NW005872374.1_61094-61794(+)
NW005871735.1_79796-80496(+)
NW005873156.1_46729-47429(-)
NW005871152.1_922899-923599(-)
NW005871068.1_8161249-8161949(
NW005871401.1_248825-249525(+)
NW005871116.1_1772520-1773220(
NW005871050.1_2773659-2774359(
NW005871058.1_691888-692588(-)
NW005871390.1_919280-919980(+)
NW005871066.1_12577481-1257818
NW005871092.1_2879818-2880518(
NW005871063.1_7226337-7227037(
NW005871262.1_2039805-2040505(
NW005871388.1_143910-144610(+)
NW005871070.1_7933259-7933959(
NW005871522.1_117869-118569(-)
Ves_b
180
200
220
240
260
280
Ves_a
NW005871211.1_1178715-1179415(
NW005871054.1_19315607-1931630
NW005871287.1_288490-289190(-)
NW005871186.1_1681298-1681998(
NW005871211.1_2769944-2770644(
NW005871480.1_161168-161868(+)
NW005871107.1_4376900-4377600(
NW005871120.1_3572479-3573179(
NW005871069.1_7168272-7168972(
NW005871078.1_6045340-6046040(
NW005871244.1_1014669-1015369(
NW005871150.1_4145567-4146267(
NW005871056.1_12476825-1247752
NW005871087.1_8166822-8167522(
NW005871289.1_1053318-1054018(
NW005871074.1_121417-122117(-)
NW005871057.1_5139767-5140467(
NW005871049.1_5721095-5721795(
NW005871094.1_2835165-2835865(
NW005871049.1_403010-403710(+)
NW005871081.1_4369079-4369779(
NW005871068.1_7662585-7663285(
NW005871696.1_281311-282011(+)
NW005871049.1_798751-799451(+)
NW005872603.1_1581-2281(+)
NW005881632.1_2456-3156(+)
NW005871510.1_254281-254981(+)
NW005871087.1_4154770-4155470(
NW005871252.1_202656-203356(-)
NW005871135.1_3992671-3993371(
NW005871137.1_1453700-1454400(
NW005871392.1_259627-260327(+)
NW005871091.1_133465-134165(+)
NW005871082.1_7704001-7704701(
NW005871090.1_2395128-2395828(
NW005871222.1_2563283-2563983(
NW005871050.1_13300253-1330095
NW005871190.1_1923906-1924606(
NW005871239.1_339794-340494(-)
NW005871108.1_4220507-4221207(
NW005871066.1_9396870-9397570(
NW005871287.1_1780891-1781591(
NW005871148.1_1379480-1380180(
NW005871206.1_218043-218743(+)
NW005877274.1_4036-4736(+)
NW005871158.1_3246059-3246759(
NW005871844.1_298161-298861(-)
NW005871059.1_6368493-6369193(

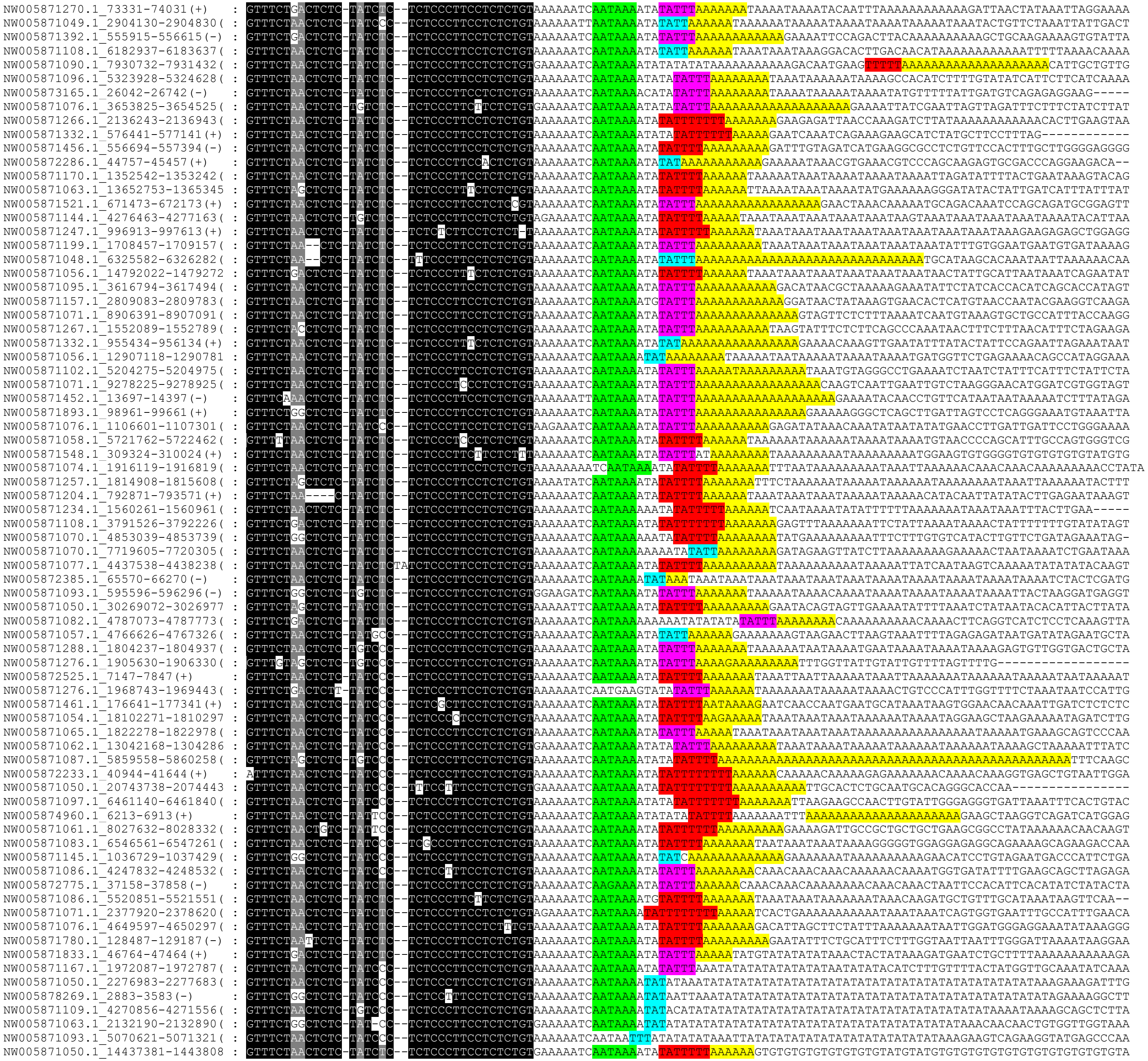
NW005871270.1_73331-74031(+)
NW005871049.1_2904130-2904830(
NW005871392.1_555915-556615(-)
NW005871108.1_6182937-6183637(
NW005871090.1_7930732-7931432(
NW005871096.1_5323928-5324628(
NW005873165.1_26042-26742(-)
NW005871076.1_3653825-3654525(
NW005871266.1_2136243-2136943(
NW005871332.1_576441-577141(+)
NW005871456.1_556694-557394(-)
NW005872286.1_44757-45457(+)
NW005871170.1_1352542-1353242(
NW005871063.1_13652753-1365345
NW005871521.1_671473-672173(+)
NW005871144.1_4276463-4277163(
NW005871247.1_996913-997613(+)
NW005871199.1_1708457-1709157(
NW005871048.1_6325582-6326282(
NW005871056.1_14792022-1479272
NW005871095.1_3616794-3617494(
NW005871157.1_2809083-2809783(
NW005871071.1_8906391-8907091(
NW005871267.1_1552089-1552789(
NW005871332.1_955434-956134(+)
NW005871056.1_12907118-1290781
NW005871102.1_5204275-5204975(
NW005871071.1_9278225-9278925(
NW005871452.1_13697-14397(-)
NW005871893.1_98961-99661(+)
NW005871076.1_1106601-1107301(
NW005871058.1_5721762-5722462(
NW005871548.1_309324-310024(+)
NW005871074.1_1916119-1916819(
NW005871257.1_1814908-1815608(
NW005871204.1_792871-793571(+)
NW005871234.1_1560261-1560961(
NW005871108.1_3791526-3792226(
NW005871070.1_4853039-4853739(
NW005871070.1_7719605-7720305(
NW005871077.1_4437538-4438238(
NW005872385.1_65570-66270(-)
NW005871093.1_595596-596296(-)
NW005871050.1_30269072-3026977
NW005871082.1_4787073-4787773(
NW005871057.1_4766626-4767326(
NW005871288.1_1804237-1804937(
NW005871276.1_1905630-1906330(
NW005872525.1_7147-7847(+)
NW005871276.1_1968743-1969443(
NW005871461.1_176641-177341(+)
NW005871054.1_18102271-1810297
NW005871065.1_1822278-1822978(
NW005871062.1_13042168-1304286
NW005871087.1_5859558-5860258(
NW005872233.1_40944-41644(+)
NW005871050.1_20743738-2074443
NW005871097.1_6461140-6461840(
NW005874960.1_6213-6913(+)
NW005871061.1_8027632-8028332(
NW005871083.1_6546561-6547261(
NW005871145.1_1036729-1037429(
NW005871086.1_4247832-4248532(
NW005872775.1_37158-37858(-)
NW005871086.1_5520851-5521551(
NW005871071.1_2377920-2378620(
NW005871076.1_4649597-4650297(
NW005871780.1_128487-129187(-)
NW005871833.1_46764-47464(+)
NW005871167.1_1972087-1972787(
NW005871050.1_2276983-2277683(
NW005878269.1_2883-3583(-)
NW005871109.1_4270856-4271556(
NW005871063.1_2132190-2132890(
NW005871093.1_5070621-5071321(
NW005871050.1_14437381-1443808

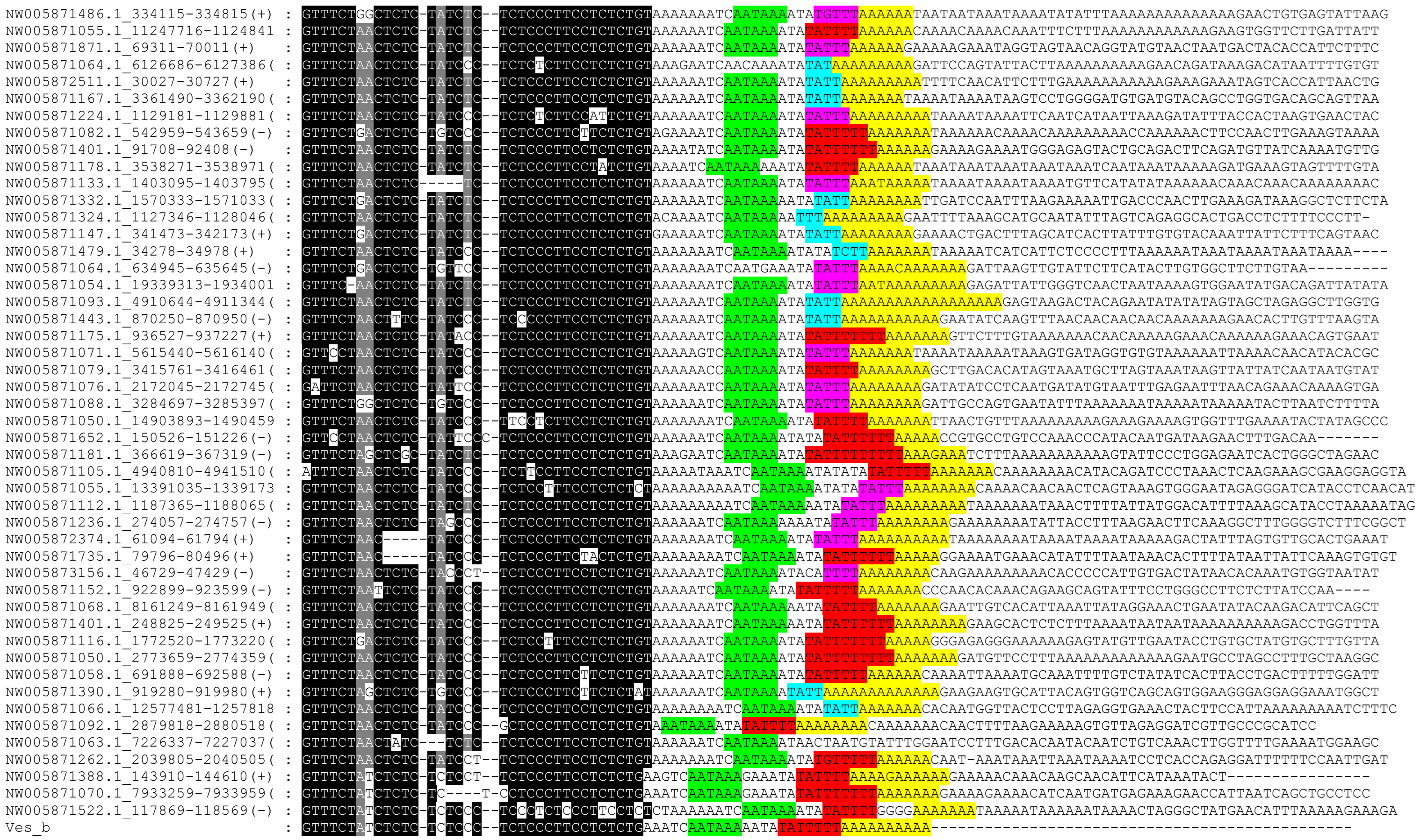

**Figure S28.** Random Ves copies present in the *M. lucifugus* genome but absent in homologous *M. myotis* loci. The nucleotide sequences are highlighted as follows: PAS, strong pol III terminators, medium terminators, rudimentary terminators, and oligo(A). All copies are Ves_a1 except the three lowest ones that are Ves_a2. The β signal sequences are shown in yellow (AGGGCATGT) or red (AGGGCACAT) characters for Ves_a1 and Ves_a2, respectively.
