# Supplementary material for: Analysis of SINE Families B2, Dip, and Ves with Special Reference to Polyadenylation Signals and Transcription Terminators
Source: Int J Mol Sci. 2021 Sep 13;22(18):9897. doi: 10.3390/ijms22189897 (PMC8466645; doi:10.3390/ijms22189897)
Supplement: Supplementary file 1 [file ijms-22-09897-s001.zip › Supplementary Vassetzky/SINE Ves. Figs S23-S32. Table S9/Fig S29.pdf]

```
                         *        20         *        40         *        60         *        80         *       100         *
Ves_a1Y consens GCCgAAACCGGTTTGGCTCAGTGGATAGAGCGTCGGCCTGCGGACTGAAaGGTCCCaGGTTCGATTCCGGTCAAGGGCATGTACCTgGGTTGCGGGCAC ATCCCCAGTaGGa
Ves_a1Y_11-1  : GCCAAAACCGGTTTGGCTCAGTGGATAGAGCGTCGGCCTGCGGACTGAGGGGTCCCAGGTTCGATTCCGGTCAAGGGCATGTACCTGGGTTGCGGGCAC-CTCCCCGTGGGA
Ves_a1Y_46-1  : GCCAAAACCGGTTTGGCTCAGTGGATAGAGCGTCGGCCTGCGGACTGAAAGGTCCCGGGTTCGATTCCGGTCAAGGGCATGTACCTGGGTTGCGGGCAC-ATCCCCCGTGGGA
Ves_a1Y_187   : GCCGAAACCGGTTTGGCTCAGTGGATAGAGCGTCGGCCTGCGGACTGAAAGGTCCCAGGTTCGATTCCGGTCAAGGGCATGTACCTGGGTTGCGGGCAC-ATCCCCAGTAGGA
Ves_a1Y_115   : GCCGAAACCGGTTTGGCTCAGTGGATAGAGCGTCGGCCTGCGGACTGAAAGGTCCCAGGTTCGATTCCGGTCAAGGGCATGTACCTGGGTTGCGGGCAC-ATCCCCAGTGGGA
Ves_a1Y_41    : GCCGAAACCGGTTTGGCTCAGTGGATAGAGCGTCAGCCTGCGGACTGAAAGGTCCCAGGTTCGATTCCGGTCAAGGGCATGTACCTGGGTTGCGGGCAC-ATCCCCAGTGGGA
Ves_a1Y_46-2  : GCCGAAACCGGTTTGGCTCAGTGGATAGAGCGTCGGCCTGCGGACTGAAAGGTCCCAGGTTCGATTCCGGTCAAGGGCATGTACCTGGGTTGCGGGCAC-ATCCCCAGTGGGG
Ves_a1Y_231   : GCCGAAACCGGTTTGGCTCAGTGGATAGAGCGTCGGCCTGCGGACTGAAAGGTCCCAGGTTCGATTCCGGTCAAGGGCATGTACCTGGGTTGCGGGCAC-ATCCCCAGTGGGA
Ves_a1Y_28    : GCCGAAACCGGTTTGGCTCAGTGGATAGAGCGTCGGCCTGTGGACTGAAAGGTCCCAGGTTCGATTCCGGTCAAGGGCATGTACCTGGGTTGCGGGCAC-ATCCCCAGTGGGA
Ves_a1Y_10-1  : GCCGAAACCGGTTTGGCTCAGTGGATAGAGCGTCGGCCTGCGGACTGAAAGGTCCCAGGTTCGATTCCGGTCAAGGGCATGTACCTGGGTTGCGGGCAC-ATCCCCAGTGGGA
Ves_a1Y_11-2  : GCCGAAACCGGTTTGGCTCAGTGGATAGAGCGTCGGCCTGCGGACTGAAAGGTCCCAGGTTCGATTCCGGTCAAGGGCATGTACCTGGGTTGCGGGCAC-ATCCCCAGTGGGA
Ves_a1Y_40-1  : GCCGAAACCGGTTTGGCTCAGTGGATAGAGCGTCGGCCTGCGGACTGAAAGGTCCCGGGTTCGATTCCGGTCAAGGGCATGTACCTGGGTTGCGGGCAC-ATCCCCAGTGGGA
Ves_a1Y_19    : GCCGAAACCGGTTTGGCTCAGTGGATAGAGCGTCGGCCTGCGGACTGAAAGGTCCCGGGTTCGATTCCGGTCAAGGGCATGTACCTGGGTTGCGGGCAC-ATCCCCAGTAGGA
Ves_a1Y_20-1  : GCCGAAACCGGTTTGGCTCAGTGGATAGAGCGTCGGCCTGCGGACTGAAGGGTCCCGGGTTCGATTCCGGTCAAGGGCATGTACCTGGGTTGCGGGCAC-ATCCCCAGTAGGA
Ves_a1Y_13-1  : GCCGAAACCGGTTTGGCTCAGTGGATAGAGCGTTGGCCTGCGGACTGAAAGGTCCCAGGTTCGATTCCGGTCAAGGGCATGTACCTGGGTTGCGGGCAC-ATCCCCAGTAGGA
Ves_a1Y_33    : GCCGAAACCGGTTTGGCTCAGTGGATAGAGCGTCGGCCTGCGGACTGAAAGGTCCCAGGTTCGATTCTGGTCAAGGGCATGTACCTGGGTTGCGGGCAC-ATCCCCAGTAGGA
Ves_a1Y_56    : GCCGAAACCGGTTTGGCTCAGTGGATAGAGCGTCGGCCTGCGGACTGAAAGGTCCCAGGTTCGATTCCGGTCAAGGGCATGTACCTGGGTTGCGGGCAC-ATCCCCAGTAGGA
Ves_a1Y_11-3  : GCCGAAACCGGTTTGGCTCAGTGGATAGAGCGTCGGCCTGCGGACTGAAAGGTCCCAGGTTCGATTCCGGTCAAGGGCATGTACCTGGGTTGCGGGCAC-ATCCCCAGTAGGA
Ves_a1Y_37    : GCCGAAACCGGTTTGGCTCAGTGGATAGAGCGTCGGCCTGCGGACTGAAAGGTCCCAGGTTCGATTCCGGTCAAGGGCATGTACCTGGGTTGCGGGCAC-ATCCCCAGTAGGA
Ves_a1Y_15-1  : GCCGAAACCAGTTTGGCTCAGTGGATAGAGCGTCGGCCTGCGGACTGAAAGGTCCCAGGTTCGATTCCGGTCAAGGGCATGTACCTGGGTTGCGGGCAC-ATCCCCAGTAGGA
Ves_a1Y_23    : GCCGAAACCGGTTTGGCTCAGTGGATAGAGCGTCGGCCTGCGGACTGAAAGGTCCCAGGTTCGATTCCGGTCAGGGGCATGTACCTGGGTTGCGGGCAC-ATCCCCAGTAGGA
Ves_a1Y_52    : GCCGAAACCGGTTTGGCTCAGTGGATAGAGCGTCGGCCTGCGGACTGAAAGGTCCCAGGTTCGATTCCGGTCAAGGGCATGTACCTGGGTTGTGGGCAC-ATCCCCAGTAGGA
Ves_a1Y_31-1  : GCCGAAACCGGTTTGGCTCAGTGGATAGAGCGTCGGCCTGCGGACTGAAAGGTCCCAGGTTCGATTCCGGTCAAGGGCATGTACCTGGGTTGCGGGCAC-ATCCCCAGTAGGA
Ves_a1Y_25-1  : GCCGAAACCGGTTTGGCTCAGTGGATAGAGCGTCGGCCTGCGGACTGAAAGGTCCCAGGTTCGATTCCGGTCAAGGGCATGTACTTGGGTTGCGGGCAC-ATCCCCAGTAGGA
Ves_a1Y_45    : GCCGAAACCGGTTTGGCTCAGTGGATAGAGCGTCGGCTTGCGGACTGAAAGGTCCCAGGTTCGATTCCGGTCAAGGGCATGTACCTGGGTTGCGGGCAC-ATCCCCAGTAGGA
Ves_a1Y_10-2  : GCCGAAACCGGTTTGGCTCAATGGATAGAGCGTCGGCCTGCGGACTGAAAGGTCCCAGGTTCGATTCCGGTCAAGGGCATGTACCTGGGTTGCGGGCAC-ATCCCCAGTAGGA
Ves_a1Y_20-2  : GCCGAAACCGGTTTGGCTCAGTGGATAGAGCGTCGGCCTGCGGACTGAAAGGTCCCAGGTTCGATTCCGGTCAAGGGCATGTACCTGGGTTGCAGGCAC-ATCCCCAGTAGGA
Ves_a1Y_15-2  : GCCGAAACCGGTTTGGCTCAGTGGATAGAGCATCGGCCTGCGGACTGAAAGGTCCCAGGTTCGATTCCGGTCAAGGGCATGTACCTGGGTTGCAGGCAC-ATCCCCAGTAGGA
Ves_a1Y_17-1  : GCCGAAACCGGTTTGGCTCAGTGGATAGAGCGTCGGCCTGCGGACTGAAAGGTCCCAGGTTCGATTCCGGTCAAGGGCATGTACCTGGGTTGCGGGCAC-ATCCCCAGTAGGA
Ves_a1Y_26-1  : GCCAAAACCGGTTTGGCTCAGTGGATAGAGCGTCGGCCTGCGGACTGAAAGGTCCCAGGTTCGATTCCGGTCAAGGGCATGTACCTGGGTTGCGGGCAC-ATCCCCAGTAGGA
Ves_a1Y_16-1  : GCCGAAACCGGTTTGGCTCAGTGGATAGAGCGTCGGCCTGTGGACTGAAAGGTCCCAGGTTCGATTCCGGTCAAGGGCATGTACCTGGGTTGCGGGCAC-ATCCCCAGTAGGA
Ves_a1Y_13-2  : GCCGAAACCGGTTTGGCTCAGTGGATAGAGCATCGGCCTGCGGACTGAAAGGTCCCAGGTTCGATTCCGGTCAAGGGCATGTACCTGGGTTGCGGGCAC-ATCCCCAGTAGGA
Ves_a1Y_12-1  : GCCGAAACCGGTTTGGCTCAGTGGATAGAGCGTCGGCCTGCGGACTGAAAGGTCCCAGGTTCGATTCCAGTCAAGGGCATGTACCTGGGTTGCGGGCAC-ATCCCCAGTAGGA
Ves_a1Y_13-3  : GCCAAAACCGGTTTGGCTCAGTGGATAGAGCATCGGCCTGCGGACTGAAAGGTCCCAGGTTCGATTCCGGTCAAGGGCATGTACCTGGGTTGCGGGCAC-ATCCCCAGTAGGA
Ves_a1Y_24    : GCCAAAACCGGTTTGGCTCAGTGGATAGAGCGTCGGCCTGCGGACTGAAAGGTCCCAGGTTCGATTCCGGTCAAGGGCATGTACCTGGGTTGCGGGCAC-ATCCCCAGTAGGA
Ves_a1Y_26-2  : GCCGAAACCGGTTTGGCTCAGTGGATAGAGCATCGGCCTGCGGACTGAAGGGTCCCAGGTTCGATTCCGGTCAAGGGCATGTACCTGGGTTGCGGGCAC-ATCCCCAGTAGGA
Ves_a1Y_25-2  : GCCGAAACCGGTTTGGCTCAGTGGATAGAGCGTCGGCCTGCGGACTGAAGGGTCCCAGGTTCGATTCCGGTCAAGGGCATGTACCTGGGTTGCGGGCAC-ATCCCCAGTAGGA
Ves_a1Y_22    : GCCAAAACCGGTTTGGCTCAGTGGATAGAGCGTCGGCCTGCGGACTGAAGGGTCCCAGGTTCGATTCCGGTCAAGGGCATGTACCTGGGTTGCGGGCAC-ATCCCCAGTAGGA
Ves_a1Y_14-8  : GCCAAAACCGGTTTGGCTCAGTGGATAGAGCGTCGGCCTGCGGACTGAAGGGTCCCAGGTTCGATTCCGGTCAAGGGCATGTACCTGGGTTGCGGGCAC-ATCCCCAGTGGGA
Ves_a1Y_25-3  : GCCGAAACCGGTTTGGCTCAGTGGATAGAGCGTCGGCCTGCGGACTGAAGGGTCCCGGGTTCGATTCCGGTCAAGGGCATGTACCTGGGTTGCGGGCAC-ATCCCCAGTGGGA
Ves_a1Y_11-4  : GCCAAAACCGGTTTGGCTCAGTGGATAGAGCGTCGGCCTGCGGACTGAAGGGTCCCAGGTTCGATTCCGGTCAAGGGCATGTACCTGGGTTGCGGGCAC-ATCCCCAGTGGGA
Ves_a1Y_20-3  : GCCGAAACCGGTTTGGCTCAGTGGATAGAGCGTCAGCCTGTGGACTGAAAGGTCCCAGGTTCGATTCCGGTCAAGGGCATGTACCTGGGTTGCGGGCAC-ATCCCCAGTAGGA
Ves_a1Y_10-3  : GCCGAAACCGGTTTGGCTCAGTGGATAGAGCGTCGGCCTGCGGACTGAAAGGTCCCAGGTTCGATTCCGGTCAAGGGCATGTACCTGGGTTGCGGGCAC-ATCCCCAATAGGA
Ves_a1Y_27    : GCCGAAACCGGTTTGGCTCAGTGGATAGAGCGTCGGCCTGCGGACTGAAAGGTCCCAGGTTCGATTCCGGTCAAGGGCATGTACCTGGGTTGCGGGCAC-ATCCCCAGTAGGG
Ves_a1Y_14-2  : GCCGAAACCGGTTTGGCTCAGTGGATAGAGCGTCGGCCTGCGGACTGAAAGGTCCCAGGTTCGATTCCGGTCAAGGGCATGTACCTGGGTTGCGGGCAC-ATCCCCAGTGGGG
Ves_a1Y_15-3  : GCCGAAACCGGTTTGGCTCAGTGGATAGAGCGTCGGCCTGCGGACTGAAAGGTCCCAGGTTCGATTCCGGTCAAGGGCATGTACCTGGGTTTCGGGCAC-ATCCCCAGTAGGA
Ves_a1Y_31-2  : GCCAGAACCGGTTTGGCTCAGTGGATAGAGCGTCGGCCTGCGGACTGAAAGGTCCCAGGTTCGATTCCGGTCAAGGGCATGTACCTGGGTTGCGGGCAC-ATCCCCAGTAGGA
Ves_a1Y_14-3  : GCCGAAACCGGTTTGGCTCAGTGGATAGAGCGTCGGCCTGCGGACTGAAAGGTCCCAGGTTCGATTCCGGTCAAGGGCATGTACCTGGGTTGCGGGCAC-ATCCCCAGTAGGG
Ves_a1Y_42    : GCCGAAACCGGTTTGGCTCAGTGGATAGAGCGTCGGCCTGCGGACTGAAAGGTCCCGGGTTCGATTCCGGTCAAGGGCATGTACCTGGGTTGCGGGCAC-ATCCCCAGTAGGA
Ves_a1Y_13-4  : GCCGAAACCGGTTTGGCTCAGTGGATAGAGCGTCAGCCTGCGGACTGAAAGGTCCCGGGTTCGATTCCGGTCAAGGGCATGTACCTGGGTTGCGGGCAC-ATCCCCAATAGGA
Ves_a1Y_181   : GCCGAAACCGGTTTGGCTCAGTGGATAGAGCGTCGGCCTGCGGACTGAAGGGTCCCAGGTTCGATTCCGGTCAAGGGCATGTACCTGGGTTGCGGGCAC-ATCCCCAGTGGGA
Ves_a1Y_11-5  : GCCGAAACCGGTTTGGCTCAGTGGATAGAGCGTCGGCCTGCGGACTGAAGGGTCCCAGGTTCGATTCCGGTCAAGGGCATGTACCTGGGTTGCGGGCAC-ATCCCCAGTGGGA
Ves_a1Y_16-2  : GCCGGAACCGGTTTGGCTCAGTGGATAGAGCGTCGGCCTGCGGACTGAAAGGTCCCAGGTTCGATTCCGGTCGAGGGCATGTACCTGGGTTGCGGGCAC-ATCCCCAGTAGGA
Ves_a1Y_32    : GCCGAAACCGGTTTGGCTCAGTGGATAGAGCGTCGGCCTGCGGACTCAAGGGTCCCAGGTTCGATTCCGGTCAAGGGCATGTACCTGGGTTGCGGGCAC-ATCCCCAGTAGGA
Ves_a1Y_14-4  : GCCGAAACCGGTTTGGCTCAGTGGATAGAGCGTCGGCCTGCGGACTCAAGGGTCCCGGGTTCGATTCCGGTCAAGGGCATGTACCTGGGTTGCGGGCAC-ATCCCCAGTGGGA
Ves_a1Y_14-5  : GCCGAAACCGGTTTGGCTCAGTGGATAGAGCGTCGGCCTGCGGACTCAAGGGTCCCGGGTTCGATTCCGGTCAAGGGCATGTACCTGGGTTGCGGGCAC-ATCCCCAGTGGGG
Ves_a1Y_30-1  : GCCGAAACTGGTTTGGCTCAGTGGATAGAGCGTCGGCCTGCGGACTGAAAGGTCCCAGGTTCGATTCCGGTCAAGGGCATGTACCTGGGTTGCGGGCAC-ATCCCCAGTAGGA
Ves_a1Y_12-2  : ACCGAAACCGGTTTGGCTCAGTGGATAGAGCGTCGGCCTGCGGACTGAAAGGTCCCAGGTTCGATTCCGGTCAAGGGCATGTACCTGGGTTGCGGGCAC-ATCCCCAGTAGGA
Ves_a1Y_13-5  : GCCAAAACCGGTTTGGCTCAGTGGATAGAGCGTCGGCCTGCGGACTGAAAGGTCCCAGGTTCGATTCCGGTCAAGGGCATGTACCTGGGTTGCGGGCAC-ATCCCCAGTAGGA
Ves_a1Y_11-6  : GCCGAAACCGGTTTGGCTCAGTGGATAGAGCGTCGGCCTGCGGACTGAAGGGTCCCAGGTTCGATTCCGGTCAAGGGCATGTACCTGGGTTGCAGGCAC-ATCCCCAGTAGGA
Ves_a1Y_17-2  : GCCGAAACCGGTTTGGCTCAGTGGATAGAGCGTCGGCCTGCGGACTGAAGGGTCCCAGGTTCGATTCCGGTCAAGGGCATGTACCTGGGTTGCGGGCAC-ATCCCCAGTAGGA
Ves_a1Y_11-7  : GCCGAAACCGGTTTGGCTCAGTGGATAGAGCGTCGGCCTGCGGACTGAAAGGTCCCAGGTTCGATTCCAGTCAAGGGCATGTACCTGGGTTGCGGGCAC-ATCCCCAGTGGGA
Ves_a1Y_15-4  : GCCGAAACCGGTTTGGCTCAGTGGATAGAGCGTCGGCCTGCGGACTGAAAGGTCCCAGGTTCGATTCCGGTCAAGGGCATGTACCTGGGTTGCGGGCAC-ATCCCCAGTGGGA
Ves_a1Y_15-5  : GCCGAAACCGGTTTGGCTCAGTGGATAGAGTGTCGGCCTGCGGACTGAAAGGTCCCAGGTTCGATTCCGGTCAAGGGCATGTACCTGGGTTGCGGGCAC-ATCCCCAGTGGGA
```

```
Ves_a1Y_40-2  : GCCGAAACCGGTTTGGCTCAGTGGATAGAGCGTCGGCCTGCGGACTGAAAGGTCCCAGGTTCGATTCCGGTCAAGGGCATGTACCTTGGTTGCGGGCAT-ATCCCCAGTAGGA
Ves_a1Y_17-3  : GCCGAAACCGGTTTGGCTCAGTGGATAGAGCGTCGGCCTGCGGACTGAAAGGTCCCAGGTTCGATTCCGGTCAAGGGCATGTACCTGGGTTGCGGACAC-ATCCCCAGTGGGA
Ves_a1Y_48    : GCCGAAACCGGTTTGGCTCAGTGGATAGAGCGTCGGCCTGCGGACCGAAAGGTCTCAGGTTCGATTCCGGTCAAGGGCATGTACCTGGGTTGCGGGCAC-ATCCCCAGTGGGA
Ves_a1Y_10-4  : GCCGAAACCGGTTTGGCTCAGTGGATAGAGCGTCGGCCTGCGGACTGAAAGGTCCCAGGTTCGATTCCGGTCAAGGGCATGTACCTGGGTTGCGGGCAC-ATCCCCAGTGGGA
Ves_a1Y_18    : GCCGAAACCGGTTTGGCTCAGTGGATAGAGCGTCGGCCTGCGGACTGAAAGGTCCCAGGTTCGATTCCGGTCAAGGGCATGTACCTGGGTTGCGGGCAC-ATCCCCCGTAGGA
Ves_a1Y_10-5  : GCCGAAACCGGTTTGGCTCAGTGGATAGAGCGTCGGCCTGCGGACTGAAAGGTCCCGGGTTCGATTCCGGTCAAGGGCATGTACGTTGGTTGCGGGCAC-ACCCCCAGTAGGA
Ves_a1Y_17-4  : GCCGAAACCGGTTTGGCTCAGTGGATAGAGCGTCGGCCTGCGGACTGAAGGGTCCCGGGTTCGATTCCGGTCAAGGGCATGTACGTTGGTTGCGGGCAC-ATCCCCAGTGGGA
Ves_a1Y_10-6  : GCCCTAACCGGTTTGGCTCAGTGGATAGAGCGTCAGCCTGCGGACTCAAGGGTCCCAGGTTCGATTCCGGTCAAGGGCATGTACCTTGGTTGCGGGCAC-ATCCCCAGTAGGG
Ves_a1Y_11-8  : GCCCTAACCGGTTTGGCTCAGTGGATAGAGCGTCGGCCTGCGGACTGAAAGGTCCCAGGTTCGATTCCGGTCAAGGGCATGTACCTTGGTTGCGGGCAC-ATCCCCAGTAGGG
Ves_a1        : GCCCTAACCGGTTTGGCTCAGTGGATAGAGCGTCGGCCTGCGGACTGAAGGGTCCCAGGTTCGATTCCGGTCAAGGGCATGTACCTTGGTTGCGGGCAC-ATCCCCAGTRGGG
Ves_a1Y_14-6  : GCCCTAACCGGTTTGGCTCAGTGGATAGAGCGTCGGCCTGCGGACTCAAGGGTCCCAGGTTCGATTCCGGTCAAGGGCATGTACCTTGGTTGCGGGCAC-ATCCCCAGTGGGG
Ves_a1Y_10-7  : TGCCAAACCGGTTTGGCTCAGTGGATAGAGCGTCGGCCTGCGGACTGAAAGGTCCCAGGTTCGATTCCGGTCAAGGGCATGTACCTTGGTTGCGGGCAC-ATCCCCAGTAGGA
Ves_a1Y_11-9  : GCCGAAACCGGTTTGGCTCAGTGGATAGAGCGTCGGCCTGCGGACTGAAAGGTCCCAGGTTCGATTCCGGTCAAGGGCATGTACCTGGGTTGCGGGCAC-AT-CCCAGTAGGA
Ves_a1Y_11-10 : GCCGAAACCGGTTTGGCTCAGTGGATAGAGCGTCGGCCTGCGGACTGAAAGGTCCCAGGTTCGATTCCGGTCAAGGGCATGTACCTGGGTTGCGGGCAC-ATCCCCAGT---A
Ves_a1Y_30-2  : GCCGAAACCGGTTTGGCTCAGTGGATAGAGCGTCGGCCTGCGGACTGAAGGGTCCCAGGTTCGATTCCGGTCAAGGGCATGTACCT-GGTTGCGGGCAC-ATCCCCAGTAGGA
Ves_a1Y_14-7  : GCCGAAACCGGTTTGGCTCAGTGGATAGAGCGTCGGCCTGCGGACTGAAAGGTCCCAGGTTCGATTCCGGTCAAGGGCATGTACCTGGGTTGCGGGCAC-ATCCCCAGTAGGA
Ves_a1Y_35    : GCCGAAACCGGTTTGGCTCAGTGGATAGAGCGTCGGCCTGCGGACTGAAAGGTCCCAGGTTCGATTCCGGTCAAGGGCATGTACCTGGGTTGCGGGCAC-ATCCCCAGTGGGA
Ves_a1Y_17-5  : GCCGAAACCGGTTTGGCTCAGTGGATAGAGCGTCGGCCTGCGGACTGAAAGGTCCCAGGTTCGATTCCGGTCAAGGGCATGTACCTGGGTTGCGGGCAC-ATCCCCAGTAGGA
Ves_a1Y_12-3  : GCCGAAACCGGTTTGGCTCAGTGGATAGAGCGTCGGCCTGCGGACTGAAAGGTCCCAGGTTCGATTCCGGTCAAGGGCATGTACCTGGGTTGTGGGCAC-ATCCCCAGTAGGA
Ves_a2        : GCCCTAGCTGGTTTGGCTCAGTGGATAGAGCATCGGCCTGYGGACTGAAGGGTCCCRGGTTCGATTCYGGTCAAGGGCACATGCCYGGGTTGYGGGCTCGATCCCCAGTAGGG

                    120         *       140         *       160         *       180         *       200         *       220
Ves_a1Y consens GaTGTGCAGGAGGCAGCTGaTCGATGTTTCTCTCTCATC   GATGTTTCTAACTCTCTATCtCTCTCCCTTCCTCTCTGTAAAAAATCAATAAAATATATTTaAAAAAAAAA
Ves_a1Y_11-1  : GATGTGCAGGAGGCGGCTGATCGATGTTTCTCTCCCATC---GATGTTTCTAACTATCTATCTCTCTCCCTTCCTCTCTGTAAAAAATCAATAAAATATATTTAAAAAAAAAA
Ves_a1Y_46-1  : GATGTGCAGGAGGCAGCTGGTCGATGTTTCTCTCCCATC---GATGTTTCTAACTATCTATCTCTCTCCCTTCCTCTCTGTAAAAAATCAATAAAATATATTTAAAAAAAAAA
Ves_a1Y_187   : GATGTGCAGGAGGCAGCTGATCGATGTTTCTCTCTCATC---GATGTTTCTAACTCTCTATCTCTCTCCCTTCCTCTCTGTAAAAAATCAATAAAATATATTTAAAAAAAAAA
Ves_a1Y_115   : GATGTGCAGGAGGCAGCTGATCGATGTTTCTCTCTCATC---GATGTTTCTAACTCTCTATCTCTCTCCCTTCCTCTCTGTAAAAAATCAATAAAATATATTTAAAAAAAAAA
Ves_a1Y_41    : GATGTGCAGGAGGCAGCTGATCGATGTTTCTCTCTCATC---GATGTTTCTAACTCTCTATCTCTCTCCCTTCCTCTCTGTAAAAAATCAATAAAATATATTTAAAAAAAAAA
Ves_a1Y_46-2  : GATGTGCAGGAGGCAGCTGATCGATGTTTCTCTCTCATC---GATGTTTCTAACTCTCTATCTCTCTCCCTTCCTCTCTGTAAAAAATCAATAAAATATATTTAAAAAAAAAA
Ves_a1Y_231   : GATGTGCAGGAGGCAGCTGGTCGATGTTTCTCTCTCATC---GATGTTTCTAACTCTCTATCTCTCTCCCTTCCTCTCTGTAAAAAATCAATAAAATATATTTAAAAAAAAAA
Ves_a1Y_28    : GATGTGCAGGAGGCAGCTGATCGATGTTTCTCTCTCATC---GATGTTTCTAACTCTCTATCTCTCTCCCTTCCTCTCTGTAAAAAATCAATAAAATATATTTAAAAAAAAAA
Ves_a1Y_10-1  : GATGTGCAGGAGGCAGCTGATCGATGTTTCTCTCTCATC---GATGTTTCTAGCTCTCTATCTCTCTCCCTTCCTCTCTGTAAAAAATCAATAAAATATATTTAAAAAAAAAA
Ves_a1Y_11-2  : GGTGTGCAGGAGGCAGCTGATCGATGTTTCTCTCTCATC---GATGTTTCTAACTCTCTATCTCTCTCCCTTCCTCTCTGTAAAAAATCAATAAAATATATTTAAAAAAAAAA
Ves_a1Y_40-1  : GATGTGCAGGAGGCAGCTGATCGATGTTTCTCTCTCATC---GATGTTTCTAACTCTCTATCTCTCTCCCTTCCTCTCTGTAAAAAATCAATAAAATATATTTAAAAAAAAAA
Ves_a1Y_19    : GATGTGCAGGAGGCAGCTGATCGATGTTTCTCTCTCATC---GATGTTTCTAACTCTCTATCTCTCTCCCTTCCTCTCTGTAAAAAATCAATAAAATATATTTAAAAAAAAAA
Ves_a1Y_20-1  : GATGTGCAGGAGGCAGCTGATCGATGTTTCTCTCTCATC---GATGTTTCTAACTCTCTATCTCTCTCCCTTCCTCTCTGTAAAAAATCAATAAAATATATTTAAAAAAAAAA
Ves_a1Y_13-1  : GATGTGCAGGAGGCAGCTGATCGATGTTTCTCTCTCATC---GATGTTTCTAACTCTCTATCTCTCTCCCTTCCTCTCTGTAAAAAATCAATAAAATATATTTAAAAAAAAAA
Ves_a1Y_33    : GATGTGCAGGAGGCAGCTGATCGATGTTTCTCTCTCATC---GATGTTTCTAACTCTCTATCTCTCTCCCTTCCTCTCTGTAAAAAATCAATAAAATATATTTAAAAAAAAAA
Ves_a1Y_56    : GATGTGCAGGAGGCAGCTGATCGATGTTTCTCTCTCATC---AATGTTTCTAACTCTCTATCTCTCTCCCTTCCTCTCTGTAAAAAATCAATAAAATATATTTAAAAAAAAAA
Ves_a1Y_11-3  : GATGTGCAAGAGGCAGCTGATCGATGTTTCTCTCTCATC---GATGTTTCTAACTCTCTATCTCTCTCCCTTCCTCTCTGTAAAAAATCAATAAAATATATTTAAAAAAAAAA
Ves_a1Y_37    : GATGTGCAGGAGGCAGCTGATCGATGTTTCTCTCTCATT---GATGTTTCTAACTCTCTATCTCTCTCCCTTCCTCTCTGTAAAAAATCAATAAAATATATTTAAAAAAAAAA
Ves_a1Y_15-1  : GATGTGCAGGAGGCAGCTGATCGATGTTTCTCTCTCATC---GATGTTTCTAACTCTCTATCTCTCTCCCTTCCTCTCTGTAAAAAATCAATAAAATATATTTAAAAAAAAAA
Ves_a1Y_23    : GATGTGCAGGAGGCAGCTGATCGATGTTTCTCTCTCATC---GATGTTTCTAACTCTCTATCTCTCTCCCTTCCTCTCTGTAAAAAATCAATAAAATATATTTAAAAAAAAAA
Ves_a1Y_52    : GATGTGCAGGAGGCAGCTGATCGATGTTTCTCTCTCATC---GATGTTTCTAACTCTCTATCTCTCTCCCTTCCTCTCTGTAAAAAATCAATAAAATATATTTAAAAAAAAAA
Ves_a1Y_31-1  : GATGTGCAGGAGGCAGCTGATCGATGTTTCTCTCTCATC---GATGTTTCTGACTCTCTATCTCTCTCCCTTCCTCTCTGTAAAAAATCAATAAAATATATTTAAAAAAAAAA
Ves_a1Y_25-1  : GATGTGCAGGAGGCAGCTGATCGATGTTTCTCTCTCATC---GATGTTTCTAACTCTCTATCTCTCTCCCTTCCTCTCTGTAAAAAATCAATAAAATATATTTAAAAAAAAAA
Ves_a1Y_45    : GATGTGCAGGAGGCAGCTGATCGATGTTTCTCTCTCATC---GATGTTTCTAACTCTCTATCTCTCTCCCTTCCTCTCTGTAAAAAATCAATAAAATATATTTAAAAAAAAAA
Ves_a1Y_10-2  : GATGTGCAGGAGGCAGCTGATCGATGTTTCTCTCTCATC---GATGTTTCTAACTCTCTATCTCTCTCCCTTCCTCTCTGTAAAAAATCAATAAAATATATTTAAAAAAAAAA
Ves_a1Y_20-2  : GATGTGCAGGAGGCAGCTGATCGATGTTTCTCTCTCATC---GATGTTTCTAACTCTCTATCTCTCTCCCTTCCTCTCTGTAAAAAATCAATAAAATATATTTAAAAAAAAAA
Ves_a1Y_15-2  : GATGTGCAGGAGGCAGCTGATCGATGTTTCTCTCTCATC---GATGTTTCTAACTCTCTATCTCTCTCCCTTCCTCTCTGTAAAAAATCAATAAAATATATTTAAAAAAAAAA
Ves_a1Y_17-1  : GATGTGCAGGAGGCAGCTGGTCGATGTTTCTCTCTCATC---GATGTTTCTAGCTCTCTATCTCTCTCCCTTCCTCTCTGTAAAAAATCAATAAAATATATTTAAAAAAAAAA
Ves_a1Y_26-1  : GATGTGCAGGAGGCAGCTGGTCGATGTTTCTCTCTCATC---GATGTTTCTAACTCTCTATCTCTCTCCCTTCCTCTCTGTAAAAAATCAATAAAATATATTTAAAAAAAAAA
Ves_a1Y_16-1  : GATGTGCAGGAGGCAGCTGGTCGATGTTTCTCTCTCATC---GATGTTTCTAACTCTCTATCTCTCTCCCTTCCTCTCTGTAAAAAATCAATAAAATATATTTAAAAAAAAAA
Ves_a1Y_13-2  : GATGTGCAGGAGGCAGCTGGTCGATGTTTCTCTCTCATC---GATGTTTCTAACTCTCTATCTCTCTCCCTTCCTCTCTGTAAAAAATCAATAAAATATATTTAAAAAAAAAA
Ves_a1Y_12-1  : GATGTGCAGGAGGCAGCTGGTCGATGTTTCTCTCTCATC---GATGTTTCTAACTCTCTATCTCTCTCCCTTCCTCTCTGTAAAAAATCAATAAAATATATTTAAAAAAAAAA
Ves_a1Y_13-3  : GATGTGCAGGAGGCAGCTGATCGATGTTTCTCTCTCATC---GATGTTTCTAACTCTCTATCTCTCTCCCTTCCTCTCTGTAAAAAATCAATAAAATATATTTAAAAAAAAAA
Ves_a1Y_24    : GATGTGCAGGAGGCAGCTGATCGATGTCTCTCTCTCATC---GATGTTTCTAACTCTCTATCTCTCTCCCTTCCTCTCTGTAAAAAATCAATAAAATATATTTAAAAAAAAAA
Ves_a1Y_26-2  : GATGTGCAGGAGGCAGCTGATCGATGTTTCTCTCTCATC---GATGTTTCTAACTCTCTATCTCTCTCCCTTCCTCTCTGTAAAAAATCAATAAAATATATTTAAAAAAAAAA
Ves_a1Y_25-2  : GATGTGCAGGAGGCGGCTGATCGATGTTTCTCTCTCATC---GATGTTTCTAACTCTCTATCTCTCTCCCTTCCTCTCTGTAAAAAATCAATAAAATATATTTAAAAAAAAAA
Ves_a1Y_22    : GATGTGCAGGAGGCAGCTGATCGATGTTTCTCTCTCATC---GATGTTTCTAACTCTCTATCTCTCTCCCTTCCTCTCTGTAAAAAATCAATAAAATATATTTAAAAAAAAAA
Ves_a1Y_14-8  : GATGTGCAGGAGGCGGCTGATCGATGTTTCTCTCTCATC---GATGTTTCTAACTCTCTATCTCTCTCCCTTCCTCTCTGTAAAAAATCAATAAAATATATTTAAAAAAAAAA
Ves_a1Y_25-3  : GATGTGCAGGAGGCAGCTGGTCGATGTTTCTCTCTCATC---GATGTTTCTAACTCTCTATCTCTCTCCCTTCCTCTCTGTAAAAAATCAATAAAATATATTTAAAAAAAAAA
Ves_a1Y_11-4  : GATGTGCAGGAGGCAGCTGGTCGATGTTTCTCTCTCATC---GATGTTTCTAACTCTCTATCTCTCTCCCTTCCTCTCTGTAAAAAATCAATAAAATATATTTAAAAAAAAAA
Ves_a1Y_20-3  : GATGTGCAGGAGGCAGCTGATCGATGTTTCTCTCTCATC---GATGTTTCTAACTCTCTATCTCTCTCCCTTCCTCTCTGTAAAAAATCAATAAAATATATTTAAAAAAAAAA
Ves_a1Y_10-3  : GATGTGCAGGAGGCAGCTGATCGATGTTTCTCTCTCATC---GATGTTTCTAGCTCTCTATCTCTCTCCCTTCCTCTCTGTAAAAAATCAATAAAATATATTTAAAAAAAAAA
```

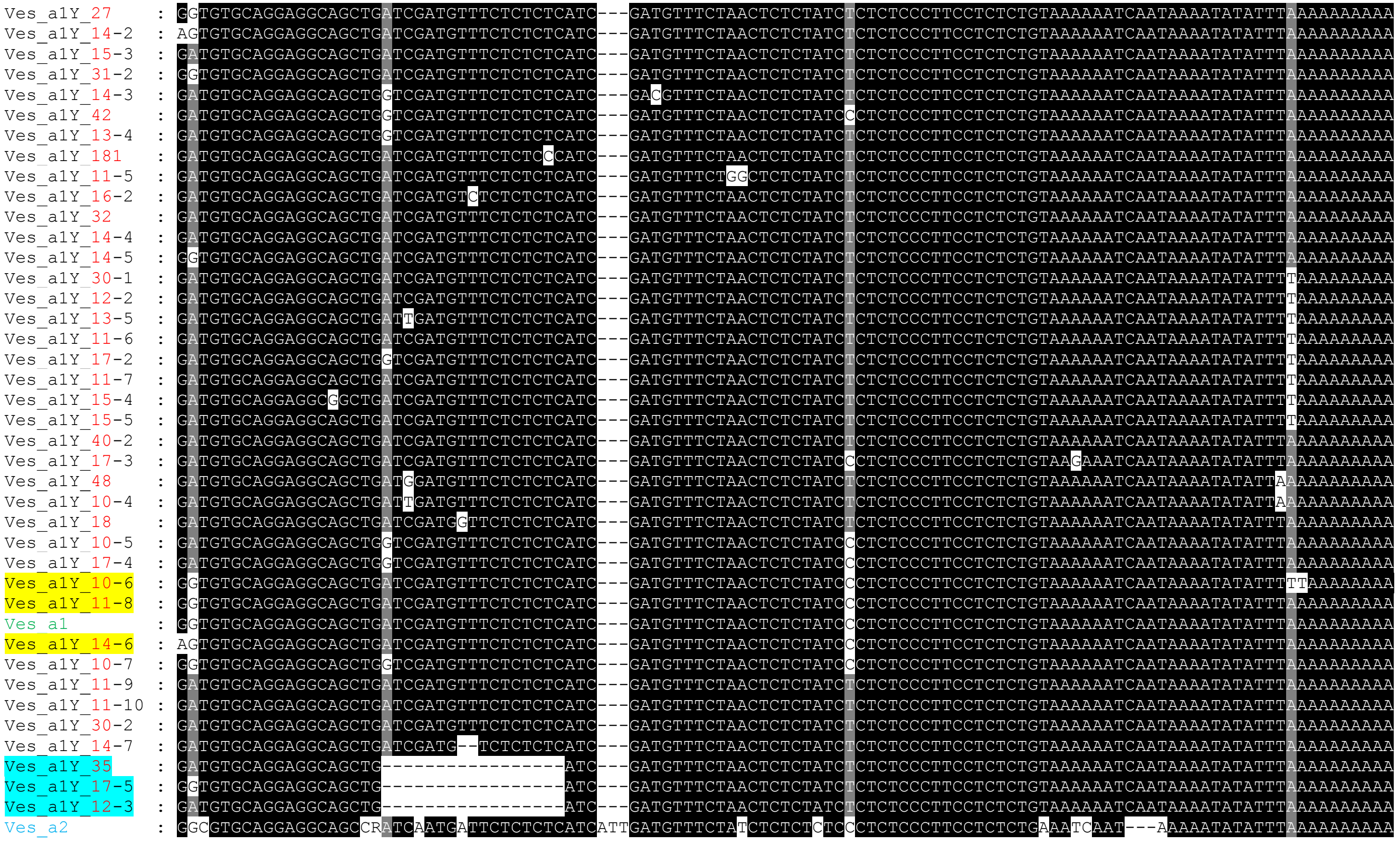

Figure S29. Consensus sequences of 81 tribes of young Ves_a. All copies correspond to Ves_a1. The consensus sequence of the tribes (Ves_a1Y) is shown above. The number of copies in each tribe is indicated in red. The names of six tribes that are less similar are highlighted in yellow and blue. The consensus sequences of Ves_a1 and Ves_a2 subfamilies are also specified.
