# Supplementary material for: Analysis of SINE Families B2, Dip, and Ves with Special Reference to Polyadenylation Signals and Transcription Terminators
Source: Int J Mol Sci. 2021 Sep 13;22(18):9897. doi: 10.3390/ijms22189897 (PMC8466645; doi:10.3390/ijms22189897)
Supplement: Supplementary file 1 [file ijms-22-09897-s001.zip › Supplementary Vassetzky/SINE Ves. Figs S23-S32. Table S9/Fig S31.pdf]

```
Ves_b                             : ---------------------------GCCCGGCCGGTGTGGCTCAGT-GGTTGAG---CATCGACCTATGAACCAGGAGGTCA-CGGTTCGATTCCCGGTCAGGGCACATGCCCGGGTTGCGGGCTCGATCCCCAGTG-GGGGGCGTGCAGGAGGCAGCC
NW005871649.1_243816-244231       : AAAAGGTGCTCAATAAATGCCAATTCTGCCCGGCCGGTGTGGCTCAGT-GGTTGAG---TGTCCACCTATGAACCAGGAGGTCA-CGGTTCGATTCCCGGTCAGGGCACATGCCCAGGTTGCAGGCTCCATCCCCAGTA-GGGGGCGTGCAGGAGGCAGCC
NW005871868.1_4796-5216           : TTCATATTCAAATTTGGGGCACCTTTGCCCGGCC-AGTGTGGCTCAGT-GGTTGGG---TGTCAACCTATGAACCAGGAGGTCA-CGGTACGATTCCCAGTCAGGGCACATGCCCAGGTTGCAGGCTCGATCCCCAGTA-GGGGGCGTGCAGGAGGCAGCC
NW005871939.1_21207-21628         : AAGGCTGTAGCAAAGAACGTACCATGGCCCGGCC-GGTGTGGCTCAGT-GGTTGAG---CGTTGACCTATGAACCAGGAGGTCA-CGGTTCGATTCCCGGTCCGGCACATGCCCGGGTTGCAGGCTCCATCCCCAGTA-GGGGGCGTGCAGGAGGCAGCC
NW005872283.1_84477-84886         : GCATCTCGGTGCTTTAAGATACCTGAGCCCGGCC-GGTGTGGCTCAGT-GGTTGAG---CATCGATCTATGAACCAGGAGGTCA-CGGTTCGATTCCCGGTCAGGGCACATGCCCAGGTTGCGGGCTCCATCCCCAGTA-GGGGGTGTGCAGGAGGCAGCC
NW005871124.1_2324569-2324972     : GTTGCCATTAAAACGATGCCCTAAAAGCCCGGCC-TGTGTGGCTCAGT-GGTTGAG---CGTCGACCTATGAACCAGGAGGCCA-CGGTTCGATTCCCGGTCAGGGCACAGGCCCAGGTTGCGGGCTTCATCCCCAGTA-GGGGGCGTGCAGGAGGCAGCC
NW005872298.1_154206-154628       : AGAACAAGTATTGTAAAGAAAAATTAGCCCAGCCGGGTGTAGCTCATC-GGTTGAG---CGTCGACCCAGGAACCGAGAGGTCACCGGTTCGATTCCCGGTCAGGGCATATGCCCAGGTTGCGGGCTCGATCCCCAGTG-GGGGGCGTGCAGGAGGCAGCC
NW005871082.1_8797663-8798084     : ACAAAGGGGCATGAAAAAGTAGTCTCACCCAGCCGGGTGTAGCTCAGC-AGTAGAG---CGTCGACCTAGGAACCAAGAGGTCACCGGTTCGATTCCCGGTCAGGGCATATGCCCAGGTTGCGAGCTCGATCCCCAGTG-GGGGGCGTGCAGGAGGCAGCC
NW005871197.1_820729-821151       : NNNNNNNNNNNNNNNNNNNNNNNATTGCCCAGCCGGGTGTAGCTCAGC-GGTTGAG---CATCGACCCAGGAACCAAGAGGTCACCGGTTCGATTCCTGGTCAGGGCATATGCCCAGGTTGCGGGCTCGATCCCCAGTG-GAGGTCCTGCAGGAGGCAGCC
NW005871558.1_161777-162199       : AAAAGTGGCGTTAGAAGAAGTGGGGGGCCCAGCCGGGTGTAGCTCAGC-GGTAGAG---CCTCGACCTAGGAACCAAGAGGTCACCAGTTCGATTCCTGGTCAGGGCACATGCCCAGGTTGCGGGCTTGATCCCCAGTG-GGGGGCGTGCAGGAGGCAGCC
NW005871270.1_1325999-1326420     : CAAGAAAAAGAAATAAAAGGCAAACAGCCCAGCCGGGTGTAGCTCAGC-GGTTGAG---CGTCGACCCAGGAACCAAGAGGTCGCCAGTTCGATTCCTGGTCAGGGCATATGCCCAGGTTGCGGGCTCGATCCCCAGTG-GGGGGCATGCAGGAGGCAGCC
NW005872015.1_42007-42429         : CCTCAGGTCTTAAGAAAGGCTTTGATGCCCAGCCGGGTGTAGCTCAGC-GGTTGAG---CGTCGACCCAGGAACCAAGAGGTCGCCGGTTCGATTCCTGGTCAGGGCATATGCCCAGGTTGTGGGCTTGATCCCCAGTG-GGGGGCATGCAGGAGGCAGCC
NW005871071.1_8334330-8334751     : AGTGGTCTTAAAAATTGTTTCAAGTCGCCCAGCCAGGTGTGGCTCAGC-GGTTGAG---CGTCGACCCAGGAACCAAGAGGTCGCCGGTTCGATTCCTGGTCAGGGCATATGCCCAGGTTGCGGGCTCGATCCCCAGTG-GGGGGCATGCAGGAGGCAGCC
NW005872280.1_42794-43216         : GTTTTATTTGAATAAAAAATATATGAGCCCAGCCAGGTGTGGCTCAGC-GGTTGAG---CGTCGACCCAGGAACCAGGAGGTCGCCAGTTCGATTCCTGGTCAGGGCATATGCCCAGGTTGCGGACTCGATCCCCAGTG-GGGGGCATGCAGGAGGCAGCC
NW005871051.1_12123379-1212380    : AAAGGTATTTTAAAAATAGTTTTGTAGCCCAGCCAGGTGTAGCTCAGC-GGTTGAG---CGTCAACCCAGGAACCAAGAGGTCACCAGTTCGATTCCTGGTCAGGGCATATGCCCAGGTTGCGGGCTTGATCCCCAGTG-GGGGGCATGCAGGAGGCAGCC
NW005871114.1_1519274-1519698     : ATGTTGAGTTAAAAAGGCAAGTGGCAGCCCAGCCGGGTGTGGCTCAGC-GGTTGAG---CCTCGACCCACGAACCAAGAGGTCGCTGGTTCGATTCCTGGTCAGGGCATATGCCCAGGTTGTGGGCTCGATCCCCAGTG-GGGGGCATGCAGGAGGCAGCC
NW005871058.1_8825394-8825807     : AATTAGTTTTATCAATAGTAAATTTTTCCCGGCCGGGTGTGGCTCAGC-AGTTGAG---CGTTGACCCAGGAACCAGGAGGCCGCCGGTTCGATTCCTTGTCAGGGCATATGCCCAGGTTGCGGGCTCGATCCCCAGTG-GGGGGCATGCAGGAGGCAGCC
NW005871048.1_14046359-1404678    : GAGGGCTGGATCAAAATGTCATGCTTGCCCAGCCGGGTGTAGCTCAGC-GGTTGAG---CATCGACCCAGGAACCAAGAGGTCGCCGGTTCAATTCCTGGTCAGGGCATATGCCCAGGTTGTGGGCTTGATCCCCAGTG-GGGGGCATGCAGGAGGCAGCC
NW005871486.1_214707-215129       : CATGGGTTAATAAATAAATAGTTGCTGCCCAGCCGGGTGTAGCTCAGC-GGTTGAG---CGTCGACCCAGGAACCAAGAGGTCGAGGGTTTGATTCCTGGTCAGGGCATATGCCCAGGTTGCGGGCTCGATCCCCAGTG-TGGGGCATGCAGGAGGCAACC
NW005871461.1_285126-285545       : TACCCCATCTAAACTTCTGTGCTTTGGCCAAGCCGGGTGTAGCTCAGC-GGTTGAG---CGTCGACCCAGGAACCAAGAGGTCGCCGGTTCGATTCCTGGTCAGGGCATGTGCCCAGGTTGTGGGTTCGATCCCCAGTG-GGGGGCATGCAGGAGGCAGCC
NW005871114.1_4597735-4598153     : CCAACCATAAAATTATTTTCGTTGCTGCCCAGCCGGGTGTAGCTCAGC-AGTTGAG---CGTCGACCCAGGAACCAAGAGGTCGCCGGTTTGATTCCTGGTCAGGGCATATGCCCAGGTTGCGGGCTTGATCCCCAGTG-GGGAGCATGCAGGAGGCAGCC
NW005871067.1_3163046-3163468     : AAATGATTTAAAAGCTCCAAGCAAAAGCCCAGCCGGGTGTAGCTCAGC-GGTTGAG---CGTCGAACCAGGAACCAAGAGGTCGCCGGTTCGATTCCTGGTCAGGGCATATGCCCAGGTTGCGGGCTTGATCCCCAGTG-GAGGGCATGCAGGAGGCAGCC
NW005871086.1_3582561-3582981     : ACATTTCATTTTAAAAAGTTAAACTTGCCCAGCAGGGTGTAGCTCAGA-GGATGAG---CGTCGACCCAGGAACCAAGAGGTCGTGGGTTCGATTCCTGGTCAGGGCATATGCCCAGGTTGTGGGCTTGATCCCCAGTG-GGGGGCATGCGGGAGGCAGCC
NW005871329.1_399265-399687       : CATGGTGACTGTAGTTAATAATTCTTGCCCAGCCGGGTGTAGCTCAGC-GGTTGAG---CGTCGACCCAGGAACCAAGAGGTCGCGGGTTCGATTCTTGGTCAGGGCATATGCCCAAGTTGCGGGCTCAATCCCCAGTG-GGGGGCATGAGGGAGGCAGCC
NW005871110.1_2866830-2867255     : TTAATGGCCACTAAAAATGTATGCTTGCCCAGCCGGGTGTAGCTCAGC-GGTTGAGCGTCGTCGACCCAGGAACCAAGAGGTCGCCGGTTCGATTCCTGGTCAGGGCATATGCCCAGGTTGCGGGCTTGATCCCCAGTG-GGGAGCATGCAGGAGGCAGCC
NW005871207.1_2427652-2428071     : GAGAAGAATAAAAAGATGCGTAATCCGCCCAGCCAGGTGTAGCTCAGC-GGTTGAG---CGTCAACCCAGGAACCAAGAGGTCACTGGTTCGATTCCTGGTCAGGGCATATGCACAGGTTGCGGGCTTGATCCCCAGTG-GGGGGCATGCAGGAGGCAGCC
NW005871169.1_1149881-1150303     : CTTTCAGATTAAAATAAAGCATGCCAACCCAGCCAGGTGTAGCTCAGA-GGTTGAG---CGTCGACCCAGGAACCAAGAGGTCGCCGGTCCGATTCCTGGTCAGGGCATATGCCCAGGTTTTGGGCTTGATCCCCAGTG-GGGGGCATGCAGGAGGCAGCC
NW005871067.1_5341238-5341657     : TGTACAGTCAAGAATTATTAAAATCAGCCCAGCCAGGTGTGGCTCAGT-GGTTGAG---CGTCGACCCAGGAACCAAGAGGTCGCCGGTTCGATTCCTGGTC--GGCATATGCCCAGGTTGCAGGCTCGATCCCCAGTG-GGGGGCATGCAGGAGGCAGCC
NW005880354.1_4369-4792           : CATCCCAAGTAAAGACTGTTTTGTCAGCCCAGCCGGGTGTAGCTCAGT-GGTTGAG---CGTCGACCCAGGAACCAAGAGGTCACCGGTTCGAATCCTGGTCAGGGCATATGCCCAGGTTGTGGGCTCGATCCCCAGTG-GGGGTCATGCAGGAGGCAGCC
NW005871107.1_4795163-4795579     : CAAAAAAGATTTAAAATAATAATAAAGCCCAGCCGGGTGTAACTCAGC-AGTTGAG---CGTCGACCCAGGAACCAAGAGGTTGCCGGTT-GATTCCTGGTCAGGGCATATGCCCAGGTTGCGGGCTCGATCCCCAGTG-GGGGGCGTGCAGGAGGCAGCC
NW005871048.1_31963432-3196385    : TTACCATGCCAACATGAACTTCTTATGCCCAGCCGGGTGTAGCTCAGC-GGTTGAG---CATCTACCCAGGAACCAAGAGGTCGCTGGTTCGATTCCTGGTCAGGGCATATGCCCAGGATGCGGGCAC-ATCCCCAGTG-GGGGGCATGCAGGAGGCAGCC
NW005871636.1_156560-156973       : TACTCAGCCATAAAAAATGAAATCTTGCCCAGCCAGGTGTAGCTCAGC-GGTTGAG---CGTCGACCCAGGAACCAAGAGGTCGCCGGTTCGTTTCCTGGTCAGGGCATATGCCCAGGTTGCGGGCTTGATCCCCAGTG--GGGGCATGCAGGAGGCAGCC
NW005871228.1_2721458-2721881     : GGGACATTTTTAAAGGGACATTAATAGCCGAGCCAGGTGTAGCTCAGT-GGTAGAG---CGTCGACCTAGGAACCAAGAGGTCACTGGTTCGATTCCCGGTCAGGGCATATGCCCAGGTTGTGGGCTTGATCCCCAGTG-GGGGGCGTGCAGGAGGCAGCC
NW005871051.1_11353621-1135404    : GTCAGAATTTTCTAGAAATGTTTGTGGCCCAGCCGGGTGTAGCTCAGC-GGTAGAG---CGTCGACCTAGGAACCAAGAGGTCACCGGTTCGATTCCCAGTCGGGGCATATGCCCAGGTTGCGGGCTTGATCCCCAGTG-GGCGACGTGCAGGAGGCAGCC
NW005871214.1_2312776-2313194     : ATCTCAAAATAAAAAGTTTATTAAAAGCCCAGCCGGGTGTAGCTCAGC-GGTGGAG---CATCGACCTAGGAACCAAGAGGTCACCAGTTCAATTCCCGGTCGGGGCATATGCCCAGGTTGCGGGCTTGATCCCCAGTG-GGGGGCGTGCAGGAGGCAGCC
NW005872061.1_31900-32322         : ATAATAACATTCAAGAATAACAATCTGCCCAGCCGGGTGTAGCTCAGT-GGTAGAG---CATCGACCTAGGAATCAAGAGGTCACCGGTTCGATTCCCAGTCAGGGCATATGCCCAGGTTGCGGGCTTGATCCCCAGTG-GGGGACGTGCAAGAAGCAGCC
NW005871271.1_1085664-1086083     : AAAAGAATAAATAAATCATAAGAATGGCCCAGCCGGGTGTAGCTCAGC-GGTAGAG---GGTTGACCTAGGAACCAAGAGGTCACTGGTTTGATTCCCAGTCAGGGCATATGCCCAGGTTGCGGGCTTGATCCCCAGTG-GGGGGCGTGCAGGAGGCAGCC
NW005871556.1_400591-401007       : AGAATGGCTTCAAAAGTAGGATAATAGCCCAGCCAGGTGTAGCTCAGC-GCTAGAG---CGTCGACCTAGGAACCAAGAGGTCACCAGTTCGATTCCCGGTCAGGGCACATGCCCAGGTTGCGGGCTTGATCCCCAGTG-GGGGGCGTGCAGGAGGCAGCC
NW005871054.1_14577759-1457817    : AACAAGATGATAAAAACCATGGGCTGGCCCAGCCAGGTGTAGCTCAGC-AGTTGAG---CGTCGACCTAGGAACCAAGAGGTCACTGGTTCGATTCCCAGTCAGGGCATATGCCCAGGTTGCGGGCTCGATCCCCAGTGAGGGAGCGTGCAGGAGGCAGCC
NW005871198.1_279261-279677       : ATTTATGTTTTAAAATGATCACTCTAGCCCAGCCGGGTGTAGCTCAGC-GGTAGAG---CGTCGACCTAGGAACCAAGAGGTCACCGGTTCGATTCCCAGTCAGGGCATATGCCCAGGTTGCGGGCTTGATCCCCAGTG-GGGGACGTGCAGGAGGCAGCC
NW005871147.1_3498458-3498876     : ATATAGTATTAAAAAATTGAAAGATAGCCCAGCCGGGTGTAGCTCAGC-GGTAGAG---CGTCGACCTAGGAACCAAGAGGTCACCGGTTCGATTCCCAGTCAGGGCATATGCCCAGGTTGCAGGCTTGATCCCCAGTG-GGGGGCGTGCAGGAGGCAGCC
NW005871048.1_25928226-2592864    : TTGAAGGAATAAAAATAGATAGTGTTGTCCAGCCGGGTGTAGCTCAGC-GGTAGAG---CGTCGACCTAGGAACCAAGAGGTCACCGGTTCGATTCCCAGTCGGGGCATATGCCCAGGTTGCGGGCTTGATCCCCAGTG-GCGGACGTGCAGGAGGCAGCC
NW005871189.1_474665-475087       : AGATGGGGTATAAAGTACAGGTATCTGCCCAGCCGGGTGTAGCTCAGG-GGTAGAG---CGTCGACCTAGGAACCAAGAGGTCACCGGTTCGATTCCCGGTCAGGGCATATACCCAGGTTGCGGGCTTGATCCCCAGTG-GGGGACATGCAAGAGGCAGCC
NW005871057.1_1196371-1196784     : AAAAGGAATATAAAACTGTTTTTGCAGCCCAGCCGGGTGTGGCTCAGC-GGTAGAG---CGTCGAGCTAGGAACCAAGAGGTCACCGGTTCGACTCCCAATCAGGGCATATGCCCAGGTTGCGGGCTTGATCCCCAGTG-GGGGGCGTGCACGAGGCAGCC
NW005871166.1_2433166-2433588     : TGTTGACAGAAAGATAAGAAATTGCAGCCCGGCT-GTGGTGGCTCAGAAGATTGAG---CGTTGTCCTATGGACCAAGAGGTCCTGGGTTCAATTCCCAGTCAGGGCACATGCCTGGGTTGTGGGCTCCATCCCCAGTA-GGGGGTGTGCAGGAGGCAACC
NW005871150.1_1463-1882           : ATGGCCAGTTTAAGAAATTCCCTGAAGCCCAGCT-GTGATGGCTCAGTGGATTGAG---CATCGTCCCATGGACCAAGAGGTCCTGGGTTCGATTCCCAGTCAGGGCACATGCCTGGGTTGCGGGCTCCATCCCCAGTA-TGGGGTGTGCAGGAGGCAACC
NW005871138.1_3298140-3298557     : GGGTAGGATTTCAAGAAACTAGTATCGCCCGGCT-GTGGTGGCTCAGTGGGTTGAG---CATCGCCCCATGGACCAAGAGGTCCTGGGTTCGATTCCCAGTCAGGGCACATGCCTGGGTTGCAGGCTTGATCCCCAGTA--GGGGTGTGCAGGAGGCAACC
NW005871060.1_124157-124569       : ACAAATAATTAGAAAACCTGAGTTCAGCCCGGCT-GTGATGGCTCAGTGGATTGAG---CGTCGTCCCATGGACCAAGAGGTCCTGGGTTCGATTCCCAGTCAGGGCACATGCCTGGGTTGCAGGCTCCATCCCCAGTA-GGGGGTGTGCAGGAGGCAACC
NW005871250.1_293148-293565       : TTTACATTGTCTTAAAAATATCTACAGCCCAGCT-GTGATGGCTCAGTGGATTGAG---CATCGTCCCATGGACCAAGAGGTCCTGGGTTCGATTCCCAGTTAGGGCACATGCCTGGGTTGCGGGCTCCATCCCCAGTA-GGGGGTGTGCAGGAGGCAACC
NW005871054.1_9498323-9498742     : CTTACTAGTTATAAAGACTTTGGTAAGCCCGGCT-GTGGTGGCTCAGTGGGTTGAG---CATCGCCCCATGGACCAAGAGGTCCTGAGTTCGATTCCCAGTCAGGGCACATGCCTGGGTTGCAGGCTTGATCCCCAGTA--GGGGTGTGCAGGAGGCAACC
NW005871124.1_1790518-1790939     : AATAAGTCAGTAAAAAAAAATACCGAGCCCAGCT-GTGGTGGCTCAGTGGGTTGAG---CATCGTCCCATGAACCAAGAGGTCCTGGGTTCGATTCCCAGTCAGGGCACATCCCTGGGTTGCAGGCTTGATCCCCAGTA--GGGGTGTGCAGGAGGCAACC
NW005871147.1_2088418-2088839     : AAGGTATCCTTTAAGAAGAGGAAGAGGCCCGGCT-GTGGTGGCTCAGTGGGTTGAG---CGTTGCCCCATGGACCAAGAGGTCCTGGATTCAATTCCCAGTCAGGGCACATGCCTGGGTTGCAGGCTTGATCCCCAGTA--GGGGCGTGCAGGAGGCAACC
NW005871413.1_381951-382368       : TCTGATATCAATAAAAATATACTTTAGCCCGGCT-GTGGTGGCTCAGTGGGTTGAG---CATCGCCCCATGGACCAAGAGGTCCTGGGTTCGATTCCCAGTCAGGGCACATACCTGGGTTGCAGGCTTGATCCCCAGTA--GGGGTGTGCAGGAGGCAACC
NW005871048.1_13323993-1332441    : TGGAACTCCTTTAAAAGTACTTCGGAGCCCGGCT-CTGGTGGCTCAGTGGGTTGAG---CGTTGCCCCATGGACCAAGAGGTCCTGGGTTCGATTCCCAGTAAGGGCACATGCCTGGGTTGCAGGCTTGATCCCCAGTA--GGGGCGTGCAGGAGGCAACC
NW005872230.1_44956-45374         : ACAAAAAGTAAATAAAAAAGGTTAATGCCCGTCT-GTGGTGGCTCAGAAGGTTGAG---CGTCGCCCCATGGACCAAGAGGTCCTGGGTTCGATTCCCAGTCAGGGCACATGCCTGGGTTGCAGGCTTGATCCCCAGTA--GGGGCGTGCAGGAGGCAACC
```

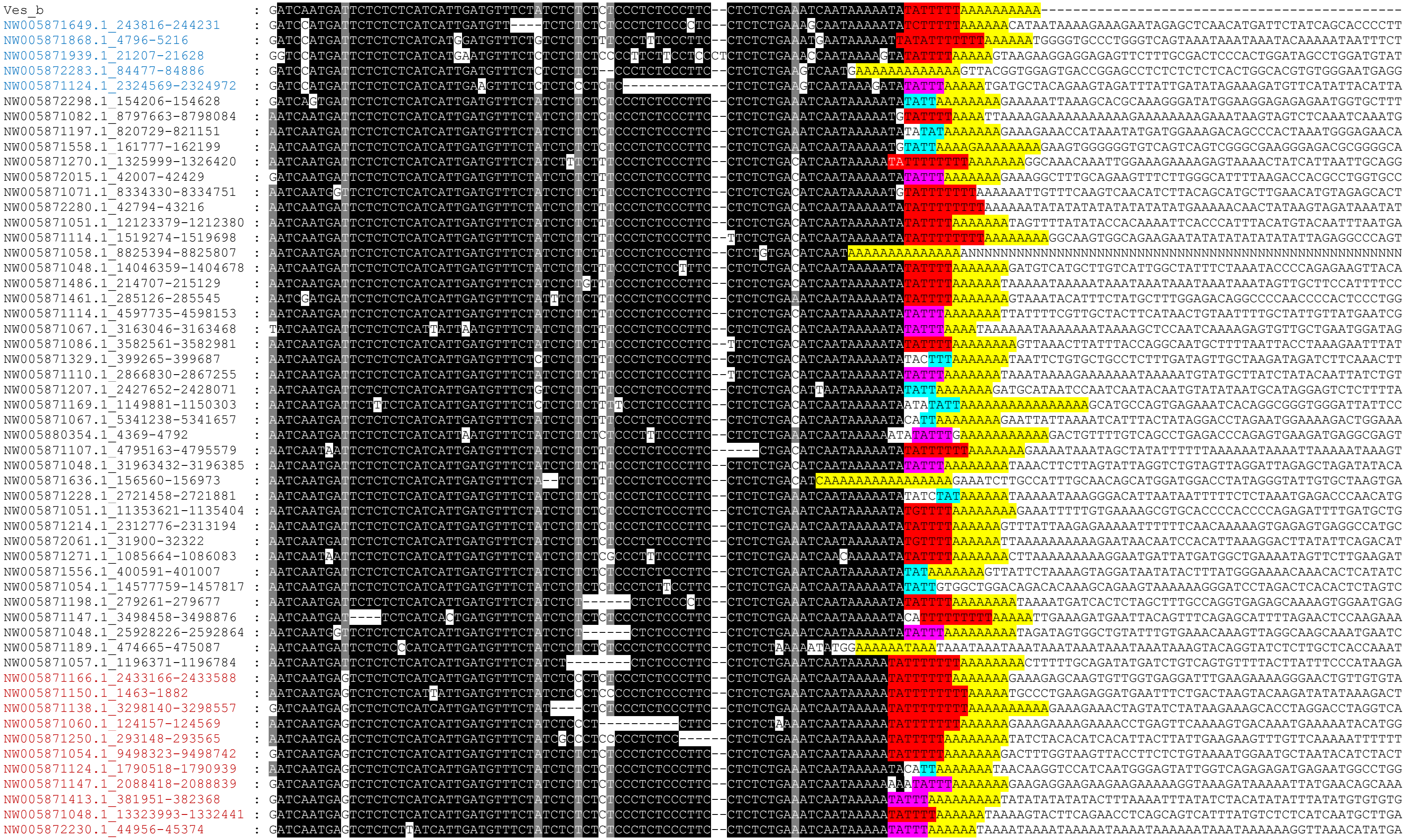

Figure S31. Three relatively young Ves_b tribes in *M. lucifugus* genome. The names of copies of the three tribes are shown in blue, black, or red. The nucleotide sequences are highlighted as follows: strong pol III terminators, medium terminators, rudimentary terminators, and oligo(A).
