# Supplementary material for: Analysis of SINE Families B2, Dip, and Ves with Special Reference to Polyadenylation Signals and Transcription Terminators
Source: Int J Mol Sci. 2021 Sep 13;22(18):9897. doi: 10.3390/ijms22189897 (PMC8466645; doi:10.3390/ijms22189897)
Supplement: Supplementary file 1 [file ijms-22-09897-s001.zip › Supplementary Vassetzky/SINE Ves. Figs S23-S32. Table S9/Fig S32.pdf]

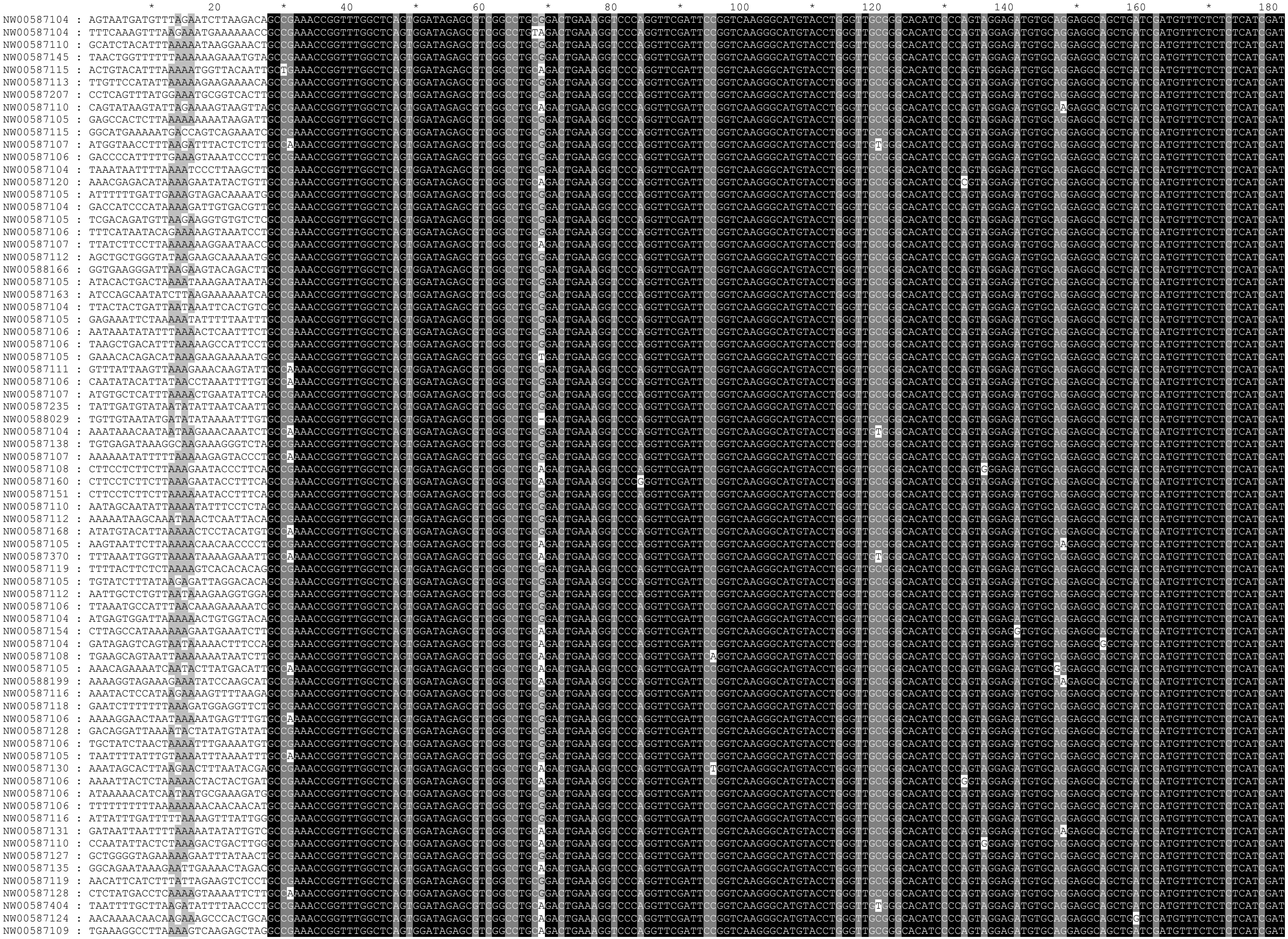

20
40
60
80
100
120
140
160
180

```
NW00587135 : ACTTAAGTATTTAATAACAAGTTTTAGGCCGAAACCGGTTTGGCTCAGTGGATAGAGCGTCGGCCTGCGGACTGAAAGGTCCCAGGTTCGATTCCGGTCAAGGGCATGTACCTGGGTTGCGGGCACATCCCCAGTAGGAGATGTGCAGGAGGCAGCTGATCGATGTTTCTCTCTCATCGAT
NW00587105 : TGAAAGGATTAAGAAGTATTAATCGTTGCCGAAACCGGTTTGGCTCAGTGGATAGAGCGTCGGCCTGCGGACTGAAAGGTCCCAGGTTCGATTCCGGTCAAGGGCATGTACCTGGGTTGCGGGCACATCCCCAGTAGGAGATGTGCAGGAGGCAGCTGATCGATGTTTCTCTCTCATCGAT
NW00587107 : GGATTGGGATTAAGAAATACAGATTGGGCCGAAACCGGTTTGGCTCAGTGGATAGAGCGTCGGCCTGCAGACTGAAAGGTCCCAGGTTCGATTCCGGTCAAGGGCATGTACCTGGGTTGCGGGCACATCCCCAGTAGGAGATGTGCAGGAGGCAGCTGATCGATGTTTCTCTCTCATTGAT
NW00587105 : ATGGAATTATTCAAGAACAAGACTTCAGCCGAAACCGGTTTGGCTCAGTGGATAGAGCGTCGGCCTGCGGACTGAAAGGTCCCAGGTTCGATTCCGGTCAAGGGCATGTACCTGGGTTGCGGGCACATCCCCAGTAGGAGATGTGCAGGAGGCAGCTGATCGATGTTTCTCTCTCATCGAT
NW00587105 : TTGCTTCACTTAAATTTTATTTTTCCAGCCGAAACCGGTTTGGCTCAGTGGATAGAGCGTCGGCCTGCGGACTGAAAGGTCCCAGGTTCGATTCCGGTCAAGGGCATGTACCTGGGTTGCGGGCACATCCCCAGTAGGAGATGTGCAGGAGGCAGCTGATCGATGTTTCTCTCTCATCGAT
NW00587107 : TGTTTCTTTTTAAAATTTTTTTATTGAGCCGAAACCGGTTTGGCTCAGTGGATAGAGCGTCGGCCTGCAGACTGAAAGGTCCCAGGTTCGATTCCGGTCAAGGGCATGTACCTGGGTTGCGGGCACATCCCCAGTAGGAGATGTGCAAGAGGCAGCTGATCGATGTTTCTCTCTCATCGAT
NW00587143 : TTCTTCTTTCTCAAGATTGCTGAAGCTGCCGAAACCGGTTTGGCTCAGTGGATAGAGCGTCGGCCTGCGGACTGAAAGGTCCCAGGTTCGATTCCGGTCAAGGGCATGTACCTGGGTTGCGGGCACATCCCCAGTAGGAGATGTGCAGGAGGCAGCTGATCGATGTTTCTCTCTCATCGAT
NW00587147 : AATTTCTCATCAAAAATTGTTATTTGGGCCGAAACCGGTTTGGCTCAGTGGATAGAGCGTCGGCCTGCGGACTGAAAGGTCCCAGGTTCGATTCCGGTCAAGGGCATGTACCTGGGTTGCGGGCACATCCCCAGTAGGAGATGTGCAGGAGGCAGCTGATCGATGTTTCTCTCTCATCGAT
NW00587109 : CTTCCTCTATGGAAAAATGTATATTCAGCCGAAACCGGTTTGGCTCAGTGGATAGAGCGTCGGCCTGCGGACTGAAAGGTCCCAGGTTCGATTCCGGTCAAGGGCATGTACCTGGGTTGCGGGCACATCCCCAGTAGGAGATGTGCAGGAGGCAGCTGATCGATGTTTCTCTCTCATCGAT
NW00587127 : CCCTTTTATGTAAAAGTATTTTAACAAGCCGAAACCGGTTTGGCTCAGTGGATAGAGCGTCGGCCTGCGGACTGAAAGGTCCCAGGTTCGATTCCGGTCAAGGGCATGTACCTGGGTTGCGGGCACATCCCCAGTAGGAGATGTGCAGGAGGCAGCTGATCGATGTTTCTCTCTCATCGAT
NW00587111 : TTTCTTTGCATAAAAGTTTTGCTATTCGCCGAAACCGGTTTGGCTCAGTGGATAGAGCGTCGGCCTGCGGACTGAAAGGTCCCAGGTTCGATTCCGGTCAAGGGCATGTACCTGGGTTGCGGGCACATCCCCAGTAGGAGATGTGCAGGAGGCAGCTGATCGATGTTTCTCTCTCATCGAT
NW00587122 : TATTTCATTAATAATTTTTTCTAGTTTGCCGAAACCGGTTTGGCTCAGTGGATAGAGCGTCGGCCTGCGGACTGAAAGGTCCCAGGTTCGATTCCGGTCAAGGGCATGTACCTGGGTTGCGGGCACATCCCCAGTAGGAGATGTGCAGGAGGCAGCTGATCGATGTTTCTCTCTCATCGAT
NW00587105 : ACAATTTTCAAGTTAAAAAACTCCCCCGCCAAAACCGGTTTGGCTCAGTGGATAGAGCGTCGGCCTGCGGACTGAAAGGTCCCAGGTTCGATTCCGGTCAAGGGCATGTACCTGGGTTGCGGGCACATCCCCAGTAGGAGATGTGCAGGAGGCAGCTGATCGATGTTTCTCTCTCATCGAT
NW00587105 : AGGTACAAAATATCCTGTAAGATTGTCGCCGAAACCGGTTTGGCTCAGTGGATAGAGCGTCGGCCTGCGGACTGAAAGGTCCCAGGTTCGATTCCGGTCAAGGGCATGTACCTGGGTTGCGGGCACATCCCCAGTAGGAGATGTGCAGGAGGCAGCTGATCGATGTTTCTCTCTCATCGAT
NW00587119 : GTTTCTCTAATAAAAGAATATGTGTGTGCCAAAACCGGTTTGGCTCAGTGGATAGAGCGTCGGCCTGCGGACTGAAAGGTCCCAGGTTCGATTCCGGTCAAGGGCATGTACCTGGGTTGCGGGCACATCCCCAGTAGGAGATGTGCAGGAGGCAGCTGATCGATGTTTCTCTCTCATCGAT
NW00587175 : TCTTGCATTAGATTGAATGATTAGATTGCCGAAACCGGTTTGGCTCAGTGGATAGAGCGTCGGCCTGCGGACTGAAAGGTCCCAGGTTCGATTCCGGTCAAGGGCATGTACCTGGGTTGCGGGCACATCCCCAGTAGGAGATGTGCAGGAGGCAGCTGATCGATGTTTCTCTCTCATCGAT
NW00587118 : ATGACTTATAAATAAATATATTCTTGTGCCAAAACCGGTTTGGCTCAGTGGATAGAGCGTTGGCCTGCAGACTGAAAGGTCCCAGGTTCGATTCCGGTCAAGGGCATGTACCTGGGTTGCGGGCACATCCCCAGTAGGAGATGTGCAGGAGGCAGCTGATCGATGTTTCTCTCTCATCGAT
NW00587162 : CAGGAGATACAGGTAAGAATTAGGTGGGCCAAAACCGGTTTGGCTCAGTGGATAGAGCGTCGGCCTGCGGACTGAAAGGTCCCAGGTTCGATTCCGGTCAAGGGCATGTACCTGGGTTGCGGGCACATCCCCAGTAGGAGATGTGCAGGAGGCAGCTGATCGATGTTTCTCTCTCATCGAT
NW00587107 : AGAGGTCTTTTTAAAAACAAAGGTCTTGCCGAAACCGGTTTGGCTCAGTGGATAGAGCGTCGGCCTGCAGACTGAAAGGCCCCAGGTTCGATTCCGGTCAAGGGCATGTACCTGGGTTGCGGGCACATCCCCAGTAGGAGATGTGCAGGAGGCAGCTGATCGATGTTTCTCTCTCATCGAT
NW00587105 : ACCACAATTAAAATATTTTTAAAAATTGCCGAAACCGGTTTGGCTCAGTGGATAGAGCGTCGGCCTGCAGACTGAAAGGTCCCAGGTTCGATTCCGGTCAAGGGCATGTACCTGGGTTGCGGGCACATCCCCAGTAGGAGATGTGCAGGAGGCAGCTGATCGATGTTTCTCTCTCATCGAT
NW00587117 : ACACTAATGAATAAGAACTATATACTTGCCGAAACCGGTTTGGCTCAGTGGATAGAGCGTCGGCCTGCGGACTGAAAGGTCCCAGGTTCGATTCCGGTCAAGGGCATGTACCTGGGTTGCGGGCACATCCCCAGTAGGAGATGTGCAGGAGGCAGCTGATCGATGTTTCTCTCTCATCGAT
NW00587105 : AATAGTTTTTTTAAAAAATATTAACTAGCCGAAACCGGTTTGGCTCAGTGGATAGAGCGTCGGCCTGCGGACTGAAAGGTCCCAGGTTCGATTCCGGTCAAGGGCATGTACCTGGGTTGCGGGCACATCCCCAGTAGGAGATGTGCAGGAGGCAGCTGATCGATGTTTCTCTCTCATCGAT
NW00587118 : AACTTTATCAATAATATCTAAAATATTGCCGAAACCGGTTTGGCTCAGTGGATAGAGCGTCGGCCTGCAGACTGAAAGGTCCCAGGTTCGATTCCGGTCAAGGGCATGTACCTGGGTTGCGGGCACATCCCCAGTGGGAGATGTGCAGGAGGCAGCTGATCGATGTTTCTCTCTCATCGAT
NW00587105 : ATAATGTCTTTCAAAATTTTAATCTGCGCCGAAACCGGTTTGGCTCAGTGGATAGAGCGTCGGCCTGCGGACTGAAAGGTCCCAGGTTCGATTCCGGTCAAGGGCATGTACCTGGGTTGCGGGCACATCCCCAGTAGGAGATGTGCAGGAGGCAGCTGATCGATGTTTCTCTCTCATCGAT
NW00587132 : TTATATTTTTTAAAAATGAACATAGTAGCCGAAACCGGTTTGGCTCAGTGGATAGAGCGTCGGCCTGCGGACTGAAAGGTCCCAGGTTCGATTCCGGTCAAGGGCATGTACCTGGGTTGCGGGCACATCCCCAGTAGGAGATGTGCAGGAGGCAGCTGATCGATGTTTCTCTCTCATCGAT
NW00587113 : TTTTATTTCTTAAAGTATTACAAAGATGCCAAAACCGGTTTGGCTCAGTGGATAGAGCGTCGGCCTGCGGACTGAAAGGTCCCAGGTTCGATTCCGGTCAAGGGCATGTACCTGGGTTGCGGGCACATCCCCAGTAGGAGATGTGCAGGAGGCAGCTGATCGATGTTTCTCTCTCATCGAT
NW00587119 : AACATCTTTTTAAAAAATTTAAATTCCGCCAAAACCGGTTTGGCTCAGTGGATAGAGCGTCGGCCTGCAGACTGAAAGGTCCCAGGTTCGATTCCGGTCAAGGGCATGTACCTGGGTTGTGGGCACATCCCCAGTAGGAGATGTGCAGGAGGCAGCTGATCGATGTTTCTCTCTCATCGAT
NW00587112 : ATGTTTATGGTTAAAATGATGGTGATGGCCGAAACCGGTTTGGCTCAGTGGATAGAGCGTCGGCCTGCAGACTGAAAGGTCCCAGGTTCGATTCCGGTCAAGGGCATGTACCTGGGTTGCGGGCACATCCCCAGTGGGAGATGTGCAGGAGGCAGCTGATCGATGTTTCTCTCTCATCGAT
NW00587105 : AGTATTTAATAAAAAAAAAAAAAATGTGCCGAAACCGGTTTGGCTCAGTGGATAGAGCGTCGGCCTGCGGACTGAAAGGTCCCAGGTTCGATTCCGGTCAAGGGCATGTACCTGGGTTGCGGGCACATCCCCAGTAGGAGATGTGCAGGAGGCAGCTGATCGATGTTTCTCTCTCATCGAT
NW00587106 : AAGTGGAAATAAAATGATTTTAAATATGCCGAAACCGGTTTGGCTCAGTGGATAGAGCGTCGGCCTGCGGACTGAAAGGTCCCAGGTTCGATTCCGGTCAAGGGCATGTACCTGGGTTGCGGGCACATCCCCAGTAGGAGATGTGCAGGAGGCAGCTGATCGATGTTTCTCTCTCATCGAT
NW00587122 : TTTGTAATCAAACATCAAGAGAAATATGCCGAAACCGGTTTGGCTCAGTGGATAGAGCGTCGGCCTGCGGACTGAAAGGTCCCAGGTTCGATTCCGGTCAAGGGCATGTACCTGGGTTGCGGGCACATCCCCAGTAGGAGATGTGCAGGAGGCAGCTGATCGATGTTTCTCTCTCATCGAT
NW00587120 : AAGGCACCGTTAAATTTTGTCTACGCTGCCGAAACCGGTTTGGCTCAGTGGATAGAGCGTCGGCCTGCGGACTGAAAGGTCCCAGGTTCGATTCCGGTCAAGGGCATGTACCTGGGTTGCGGGCACATCCCCAGTAGGAGATGTGCAGGAGGCAGCTGATCGATGTTTCTCTCTCATCGAT
NW00587184 : AAGACTCCATCAAAAAACTACTAGATTGCCGAAACCGGTTTGGCTCAGTGGATAGAGCGTCGGCCTGCGGACTGAAAGGTCCCAGGTTCGATTCCGGTCAAGGGCATGTACCTGGGTTGCGGGCACATCCCCAGTAGGAGATGTGCAGGAGGCAGCTGATCGATGTTTCTCTCTCATCGAT
NW00587105 : GTCTTATAAATAAATGATAAATGTCTTGCCAAAACCGGTTTGGCTCAGTGGATAGAGCGTCGGCCTGCAGACTGAAAGGTCCCAGGTTCGATTCCGGTCAAGGGCATGTACCTGGGTTGCGGGCACATCCCCAGTAGGAGATGTGCAGGAGGCAGCTGATCGATGTTTCTCTCTCATCGAT
NW00587136 : TGAGGAATGGTCAAATATAAGAGTAGGGCCGAAACCGGTTTGGCTCAGTGGATAGAGCGTCGGCCTGCAGACTGAAAGGTCCCAGGTTCGATTCCGGTCAAGGGCATGTACCTGGGTTGCGGGCACATCCCCAGTAGGAGATGTGCAGGAGGCAGCTGATCAATGTTTCTCTCTCATCGAT
NW00587119 : AAGAAATAACATCCATAGAAAATCATGGCCGAAACCGGTTTGGCTCAGTGGATAGAGCGTCGGCTTGCAGACTGAAAGGTCCCAGGTTCGATTCCGGTCAAGGGCATGTACCTGGGTTGCGGGCACATCCCCAGTAGGAGATGTGCAGGAGGCAGCTGATCGATGTTTCTCTCTCATCGAT
NW00587106 : AAAAAGTTTAATAAAAAACAAAATTCAGCCGAAACCGGTTTGGCTCAGTGGATAGAGCGTCGGCCTGCGGACTGAAAGGTCCCAGGTTCGATTCCGGTCAAGGGCATGTACCTGGGTTGCGGGCACATCCCCAGTAGGAGATGTGCAGGAGGCAGCTGATCGATGTTTCTCTCTCATCGAT
NW00587109 : GTGAAGTGAAATAAAATTTTGTTTAAGGCCGAAACCGGTTTGGCTCAGTGGATAGAGCGTCGGCCTGCGGACTGAAAGGTCCCAGGTTCGATTCCGGTCAAGGGCATGTACCTGGGTTGCGGGCACATCCCCAGTAGGAGATGTGCAGGAGGCAGCTGATCGATGTTTCTCTCTCATCGAT
NW00587114 : GCCTCTTGACCATCAGAGAAACAACCTGCCAAAACCGGTTTGGCTCAGTGGATAGAGCGTCGGCCTGCGGACTGAAAGGTCCCAGGTTCGATTCCGGTCAAGGGCATGTACCTGGGTTGCGGGCACATCCCCAGTAGGAGATGTGCAGGAGGCAGCTGATCGATGTTTCTCTCTCATCGAT
NW00587115 : CTAATCTGCTTTCTAAAAGAAAAATGAGCCAAAACCGGTTTGGCTCAGTGGATAGAGCGTCGGCCTGCAGACTGAAAGGTCCCAGGTTCGATTCCGGTCAAGGGCATGTACCTGGGTTGCGGGCACATCCCCAGTAGGAGATGTGCAGGAGGCAGCTGATCGATGTTTCTCTTTCATCGAT
NW00587107 : TTGATATAAAAGACTATCCTTATTTTCGCCGAAACCGGTTTGGCTCAGTGGATAGAGCGTCGGCCTGCGGACTGAAAGGTCCCAGGTTCGATTCCGGTCAAGGGCATGTACCTGGGTTGCGGGCACATCCCCAGTAGGAGATGTGCAGGAGGCAGCTGATCGATGTTTCTCTCTCATCGAT
NW00587639 : GGGGCTTAGCATAAATGCCTGTGTTTTGCCGAAACCGGTTTGGCTCAGTGGATAGAGCGTCGGCCTGCGGACTGAAAGGTCCCAGGTTCGATTCCGGTCAAGGGCATGTACCTGGGTTGCGGGCACATCCCCAGTAGGAGATGTGCAGGAGGCAGCTGATCGATGTTTCTCTCTCATCGAT
NW00587153 : TAGGTAGATTTATAAAATATGATTGCAGCCGAAACCGGTTTGGCTCAGTGGATAGAGCGTCGGCCTGCGGACTGAAAGGTCCCAGGTTCGATTCCGGTCAAGGGCATGTACCTGGGTTGCGGGCACATCCCCAGTAGGAGATGTGCAGGAGGCAGCTGATCGATGTTTCTCTCTCATCGAT
NW00587105 : ACCAGCTGAAGTAAAAAAATAGTAATAGCCGAAACCGGTTTGGCTCAGTGGATAGAGCGTCGGCCTGCAGACTGAAAGGTCCCAGGTTCGATTCCGGTCAAGGGCATGTACCTGGGTTGCGGGCACATCCCCAGTAGGAGATGTGCAGGAGGCAGCTGATCGATGTTTCTCTCTCATCGAT
NW00587107 : AACAATAGCAACATAGAAGGTATACTTGCCGAAACCGGTTTGGCTCAGTGGATAGAGCGTCGGCCTGCAGACTGAAAGGTCCCAGGTTCGATTCCGGTCAAGGGCATGTACCTGGGTTGCGGGCACATCCCCAGTAGGAGATGTGCAAGAGGCAGCTGATCGATGTTTCTCTCTCATCGAT
NW00587107 : CTCAAAAAAATTAAAAATAGAATTACCGCCGAAACCGGTTTGGCTCAGTGGATAGAGCGTCGGCCTGCAGACTGAAAGGTCCCAGGTTCGATTCCGGTCAAGGGCATGTACCTGGGTTGCGGGCACATCCCCAGTGGGAGATGTGCAGGAGGCAGCTGATCGATGTTTCTCTCTCATCGAT
NW00587110 : CTCAAAAACATTAAAAATAGAACTATCGCCGAAACCGGTTTGGCTCAGTGGATAGAGCGTCGGCCTGCAGACTGAAGGGTCCCAGGTTCGATTCCGGTCAAGGGCATGTACCTGGGTTGCGGGCACATCCCCAGTAGGAGATGTGCAGGAGGCAGCTGATCGATGTTTCTCTCTCATCGAT
NW00587891 : CTGAAAGTAACCTTAAAGGGAGGATGTGCCAAAACCGGTTTGGCTCAGTGGATAGAGCATCGGCCTGCAGACTGAAAGGTCCCAGGTTCGATTCCGGTCAAGGGCATGTACCTGGGTTGCGGGCACATCCCCAGTAGGAGATGTGCAGGAGGCAGCTGATCGATGTTTCTCTCTCATCGAT
NW00587173 : TGAAGTAGCAACGAAAATAATTTTATGGCCGAAACCGGTTTGGCTCAGTGGATAGAGCGTCGGCCTGCAGACTGAAAGGTCCCAGGTTCGATTCCGGTCAAGGGCATGTACCTGGGTTGCGGGCACATCCCCCGTAGGAGATGTGCAGGAGGCAGCTGATCGATGTTTCTCTCTCATCGAT
NW00587113 : AAAAAAGTAGTTAATTAAGAATCACTTGCCGAAACCGGTTTGGCTCAGTGGATAGAGCGTCGGCCTGCGGACTGAAAGGTCCCAGGTTCGATTCCGGTCAAGGGCATGTACCTGGGTTGCGGGCACATCCCCAGTAGGAGATGTGCAGGAGGCAGCTGATCGATGTTTCTCTCTCATCGAT
NW00587106 : AAAATTGCATTTTATCAAGATTTTATTGCCGAAACCGGTTTGGCTCAGTGGATAGAGCGTCGGCCTGCGGACTGAAAGGTCCCAGGTTCGATTCCGGTCAAGGGCATGTACCTGGGTTGCGGGCACATCCCCAGTAGGAGATGTGCAGGAGGCAGCTGATCGATGTTTCTCTCTCATCGAT
NW00587147 : TGAATACAGTTTTATTTTAAAGCATACGCCAAAACCGGTTTGGCTCAGTGGATAGAGCGTCGGCCTGCGGACTGAAAGGTCCCAGGTTCGATTCCGGTCAAGGGCATGTACCTGGGTTGCGGGCACATCCCCAGTAGGAGATGTGCAGGAGGCAGCTGATCGATGTTTCTCTCTCATCGAT
NW00587131 : GGGGATGGAGTAAGTCAGTAACTGTGTGCCGAAACCGGTTTGGCTCAGTGGATAGAGCGTCGGCCTGCGGACTGAAAGGTCCCAGGTTCGATTCCGGTCAAGGGCATGTACCTGGGTTGCGGGCACATCCCCAGTAGGAGATGTGCAGGAGGCAGCTGATCGATGTTTCTCTCTCATCGAT
NW00587110 : CCAGTGAATGCATAAATAAGTGGAACAGCCGAAACCGGTTTGGCTCAGTGGATAGAGCGTCGGCCTGCGGACTGAAAGGTCCCAGGTTCGATTCCGGTCAAGGGCATGTACCTGGGTTGCGGGCACATCCCCAGTAGGAGATGTGCAGGAGGCAGCTGATCGATGTTTCTCTCTCATCGAT
NW00587142 : ACGTGTCATTGAAAACTGTTGGGAAGTGCCAAAACCGGTTTGGCTCAGTGGATAGAGCGTCGGCCTGCGGACTGAAAGGTCCCAGGTTCGATTCCGGTCAAGGGCATGTACCTGGGTTGCGGGCACATCCCCAGTAGGAGATGTGCAGGAGGCAGCTGATCGATGTTTCTCTCTCATCGAT
NW00587144 : GTTGGACATAAAAAAAAAATAAAAACAGCCGAAACCGGTTTGGCTCAGTGGATAGAGCGTCGGCCTGCGGACTGAAAGGTCCCAGGTTCGATTCCGGTCAAGGGCATGTACCTGGGTTGCGGGCACATCCCCAGTAGGAGATGTGCAGGAGGCAGCTGATCGATGTTTCTCTCTCATCGAT
NW00587142 : ATGACTATGATTAAAAGACAGACAATAGCCGAAACCGGTTTGGCTCAGTGGATAGAGCGTCGGCCTGCGGACTGAAAGGTCCCAGGTTCGATTCCGGTCAAGGGCATGTACCTGGGTTGCGGGCACATCCCCAGTAGGAGATGTGCAGGAGGCAGCTGATCGATGTTTCTCTCTCATCGAT
NW00587147 : GAAAATTTCAACAAAAGAGTGCGTATAGCCAAAACCGGTTTGGCTCAGTGGATAGAGCGTCGGCCTGCGGACTGAAAGGTCCCAGGTTCGATTCCGGTCAAGGGCATGTACCTGGGTTGCGGGCACATCCCCAGTAGGAGATGTGCAGGAGGCAGCTGATCGATGTTTCTCTCTCATCGAT
NW00587168 : AACAAGTTGTTTAAAATATAAAATGTGGCCGAAACCGGTTTGGCTCAGTGGATAGAGCGTCGGCCTGCAGACTGAAAGGTCCCAGGTTCGATTCCGGTCAAGGGCATGTACCTGGGTTGCGGGCACATCCCCAGTAGGAGATGTGCAGGAGGCAGCTGATCGATGTTTCTCTCTCATCGAT
NW00587116 : TCCACACTCCTAAAGGATAGACTGCACGCCGAAACCGGTTTGGCTCAGTGGATAGAGCGTCGGCCTGCGGACTGAAAGGTCCCAGGTTCGATTCCGGTCAAGGGCATGTACCTGGGTTGCGGGCACATCCCCAGTAGGAGATGTGCAGGAGGCAGCTGATCGATGTTTCTCTCTCATCGAT
NW00587117 : AAAAATCAACCAAAAAAATGAGTTCACGCCGAAACCGGTTTGGCTCAGTGGATAGAGCGTCGGCCTGCGGACTGAAAGGTCCCAGGTTCGATTCCGGTCAAGGGCATGTACCTGGGTTGCGGGCACATCCCCAGTAGGAGATGTGCAGGAGGCAGCTGATCGATGTTTCTCTCTCATCGAT
NW00587659 : GTGGCAAATTAAAAAAAGAGGGTGTCTGCCGAAACCGGTTTGGCTCAGTGGATAGAGCGTCGGCCTGCGGACTGAAAGGTCCCAGGTTCGATTCCGGTCAAGGGCATGTACCTGGGTTGCGGGCACATCCCCAGTAGGAGATGTGCAGGAGGCAGCTGATCGATGTTTCTCTCTCATCGAT
NW00587125 : CCAGGCAGCTATAAAAAGCAGATCGTTGCCGAAACCGGTTTGGCTCAGTGGATAGAGCGTCGGCCTGCAGACTGAAAGGTCCCAGGTTCGATTCCGGTCAAGGGCATGTACCTGGGTTGCGGGCACATCCCCAGTAGGAGGTGTGCAGGAGGCAGCTGATCGATGTTTCTCTCTCATCGAT
NW00587104 : TGAATTAACATAAAAATAGTATCTCATGCCGAAACCGGTTTGGCTCAGTGGATAGAGCGTCGGCCTGCAGACTGAAAGGTCCCAGGTTCGATTCCGGTCAAGGGCATGTACCTGGGTTGCGGGCACATCCCCAGTGGGAGATGTGCAGGAGGCAGCTGATCGATGTTTCTCTCTCATCGAT
NW00587105 : TTTTCCTCGGATAAAAAAGTATTTCGAGCCGAAACCGGTTTGGCTCAGTGGATAGAGCGTCGGCCTGCAGACTGAAAGGTCCCAGGTTCGATTCCGGTCAAGGGCATGTACCTGGGTTTCGGGCACATCCCCAGTAGGAGATGTGCAGGAGGCAGCTGATCGATGTTTCTCTCTCATCGAT
NW00587126 : TGGTTGTCTAATAAGAATGTTCTTTGGGCCGAAACCGGTTTGGCTCAGTGGATAGAGCGTCGGCCTGCGGACTGAAAGGTCCCAGGTTCGATTCCGGTCAAGGGCATGTACCTGGGTTGCGGGCACATCCCCAGTAGGAGATGTGCAGGAGGCAGCTGATCGATGTTTCTCTCTCATCGAT
NW00587157 : CCATTGTCCCATCAGATTTCACTTACAGCCGAAACCGGTTTGGCTCAGTGGATAGAGCGTCGGCCTGCGGACTGAAAGGTCCCAGGTTCGATTCCGGTCAAGGGCATGTACCTGGGTTGCGGGCACATCCCCAGTAGGAGATGTGCAGGAGGCAGCTGATCGATGTTTCTCTCTCATCGAT
NW00587177 : TGAAAATGCATTAAAAACAAGGGAGAAGCCGAAACCGGTTTGGCTCAGTGGATAGAGCGTCGGCCTGCGGACTGAAAGGTCCCAGGTTCGATTCCGGTCAAGGGCATGTACCTGGGTTGCGGGCACATCCCCAGTAGGAGATGTGCAGGAGGCAGCTGATCGATGTTTCTCTCTCATCGAT
NW00587130 : TGATATGTATTAAAAATAACATGGATCGCCAAAACCGGTTTGGCTCAGTGGATAGAGCGTCGGCCTGCAGAGTGAAAGGTCCCAGGTTCGATTCCGGTCAAGGGCATGTACCTGGGTTGCGGGCACATCCCCAGTAGGAGATGTGCAGGAGGCAGCTGATCGATGTTTCTCTCTCATCGAT
NW00587108 : GAAGGAGATAATAAAGATTAGAGCAGAGCCAAAACCGGTTTGGCTCAGTGGATAGAGCGTCGGCCTGCGGACTGAAAGGTCCCAGGTTCGATTCCGGTCAAGGGCATGTACCTGGGTTGCGGGCACATCCCCAGTAGGAGATGTGCAGGAGGCAGCTGATCGATGTTTCTCTCTCATCGAT
NW00587106 : TAAGGCTGCTATAAACATCTGTGCAGGGCCGAAACCGGTTTGGCTCAGTGGATAGAGCGTCGGCCTGCGGACTGAAAGGTCCCAGGTTCGATTCCGGTCAAGGGCATGTACCTGGGTTGCGGGCACATCCCCAGTAGGAGATGTGCAGGAGGCAGCTGATCGATGTTTCTCTCTCATCGAT
NW00587112 : GTGGTTATGATTAATTTTAAAATTGCAGCCGAAACCGGTTTGGCTCAGTGGATAGAGCGTCGGCCTGCGGACTGAAAGGTCCCAGGTTCGATTCCGGTCAAGGGCATGTACCTGGGTTGCGGGCACATCCCCAGTAGGAGATGTGCAGGAGGCAGCTGATCGATGTTTCTCTCTCATCGAT
NW00587113 : CATTGAACCCACAAAGATGACGTTCCAGCCGAAACCGGTTTGGCTCAGTGGATAGAGCGTCGGCCTGCAGACTGAAAGGTCCCAGGTTCGATTTCGGTCAAGGGCATGTACCTGGGTTGCGGGCACATCCCCAGTAGGAGATGTGCAGGAGGCAGCTGATCGATGTTTCTCTCTCATCGAT
NW00587123 : ATTCAGTGGTAAGATATAAAAAGAACTGCCAAAACCGGTTTGGCTCAGTGGATAGAGCGTCGGCCTGCGGACTGAAAGGTCCCAGGTTCGATTCCGGTCAAGGGCATGTACCTGGGTTGCGGGCACATCCCCAGTAGGAGATGTGCAGGAGGCAGCTGATCGATGTTTCTCTCTCATCGAT
NW00587136 : ACATAAAACAAGATTATATGTATTGCTGCCGAAACCGGTTTGGCTCAGTGGATAGAGCGTCGGCCTGCGGACTGAAAGGTCCCAGGTTCGATTCCGGTCAAGGGCATGTACCTGGGTTGCGGGCACATCCCCAGTAGGAGATGTGCAGGAGGCAGCTGATCGATGTTTCTCTCTCATCGAT
NW00587123 : ATAAAACATGTAATTAGAATCAATCATGCCGAAACCGGTTTGGCTCAGTGGATAGAGCGTCGGCCTGCGGACTGAAAGGTCCCAGGTTCGATTCCGGTCAAGGGCATGTACCTGGGTTGCGGGCACATCCCCAGTAGGAGATGTGCAGGAGGCAGCTGATCGATGTTTCTCTCTCATCGAT
```

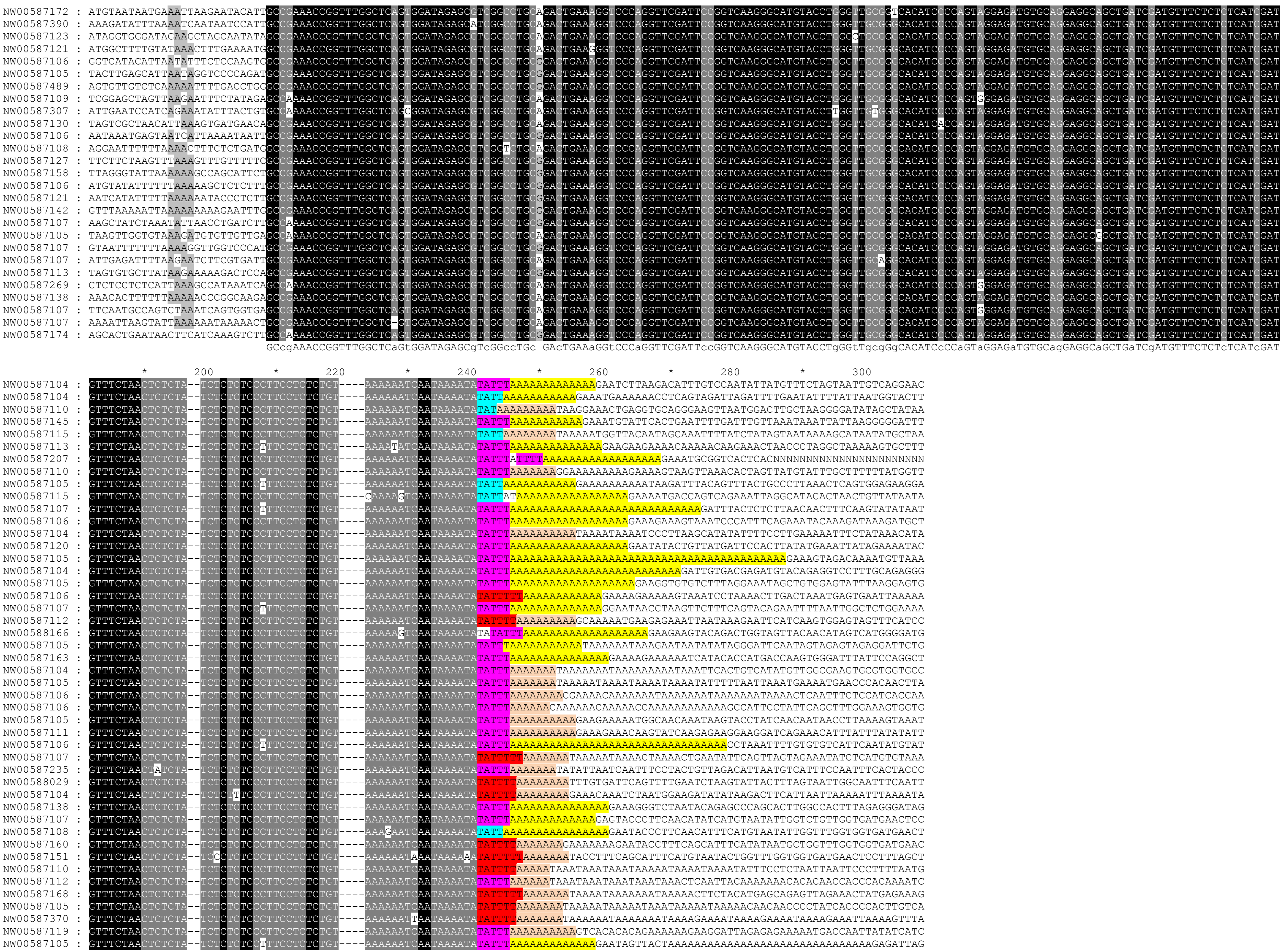

200
220
240
260
280
300

```
NW00587112 : GTTTCTAACTCTCTA--TCTCTCTCCCTTCCTCTCTGT----AAAAAATCAATAAAATATATTAAAGAAAAAAAAAGAAGGTGGAAGGGGAGAAGAAAAGGAGGAGGAGGAGGAGGAGGAGGAG
NW00587106 : GTTTCTAACTCTCTA--TCTCTCTCCCTTCCTCTCTGT----AGAAAATCAATAAAATATATTTAAAAAAAAATGCATTACAACATTTAAAAAAAAAAAAACAAAACAAACAAAAAAAAAAAAAAAC
NW00587104 : GTTTCTAACTCTCTA--TCTCTCTCCCTTCCCCTCTGT----AAAAAATCAATAAAATATATTAAAAAAAAACTGTGGTACATCTACACAATGGAATACTACGCTGCTGTAAAAAAAACTCTTACCA
NW00587154 : GTTTCTAACTCTCTA--TCTCTCTCCCTTCCTCTCTGT----AAAAAATCAATAAAATATATTAAAAAAAAAAAAAAAGAATGAAATCTTGCCTTCTGCAACAATATGGATGGACCTAGAGGCTATT
NW00587104 : GTTTCTAACTCTCTA--TCTCTCTCCCTTCCTCTCTGT----AAAAAATCAATAAAATATATTTAAAAATAAAAATAAAAAAATAAAAATAAAAACTTTCCAACAAAGAAAAGCTCTGGACCACATG
NW00587108 : GTTTCTAACTCTCTA--TCTCTCTCCCTTCCTCTCTGT---AAAAAAATCAATAAAATATATTTTTTTTAAAAAATCTTCCCAGGAAAGAAAAACCCTGGTCCAGATGGCTTCACAGGGTAGTTTTA
NW00587105 : GTTTCTAACTCTCTA--TCTCTCTCCCTTCCTCTCTGT----AAAAAACCAATAAAATATATTTAAAAAAAAAAGAAAATTCTATAACATTTTAGGATTAAGAATTATTTGGAAACCCAACAGACTA
NW00588199 : GTTTCTAACTCTCTA--TCTCTCTCCCTTCCTCTCTGT----AAAAAATCAATAAAATATATTTAAAAAAAAAAAGAAATACCCAAGCAGAGCAGCATCTAGAAAAAAAAAAAAACGTAAAAAACAG
NW00587116 : GTTTCTAACTCTCTA--TCTCTCTCCCTTCCTCTCTGT----AAAAAATCAATAAAATATATTAAAAAAAAAAAAGAAAAGTTTTAAGAAATAGAAATTTAGAAGAAAAGATGCAAGGGGCACTTGG
NW00587118 : GTTTCTAACTCTCTA--TCTCTCTCCCTTCCTCTCTGT----GAAAAATCAATAAAATATATTTAAAAAAAAAAAGATGGAGGTTCAGAGCTGCAGGGAGAAAAAAGGGATGTATATTTCACTCTTA
NW00587106 : GTTTCTAACTCTCTA--TCTCTCTCCCTTTCTCTCTGT----AAAAAATCAATAAAATATATTTAAAAAAATAAAAAAAGAGTTTGTATGTAAAAATACTATCCTGTACATACACACACAACAAACA
NW00587128 : GTTTCTAACTCTCTA--TCTCTCTCCCTTCCTCTCTGT----AAAAAATCAATAAAATATATTTAAAAAAAATAAAAAAATAAAAATAAAATACTATATATATATATATAATGCACCCAGATTAGTAT
NW00587106 : GTTTCTAACTCTCTC--TCTCTCTCCCTTCCTCTCTGT----AAAAAATCAATAAAATATATTTAAAAAAAAAGAAAATGTGTTTAACACATCAAATATAAATATTATCTTTTATAATACAGCACTC
NW00587105 : GTTTCTAACTCTCTA--TCTCTCTCCCTTCCTCTCTGT----AAAAAATCAATAAAATATATTTTTATTTTTTAAAAATGGAATGTGGCATTGGCTCAGTTTTGAAAAAACTTAAGGATTGAGAGAG
NW00587130 : GTTTCTAACTCTCTA--TCTCTCTCCCTTCCTCTCTGT----AAAAAATCAATAAAATATATTTTTTTAAAAAAACAACATATTTTTAAAAATAAATTAAAGTAATTTTTCTAAAAAAAGAAAAGAA
NW00587106 : GTTTCTAACTCTCTA--TCTCTCTCCCTTCCTCTCTGT----AAAAAATCAATAAAATATATTTAAAAAAAAAAAAACTACTACTGAAATAATCTGAAAAATAAAGTCCATTAATTTTTTTTAAATA
NW00587106 : GTTTCTAACTCTCTA--TCTCTCTCCCTTCCTCTCTGT----AAAAAATCAATAAAGTATATTTAAAAAAAAATAACGAGAAAGACTCATTGATTGGCTGCCTCCTGCATGCCCCCTACTGGGGATC
NW00587106 : GTTTCTAACTCTCTCTCTCTCTCTCCCTTCCTCTCTGT----AAAAAATCAATAAAATATATTTAAAAAAAACAAAAAAAAACCAACAACATAATTTTATTGATTTCAGAGAGGAAGGAAGAGGGAG
NW00587116 : GTTTCTAACTCTCTA--TCTCTCTCCCTTCCTCTCTGT----AAAAAATCAATAAAATATATTTAAAAAAAAAAGTTTATTGGTATGATTTTTTAAAATATATTTTTTATTGATTTCAGAGAGGAAA
NW00587131 : GTTTCTAACTCTCTA--TCTCTCTCCCTTCCTCTCTGT----AAAAAATCAATAAAATATATTAAAAAAATATATTGTCATTGATTTTAGAGAGGGAGAGAGAGATAGAAACATCAATAATGAGAGA
NW00587110 : GTTTCTAACTCTCTA--TCCCTCTCCCTTCCTCTCTGT----AAGAAATCAATAAAATATATTAAAAAAAAAAAAAAAAAAAAAAAAAAGACTGACTTGGAAGATAAAGAAAAAAAAAACTGGCATCT
NW00587127 : GTTTCTAACTCTCTA--TCTCTCTCTTCTTCCTCTCTGT----AAAAAATCAATAAAATATAAAAAAAAAAAAAAAAAAGAATTTATAACTGACAAAATAAACTTTAAAACAAAAGTTATAATAAGAG
NW00587135 : GTTTCTAACTCTCTA--TCTCTCTCCCTTCCTCTCTGT----AAAAAATCAATAAAATATATTTTAAAAAAAAAGAAAAGAAAACTGGAAAGCCAAGTGAAGTGGAGGGAAATGAAAAATGCATGAA
NW00587119 : GTTTCTAACTCTCTA--TCTCTCTCCCTTCTTCTCTGT----AAAAAATCAATAAAATATATTAAAAAAAAAAAAAAGAAGTCTCCTAAAAACAAAGAGAAATTTGGAAATATTTGCAATTTTATCA
NW00587128 : GTTTCTAACTCTCTA--TCTCTCTCCCTTCCTCTCTGT----AAAAAATCAATAAAATATATTAAAAAAAAAAAGTAAAATTCTTGTGTGGCATGCAAAGCTATCATATTATGTCCATAGCTACCTC
NW00587404 : GTTTCTAACTCTCTA--TCTCTCTCCCTTCCTCTCTGT----AAAAAATCAATAAAATATATTTAAAAAAAAAAGATATTTTAACCCTTTGTGTCTAAAGATATGCTTTAGATAGTTTTCTCATTAG
NW00587124 : GTTTCTAACTCTCTA--TCTCTCTCCCTTCCTCTCTGT----AAAAAATCAATAAAATATATTTAAAAAAAAAAAAAAAGAAAGCCCACTGCATGGGAGAACATATTTGCCAATGTTATCACCGATA
NW00587109 : GTTTCTAACTCTCTA--TCTCTCTCCCTTCCTCTCTGT----AAAAAATCAATAAAATATATTTTAAAAAAAAAAGTCAAGAGCTAGGGCGCCCCGCAAAGCCTTATTTTCTATCCTAAAGTAACAG
NW00587135 : GTTTCTAACTCTCTA--TCTCTCTCCCTTCCTCTCTGT----AAAAAATCAATAAAATATATTAAAAAAAAAAATAACAAGTTTTAGGTCAACAAAAGGCATTGCGTTGCTTTCTGGCCCTACTTAC
NW00587105 : GTTTCTAACTCTCTA--TCTCTCTCCCTTCCTCTCTGT----AAAAAATCAATAAAATATATATATAAAAAAAAAGAAGTACTAATCGGTACAGAATAGGCATGGGGATGTACAATACAACATAGGGA
NW00587107 : GTTTCTAACTCTCTA--TCTCTCTCCCTTCCTCTCTGT----AAAAAATCAATAAAATATATTTTAAAAAAAAAGAAATACAGATTGGTAGTTACAATATAGTCAGTACAGGGAATATAGTCAGTAA
NW00587105 : GTTTCTAACTCTCTA--TCTCTCTCCCTTCCTCTCTGA----AAAAAATCAATAAAATATATTTTTTAAAAAAGAGACTTCATAAGATAATAGATTACCTCTGTATTCACATCTTACTGAGATAGAT
NW00587105 : GTTTCTAACTCTCTA--TCTCTCTCCCTTCCTCTCTGT----AAAAAATCAATGAAATATATTTTAAAAAAATAAAATAAATTTTATTTTTCCATAAATATAGTTTTACTTTGCTTGTACAATAAAT
NW00587107 : GTTTCTAACTCTCTA--TCTCTCTCCCTTCCTCTCTGT----AAAAAATCAATAAAATATATTTTTTTAAAAAATTTTTTATTGAGGTATCATTTGGGGATTTTTTTTTCATTTATTATGCTGAGCA
NW00587143 : GTTTCTAACTCTCTA--TCTCTCTCCCTTCCTCTCTGT----AAAAAATCAATAAAATATATTTTTTAAAAAAAAGATTGCTGAAGCTATTTGGGGTCTTTTGGGTTCCATATAAATTTTTGGAATA
NW00587147 : GTTTCTAACTCTCTA--TCTCTCTCCCTTCCTCTCTGT----AAAAAATCAATAAAGTATATTTTAAAAAAATTGTTATTTGGGTATATTAGTATATGGATAATTATTTATACAAGTATTAAACTTG
NW00587109 : GTTTCTAACTCTCTA--TCTCTCTCCCTTTCTCTCTGT----AAAAAATCAATAAAATATATTTTAAAAAAATGTATATTCAAACCCTTTGCTCATTTTAAAATTGAGTGGTTTGTCTTTTTGTTGT
NW00587127 : GTTTCTAACTCTCTA--TCTCTCTCCCTTCCTCTCTGT----GGAAAATCAATAAAATATATTTTTTAAAAAAAGTATTTTAACAAAGTAATGACTTCTTAGCCATAGAACTTGAAAAGCTCAAAT
NW00587111 : GTTTCTAACTCTCTA--TCTCTCTCCCTTCCTCTCTGT----AAAAAATCAATAAAATATATTTTAAAAAAAAAAAGTTTTGCTATTAAGATTAAACAATTTTTTCTCAATATTCAGGCTATTCAGA
NW00587122 : GTTTCTAACTCTCTG--TCTCTCTCCCTTCCTCTCTGT----AAAAAATCAATAAAATATATTTAAAAAAAAATAATAATAATTTTTTCTAGTTAGAAAAAAACCTCCATGCTGCTCTCAAAAATTT
NW00587105 : GTTTCTAACTCTCTA--TCTCTCTCCTTTCCTCTCTGT----AAAAAATCAATAAAATATATTTAAAAAAAAAAAAAAGAAAGAAAAAACTCCTCATCTTCAGTGATATACTCTTCCTAAGAGCAGC
NW00587105 : GTTTCTAACTCTCTA--TCTCTCTCCCTTCCTCTCTGT----AAAAAATCAATAAAATATATTTAAAAAAAAAAAAAAGATTGTATTTTATGCCCCCTCTCTCCAGAATTTTATATATAGAGTGAGG
NW00587119 : GTTTCTAACTCTCTA--TCTCTCTCCCTTCCACTCTGT----AAAAAATCAATAAAATATATTTAAAAAAAAAGAATAGGTGTGATGATTGGACAGGTTACAAGTAGTAAATAGTAATTAAAAAAAA
NW00587175 : GTTTCTAACTCTCTA--TCTCTCTCCCTTTCTCTCTGT----AAAAAATCAATAAAATATATTTAAAAAAAAAAAAATTGAATGATTAGATTTTTTTATCTTTAATTTATTTGTATTGACAATTAGCT
NW00587118 : GTTTCTAACTCTCTA--TCTCTCTCCCTTTCTCTCTGT----AAAAAATCAATAAAATATATTTAAAAAAAAATATATATATATTCTTGTTGAGGATCTCCTGTAATTCTTTTTATGGTTTAAATTCT
NW00587162 : GTTTCTAACTCTCTA--TCTCTCTCCCTTCCTCTCTGT----AAAAAATCAATAAAATACATTTAAAAAAAAAAAAAAGAATTAGGTGGGATTTTTTTGTTTGTTTGTTTGTTTTGTTTTGTTTTGGA
NW00587107 : GTTTCTAACTCTCTA--TCTCTCTCTTCTTCCTCTCTGT----AAAAAATCAATAAAATATATTTTTAAAAAATAAAAATAAAAACAAGGGTCAATCTCCTTCACCTGTGAACATTCCTATCCTTTTG
NW00587105 : GTTTCTAACTCTCTA--CCTCTCTCCCTTCCTCTCTGT----AAAAAATCAATAAAATATATTTTTTTAAAAAAAGAGAAAAAAATATTTTTAAAAATAAATTGACTGTAGATGTATGGAGTTTATTTC
NW00587117 : GTTTCTAACTCTCTA--TCTCTCTCCCTTCCTCTCTGT----AGAAAATCAATAAAATATATTTTTTTAAAAAAAGAACTATATACTAATCATCAAAGATATATTTTTGATGGATTCTCTTAAAATAT
NW00587105 : GTTTCTAACTCTCTA--TCTCTCTCCCTTCCTCTCTGT----AAAAAATCAATAAAATATATTTAAAAAAAATATTAACTAGAATAATTCCATCTTTTCCATCTTTCTAATTAAAAACTCAGATCAA
NW00587118 : GTTTCTAACTCTCTTTATCTCTCTCCCTTCCACTCTGT----AAAAAACCAATAAAATATATTAAAAAAATAATATCTAAAATATTATCTTGTGTTTTATCTTATGAATTTGAAATCTCTTTTTATA
NW00587105 : GTTTCTAACTCTCTA--TCTCTCTCCCTTCCTCTCTGT----AAAAAATCAATAAAATATATTTCTTTCAAAATTATTATCTGCTATAGTCCAAGTTATTGCATATTATCAATTTATTAACTAGTAT
NW00587132 : GTTTCTAACTCTCTA--TCTCTCTCCCTTCCTCTCTGT----AAAAAATCAATAAAATATATTTTTAAAAAAATGAACATAGTAACCACATTGTAATTTTTTGCAATTTTATTTATTTATTTTTATT
NW00587113 : GTTTCTAACTCTCTA--TCTCTCTCCCTTCCTCTCTGT----AAAAAATCAATAAAATACATTTAAAAAAAATAAAATAAAGTATTACAAAGAGTATTACATATGTCTCCTTCCACCCCCCCCCTGC
NW00587119 : GTTTCTAACTCACTA--TCTCTCTCCCTTCCTCTCTGT----AAAAAATCAATAAAATATATTTTTAAAAAAATTTAAATTCTTTATTATTTAAAGTATTACATAAGTCTCCTTTTCCCCCTCTTGA
NW00587112 : GTTTCTAACTCTCTA--TCTCTCTCCCTTCCTCTCTGT----AAAAAATCAATAAAATATATTTTAAAAAAAATGATGGTGATGTGCTGTAGAATATTTGTCATATTTACTTTTATCCTTATCTTAT
NW00587105 : GTTTCTAACTCTCTA--TCTCTCTCCCTTCCTCTCTGT----AAAAAATCAATAAAATATATTAAAAAAAAAAAATGTGCATACTGTCCTTGTGAGTTTGCTTTTAGTTAACAAACAGAGTTTATTT
NW00587106 : GTTTCTAACTGTCTA--TCTCTCTCCCTTCCTCTCTAT----AAAAAATCAATAAAATATATTTAAAAAAATGATTTTAAATATAATAATGTTACATGAAGTAAACCTTAATATTTCAAGAAAACCA
NW00587122 : GTTTCTAACTCTCTA--TCTCTCTCCCTTCCTCTCTGT----AAAAAATCAATAAAATATATTAAAAAAAAGAGAGAAATAAATATTGGCTCCAATTTTGACACCAGCACTTAACAATTGAGAGTTT
NW00587120 : GTTTCTAACTCTCTA--TCTCTCTCCCTTCCTCTCTGT----AAAAAATCAATAAAATATATTTAAAAAAAAATTTGTCTACGCTTTTCAAGACTCCTTTTTCCTCGTTTTCTTAATGGGAACAAAT
NW00587184 : GTTTCTAACTATCTA--TCTCTCTCCCTTCCTCTCTGT----AAAAAATCAATAAAATATATTAAAAAAAAAAAAAACTACTAGATTTAATAAATGAATTCGGCAATGTAGCACGATAAAAAAAAATC
NW00587105 : GTTTCTAACTCTCTA--TCCCTCTCCCTTCCTCTCTGT----AAAAAATCAAATCACTATATATATATATATATATATATATATATATATATATATATATATATAAATAAATAAATAAATGGTAAGTGTCT
NW00587136 : GTTTCTAACTCTCTA--TCTCTCTCCCTTCCTCTCTGT----AAAAAATCAATAAAATATATTTTAAAACATATATATATATATATATATATATATATATATATATATATATAAGAATAGGCTAGTGGGCC
NW00587119 : GTTTCTAACTCTCTA--TCTCTCTCCCTTCCTCTCTGT----AAAAACTCAATAAAATATATTTTTTTAAAAAAAGAAAATCAGGTATAGTCATACTTATATCTGACCAAATAGATTTAAAGATAAC
NW00587106 : GTTTCTAACTCTCTA--TCTCTCTCCCTTCCTCTCTGT----AAAAAATCAATAAAATATATTTTAAAAAATTTCATCACAAATGTTGTAGCATTACTTATAATAGCTCCAAACTGGAAGCAACTCA
NW00587109 : GTTTCTAACTCTCTA--TCTCTCTCCCTTCCTCTCTGT----AAAAAATCAATAAAATATATTTTAAAAAATTTGTTTAAGAATGAATAGAATTTTTCATAAAGACATAAAACAAAATGAGAAAATT
NW00587114 : GTTTCTAACTCTCTA--TCTCTCTCCCTTCCTCTCTGT----AAAAAATCAATAAAAATATATTTAAAAAAAAAAGAAACAACCTGCCAAAGGGCTAATATTAAATCTTCCTGGTCAGAAATAATT
NW00587115 : GTTTCTAACTCTCTA--TCTCTCTCCCTTCCTCTCTGT----AAAAAATCAATAAAATATATTTTTAAAAAAAAGAAAAATGAATATTAACGCAAAGGCAAAATCTCTATTAACTGATCAATAGAGG
NW00587107 : GTTTCTAACTCTCTA--TCTCTCTCCCTTCCTCTCTGT----AAAAAATCAATAAAATATATTTAAAAAAAAAATAAAAGACTATCCTTATTTTCAATGTAATTTCTTTAAAAAGGACTGCTAGGGT
NW00587639 : GTTTCTAACTCTCTA--TCTCTCTCCCTTCCTCTCTGT----AGAAAATCAATAAAATATATTTAAAAAAAAAAAAAATGCCTGTGTTTTGAGCCTCGTCTGGACGCTTGAACAGTAACAACAGATG
NW00587153 : GTTTCTAACTCTCTA--TCTCTCTCTTCTTCCTCTCTGT----AAAAAACCAATAAAATATATTTAAAAAAAAATATGATTGCAGTGTATTTGGAAGAAATGGCTGAAATTTATTGAACATTGACTTG
NW00587105 : GTTTCTAACTCTCTA--TCTCTCTCCCTTTCTCTCTGT----AAAAAATCAATAAAATATATTAAAAAAAATAGTAATAATACCAAATACTGGTGATGATGCTGAGAAACTGATTAGAGGGGTCAGG
NW00587107 : GTTTCTAACTCTCTA--TCTCTCTCCCTTCCTCTCTGT----AAAAAATCAATAAAATATATTAAAAAAAATAGAAGGTATACTTGAGTCTGAAAATTGGACTCAGGAAGACATGGTAATGCAAAAG
NW00587107 : GTTTCTAACTCTCTA--TCTCTCTCCCTTCCTCTCTGT----AAAAAATCAATAAAATACATTTTAAAAAAAAAAGAATTACCATGTGATCCAGCAATTCCTCTTCTGGGTATATACCCAAAAGAAT
NW00587110 : GTTTCTAACTCTCTA--TCTCTCTCCCTTCCTCTCTGT----AAAAAATCAATAAAATATATTTTAAAAAAATAGAACTATCATATAGTCCTTCTTTTTCCTTGCTCCATAAAGCATGGACACAATG
NW00587891 : GTTTCTAACTCTCTA--TCTCTCTCCCTTCCTCTCTGT----GAAAAATCAATAAAATATATTTAAAAAAAAAAGGGAGGATGTGTTCTTTCTTTGGCCCTTCTTTCTTCTAGCTGATTGGAATGCT
```

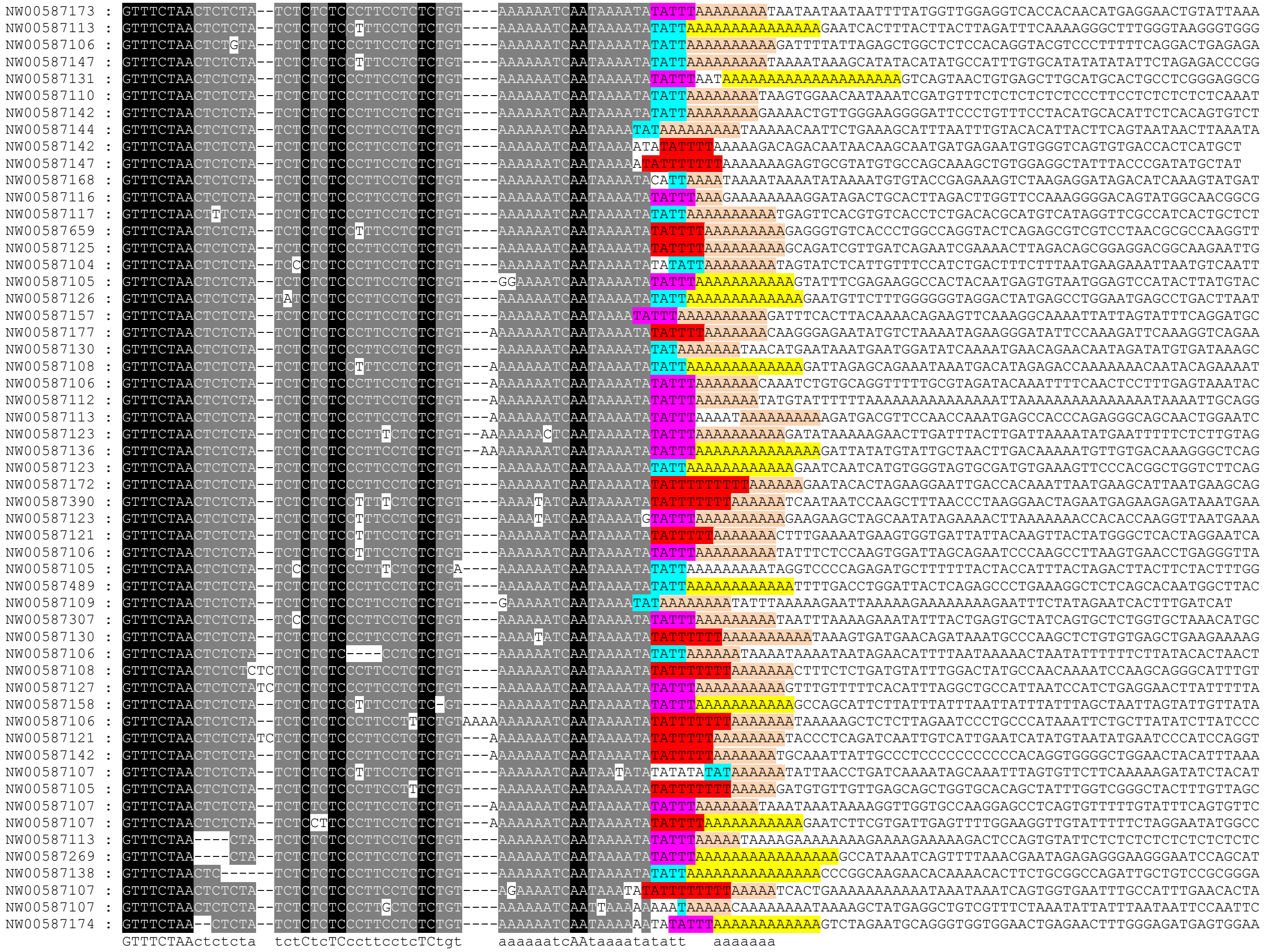

Figure S32. A young Ves tribe Ves_a1Y_187 from *M. lucifugus* genome. Moderate and strong terminators are marked in magenta and red, respectively. Rudimentary terminators are shown in blue. Oligo(A)s with $A_{\geq 11}$ and $A_{\leq 10}$ are highlighted in yellow and beige, respectively.
