# Supplementary material for: Analysis of SINE Families B2, Dip, and Ves with Special Reference to Polyadenylation Signals and Transcription Terminators
Source: Int J Mol Sci. 2021 Sep 13;22(18):9897. doi: 10.3390/ijms22189897 (PMC8466645; doi:10.3390/ijms22189897)
Supplement: Supplementary file 1 [file ijms-22-09897-s001.zip › Supplementary Vassetzky/SINE Ves. Figs S23-S32. Table S9/Table S9.pdf]

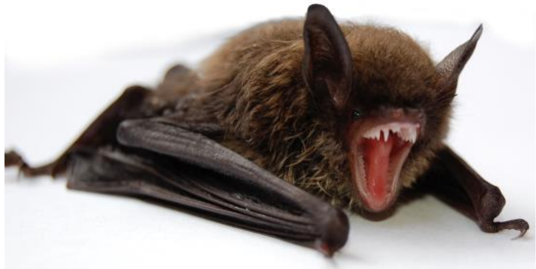

Little brown bat

Myotis lucifugus

Table S9

Incidence of TNT_mA_n patterns (≥5% & >1 sequence) **in Ves_a** (155,132 sequences), **Ves_b** (154,451 sequences), and **Ves_c** (25,157 sequences) **banks**

Ves_a

	A_{5-10}			A_{11-20}			$A_{>20}$		
T_2	T_2	100%	30,838	T_2	100%	10,050	T_2	100%	507
TNT	T_3<br>TAT<br>TGT<br>TCT	70.7%<br>17.2%<br>6.1%<br>6.1%	21,789<br>5,292<br>1,880<br>1,877	TAT<br>TCT<br>TGT	54.6%<br>22.8%<br>22.6%	220<br>92<br>91	TAT<br>TCT	64.0%<br>24.0%	16<br>6
TNT_2	T_4<br>TAT_2<br>Others	68.0%<br>23.8%<br>8.2%	13,785<br>4,816<br>1,662	n/a			n/a		
TNT_3	T_5<br>TAT_3<br>Others	56.4%<br>35.5%<br>8.1%	9,367<br>5,890<br>1,345	n/a			n/a		
$TNT_{\geq 4}$	T_6<br>T_7<br>TAT_5<br>TAT_4<br>TAT_6<br>TAT_7<br>T_8<br>Others	20.4%<br>16.6%<br>13.8%<br>13.7%<br>11.3%<br>7.9%<br>5.8%<br>10.5%	6,431<br>5,219<br>4,345<br>4,316<br>3,546<br>2,496<br>1,835<br>3,310	n/a			n/a		

Ves_b

	A_{5-10}			A_{11-20}			$A_{>20}$		
T_2	T_2	100%	23,427	T_2	100%	1,220	T_2	100%	10
TNT	T_3<br>TAT<br>TCT<br>TGT	65.5%<br>18.3%<br>8.5%<br>7.7%	14,766<br>4,126<br>1,915<br>1,727	TCT<br>TGT TAT	47.7%<br>31.5%<br>20.7%	53<br>35<br>23	TCT<br>TGT	57.1%<br>28.6%	4<br>2
TNT_2	T_4<br>TAT_2<br>TCT_2<br>TGT_2	71.0%<br>15.6%<br>8.1%<br>5.3%	8,973<br>1,972<br>1,026<br>674	n/a			n/a		
TNT_3	T_5<br>TAT_3<br>TCT_3<br>TGT_3	66.1%<br>19.3%<br>9.3%<br>5.3%	5,639<br>1,646<br>794<br>449	n/a			n/a		
$TNT_{\geq 4}$	T_6<br>TAT_4<br>TAT_5<br>T_7<br>TAT_6<br>Others	28.7%<br>14.3%<br>12.1%<br>12.0%<br>6.3%<br>26.6%	3,049<br>1,520<br>1,281<br>1,279<br>673<br>2,826	n/a			n/a		

Ves_c

	A_{5-10}			A_{11-20}			$A_{>20}$		
T_2	T_2	100%	4,772	T_2	100%	830	T_2	100%	22
TNT	T_3<br>TAT<br>TCT<br>TGT	65.1%<br>18.6%<br>8.9%<br>7.4%	1916<br>546<br>261<br>219	TCT<br>TGT<br>TAT	52.2%<br>26.1%<br>21.7%	12<br>6<br>5	n/a		
TNT_2	T_4<br>TAT_2<br>TCT_2<br>TGT_2	55.8%<br>24.8%<br>10.4%<br>8.9%	627<br>279<br>117<br>100	n/a			n/a		
TNT_3	T_5<br>TAT_3<br>TCT_3<br>TGT_3	55.4%<br>25.6%<br>12.5%<br>6.5%	394<br>182<br>89<br>46	n/a			n/a		
$TNT_{\geq 4}$	T_6<br>T_7<br>TAT_4<br>TAT_5<br>T_8<br>TAT_6<br>Others	21.2%<br>15.4%<br>11.2%<br>10.3%<br>8.1%<br>7.1%<br>26.7%	394<br>166<br>120<br>111<br>87<br>76<br>287	n/a			n/a		

Terminator statistics

Ves_a	A_{5-10}	
T_2, TVT	39,887	30.7%
T_3, TVT_2	28,267	21.7%
T_4, TVT_3	21,020	16.1%
$T_{\geq 5}$, $TVT_{\geq 4}$	40,865	31.4%
Total	130,039	

Ves_b	A_{5-10}	
T_2, TVT	31,195	40.0%
T_3, TVT_2	18,438	23.7%
T_4, TVT_3	11,907	15.3%
$T_{\geq 5}$, $TVT_{\geq 4}$	16,267	20.9%
Total	77,807	

Ves_c	A_{5-10}	
T_2, TVT	5,798	53.7%
T_3, TVT_2	2,412	22.3%
T_4, TVT_3	944	8.8%
$T_{\geq 5}$, $TVT_{\geq 4}$	1,635	15.2%
Total	10,789	

V = A, C or G
