# Supplementary figures and images for: Analysis of SINE Families B2, Dip, and Ves with Special Reference to Polyadenylation Signals and Transcription Terminators
Source: Int J Mol Sci. 2021 Sep 13;22(18):9897. doi: 10.3390/ijms22189897 (PMC8466645; doi:10.3390/ijms22189897)

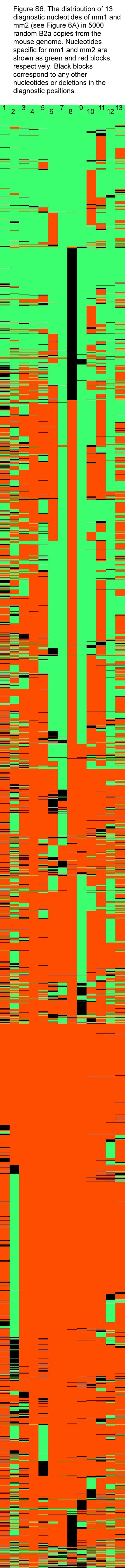

Supplement: Supplementary file 1 [file ijms-22-09897-s001.zip › Supplementary Vassetzky/SINE B2. Figs S1-S6. Tables S1-S5/Fig S6.jpg]

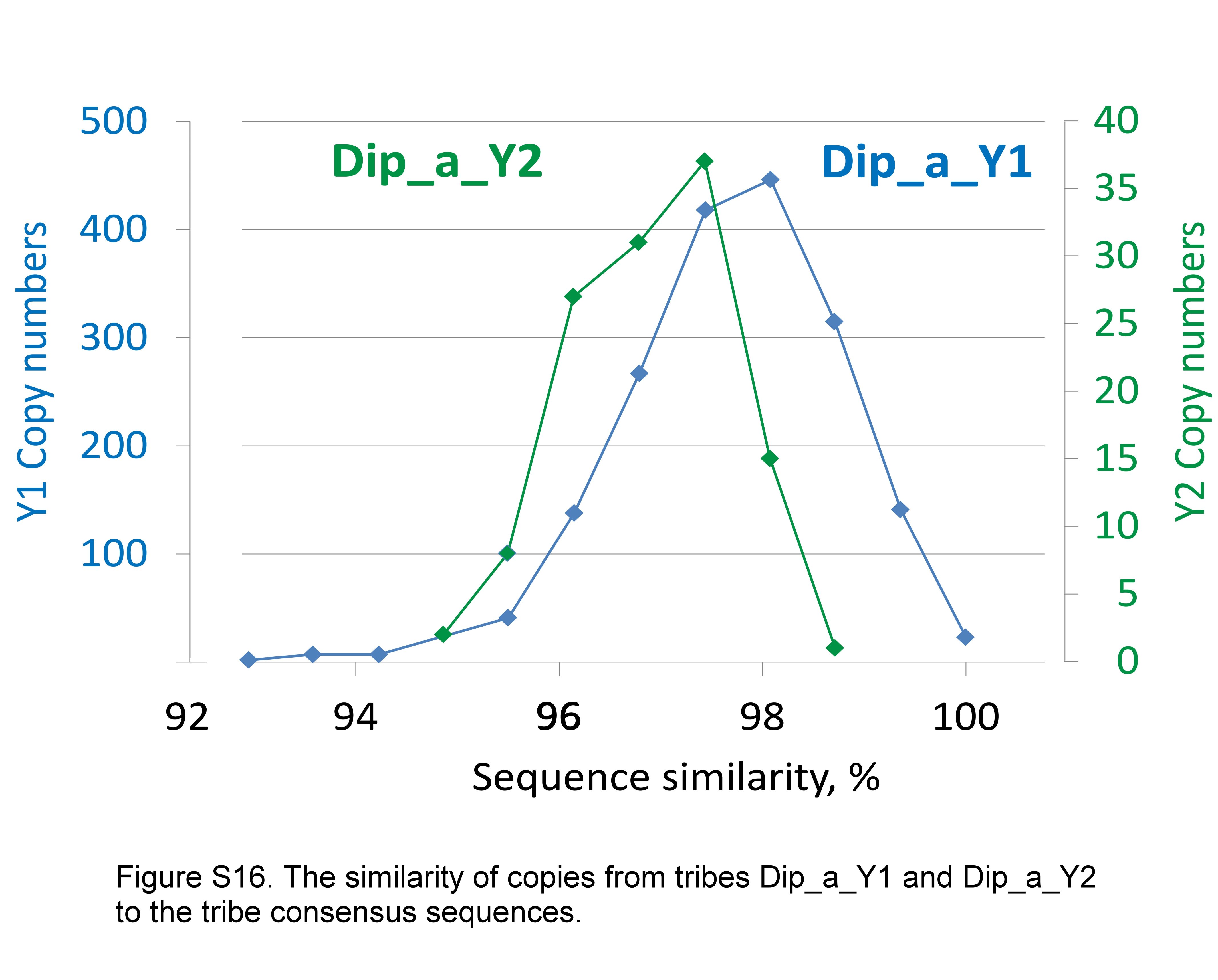

Supplement: Supplementary file 1 [file ijms-22-09897-s001.zip › Supplementary Vassetzky/SINE Dip. Figs S7-S22. Tables S6-S8/Fig S16.jpg]

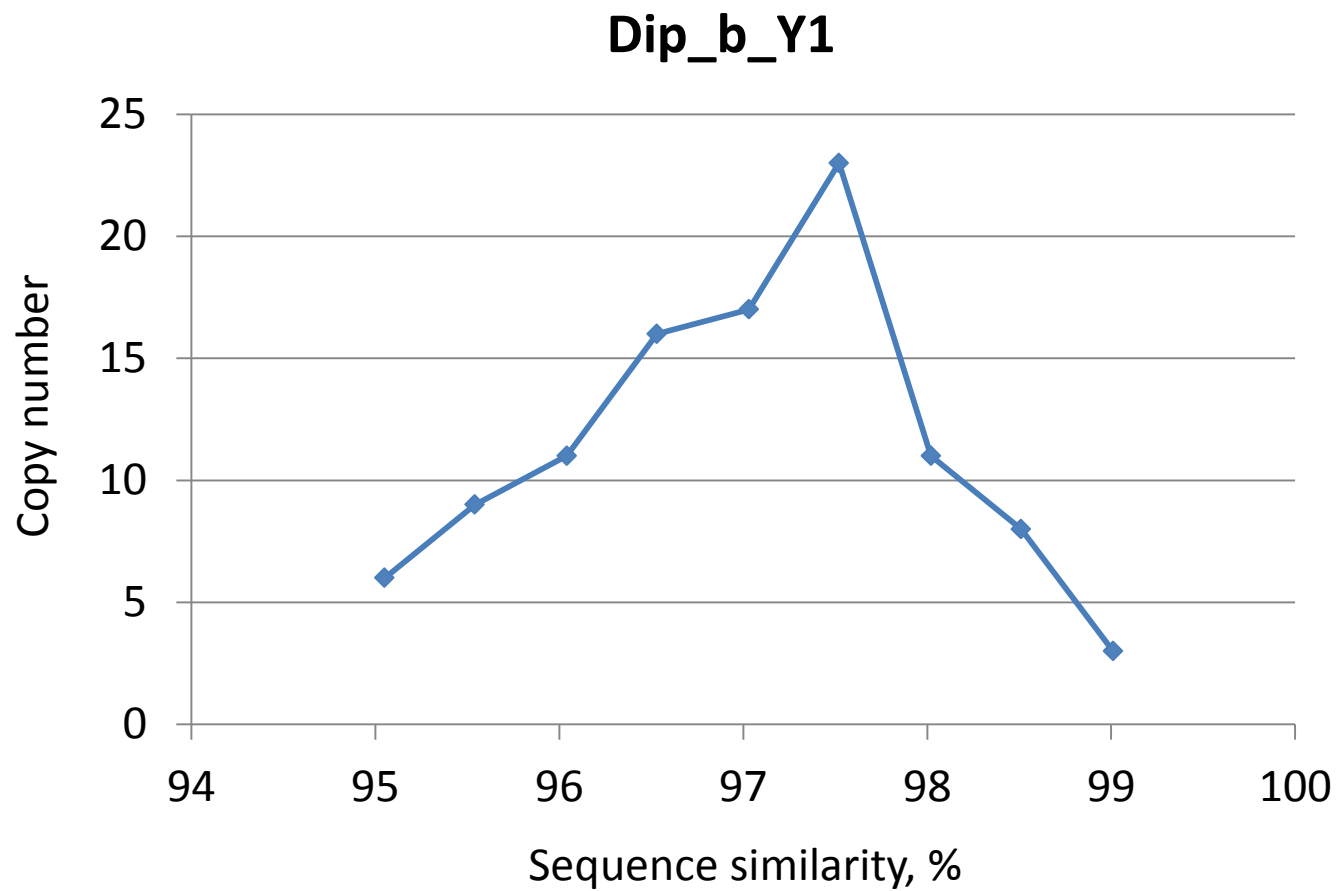

**Figure S18.** The similarity of copies from tribe Dip\_b\_Y1 to the tribe consensus.

Supplement: Supplementary file 1 [file ijms-22-09897-s001.zip › Supplementary Vassetzky/SINE Dip. Figs S7-S22. Tables S6-S8/Fig S18.pdf]

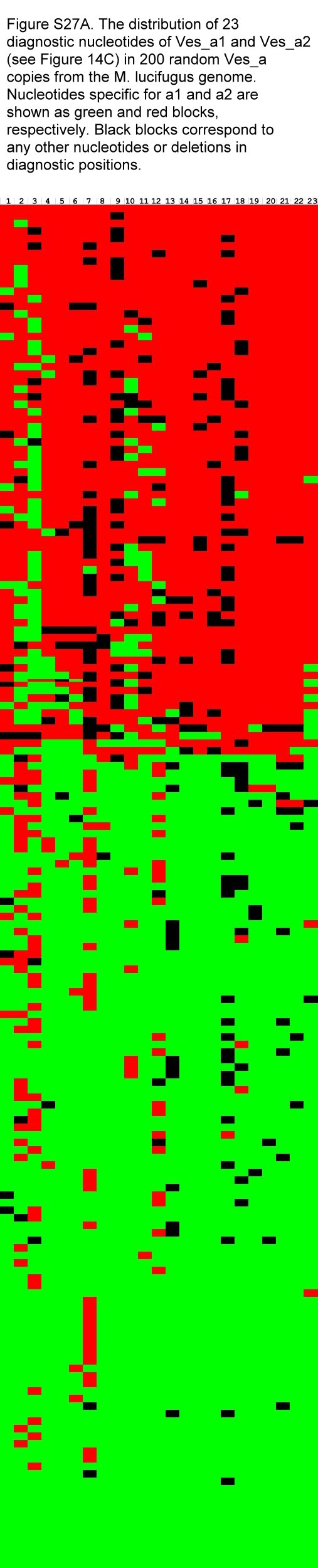

Supplement: Supplementary file 1 [file ijms-22-09897-s001.zip › Supplementary Vassetzky/SINE Ves. Figs S23-S32. Table S9/Fig S27A.jpg]
