# Supplementary material for: Analysis of SINE Families B2, Dip, and Ves with Special Reference to Polyadenylation Signals and Transcription Terminators
Source: Int J Mol Sci. 2021 Sep 13;22(18):9897. doi: 10.3390/ijms22189897 (PMC8466645; doi:10.3390/ijms22189897)
Supplement: Supplementary file 1 [file Fig S2.pdf › Supplementary Vassetzky/SINE B2. Figs S1-S6. Tables S1-S5/Fig S2.pdf]

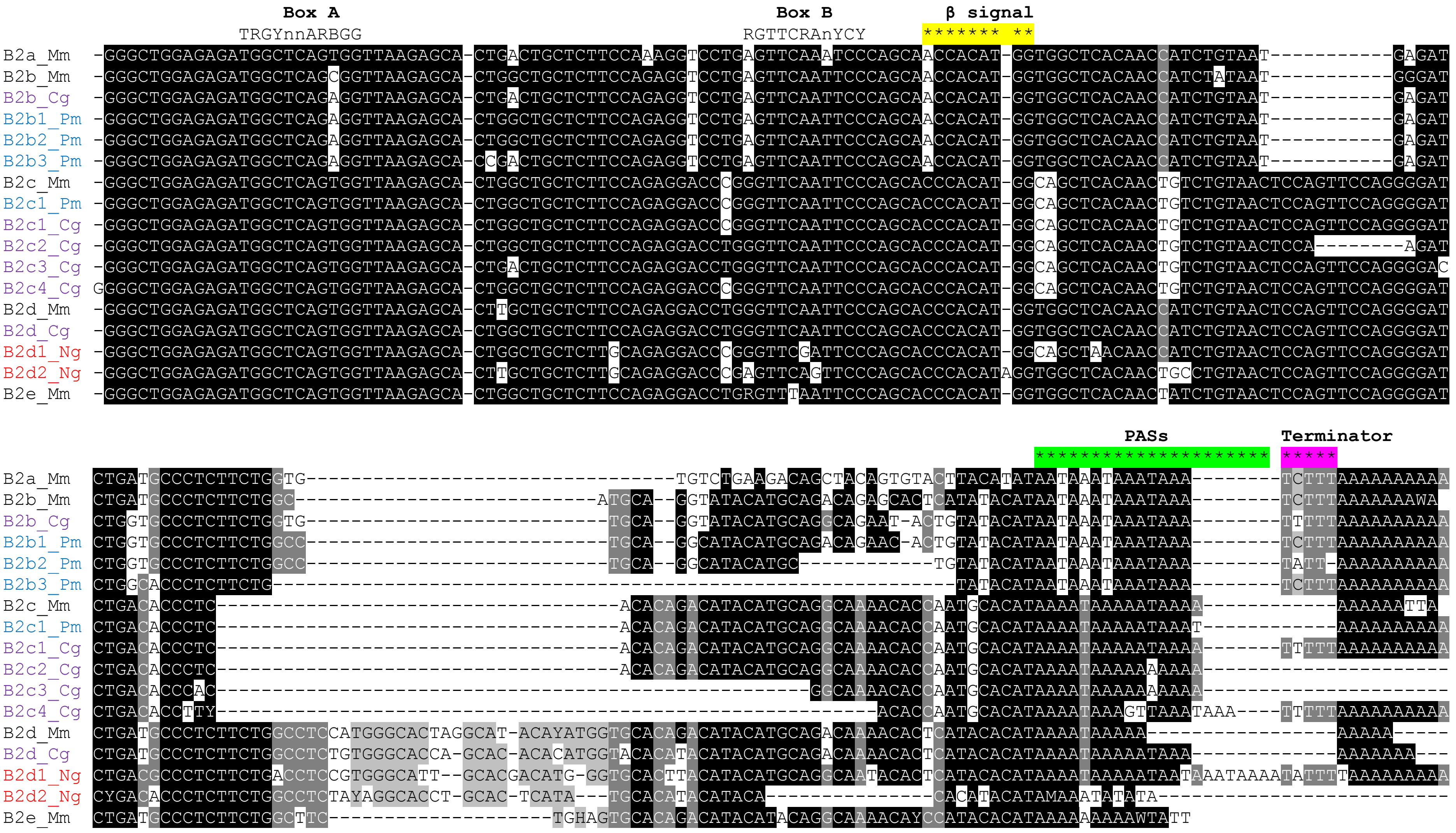

Figure S2. Consensus sequences of B2 subfamilies from cricetids *Cricetulus griseus* (Cg) and *Peromyscus maniculatus* (Pm), as well as from a spalacid *Nannospalax galili* (Ng). B2 subfamilies from the *Mus musculus* (Mm) are shown for comparison. Consensus sequences of the A and B boxes of pol III promoter are shown above the alignment. β signal, PASs, and pol III transcription terminator are marked by asterisks in different colors.
