# Supplementary material for: Analysis of SINE Families B2, Dip, and Ves with Special Reference to Polyadenylation Signals and Transcription Terminators
Source: Int J Mol Sci. 2021 Sep 13;22(18):9897. doi: 10.3390/ijms22189897 (PMC8466645; doi:10.3390/ijms22189897)
Supplement: Supplementary file 1 [file Fig S15.pdf › Supplementary Vassetzky/SINE Dip. Figs S7-S22. Tables S6-S8/Fig S15.pdf]

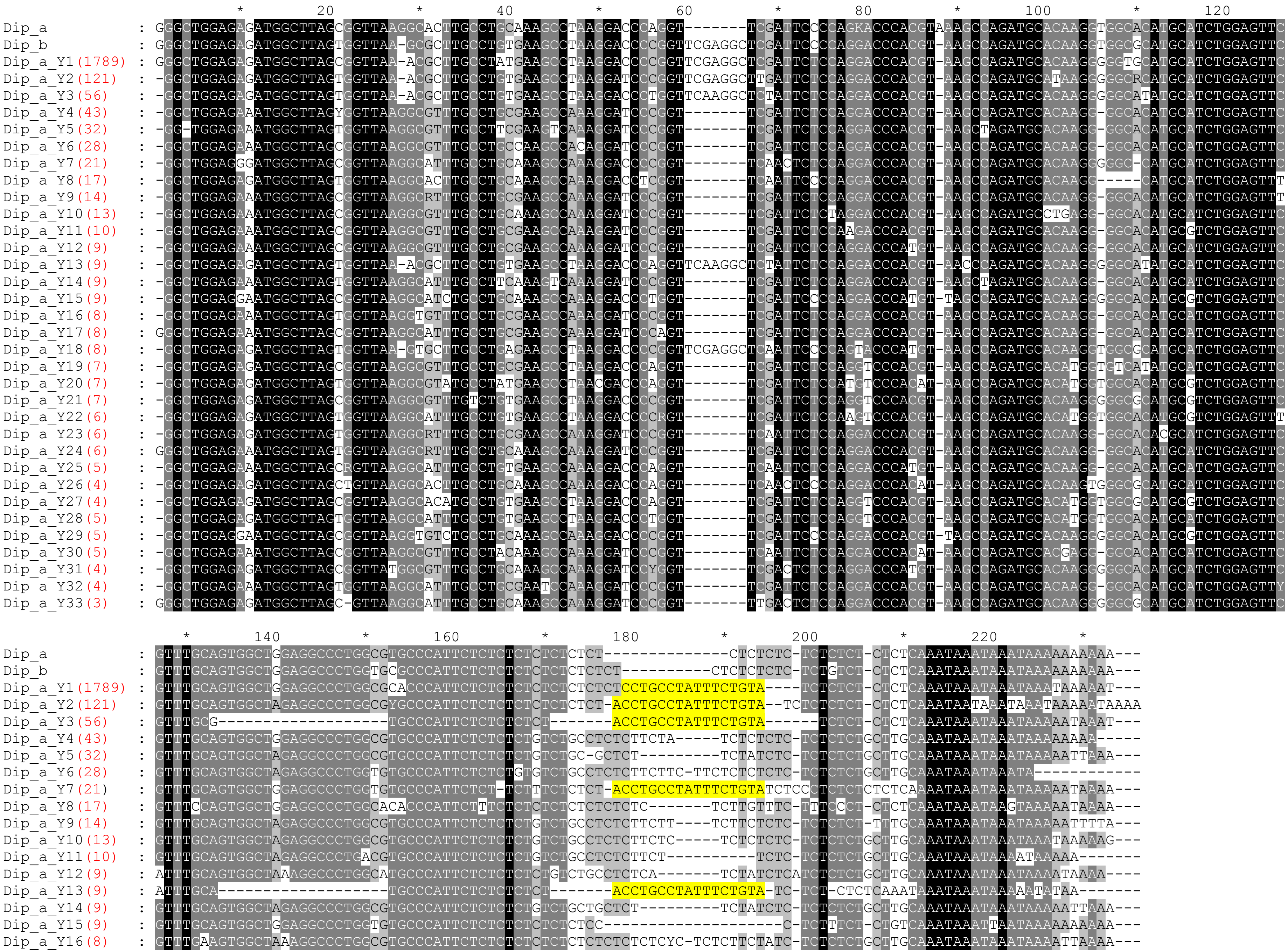
* 20 * 40 * 60 * 80 * 100 * 120
Dip_a : GGGCTGGAGAGATGGCTTAGCGGTTAAGGCACTTGCCTGCAAAGCCTAAGGACCCAGGT-------TCGATTCCCCAGKACCCACGTAAAGCCAGATGCACAAGGTGGCACATGCATCTGGAGTTC
Dip_b : GGGCTGGAGAGATGGCTTAGTGGTTAA-GCGCTTGCCTGTGAAGCCTAAGGACCCCGGTTCGAGGCTCGATTCCCCAGGACCCACGT-AAGCCAGATGCACAAGGTGGCGCATGCATCTGGAGTTC
Dip_a_Y1(1789) : GGGCTGGAGAGATGGCTTAGCGGTTAA-ACGCTTGCCTATGAAGCCTAAGGACCCCGGTTCGAGGCTCGATTCTCCAGGACCCACGT-AAGCCAGATGCACAAGGGGTGCATGCATCTGGAGTTC
Dip_a_Y2(121) : -GGCTGGAGAGATGGCTTAGTGGTTAA-ACGCTTGCCTGTGAAGCCTAAGGATCCCGGTTCGAGGCTTGATTCTCCAGGACCCACGT-AAGCCAGATGCATAAGGGGGCRCATGCATCTGGAGTTC
Dip_a_Y3(56) : -GGCTGGAGAGATGGCTTAGTGGTTAA-ACGCTTGCCTGTGAAGCCTAAGGACCCTGGTTCAAGGCTCTATTCTCCAGGACCCACGT-AAGCCAGATGCACAAGGGGGCATATGCATCTGGAGTTC
Dip_a_Y4(43) : -GGCTGGAGAAATGGCTTAGYGGTTAAGGCGTTTGCCTGCGAAGCCAAAGGATCCCGGT-------TCGATTCTCCAGGACCCACGT-AAGCCAGATGCACAAGG-GGCACATGCATCTGGAGTTC
Dip_a_Y5(32) : -GG-TGGAGAAATGGCTTAGTGGTTAAGGCGTTTGCCTTCGAAGTCAAAGGATCCCGGT-------TCGATTCTCCAGGACCCACGT-AAGCTAGATGCACAAGG-GGCACATGCATCTGGAGTTC
Dip_a_Y6(28) : -GGCTGGAGAAATGGCTTAGCGGTTAAGGCGTTTGCCTGCCAAGCCACAGGATCCCGGT-------TCGATTCTCCAGGACCCACGT-AAGCCAGATGCACAAGG-GGCACATGCATCTGGAGTTC
Dip_a_Y7(21) : -GGCTGGAGGGATGGCTTAGCGGTTAAGGCATTTGCCTGCAAAGCCAAAGGACCCCGGT-------TCAACTCTCCAGGACCCACGT-AAGCCAGATGCACAAGGGGGC-CATGCATCTGGAGTTC
Dip_a_Y8(17) : -GGCTGGAGAGATGGCTTAGTGGTTAAGGCACTTGCCTGCAAAGCCAAAGGACCTCGGT-------TCAATTCCCCAGGACCCACGT-AAGCCAGATGCACAAGG-----CATGCATCTGGAGTTT
Dip_a_Y9(14) : -GGCTGGAGAAATGGCTTAGCGGTTAAGGCRTTTGCCTGCGAAGCCAAAGGATCCCGGT-------TCGATTCTCCAGGACCCACGT-AAGCCAGATGCACAAGG-GGCACATGCATCTGGAGTTT
Dip_a_Y10(13) : -GGCTGGAGAAATGGCTTAGCGGTTAAGGCGTTTGCCTGCAAAGCCAAAGGATCCCGGT-------TCGATTCTCTAGGACCCACGT-AAGCCAGATGCCTGAGG-GGCACATGCATCTGGAGTTC
Dip_a_Y11(10) : -GGCTGGAGAAATGGCTTAGCGGTTAAGGCGTTTGCCTGCGAAGCCAAAGGATCCCGGT-------TCGATTCTCCAAGACCCACGT-AAGCCAGATGCACAAGG-GGCACATGCGTCTGGAGTTC
Dip_a_Y12(9) : -GGCTGGAGAAATGGCTTAGCGGTTAAGGCGTTTGCCTGCGAAGCCAAAGGATCCCGGT-------TCGATTCTCCAGGACCCATGT-AAGCCAGATGCACAAGG-GGCACATGCATCTGGAGTTC
Dip_a_Y13(9) : -GGCTGGAGAGATGGCTTAGTGGTTAA-ACGCTTGCCTGTGAAGCCTAAGGACCCAGGTTCAAGGCTCTATTCTCCAGGACCCACGT-AACCCAGATGCACAAGGGGGCATATGCATCTGGAGTTC
Dip_a_Y14(9) : -GGCTGGAGAAATGGCTTAGTGGTTAAGGCATTTGCCTTCAAAGTCAAAGGATCCCGGT-------TCGATTCTCCAGGACCCACGT-AAGCTAGATGCACAAGG-GGCACATGCATCTGGAGTTC
Dip_a_Y15(9) : -GGCTGGAGGAATGGCTTAGCGGTTAAGGCATCTGCCTGCAAAGCCAAAGGACCCTGGT-------TCGATTCCCCAGGACCCATGT-TAGCCAGATGCACAAGGGGGCACATGCGTCTGGAGTTC
Dip_a_Y16(8) : -GGCTGGAGAAATGGCTTAGTGGTTAAGGTGTTTGCCTGCGAAGCCAAAGGATCCCGGT-------TCGATTCTCCAGGACCCACGT-AAGCCAGATGCACAAGG-GGCACATGCATCTGGAGTTC
Dip_a_Y17(8) : GGGCTGGAGAAATGGCTTAGCGGTTAAGGCATTTGCCTGCGAAGCCAAAGGATCCCAGT-------TCGATTCTCCAGGACCCACGT-AAGCCAGATGCACAAGG-GGCACATGCATCTGGAGTTC
Dip_a_Y18(8) : -GGCTGGAGAGATGGCTTAGTGGTTAA-GTGCTTGCCTGAGAAGCCTAAGGACCCCGGTTCGAGGCTCAATTCCCCAGTACCCATGT-AAGCCAGATGCACAAGGTGGCGCATGCATCTGGAGTTC
Dip_a_Y19(7) : -GGCTGGAGAGATGGCTTAGCGGTTAAGGCGTTTGCCTGCGAAGCCTAAGGACCCAGGT-------TCGATTCTCCAGGTCCCACGT-AAGCCAGATGCACATGGTGTCATATGCATCTGGAGTTC
Dip_a_Y20(7) : -GGCTGGAGAGATGGCTTAGTGGTTAAGGCGTATGCCTATGAAGCCTAACGACCCAGGT-------TCGATTCTCCATGTCCCACAT-AAGCCAGATGCACATGGTGGCACATGCGTCTGGAGTTC
Dip_a_Y21(7) : -GGCTGGAGAGATGGCTTAGCGGTTAAGGCGTTTGTCTGTGAAGCCTAAGGACCCAGGT-------TCGATTCTCCAGGTCCCACGT-AAGCCAGATGCACAAGGGGGCACATGCGTCTGGAGTTC
Dip_a_Y22(6) : -GGCTGGAGAGATGGCTTAGTGGTTAAGGCATTTGCCTGTGAAGCCTAAGGACCCCRGT-------TCGATTCTCCAAGTCCCACGT-AAGCCAGATGCACATGGTGGCACATGCGTCTGGAGTTT
Dip_a_Y23(6) : -GGCTGGAGAGATGGCTTAGTGGTTAAGGCRTTTGCCTGCGAAGCCAAAGGATCCCGGT-------TCAATTCTCCAGGACCCACGT-AAGCCAGATGCACAAGG-GGCACACGCATCTGGAGTTC
Dip_a_Y24(6) : GGGCTGGAGAAATGGCTTAGTGGTTAAGGCRTTTGCCTGCAAAGCCAAAGGATCCCGGT-------TCGATTCTCCAGGACCCACGT-AAGCCAGATGCACAAGG-GGCACATGCATCTGGAGTTC
Dip_a_Y25(5) : -GGCTGGAGAAATGGCTTAGCRGTTAAGGCATTTGCCTGTGAAGCCAAAGGACCCAGGT-------TCAATTCTCCAGGACCCATGT-AAGCCAGATGCACAAGG-GGCACATGCATCTGGAGTTC
Dip_a_Y26(4) : -GGCTGGAGAGATGGCTTAGCTGTTAAGGCACTTGCCTGCAAAGCCAAAGGACCCAGGT-------TCAACTCCCCAGGACCCACAT-AAGCCAGATGCACAAGTGGGCGCATGCATCTGGAGTTC
Dip_a_Y27(4) : -GGCTGGAGAGATGGCTTAGCGGTTAAGGCACATGCCTGTGAAGCCTAAGGACCCAGGT-------TCGATTCTCCAGGTCCCACGT-AAGCCAGATGCACATGGTGGCGCATGCGTCTGGAGTTC
Dip_a_Y28(5) : -GGCTGGAGAGATGGCTTAGTGGTTAAGGCATTTGCCTGTGAAGCCTAAGGACCCTGGT-------TCGATTCTCCAGGTCCCACGT-AAGCCAGATGCACATGGTGGCACATGCATCTGGAGTTC
Dip_a_Y29(5) : -GGCTGGAGGAATGGCTTAGCGGTTAAGGTGTCTGCCTGCAAAGCCAAAGGATCCCGGT-------TCGATTCCCCAGGACCCACGT-TAGCCAGATGCACAAGGGGGCACATGCGTCTGGAGTTC
Dip_a_Y30(5) : -GGCTGGAGAAATGGCTTAGCGGTTAAGGCGTTTGCCTACAAAGCCAAAGGATCCCGGT-------TCAATTCTCCAGGACCCACAT-AAGCCAGATGCACGAGG-GGCACATGCATCTGGAGTTC
Dip_a_Y31(4) : -GGCTGGAGAGATGGCTTAGCGGTTATGGCGTTTGCCTGCAAAGCCAAAGGATCCYGGT-------TCGACTCTCCAGGACCCATGT-AAGCCAGATGCACAAGGGGGCACATGCATCTGGAGTTC
Dip_a_Y32(4) : -GGCTGGAGAAATGGCTTAGTGGTTAAGGCATTTGCCTGCGAATCCAAAGGATCCCGGT-------TCGATTCTCCAGGACCCACGT-AAGCCAGATGCACAAGG-GGCACATGCATCTGGAGTTY
Dip_a_Y33(3) : GGGCTGGAGAGATGGCTTAGC-GTTAAGGCATTTGCCTGCAAAGCCAAAGGATCCCGGT-------TTGACTCTCCAGGACCCACGT-AAGCCAGATGCACAAGGGGGCGCATGCATCTGGAGTTC
* 140 * 160 * 180 * 200 * 220 *
Dip_a : GTTTGCAGTGGCTGGAGGCCCCTGGCGTGCCCATTCTCTCTCTCTCTCTCT--------------CTCTCTC-TCTCTCT-CTCTCAAATAAATAAATAAAAAAAAA---
Dip_b : GTTTGCAGTGGCTGGAGGCCCCTGGTGCGCCCATTCTCTCTCTCTCTCTCTCT-----------CTCTCTC-TCTGTCT-CTCTCAAATAAATAAATAAAAAAAAA---
Dip_a_Y1(1789) : GTTTGCAGTGGCTGGAGGCCCCTGGCGCACCCATTCTCTCTCTCTCTCTCTCTCTCCTGCCTATTTCTGTA----TCTCTCT-CTCTCAAATAAATAAATAAATAAAAAT---
Dip_a_Y2(121) : GTTTGCAGTGGCTAGAGGCCCCTGGCGYGCCCATTCTCTCTCTCTCTCTCT-ACCTGCCTATTTCTGTA--TCTCTCT-CTCTCAAATAATAAATAAATAAAAATAAAA
Dip_a_Y3(56) : GTTTGGG--------------------TGCCCATTCTCTCTCT--------ACCTGCCTATTTCTGTA-------TCTCT-CTCTCAAATAAATAAATAAAAATAAAT---
Dip_a_Y4(43) : GTTTGCAGTGGCTGGAGGCCCCTGGCGTGCCCATTCTCTCTCTGTCTGCCTCTCTTCTA-----TCTCTCTC-TCTCTCTGCTTGCAAATAAATAAATAAAAAAAA-----
Dip_a_Y5(32) : GTTTGCAGTGGCTAGAGGCCCCTGGCGTGCCCATTCTCTCTCTGTCTGC-GCTCT---------TCTATCTC-TCTCTCTGCTTGCAAATAAATAAATAAAAATTAAA---
Dip_a_Y6(28) : GTTTGCAGTGGCTAGAGGCCCCTGGTGTGCCCATTCTCTCTGTGTCTGCCTCTCTTCTTC-TTCTCTCTCTC-TCTCTCTGCTTGCAAATAAATAAATA------------
Dip_a_Y7(21) : GTTTGCAGTGGCTGGAGGCCCCTGGTGTGCCCATTCTCT-TCTTTCTCTCT-ACCTGCCTATTTCTGTATCTCCCTCTCTCTCTCAAATAAATAAATAAAAATAAAA---
Dip_a_Y8(17) : GTTTCCAGTGGCTGGAGGCCCCTGGCACACCCATTCTTTCTCTCTCTCTCTCTCTC-------TCTTGTTTC-TTTCCCT-CTCTCAAATAAATAAGTAAAATAAAA---
Dip_a_Y9(14) : GTTTGCAGTGGCTAGAGGCCCCTGGCGTGCCCATTCTCTCTCTGTCTGCCTCTCTTCTT----TCTCTCTC-TCTCTCT-TTTGCAAATAAATAAATAAAAATTTTA---
Dip_a_Y10(13) : GTTTGCAGTGGCTAGAGGCCCCTGGCGTGCCCATTCTCTCTCTGTCTGCCTCTCTTCTC-----TCTCTCTC-TCTCTCTGCTTGCAAATAAATAAATAAATAAAAG---
Dip_a_Y11(10) : GTTTGCAGTGGCTAGAGGCCCCTGACGTGCCCATTCTCTCTCTGTCTGCCTCTCTTCT----------TCTC-TCTCTCTGCTTGCAAATAAATAAAATAAAAA--------
Dip_a_Y12(9) : ATTTGCAGTGGCTAAAGGCCCCTGGCATGCCCATTCTCTCTCTCTGTCTGCCTCTCA-------TCTATCTCATCTCTCTGCTTGCAAATAAATAAATAAAATAAAA----
Dip_a_Y13(9) : ATTTGCA--------------------TGCCCATTCTCTCTCT--------ACCTGCCTATTTCTGTA-TC-TCT-CTCTCAAATAAATAAATAAAAATATAA-------
Dip_a_Y14(9) : GTTTGCAGTGGCTAGAGGCCCCTGGCGTGCCCATTCTCTCTCTGTCTGCTGCTCT---------TCTATCTC-TCTCTCTGCTTGCAAATAAATAAATAAAAATTAAA---
Dip_a_Y15(9) : GTTTGCAGTGGCTGGAGGCCCCTGGTGTGCCCATTCTCTCTCTCTCTCTCC--------------------C-TCTTTCT-CTGTCAAATAAATTAATAAAAAAAAA---
Dip_a_Y16(8) : GTTTCAAGTGGCTAAAGGCCCCTGGCGTGCCCATTCTCTCTCTCTCTCTCTCTCTCTCYC-TCTCTTCTATC-TCTCTCTGCTTGCAAATAAATAAATAAAATTAAAA---

```
Dip_a_Y17(8)    : RTTTGCAGTGGCTAGAGGCCCTGGCATGCCCATTCTCTCTCTGTCTGCCTCTCTTCTAT-CTCTCTCTCTC-TCTCTCTGCTTGCAAATAAATAAATAATTTAAAA----
Dip_a_Y18(8)    : ATTTGCAGTGGCTGGAGGCCCTGGTGTGCCCATTCTCTCTCTCTCTTCCTCTTCT-------CTCTCTCTC-TCTGTCR-CTCTCAAATAAATAAATAATAAAAAAA---
Dip_a_Y19(7)    : GTTTGCAGTGGCTAGAGGCCCTGGTGTGCCCATTCTCACTCCCTGCCTGT--------------CTCTTCT-CTTCTCT-GTCTCTAATAAATAAATAAAAATAAAA---
Dip_a_Y20(7)    : GTATGCAGTGGCTAGAGGCCCTGGTGTGCCCATTCTCACTATCTCTCTGTCT----------------TCCC-TCTCTCT-ATCTCAAATAAATAAATAAAATAAAAT---
Dip_a_Y21(7)    : GTTTGCAGTGGCTAGAGGCCCTGGCGC-CCCATTCTCTCTCTCTTCTCTCTCTATCT----CTCTTTCCC-TCTCTCT-GTCCCTAATAAATAAATAAAAATAAAA---
Dip_a_Y22(6)    : GTTTGCAGTGGCTAGAGGCCCTGGCGTGCCCATTCTCACTCTCTCTCTTT------------CTCTCCCCC-TCTCTCT-GTCTCTAATAAATAAATAAATAAAAATAAA
Dip_a_Y23(6)    : GTTTGCAGTGGCTAGAGGCCCTGGCGTGCCCAT--TCTCTCTGTCTGCCTCTCTTCTA-----TCTMTCTC-TCTCTCTGCTTGCAAATAAATAAATTAATTWAAAA---
Dip_a_Y24(6)    : GTTTGCAGTGGCTAGAGGCCCTGGCRTGCCCATTCTCTCTCTGTCTSYCYCTCTYY-------TMTCTCTC-TCTCTCTGCTTGCAAATAAATAAATAAATATAATT---
Dip_a_Y25(5)    : GTTTGYAGTGGCTAGAGGCCCTGGCATGCCCATTCTSTCTCTGTCTSYCTCTCTCC-------TCTCTCTC-TCTCTCTGCTTGCAAATAAATAAATAAAAAA-------
Dip_a_Y26(4)    : GTTTGCAGTGGCTGGAGCCCCTGGCGTGCCCATTCTCTCTCTCTCTCTCTCTCTCCT-TCCTGCCTAGT-TCTGTCT-CTCTCAAATAAATAAATAAAAATAAAA---
Dip_a_Y27(4)    : GTTTGCAGTGGATAGAGGCCCTGGTGTGCCCATTCACACTCTCTCTCTCC------------------CCCA-GCTCTGT-ATCTCTAATAAATAAATAAAAATTAAA---
Dip_a_Y28(5)    : GTTTGCAGTGGCTAGAGGCCCTGGCATGCCCATTCTCACTCTCTCTCTCTT-----------CTCTCCCCC-TCTCTCT-GTCTCTAATAAATAAA-------------
Dip_a_Y29(5)    : ATTTGCAGTGGCTGGAAGCCCTGGCGTGCCCATTCTCTCTCTCCC---------------------------TCTTTCT-CTGTCAAATAAATTAATAAAAATAAAA---
Dip_a_Y30(5)    : GTTTGCAGTGGCTAGAGGCCCTGGC-------CTCTCTCTGTGTCTGCCTCTCT--------------TCTA-TCTCTCTGCTTGCAAATAAATAAATTAAAATTAAA---
Dip_a_Y31(4)    : ATTTGCAGTGGCTGGAGGCCCTGGTGTGCCCATTCTTTCTCTCTCTTTCT------------GTGTCTTTC-TCTCTCT-GAAATAAATAAATAAATWAAATAWAAA---
Dip_a_Y32(4)    : GTTTGCAGTGGCTAGAGGCCCTGGCRTGCCCAT--TCTCTCTGTCTGACTCTCTTC-------TATCTTTC-TCTCTCTGCTTGCAAATAAATAAATAAATAWWTW----
Dip_a_Y33(3)    : GTTTGCAGTGGCTGGAGGCCCTGGTGTGCCCATTCTCTCTCTCTCTCT--------------CTCTCTCTC-TCTTTCT-CTCTTAAATAAATAATAAATAAATAAA---
```

**Figure S15**. Consensus sequences of 33 tribes of young Dip_a copies from the *A. bullata* genome. The numbers of tribe members are indicated in brackets (in red). Specific 17-bp insertion in the TC-motif of Dip_a_Y1, Y2, Y3, Y7, and Y13 is highlighted in yellow.
