# Supplementary material for: Analysis of SINE Families B2, Dip, and Ves with Special Reference to Polyadenylation Signals and Transcription Terminators
Source: Int J Mol Sci. 2021 Sep 13;22(18):9897. doi: 10.3390/ijms22189897 (PMC8466645; doi:10.3390/ijms22189897)
Supplement: Supplementary file 1 [file Fig S17.pdf › Supplementary Vassetzky/SINE Dip. Figs S7-S22. Tables S6-S8/Fig S17.pdf]

```
                          *        20         *        40         *        60         *        80         *       100         *       120
Dip_b1        : GGGCTGGAGAGATGGCTTAGCGGTTAAGGCGCCTGCCTGCTAAGCCTAAGAACGCAKGTTCCAATCT----------------CCAGGTCCCACGTTTAGCCAGATGCAAAGTGACGCAAGGGCGCAAT
Dip_b2        : GGGCTGGAGAGATGGCTTAGCGGTTAAGGCGCCTGCCTCCTAAGCCTAAGAACGCTGGTTCCAATCTCCAGCGGTTCCAATCTCCAGGTCCCACGTTTAGCCAGATGCAAAGTGACGCAAGGGCGCAAT
Dip-b_Y1(108) : -GGCTGGAGAGATGGCTTAGCGGTTAAGGCGCCTGCCTCCTAAGCCTAAGAACGCTGGTTCCAATCTCCAGCGGTTCCAATCTCCAGGTCCCACGTTTAGCCAGATGCAAAGTGACGCAAGGGCGCAAT
Dip-b_Y2(27)  : -GGCTGGAGAGATGGCTTAGCGGCTAAGGCGCCTGCCTGCTAAGCCTAAGAACGCTGGTTCCAATGTCCAGCGGTTCCAATCTCCAGGTCCCACGTTTAGCCAGATGC--AGTGAGGCAAGGGCGCAAT
Dip-b_Y3(15)  : GGGCTGGAGAGATGGCTTAGCGGTTAAGGCGCCTGCCTGCTAAGCCTAAGAACCC-GGTTCCAATCT----------------CCAGGTCCCACGTTTAGCCAGATGCAAAGTGACGCAAGGGCGCAAT
Dip-b_Y4(11)  : GGGCTGGAGAGATGGCTTAGCGGTTAAGGCGCCTGCCTGCTAAGCCTAAGAACGCTGGTTCCAATCTCCAGCGGTTCCAATCTCCAGGTCCCACGTTTAGCCAGATGCAAAGTGACGCAAGGGCGCAAT

                *        140         *        160         *        180         *        200         *        220         *        240         *        2
Dip_b1        : GCCGCACTTGCCCACAAGGGGGCGCACGCATCTGGAGTTCGTTCACAGCAGCTGAGAGGCCGCTGGCGCGCCCA-------------------------------------------------------
Dip_b2        : GCCGCACTTGACCACAAGGGGGCGCACACATCTGGATTTCGTTCACAGCAGCTGAGAGACTGCTGGCGCGCCCA-------------------------------------------------------
Dip-b_Y1(108) : GCCGCACTTGACCACAAGGGGGCGCACGCATCTGGATTTCGTTCACAGCAGCTGAGAGACTGCTGGCGCGCCCATCTCTT----CTTCTCCCTCTCCTCTC------TTCTCTCTCTGTGTCTCTCTCC
Dip-b_Y2(27)  : GCCGCACTTGCCCACAAGGGGGCGCAC-CATCTGGATTTCGTTCACAGCAGCTGAGAGACTGCTGGCGCGCCCATCTCTGTCTCCCTCTCCCTCTCTGTCT------ATCTCTCTCTGTCTCTCTCTCC
Dip-b_Y3(15)  : GCCGCACTTGACCACAAGGGGGCGCACACATCTGGATTTCGTTCACAGCAGCTGAGAGACTGCTGGCGCGCCCATCTCTATCTGTCTCCCTCTCCCTCTCTTCTGTCTTCTCTCTCTGTGTCTCTCTCC
Dip-b_Y4(11)  : GCTGCACTTGCCCACAAGGGGGCGCACGCATCTGGATTTCGTTCACAGCAGCT--GAGACTGCTGGCGCGCCCATCTCTGTCT-CCTCTCCCTCTCTGTCT------ATCTCTCTCTGTGTCTCTCTCC

                60         *        280
Dip_b1        : ------------------------
Dip_b2        : ------------------------
Dip-b_Y1(108) : AAAAATAAATAAAAAAAAAAAAAA
Dip-b_Y2(27)  : AAAAATAAATAAAAAAAAAAAAAA
Dip-b_Y3(15)  : AAAAATAAAAAAAAATAAAAAAAA
Dip-b_Y4(11)  : AAAAATAAATAAAATAATTAAAA
```

Figure S17. Consensus sequences of four tribes of young Dip_b copies from the *A. bullata* genome. Numbers of tribe members are indicated in brackets (in red).
